# Real-world adherence to, and persistence with, once- and twice-daily oral disease-modifying drugs in patients with multiple sclerosis: a systematic review and meta-analysis

**DOI:** 10.1186/s12883-020-01830-0

**Published:** 2020-07-16

**Authors:** Jacqueline A. Nicholas, Natalie C. Edwards, Roger A. Edwards, Anna Dellarole, Megan Grosso, Amy L. Phillips

**Affiliations:** 1 OhioHealth Multiple Sclerosis Center, Columbus, OH USA; 2 Health Services Consulting Corporation, Boxborough, MA USA; 3 Fair Dynamics Consulting, Milan, Italy; 4 EMD Serono, Inc. (an affiliate of Merck KGaA, Darmstadt, Germany), Rockland, MA USA

**Keywords:** Adherence, Dimethyl fumarate, Discontinuation, Fingolimod, Meta-analysis, Persistence, Real-world, Teriflunomide

## Abstract

**Background:**

Nonadherence to disease-modifying drugs (DMDs) for multiple sclerosis (MS) is associated with poorer clinical outcomes, including higher rates of relapse and disease progression, and higher medical resource use. A systematic review and quantification of adherence and persistence with oral DMDs would help clarify the extent of nonadherence and nonpersistence in patients with MS to help prescribers make informed treatment plans and optimize patient care.

The objectives were to: 1) conduct a systematic literature review to assess the availability and variability of oral DMD adherence and/or persistence rates across ‘real-world’ data sources; and 2) conduct meta-analyses of the rates of adherence and persistence for once- and twice-daily oral DMDs in patients with MS using real-world data.

**Methods:**

A systematic review of studies published between January 2010 and April 2018 in the PubMed database was performed. Only studies assessing once- and twice-daily oral DMDs were available for inclusion in the analysis. Study quality was evaluated using a modified version of the Newcastle-Ottawa Scale, a tool for assessing quality of observational studies. The random effects model evaluated pooled summary estimates of nonadherence.

**Results:**

From 510 abstracts, 31 studies comprising 16,398 patients with MS treated with daily oral DMDs were included. Overall 1-year mean medication possession ratio (MPR; *n* = 4 studies) was 83.3% (95% confidence interval [CI] 74.5–92.1%) and proportion of days covered (PDC; *n* = 4 studies) was 76.5% (95% CI 72.0–81.1%). Pooled 1-year MPR ≥80% adherence (*n* = 6) was 78.5% (95% CI 63.5–88.5%) and PDC ≥80% (*n* = 5 studies) was 71.8% (95% CI 59.1–81.9%). Pooled 1-year discontinuation (*n* = 20) was 25.4% (95% CI 21.6–29.7%).

**Conclusions:**

Approximately one in five patients with MS do not adhere to, and one in four discontinue, daily oral DMDs before 1 year. Opportunities to improve adherence and ultimately patient outcomes, such as patient education, medication support/reminders, simplified dosing regimens, and reducing administration or monitoring requirements, remain. Implementation of efforts to improve adherence are essential to improving care of patients with MS.

## Background

Multiple sclerosis (MS) is a progressive, inflammatory, autoimmune, neurodegenerative disease of the central nervous system that often begins in early adult life. Guidelines recommend that clinicians should offer disease-modifying drugs (DMDs) to people diagnosed with relapsing forms of MS (RMS) [1, 2]. DMDs have been shown to reduce the rate of relapse, slow the rate of disease progression, [3–6] and improve long-term outcomes for patients with RMS [7, 8]. It is also recommended that clinicians monitor for medication adherence, adverse events, tolerability, safety, and effectiveness of the therapy in people with MS on DMDs [1].

Medication adherence and persistence are challenging for patients with MS [9, 10]. Nonadherence to or nonpersistence with DMD therapy for MS has been associated with poorer clinical outcomes, including higher rates of relapse and disease progression, and higher medical resource use [9, 11–14]. Although it has been hypothesized that the oral route of DMD administration may offer improved adherence to the injectable route of administration, recent studies have reported that real-world adherence to and persistence with the once- and twice-daily oral maintenance DMDs (i.e., fingolimod, dimethyl fumarate, and teriflunomide) may be similar to that of self-injectable DMDs [11, 15–18].

A systematic review and quantification of the real-world adherence to and persistence with oral DMDs would help clarify the extent of nonadherence and nonpersistence in patients with MS. Kantor et al. 2018 conducted a systematic review and meta-analysis of real-world persistence with fingolimod in patients with relapsing-remitting MS (RRMS) and reported a consensus 1-year persistence rate of 82% (95% confidence interval [CI] 79–85%) [19]. The current study aimed to expand on this previous meta-analysis to include other daily oral DMDs and to evaluate both adherence and persistence with oral DMDs. Specifically, the objectives of this study were to: 1) conduct a systematic literature review to assess the availability and variability of oral DMD adherence and/or persistence rates across ‘real-world’ data sources; and 2) conduct meta-analyses of the rates of adherence and persistence for all currently available oral DMDs in patients with MS.

## Methods

### Systematic literature review

A systematic literature search was performed of all studies published between January 2010 and April 2018 in the PubMed database that evaluated adherence or persistence to oral DMDs. The search strategy used the following terms: (Aubagio OR cladribine OR dimethyl fumarate OR fingolimod OR Gilenya OR Tecfidera OR teriflunomide OR oral OR disease modifying drug OR DMD OR disease modifying therapy OR DMT) AND multiple sclerosis AND (adherence OR compliance OR persistence OR discontinuation). A priori exclusion criteria were: lack of primary data; lack of primary real-world DMD adherence/persistence data; lack of oral DMD adherence/persistence data; pediatric studies; non-English studies; and abstract-only available. Two reviewers independently reviewed the search results and reference lists of selected articles to identify additional appropriate studies and carried out data extraction. Full search strategy and search results are provided in Additional File 1.

Information extracted from the screened articles included the type of study/data source; study population (baseline demographic and clinical characteristics); treatment arms; duration of follow-up after DMD initiation; sample size; outcomes evaluated (including definition of adherence and method of measurement); clinical results; secondary results; and strengths and limitations of the studies.

The quality of selected studies was evaluated using a modified version of the Newcastle-Ottawa Scale (NOS), a tool for assessing the quality of observational studies [20]. The NOS was modified as it was primarily designed to evaluate case-control and comparative studies. Study quality was evaluated with the modified NOS from the perspective of study design and patient selection as well as outcome to generate an overall assessment of the quality of the studies and their internal validity. Three criteria were evaluated under the NOS ‘study design and patient selection’ perspective: ascertainment of the intervention/validity of study design, patient selection, and outcome not present at the start of the study. Criteria evaluated under the ‘outcome evaluation’ perspective were appropriateness of the measure of adherence/persistence, adequacy or appropriateness of duration of follow-up, and thoroughness of follow-up for all patients. Each criterion was given a full-, partial-, or poor-quality score (Table 1). Per Cochrane Collaboration [21] and other recommendations [22] that meta-analyses not be adjusted on the basis of quality, the results of the study quality assessment were presented as standard tables and systematic narrative description and commentary about each of the elements.

**Table 1:** Study quality as evaluated using a modified version of the Newcastle-Ottawa Scale

Reference	Study design and patient selection			Outcome evaluation		
	Ascertainment of intervention/validity of study design	Patient selection	Outcome not present at start	Appropriate measure of adherence/persistence	Adequate/appropriate duration of follow-up	All patients accounted for followed up
Lanzillo R, et al. *J Neurol.* 2018;265:1174–1183	◉	●	●	●	●	●
Ferraro D, et al. *Curr Med Res Opin*. 2018;34:1803–1807	◉	●	●	●	●	●
Granqvist M, et al. *JAMA Neurol.* 2018;75:320–327	●	●	●	●	●	●
Hua LH, et al. *Mult Scler.* 2018;1,352,458,518,765,656	◉	◉	●	●	◉	●
Eriksson I, et al. *Eur J Clin Pharmacol.* 2018;74:219–226	●	●	●	●	●	●
Williams MJ, et al. *Curr Med Res Opin.* 2018;34:107–115	●	◉	○	●	●	●
Ernst FR, et al. *Curr Med Res Opin.* 2017;33:2099–2106	◉	●	●	●	◉	●
Lattanzi S, et al. *J Neurol.* 2017;264:2325–2329	◉	●	●	●	●	●
Gerber B, et al. *Mult Scler Relat Disord.* 2017;18:218–224	●	◉	○	●	●	●
Zimmer A, et al. *Patient Prefer Adherence.* 2017;11:1815–1830	◉	●	◉	◉	◉	●
Hersh CM, et al. *Mult Scler J Exp Transl Clin.* 2017;3:2055217317715485	◉	●	●	●	●	●
Vollmer B, et al. *Mult Scler J Exp Transl Clin.* 2017;3:2055217317725102	◉	◉	◉	●	◉	●
Johnson KM, et al. *J Manag Care Spec Pharm.* 2017;23:844–852	●	◉	●	●	●	●
Smoot K, et al. *Mult Scler.* 2017;1,352,458,517,709,956	●	●	●	●	●	●
Burks J, et al. *Clinicoecon Outcomes Res.* 2017;9:251–260	●	◉	○	●	●	●
Munsell M, et al. *Patient Prefer Adherence.* 2016;11:55–62	●	◉	○	●	●	●
Hersh CM, et al. *Mult Scler Relat Disord.* 2016;10:44–52	◉	◉	●	●	●	●
Zhovtis L, et al. *Ther Adv Neurol Disord.* 2016;9:454–461	◉	●	●	●	●	●
Nazareth T, et al. *BMC Neurol.* 2016;16:187	●	●	●	◉	●	●
Wicks P, et al. *BMC Res Notes.* 2016;9:434	◉	●	●	●	○	◉
Warrender-Sparkes M, et al. *Mult Scler.* 2016;22:520–532	●	◉	●	●	◉	●
Lapierre Y, et al. *Can J Neurol Sci.* 2016;43:278–283 (29)	◉	○	◉	●	●	●
Braune S, et al. *J Neurol*. 2016;263:327–333	●	●	●	●	●	◉
Frisell T, et al. *Mult Scler.* 2016;22:85–93	●	◉	●	●	●	●
Longbrake EE, et al. *Mult Scler J Exp Transl Clin.* 2016;2	◉	◉	●	●	◉	●
He A, et al. *JAMA Neurol.* 2015;72:405–413	●	●	●	◉	●	●
Hersh CM, et al. *Int J Neurosci.* 2015;125:678–685	◉	●	●	●	◉	●
Bergvall N, et al. *J Med Econ*. 2014;17:696–707	●	◉	○	●	●	●
Al-Hashel J, et al. *CNS Drugs*. 2014;28:817–824	◉	●	●	◉	◉	●
Agashivala N, et al. *BMC Neurol.* 2013;13:138	●	◉	○	●	●	●
Ontaneda D, et al. *J Neurol Sci*. 2012;323:167–172	◉	◉	●	●	○	●

**Abbreviations:***RRMS* relapsing-remitting multiple sclerosis, ● full-quality score, ◉ partial-quality score, ○ poor-quality score

For the **ascertainment of the intervention/validity of study design** criterion, if the study was a medical chart review, evidence that there was an effort made to validate reported data resulted in a full-quality score. Otherwise, the medical chart review or registry study was assigned a partial-quality score. Prospective cohort studies and administrative claims database evaluations were given a full-quality score for this criterion

For the **patient selection** criterion, studies were given a full-quality score if the patients were well characterized (i.e., age, sex, region, duration of MS diagnosis, MS severity, prior treatments, current treatment) and were representative of patients with RRMS. Studies were not penalized for including selected populations or for only evaluating a single center because it was felt that these studies were still valid cohort studies. Studies were given a poor-quality score if the patient population was not well-characterized and was not representative of patients with RRMS

For the **outcome of interest not present at the start of the study** criterion, studies were given a full-quality score if they attempted to capture patient prescription abandonment and thoroughly described how it was ascertained. Administrative claims database analyses were not able to ascertain this, and were therefore given a poor-quality score for this criterion

For the **appropriate measurement of adherence/persistence** criterion, studies with a full-quality score had to appropriately define and measure adherence and persistence and include and/or delineate switching for discontinuation

The **adequate/appropriate duration of follow-up** criterion required full-quality studies to measure adherence/persistence over 1 year as this was the most common time horizon used and enabled comparability

The **all patients accounted at follow-up** criterion required that all patients evaluated were followed up throughout the study and did not have missing data

### Meta-analyses

The selection of endpoints was driven by the availability of data in the published, peer-reviewed literature. Adherence was evaluated using either the medication possession ratio (MPR) or the proportion of days covered (PDC). MPR was calculated as the total number of days of medication supply between the first prescription claim and the last prescription claim issued during the follow-up period divided by the total number of days in the follow-up period. A variable follow-up period was used for the MPR denominator (number of days between the index date and the last prescription dispensed inclusive of supply) [23]. PDC was calculated as the total number of days in the follow-up period in which medication was available (excluded overlapping days’ supply) divided by the duration of the observation period (i.e., 1 year in the case of oral DMD adherence studies). Adherence was calculated as means (mean MPR and mean PDC) and the proportion of patients attaining the 0.8 threshold, which is commonly considered an acceptable level of adherence [24]. Discontinuation was defined as the proportion of patients who either switched DMDs or discontinued DMD medications altogether. Analyses were conducted for 5 separate endpoints over a 1-year follow-up period: (1) mean MPR for patients overall (2); mean PDC for patients overall (3); proportion of patients ‘adherent’, defined as proportion with MPR ≥80% (4); proportion of patients ‘adherent’, defined as proportion with PDC ≥80%; and (5) proportion of patients who discontinued the initial treatment.

In line with published recommendations regarding the use of real-world data in meta-analyses, [25] statistical heterogeneity was evaluated using Cochran’s Q test and the *I*^*2*^ statistic, which provide a measure of the presence of heterogeneity and the share of dispersion across studies, respectively [26]. If Q was significant and *I*^*2*^ was > 50%, the random-effects model (REM) was used to calculate pooled summary estimates; otherwise, a fixed-effect model was used. The studies were weighted according to the extent of variation among the intervention effects. Egger’s test was used to detect publication bias [27]. The ‘metaprop’ and the ‘metamean’ functions in the R statistical programming language [28] were used to conduct the meta-analysis, and a *p*-value of < 0.05 was considered statistically significant. Subgroup analyses conducted included study location (i.e., US/ex-US studies) and study design (i.e., prospective cohort vs. retrospective chart review vs. administrative claims database evaluation). The impact of any single study on the overall results was assessed using a leave-one-out sensitivity analysis, in which each study was iteratively removed and the findings compared to the overall meta-analysis.

## Results

### Systematic literature review

**Fig. 1:**
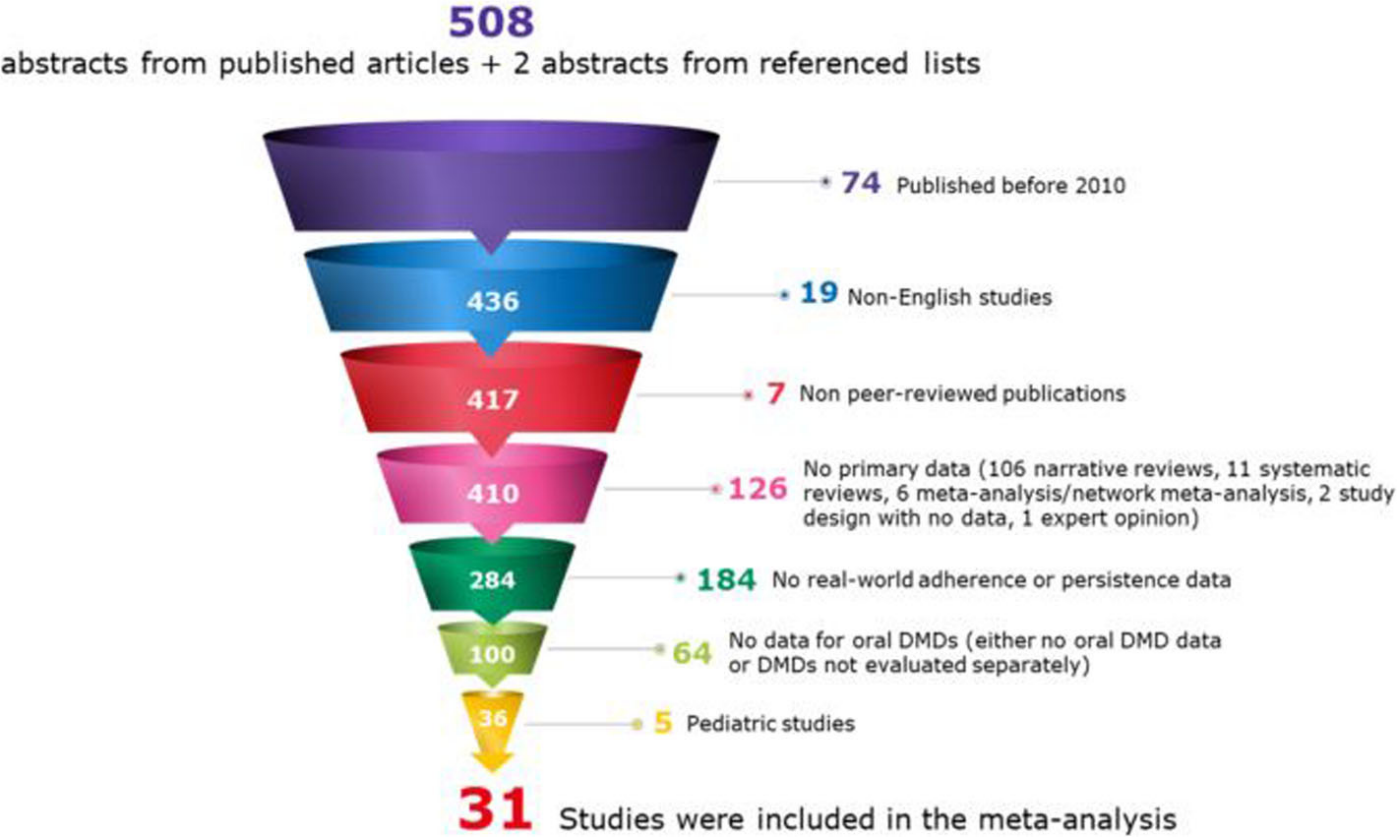
Study selection flowchart. **Abbreviations:***DMD* disease-modifying drug

From a total of 510 abstracts identified, 31 studies comprising 16,398 patients were included in the systematic review after applying exclusion criteria (Fig. 1). Table 2 provides information about the individual studies. No studies evaluating cladribine tablets were identified due to its recent approval, therefore the analyses focused on once- and twice-daily oral maintenance DMDs. Siponimod was not available at the time of the study and is not included in the analyses.

**Table 2:** Studies included in the systematic review (*n* = 31) [11, 12, 15–18, 29–53]

Reference	Study design	Geographical area	Sample size for oral DMD(s) studied	Study population	Outcomes evaluated
Agashivala N, et al. *BMC Neurol*. 2013;13:138	Retrospective administrative claims database evaluation	USA	Fingolimod (*n* = 248)	Patients with MS	Mean MPR and PDC; MPR ≥80%; PDC ≥80%; discontinuation rate
				mean age 46.4 (10.7) years;	
				79.0% female;	
Al-Hashel J, et al. *CNS Drugs.* 2014;28:817–824^a^	Retrospective evaluation of patient MS registry	Kuwait	Fingolimod (*n* = 175)	Patients with RRMS	% discontinuation
				mean age 33.3 ± 9.2 years;	
				75.4% female	
Bergvall N, et al. *J Med Econ.* 2014;17:696–707	Retrospective administrative claims database evaluation	USA	Fingolimod (*n* = 889)	889 patients (age range NS) with MS initiating fingolimod	MPR ≥80%; PDC ≥80%; discontinuation rate
Braune S, et al. *J Neurol.* 2016;263:327–333	Prospective, observational, multi-center cohort study	Germany	Fingolimod (*n* = 99)	Patients with RRMS (age range NS) with failure of earlier therapy with injectable DMT initiating fingolimod	Discontinuation rate
Burks J, et al. *Clinicoecon Outcomes Res.* 2017;9:251–260	Retrospective administrative claims database study	USA	*N* = 1018	Patients with MS (aged 18–65 years) initiating an oral DMD	Mean PDC; proportion of patients with PDC ≥80%; discontinuation rate
			Teriflunomide, fingolimod, DMF		
				mean age 44.41 (10.52); 72.1% female	
Eriksson I, et al. *Eur J Clin Pharmacol.* 2018;74:219–226	Prospective cohort which includes retrospective claims and other health-related data analysis	Stockholm county, Sweden	DMF (*n* = 400)	400 patients with RRMS (age range NS) initiating DMF	Discontinuation rate
				Mean age ranged from 35.3–40.5 years	
				61% previously treated.	
Ernst FR, et al. *Curr Med Res Opin.* 2017;33:2099-2106^a^	Retrospective medical chart review	USA	DMF (*n* = 307)	307 patients (aged ≥18 years) with RRMS initiating DMF	Discontinuation rate
				Mean age 46.6 ± 11.8 years;	
				77.9% female;	
Ferraro D, et al. *Curr Med Res Opin.* 2018;34:1803–1807	Prospective observational cohort study	Italy	*N* = 258	Patients with RRMS (age range NS) initiating oral DMD	Discontinuation rate
			Teriflunomide, DMF		
				mean age 43 years,	
				29.8% female	
Frisell T, et al. *Mult Scler.* 2016;22:85–93	Prospective, observational, multi-center cohort study	Sweden	Fingolimod (*n* = 876)	Patients with RRMS initiating fingolimod tx	Discontinuation rate
				mean (SD) age 38 (10) years;	
				67% females	
Gerber B, et al. *Mult Scler Relat Disord.* 2017;18:218–224	Retrospective administrative database evaluation	Alberta, Canada	*N* = 72	72 patients with MS (aged < 35–≥65 years) initiating an oral DMD	MPR ≥80%; discontinuation rate
			Fingolimod, teriflunomide, DMF		
				61.7% aged 35–55 years;	
				73.8% female	
Granqvist M, et al. *JAMA Neurol.* 2018;75:320–327	Retrospective medical chart review from MS registry	Sweden	*N* = 103	Patients with RRMS initiating DMD tx	Discontinuation rate
			DMF (*n* = 86), fingolimod (n = 17)	The median (interquartile) age 34.4 (27.4–43.4) years;	
				68% female	
He A, et al. *JAMA Neurol.* 2015;72:405–413	Matched retrospective analysis of data collected prospectively from an international, observational cohort study	International	Fingolimod (*n* = 148)	Patients with MS (age range NS) switching to fingolimod tx	Discontinuation rate
Hersh CM, et al. *Int J Neurosci.* 2015;125:678–685^a^	Retrospective, single-center medical chart review	Cleveland, OH, USA	Fingolimod (*n* = 306)	Patients with MS (age range NS) initiating fingolimod	Discontinuation rate
				3.5% treatment naïve and 24.0% had remote DMT use prior to fingolimod	
Hersh CM, et al. *Mult Scler J Exp Transl Clin*. 2017;3:2055217317715485^a^	Retrospective, single-center medical chart review	Cleveland, OH, USA	Fingolimod (*n* = 264), DMF (*n* = 396)	Patients with MS (age range NS) being treated with DMD for ≥1 year	Discontinuation rate
Hersh CM, et al. *Mult Scler Relat Disord.* 2016;10:44–52	Retrospective, single-center medical chart review	Cleveland, USA	Fingolimod (*n* = 317), DMF (*n* = 458)	Patients with MS initiating DMD tx	Discontinuation rate
				mean age DMF 47.1 ± 11.2 years fingolimod 43.9 ± 9.2 years;	
				RRMS DMF 73.5% fingolimod 81.7%	
Hua LH, et al. *Mult Scler.* 2018;1352458518765656^a^	Retrospective medical chart review	Cleveland, OH; Las Vegas, NV; Weston, FL, USA	Fingolimod (n = 10), DMF (*n* = 74), teriflunomide (n = 40)	Patients (aged over 60 years) with MS on DMD for ≥2 years	Discontinuation rate
Johnson KM, et al. *J Manag Care Spec Pharm.* 2017;23:844–852	Retrospective administrative claims database evaluation	USA	Fingolimod (*n* = 195), DMF (*n* = 1160), teriflunomide (*n* = 143)	Patients with MS (aged ≥18 years) initiating DMD tx	MPR, MPR ≥80%, mean PDC, PDC ≥80%, discontinuation rate
				Mean age range 44.4–53.2 years; 75.5–83.6% female	
Lanzillo R, et al. *J Neurol.* 2018;265:1174–1183	Retrospective medical chart review	Italy	*N* = 1312	Patients with RRMS (age range NS) initiating oral DMD	Discontinuation rate
			Teriflunomide, DMF		
				mean age 40.0 (11.2) year	
Lapierre Y, et al. *Can J Neurol Sci.* 2016;43:278–283^a^	Prospective, observational, multi-center cohort study	Canada	Fingolimod (*n* = 2399)	Patients with RRMS (age range NS) receiving fingolimod and participating in an education and support program	Discontinuation rate
				Mean age was 41.2 years (range 18–75.5); 75.2% were female.	
Lattanzi S, et al. *J Neurol.* 2017;264:2325–2329	Retrospective medical chart review	Italy	Fingolimod (*n* = 129), teriflunomide (*n* = 64), DMF (*n* = 114)	Patients with RRMS;	Discontinuation rate
				mean age was 41.2 (10.3) years, 66.5% female	
Longbrake EE, et al. *Mult Scler J Exp Transl Clin.* 2016;2^a^	Retrospective, single-center medical chart review	USA	Teriflunomide (*n* = 83), fingolimod (*n* = 92), DMF (*n* = 254)	Patients (age range NS) with relapsing forms of MS initiating oral DMD tx	Discontinuation rate
				mean age 39.8–49.4 years; 72.0–81.9% female	
Munsell M, et al. *Patient Prefer Adherence.* 2016;11:55–62	Retrospective administrative claims database evaluation	USA	*N* = 1175	Patients with MS (aged 18–64 years) initiating an oral DMD; mean age 44.9 years; 76.2% female	Mean MPR; proportion of patients with MPR ≥80%; discontinuation rate
			Teriflunomide, fingolimod, DMF		
Nazareth T, et al. *BMC Neurol.* 2016;16:187	Retrospective medical chart review	USA	Teriflunomide (n = 31), fingolimod (*n* = 55), DMF (*n* = 65)	Patients with MS (aged ≥18 years) initiating DMF;	Discontinuation rate
				Age range (mean ± SD); 44.2 ± 10.7 to 50.6 ± 9.6 years.	
Ontaneda D, et al. *J Neurol Sci.* 2012;323:167–172^a^	Retrospective, single-center medical chart review	Cleveland, OH, USA	Fingolimod (n = 317)	Patients with MS (age group NS) prescribed fingolimod	Discontinuation rate
Smoot K, et al. *Mult Scler.* 2017;1,352,458,517,709,956	Prospective registry at a single site	Oregon, USA	DMF (*n* = 417)	Patients (aged ≥18 years) with RMS initiating DMF tx;	Discontinuation rate
				mean age 49.4 ± 12.0 years	
Vollmer B, et al. *Mult Scler J Exp Transl Clin.* 2017;3:2055217317725102	Retrospective, single-center medical chart review	Colorado, USA	Fingolimod (*n* = 271), DMF (*n* = 342)	Patients with MS (age range NS) initiating DMD tx;	Discontinuation rate
				mean age range 42.5–45.8 years;	
				69.6–72.0% female	
Warrender-Sparkes M, et al. *Mult Scler.* 2016;22:520–532^a^	Prospective, observational multi-center cohort study	International	Fingolimod (*n* = 426)	Patients with CIS or early RRMS (age range NS) initiating fingolimod tx	Discontinuation rate
Wicks P, et al. *BMC Res Notes.* 2016;9:434^a^	Online community patient survey	USA	Fingolimod (*n* = 93), DMF (*n* = 188)	Patients (aged ≥18 years) with RRMS with current or past experience of fingolimod or DMF tx	Discontinuation rate
				mean age 46.2–51.8 years; % female 77.0–93.8%;	
Williams MJ, et al. *Curr Med Res Opin.* 2018;34:107–115	Retrospective administrative claims database evaluation	USA	DMF (*n* = 133)	Patients (aged ≥18 years) with MS initiating DMD tx	Mean MPR; MPR ≥80%; mean PDC; PDC ≥80%; discontinuation rate
Zhovtis Ryerson L, et al. *Ther Adv Neurol Disord.* 2016;9(6):454–461	Retrospective medical chart review in 2 tertiary MS clinics	New York/New Jersey, USA	DMF (*n* = 382)^b^	Patients (aged ≥18 years) with RRMS initiating DMF	Discontinuation rate
				range across subgroups: mean age 42.5–47.4 years, 71–83% female	
Zimmer A, et al. *Patient Prefer Adherence.* 2017;11:1815–1830^a^	Prospective, observational, single-center cohort study	Basel, Switzerland	Fingolimod (*n* = 98)	Patients with relapsing MS (aged ≥18 years) initiating fingolimod;	Discontinuation rate; % nonadherent (pill count)
				80% female	

Note: ^a^ Study excluded from the meta-analysis

Dosage: DMF 120 mg PO BID for initial 7 days, increase to 240 mg PO BID; fingolimod 0.5 mg PO QD; teriflunomide 7 mg or 14 mg PO QD

**Abbreviations:***BID* twice a day, *CIS* clinically isolated syndrome, *DMD* disease-modifying drug, *DMF* dimethyl fumarate, *MPR* medication possession ratio, *MS* multiple sclerosis, *NS* not specified, *PO* oral administration, *PDC* proportion of days covered, *QD* once a day, *RRMS* relapsing-remitting MS, *tx* therapy

Most studies (*n* = 18; 58.1%) were conducted in the US, one-quarter (25.8%; *n* = 8) were from Europe, two (6.5%) were multinational, two (6.5%) were from Canada, and one (3.2%) was from Kuwait (Table 2). Twelve studies (38.7%) were retrospective analyses of chart/electronic medical records; eight (25.8%) analyzed administrative claims databases; seven (22.6%) were prospective observational cohort studies; three (9.7%) used patient registries; and one (3.2%) was a patient survey (Table 2). The duration of follow-up for the studies ranged from 3 months to 3 years, with 21 studies (67.7%) reporting data at 1-year follow-up. All 31 studies evaluated treatment discontinuation for various follow-up periods. The 1-year treatment discontinuation range was 5.1–42.3% (*n* = 20 studies). For 1-year adherence, 4 studies reported the mean MPR, 6 studies reported MPR ≥80%, 4 studies reported the mean PDC, and 5 studies reported PDC ≥80%.

Quality assessments of the selected studies are presented in Table 1. For the ascertainment of the intervention/validity of study design criteria in the study design and patient selection perspective, approximately half of the studies had a full-quality score and half had a partial-quality score. One study (Lapierre et al. 2016) was assigned a poor-quality score for patient selection because the patient population was neither well-characterized nor representative of patients with RRMS [29]. For the criterion of outcome of interest not present at the start of the study, poor-quality scores were assigned to administrative claims database analyses (Williams et al. 2018; Gerber et al. 2017; Johnson et al. 2017; Burks et al. 2017; Munsell et al. 2016; Bergvall et al. 2014; Agashivala et al. 2013) as they were unable to ascertain patient prescription abandonment [5, 11, 12, 17, 30–32]. For the three criteria in the ‘outcome evaluation’ perspective (appropriateness of the measure of adherence/persistence, adequacy or appropriateness of duration of follow-up, and thoroughness of follow-up for all patients), most studies had full-quality scores. Two studies (Wicks et al. 2016; Ontaneda et al. 2012) were assigned poor-quality scores for the appropriateness of duration of follow-up criterion: Wicks et al. 2016 had a variable follow-up period across patients and Ontaneda et al. 2012 followed patients for only 3 months [33, 34].

### Meta-analysis

A significant Cochran’s Q statistic and an *I*^*2*^ > 50% confirmed the requirement to use random effects models (REMs). A lack of significance on the Egger’s test suggested an absence of publication bias.

**Fig. 2:**
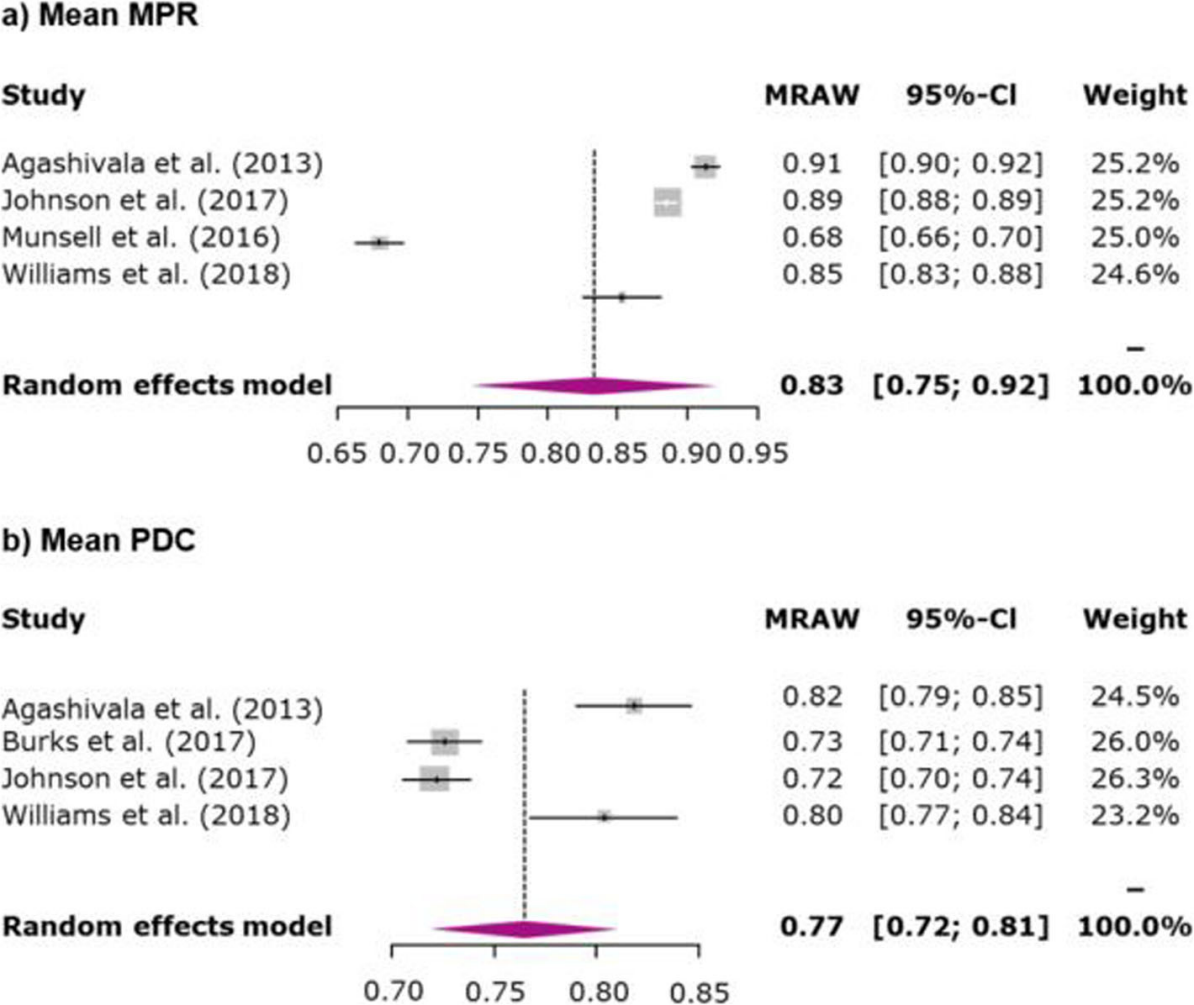
Meta-analysis of mean adherence rate as determined by a) MPR or b) PDC. Note: For studies for which results for treatment-naive and treatment-experienced patients were reported separately (combined data were not available), data were combined; for studies reporting data for more than 1 oral DMD (combined data were not reported), data were combined; for studies reporting data for subgroups (combined data were not reported), data were combined. The area of each grey square is proportional to the study’s weight in the meta-analysis. Weight values are rounded. **Abbreviations:** CI, confidence interval; DMD, disease-modifying drug; MPR, medication possession ratio; MRAW, raw mean; PDC, proportion of days covered

**Fig. 3:**
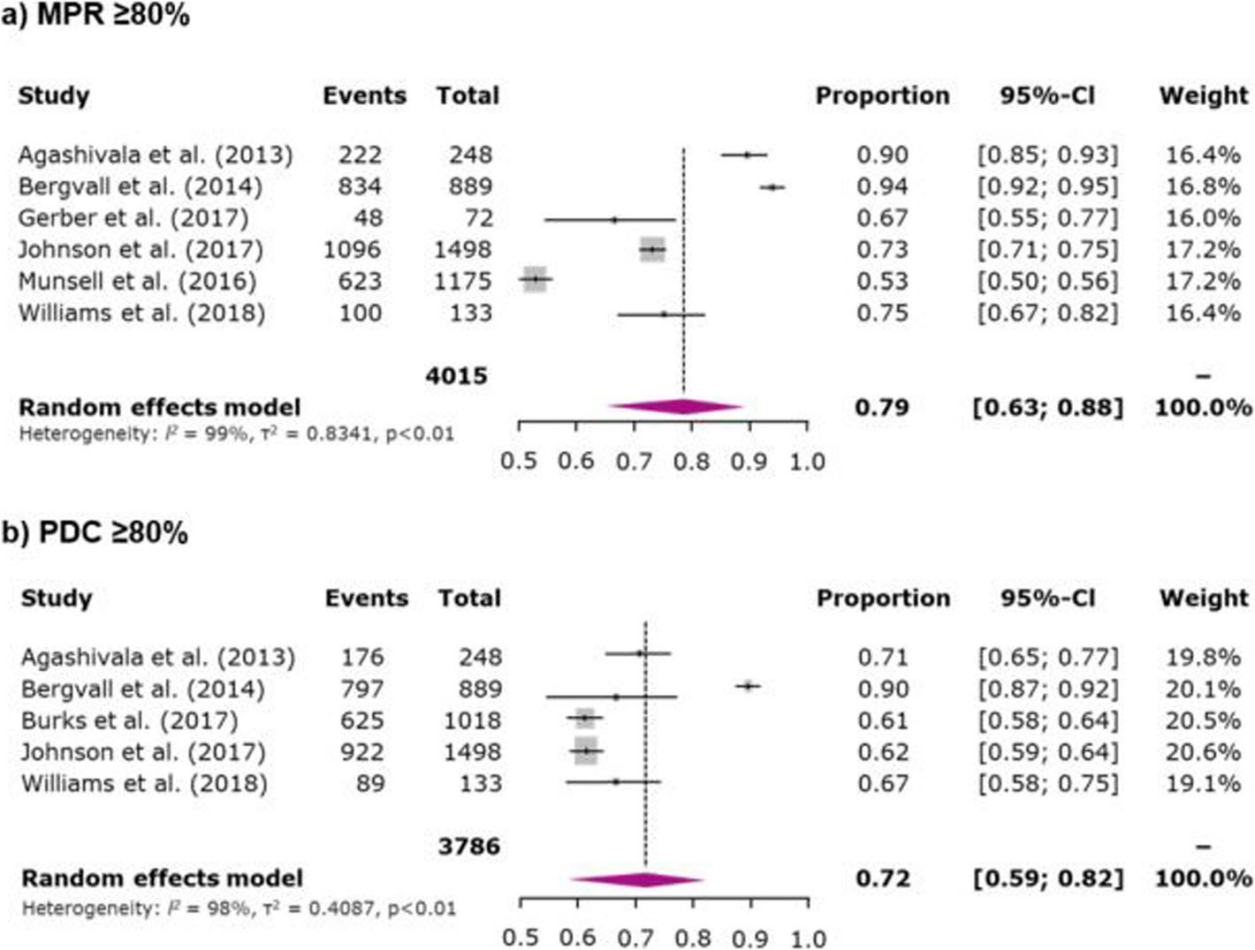
Meta-analysis of proportion of patients adherent to a DMD as determined by MPR or PDC. Note: For studies for which results for treatment-naive and treatment-experienced patients were reported separately (combined data were not available), data were combined; for studies reporting data for more than 1 oral DMD (combined data were not reported), data were combined; for studies reporting data for subgroups (combined data were not reported), data were combined. The area of each grey square is proportional to the study’s weight in the meta-analysis. Weight values are rounded. **Abbreviations:** CI: confidence interval; DMD: disease-modifying drug; MPR: medication possession ratio; PDC: proportion of days covered

**Fig. 4:**
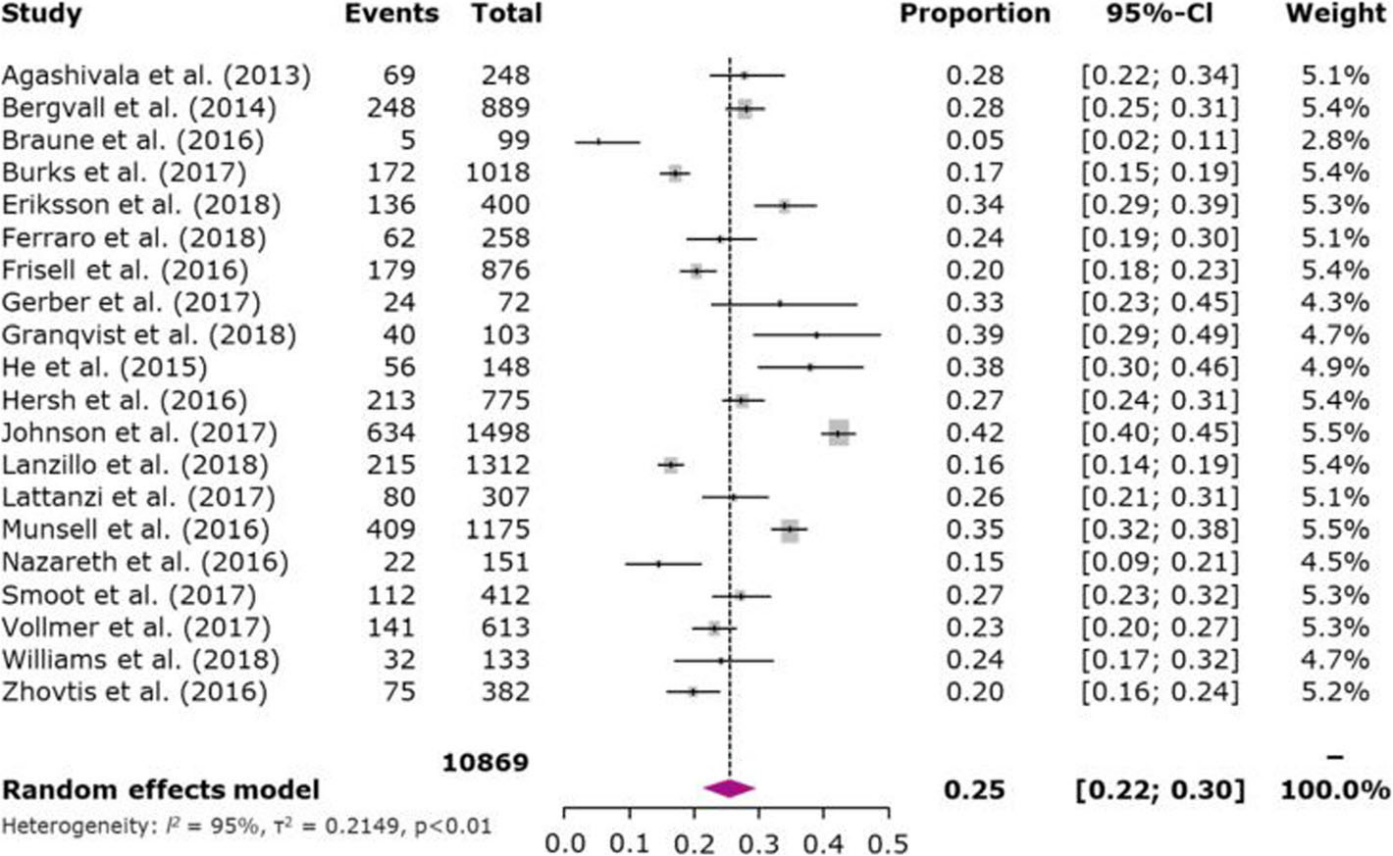
Meta-analysis of proportion of patients discontinuing a DMD. Note: For studies for which results for treatment-naive and treatment-experienced patients were reported separately (combined data were not available), data were combined; for studies reporting data for more than 1 oral DMD (combined data were not reported), data were combined; for studies reporting data for subgroups (combined data were not reported), data were combined; for Lattanzi et al., number of patients discontinuing at 12 months included patients for whom data were not available at 12 months because they stopped taking medications (*n* = 34); 34 + 46 = 80 of 307 or 26.05%; for Vollmer et al. and He et al., for which data were reported in Kaplan–Meier curves only, 1-year persistence rates were extracted from the curves using a digitizer (Guyot P et al. 2012); for Zhovtis et al. 2016, number of patients discontinuing at 12 months was derived from the reported 14-month rate (*n* = 76.4). The area of each grey square is proportional to the study’s weight in the meta-analysis. Weight values are rounded. **Abbreviations:** CI, confidence interval; DMD, disease-modifying drug

The overall mean MPR during the 1-year follow-up period for once- and twice-daily oral maintenance DMDs (4 studies) was 83.3% (95% CI 74.5–92.1%) (Fig. 2a) whereas the overall 1-year mean PDC (4 studies) was 76.5% (95% CI 72.0–81.1%) (Fig. 2b). The pooled MPR ≥80% adherence rate during the 1-year follow-up period across 6 studies was 78.5% (95% CI 63.5–88.5%) (Fig. 3a) and 1-year pooled PDC ≥80% adherence rate (5 studies) was 71.8% (95% CI 59.1–81.9%) (Fig. 3b). All 31 studies evaluated treatment discontinuation using various follow-up periods; the 1-year pooled discontinuation rate across 20 studies for oral maintenance DMDs was 25.4% (95% CI 21.6–29.7%) (Fig. 4).

**Fig. 5:**
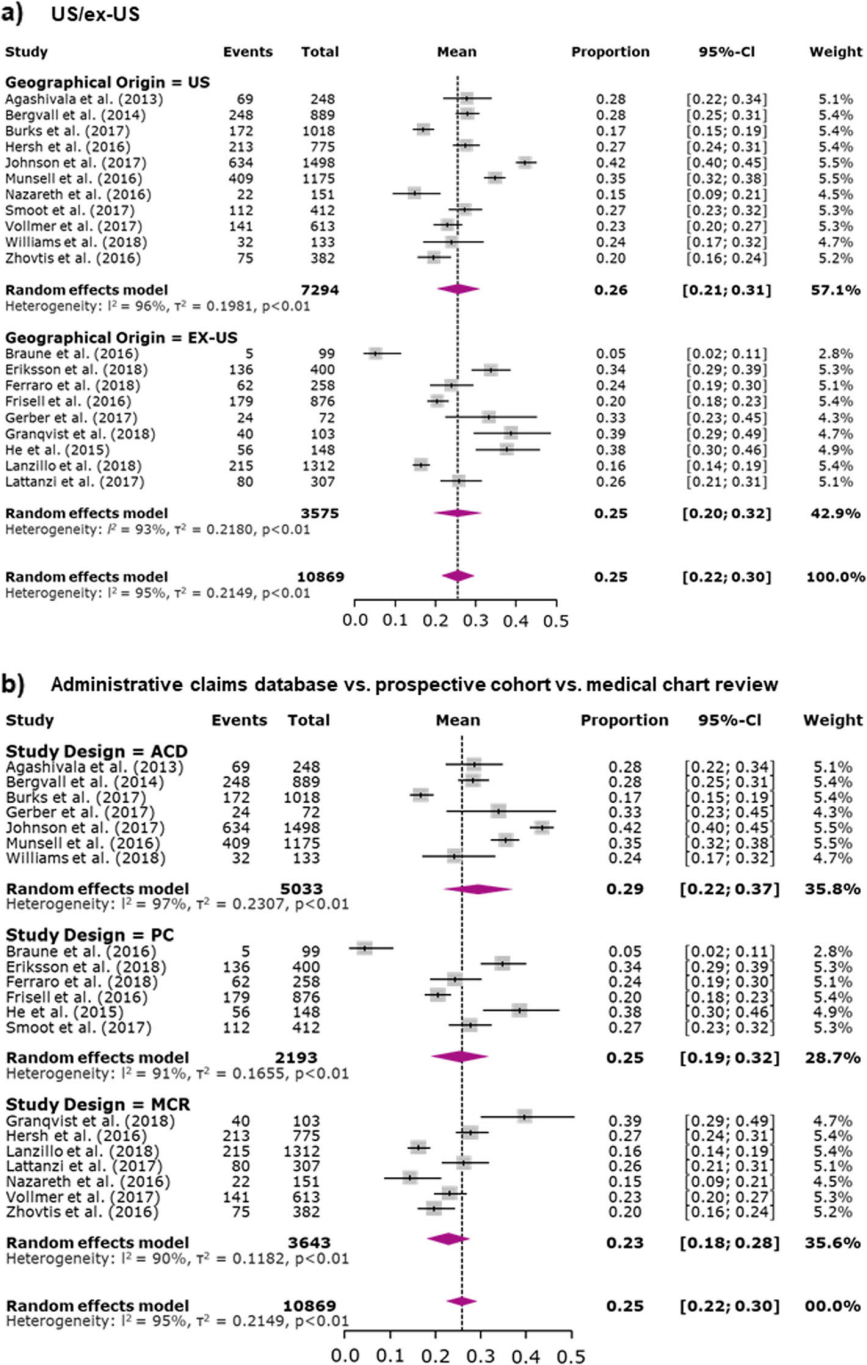
Subgroup meta-analyses of proportion of patients discontinuing a DMD. Note: For studies for which results for treatment naive and treatment-experienced patients were reported separately (combined data were not available), data were combined; for studies reporting data for more than 1 oral DMD (combined data were not reported), data were combined; for studies reporting data for subgroups (combined data were not reported), data were combined. The area of each grey square is proportional to the study’s weight in the meta-analysis. Weight values are rounded. **Abbreviations:** ACD, administrative claims database; CI, confidence interval; MCR, medical chart review; PC, prospective cohort

Subgroup analyses were only conducted for the outcome of discontinuation due to the small number of studies reporting MPR and PDC oral DMD adherence. When studies were analyzed by US and ex-US groupings, similar proportions of patients were found to discontinue DMD therapy (25.6% [95% CI 20.7–31.1%] vs. 25.3% [95% CI 19.6–31.9%], respectively) (Fig. 5a). The proportion of patients discontinuing DMD therapy was greatest for studies evaluating administrative claims databases (29.0, 95% CI 22.0–37.2%), followed by prospective cohort studies (24.7, 95% CI 18.7–31.9%) and medical chart reviews (22.9, 95% CI 18.4–28.1%) (Fig. 5b), though some overlapping of CIs was apparent. The results of the leave-one-out sensitivity analysis confirmed that removal of individual studies did not affect the results (Supplementary Figs. 1, 2a, and 2b).

## Discussion

This is the first meta-analysis to assess real-world adherence to and persistence with multiple oral maintenance DMDs in patients with MS. Results showed that one in five patients do not adhere to once- or twice-daily oral maintenance DMDs, and one in four patients discontinue the initially-prescribed oral DMDs before 1 year. Ninety-five percent CIs for the estimates of adherence and persistence were wide, reflecting the heterogeneity in rates of adherence and discontinuation.

Adherence to DMDs is an important aspect of optimizing patient care in MS as greater adherence has been shown to be associated with improvements in relapse outcomes and quality of life, fewer hospitalizations and emergency room visits, decrease in neuropsychological issues, fewer days of work lost, and lower MS-related medical costs [11–14, 54–57]. Compared with nonadherence, adherence to newly initiated DMDs (oral or injectable) over 1 year among patients in the US was associated with a decrease of 42% in the likelihood of relapse, 38% in emergency visits, and 52% in hospitalizations, as well as an average of 0.7 fewer outpatient visits annually (all *p* < 0.0001) [11]. Based on the differences in predicted US mean costs over the 12-month post-initiation period, adherence was estimated to decrease the total annual medical care costs by $5816 per patient (all costs are reported in 2015 US$), including outpatient visits ($2802), emergency visits ($171), and hospitalizations ($1953) compared with nonadherence [11]. Persistence with DMDs for MS is also important for achieving the best clinical outcomes, as DMD persistence has been shown to be associated with decreased likelihoods of inpatient admission or emergency room visits, [58] decreased relapses and disease progression, [59] and lower costs [60].

An increased understanding of barriers to DMD adherence and persistence and the implementation of efforts to improve adherence and persistence are important. Real-world data on adherence to DMDs in patients with MS can help inform therapeutic decision making [61]. Factors that generally influence adherence and persistence in chronic illness, particularly when cognition is a factor (as in MS), include the frequency and complexity of the dosing regimen, [62, 63] the need for ongoing safety monitoring, office visits, or waiting time associated with dosing, [64] prior treatment experience, [64] and the presence or absence of active symptoms at the time of dosing [64]. Studies in other therapeutic areas have shown that simpler and less frequent dosing produces greater adherence than more frequent administration [62, 63, 65]. A study of MS patient preferences for oral treatments also found that the most important driver of predicted nonadherence was frequency of daily dosing (17.4% out of 100%) [66].

Depression has also been shown to be associated with decreased DMD adherence and persistence in patients with MS. Gerber, et al. and Munsell, et al. both reported a significant relationship between comorbid depression and nonadherence, [12, 17] whereas Lattanzi, et al. reported a significant relationship between depression and persistence [35]. Previous studies have shown that patients with MS and comorbid depression are significantly more likely to be nonadherent to DMDs than patients with MS without comorbid depression [67, 68].

The rates of adherence and discontinuation may differ among oral DMDs due to several potential factors such as the dosing regimen (e.g. once- versus twice-daily), efficacy, tolerability, and adverse events [15, 35, 36]. Assessments of the specific impacts of these factors could not be conducted in the current study due to the small number of studies evaluating the individual oral DMDs’ adherence/persistence over 1 year (i.e., 7 studies evaluated dimethyl fumarate, 10 studies evaluated fingolimod, and only 2 studies evaluated teriflunomide) and due to the challenges in elucidating the specific reasons for any associations that might be observed among studies with significant heterogeneity.

This study was conducted in line with recommendations available in the literature for the use of real-world evidence in meta-analyses [25]. In our findings, 95% CIs showed a wide range of values, particularly for the proportion of patients who were adherent, but this is expected when pooling observational (real-world) data [25]. Heterogeneity is likely to arise because of differences in patient populations, treatments, study design, outcomes, and data quality [69]. This was evident in the results of our assessment of the quality of the studies, which highlighted how the appraisal of the studies needed to be adapted for their individual design (hence our modification of the NewCastle Ottawa Scale [NOS]). Study location, in and of itself, was not a source of heterogeneity in the current meta-analysis.

The *I*^*2*^ statistics obtained in this study ranged from 93.8 to 99.5%. These values are consistent with those noted in other published meta-analyses of medication adherence across indications [19, 70–73].

Subgroup analyses demonstrated that there were essentially no differences between US- and ex-US-based studies and support the need for a better understanding of why patients worldwide discontinue oral DMD treatment. The study design subgroup analyses showed overlapping CIs, indicating a lack of significant differences. However, numerical differences suggested that administrative claims data analyses may more fully capture discontinuation than prospective cohort studies and medical chart reviews. With prospective cohort studies and medical chart reviews, bias may arise from patient or investigator report, whereas administrative claims database analyses provide objective billing information for medication dispensed. Also, cohort studies may also inherently encourage patients to remain in the study and continue treatment. Chart reviews may not capture all discontinuations, depending upon the availability and quality of follow-up data. Further subgroup analyses were limited by the small number of real-world/observational studies, suggesting that more real-world research is needed.

This is the second published meta-analysis of real-world adherence to or persistence with oral DMDs in patients with MS. Kantor et al. 2018 conducted a systematic review and meta-analysis focusing on the real-world persistence with fingolimod in patients with RRMS [19]. In contrast, our meta-analysis evaluated both adherence and persistence in all once- and twice-daily oral maintenance DMDs. Unlike the Kantor et al. 2018 meta-analysis, which included conference posters as well as published studies, the current analysis restricted studies to those published in the peer-reviewed medical literature. Several published studies not captured in the Kantor et al. 2018 evaluation (which captured studies through 2015) were available for inclusion since the literature search was extended through April 2018. The consensus 1-year persistence rate of 82% (95% CI 79–85%) reported by Kantor et al. 2018 corresponds to a mean adherence rate of 77–83% of once- and twice-daily oral maintenance DMDs (depending on the method used to measure adherence) reported in this meta-analysis.

There are limitations to this study. Although nonrandomized cohort studies or observational studies may provide a more ‘real-world’ representation of outcomes, costs, and utilization, differences in baseline characteristics can introduce biases, and the influence of unmeasured factors cannot be ruled out. As described, the substantial proportion of heterogeneity found across the studies is also a limitation. The use of a variable follow-up period for the MPR denominator potentially contributed to an inflated mean MPR [23]. Additionally, MPR may overestimate medication adherence as compared to PDC, as it counts the total number of days of medication supply, which may be inflated by patients who fill their prescriptions early and gain extra supply within a period [74]. No studies evaluating cladribine tablets were identified due to its recent approval, therefore the analyses focused on once- and twice-daily oral maintenance DMDs. Siponimod was not available at the time of the study and is not included in the analyses. Finally, the limited number of studies restricted the ability to analyze MPR and PDC adherence in greater detail and the ability to perform subgroup analysis across adherence and persistence measures.

## Conclusions

Adherence to treatment is an important issue for the management of patients with MS. This meta-analysis of real-world studies showed that approximately one in five patients with MS do not adhere to once- or twice-daily oral maintenance DMD treatment regimens, and one in four patients with MS discontinue once- or twice-daily oral maintenance DMDs before 1 year. Wide 95% CIs for the estimates of adherence and discontinuation reflect the heterogeneity in the rates that was observed. Opportunities to address barriers to DMD adherence in patients with MS remain, such as patient education efforts to manage expectations and to emphasize the importance of adherence, implementation of medication support/reminder techniques, simplification of dosing regimens, and reducing the need for specialized administration or monitoring requirements [61]. Implementation of efforts to improve adherence are essential to improving care of patients with MS.

## Supplementary information

**Additional file 1: Supplementary Methods: Electronic Search Strategy.** Details of electronic literature search strategy, exclusion criteria and full list of search results (including abstract citation, title, text, digital objective identifier [DOI], PubMed ID [PMID], and author names and information).

**Additional file 2: Supplementary Figure 1.** Leave-one-out sensitivity analysis for the proportion of patients discontinuing a DMD. For studies for which results for treatment naive and treatment-experienced patients were reported separately (combined data were not available), data were combined; for studies reporting data for more than 1 oral DMD (combined data were not reported), data were combined; for studies reporting data for subgroups (combined data were not reported), data were combined. **Abbreviations**: DMD, disease-modifying drug.

**Additional file 3: Supplementary Figure 2.** Leave-one-out sensitivity analysis for subgroup analyses for the proportion of patients discontinuing a DMD. For studies for which results for treatment naive and treatment-experienced patients were reported separately (combined data were not available), data were combined; for studies reporting data for more than 1 oral DMD (combined data were not reported), data were combined; for studies reporting data for subgroups (combined data were not reported), data were combined. **Abbreviations**: DMD: disease-modifying drug.

## Data Availability

The datasets used and/or analyzed during the current study are available from the corresponding author on reasonable request.

## Abbreviations

CI: Confidence interval
DMD: Disease-modifying drug
MPR: Medication possession ratio
MS: Multiple sclerosis
NOS: Newcastle-Ottawa Scale
PDC: Proportion of days covered
REM: Random-effects model
RMS: Relapsing MS
RRMS: Relapsing-remitting MS
