# Supplementary material for: Real-world adherence to, and persistence with, once- and twice-daily oral disease-modifying drugs in patients with multiple sclerosis: a systematic review and meta-analysis
Source: BMC Neurol. 2020 Jul 16;20:281. doi: 10.1186/s12883-020-01830-0 (PMC7371467; doi:10.1186/s12883-020-01830-0)
Supplement: Supplementary file 1 — Additional file 1: Supplementary Methods: Electronic Search Strategy. Details of electronic literature search strategy, exclusion criteria and full list of search results (including abstract citation, title, text, digital objective identifier [DOI], PubMed ID [PMID], and author names and information). [file 12883_2020_1830_MOESM1_ESM.docx]

**Additional File 1**

**Supplementary Methods: Electronic Search Strategy**

**Database:** Pubmed

**Date of Search:** April 4, 2018

**Dates of Coverage:** January 1, 2010 to April 4, 2018

**Peer Review:** Two reviewers independently reviewed the search results and reference lists of selected articles to identify additional appropriate studies and carried out data extraction.

**Search Strategy:** (Aubagio[All Fields] OR ("dimethyl fumarate"[MeSH Terms] OR ("cladribine"[MeSH Terms] OR "cladribine"[All Fields]) OR ("dimethyl"[All Fields] AND "fumarate"[All Fields]) OR "dimethyl fumarate"[All Fields]) OR ("fingolimod hydrochloride"[MeSH Terms] OR ("fingolimod"[All Fields] AND "hydrochloride"[All Fields]) OR "fingolimod hydrochloride"[All Fields] OR "fingolimod"[All Fields]) OR ("fingolimod hydrochloride"[MeSH Terms] OR ("fingolimod"[All Fields] AND "hydrochloride"[All Fields]) OR "fingolimod hydrochloride"[All Fields] OR "gilenya"[All Fields]) OR ("dimethyl fumarate"[MeSH Terms] OR ("dimethyl"[All Fields] AND "fumarate"[All Fields]) OR "dimethyl fumarate"[All Fields] OR "tecfidera"[All Fields]) OR ("teriflunomide"[Supplementary Concept] OR "teriflunomide"[All Fields]) OR ("mouth"[MeSH Terms] OR "mouth"[All Fields] OR "oral"[All Fields]) OR (("disease"[MeSH Terms] OR "disease"[All Fields]) AND modifying[All Fields] AND drug[All Fields]) OR DMD[All Fields] OR (("disease"[MeSH Terms] OR "disease"[All Fields]) AND modifying[All Fields] AND ("therapy"[Subheading] OR "therapy"[All Fields] OR "therapeutics"[MeSH Terms] OR "therapeutics"[All Fields])) OR DMT[All Fields]) AND ("multiple sclerosis"[MeSH Terms] OR ("multiple"[All Fields] AND "sclerosis"[All Fields]) OR "multiple sclerosis"[All Fields]) AND (adherence[All Fields] OR ("patient compliance"[MeSH Terms] OR ("patient"[All Fields] AND "compliance"[All Fields]) OR "patient compliance"[All Fields] OR "compliance"[All Fields] OR "compliance"[MeSH Terms]) OR persistence[All Fields] OR discontinuation[All Fields]) AND (("2010/01/01"[PDAT] : "2018/12/31"[PDAT]) AND English[lang])

***priori* exclusion criteria:** lack of primary data; lack of primary real-world DMD adherence/persistence data; lack of oral DMD adherence/persistence data; pediatric studies; non-English studies; and abstract-only available.

**# abstracts obtained:** 415 (Excluded 74 published before 2010, 19 non-English studies, and 2 abstracts from referenced lists [Figure 1: Study selection flowchart]; **Studies highlighted in yellow were included in the meta-analysis)**

1. J Behav Med. 2018 Apr;41(2):253-260. doi: 10.1007/s10865-017-9900-9. Epub 2017

Nov 9.

Baseline predictors of DMT reinitiation among patients with multiple sclerosis

following an MI-CBT intervention.

Excluded b/c no real-world adherence/persistence

Thelen J(1), Bruce A(2)(3), Catley D(3), Lynch S(4), Goggin K(5)(6),

Bradley-Ewing A(5), Glusman M(7), Norouzinia A(7), Strober L(8), Bruce J(7).

Author information:

(1)Department of Psychology, University of Missouri - Kansas City, Kansas City,

MO, USA. jmtyd3@mail.umkc.edu.

(2)Department of Pediatrics, University of Kansas Medical Center, Kansas City,

KS, USA.

(3)Children's Mercy Hospital, Kansas City, MO, USA.

(4)Department of Neurology, University of Kansas Medical Center, Kansas City, KS,

USA.

(5)Health Services and Outcome Research, Children's Mercy Hospitals and Clinics,

Kansas City, MO, USA.

(6)Schools of Medicine and Pharmacy, University of Missouri - Kansas City, Kansas

City, MO, USA.

(7)Department of Psychology, University of Missouri - Kansas City, Kansas City,

MO, USA.

(8)Kessler Foundation, West Orange, NJ, USA.

Patients with multiple sclerosis (MS) are often nonadherent to their disease

modifying therapy (DMT). While recent studies demonstrate enhanced DMT adherence

following intervention grounded in motivational interviewing (MI), little is

known about how to address DMT reinitiation among MS patients who have

prematurely discontinued DMT against medical advice and do not intend to

reinitiate. We examined baseline predictors of DMT reinitiation among patients

with MS who discontinued medications against medical advice following a

telephone-based MI and Cognitive Behavioral Therapy (MI-CBT) intervention.

Following MI-CBT intervention, 66 patients reported whether or not they opted to

reinitiate DMT. Rate of disease progression (β = 0.295) and perceived personal

control (β = - 0.131) emerged as unique significant predictors of DMT

reinitiation following intervention. Clinical characteristics and health-related

beliefs may be used to prospectively identify patients most likely to reinitiate

DMT following MI-CBT intervention, furthering the goal of preserving brain health

and preventing neurologic decline in MS via appropriate DMT utilization. Further

study is warranted to delineate potential mediators and moderators of DMT

reinitiation outcomes.

DOI: 10.1007/s10865-017-9900-9

PMID: 29124557

2. J Neurol. 2018 Mar 16. doi: 10.1007/s00415-018-8831-x. [Epub ahead of print]

Ex-US

A multicentRE observational analysiS of PErsistenCe to Treatment in the new

multiple sclerosis era: the RESPECT study.

Lanzillo R(1), Prosperini L(2)(3), Gasperini C(4), Moccia M(5), Fantozzi R(6),

Tortorella C(4), Nociti V(7), Annovazzi P(8), Cavalla P(9), Radaelli M(10),

Malucchi S(11), Clerici VT(12), Boffa L(13), Buttari F(6)(13), Ragonese P(14),

Maniscalco GT(15), Di Filippo M(16), Buscarinu MC(17), Pinardi F(18), Gallo

A(19), Coghe G(20), Pesci I(21), Laroni A(22), Gajofatto A(23), Calabrese M(23),

Tomassini V(24), Cocco E(20), Solaro C(25); R.I.Re.MS study group.

Author information:

(1)Department of Neurosciences, Reproductive and Odontostomatological Sciences,

Federico II University, Naples, Italy. robertalanzillo@libero.it.

(2)Department of Neurosciences, S. Camillo-Forlanini Hospital, C.ne Gianicolense

87, 00152, Rome, Italy. luca.prosperini@gmail.com.

(3)Department of Neurology and Psychiatry, Sapienza University, Rome, Italy.

luca.prosperini@gmail.com.

(4)Department of Neurosciences, S. Camillo-Forlanini Hospital, C.ne Gianicolense

87, 00152, Rome, Italy.

(5)Department of Neurosciences, Reproductive and Odontostomatological Sciences,

Federico II University, Naples, Italy.

(6)Department of Neurology, IRCCS NEUROMED, Pozzilli, IS, Italy.

(7)Department of Geriatrics, Neurosciences and Orthopedics, Institute of

Neurology, Catholic University, Rome, Italy.

(8)MS Study Center, ASST Valle Olona, Gallarate Hospital, Gallarate, VA, Italy.

(9)MS Center, Neurology 1 Unit, City of Health and Science University Hospital,

Turin, Italy.

(10)Division of Neuroscience, Institute of Experimental Neuroscience (INSpe), S.

Raffaele Scientific Institute, Milan, Italy.

(11)SCDO Neurologia 2-Regional Multiple Sclerosis Center, University Hospital San

Luigi Gonzaga, Orbassano, TO, Italy.

(12)Department of Neuroimmunology and Neuromuscular Diseases, Neurological

Institute C. Besta IRCCS Foundation, Milan, Italy.

(13)MS Clinical and Research Unit, Department of Systems Medicine, Tor Vergata

University, Rome, Italy.

(14)Department of Experimental Biomedicine and Clinical Neurosciences, University

of Palermo, Palermo, Italy.

(15)MS Centre, Neurology Unit, Cardarelli Hospital, Naples, Italy.

(16)Neurology Unit, Medicine Department, University of Perugia, Perugia, Italy.

(17)Department of Neurosciences, Center for Experimental Neurological Therapies,

S. Andrea Hospital, Mental Health and Sensory Organs (NESMOS), Sapienza

University, Rome, Italy.

(18)MS Centre, Bellaria Hospital, UOSI-SM Rehabilitation, Bellaria, BO, Italy.

(19)I Clinic of Neurology, University of Campania, Naples, Italy.

(20)Department of Medical Sciences and Public Health, University of Cagliari,

Cagliari, Italy.

(21)MS Centre, Neurology Unit, S. Secondo Hospital, Fidenza, PA, Italy.

(22)Department of Neurology, Rehabilitation, Ophthalmology, Genetics, Maternal

and Child Health (DINOGMI), University of Genoa, Genoa, Italy.

(23)Department of Neuroscience, Biomedicine and Movement Sciences, University

Hospital of Verona, Verona, Italy.

(24)Division of Psychological Medicine and Clinical Neurosciences, Cardiff

University School of Medicine, University Hospital of Wales, Cardiff, UK.

(25)Neurology Unit, Centro di Recupero e Rieducazione Funzionale, Moncrivello,

VC, Italy.

In this independent, multicenter, retrospective study, we investigated the

short-term persistence to treatment with first-line self-injectable or oral

disease-modifying treatments (DMTs) in patients with relapsing-remitting multiple

sclerosis. Data of patients regularly attending 21 Italian MS Centres who started

a self-injectable or an oral DMT in 2015 were collected to: (1) estimate the

proportion of patients discontinuing the treatment; (3) explore reasons for

discontinuation; (3) identify baseline predictors of treatment discontinuation

over a follow-up period of 12 months. We analyzed data of 1832 consecutive

patients (1289 women, 543 men); 374 (20.4%) of them discontinued the prescribed

DMT after a median time of 6 months (range 3 days to 11.5 months) due to poor

tolerability (n = 163; 43.6%), disease activity (n = 95; 25.4%), adverse events

(n = 64; 17.1%), convenience (i.e. availability of new drug formulations) and

pregnancy planning (n = 21; 1.1%). Although the proportion of discontinuers was

higher with self-injectable (n = 107; 22.9%) than with oral DMT (n = 215; 16.4%),

the Cox regression model revealed no significant between-group difference

(p = 0.12). Female sex [hazard ratio (HR) = 1.39, p = 0.01] and previous exposure

to ≥ 3 DMTs (HR = 1.71, p = 0.009) were two independent risk factors for

treatment discontinuation, regardless of prescribed DMTs. Our study confirms that

persistence to treatment represents a clinical challenge, irrespective of the

route of administration.

DOI: 10.1007/s00415-018-8831-x

PMID: 29549468

3. Curr Med Res Opin. 2018 Mar 10:1-12. doi: 10.1080/03007995.2018.1451311. [Epub

Ex-US

ahead of print]

First-line disease-modifying drugs in relapsing-remitting multiple sclerosis: an

Italian real-life multicenter study on persistence.

Ferraro D(1)(2), Camera V(2), Baldi E(3), Vacchiano V(4), Curti E(5), Guareschi

A(6), Malagù S(7), Montepietra S(8), Strumia S(9), Santangelo M(10), Caniatti

L(3), Foschi M(4), Lugaresi A(4)(11), Granella F(5), Pesci I(6), Motti L(8), Neri

W(9), Immovilli P(12), Montanari E(13), Vitetta F(1), Simone AM(2), Sola P(1).

Author information:

(1)a Department of Neurosciences , Ospedale Civile, Azienda

Ospedaliero-Universitaria , Via Pietro Giardini, 1355 , 41126 Modena , Italy.

(2)b Department of Biomedical, Metabolic and Neurosciences , University of Modena

and Reggio Emilia , Via Giuseppe Campi, 287 , 41125 Modena , Italy.

(3)c Neurology Unit, Department of Neuroscience/Rehabilitation , Azienda

Ospedaliera-Universitaria S. Anna , Via aldo Moro, 8, Cona , 44124 Ferrara ,

Italy.

(4)d Department of Biomedical and Neuromotor Sciences , University of Bologna ,

Via Altura, 3A , 40139 , Bologna , Italy.

(5)e Neurology Unit, Department of Medicine and Surgery , University of Parma ,

Via Gramsci, 14 , 43126 Parma , Italy.

(6)f Neurology Unit, Vaio-Fidenza Hospital , Via Don Enrico Tincati, 5, Fidenza ,

43036 Parma , Italy.

(7)g Neurology Unit, Bufalini Hospital , Via Giovanni Ghirotti, 286 , 47023

Cesena , Italy.

(8)h Neurology Unit, Arcispedale Santa Maria Nuova-IRCCS , Via Amendola, 2 ,

42123 Reggio Emilia , Italy.

(9)i Neurology Unit- Ospedale G.B. Morgagni - L. Pierantoni , Via Carlo Forlanini

, 47100 Forlì , Italy.

(10)j Neurology Unit, Ospedale Ramazzini , Via G. Molinari, 2, 41012 Carpi ,

Modena , Italy.

(11)k IRCCS, Istituto delle Scienze Neurologiche di Bologna , Via Altura, 3A ,

40139 Bologna , Italy.

(12)l Neurology Unit, Department of Specialistic Medicine , G. da Saliceto

Hospital , Via Taverna, 49 , 29121 Piacenza , Italy.

(13)m Polo Neurologico Interaziendale , Via Don Enrico Tincati, 5, 43036 Fidenza

, Parma , Italy.

OBJECTIVE: The introduction of oral disease-modifying drugs (DMDs) in addition to

the available, injectable, ones for Relapsing-Remitting Multiple Sclerosis (RRMS)

could be expected to improve medication persistence due to a greater

acceptability of the route of administration. Aim of the study was to compare the

proportion of patients discontinuing injectable DMDs (interferon beta 1a/1b,

pegylated interferon, glatiramer acetate) with those discontinuing oral DMDs

(dimethylfumarate and teriflunomide) during an observation period of at least 12

months. Secondary aims were to compare the time to discontinuation and the

reasons for discontinuation between the two groups and to explore the demographic

and clinical factors associated with DMD discontinuation.

METHODS: In this prospective, multi-center, real-life observational study,

patients commencing any first-line DMD between January 1st 2015 and July 31st

2016 were enrolled and followed-up for at least twelve months or until the drug

was discontinued.

RESULTS: Of the 520 included patients, 262 (49.6%) started an injectable, and 258

(50.4%) an oral DMD. There was no difference in the proportion of patients on

oral (nr = 62, 24%) or on injectable (nr = 60, 23%) DMDs discontinuing treatment,

the most frequent reason being adverse events/side-effects. Higher baseline EDSS

scores and younger age increased the odds of treatment withdrawal. Time to

treatment discontinuation was not different between the two groups and was not

influenced by the initiated DMD (oral versus injectable), even after adjustment

for baseline differences.

CONCLUSION: The sole route of administration (i.e. oral versus injectable) was

not a significant predictor of persistence with first-line DMDs in RRMS.

DOI: 10.1080/03007995.2018.1451311

PMID: 29526118

Excluded b/c no primary data (narrative review)

4. Cold Spring Harb Perspect Med. 2018 Mar 2. pii: a029066. doi:

10.1101/cshperspect.a029066. [Epub ahead of print]

Natalizumab: Perspectives from the Bench to Bedside.

Shirani A(1), Stüve O(1)(2).

Author information:

(1)Department of Neurology and Neurotherapeutics, University of Texas

Southwestern Medical Center, Dallas, Texas 75390.

(2)Neurology Section, VA North Texas Health Care System, Medical Service Dallas,

VA Medical Center, Dallas, Texas 75216.

Probably no other disease-modifying drug for multiple sclerosis has a more

fascinating story than natalizumab from both the bench to bedside perspective and

the postmarketing experience standpoint. Natalizumab is a monoclonal antibody

that inhibits the trafficking of lymphocytes from the blood into the central

nervous system by blocking the adhesion molecule α4-integrin. Natalizumab was

approved as a disease-modifying drug for relapsing remitting multiple sclerosis

only 12 years after the discovery of its target molecule-a time line that is

rather fast for drug development. However, a few months after its U.S. Food and

Drug Administration approval, natalizumab was withdrawn from the market because

of an unanticipated complication-progressive multifocal leukoencephalopathy. It

was later reinstated with required adherence to a strict monitoring program and

incorporation of mitigation strategies.

Copyright © 2018 Cold Spring Harbor Laboratory Press; all rights reserved.

DOI: 10.1101/cshperspect.a029066

PMID: 29500304

5. Expert Opin Drug Metab Toxicol. 2018 Mar;14(3):341-352. doi:

Excluded b/c no primary data (narrative review)

10.1080/17425255.2018.1432594. Epub 2018 Jan 30.

Pharmacokinetic drug evaluation of daclizumab for the treatment of

relapsing-remitting multiple sclerosis.

Patti F(1), Chisari CG(1), D'Amico E(1), Zappia M(1).

Author information:

(1)a Department "GF Ingrassia", Section of Neurosciences, Multiple Sclerosis

Center , University of Catania , Catania , Italy.

INTRODUCTION: Multiple sclerosis (MS) is a chronic inflammatory demyelinating

disease of the central nervous system. Despite the availability of several

disease-modifying therapies for relapsing MS, there is a need for highly

efficacious targeted therapy with a favorable benefit-risk profile and a high

level of treatment adherence. Daclizumab is a humanized monoclonal antibody

directed against CD25, the α subunit of the high-affinity interleukin 2 (IL-2)

receptor, that reversibly modulates IL-2 signaling. Areas covered: Daclizumab

blocks the activation and expansion of autoreactive T cells that plays a role in

the immune pathogenesis of MS. As its modulatory effects on the immune system,

daclizumab's potential for use in MS was tested extensively showing a high

efficacy in reducing relapse rate, disability progression and the number and

volume of gadolinium-enhancing lesions on brain magnetic resonance imaging.

Moreover, phase II and III trials showed a favorable pharmacokinetic (PK) profile

with slow clearance, linear pharmacokinetics at doses above 100 mg and high

subcutaneous bioavailability, not influenced by age, sex or other clinical

parameters. Expert opinion: Among the new emerging drugs for MS, daclizumab also,

thanks to a favorable PK profile, may represent an interesting and promising

therapeutic option in the wide MS therapies armamentarium.

DOI: 10.1080/17425255.2018.1432594

PMID: 29363337 [Indexed for MEDLINE]

6. JAMA Neurol. 2018 Mar 1;75(3):320-327. doi: 10.1001/jamaneurol.2017.4011.

Comparative Effectiveness of Rituximab and Other Initial Treatment Choices for

Multiple Sclerosis.

Granqvist M(1), Boremalm M(2), Poorghobad A(1), Svenningsson A(3), Salzer J(2),

Frisell T(4), Piehl F(1).

Author information:

(1)Department of Clinical Neuroscience, Karolinska Institutet, Stockholm, Sweden.

(2)Department of Pharmacology and Clinical Neuroscience, Section for Neurology,

Umeå University, Umeå, Sweden.

(3)Department of Clinical Sciences Danderyds Hospital, Karolinska Institutet,

Stockholm, Sweden.

(4)Clinical Epidemiology Unit, Department of Medicine Solna, Karolinska

Institutet, Stockholm, Sweden.

Importance: Comparative real-world effectiveness studies of initial

disease-modifying treatment (DMT) choices for relapsing-remitting multiple

sclerosis (RRMS) that include rituximab are lacking.

Objective: To assess the effectiveness and drug discontinuation rates of

rituximab among patients with newly diagnosed RRMS compared with injectable DMTs,

dimethyl fumarate, fingolimod, or natalizumab.

Design, Setting, and Patients: This retrospective cohort study used prospectively

collected data to examine specialized care of 2 Swedish county-based community

samples of patients with RRMS. Patients with RRMS who received diagnoses from

January 1, 2012, to October 31, 2015, who resided in Stockholm or Västerbotten

Counties were identified from a Swedish multiple sclerosis registry.

Main Outcomes and Measures: All reasons for drug discontinuation of initial

treatment choice (main outcome) and specific reasons for switching (secondary

outcomes) were analyzed with multivariable Cox regression, including propensity

scores.

Results: Among 494 patients (median [interquartile range] age, 34.4 [27.4-43.4]

years; 158 men [32.0%]), 215 received an injectable DMT (43.5%); 86 (17.4%),

dimethyl fumarate; 17 (3.4%), fingolimod; 50 (10.1%), natalizumab; 120 (24.3%),

rituximab; and 6 (1.2%), other DMT. Regional preferences were pronounced, with 42

of 52 (81%) and 78 of 442 (18%) receiving rituximab in Västerbotten and

Stockholm, respectively. The annual discontinuation rate for rituximab,

injectable DMTs, dimethyl fumarate, fingolimod, and natalizumab were 0.03, 0.53,

0.32, 0.38, and 0.29, respectively. Continued disease activity was the main

reason for discontinuation of injectable DMTs, dimethyl fumarate, and fingolimod;

positive John Cunningham virus serology results were the main reason for

discontinuation of natalizumab. Rate of clinical relapses and/or neuroradiologic

disease activity were significantly lower for rituximab compared with injectable

DMTs and dimethyl fumarate, with a tendency for lower relapse rates also compared

with natalizumab and fingolimod. The annual discontinuation rate of initial

treatment choice was significantly lower in Västerbotten compared with Stockholm

(0.09 and 0.37, respectively).

Conclusions and Relevance: Rituximab was superior to all other DMT in terms of

drug discontinuation and displayed better clinical efficacy compared with

injectable DMTs and dimethyl fumarate with borderline significance compared with

natalizumab and fingolimod. The county where rituximab constituted the main

initial treatment choice displayed better outcomes in most measured variables.

Collectively, our findings suggest that rituximab performs better than other

commonly used DMTs in patients with newly diagnosed RRMS.

DOI: 10.1001/jamaneurol.2017.4011

PMID: 29309484

7. Mult Scler. 2018 Mar 1:1352458518765656. doi: 10.1177/1352458518765656. [Epub

ahead of print]

Discontinuation of disease-modifying therapy in patients with multiple sclerosis

over age 60.

Hua LH(1), Fan TH(2), Conway D(3), Thompson N(4), Kinzy TG(4).

Author information:

(1)Lou Ruvo Center for Brain Health, Cleveland Clinic, Las Vegas, NV, USA; Mellen

Center for Multiple Sclerosis Treatment and Research, Cleveland Clinic,

Cleveland, OH, USA.

(2)School of Medicine, Touro University Nevada, Las Vegas, NV, USA.

(3)Mellen Center for Multiple Sclerosis Treatment and Research, Cleveland Clinic,

Cleveland, OH, USA.

(4)Department of Quantitative Health Sciences, Cleveland Clinic, Cleveland, OH,

USA.

BACKGROUND: The risk-benefit ratio of continuing immunomodulating

disease-modifying therapy (DMT) in older multiple sclerosis (MS) patients is

unknown.

OBJECTIVE: To evaluate clinical and patient-reported outcomes after stopping DMT

in older MS patients.

METHODS: Retrospective, observational study identifying patients from our MS

clinics who were aged over 60 and on DMT > 2 years. Cause-specific Cox

proportional hazards regression modeled time to discontinuation and time to

reinitiation of therapy. Pre- and post-discontinuation comparisons of Performance

Scales (PS), Timed 25-Foot Walk, and Patient Health Questionnaire-9 (PHQ9) were

analyzed using linear mixed models.

RESULTS: A total of 600 patients were included, with 178 (29.7%) discontinuing.

Discontinuers were 2.2 years older, had 3.2 years longer disease duration, and

1.6 years lesser treatment exposure. Providers initiated discontinuation more

than patients (68.0%). Only one clinical relapse occurred in discontinuers. A

proportion (10.7%) reinitiated DMT. Provider-initiated discontinuers restarted

less often (hazard ratio (HR): 0.34; 95% confidence interval (CI): 0.12-0.9). In

discontinuers, relapsing-remitting patients had lower PS on average than primary

progressive. Provider-initiated discontinuation was associated with lower PS than

patient- initiated discontinuation. PHQ9 scores appeared higher in those stopping

intravenous (IV) therapies than interferons. Lower PS and PHQ9 indicate better

outcomes.

CONCLUSION: Most patients over age 60, who discontinued DMT, remained off DMT.

This study provides real-world data that may guide clinicians considering

discontinuing DMT.

DOI: 10.1177/1352458518765656

PMID: 29557704

8. Mult Scler Relat Disord. 2018 Feb 26;22:27-34. doi: 10.1016/j.msard.2018.02.028.

Excluded b/c no real-world adherence/persistence

[Epub ahead of print]

Clinical outcomes in patients with relapsing-remitting multiple sclerosis who

switch from natalizumab to delayed-release dimethyl fumarate: A multicenter

retrospective observational study (STRATEGY).

Cohan SL(1), Moses H(2), Calkwood J(3), Tornatore C(4), LaGanke C(5), Smoot

KE(6), Meka V(7), Okwuokenye M(8), Hotermans C(9), Mendoza JP(10), Mann MK(11),

Meltzer LA(12).

Author information:

(1)Providence Multiple Sclerosis Center, Providence Health & Services, 9427 SW

Barnes Road, Portland, OR 97225, USA; Providence Brain and Spine Institute,

Providence Health & Services, 9135 SW Barnes Road, Suite 461, Portland, OR 97225,

USA. Electronic address: stanley.cohan@providence.org.

(2)Vanderbilt-Ingram Cancer Center, 691 Preston Building, Nashville, TN 37232,

USA. Electronic address: harold.moses@Vanderbilt.Edu.

(3)Schapiro Center for Multiple Sclerosis, Minneapolis Clinic of Neurology, 4225

Golden Valley Road, Golden Valley, MN 55422, USA. Electronic address:

Jcalkwood@gmail.com.

(4)Department of Neurology, Medstar Georgetown University Hospital, Pasquerilla

Healthcare Center (PHC), 7th Floor, 3800 Reservoir Road, N.W., Washington, D.C.

20007, USA. Electronic address: Tornatoc@gunet.georgetown.edu.

(5)North Central Neurology Associates, 1809 Kress St., Cullman, AL 35058, USA.

Electronic address: claganke@prn-inc.net.

(6)Providence Multiple Sclerosis Center, Providence Health & Services, 9427 SW

Barnes Road, Portland, OR 97225, USA; Providence Brain and Spine Institute,

Providence Health & Services, 9135 SW Barnes Road, Suite 461, Portland, OR 97225,

USA. Electronic address: kyle.smoot@providence.org.

(7)Biogen, 225 Binney St., Cambridge, MA 02142, USA. Electronic address:

vennmeka@yahoo.com.

(8)Biogen, 225 Binney St., Cambridge, MA 02142, USA. Electronic address:

chiefendorce@hotmail.com.

(9)Biogen, 225 Binney St., Cambridge, MA 02142, USA. Electronic address:

Christophe.Hotermans@Biogen.com.

(10)Biogen, 225 Binney St., Cambridge, MA 02142, USA. Electronic address:

Jason.Mendoza@biogen.com.

(11)Biogen, 225 Binney St., Cambridge, MA 02142, USA. Electronic address:

monica.mann@Biogen.com.

(12)Biogen, 225 Binney St., Cambridge, MA 02142, USA. Electronic address:

lmeltzer@gmail.com.

BACKGROUND: Delayed-release dimethyl fumarate (DMF) may be a therapeutic option

for patients with relapsing-remitting multiple sclerosis (RRMS) who are treated

with natalizumab and require a change in therapy. However, there is limited

information regarding predictors of favorable treatment outcomes in patients

switching from natalizumab to DMF. Clinical practices and sequencing protocols

vary. Herein, we present the clinical results, including annualized relapse rate

(ARR) and risk of relapse, of a phase 4 retrospective observational study of

patients with RRMS who switched from natalizumab to DMF in a community practice

setting (STRATEGY).

METHODS: STRATEGY was performed through a single time point medical record

abstraction; no study visits or procedures were required. Key inclusion criteria

included age ≥ 18 years, RRMS diagnosis (McDonald criteria, 2010 revised), ≥ 12

months of continuous treatment with natalizumab monotherapy before DMF

initiation, and initiation of DMF ≥ 12 months before enrollment. Patients were

eligible to enroll regardless of current DMF use.

RESULTS: A total of 530 patients at 45 US sites enrolled, and 506 met the

inclusion criteria and were included in the modified evaluable population for

analysis. Mean (SD) age at DMF initiation was 47.0 (10.9) years, with a mean (SD)

of 12.7 (7.2) years since MS diagnosis. The mean (SD) duration of natalizumab

treatment was 3.4 (1.9) years, and the mean (SD) washout from natalizumab

discontinuation to DMF initiation (n = 502) was 101.6 (164.0) days. Overall risk

of relapse 12 months after DMF initiation was 19.6%. Overall unadjusted ARR was

higher during the 12 months following initiation of DMF treatment compared with

the 12 months following initiation of natalizumab treatment (rate ratio, 2.32

[95% CI, 1.69-3.18]; p < 0.0001), but was lower compared with that observed in

the year before initiation of natalizumab (rate ratio, 0.51 [95% CI, 0.40-0.64];

p < 0.0001). At 1 year following initiation of DMF treatment, the relapse rate

was lower for patients who did not experience a relapse during 1 year following

initiation of natalizumab treatment than for those who did (rate ratio for

relapse rate, 0.47 [95% CI, 0.16-1.38]; p = 0.1664). The relapse rate for

patients who did not relapse during natalizumab treatment was significantly lower

with a washout period of ≤ 90 days as compared with a washout period of > 90 days

(rate ratio for relapse rate, 0.49 [95% CI, 0.26-0.90]; p = 0.0216). A total of

42 (8%) patients reported ≥ 1 adverse event leading to DMF discontinuation during

the study; the most commonly reported events were gastrointestinal disorders

(n = 21; 4%).

CONCLUSIONS: Results from this multicenter retrospective observational study

suggest that DMF may be an effective treatment option for patients who

discontinue natalizumab in routine clinical practice. ARR was lower in patients

who initiated DMF within 90 days of natalizumab discontinuation compared with

patients who initiated DMF after 90 days of natalizumab discontinuation.

TRIAL REGISTRATION NUMBER: ClinicalTrials.gov identifier NCT02159573.

Copyright © 2018 Biogen. Published by Elsevier B.V. All rights reserved.

DOI: 10.1016/j.msard.2018.02.028

PMID: 29524759

9. Curr Med Chem. 2018 Feb 25. doi: 10.2174/0929867325666180226105612. [Epub ahead

Excluded b/c no primary data (narrative review)

of print]

New life to an old treatment: pegylated Interferon beta 1a in the management of

multiple sclerosis.

Ortiz MA(1), Espino-Paisan L(1), Nunez C(2), Alvarez-Lafuente R(3), Urcelay E(1).

Author information:

(1)Servicio de Inmunologia Clinica, Hospital Clinico San Carlos, Instituto de

Investigacion Sanitaria del Hospital Clinico San Carlos (IdISSC), 28040 Madrid.

Spain.

(2)Laboratorio de Investigacion en Genetica de Enfermedades Complejas, Instituto

de Investigacion Sanitaria del Hospital Clinico San Carlos (IdISSC), 28040

Madrid. Spain.

(3)Servicio de Neurologia, Hospital Clinico San Carlos, Instituto de

Investigacion Sanitaria del Hospital Clinico San Carlos (IdISSC), 28040 Madrid.

Spain.

In the 1990s, the betainterferons and glatiramer acetate were introduced for

treating relapsing-remitting multiple sclerosis. These medications have a

demonstrated record of efficacy and safety, although they require frequent

administration via injection and are only partially effective. The optimization

of treatment in patients who do not respond adequately to this first-line therapy

is essential for attaining the best long-term outcomes. Switching to the recently

approved emergent therapies is a strategy to consider for treatment of patients

with a suboptimal response. This review summarizes the mechanisms of action,

clinical benefits, and safety profiles of current multiple sclerosis

disease-modifying therapies, including highly efficacious monoclonal antibodies

or convenient oral therapies. Although the first-line interferon beta exhibits a

favorable benefit-to-risk profile, treatment compliance is compromised

potentially due to its known adverse events and frequent injectable

administration. Less frequent dosing and improved pharmacological properties have

been achieved by reaction of interferon beta with chemically activated

polyethylene glycol. Provided that none of the available therapies shows better

effectiveness for all outcomes and their safety in clinical practice is a

fundamental concern, the pegylated form of interferon beta seems to keep its

place as a competitive therapeutic option.

Copyright© Bentham Science Publishers; For any queries, please email at

epub@benthamscience.org.

DOI: 10.2174/0929867325666180226105612

PMID: 29484976

10. ACS Chem Neurosci. 2018 Feb 1. doi: 10.1021/acschemneuro.7b00519. [Epub ahead of

print]

Excluded b/c no real-world adherence/persistence

Preclinical Explorative Assessment of Dimethyl Fumarate-Based Biocompatible

Nanolipoidal Carriers for the Management of Multiple Sclerosis.

Kumar P(1), Sharma G(2), Gupta V(3), Kaur R(4), Thakur K(2), Malik R(1), Kumar

A(3), Kaushal N(4), Raza K(1).

Author information:

(1)Department of Pharmacy, School of Chemical Sciences and Pharmacy, Central

University of Rajasthan , Bandar Sindri, Distt. Ajmer, Rajasthan, India 305817.

(2)Division of Pharmaceutics, University Institute of Pharmaceutical Sciences,

Panjab University , Chandigarh, India 160014.

(3)Pharmacology Division, University Institute of Pharmaceutical Sciences, UGC

Centre of Advanced Studies (UGC-CAS), Panjab University , Chandigarh, India

160014.

(4)Department of Biophysics, Panjab University , Chandigarh, India 160014.

Multiple sclerosis (MS) is a neurodegenerative disease in which myelin sheath

damage occurs due to internal and external factors. MS especially affects the

young population. Dimethyl fumarate (DMF) is a promising agent for MS treatment,

although it is associated with concerns such as poor brain permeation, multiple

dosing, and gastrointestinal flushing. The present study attempts to evaluate the

preclinical performance of specially designed DMF-based lipoidal nanoparticles in

a cuprizone-induced demyelination model in rodents. The studies proved the

efficacy of lipid-based nanoparticles containing DMF in a once-a-day dosage

regimen over that of thrice-a-day plain DMF administration on crucial parameters

like motor coordination, grip strength, mortality, body weight, and locomotor

activity. However, neither blank lipid nor blank neuroprotective (vitamins A, D,

and E) loaded nanoparticles were able to elicit any desirable behavioral

response. Histopathological studies showed that the designed once-a-day DMF

nanomedicines were well tolerated and rejuvenated the myelin sheath vis-à-vis the

plain DMF thrice-a-day regimen. These findings provide proof of concept for a

biocompatible nanomedicine for MS with tremendous promise for effective brain

delivery and patient compliance on the grounds of a reduction in the dosage

frequency.

DOI: 10.1021/acschemneuro.7b00519

PMID: 29357233

11. Eur J Clin Pharmacol. 2018 Feb;74(2):219-226. doi: 10.1007/s00228-017-2366-4.

Ex-US

Epub 2017 Nov 11.

Persistence with dimethyl fumarate in relapsing-remitting multiple sclerosis: a

population-based cohort study.

Eriksson I(1)(2), Cars T(3), Piehl F(4), Malmström RE(5)(6), Wettermark B(5)(7),

von Euler M(5)(6)(8).

Author information:

(1)Department of Medicine Solna, Karolinska Institutet, Stockholm, Sweden.

irene.eriksson@ki.se.

(2)Department of Healthcare Development, Stockholm County Council, Stockholm,

Sweden. irene.eriksson@ki.se.

(3)Department of Medical Sciences, Uppsala University, Uppsala, Sweden.

(4)Department of Clinical Neuroscience, Karolinska Institutet, Stockholm, Sweden.

(5)Department of Medicine Solna, Karolinska Institutet, Stockholm, Sweden.

(6)Clinical Pharmacology, Karolinska University Hospital, Stockholm, Sweden.

(7)Department of Healthcare Development, Stockholm County Council, Stockholm,

Sweden.

(8)Department of Clinical Science and Education, Södersjukhuset, Karolinska

Institutet, Stockholm, Sweden.

PURPOSE: To describe patients initiating dimethyl fumarate (DMF) and measure

persistence with DMF, discontinuation, and switching in treatment-naïve DMF

patients and patients switching to DMF from other multiple sclerosis

disease-modifying treatments (DMTs).

METHODS: A population-based cohort study of all Stockholm County residents

initiating DMF from 9 May 2014 until 31 May 2017. All data were derived from a

regional database that collects individual-level data on healthcare and drug

utilization of all residents. The study outcomes were persistence with DMF and

DMF discontinuation and switching to other DMTs. Persistence was measured as the

number of days until either DMF discontinuation (treatment gap ≥ 60 days) or

switching to another DMT.

RESULTS: The study included 400 patients (median follow-up = 2.5 years). The

majority had previously been treated with other DMTs (61%). Throughout the

follow-up period, 124 patients (31%) discontinued DMF and 114 patients (29%)

switched treatment. Overall, 34% of patients initiating DMF stopped treatment

within 1 year and only 43% of patients remained on DMF at 2 years from treatment

initiation.

CONCLUSIONS: DMF had a rapid market uptake likely due to high expectations held

by both patients and clinicians. However, persistence with DMF in routine

clinical practice was found to be low.

DOI: 10.1007/s00228-017-2366-4

PMCID: PMC5765201

PMID: 29128972

12. Mult Scler. 2018 Feb;24(2):175-185. doi: 10.1177/1352458517695469. Epub 2017 Feb

Excluded b/c pediatric patients

1.

Risk factors for non-adherence to disease-modifying therapy in pediatric multiple

sclerosis.

Schwartz CE(1), Grover SA(2), Powell VE(3), Noguera A(4), Mah JK(5), Mar S(6),

Mednick L(7), Banwell BL(8), Alper G(9), Rensel M(10), Gorman M(7), Waldman A(8),

Schreiner T(11), Waubant E(12), Yeh EA(13).

Author information:

(1)DeltaQuest Foundation, Inc., Concord, MA, USA/Departments of Medicine and

Orthopaedic Surgery, School of Medicine, Tufts University, Boston, MA, USA.

(2)Department of Neuroscience and Mental Health, Research Institute, The Hospital

for Sick Children, Toronto, ON, Canada.

(3)DeltaQuest Foundation, Inc., Concord, MA, USA.

(4)Hospital for Sick Children, Toronto, ON, Canada/Division of Neurology and

Division of Neuroscience and Mental Health, Department of Pediatrics, Research

Institute, Hospital for Sick Children, Toronto, ON, Canada.

(5)Division of Neurology, Department of Pediatrics, Cumming School of Medicine,

Alberta Children's Hospital, University of Calgary, Calgary, AB, Canada.

(6)Departments of Neurology and Pediatrics, St. Louis Children's Hospital,

Washington University School of Medicine in St. Louis, St. Louis, MO, USA.

(7)Boston Children's Hospital, Harvard Medical School, Boston, MA, USA.

(8)Division of Neurology, Children's Hospital of Philadelphia, Perelman School of

Medicine, University of Pennsylvania, Philadelphia, PA, USA.

(9)Division of Child Neurology, Department of Pediatrics, School of Medicine,

University of Pittsburgh, Pittsburgh, PA, USA.

(10)Department of Neurology, The Mellen Center, Cleveland Clinic, Cleveland, OH,

USA.

(11)Departments of Neurology and Pediatrics, University of Colorado Denver,

Denver, CO, USA.

(12)Department of Neurology, University of San Francisco, San Francisco, CA, USA.

(13)Division of Neurology and Department of Neuroscience and Mental Health,

Department of Pediatrics, Research Institute, Hospital for Sick Children,

Toronto, ON, Canada/Faculty of Medicine, The University of Toronto, Toronto, ON,

Canada.

BACKGROUND: Adherence to disease-modifying therapies (DMTs) in pediatric multiple

sclerosis (MS) is not well understood. We examined the prevalence and risk

factors for poor adherence in pediatric MS.

METHODS: This cross-sectional study recruited youth with MS from 12 North

American pediatric MS clinics. In addition to pharmacy-refill data, patients and

parents completed self-report measures of adherence and quality of life.

Additionally, patients completed measures of self-efficacy and well-being. Factor

analysis and linear regression methods were used.

RESULTS: A total of 66 youth (mean age, 15.7 years) received MS DMTs (33% oral,

66% injectable). Estimates of poor adherence (i.e. missing >20% of doses) varied

by source: pharmacy 7%, parent 14%, and patient 41%. Factor analysis yielded two

composites: adherence summary and parental involvement in adherence. Regressions

revealed that patients with better self-reported physical functioning were more

adherent. Parents were more likely to be involved in adherence when their child

had worse parent-reported PedsQL School Functioning and lower MS Self-Efficacy

Control. Oral DMTs were associated with lesser parental involvement in adherence.

CONCLUSION: Rates of non-adherence varied by information source. Better

self-reported physical functioning was the strongest predictor of adherence.

Parental involvement in adherence was associated with worse PedsQL School

Functioning and lower MS Self-Efficacy-measured confidence in controlling MS.

DOI: 10.1177/1352458517695469

PMID: 28273780

13. Ther Adv Neurol Disord. 2018 Jan 23;11:1756285617748845. doi:

Excluded b/c no real-world adherence/persistence

10.1177/1756285617748845. eCollection 2018.

Patient satisfaction and healthcare services in specialized multiple sclerosis

centres in Germany.

Becker V(1), Heeschen V(2), Schuh K(2), Schieb H(2), Ziemssen T(3).

Author information:

(1)Neurologische Praxis Eppendorf, Hamburg, Germany.

(2)Novartis Pharma GmbH, Nuremberg, Germany.

(3)Center of Clinical Neuroscience, University Clinic Carl Gustav Carus Dresden,

Fetscherstraße 74, 01307 Dresden, Germany.

Background: As patients with multiple sclerosis (MS) require lifelong treatment,

optimization of therapy with respect to efficacy and safety is needed to limit

long-term disease progression. Patients with MS also need a range of

health-related services. Satisfaction with these as well as treatment is

clinically relevant because satisfied patients are more likely to adhere to

therapy. The aim of this study was to determine the status of patient

satisfaction and of healthcare services in 70 specialized MS centres in Germany.

Methods: In 2011, patients with MS responded to a questionnaire, which solicited

clinical and demographic information, as well as patients' perceptions of their

overall situation and their satisfaction with treatment.

Results: Of 2791 patients surveyed, 81.9% had relapsing-remitting MS with mild

disability [mean (standard deviation) Expanded Disability Status Scale score: 2.6

(1.8)]. Disease activity data were collected from 2205 patients, of whom 57.6%

had remained relapse-free during the preceding 12 months. However, 38.9% had

experienced one or more relapses, most of whom (67.3%) while receiving

immunomodulatory treatment. About one-third of the patients indicated that they

were more dissatisfied with their overall situation compared with the time before

diagnosis. However, many patients (58.3%) were satisfied with their existing

medication. Overall, 72.8% of patients would prefer oral to injectable

treatments, assuming there was no difference in their efficacy.

Conclusions: A substantial proportion of patients experienced breakthrough

disease on treatment and may potentially benefit from a change of therapy.

Although largely satisfied with treatment, most patients with MS would choose

oral over injectable treatments.

DOI: 10.1177/1756285617748845

PMCID: PMC5788086

PMID: 29399052

Conflict of interest statement: Conflict of interest statement: Veit Becker has

received reimbursements for participation in scientific advisory boards and

speaker honoraria from Merck, Biogen, Novartis Pharma AG, Genzyme, Sanofi. He

received research support from Novartis Pharma AG, Merck and Teva. Volker

Heeschen, Katrin Schuh and Heinke Schieb are employees of Novartis Pharma GmbH.

Tjalf Ziemssen has received reimbursements for participation in scientific

advisory boards from Bayer Healthcare, Biogen Idec, Novartis Pharma AG, Merck

Serono, Teva, Genzyme, and Synthon. He has also received speaker honorarium from

Bayer Healthcare, Biogen Idec, Genzyme, Merck Sharp & Dohme, GlaxoSmithKline,

Novartis Pharma AG, Teva, Sanofi Aventis, and Almirall. He has also received

research support from Bayer Healthcare, Biogen Idec, Genzyme, Novartis Pharma AG,

Teva, and Sanofi Aventis.

14. J Neurol. 2018 Jan 22. doi: 10.1007/s00415-018-8752-8. [Epub ahead of print]

Excluded b/c no primary data (NMA)

Short- and long-term clinical outcomes of use of beta-interferon or glatiramer

acetate for people with clinically isolated syndrome: a systematic review of

randomised controlled trials and network meta-analysis.

Armoiry X(1), Kan A(2), Melendez-Torres GJ(2), Court R(2), Sutcliffe P(2),

Auguste P(2), Madan J(2), Counsell C(3), Clarke A(2).

Author information:

(1)Warwick Medical School, Division of Health Sciences, University of Warwick,

Gibbet Hill Road, CV4 7AL, Coventry, England, UK. armoiryxa@gmail.com.

(2)Warwick Medical School, Division of Health Sciences, University of Warwick,

Gibbet Hill Road, CV4 7AL, Coventry, England, UK.

(3)Division of Applied Health Sciences, University of Aberdeen, Aberdeen,

Scotland, UK.

BACKGROUND: Beta-interferon (IFN-β) and glatiramer acetate (GA) have been

evaluated in people with clinically isolated syndrome (CIS) with the aim to delay

a second clinical attack and a diagnosis of clinically definite multiple

sclerosis (CDMS). We systematically reviewed trials evaluating the short- and

long-term clinical effectiveness of these drugs in CIS.

METHODS: We searched multiple electronic databases. We selected randomised

controlled studies (RCTs) conducted in CIS patients and where the interventions

were IFN-β and GA. Main outcomes were time to CDMS, and discontinuation due to

adverse events (AE). We compared interventions using random-effect network

meta-analyses (NMA). We also reported outcomes from long-term open-label

extension (OLE) studies.

RESULTS: We identified five primary studies. Four had open-label extensions

following double-blind periods comparing outcomes between early vs delayed DMT.

Short-term clinical results (double-blind period) showed that all drugs delayed

CDMS compared to placebo. Indirect comparisons did not suggest superiority of any

one active drug over another. We could not undertake a NMA for discontinuation

due to AE. Long-term clinical results (OLE studies) showed that the risk of

developing CDMS was consistently reduced across studies after early DMT treatment

compared to delayed DMT (HR = 0.64, 95% CI 0.55, 0.74). No data supported the

benefit of DMTs in reducing the time to, and magnitude of, disability

progression.

CONCLUSIONS: Meta-analyses confirmed that IFN-β and GA delay time to CDMS

compared to placebo. In the absence of evidence that early DMTs can reduce

disability progression, future research is needed to better identify patients

most likely to benefit from long-term DMTs.

DOI: 10.1007/s00415-018-8752-8

PMID: 29356977

15. Curr Med Res Opin. 2018 Jan;34(1):107-115. doi: 10.1080/03007995.2017.1374937.

Epub 2017 Oct 3.

Adherence, persistence, and discontinuation among Hispanic and African American

patients with multiple sclerosis treated with fingolimod or glatiramer acetate.

Williams MJ(1), Johnson K(2), Trenz HM(3), Korrer S(3), Halpern R(3), Park Y(4),

Herrera V(2).

Author information:

(1)a Multiple Sclerosis Center of Atlanta , Atlanta , GA , USA.

(2)b Novartis Pharmaceuticals Corporation , East Hanover , NJ , USA.

(3)c Optum , Eden Prairie , MN , USA.

(4)d University of Maryland School of Pharmacy , Baltimore , MD , USA.

OBJECTIVE: Few studies have examined compliance to disease-modifying therapies

(DMTs) for multiple sclerosis (MS) in minority populations. This study compared

adherence, discontinuation, and persistence for fingolimod (FTY) and glatiramer

acetate (GA) initiators among Hispanic and African American patients with MS.

METHODS: This retrospective claims data study examined Hispanic and African

American adults with MS who initiated FTY or GA between September 1, 2010 and

June 30, 2014. Outcomes (adherence, discontinuation, and persistence) were

analyzed descriptively and with multivariable models, comparing FTY and GA

cohorts within racial/ethnic groups. Adherence was assessed using medication

possession ratio (MPR) and proportion of days covered (PDC).

RESULTS: There were 171 patients in the Hispanic group (62 FTY, 109 GA) and 210

in the African American group (71 FTY, 139 GA). A larger proportion of GA

initiators than FTY initiators were treatment-naïve; other baseline

characteristics were similar between cohorts. Hispanic FTY initiators had greater

mean MPR, PDC, and persistence and less discontinuation than GA initiators.

African American FTY initiators had greater mean PDC than GA initiators; other

outcomes favored FTY but were not statistically significant. Multivariable

analysis results were consistent with the unadjusted results, but differences

between treatment cohorts were not statistically significant.

CONCLUSIONS: Hispanic and African American patients with MS who initiated FTY had

higher adherence than those who initiated GA, similar to the general MS

population. These findings suggest that adherence should be considered in DMT

selection, and racial/ethnic variations in MS disease course may not be primarily

attributable to differences in DMT compliance.

DOI: 10.1080/03007995.2017.1374937

PMID: 28857632

Excluded b/c no real-world adherence/persistence

16. Acta Neurol Scand. 2017 Dec 3. doi: 10.1111/ane.12882. [Epub ahead of print]

Severe multiple sclerosis reactivation during prolonged lymphopenia after

dimethyl fumarate discontinuation.

Zecca C(1)(2), Antozzi CG(2), Torri Clerici V(2), Ferrazzini M(3), Mantegazza

RE(2), Rossi S(2), Gobbi C(1).

Author information:

(1)Neurocenter of Southern Switzerland, Ospedale Regionale di Lugano, Lugano,

Switzerland.

(2)Neuroimmunology and Neuromuscular Diseases Unit, IRCCS Fondazione Istituto

Neurologico Carlo Besta, Milan, Italy.

(3)Studio medico neurologico, Locarno, Switzerland.

BACKGROUND: Delayed-release dimethyl fumarate (DMF) treatment can be associated

with reduced lymphocyte and leucocyte counts, which might persist after DMF

discontinuation.

CASE PRESENTATION: We report the case of a patient with severe disease

reactivation despite prolonged lymphopenia after DMF discontinuation. We describe

the frequency and impact of prolonged lymphopenia after DMF discontinuation at

two tertiary MS centres. A 36-year-old female patient with multiple sclerosis was

switched to DMF after 14 years of treatment with interferon beta-1a. DMF was

suspended after 4 months because of persistent lymphopenia for 3 months. Six

months later, the patient had a severe relapse with multiple enhancing brain

lesions at MRI although lymphopenia was still persistent. Haematological

assessment excluded other causes of lymphopenia, which was evaluated as a

probable iatrogenic complication of DMF. The patient was treated with i.v.

methylprednisolone 1 gr daily for 3 days with clinical recovery.

CONCLUSIONS: Prolonged lymphopenia after DMT discontinuation does not protect

against disease reactivation. Starting a new immune therapy should be balanced

against the option of a "wait and see." A different immunotherapeutic strategy

such as an anti-B therapeutic approach could be considered.

© 2017 John Wiley & Sons A/S. Published by John Wiley & Sons Ltd.

DOI: 10.1111/ane.12882

PMID: 29205270

17. Curr Med Res Opin. 2017 Dec;33(12):2099-2106. doi: 10.1080/03007995.2017.1380616.

Epub 2017 Sep 28.

Relapse outcomes, safety, and treatment patterns in patients diagnosed with

relapsing-remitting multiple sclerosis and initiated on subcutaneous interferon

β-1a or dimethyl fumarate: a real-world study.

Ernst FR(1), Barr P(1), Elmor R(1), Wong SL(2).

Author information:

(1)a Health Economics and Outcomes Research , Indegene, Inc. , Kennesaw , GA ,

USA.

(2)b Global Evidence & Value Development, Global Research & Development , EMD

Serono, Inc. , Billerica , MA , USA.

OBJECTIVE: To estimate real-world treatment patterns, safety, and relapse

outcomes of subcutaneous (sc) interferon (IFN) β-1a (Rebif) vs dimethyl fumarate

(DMF; Tecfidera), to treat relapsing-remitting multiple sclerosis (RRMS).

METHODS: A US retrospective chart review of 450 randomly selected adults newly

diagnosed with RRMS who received sc IFN β-1a (n = 143) or DMF (n = 307) was

conducted. Patients were either (a) treatment-naïve, initiating first-line

treatment with sc IFN β-1a or DMF, or (b) previously treated, switching to sc IFN

β-1a or DMF. Two years' follow-up data were captured. Patient characteristics,

persistence, and adverse events between treatment groups were compared using

t-tests or Chi-square tests. Kaplan-Meier curves with log-rank tests and Cox

proportional hazards models were used to compare time to, and risk of

non-persistence. Annualized Relapse Rates (ARR) were calculated using a robust

variance Poisson model adjusting for covariates. Propensity scores were used to

address possible selection bias.

RESULTS: One hundred and twelve patients became non-persistent, most commonly due

to an adverse event (n = 37). No difference was observed in time to overall

non-persistence between sc IFN β-1a and DMF patients. Among treatment-naïve

patients, those receiving DMF had 2.4-times the risk (HR = 2.439, 95%

CI = 1.007-5.917, p = .0483) of experiencing a discontinuation than patients

receiving sc IFN β-1a. Non-persistent patients receiving DMF had 2.3-times the

risk (HR = 2.311, 95% CI = 1.350-3.958, p = .0023) of experiencing an adverse

event at a given time point than patients prescribed sc IFN β-1a. No differences

in relapse risk or ARR between sc IFN β-1a- and DMF-treated patients were

observed.

CONCLUSIONS: sc IFN β-1a-treated patients had comparable persistence and relapse

outcomes, and better safety outcomes vs DMF-treated patients across 2 years.

DOI: 10.1080/03007995.2017.1380616

PMID: 28906152

18. Exp Clin Psychopharmacol. 2017 Dec;25(6):479-484. doi: 10.1037/pha0000152.

Excluded b/c no primary data (narrative review)

On how patients with multiple sclerosis weigh side effect severity and treatment

efficacy when making treatment decisions.

Jarmolowicz DP(1), Bruce AS(2), Glusman M(3), Lim SL(3), Lynch S(4), Thelen J(3),

Catley D(5), Zieber N(3), Reed DD(6), Bruce JM(3).

Author information:

(1)Department of Applied Behavioral Science and Problem Gambling Research and

Education Support System.

(2)Department of Pediatrics, University of Kansas Medical Center.

(3)Department of Psychology, University of Missouri-Kansas City.

(4)Department of Neurology, University of Kansas Medical Center.

(5)Department of Pediatrics, Children's Mercy Hospital.

(6)Department of Applied Behavioral Science, University of Kansas.

Although effective disease-modifying treatments (DMTs) are available for

individuals suffering from multiple sclerosis (MS), many patients fail to take

their recommended medications. Unlike medications that provide immediate relief

from existing symptoms, DMTs decrease the probability of future symptoms (i.e., a

probabilistic benefit) while concurrently carrying an appreciable risk of

immediate side effects (i.e., a probabilistic cost). Prior research has shown

that both the probability of reducing disease progression and the probability of

experiencing side effects impact patients' likelihood of taking a hypothetical

DMT. The role that side effect severity plays in treatment decisions remains

unexplored. The present study examined how probability of medication efficacy and

side effect severity impact patients' likelihood of taking hypothetical DMTs.

Patients' likelihood of taking a DMT systematically decreased as medication

efficacy decreased and side effect severity increased. Because side effect

severity appears to impact decision-making processes in unique ways, the present

results suggest that providers should present information on severe (which are

typically rare) and mild to moderate side effects (which are more common)

separately. (PsycINFO Database Record

(c) 2017 APA, all rights reserved).

DOI: 10.1037/pha0000152

PMID: 29251977 [Indexed for MEDLINE]

19. Expert Opin Drug Saf. 2017 Dec;16(12):1359-1371. doi:

Excluded b/c no primary data (narrative review)

10.1080/14740338.2017.1388371. Epub 2017 Oct 12.

Drugs approved for the treatment of multiple sclerosis: review of their safety

profile.

Auricchio F(1), Scavone C(1), Cimmaruta D(1), Di Mauro G(1), Capuano A(1),

Sportiello L(1), Rafaniello C(1).

Author information:

(1)a Department of Experimental Medicine, Section of Pharmacology "L. Donatelli",

School of Medicine , University of Campania "Luigi Vanvitelli" , Naples , Italy.

INTRODUCTION: Multiple sclerosis (MS) is a chronic immune-mediated inflammatory

disorder of the brain and spinal cord characterized by inflammation,

demyelination, and axonal degeneration. Area covered: Even though the

pharmacological armamentarium for MS treatment is considerably improved in the

last 20 years, safety data especially for the second-line and innovative

treatments are lacking. In order to analyze the safety profile of drugs used for

the treatment of MS, a literature review of pre-marketing, post-marketing studies

and case reports was performed. Expert opinion: Nowadays, the numerous drugs

approved in the last years for the treatment of MS allow a better control of the

disease and a better patient compliance. The main advantages of the new

disease-modifying agents for MS (DMTs), in fact, derive from the new oral

administration and the prolonged half-life with consequent improvement in

compliance compared to first-line therapy which required subcutaneous

administrations. However, DMTs can cause serious, sometimes life-threatening or

fatal, drug adverse reactions. Due to the lack of safety data and given the

recent marketing approval of the last DMTs for MS, observational studies and

post-marketing surveillance activities will be necessary in order to improve the

knowledge about the safety profile of these drugs and the improvement of their

use in clinical practice.

DOI: 10.1080/14740338.2017.1388371

PMID: 28976217 [Indexed for MEDLINE]

20. J Neurol Neurosurg Psychiatry. 2017 Dec;88(12):1073-1078. doi:

Excluded b/c no real-world adherence/persistence

10.1136/jnnp-2017-316236. Epub 2017 Aug 26.

Dimethyl fumarate: a possible exit strategy from natalizumab treatment in

patients with multiple sclerosis at risk for severe adverse events.

Calabrese M(1), Pitteri M(1), Farina G(1), Bajrami A(1), Castellaro M(2),

Magliozzi R(1)(3), Monaco S(1).

Author information:

(1)Neurology B, Department of Neurosciences, Biomedicine and Movement, University

of Verona, Verona, Italy.

(2)Department of Information Engineering, University of Padova, Padova, Italy.

(3)Division of Brain Sciences, Faculty of Medicine, Imperial College London,

London, UK.

INTRODUCTION: Among disease-modifying treatments for multiple sclerosis,

natalizumab (NTZ) is highly effective, well tolerated and generally safe. Major

concerns regard the risk of developing progressive multifocal leukoencephalopathy

(PML), and the occurrence of rebounds or disease activity after its

discontinuation. The aim of this study was to explore the efficacy of dimethyl

fumarate (DMF) in preventing disease reactivation after NTZ discontinuation.

METHODS: Thirty-nine patients with relapsing remitting multiple sclerosis, at

high risk of PML, were switched from NTZ to DMF and underwent neurological and 3T

MRI monitoring for 2 years. Clinical and MRI data regarding the 2-year period

preceding NTZ treatment, the 2 years of NTZ treatment and the 2 years of DMF were

collected.

RESULTS: During the DMF phase, among the 39 patients, one or more relapses

occurred in five patients (12.8%), increased disability progression in 4 (10.3%)

and MRI activity in 8 (20.5%). Post-NTZ rebound effect was observed only in one

patient. Overall, only two dropouts (one rebound activity and one

gastrointestinal side effect) were registered and almost 80% of the patients have

still no evidence of disease activity at the end of DMF treatment. The multiple

linear regression model revealed that the number of relapses and MRI parameters

before DMF treatment were good predictors of disease activity during treatment

with DMF.

DISCUSSION: DMF appeared generally safe and no carryover PML among investigated

cases was observed. Although DMF did not eliminate the possibility of disease

reactivation, it seems anyway a promising drug for those patients who shall

discontinue NTZ. The clinical and radiological activity preceding the DMF

treatment might be used as a prognostic marker of therapy response.

© Article author(s) (or their employer(s) unless otherwise stated in the text of

the article) 2017. All rights reserved. No commercial use is permitted unless

otherwise expressly granted.

DOI: 10.1136/jnnp-2017-316236

PMID: 28844068 [Indexed for MEDLINE]

Conflict of interest statement: Competing interests: None declared.

21. Neurol Ther. 2017 Dec;6(2):189-196. doi: 10.1007/s40120-017-0080-x. Epub 2017 Aug

Excluded b/c no real-world adherence/persistence

5.

Effectiveness and Safety of Dimethyl Fumarate Treatment in Relapsing Multiple

Sclerosis Patients: Real-World Evidence.

Alroughani R(1), Ahmed SF(2)(3), Behbehani R(4), Al-Hashel J(2)(5).

Author information:

(1)Division of Neurology, Department of Medicine, Amiri Hospital, Sharq, Kuwait

City, Kuwait. raed.alroughani@dasmaninstitute.org.

(2)Department of Neurology, Ibn Sina Hospital, Sabah Medical Area, Kuwait City,

Kuwait.

(3)Department of Neurology and Psychiatry, Minia University, Minia, Egypt.

(4)Department of Ophthalmology, Al-Bahar Eye Center, Sabah Medical Area, Kuwait

City, Kuwait.

(5)Department of Medicine, Faculty of Medicine, Kuwait University, Jabriya,

Kuwait.

INTRODUCTION: Dimethyl fumarate (DMF) has been recently approved as a

disease-modifying therapy for the treatment of multiple sclerosis (MS).

Post-marketing studies are important to confirm what was established in clinical

trials.

OBJECTIVE: To evaluate effectiveness and safety of DMF and to measure the

occurrence of lymphopenia in a cohort of MS patients in a clinical setting.

METHODS: Using the national MS registry, we prospectively assessed relapsing MS

patients who had been prescribed DMF for at least 6 months. Primary outcome

measure was the proportion of relapse-free patients at last follow-up visit.

Secondary outcome measures were the mean change in expanded disability status

scale (EDSS) and the proportion of patients with radiological activity

(gadolinium-enhancing or new T2 lesions) at the last follow-up visit. Absolute

lymphocyte count (ALC) was assessed at baseline (within 6 months prior to DMF

initiation) and at one or more times during DMF treatment 3 months

post-initiation.

RESULTS: Of 134 patients identified, 119 were eligible and included in the

analysis. Women represented 59.7% of the studied cohort. Mean age and mean

disease duration were 33.5 ± 11.1 and 8.3 ± 7 years, respectively. A total of

75.6% of the patients received prior disease-modifying therapies. Mean duration

of DMF exposure was 20.5 ± 9.5 months. The proportion of relapse-free patients

increased significantly from 51.2% to 89.9% (p < 0.0001), while the mean EDSS

score decreased from 2.8 ± 1.8 at baseline to 2.3 ± 1.7 (p < 0.058) at last

follow-up visit. The proportion of patients with MRI activity decreased

significantly from 61.1% to 15.1% (p < 0.0001). The mean ALCs decreased from 2170

to 1430 cells/μl (34% decrease). Lymphopenia was seen in 13 (10.9%) patients, of

whom 3 (2.5%) patients had grade 3 lymphopenia necessitating discontinuation of

DMF. Although no serious adverse events were reported, 19.3% of patients

discontinued DMF.

CONCLUSION: In clinical practice, DMF appeared to be effective in reducing

disease activity and progression of disability throughout the observational

period. DMF was well tolerated with no serious adverse events. ALC profiles in

DMF-treated patients were generally stable throughout the observational period.

The proportion of patients who developed severe lymphopenia was similar to

figures in clinical trials.

DOI: 10.1007/s40120-017-0080-x

PMCID: PMC5700902

PMID: 28780745

22. Neurol Ther. 2017 Dec;6(2):175-187. doi: 10.1007/s40120-017-0077-5. Epub 2017 Aug

Excluded b/c no real-world adherence/persistence

2.

Efficacy and Tolerability of Delayed-release Dimethyl Fumarate in Black,

Hispanic, and Asian Patients with Relapsing-Remitting Multiple Sclerosis: Post

Hoc Integrated Analysis of DEFINE and CONFIRM.

Fox RJ(1), Gold R(2), Phillips JT(3), Okwuokenye M(4), Zhang A(5), Marantz JL(5).

Author information:

(1)Cleveland Clinic, Mellen Center for Multiple Sclerosis Treatment and Research,

Cleveland, OH, USA.

(2)St. Josef Hospital, Ruhr University, Bochum, Germany.

(3)Multiple Sclerosis Program, Baylor Institute for Immunology Research, Dallas,

TX, USA.

(4)Biogen, Cambridge, MA, USA. macaulay.okwuokenye@biogen.com.

(5)Biogen, Cambridge, MA, USA.

INTRODUCTION: Clinical course and treatment response may vary according to

race/ethnicity in multiple sclerosis (MS) patients. Delayed-release dimethyl

fumarate (DMF; also known as gastro-resistant DMF) demonstrated significant

efficacy and a favorable benefit-risk profile in relapsing-remitting MS (RRMS)

patients in the 2-year phase III DEFINE/CONFIRM studies.

METHODS: In this post hoc analysis of integrated data from DEFINE/CONFIRM, we

assessed clinical efficacy and safety/tolerability in black, Hispanic, and Asian

patients treated with DMF 240 mg twice daily (approved dosage) or placebo.

Eligible patients were 18-55 years of age with an Expanded Disability Status

Scale score of 0-5.0. In the integrated intention-to-treat population, 769 and

771 patients were treated with DMF or placebo, respectively, of whom 10 and 19

were black, 31 and 23 were Hispanic, and 66 and 70 were Asian.

RESULTS: In the black, Hispanic, and Asian subgroups, DMF was associated with

lower annualized relapse rates at 2 years compared with placebo [rate ratio (95%

confidence interval (CI)), 0.05 (0.00-1.07); 0.31 (0.10-0.95); and 0.64

(0.30-1.34), respectively]. The percentage of black, Hispanic, and Asian patients

with 12-week confirmed disability progression was lower with DMF (43%, 8%, and

20%, respectively) compared with placebo [57%, 30%, and 25%, respectively; hazard

ratio (95% CI), 0.53 (0.02-1.39); 0.17 (0.00-0.60); and 0.71 (0.32-1.58),

respectively]. The safety/tolerability profile of DMF was generally consistent

with that in the overall population of DEFINE/CONFIRM. The incidence of adverse

events leading to treatment discontinuation in black, Hispanic, and Asian

patients was 2/10, 2/31, and 3/66, respectively, with DMF, and 2/19, 1/23, and

8/70, respectively, with placebo.

CONCLUSION: DMF may be an efficacious treatment with a favorable benefit-risk

profile in black, Hispanic, and Asian patients with RRMS. Further clinical

studies are needed to characterize differences in MS presentation and treatment

outcomes across ethnic and racial groups.

FUNDING: Biogen.

TRIAL REGISTRATION: DEFINE: ClinicalTrials.gov identifier NCT00420212; CONFIRM

ClinicalTrials.gov identifier NCT00451451.

DOI: 10.1007/s40120-017-0077-5

PMCID: PMC5700899

PMID: 28770420

23. Neurology. 2017 Nov 28;89(22):2222-2229. doi: 10.1212/WNL.0000000000004686. Epub

Excluded b/c no oral DMD results

2017 Nov 1.

Assessing association of comorbidities with treatment choice and persistence in

MS: A real-life multicenter study.

Laroni A, Signori A, Maniscalco GT, Lanzillo R, Russo CV, Binello E, Lo Fermo S,

Repice A, Annovazzi P, Bonavita S, Clerico M, Baroncini D, Prosperini L, La Gioia

S, Rossi S, Cocco E, Frau J, Torri Clerici V, Signoriello E, Sartori A, Zarbo IR,

Rasia S, Cordioli C, Cerqua R, Di Sapio A, Lavorgna L, Pontecorvo S, Barrilà C,

Saccà F, Frigeni B, Esposito S, Ippolito D, Gallo F, Sormani MP; iMUST group.

Collaborators: Annovazzi P, Baroncini D, Barrilà C, Binello E, Bonavita S,

Bucello S, Cerqua R, Cordioli C, Clerico M, Cocco E, Di Sapio A, Esposito S, Frau

J, Frigeni B, Gallo F, Immovilli P, Ippolito D, La Gioia S, Lanzillo R, Laroni A,

Lavorgna L, Lo Fermo S, Maniscalco GT, Pontecorvo S, Prosperini L, Rasia S,

Repice A, Rossi S, Russo CV, Sartori A, Signoriello E, Clerici VT, Saccà F,

Signori A, Sormani MP, Zarbo IR.

OBJECTIVE: To assess whether the presence of concomitant diseases at multiple

sclerosis (MS) diagnosis is associated with the choice and the treatment

persistence in an Italian MS cohort.

METHODS: We included newly diagnosed patients (2010-2016) followed in 20 MS

centers and collected demographic and clinical data. We evaluated baseline

factors related to the presence of comorbidities and the association between

comorbidities and the clinical course of MS and the time to the first treatment

switch.

RESULTS: The study cohort included 2,076 patients. Data on comorbidities were

available for 1,877/2,076 patients (90.4%). A total of 449/1,877 (23.9%) patients

had at least 1 comorbidity at MS diagnosis. Age at diagnosis (odds ratio 1.05,

95% confidence interval [CI] 1.04-1.06; p < 0.001) was the only baseline factor

independently related to the presence of comorbidities. Comorbidities were not

significantly associated with the choice of the first disease-modifying

treatment, but were significantly associated with higher risk to switch from the

first treatment due to intolerance (hazard ratio 1.42, CI 1.07-1.87; p = 0.014).

Association of comorbidities with risk of switching for intolerance was

significantly heterogeneous among treatments (interferon β, glatiramer acetate,

natalizumab, or fingolimod; interaction test, p = 0.04).

CONCLUSIONS: Comorbidities at diagnosis should be taken into account at the first

treatment choice because they are associated with lower persistence on treatment.

© 2017 American Academy of Neurology.

DOI: 10.1212/WNL.0000000000004686

PMID: 29093064 [Indexed for MEDLINE]

24. J Neurol. 2017 Nov;264(11):2325-2329. doi: 10.1007/s00415-017-8595-8. Epub 2017

Ex-US

Aug 22.

Persistence to oral disease-modifying therapies in multiple sclerosis patients.

Lattanzi S(1), Danni M(2), Taffi R(2), Cerqua R(2), Carlini G(2), Pulcini A(2),

Provinciali L(2), Silvestrini M(2).

Author information:

(1)Neurological Clinic, Department of Experimental and Clinical Medicine, Marche

Polytechnic University, Via Conca 71, 60020, Ancona, Italy.

alfierelattanzisimona@gmail.com.

(2)Neurological Clinic, Department of Experimental and Clinical Medicine, Marche

Polytechnic University, Via Conca 71, 60020, Ancona, Italy.

Dimethyl fumarate (DMF), fingolimod (FTY) and teriflunomide (TFN) are oral

disease-modifying therapies (DMTs) approved for relapsing-remitting multiple

sclerosis (RRMS) whose efficacy and tolerability have been separately assessed in

phase III trials. Conversely, little evidence exists about their head-to-head

comparison. The aim of the study was to evaluate the 1-year persistence to DMF,

FTY and TFN in patients with RRMS. Patients affected by RRMS who started

treatment with DMF, FTY or TFN were identified. The study end-point was 12-month

drug persistence as time to discontinuation and proportion of patients who

discontinued medication within 1-year. A total of 307 patients were included

(DMF = 114, FTY = 129, TFN = 64). The mean times to discontinuation were 144

(84), 189 (72) and 138 (120) days in the DMF, FTY and TFN cohorts (p = 0.036). At

12-month, the proportion of patients discontinuing medication was lower for

subjects taking FTY (9.8%) compared with those starting DMF (21.9%) and TFN

(23.6%) (p = 0.020). Compared to FTY cohort, DMF [adjOR = 3.26 (1.38-7.70);

p = 0.007] and TFN [adjOR = 2.89 (1.10-7.63); p = 0.032] treated patients were

more likely to have discontinued their drug at 1-year since initiation. In

patients with RRMS, FTY was associated with a better persistence profile as

compared to DMF and TFN.

DOI: 10.1007/s00415-017-8595-8

PMID: 28831550

Excluded b/c no real-world adherence/persistence

25. Mult Scler Relat Disord. 2017 Nov;18:60-64. doi: 10.1016/j.msard.2017.09.014.

Epub 2017 Sep 21.

Delayed lymphocyte re-population following discontinuation of dimethyl fumarate

and after switching to other disease modifying drug therapies.

Khatri BO(1), Tarima SS(2), Essig B(3), Sesing J(4), Olapo T(5).

Author information:

(1)Wheaton Franciscan Healthcare, Center for Neurological Disorders, 3237 South

16th Street, Milwaukee, WI 53215, USA. Electronic address: bokhatri@aol.com.

(2)Division of Biostatistics, Medical College of Wisconsin, 9200W. Wisconsin

Avenue, Milwaukee, WI 53226, USA. Electronic address: sergey.s.tarima@gmail.com.

(3)Wheaton Franciscan Healthcare, Center for Neurological Disorders, 3237 South

16th Street, Milwaukee, WI 53215, USA. Electronic address:

benjamin.essig@ascension.org.

(4)Wheaton Franciscan Healthcare, Center for Neurological Disorders, 3237 South

16th Street, Milwaukee, WI 53215, USA. Electronic address:

jean.sesing@ascension.org.

(5)Wheaton Franciscan Healthcare, Center for Neurological Disorders, 3237 South

16th Street, Milwaukee, WI 53215, USA. Electronic address:

tayo.olapo@ascension.org.

BACKGROUND: Dimethyl fumarate (DMF) reduces absolute lymphocyte counts, CD4, and

CD8 counts, without significantly affecting total white blood cell counts.

However, the recovery rate of these cells after discontinuation of DMF is

unknown. The effect of subsequent disease modifying therapies (DMTs) on

re-population rate is also unknown.

OBJECTIVES: 1. To study the re-population rate of absolute lymphocytes, CD4, and

CD8 counts back to baseline after discontinuation of DMF. 2. To measure the

effect of subsequent DMTs on the re-population rate of these cells after DMF

therapy. 3. To study the effect of the duration of exposure to DMF on

repopulation of these cells.

METHODS: A retrospective chart review of subjects who had discontinued DMF and in

whom, CBC with differential, CD4 and CD8 counts were available at baseline,

discontinuation and at follow-up (n = 113). Linear mixed models were used to

analyze and assess linear trends in lymphocyte counts after DMF had been

discontinued.

RESULTS: DMF causes a significant drop in absolute lymphocyte, CD4, and CD8

counts. Re-population of these cells after discontinuation of DMF is

significantly delayed, irrespective of whether or not a subsequent DMT is used,

although there is a difference in re-population rate among DMTs. The

re-population rate is also dependent on the duration of time patients have been

exposed to DMF; longer exposure was associated with more delayed recovery.

CONCLUSION: During this 30 month study period, re-population rates were

significantly delayed post-DMF, irrespective of what subsequent DMT the patients

received. Furthermore, no recovery of lymphocyte counts occurred in patients who

were started on fingolimod or alemtuzumab after DMF was discontinued; in fact

there was a continued decline in all of the cell populations studied.

Copyright © 2017 Elsevier B.V. All rights reserved.

DOI: 10.1016/j.msard.2017.09.014

PMID: 29141823

26. Mult Scler Relat Disord. 2017 Nov;18:218-224. doi: 10.1016/j.msard.2017.10.001.

Ex-US

Epub 2017 Oct 6.

The impact of treatment adherence on clinical and economic outcomes in multiple

sclerosis: Real world evidence from Alberta, Canada.

Gerber B(1), Cowling T(2), Chen G(3), Yeung M(4), Duquette P(5), Haddad P(6).

Author information:

(1)Medlior Health Outcomes Research Ltd., 160 Quarry Park Blvd. SE, Suite 300,

Calgary, AB, Canada T2C 3G3. Electronic address: brittany.gerber@medlior.com.

(2)Medlior Health Outcomes Research Ltd., 160 Quarry Park Blvd. SE, Suite 300,

Calgary, AB, Canada T2C 3G3. Electronic address: tara.cowling@medlior.com.

(3)University of Calgary, Calgary, Alberta, Canada. Electronic address:

guchen@ucalgary.ca.

(4)University of Calgary, Multiple Sclerosis Clinic, Calgary, Alberta, Canada.

Electronic address: Michael.Yeung@albertahealthservices.ca.

(5)Notre Dame Hospital, Université de Montréal, Montreal, Canada. Electronic

address: pierre.duquette.1@umontreal.ca.

(6)Novartis Pharmaceuticals Canada Inc., 385, boulev. Bouchard, CDN, Dorval,

Quebec, Canada H9S 1A9. Electronic address: paola.haddad@novartis.com.

BACKGROUND: Approximately 1 in 400 Albertans has multiple sclerosis (MS). The

current study objective was to determine the real-world impact of adherence to

disease-modifying therapies (DMTs) on healthcare utilization and costs among MS

patients utilizing administrative data from the Alberta health system in Canada.

METHODS: MS patients were identified using a validated case definition (≥ 1

inpatient record or ≥ 5 practitioner claims within 2 years) and the study index

DMT was defined as the first claim for a DMT between 1 April 2011 and 31 March

2014. Treatment adherence was calculated using medication possession ratio (MPR),

and patients with MPR ≥ 80% were considered adherent; healthcare utilization and

costs were explored using multivariable negative binominal regression and

logistic regression models.

RESULTS: The majority of the 2864 MS patients identified were females, aged 35-55

years old. Overall, 66% of patients were adherent. Compared to non-adherent

patients, adherent patients had fewer ambulatory care visits (all-cause: 8.8 vs

10.9, p = 0.0012; MS-related: 4.3 vs 5.3; p = 0.001), physician visits

(all-cause: 15.1 vs 18.2, p = 0.0001; MS-related: 3.6 vs 4.4; p = 0.0001), and

hospitalizations (all-cause: 5.2% vs 10.2%, p < 0.0001; MS-related: 1.2% vs 2.5%,

p = 0.0088). After adjusting for potential confounding factors adherent patients

had approximately 20% less physician visits (MS-related: IRR 0.82 (0.79,0.86), p

< 0.0001; all-cause: IRR 0.83 (0.81,0.85), p < 0.0001) and ambulatory care visits

(MS-related IRR 0.80 (0.77,0.84), p < 0.0001; all-cause: IRR 0.82 (0.80,0.84), p

< 0.0001) and approximately 50% fewer hospitalizations (MS-related: OR 0.50

(0.28-0.89), p < 0.0001; all-cause: OR 0.48 (0.35-0.64), p < 0.0001) than

non-adherent patients.

CONCLUSIONS: The current study found a significant impact of non-adherence to MS

therapy on increased health system utilization. These findings demonstrate the

importance of treatment adherence on clinical decision-making for patients with

MS.

Copyright © 2017 Elsevier B.V. All rights reserved.

DOI: 10.1016/j.msard.2017.10.001

PMID: 29141814

27. Mult Scler Relat Disord. 2017 Nov;18:170-172. doi: 10.1016/j.msard.2017.09.029.

Excluded b/c no real-world adherence/persistence

Epub 2017 Sep 25.

"Nail loss after teriflunomide treatment: A new potential adverse event".

Mancinelli L(1), Amerio P(2), di Ioia M(3), Di Tommaso V(4), De Luca G(5), Onofrj

M(6), Lugaresi A(7).

Author information:

(1)Department of Biomedical and NeuroMotor Sciences (DIBINEM), Alma Mater

Studiorum - University of Bologna, Italy. Electronic address:

luca.mancinelli86@gmail.com.

(2)Department of Dermatology and Venereology, University "G. d'Annunzio"

Chieti-Pescara, Italy. Electronic address: p.amerio@unich.it.

(3)Department of Neuroscience Imaging and Clinical Science, University "G.

d'Annunzio" Chieti-Pescara, Italy. Electronic address: maria.diioia@unich.it.

(4)Department of Neuroscience Imaging and Clinical Science, University "G.

d'Annunzio" Chieti-Pescara, Italy. Electronic address: valeria.dtm@gmail.com.

(5)UOC Clinica Neurologica, PO "SS. Annunziata" Chieti, Italy. Electronic

address: gio.deluca05@yahoo.com.

(6)Department of Neuroscience Imaging and Clinical Science, University "G.

d'Annunzio" Chieti-Pescara, Italy. Electronic address: onofrj@unich.it.

(7)Department of Biomedical and NeuroMotor Sciences (DIBINEM), Alma Mater

Studiorum - University of Bologna, Italy; IRCCS Istituto delle Scienze

Neurologiche c/o Ospedale Bellaria - PAD Tinozzi - "UOSI Riabilitazione Sclerosi

Multipla", Via Altura, 3, 40139 Bologna, Italy. Electronic address:

alessandra.lugaresi2@unibo.it.

Nail loss might represent a new, reversible, adverse event associated with

teriflunomide treatment. It shares close analogies with hair loss and thinning,

known adverse events of teriflunomide. MS specialists should be aware of this

possibility and evaluate treatment discontinuation.

Copyright © 2017 Elsevier B.V. All rights reserved.

DOI: 10.1016/j.msard.2017.09.029

PMID: 29141803

28. Patient Prefer Adherence. 2017 Oct 20;11:1815-1830. doi: 10.2147/PPA.S140293.

Ex-US

eCollection 2017.

Adherence to fingolimod in multiple sclerosis: an investigator-initiated,

prospective, observational, single-center cohort study.

Zimmer A(1), Coslovsky M(2), Abraham I(3), Décard BF(1).

Author information:

(1)Neurologic Clinic and Policlinic, Department of Medicine, University Hospital

Basel, University of Basel, Basel.

(2)Clinical Trial Unit, Department of Clinical Research, University Hospital

Basel, University of Basel, Basel, Switzerland.

(3)Center for Health Outcomes and Pharmacoeconomic Research, University of

Arizona, Tuscon, AZ, USA.

Objectives: Adherence to multiple sclerosis (MS) treatment is essential to

optimize the likelihood of full treatment effect. This prospective,

observational, single-center cohort study investigated adherence to fingolimod

over the 2 years following treatment initiation. Two facets of adherence -

implementation and persistence - were examined and compared between new and

experienced users of disease-modifying treatments (DMTs).

Materials and methods: Implementation rates were based on the proportion of days

covered and calculated as percentages per half-yearly visits and over 2 years,

captured through refill data, pill count, and self-report. Nonadherence was

defined as taking less than 85.8% of prescribed pills. Implementation rates were

classified as nonadherent (<85.8%), suboptimally adherent (≥85.8% but <96.2%),

and optimally adherent (≥96.2%), including perfectly adherent (100%).

Persistence, ie, time until discontinuation, was analyzed by Kaplan-Meier

analysis. Reasons for discontinuation were recorded.

Results: The cohort included 98 patients with relapsing MS, all of whom received

a dedicated education session about their medication. Of these 80% were women,

31.6% had fingolimod as first DMT, and 68.4% had switched from other DMTs. The

mean implementation rate over 2 years was 98.6% (IQR1-3 98.51%-98.7%) and did not

change significantly over time; 89% of measurements were in the optimally

adherent category, 45.6% in the perfectly adherent category. There was one single

occurrence of nonadherence. New users of DMTs were 1.29 times more likely to be

adherent than experienced users (OR 1.29, 95% CI 1.11-1.51; P<0.001), but not

more persistent. Nineteen of 98 patients discontinued fingolimod.

Conclusion: The very high implementation rates displayed in this sample of MS

patients suggest that facilitation by health care professionals in preserving

adherence behavior may be sufficient for the majority of patients. Targeted

interventions should focus on patients who are nonadherent or who stop treatment

without intention to reinitiate.

DOI: 10.2147/PPA.S140293

PMCID: PMC5659224

PMID: 29118575

Conflict of interest statement: Disclosure AZ has received travel grants for

participation in investigator meetings of clinical studies and nurse meetings,

and her institution has received consultancy fees for nurse-advisory activities

by Actelion, Biogen, Genzyme, Merck Serono, Novartis, Roche, and Sanofi. She has

no conflicts of interest relative to the study reported here. MC has no conflicts

of interest to declare relative to the study reported here. IA is an

equity-holding partner in Matrix45. Matrix45 provides scientific services to

pharmaceutical companies on a nonexclusive basis. Employees may not hold equity

in sponsor organizations, and may not accept direct payments or other benefits

from sponsor organizations. As a faculty member of the University of Arizona, IA

has no conflicts of interest to declare relative to the study reported here. BFD

received for the institution (University Hospital Basel) advisory board or

speaker fees from Biogen, Teva and Novartis, which were used exclusively for

research support. BFD received travel support from Novartis, Biogen, and Genzyme.

BFD has no conflicts of interests in this work.

29. Patient Prefer Adherence. 2017 Oct 19;11:1789-1796. doi: 10.2147/PPA.S142373.

Excluded b/c no real-world adherence/persistence

eCollection 2017.

The impact of quality of life on treatment preferences in multiple sclerosis

patients.

Lee Mortensen G(1), Rasmussen PV(2).

Author information:

(1)Medical Anthropology Department, AnthroConsult.

(2)Department of Neurology, University Hospital of Aarhus, Aarhus C, Denmark.

INTRODUCTION: Multiple sclerosis (MS) is a demyelinating disorder with an

unpredictable and often disabling course. MS symptoms are very heterogeneous and

may lead to reduced physical, cognitive, and psychosocial functioning decreasing

patients' quality of life (QoL). Today, various disease-modifying treatments

(DMTs) may prevent disease progression. However, it is increasingly complex to

select the right therapy for a given patient and patient preferences should be

considered when making treatment decisions. This study aimed to explore the main

factors affecting patients' preferences regarding MS treatment and health care.

METHODS: Five qualitative focus group interviews were carried out with a total of

40 participants from across Denmark. A semistructured question guide included

questions that were identified in a systematic literature study about QoL and

treatment preferences in patients with MS. The participants were asked to

describe their disease experiences, their health-related QoL, and reasons behind

their preferences with regard to treatment and care. The data were analyzed using

content analysis and a constructivist approach.

RESULTS: The participants' physical, cognitive, and psychosocial QoL and

functioning were reduced by disease symptoms, treatment side effects, and mode of

administration. Their ability to uphold meaningful role functioning was crucial

to their treatment priorities. The preeminence of anticipated efficacy, ie, the

patients' hope that DMT might prevent disease deterioration in the future, was

modified by their present QoL and functioning when ultimately framing their

treatment preferences. There was an unmet information and support need from

neurology clinics, particularly at the time of diagnosis.

CONCLUSION: The participants' treatment preferences were influenced by a matrix

of treatment and QoL-related factors and evolved with time and along with

personal and professional changes in life. The patients preferred to receive a

clear recommendation of DMT from the neurologist taking into account their

individual functioning and present QoL priorities.

DOI: 10.2147/PPA.S142373

PMCID: PMC5656344

PMID: 29089746

Conflict of interest statement: Disclosure GLM is the owner of AnthroConsult who

received an unrestricted research grant from Biogen Idec for the present study.

AnthroConsult has previously received research grants and/or honoraria from

Sanofi Pasteur MSD, Amgen, Pfizer, and Lundbeck A/S, though none related to

studies of neurological disorders. PVR received a personal fee from Biogen Idec

related to the present study. Within the past 2 years, he has participated in

advisory board meetings with Biogen, Novartis, TEVA, Allergan, Roche, and

Genzyme, acted as educator for TEVA, Allergan, and Novartis, and undertook

letterbox assignments for Biogen. The authors report no other conflicts of

interest in this work.

30. PLoS One. 2017 Oct 19;12(10):e0185766. doi: 10.1371/journal.pone.0185766.

Excluded b/c no real-world adherence/persistence

eCollection 2017.

Treatment satisfaction with injectable disease-modifying therapies in patients

with relapsing-remitting multiple sclerosis (the STICK study).

Fernández O(1), Duran E(2), Ayuso T(3), Hernández L(4), Bonaventura I(5), Forner

M(6); STICK Study Investigators Group.

Author information:

(1)Institute of Biomedical Research (IBIMA), Regional University Hospital,

Malaga, Spain.

(2)Juan Ramon Jimenez-Infanta Elena Hospitals, Huelva, Spain.

(3)Navarra Hospital, Pamplona, Spain.

(4)León Hospital, León, Spain.

(5)Mutua Terrassa, Terrasa, Spain.

(6)Sanofi Genzyme, Barcelona, Spain.

BACKGROUND: Treatment satisfaction in patients with relapsing-remitting multiple

sclerosis (RRMS) may impact adherence and thus clinical outcomes. The objective

of this study was to measure the satisfaction of patients with RRMS with

injectable disease-modifying therapies (DMTs) and to evaluate the factors

associated with treatment satisfaction.

MATERIAL AND METHODS: In this observational retrospective study conducted in the

neurology departments of 35 hospitals throughout Spain, demographic data, disease

characteristics, and information on treatment with injectable DMTs were collected

at a single scheduled visit. Treatment satisfaction was assessed using the

Treatment Satisfaction Questionnaire for Medication (TSQM), version 1.4. Patients

also answered complementary questions about the factors that might affect

treatment satisfaction. The data collected were analyzed descriptively. A

regression model was used to explore the factors associated with treatment

satisfaction.

RESULTS: The study included 445 patients (mean±SD age, 41±10.2 years; two-thirds

women). The percentages treated with each DMT were Avonex 28.5%, Rebif 44 μg

24.5%, Copaxone 22.5%, Betaferon 13.0%, Rebif22 μg 8.3% and Extavia 3.1%. The

mean±SD overall satisfaction according to the TSQM was 68.8±18.6 and the highest

overall satisfaction was reported for Rebif 22 μg (72.4±20.3) and the lowest for

Extavia (61.7±23.7). In the regression analysis, rehabilitation, interference

with social life, pain on injection and number of MS treatments received were

significantly associated with a decrease in overall TSMQ score. A small but

significant negative correlation was found between EDSS scores and TSMQ scores

(rho = -0.11, p = 0.02) and effectiveness (rho = -0.17, p<0.001). A perceived

inconvenience of injections was reflected by the stated preference of 83% for

once-daily oral treatment over other administration routes.

CONCLUSIONS: Patients on stable injectable DMT therapy were reasonably satisfied

with their treatment. Our results suggest that the main source of dissatisfaction

with the current treatment is the inconvenience of the administration regimen.

DOI: 10.1371/journal.pone.0185766

PMCID: PMC5648132

PMID: 29049356 [Indexed for MEDLINE]

31. Drugs. 2017 Oct;77(16):1755-1768. doi: 10.1007/s40265-017-0814-1.

Excluded b/c no primary data (narrative review)

Benefit-Risk Profile of Sphingosine-1-Phosphate Receptor Modulators in Relapsing

and Secondary Progressive Multiple Sclerosis.

Comi G(1), Hartung HP(2)(3), Bakshi R(4), Williams IM(5), Wiendl H(6).

Author information:

(1)Department of Neurology and INSPE, Scientific Institute Hospital San Raffaele,

Vita-Salute San Raffaele University, Milan, Italy. comi.giancarlo@hsr.it.

(2)Department of Neurology, Medical Faculty, Heinrich Heine University,

Düsseldorf, Germany.

(3)Center for Neuropsychiatry, LVR Klinikum, Düsseldorf, Germany.

(4)Novartis Pharma AG, Basel, Switzerland.

(5)Oxford PharmaGenesis Ltd, Oxford, UK.

(6)Department of Neurology, University Hospital Münster, Münster, Germany.

Since the approval of fingolimod, several selective sphingosine-1-phosphate

receptor modulators have entered clinical development for multiple sclerosis.

However, side effects can occur with sphingosine-1-phosphate receptor modulators.

By considering short-term data across the drug class and longer term fingolimod

data, we aim to highlight the potential of sphingosine-1-phosphate receptor

modulators in multiple sclerosis, while offering reassurance that their

benefit-risk profiles are suitable for long-term therapy. Short-term fingolimod

studies demonstrated the efficacy of this drug class, showed that cardiac events

upon first-dose administration are transient and manageable, and showed that

serious adverse events are rare. Early-phase studies of selective

sphingosine-1-phosphate receptor modulators also show efficacy with a similar or

improved safety profile, and treatment initiation effects were reduced with dose

titration. Longer term fingolimod studies demonstrated sustained efficacy and

raised no new safety concerns, with no increases in macular edema, infection, or

malignancy rates. Switch studies identified no safety concerns and greater

patient satisfaction and persistence with fingolimod when switching from

injectable therapies with no washout period. Better outcomes were seen with short

than with long washouts when switching from natalizumab. The specific

immunomodulatory effects of sphingosine-1-phosphate receptor modulators are

consistent with the low observed rates of long-term, drug-related adverse effects

with fingolimod. Short-term data for selective sphingosine-1-phosphate receptor

modulators support their potential effectiveness in multiple sclerosis, and

improved side-effect profiles may widen patient access to this drug class. The

long-term safety, tolerability, and persistence profiles of fingolimod should

reassure clinicians that sphingosine-1-phosphate receptor modulators are likely

to be suitable for the long-term treatment of multiple sclerosis.

DOI: 10.1007/s40265-017-0814-1

PMCID: PMC5661009

PMID: 28905255

Excluded b/c no primary data (narrative review)

32. J Am Assoc Nurse Pract. 2017 Oct;29(10):629-638. doi: 10.1002/2327-6924.12514.

Treatment and disease management of multiple sclerosis patients: A review for

nurse practitioners.

Roman C(1), Menning K(1).

Author information:

(1)Rocky Mountain MS Clinic, Salt Lake City, Utah.

BACKGROUND AND PURPOSE: This review discusses the role of the nurse practitioner

(NP) in evaluating the clinical effects, potential side effects, and monitoring

requirements for treatment options in multiple sclerosis (MS) and provides

guidance on how to help patients understand these issues.

METHODS: A literature search was conducted on PubMed to identify publications on

monitoring and disease management of MS patients. Additional resources included

drug information web sites and package inserts.

CONCLUSIONS: NPs play an active role in the management of MS patients via

effective monitoring and communication throughout the patient's treatment regimen

and disease course. In the shared decision-making model of MS treatment, NPs

ensure that patients understand the implications of their disease-modifying

therapies (DMTs). As patients move through treatments during the course of their

disease, the importance of this role increases, and it is critical that NPs

follow the guidelines in each medication's product label and take into account

any potential lingering effects of prior medications.

IMPLICATIONS FOR PRACTICE: It is critical for NPs to promote patient adherence,

to ensure that patients understand treatment side effects and monitoring

requirements, and to take sequencing and reversibility implications of DMTs into

account when making clinical decisions.

©2017 American Association of Nurse Practitioners.

DOI: 10.1002/2327-6924.12514

PMID: 29029375 [Indexed for MEDLINE]

Excluded b/c no real-world adherence/persistence

33. Mult Scler Relat Disord. 2017 Oct;17:123-127. doi: 10.1016/j.msard.2017.07.007.

Epub 2017 Jul 6.

Clinical and MRI outcomes after stopping or switching disease-modifying therapy

in stable MS patients: a case series report.

Berkovich R(1).

Author information:

(1)MS Comprehensive Care Center at the University of Southern California Keck

School of Medicine of USC, USA. Electronic address: rberkovi@usc.edu.

OBJECTIVE: To evaluate clinical and MRI outcomes after stopping or switching

disease-modifying therapy in patients with stable MS.

METHODS: A retrospective chart review was conducted of stable MS patients who

discontinued or switched their DMT from 2011 to 2015. Clinical and MRI outcomes

were obtained at baseline and 1-year follow-up.

RESULTS: For the DMT discontinuation group, 15 patients were included, with 67%

female, 53% Caucasian, mean age of 45.3 ± 12.2 years, disease duration of 9.1 ±

4.3 years, MS type (80% RRMS, 20% SPMS), and EDSS of 3.7 ± 1.6. The average

duration of stable MS course was 5.5 ± 3.7 years. Within a mean of 6.4 ± 2.2

months after DMT discontinuation, all 15 patients experienced worsening of MS

disease. After re-evaluation of MS treatment options, all 15 patients were

restarted on DMT, of which, 6 (40%) restarted on their prior DMT, 4 (26.7%)

switched to another DMT due to adverse events on prior DMT, and 5 (33.3%)

switched to a more potent DMT due to worsening of MS activity. One year follow-up

showed 2 patients (13.3%) who were restarted on their prior DMT experienced a

relapse and the remaining 13 patients (86.7%) had no clinical or MRI activities.

For the DMT switch group, 23 patients were included, with 65% female, 61%

Caucasian, a mean age of 46.9 ± 11.6 years, disease duration of 11.7 ± 5.1 years,

MS Type (83% RRMS, 17% SPMS), and EDSS of 3.5 ± 0.9. After switching DMT, 9

(39.1%) patients experienced worsening of clinical or MRI outcomes at the 1-year

follow-up. Of the 9 switch failures, the majority (N = 6) were due to switching

to dimethyl fumarate.

CONCLUSION: DMT discontinuation in stable MS patients resulted in worsening of MS

disease course for all patients, which improved upon DMT restart or switch. In

contrast, 39% of MS stable patients experienced worsening of MS disease course

when switched to another DMT, with DMT selection potentially impacting switch

outcomes.

Copyright © 2017 Elsevier B.V. All rights reserved.

DOI: 10.1016/j.msard.2017.07.007

PMID: 29055441

34. Mult Scler Relat Disord. 2017 Oct;17:107-115. doi: 10.1016/j.msard.2017.07.006.

Excluded b/c no real-world adherence/persistence

Epub 2017 Jul 6.

Patient-reported outcomes in relapsing forms of MS: Real-world, global treatment

experience with teriflunomide from the Teri-PRO study.

Coyle PK(1), Khatri B(2), Edwards KR(3), Meca-Lallana JE(4), Cavalier S(5), Rufi

P(6), Benamor M(7), Brette S(8), Robinson M(9), Gold R(10); Teri-PRO Trial Group.

Author information:

(1)Department of Neurology, Stony Brook University, HSC T12-020, Stony Brook, NY

11794-8121, USA. Electronic address: Patricia.Coyle@stonybrookmedicine.edu.

(2)The Regional MS Center, Center for Neurological Disorders, Wheaton Franciscan

Health Care, 3237 S 16th St, Milwaukee, WI 53215, USA. Electronic address:

bokhatri@aol.com.

(3)Multiple Sclerosis Center of Northeastern New York, 1205 Troy-Schenectady Rd,

Ste 105, Latham, NY 12110, USA. Electronic address: kedwards@tristateneuro.com.

(4)Hospital Virgen de la Arrixaca, Ctra., Madrid-Cartagena, s/n, 30120 Murcia,

Spain; Cátedra de Neuroinmunología Clínica y Esclerosis Múltiple, UCAM

Universidad Católica San Antonio de Murcia, Campus de los Jerónimos, Guadalupe,

30107 Murcia, Spain. Electronic address: pmecal@gmail.com.

(5)Sanofi Genzyme, 500 Kendall Street, 6th Floor, Cambridge, MA 02142, USA.

Electronic address: steven.cavalier@sanofi.com.

(6)Sanofi Genzyme, 1 Avenue Pierre Brossolette, 91385 Chilly-Mazarin, France.

Electronic address: pascal.rufi@sanofi.com.

(7)Sanofi Genzyme, 1 Avenue Pierre Brossolette, 91385 Chilly-Mazarin, France.

(8)Aixial, 4 Rue Barthelemy Danjou, 92100 Boulogne-Billancourt, France.

Electronic address: Sandrine.brette@sanofi.com.

(9)Sanofi, 55 Corporate Drive, Bridgewater, NJ 08807, USA. Electronic address:

Miqun.Robinson@sanofi.com.

(10)St Josef Hospital, Ruhr University Bochum, 5092414 Gudrunstrasse 56, D-44791

Bochum, Germany. Electronic address: ralf.gold@ruhr-uni-bochum.de.

BACKGROUND: Patient-reported outcomes (PROs) provide clinicians with further

understanding of the impact of treatment on patients' daily lives. In addition,

real-world studies, which employ broader inclusion criteria than randomized

trials, may help to inform prescribing decisions when selecting a

disease-modifying therapy (DMT) to treat relapsing forms of MS (RMS). We sought

to use PROs to determine patient treatment satisfaction and other treatment

outcomes, and report safety and tolerability associated with teriflunomide, in

the global, phase 4 Teri-PRO study (NCT01895335).

METHODS: Patients with RMS (N = 1000) received teriflunomide for 48 weeks per

local labeling. The primary endpoint was Global Satisfaction with teriflunomide

treatment measured by the Treatment Satisfaction Questionnaire for Medication

(TSQM, V1.4). Secondary endpoints included TSQM scores at Week (W)48 vs baseline

in patients switching to teriflunomide from other DMTs ('switchers'), additional

PROs, and safety.

RESULTS: Mean TSQM Global Satisfaction score at W48 was high (68.2). Switchers

reported significant improvements across all four TSQM domains at W48 vs baseline

(all p < 0.0001). Adverse events were consistent with teriflunomide clinical

trials.

CONCLUSION: Patients reported high treatment satisfaction with teriflunomide,

with switchers also reporting improved treatment satisfaction vs baseline. High

treatment satisfaction in patients with RMS may lead to improved adherence, and

hence treatment outcomes.

Copyright © 2017 The Authors. Published by Elsevier B.V. All rights reserved.

DOI: 10.1016/j.msard.2017.07.006

PMID: 29055438

35. Neurotherapeutics. 2017 Oct;14(4):1134-1147. doi: 10.1007/s13311-017-0550-y.

Excluded b/c no real-world adherence/persistence

Fingolimod Exerts only Temporary Antiepileptogenic Effects but Longer-Lasting

Positive Effects on Behavior in the WAG/Rij Rat Absence Epilepsy Model.

Leo A(1), Citraro R(1), Amodio N(2), De Sarro C(1), Gallo Cantafio ME(2),

Constanti A(3), De Sarro G(1), Russo E(4).

Author information:

(1)Science of Health Department, School of Medicine, University "Magna Graecia"

of Catanzaro, Catanzaro, Italy.

(2)Department of Experimental and Clinical Medicine, Magna Graecia University and

Translational Medical Oncology Unit, Salvatore Venuta University Campus,

Catanzaro, Italy.

(3)Department of Pharmacology, UCL School of Pharmacy, London, UK.

(4)Science of Health Department, School of Medicine, University "Magna Graecia"

of Catanzaro, Catanzaro, Italy. erusso@unicz.it.

One of the major challenges in the epilepsy field is identifying

disease-modifying drugs in order to prevent or delay spontaneous recurrent

seizure onset or to cure already established epilepsy. It has been recently

reported that fingolimod, currently approved for the treatment of

relapsing-remitting multiple sclerosis, has demonstrated antiepileptogenic

effects in 2 different preclinical models of acquired epilepsy. However, to date,

no data exist regarding the role of fingolimod against genetic epilepsy.

Therefore, we have addressed this issue by studying the effects of fingolimod in

Wistar Albino Glaxo/Rijswijk (WAG/Rij) rats, a well-established genetic model of

absence epilepsy, epileptogenesis, and neuropsychiatric comorbidity. Our results

have demonstrated that an early long-term treatment with fingolimod

(1 mg/kg/day), started before absence seizure onset, has both antiepileptogenic

and antidepressant-like effects in WAG/Rij rats. However, these effects were

transitory, as 5 months after treatment discontinuation, both absence seizure and

depressive like-behavior returned to control levels. Furthermore, a temporary

reduction of mTOR signaling pathway activity, indicated by reduced phosphorylated

mammalian target of rapamycin and phosphorylated p70S6k levels, and by increased

phosphorylated Akt in WAG/Rij rats of 6 months of age accompanied the transitory

antiepileptogenic effects of fingolimod. Surprisingly, fingolimod has

demonstrated longer-lasting positive effects on cognitive decline in this strain.

This effect was accompanied by an increased acetylation of lysine 8 of histone H4

(at both 6 and 10 months of age). In conclusion, our results support the

antiepileptogenic effects of fingolimod. However, the antiepileptogenic effects

were transitory. Moreover, fingolimod might also have a positive impact on animal

behavior and particularly in protecting the development of memory decline.

DOI: 10.1007/s13311-017-0550-y

PMCID: PMC5722759 [Available on 2018-10-01]

PMID: 28653281

36. Mult Scler J Exp Transl Clin. 2017 Sep 27;3(3):2055217317730096. doi:

Excluded b/c no real-world adherence/persistence

10.1177/2055217317730096. eCollection 2017 Jul-Sep.

A comparison of multiple sclerosis disease activity after discontinuation of

fingolimod and placebo.

Vermersch P(1), Radue EW(2), Putzki N(3), Ritter S(3), Merschhemke M(4), Freedman

MS(5).

Author information:

(1)University of Lille, CHU Lille, LIRIC - INSERM U995, FHU Imminent, France.

(2)Medical Image Analysis Center, University Hospital Basel, Switzerland.

(3)Novartis Pharmaceuticals Corporation, USA.

(4)Novartis Pharma AG, Switzerland.

(5)University of Ottawa, The Ottawa Hospital Research Institute, Canada.

BACKGROUND: Cases of higher-than-expected disease activity have been reported

following fingolimod discontinuation.

OBJECTIVE: The objective of this paper is to assess the risk of substantially

higher-than-expected disease activity post-study drug discontinuation (SDD) at

the individual patient level using data from the Phase III, placebo-controlled

FREEDOMS and FREEDOMS II trials.

METHODS: Baseline gadolinium-enhancing T1-lesion volumes were used to

statistically model the expected level of MRI disease activity post-SDD. Patients

exceeding this level were classed as "MRI outliers." Patients with an unusually

high increase in Expanded Disability Status Scale score, hospitalization for

relapse, severe relapse, or relapse with incomplete recovery post-SDD were

classed as "clinical outliers."

RESULTS: In FREEDOMS, the number of MRI outliers post-SDD was 2/69 (2.9%), 1/65

(1.5%) and 7/83 (8.4%) for the placebo, fingolimod 0.5 mg, and fingolimod 1.25 mg

groups, respectively. In FREEDOMS II, the corresponding numbers were 4/72 (5.6%),

6/79 (7.6%) and 3/73 (4.1%). The number of clinical outliers across both trials

was low. No consistent evidence of placebo vs fingolimod, dose-related or

inter-trial patterns was discernable.

CONCLUSION: The low number of clinical and MRI outliers and lack of any

discernible pattern within and between trials, including between placebo and

fingolimod, argues against a systematic risk of higher-than-expected recurrence

of disease activity following discontinuation of fingolimod.

DOI: 10.1177/2055217317730096

PMCID: PMC5624444

PMID: 28989795

37. J Neurol Sci. 2017 Sep 15;380:79-81. doi: 10.1016/j.jns.2017.07.014. Epub 2017

Excluded b/c no real-world adherence/persistence

Jul 9.

Rebound syndrome after teriflunomide cessation in a patient with multiple

sclerosis.

Yamout BI(1), Said M(2), Hannoun S(2), Zeineddine M(2), Massouh J(2), Khoury

SJ(2).

Author information:

(1)Nehme and Therese Tohme Multiple Sclerosis Center, American University of

Beirut Medical Center, Beirut, Lebanon. Electronic address: yamoutba@gmail.com.

(2)Nehme and Therese Tohme Multiple Sclerosis Center, American University of

Beirut Medical Center, Beirut, Lebanon.

We report a case of relapsing remitting multiple sclerosis (RRMS) with severe

rebound syndrome 12weeks following discontinuation of teriflunomide therapy. The

patient developed severe clinical relapses with significant increase in the

number of brain and spine magnetic resonance imaging (MRI) lesions. She responded

well to intravenous and oral steroids and was later maintained on rituximab.

Copyright © 2017 Elsevier B.V. All rights reserved.

DOI: 10.1016/j.jns.2017.07.014

PMID: 28870594

38. BMC Neurol. 2017 Sep 6;17(1):174. doi: 10.1186/s12883-017-0953-8.

Excluded b/c no oral DMD results

Adherence, satisfaction and functional health status among patients with multiple

sclerosis using the BETACONNECT® autoinjector: a prospective observational cohort

study.

Kleiter I(1)(2), Lang M(3), Jeske J(4), Norenberg C(5), Stollfuß B(6), Schürks

M(7).

Author information:

(1)St. Josef Hospital, University Hospital Bochum, Bochum, Germany.

(2)Present Address: Marianne-Strauß-Klinik, Behandlungszentrum Kempfenhausen für

Multiple Sklerose Kranke, Berg, Germany.

(3)Joint Neurological Practice, Ulm, Germany.

(4)Neurological Practice, Wuppertal, Germany.

(5)Bayer AG, Wuppertal, Germany.

(6)Bayer Vital GmbH, Leverkusen, Germany.

(7)Bayer Vital GmbH, Leverkusen, Germany. markus.schuerks@bayer.com.

BACKGROUND: Maintaining patient adherence to disease modifying drugs in multiple

sclerosis is a challenge, which can be improved by autoinjectors. The

BETACONNECT® is a fully electronic autoinjector for the injection of interferon

beta-1b (IFN beta-1b) automatically recording injections.

METHODS: The BETAEVAL study was a prospective, observational, cohort study over

24 weeks among patients with relapsing remitting multiple sclerosis or clinically

isolated syndrome treated with IFN beta-1b in Germany using the BETACONNECT®. The

primary aim was to investigate treatment adherence, secondary aims included

assessing satisfaction and functional health status. Adherence was evaluated from

injection data recorded by the device. Patient-related data were obtained from

clinical examinations and patient questionnaires.

RESULTS: Of the 151 patients enrolled, 143 were available for analysis.

Thirty-four patients discontinued the study prematurely. 107/143 (74.8%) patients

still used the BETACONNECT® at the end of the study. Injection data from the

device at any visit was available for 107 patients. Among those, the percentage

of adherent patients injecting ≥80% of doses and still participating in the study

was 57.9% at week 24. 29% of patients prematurely stopped the study, 13.1%

injected <80%. Among patients with BETACONNECT® data at the respective visit, the

proportion of adherent patients was high over the entire study period (week 4:

81.1% [N = 95], week 12: 86.7% [N = 83], week 24: 80.5% [N = 77]). Participants

(N = 143) indicated high satisfaction with the BETACONNECT®. At week 24, 98.0% of

patients who completed the corresponding questionnaire (strongly) agreed that it

was user-friendly, 81.2% felt confident in using it compared to their previous

way and 85.5% preferred it to their previous way of injection. Injection-related

pain was rated as mild to moderate at all follow-up visits. Whereas 17.2% of

patients with corresponding questionnaire indicated using analgesics prior to

injection at week 4, only 9.1% did at week 24. Outcomes from questionnaires

assessing functional health status, depression, fatigue and cognitive function

were very similar throughout the study course.

CONCLUSIONS: The majority of patients continued using the BETACONNECT® for IFN

beta-1b treatment during the 24-week study period. Adherence was high among

participants still using the BETACONNECT® and patients were highly satisfied with

the device. Ongoing studies will evaluate long-term adherence and treatment

outcomes in patients using the BETACONNECT®.

TRIAL REGISTRATION: clinicaltrails.gov NCT02121444 (registered April 22, 2014).

DOI: 10.1186/s12883-017-0953-8

PMCID: PMC5588619

PMID: 28877664 [Indexed for MEDLINE]

39. Neurology. 2017 Sep 5;89(10):1050-1059. doi: 10.1212/WNL.0000000000004330. Epub

Excluded b/c no real-world adherence/persistence

2017 Aug 9.

Anti-inflammatory disease-modifying treatment and short-term disability

progression in SPMS.

Lorscheider J, Jokubaitis VG, Spelman T, Izquierdo G, Lugaresi A, Havrdova E,

Horakova D, Trojano M, Duquette P, Girard M, Prat A, Grand'Maison F, Grammond P,

Pucci E, Boz C, Sola P, Ferraro D, Spitaleri D, Lechner-Scott J, Terzi M, Van

Pesch V, Iuliano G, Bergamaschi R, Ramo-Tello C, Granella F, Oreja-Guevara C,

Butzkueven H, Kalincik T; MSBase Study Group.

Collaborators: Hupperts R, Bolaños RF, Rio ME, CabreraGomez JA, Verheul F, Slee

M, McCombe P, Olascoaga J, Saladino ML, Amato MP, Alroughani R, Cristiano E, Deri

N, Sánchez Menoyo JL, Hodgkinson S, Flechter S, Moore F, Petersen T, Ampapa R,

Gray O, Skibina O, Csepan T, Singhal B, Braber-Moerland LD, Sirbu CA, Vucic S,

Arruda WO, Prévost J, Kasa K, Kermode A, Barnett MH, Shuey N, Imre P, Daskalovska

V, Vella N.

OBJECTIVE: To investigate the effect of disease-modifying treatment on short-term

disability outcomes in secondary progressive multiple sclerosis (SPMS).

METHODS: Using MSBase, an international cohort study, we previously validated a

highly accurate definition of SPMS. Here, we identified patients in MSBase who

were either untreated or treated with a disease-modifying drug when meeting this

definition. Propensity score matching was used to select subpopulations with

comparable baseline characteristics. Disability outcomes were compared in paired,

pairwise-censored analyses adjusted for treatment persistence, visit density, and

relapse rates.

RESULTS: Of the 2,381 included patients, 1,378 patients were matchable (treated n

= 689, untreated n = 689). Median pairwise-censored follow-up was 2.1 years

(quartiles 1.2-3.8 years). No difference in the risk of 6-month sustained

disability progression was observed between the groups (hazard ratio [HR] 0.9,

95% confidence interval [CI] 0.7-1.1, p = 0.27). We also did not find differences

in any of the secondary endpoints: risk of reaching Expanded Disability Status

Scale (EDSS) score ≥7 (HR 0.6, 95% CI 0.4-1.1, p = 0.10), sustained disability

reduction (HR 1.0, 95% CI 0.8-1.3, p = 0.79), or change in disability burden

(area under the EDSS-time curve, β = -0.05, p = 0.09). Secondary and sensitivity

analyses confirmed the results.

CONCLUSIONS: Our pooled analysis of the currently available disease-modifying

agents used after conversion to SPMS suggests that, on average, these therapies

have no substantial effect on relapse-unrelated disability outcomes measured by

the EDSS up to 4 years.

CLASSIFICATION OF EVIDENCE: This study provides Class IV evidence that for

patients with SPMS, disease-modifying treatment has no beneficial effect on

short-term disability progression.

© 2017 American Academy of Neurology.

DOI: 10.1212/WNL.0000000000004330

PMCID: PMC5589791 [Available on 2018-09-05]

PMID: 28794248 [Indexed for MEDLINE]

40. Brain. 2017 Sep 1;140(9):2426-2443. doi: 10.1093/brain/awx185.

Excluded b/c no real-world adherence/persistence

Towards personalized therapy for multiple sclerosis: prediction of individual

treatment response.

Kalincik T(1)(2), Manouchehrinia A(3), Sobisek L(4)(5), Jokubaitis V(2)(6),

Spelman T(2)(6), Horakova D(4), Havrdova E(4), Trojano M(7), Izquierdo G(8),

Lugaresi A(9)(10), Girard M(11), Prat A(11), Duquette P(11), Grammond P(12), Sola

P(13), Hupperts R(14), Grand'Maison F(15), Pucci E(16), Boz C(17), Alroughani

R(18), Van Pesch V(19), Lechner-Scott J(20), Terzi M(21), Bergamaschi R(22),

Iuliano G(23), Granella F(24), Spitaleri D(25), Shaygannejad V(26), Oreja-Guevara

C(27), Slee M(28), Ampapa R(29), Verheul F(30), McCombe P(31), Olascoaga J(32),

Amato MP(33), Vucic S(34), Hodgkinson S(35), Ramo-Tello C(36), Flechter S(37),

Cristiano E(38), Rozsa C(39), Moore F(40), Luis Sanchez-Menoyo J(41), Laura

Saladino M(42), Barnett M(43), Hillert J(3), Butzkueven H(2)(6)(44); MSBase Study

Group.

Author information:

(1)CORe, Department of Medicine, University of Melbourne, 300 Grattan St,

Melbourne, 3050, Australia.

(2)Department of Neurology, Royal Melbourne Hospital, 300 Grattan St, Melbourne,

3050, Australia.

(3)Department of Clinical Neuroscience, Karolinska Institutet, Stockholm,

SE-17177, Sweden.

(4)Department of Neurology and Center of Clinical Neuroscience, General

University Hospital and Charles University in Prague, Katerinska 30, Prague,

12808, Czech Republic.

(5)Department of Statistics and Probability, University of Economics in Prague,

Winston Churchill Sq 1938/4, Prague, 13067, Czech Republic.

(6)Department of Medicine, University of Melbourne, 300 Grattan St, Melbourne,

3050, Australia.

(7)University of Bari, Via Calefati 53, Bari, 70122, Italy.

(8)Hospital Universitario Virgen Macarena, Amador de los Rios 48-50. 4a, Sevilla,

41003, Spain.

(9)Department of Neuroscience, Imaging and Clinical Sciences, University 'G.

d'Annunzio', Via dei Vestini, Chieti, 66100, Italy.

(10)Department of Biomedical and Neuromotor Sciences, University of Bologna, Via

dei Vestini, Bologna, 66100, Italy.

(11)Hopital Notre Dame, 1560 Sherbrooke East, Montreal, H2L 4M1, Canada; CHUM and

Universite de Montreal, Montreal, Canada.

(12)Centre de réadaptation déficience physique Chaudière-Appalache, 9500 blvd

Centre-Hospitalier, Levis, G6X 0A1, Canada.

(13)Nuovo Ospedale Civile Sant'Agostino/Estense, via giardini 1355, Modena,

41100, Italy.

(14)Zuyderland Ziekenhuis, Walramstraat 23, Sittard, 6131 BK, The Netherlands.

(15)Neuro Rive-Sud, 4896 boul. Taschereau, suite 250, Greenfield Park, J4V 2J2,

Canada.

(16)Azienda Sanitaria Unica Regionale Marche - AV3, Via Santa Lucia 2, Macerata,

62100, Italy.

(17)KTU Medical Faculty Farabi Hospital, Karadeniz Technical University, Trabzon,

61080, Turkey.

(18)Amiri Hospital, P.O. Box 1661. Qurtoba, Kuwait, 73767, Kuwait.

(19)Cliniques Universitaires Saint-Luc, avenue Hippocrate, 10 UCL10/80, Brussels,

1200 BXL, Belgium.

(20)University of Newcastle, Lookout Road, Newcastle, 2305, Australia.

(21)Ondokuz Mayis University, Medical Faculty, Kurupelit, Samsun, 55160, Turkey.

(22)C. Mondino National Neurological Institute, via Mondino 2, Pavia, 27100,

Italy.

(23)Ospedali Riuniti di Salerno, Via s. Leonardo, Salerno, 84100, Italy.

(24)University of Parma, Via Gramsci, 14, Parma, 43100, Italy.

(25)Azienda Ospedaliera di Rilievo Nazionale San Giuseppe Moscati Avellino,

Contrada Amoretta, Avellino, 83100, Italy.

(26)Isfahan University of Medical Sciences, Soffeh St, Isfahan, 81744, Iran.

(27)Hospital Universitario La Paz, Paseo de la Castellana 261, Madrid, 28050,

Spain.

(28)Flinders Medical Centre, Flinders Drive, Adelaide, 5042, Australia.

(29)Nemocnice Jihlava, Vrchlickeho 59, Jihlava, 58633, Czech Republic.

(30)Groene Hart ziekenhuis, bleulandweg 10, Gouda, 2800 BB, The Netherlands.

(31)Royal Brisbane and Women's Hospital, 33 North Street, Spring Hill, QLD 4000,

Australia.

(32)Hospital Donostia, Paseo de Begiristain, San Sebastián, 20014, Spain.

(33)University of Florence, Viale Morgagni 85, Florence, 50134, Italy.

(34)Westmead Hospital, Hawkesbury Rd, Sydney, 2145, Australia.

(35)Liverpool Hospital, Elizabeth St, Liverpool, 21, Australia.

(36)Hospital Germans Trias i Pujol, Crtra de Canyet s/n, Badalona, 8916, Spain.

(37)Assaf Harofeh Medical Center, Zerifin, Beer-Yaakov, 70100, Israel.

(38)Hospital Italiano, Guise 1870, Buenos Aires, 1425, Argentina.

(39)Jahn Ferenc Teaching Hospital, Köves u. 1., Budapest, 1101, Hungary.

(40)Jewish General Hospital, 3755 Cote-Sainte-Catherine, Montreal, J7A 4T8,

Canada.

(41)Hospital de Galdakao-Usansolo, Barrio Labeaga s.n., Galdakao, 48660, Spain.

(42)INEBA - Institute of Neuroscience Buenos Aires, Guardia Vieja 4435, Buenos

Aires, C1192AAW, Argentina.

(43)Brain and Mind Centre, University of Sydney, 100 Mallett, Camperdown, 2050,

Australia.

(44)Department of Neurology, Box Hill Hospital, Monash University, Melbourne,

Australia.

Timely initiation of effective therapy is crucial for preventing disability in

multiple sclerosis; however, treatment response varies greatly among patients.

Comprehensive predictive models of individual treatment response are lacking. Our

aims were: (i) to develop predictive algorithms for individual treatment response

using demographic, clinical and paraclinical predictors in patients with multiple

sclerosis; and (ii) to evaluate accuracy, and internal and external validity of

these algorithms. This study evaluated 27 demographic, clinical and paraclinical

predictors of individual response to seven disease-modifying therapies in MSBase,

a large global cohort study. Treatment response was analysed separately for

disability progression, disability regression, relapse frequency, conversion to

secondary progressive disease, change in the cumulative disease burden, and the

probability of treatment discontinuation. Multivariable survival and generalized

linear models were used, together with the principal component analysis to reduce

model dimensionality and prevent overparameterization. Accuracy of the individual

prediction was tested and its internal validity was evaluated in a separate,

non-overlapping cohort. External validity was evaluated in a geographically

distinct cohort, the Swedish Multiple Sclerosis Registry. In the training cohort

(n = 8513), the most prominent modifiers of treatment response comprised age,

disease duration, disease course, previous relapse activity, disability,

predominant relapse phenotype and previous therapy. Importantly, the magnitude

and direction of the associations varied among therapies and disease outcomes.

Higher probability of disability progression during treatment with injectable

therapies was predominantly associated with a greater disability at treatment

start and the previous therapy. For fingolimod, natalizumab or mitoxantrone, it

was mainly associated with lower pretreatment relapse activity. The probability

of disability regression was predominantly associated with pre-baseline

disability, therapy and relapse activity. Relapse incidence was associated with

pretreatment relapse activity, age and relapsing disease course, with the

strength of these associations varying among therapies. Accuracy and internal

validity (n = 1196) of the resulting predictive models was high (>80%) for

relapse incidence during the first year and for disability outcomes, moderate for

relapse incidence in Years 2-4 and for the change in the cumulative disease

burden, and low for conversion to secondary progressive disease and treatment

discontinuation. External validation showed similar results, demonstrating high

external validity for disability and relapse outcomes, moderate external validity

for cumulative disease burden and low external validity for conversion to

secondary progressive disease and treatment discontinuation. We conclude that

demographic, clinical and paraclinical information helps predict individual

response to disease-modifying therapies at the time of their commencement.

© The Author (2017). Published by Oxford University Press on behalf of the

Guarantors of Brain. All rights reserved. For Permissions, please email:

journals.permissions@oup.com.

DOI: 10.1093/brain/awx185

PMID: 29050389 [Indexed for MEDLINE]

41. Mult Scler. 2017 Sep 1:1352458517731913. doi: 10.1177/1352458517731913. [Epub

ahead of print]

Excluded b/c no real-world adherence/persistence

Recurrence of disease activity during pregnancy after cessation of fingolimod in

multiple sclerosis.

Meinl I(1), Havla J(1), Hohlfeld R(2), Kümpfel T(1).

Author information:

(1)Institute of Clinical Neuroimmunology, Biomedical Center and University

Hospital, Ludwig-Maximilians-Universität München, Munich, Germany.

(2)Institute of Clinical Neuroimmunology, Biomedical Center and University

Hospital, Ludwig-Maximilians-Universität München, Munich, Germany/The Munich

Cluster for Systems Neurology (SyNergy), Munich, Germany.

BACKGROUND: Fingolimod is an effective treatment for active relapsing-remitting

multiple sclerosis (MS). Discontinuation of therapy may be followed by recurrence

of disease activity. Thus, female MS patients may be at risk of relapse during

pregnancy after stopping fingolimod.

OBJECTIVES AND METHODS: To report the disease course during pregnancy of five

women who interrupted therapy with fingolimod for pregnancy.

RESULTS: All patients experienced relapses during pregnancy and/or postpartum

after stopping fingolimod.

CONCLUSION: The risk of recurrence of disease activity during pregnancy after

stopping fingolimod may be substantial. This should be considered and discussed

with MS patients who are planning to become pregnant.

DOI: 10.1177/1352458517731913

PMID: 28920764

Excluded b/c pediatric patients

42. Qual Life Res. 2017 Sep;26(9):2333-2349. doi: 10.1007/s11136-017-1571-z. Epub

2017 Apr 9.

Impact of an electronic monitoring device and behavioral feedback on adherence to

multiple sclerosis therapies in youth: results of a randomized trial.

Yeh EA(1)(2), Grover SA(3), Powell VE(4), Alper G(5), Banwell BL(6), Edwards

K(7), Gorman M(8), Graves J(9), Lotze TE(10), Mah JK(11), Mednick L(8), Ness

J(12), Obadia M(13)(14), Slater R(7), Waldman A(6), Waubant E(9), Schwartz

CE(4)(15); Pediatric MS Adherence Study Group.

Collaborators: Aaen G, Alper G, Banwell BL, Belsole C, Berenbaum T, Breiner P,

Camposano S, Chohan H, Darrell C, Dowdy S, Edwards K, Gorman M, Graves J, Grayson

J, Grover SA, Haig T, Hamer S, Hart J, Jenkins K, Lavery A, Liu G, Lotze T, Mah

JK, Mahabir R, Mar S, Mednick L, Mendoza ER, Moodley M, Ness J, Noguera A, Obadia

M, Petty M, Pope SP, Pohl D, Pontifes M, Powell VE, Quon E, Rensel M, Resto J,

Rossman I, Rundquist M, Sanchez K, Schreiner T, Schwartz CE, Slater R, Smith M,

Sorum J, Stein A, Stosic M, Tillema JM, Venkateswaran S, Vincent J, Waldman A,

Waubant E, Yeh EA.

Author information:

(1)Pediatric MS and Neuroinflammatory Disorders Program, Division of Neurology,

Department of Pediatrics, Neuroscience and Mental Health, Hospital for Sick

Children Research Institute, Hospital for Sick Children, 555 University Avenue,

Rm 6D33, Toronto, ON, M5G1X8, Canada. ann.yeh@sickkids.ca.

(2)Faculty of Medicine, The University of Toronto, 1 King's College Circle #3172,

Toronto, ON, M5S 1A8, Canada. ann.yeh@sickkids.ca.

(3)Pediatric MS and Neuroinflammatory Disorders Program, Division of Neurology,

Department of Pediatrics, Neuroscience and Mental Health, Hospital for Sick

Children Research Institute, Hospital for Sick Children, 555 University Avenue,

Rm 6D33, Toronto, ON, M5G1X8, Canada.

(4)DeltaQuest Foundation Inc., 31 Mitchell Road, Concord, MA, 01742, USA.

(5)Children's Hospital of Pittsburgh, University of Pittsburgh School of

Medicine, 4401 Penn Avenue, Pittsburgh, PA, 15224, USA.

(6)Division of Neurology, Children's Hospital of Philadelphia, 3401 Civic Center

Blvd., Philadelphia, PA, 19104, USA.

(7)Department of Psychiatry, The Hospital for Sick Children, 555 University

Avenue, Toronto, ON, M5G1X8, Canada.

(8)Boston Children's Hospital, Harvard Medical School, 300 Longwood Avenue,

Boston, MA, 02115, USA.

(9)University of California San Francisco, 505 Parnassus Avenue, San Francisco,

CA, 94143, USA.

(10)Texas Children's Hospital, Baylor College of Medicine, 6621 Fannin Street,

Houston, TX, 77030, USA.

(11)Alberta Children's Hospital, 2888 Shanganappi Trail NW, Calgary, AB, T3B 6A8,

Canada.

(12)University of Alabama at Birmingham, 1720 2nd Avenue, Birmingham, AL, 35294,

USA.

(13)ELLICSR: Health, Wellness, and Cancer Survivorship Centre, University Health

Network, 585 University Avenue, Toronto, ON, M5G 2C4, Canada.

(14)Department of Psychology, Faculty of Medicine, University of Toronto, 1

King's College Circle #3172, Toronto, ON, M5S 1A8, Canada.

(15)Departments of Medicine and Orthopaedic Surgery, Tufts University Medical

School, 800 Washington Street, Boston, MA, 02111, USA.

Erratum in

Qual Life Res. 2017 Dec 23;:.

PURPOSE: To report the results of a randomized controlled trial using an

electronic monitoring device (EM) plus a motivational interviewing (MI)

intervention to enhance adherence to disease-modifying therapies (DMT) in

pediatric MS.

METHODS: Fifty-two youth with MS (16.03 ± 2.2 years) were randomized to receive

either MI (n = 25) (target intervention) or a MS medication video (n = 27)

(attention control). Primary endpoint was change in adherence. Secondary outcomes

included changes in quality of life, well-being and self-efficacy. Random effects

modeling and Cohen's effect size computation evaluated intervention impact.

RESULTS: Longitudinal random effect models revealed that the MI group decreased

their EM adherence (GroupxTime interaction = -0.19), while increasing frequency

of parental DMT reminder (26.01)/administration (11.69). We found decreased EM

use in the MI group at 6 months (Cohen's d = -0.61), but increased pharmacy

refill adherence (d = 0.23). Parental reminders about medication increased in MI

subjects vs controls (d = 0.59 at 3 months; d = 0.70 at 6 months). We found

increases in self-reported adherence (d = 0.21) at 3 but not 6 months, fewer

barriers to adherence at three (d = -0.58) and six months (d = -0.31), better

physical (d = 0.23 at 3 months; d = 0.45 at 6 months), emotional (d = 0.25 at

3 months) and self-efficacy function (d = 0.55 at 3 months; 0.48 at 6 months),

but worse well-being, including self-acceptance (d = -0.53 at 6 months) and

environmental mastery (d = -0.42 at 3 and 6 months) in intervention as compared

to control patients.

CONCLUSIONS: Participants receiving MI + EM experienced worsening on objective

measures of adherence and increased parental involvement, but improved on some

self- and parent-reported measures. MI participants reported improvements in

quality of life and self-efficacy, but worsened well-being.

DOI: 10.1007/s11136-017-1571-z

PMID: 28393317

43. Brain Behav. 2017 Aug 29;7(10):e00804. doi: 10.1002/brb3.804. eCollection 2017

Oct.

Excluded b/c no real-world adherence/persistence

Gender differences in safety issues during Fingolimod therapy: Evidence from a

real-life Relapsing Multiple Sclerosis cohort.

Manni A(1), Direnzo V(1), Iaffaldano A(1), Di Lecce V(1), Tortorella C(1),

Zoccolella S(1), Iaffaldano P(1), Trojano M(1), Paolicelli D(1).

Author information:

(1)Department of Basic Medical Sciences, Neuroscience and Sense OrgansUniversity

of Bari" Aldo Moro"BariItaly.

OBJECTIVE: Benefits and risks of new therapies in Multiple Sclerosis (MS) must be

balanced carefully and tailored to patients. We aimed to describe our experience

with Fingolimod (FTY), correlating demographics, clinical and hematological

features of the Relapsing MS (RMS) cohort with the occurring Adverse Events

(AEs).

MATERIAL AND METHODS: Pretreatment screening tests, cardiological observation,

and safety follow-up data were analyzed in 225 RMS patients. Changes in

continuous data were analyzed post hoc with Wilcoxon ranks test; categorical

variables were examined using McNemar test. Two-way repeated-measures analysis of

variance (ANOVA) was used to analyze differences between baseline characteristic

of the cohorts and Liver Function Tests (LFT) alterations. Binary logistic

regression models were used to identify which of the baseline factors influenced

LFT alterations and the occurrence of infections.

RESULTS: During 2 years of follow-up 24 patients (10%) interrupted FTY.

Discontinuation most often was due to AEs (n = 14) or breakthrough disease

(n = 5). The most frequently AEs were infections (10.6%). After the first year

patients showing an infectious episode were mostly female (p = .04). The

infections did not correlate with the decrease in white blood cells or to

lymphocyte count. AST and ALT alterations ​​were observed mostly in males

(p = .002 and p = .01, respectively), and increase in GGT ​​was reported in

subjects older at FTY beginning (p < .05).

CONCLUSIONS: For a patient-centered safety monitoring of FTY, we may apply

gender-specific warnings, for the detection of transaminases abnormalities and

infectious episodes.

DOI: 10.1002/brb3.804

PMCID: PMC5651388

PMID: 29075564

44. Mult Scler J Exp Transl Clin. 2017 Aug 24;3(3):2055217317715485. doi:

10.1177/2055217317715485. eCollection 2017 Jul-Sep.

Comparative efficacy and discontinuation of dimethyl fumarate and fingolimod in

clinical practice at 24-month follow-up.

Hersh CM(1), Love TE(2), Bandyopadhyay A(3), Cohn S(4), Hara-Cleaver C(5), Bermel

RA(5), Fox RJ(5), Cohen JA(5), Ontaneda D(5).

Author information:

(1)Lou Ruvo Center for Brain Health, Cleveland Clinic, USA.

(2)Departments of Medicine and Epidemiology and Biostatistics, Case Western

Reserve University, USA.

(3)Emory University, USA.

(4)Department of Neurology, Cleveland Clinic, USA.

(5)Mellen Center for Multiple Sclerosis Treatment and Research, Cleveland Clinic,

USA.

BACKGROUND: Dimethyl fumarate and fingolimod are oral disease-modifying therapies

approved to treat relapsing multiple sclerosis. Prior observational studies and

our previous 12-month investigation showed comparable clinical efficacy.

OBJECTIVE: The purpose of this study was to assess real-world efficacy and

discontinuation of dimethyl fumarate and fingolimod over 24 months in patients

with multiple sclerosis.

METHODS: Patients treated with dimethyl fumarate (n = 395) or fingolimod

(n = 264) completed 24-month follow-up in a large academic multiple sclerosis

center. Discontinuation rates and measures of disease activity were compared

after propensity score weighting. The primary outcome was on-treatment annualized

relapse rate ratio. Other measures included rate of drug discontinuation and

brain magnetic resonance imaging activity defined as new T2 and/or

gadolinium-enhancing lesions.

RESULTS: Propensity score weighting showed excellent covariate balance. At 24

months, dimethyl fumarate demonstrated comparable annualized relapse rate (rate

ratio = 1.45, 95% confidence interval 0.53-3.99) and brain magnetic resonance

imaging activity (odds ratio = 1.38, 95% confidence interval 0.83-2.32). Dimethyl

fumarate patients discontinued therapy earlier compared to fingolimod (hazard

ratio = 1.40, 95% confidence interval 1.11-1.77) and were more likely to

discontinue therapy due to intolerability (odds ratio = 1.98, 95% confidence

interval 1.18-3.23).

CONCLUSION: Dimethyl fumarate and fingolimod had similar reductions in annualized

relapse rate in clinical trials, and our real-world experience supports this

observation. Dimethyl fumarate-treated patients had higher likelihood of early

discontinuation, and this was mostly due to intolerability.

DOI: 10.1177/2055217317715485

PMCID: PMC5574489

PMID: 28890796

45. Mult Scler J Exp Transl Clin. 2017 Aug 17;3(3):2055217317725102. doi:

10.1177/2055217317725102. eCollection 2017 Jul-Sep.

Comparison of fingolimod and dimethyl fumarate in the treatment of multiple

sclerosis: Two-year experience.

Vollmer B(1), Nair KV(2), Sillau SH(1), Corboy J(1), Vollmer T(1), Alvarez E(1).

Author information:

(1)Rocky Mountain MS Center at University of Colorado, USA.

(2)Skaggs School of Pharmacy and Pharmaceutical Sciences, University of Colorado

Denver, United States.

BACKGROUND: Fingolimod (FTY) and dimethyl fumarate (DMF) are multiple sclerosis

(MS) oral therapies that became available in 2010 and 2013, respectively.

OBJECTIVE: The objective of this article is to compare discontinuation rates,

efficacy, and adverse events (AEs) of FTY and DMF over two years.

METHODS: Patients prescribed FTY or DMF at the Rocky Mountain MS Center at

University of Colorado prior to October 2013 were identified. Clinician-reported

data were retrospectively collected. Primary outcome was discontinuation of drug

by the end of year two. Reasons for discontinuation were evaluated.

RESULTS: A total of 271 FTY and 342 DMF patients were evaluated. Patients had a

mean age of 42.5 (FTY) and 45.8 (DMF) years and were predominantly female (72.0%

FTY; 69.6% DMF) and white (86.3% FTY; 82.2% DMF). At ≤24 months, 93 (34.3%) and

161 (47.1%) discontinued FTY and DMF, respectively, with an unadjusted odds ratio

(OR) of 1.70 (1.23-2.37, p = 0.002), or 1.69 (1.16-2.46, p = 0.006) for the

doubly robust propensity score weighted estimator. Primary reason for

discontinuation was AEs, which were less likely for FTY 46 (17.0%) compared to

DMF 82 (24.0%) (OR 1.54, 1.03-2.31, p = 0.035). Discontinuation due to disease

activity (FTY (10%) DMF (11.1%); OR 1.13, 0.67-1.90, p = 0.647) and breakthrough

disease activity, regardless of discontinuation (FTY (34.7%) DMF (33.6%); OR

0.95, 0.68-1.34, p = 0.783), were similar.

CONCLUSIONS: The odds of discontinuation were less for FTY than DMF, and were

driven by AEs for both drugs.

DOI: 10.1177/2055217317725102

PMCID: PMC5564884

PMID: 28839949

46. BMC Neurol. 2017 Aug 9;17(1):156. doi: 10.1186/s12883-017-0928-9.

Excluded b/c no real-world adherence/persistence

Patient satisfaction with ExtaviPro™ 30G, a new auto-injector for administering

interferon β-1b in multiple sclerosis: results from a real-world, observational

EXCHANGE study.

Hoffmann FA(1), Trenova A(2), Llaneza MA(3), Fischer J(4), Lus G(5), von Bredow

D(6), Lara N(7), Lam E(8), Van Hoef M(9), Bakshi R(10).

Author information:

(1)Department of Neurology, Hospital Martha-Maria Halle-Dölau, Halle, Germany.

(2)Department of Neurology, Medical University of Plovdiv, Plovdiv, Bulgaria.

(3)Neurology Department, Ferrol University Hospital, Ferrol, Spain.

(4)Neurologische Praxis (NTDStudy-Group), Lappersdorf, Germany.

(5)Multiple Sclerosi Center university of Campania L. Vanvitelli, Naples, Italy.

(6)QuintilesIMS, IMS Health GmbH & Co. OHG, Munich, Germany.

(7)QuintilesIMS, Barcelona, Spain.

(8)Novartis Pharmaceuticals Corporation, East Hanover, NJ, USA.

(9)Novartis Pharma AG, Fabrikstrasse 12-3.03.12, Postfach, CH-4002, Basel,

Switzerland.

(10)Novartis Pharma AG, Fabrikstrasse 12-3.03.12, Postfach, CH-4002, Basel,

Switzerland. rajesh.bakshi@novartis.com.

BACKGROUND: Patients with multiple sclerosis (MS) receiving long-term,

subcutaneous interferon β-1b (IFN β-1b; Extavia®) often experience injection-site

reactions and injection-site pain, which together with other side-effects (such

as flu-like symptoms) result in suboptimal treatment compliance/adherence. The

EXCHANGE study evaluated patient satisfaction with IFN β-1b treatment,

administered using ExtaviPro™ 30G, a new auto-injector, in a real-world setting.

METHODS: This 26-week, open-label, prospective, non-interventional,

observational, multi-country multi-centre study enrolled patients with MS who had

been treated with IFN β-1b or other disease-modifying therapies with a

self-administered auto-injector for ≥3 months and who were planned to switch to

IFN β-1b treatment administered using ExtaviPro™ 30G as part of routine clinical

care. Patient-reported outcomes included overall patient satisfaction (primary

outcome) and satisfaction associated with treatment effectiveness, convenience

and side-effects, assessed using Treatment Satisfaction Questionnaire for

Medication (TSQM)-14. The changes in TSQM scores from baseline to Week 26 were

reported. All data were analysed using SAS statistical software (version 9.4).

RESULTS: Of the 336 patients enrolled, 324 were included in the analysis. At

baseline, mean ± standard deviation (SD) age of patients was 41.8 ± 11.3 years

and 68.2% were women. The mean ± SD of MS disease duration was 6.9 ± 6.6 years,

and the majority of patients (94.1%) had relapsing-remitting MS. The mean ± SD of

TSQM score for overall patient satisfaction at Week 26 was 75.6 ± 16.46

(baseline, 73.0 ± 17.14; p = 0.0342). The mean ± SD of TSQM subscale scores for

patient satisfaction with effectiveness, side-effects and convenience were

75.0 ± 18.65 (baseline, 71.6 ± 19.45; p = 0.0356), 88.5 ± 18.98 (baseline,

82.7 ± 22.93; p = 0.0002) and 77.6 ± 16.72 (baseline, 71.1 ± 17.53; p < 0.0001),

respectively.

CONCLUSION: The results from this real-world study suggest that administering IFN

β-1b with the new ExtaviPro™ auto-injector significantly improves overall patient

satisfaction, including satisfaction associated with effectiveness, side-effects

and convenience in MS patients.

DOI: 10.1186/s12883-017-0928-9

PMCID: PMC5549369

PMID: 28793876 [Indexed for MEDLINE]

47. Acta Neurol Scand. 2017 Aug;136(2):116-121. doi: 10.1111/ane.12705. Epub 2016 Oct

Excluded b/c no oral DMD results

30.

Baseline predictors of persistence to first disease-modifying treatment in

multiple sclerosis.

Zettl UK(1), Schreiber H(2), Bauer-Steinhusen U(3), Glaser T(3), Hechenbichler

K(4), Hecker M(1); BETAPATH Study Group.

Author information:

(1)Department of Neurology, Neuroimmunology Section, University of Rostock,

Rostock, Germany.

(2)Neurological Practice Center, Ulm, Germany.

(3)Neurology, Immunology, and Ophthalmology, Bayer Vital GmbH, Leverkusen,

Germany.

(4)Institute Dr. Schauerte, München, Germany.

OBJECTIVES: Patients with multiple sclerosis (MS) require lifelong therapy.

However, success of disease-modifying therapies is dependent on patients'

persistence and adherence to treatment schedules. In the setting of a large

multicenter observational study, we aimed at assessing multiple parameters for

their predictive power with respect to discontinuation of therapy.

MATERIALS AND METHODS: We analyzed 13 parameters to predict discontinuation of

interferon beta-1b treatment during a 2-year follow-up period based on data from

395 patients with MS who were treatment-naïve at study onset. Besides clinical

characteristics, patient-related psychosocial outcomes were assessed as well.

RESULTS: Among patients without clinically relevant fatigue, males showed a

higher persistence rate than females (80.3% vs 64.7%). Clinically relevant

fatigue scores decreased the persistence rate in men and especially in women

(71.4% and 51.2%). Besides gender and fatigue, univariable and multivariable

analyses revealed further factors associated with interferon beta-1b therapy

discontinuation, namely lower quality of life, depressiveness, and higher relapse

rate before therapy initiation, while higher education, living without a partner,

and higher age improved persistence.

CONCLUSIONS: Patients with higher grades of fatigue and depressiveness are at

higher risk to prematurely discontinue MS treatment; especially, women suffering

from fatigue have an increased discontinuation rate.

© 2016 John Wiley & Sons A/S. Published by John Wiley & Sons Ltd.

DOI: 10.1111/ane.12705

PMID: 27796033 [Indexed for MEDLINE]

48. Autoimmun Rev. 2017 Aug;16(8):845-855. doi: 10.1016/j.autrev.2017.05.016. Epub

Excluded b/c no primary data (narrative review)

2017 May 28.

Survivin in autoimmune diseases.

Gravina G(1), Wasén C(2), Garcia-Bonete MJ(3), Turkkila M(4), Erlandsson MC(5),

Töyrä Silfverswärd S(6), Brisslert M(7), Pullerits R(8), Andersson KM(9), Katona

G(10), Bokarewa MI(11).

Author information:

(1)Department of Rheumatology and Inflammation Research, Institute of Medicine,

Sahlgrenska Academy, University of Gothenburg, Gothenburg, Sweden. Electronic

address: giacomo2392@gmail.com.

(2)Department of Rheumatology and Inflammation Research, Institute of Medicine,

Sahlgrenska Academy, University of Gothenburg, Gothenburg, Sweden. Electronic

address: caroline.wasen@rheuma.gu.se.

(3)Department of Chemistry and Molecular Biology, University of Gothenburg,

Gothenburg, Sweden. Electronic address: maria-jose.garcia.bonete@gu.se.

(4)Department of Rheumatology and Inflammation Research, Institute of Medicine,

Sahlgrenska Academy, University of Gothenburg, Gothenburg, Sweden. Electronic

address: minnaturkkila@outlook.com.

(5)Department of Rheumatology and Inflammation Research, Institute of Medicine,

Sahlgrenska Academy, University of Gothenburg, Gothenburg, Sweden; Rheumatology

Clinic, Sahlgrenska University Hospital, Gothenburg, Sweden. Electronic address:

malin.erlandsson@rheuma.gu.se.

(6)Department of Rheumatology and Inflammation Research, Institute of Medicine,

Sahlgrenska Academy, University of Gothenburg, Gothenburg, Sweden. Electronic

address: sofia.silfversward@rheuma.gu.se.

(7)Department of Rheumatology and Inflammation Research, Institute of Medicine,

Sahlgrenska Academy, University of Gothenburg, Gothenburg, Sweden. Electronic

address: mikael.brisslert@gu.se.

(8)Department of Rheumatology and Inflammation Research, Institute of Medicine,

Sahlgrenska Academy, University of Gothenburg, Gothenburg, Sweden; Department of

Clinical Immunology and Transfusion Medicine, Sahlgrenska University Hospital,

Gothenburg, Sweden. Electronic address: rille.pullerits@rheuma.gu.se.

(9)Department of Rheumatology and Inflammation Research, Institute of Medicine,

Sahlgrenska Academy, University of Gothenburg, Gothenburg, Sweden. Electronic

address: karin.andersson@rheuma.gu.se.

(10)Department of Chemistry and Molecular Biology, University of Gothenburg,

Gothenburg, Sweden. Electronic address: gergely.katona@gu.se.

(11)Department of Rheumatology and Inflammation Research, Institute of Medicine,

Sahlgrenska Academy, University of Gothenburg, Gothenburg, Sweden; Rheumatology

Clinic, Sahlgrenska University Hospital, Gothenburg, Sweden. Electronic address:

maria.bokarewa@rheuma.gu.se.

Survivin is a protein functionally important for cell division, apoptosis, and

possibly, for micro-RNA biogenesis. It is an established marker of malignant cell

transformation. In non-malignant conditions, the unique properties of survivin

make it indispensable for homeostasis of the immune system. Indeed, it is

required for the innate and adaptive immune responses, controlling

differentiation and maintenance of CD4+ and CD8+ memory T-cells, and in B cell

maturation. Recently, survivin has emerged as an important player in the

pathogenesis of autoimmune diseases. Under the conditions of unreserved

inflammation, survivin enhances antigen presentation, maintains persistence of

autoreactive cells, and supports production of autoantibodies. In this context,

survivin takes its place as a diagnostic and prognostic marker in rheumatoid

arthritis, psoriasis, systemic sclerosis and pulmonary arterial hypertension,

neuropathology and multiple sclerosis, inflammatory bowel diseases and oral

lichen planus. In this review, we summarise the knowledge about non-malignant

properties of survivin and focus on its engagement in cellular and molecular

pathology of autoimmune diseases. The review highlights utility of survivin

measures for clinical applications. It provides rational for the survivin

inhibiting strategies and presents results of recent reports on survivin

inhibition in modern therapies of cancers and autoimmune diseases.

Copyright © 2017 Elsevier B.V. All rights reserved.

DOI: 10.1016/j.autrev.2017.05.016

PMID: 28564620 [Indexed for MEDLINE]

Excluded b/c no real-world adherence/persistence

49. J Manag Care Spec Pharm. 2017 Aug;23(8):822-830. doi:

10.18553/jmcp.2017.23.8.822.

Factors Associated with Patient Preferences for Disease-Modifying Therapies in

Multiple Sclerosis.

Hincapie AL(1), Penm J(1), Burns CF(2).

Author information:

(1)1 James L. Winkle College of Pharmacy, University of Cincinnati, Cincinnati,

Ohio.

(2)2 College of Pharmacy, University of Oklahoma, Tulsa.

BACKGROUND: Treatment adherence in patients with multiple sclerosis (MS) is

essential to reduce the rate of acute neurological attacks, severity of relapses,

and hospitalizations and to slow its progression. Adherence rates in MS patients

have been shown to be affected by multiple factors, including physical or

cognitive difficulties, perceived lack of treatment efficacy, treatment-related

adverse events, injection anxiety, and frequency of administration.

OBJECTIVE: To elicit the preferences of MS patients for noneconomic and economic

attributes of current disease-modifying therapies (DMTs).

METHODS: We used conjoint analysis to estimate preferences from a convenience

sample through a web-based online survey. Patients were invited to participate in

the study using web portals and newsletters for MS patients. The conjoint survey

included the following 6 attributes: (1) overall efficacy based on autoimmune

disease progression stabilization; (2) acute increase in disease activity

(flare-up); (3) rate of respiratory tract infections; (4) rate of serious

respiratory tract infections (leading to hospitalization); (5) medication use;

and (6) patient monthly out-of-pocket medication costs. Using a fractional

factorial design, 24 product profiles were created. Each respondent reviewed a

random selection of 8 profiles. With each profile, subjects were asked to

indicate their likelihood to try the hypothetical products on a scale from 0 to

100. Random effects linear regression was used to elicit preferences.

RESULTS: After exclusion of respondents with incomplete information, data from

129 subjects were included in the analysis. The overall relative importance of

each attribute for the ranges presented were (1) 38.4% for monthly out-of-pocket

cost; (2) 21.5% for route and frequency of administration; (3) 15.9% for risk of

hospitalization by infection; (4) 11.9% for risk of respiratory tract infection;

(5) 7.4% for risk of flare-ups; and (6) 5.0% for disease progression

stabilization. Preference weights indicated that subjects favored subcutaneous

(beta coefficient [β] = -2.26, 95% CI = -4.22 to -0.22) and oral administration

(β = 7.93, 95% CI = 5.95 to 10.2) over intramuscular (β = -5.67, 95% CI = -8.67

to -3.56), but no significant differences were found between subcutaneous over

intramuscular administration. Monthly out-of-pocket cost was the most influential

attribute, with an overall relative importance of 38%. The most preferred level

was $75 (β = 12.85, 95% CI = 10.64 to 15.06) followed by $150 (β = 3.41, 95% CI =

0.98 to 5.84) when compared between $75, $150, $300, and $450 a month.

CONCLUSIONS: Conjoint analysis proved to be a convenient tool to quantify

respondents' relative preferences for DMT characteristics. Respondents gave

higher weight to DMT monthly out-of-pocket costs and mode of administration than

to adverse effects or efficacy. These findings may assist in the development of

DMT cost-sharing strategies and shared decision making at the point of care.

DISCLOSURES: No outside funding supported this study. The authors declare no

potential conflicts of interest. Study concept and design were contributed by

Hincapie and Burns. Data were collected by Hincapie and Burns, and interpreted by

all the authors. The manuscript was written by Hincapie, Penm, and Burns and

revised by Penm, Hincapie, and Burns. At the time of data collection, Burns was a

PhD candidate at The University of Oklahoma, College of Pharmacy.

DOI: 10.18553/jmcp.2017.23.8.822

PMID: 28737987

50. J Manag Care Spec Pharm. 2017 Aug;23(8):844-852. doi:

10.18553/jmcp.2017.23.8.844.

Real-World Adherence and Persistence to Oral Disease-Modifying Therapies in

Multiple Sclerosis Patients Over 1 Year.

Johnson KM(1), Zhou H(2), Lin F(1), Ko JJ(1), Herrera V(1).

Author information:

(1)1 Novartis Pharmaceuticals, East Hanover, New Jersey.

(2)2 Consulting, Morristown, New Jersey.

BACKGROUND: Disease-modifying therapies (DMTs) are indicated to reduce relapse

rates and slow disease progression for relapsing-remitting multiple sclerosis

(MS) patients when taken as prescribed. Nonadherence or non-persistence in the

real-world setting can lead to greater risk for negative clinical outcomes.

Although previous research has demonstrated greater adherence and persistence to

oral DMTs compared with injectable DMTs, comparisons among oral DMTs are lacking.

OBJECTIVE: To compare adherence, persistence, and time to discontinuation among

MS patients newly prescribed the oral DMTs fingolimod, dimethyl fumarate, or

teriflunomide.

METHODS: This retrospective study used MarketScan Commercial and Medicare

Supplemental claims databases. MS patients with ≥ 1 claim for specified DMTs from

April 1, 2013, to June 30, 2013, were identified. The index drug was defined as

the first oral DMT within this period. To capture patients newly initiating index

DMTs, patients could not have a claim for their index drugs in the previous 12

months. Baseline characteristics were described for patients in each treatment

cohort. Adherence, as measured by medication possession ratio (MPR) and

proportion of days covered (PDC); persistence (30-day gap allowed); and time to

discontinuation over a 12-month follow-up period were compared across treatment

cohorts. Adjusted logistic regression models were used to examine adherence, and

Cox regression models estimated risk of discontinuation.

RESULTS: 1,498 patients newly initiated oral DMTs and met study inclusion

criteria: fingolimod (n = 185), dimethyl fumarate (n = 1,160), and teriflunomide

(n = 143). Patients were similar across most baseline characteristics, including

region, relapse history, and health care resource utilization. Statistically

significant differences were observed across the treatment cohorts for age,

gender, previous injectable/infused DMT use, and comorbidities. Adherence and

time to discontinuation were adjusted for age, gender, region, previous oral and

injectable/infused DMT use, relapse history, and Charlson Comorbidity Index

score. Relative to fingolimod patients, dimethyl fumarate and teriflunomide

patients were significantly less likely to have an MPR ≥ 80% (OR = 0.18; 95% CI =

0.09-0.36; P < 0.001 and OR = 0.19; 95% CI = 0.08-0.42; P < 0.001, respectively).

Similarly, relative to fingolimod patients, dimethyl fumarate and teriflunomide

patients were significantly less likely to have PDC ≥ 80% (OR = 0.47; 95% CI =

0.33-0.67; P < 0.001 and OR = 0.37; 95% CI = 0.23-0.59; P < 0.001, respectively).

Additionally, the HR for discontinuation was about 2 times greater for dimethyl

fumarate (HR = 1.93; 95% CI = 1.44-2.59; P < 0.001) and teriflunomide patients

(HR = 2.27; 95% CI = 1.57-3.28; P < 0.001) compared with fingolimod.

CONCLUSIONS: In a real-world setting, patients taking fingolimod had better

adherence and persistence compared with patients taking other oral DMTs over 12

months. Coupled with clinical factors, medication adherence and persistence

should be important considerations when determining coverage decisions for MS

patients.

DISCLOSURES: This research was funded by Novartis Pharmaceuticals. Johnson, Lin,

Ko, and Herrera are employed by Novartis Pharmaceuticals and own Novartis stock.

Huanxue Zhou is employed by KMK Consulting, which provides consulting services to

Novartis. Study concept and design were contributed by Johnson, Lin, Ko, and

Herrera. Zhou collected the data, and data interpretation was performed by

Johnson, Lin, Ko, and Herrera. All authors were involved in manuscript revision.

The abstract for this study was presented at the AMCP Nexus 2015; October 26-29,

2015; Orlando, Florida.

DOI: 10.18553/jmcp.2017.23.8.844

PMID: 28737986

51. Mult Scler. 2017 Aug;23(9):1249-1257. doi: 10.1177/1352458516676643. Epub 2016

Oct 25.

Excluded b/c no real-world adherence/persistence

Improved treatment satisfaction after switching therapy to rituximab in

relapsing-remitting MS.

de Flon P(1), Laurell K(2), Söderström L(3), Gunnarsson M(4), Svenningsson A(5).

Author information:

(1)Department of Neurology, Östersund Hospital, Östersund, Sweden/Neurology Unit,

Department of Pharmacology and Clinical Neuroscience, Umeå University, Östersund,

Sweden.

(2)Neurology Unit, Department of Pharmacology and Clinical Neuroscience, Umeå

University, Östersund, Sweden.

(3)Unit of Research, Education and Development, Östersund Hospital, Region

Jämtland Härjedalen, Östersund, Sweden.

(4)Department of Neurology, School of Medical Sciences, Örebro University,

Örebro, Sweden.

(5)Department of Pharmacology and Clinical Neuroscience, Umeå University, Umeå,

Sweden/Department of Clinical Sciences, Danderyd Hospital, Karolinska Institutet,

Stockholm, Sweden.

OBJECTIVE: New disease-modifying treatment strategies in multiple sclerosis offer

possibilities for individualised treatment. In this study, we evaluated

patient-reported outcome measures before and after a switch in therapy from

first-line injectable treatments to rituximab.

METHOD: A total of 75 patients with clinically stable relapsing-remitting

multiple sclerosis (RRMS) receiving ongoing first-line injectable treatment at

three Swedish centres had their treatment switched to rituximab in this

open-label phase II multicentre study. Assessment of treatment satisfaction,

patient-perceived impact of the disease on daily life, fatigue, cognitive

symptoms and disease progression was performed 3 months before and at the time of

the treatment shift and then for a subsequent 2-year period.

RESULTS: The overall treatment satisfaction rating improved significantly from a

mean of 4.8 (scale range: 1-7), while on injectable therapies, to a mean of 6.3

after 1 year of rituximab treatment ( p < 0.001). This improvement was sustained

after 2 years. There was no significant change in scores for patient-perceived

impact of disease, fatigue or disease progression.

CONCLUSION: A shift in therapy from first-line injectables to rituximab in a

cohort of clinically stable RRMS patients was followed by improved treatment

satisfaction. This is clinically relevant as it may influence long-term adherence

to immunomodulating therapy.

DOI: 10.1177/1352458516676643

PMID: 27780912

52. Mult Scler. 2017 Aug;23(9):1241-1248. doi: 10.1177/1352458516675751. Epub 2016

Oct 20.

Excluded b/c no real-world adherence/persistence

Discontinuation of disease-modifying therapies in multiple sclerosis - Clinical

outcome and prognostic factors.

Bsteh G(1), Feige J(1), Ehling R(1), Auer M(1), Hegen H(1), Di Pauli F(1),

Deisenhammer F(1), Reindl M(1), Berger T(1).

Author information:

(1)Clinical Department of Neurology, Medical University of Innsbruck, Innsbruck,

Austria.

BACKGROUND: Stable disease course may prompt consideration of disease-modifying

treatment (DMT) discontinuation in relapsing-remitting multiple sclerosis (RRMS).

OBJECTIVE: To investigate the clinical outcome after DMT discontinuation and to

identify predictive factors supporting decision-making.

METHODS: We included 221 RRMS patients, who discontinued DMT after ⩾12 months and

had documented follow-up ⩾2 years after discontinuation. Hazard ratios (HRs) with

95% confidence intervals (CIs) regarding relapse and disability progression after

DMT discontinuation were calculated from Cox regression models.

RESULTS: Age >45 years at discontinuation (HR = 0.47, CI = 0.23-0.95, p = 0.038),

absence of relapses for ⩾4 years on DMT before discontinuation (HR = 0.29,

CI = 0.10-0.82, p = 0.020) and absence of contrast enhancing lesions (HR = 0.46,

CI = 0.28-0.78, p = 0.004) were independent predictors of absence of relapse

after discontinuation. Age >45 years and absence of relapses ⩾4 years on DMT

combined had an HR of 0.06 (CI = 0.01-0.44, p < 0.001). Higher Expanded

Disability Status Scale (EDSS) at discontinuation, age >45 years and longer

disease duration were significantly associated with disability progression after

discontinuation.

CONCLUSION: While freedom from further disease activity is generally

unpredictable, there is a subset of patients (age ⩾45 years, DMT intake ⩾4 years

without evidence of clinical or radiological disease activity) having a high

likelihood of remaining relapse-free after DMT discontinuation. However, close

clinical monitoring for recurrent disease activity is mandatory after

discontinuing treatment.

DOI: 10.1177/1352458516675751

PMID: 27765877

53. Brain Sci. 2017 Jul 7;7(7). pii: E78. doi: 10.3390/brainsci7070078.

Excluded b/c no primary data (narrative review)

Multiple Sclerosis: Immunopathology and Treatment Update.

Dargahi N(1), Katsara M(2), Tselios T(3), Androutsou ME(4), de Courten M(5),

Matsoukas J(6), Apostolopoulos V(7).

Author information:

(1)Centre for Chronic Disease, College of Health and Biomedicine, Victoria

University, Melbourne VIC 3030, Australia. narges.dargahi@live.vu.edu.au.

(2)Medical Department, Novartis (Hellas) SACI, Metamorphosis, Athens 14452,

Greece. maria.katsara@novartis.com.

(3)Department of Chemistry, University of Patras, Rio, Patras 26500, Greece.

ttselios@upatras.gr.

(4)Vianex S.A., Metamorphosis, Attikis, Athens 14451, Greece.

AndroutsouM@vianex.gr.

(5)Centre for Chronic Disease, College of Health and Biomedicine, Victoria

University, Melbourne VIC 3030, Australia. Maximilian.deCourten@vu.edu.au.

(6)ELDrug S.A., Patras Science Park, Platani, Patras 26504, Greece.

imats1953@gmail.com.

(7)Centre for Chronic Disease, College of Health and Biomedicine, Victoria

University, Melbourne VIC 3030, Australia. vasso.apostolopoulos@vu.edu.au.

The treatment of multiple sclerosis (MS) has changed over the last 20 years. All

immunotherapeutic drugs target relapsing remitting MS (RRMS) and it still remains

a medical challenge in MS to develop a treatment for progressive forms. The most

common injectable disease-modifying therapies in RRMS include β-interferons 1a or

1b and glatiramer acetate. However, one of the major challenges of injectable

disease-modifying therapies has been poor treatment adherence with approximately

50% of patients discontinuing the therapy within the first year. Herein, we go

back to the basics to understand the immunopathophysiology of MS to gain insights

in the development of new improved drug treatments. We present current

disease-modifying therapies (interferons, glatiramer acetate, dimethyl fumarate,

teriflunomide, fingolimod, mitoxantrone), humanized monoclonal antibodies

(natalizumab, ofatumumb, ocrelizumab, alentuzumab, daclizumab) and emerging

immune modulating approaches (stem cells, DNA vaccines, nanoparticles, altered

peptide ligands) for the treatment of MS.

DOI: 10.3390/brainsci7070078

PMCID: PMC5532591

PMID: 28686222

Conflict of interest statement: V.A. is supported by Vianex S.A. Greece in

developing immunotherapeutics against MS; N.D. is supported under VU-Vianex

contract 2 (specific task agreement MS immunotherapeutics) in developing

immunotherapeutics against MS; J.M. is head of the scientific advisory board of

ELDrug a spin off company of Vianex S.A.; M.-E.A. works for Vianex S.A. Greece;

T.T. has an association with Vianex S.A. Greece in relation to supporting his

research; M.K. is an employee of Novartis (Hellas) Greece; M.d.C. declares no

conflicts of interest. The review represents a detailed literature search in the

areas of drugs and treatments against MS with no bias towards immunotherapeutics

developed by Vianex S.A.

54. Patient Prefer Adherence. 2017 Jun 28;11:1093-1101. doi: 10.2147/PPA.S138263.

Excluded b/c no oral DMD results

eCollection 2017.

Persistence to disease-modifying therapies for multiple sclerosis in a Canadian

cohort.

Melesse DY(1)(2), Marrie RA(2)(3), Blanchard JF(1), Yu BN(1)(4), Evans C(5).

Author information:

(1)Centre for Global Public Health.

(2)Department of Community Health Sciences.

(3)Department of Internal Medicine, University of Manitoba.

(4)Public Health, Manitoba Health Seniors and Active Living, Winnipeg, Manitoba.

(5)College of Pharmacy & Nutrition, University of Saskatchewan, Saskatoon,

Saskatchewan, Canada.

PURPOSE: To examine the long-term persistence to the first-line injectable

disease-modifying therapies (DMTs) for multiple sclerosis (MS) and to identify

the factors associated with nonpersistence.

PATIENTS AND METHODS: We used population-based administrative data from Manitoba,

Canada. All adult subjects who were diagnosed with MS and dispensed a first-line

injectable DMT (beta-interferon-1b, beta-interferon-1a, and glatiramer acetate)

between 1996 and 2011 and had a minimum of 1 year of follow-up were included. The

primary outcome was the median time to discontinuation of any DMT. The

associations between potential predictors and persistence were estimated using

multivariable Cox-proportional hazard models.

RESULTS: Overall, 721 subjects were followed for a median of 7.8 years

(interquartile range 6.1). The median time to discontinuation of all first-line

DMTs was 4.2 years (25th and 75th percentile: 1.7, 10.6 years). Of the 451

(62.6%) subjects who discontinued their DMT during the study period, 259 (57.4%)

eventually resumed or restarted a DMT. Subjects who were younger when starting a

DMT, had prior MS-related hospitalizations, were more recently diagnosed with MS,

or had a greater lag time between their MS diagnosis and DMT initiation were more

likely to discontinue therapy.

CONCLUSION: Over half of the individuals receiving a DMT for MS in Manitoba

remained on therapy for at least 4 years. DMT discontinuation occurred in 60% of

the cohort, but most restarted a DMT within 1 year. While not all of the factors

identified with discontinuing DMT are modifiable, they may help practitioners

enhance MS care by identifying individuals who may be at particular risk for DMT

discontinuation.

DOI: 10.2147/PPA.S138263

PMCID: PMC5499788

PMID: 28721023

Conflict of interest statement: Disclosure Dr Marrie received research funding

from Canadian Institutes of Health Research, Research Manitoba, Multiple

Sclerosis Society of Canada, Multiple Sclerosis Scientific Foundation, National

Multiple Sclerosis Society, Rx & D Health Research Foundation and has conducted

clinical trials funded by Sanofi-Aventis. The other authors report no conflicts

of interest in this work.

55. Br J Radiol. 2017 Jun;90(1074):20160721. doi: 10.1259/bjr.20160721. Epub 2017 Apr

26.

Multiple sclerosis update: use of MRI for early diagnosis, disease monitoring and

Excluded b/c no primary data (narrative review)

assessment of treatment related complications.

Igra MS(1), Paling D(2), Wattjes MP(3), Connolly DJA(1), Hoggard N(4).

Author information:

(1)1 Department of Neuroradiology, Royal Hallamshire Hospital, Sheffield, UK.

(2)2 Department of Clinical Neurology, Royal Hallamshire Hospital, Sheffield, UK.

(3)3 Department of Radiology and Nuclear Medicine, VU University Medical Center,

Amsterdam, Netherlands.

(4)4 Academic Unit of Radiology, University of Sheffield, Sheffield, UK.

MRI has long been established as the most sensitive in vivo technique for

detecting multiple sclerosis (MS) lesions. The 2010 revisions of the McDonald

Criteria have simplified imaging criteria, such that a diagnosis of MS can be

made on a single contrast-enhanced MRI scan in the appropriate clinical context.

New disease-modifying therapies have proven effective in reducing relapse rate

and severity. Several of these therapies, most particularly natalizumab, but also

dimethyl fumarate and fingolimod, have been associated with progressive

multifocal leukoencephalopathy (PML). PML-immune reconstitution inflammatory

syndrome (IRIS) has been recognized in patients following cessation of

natalizumab owing to PML, and discontinuation for other reasons can lead to the

phenomenon of rebound MS. These complications often provide a diagnostic dilemma

and have implications for imaging surveillance of patients. We demonstrate how

the updated McDonald Criteria aid the diagnosis of MS and describe the imaging

characteristics of conditions such as PML and PML-IRIS in the context of MS.

Potential imaging surveillance protocols are considered for the diagnosis and

assessment of complications. We will explain how changes in MS treatment are

leading to new imaging demands in order to monitor patients for disease

progression and treatment-related complications.

DOI: 10.1259/bjr.20160721

PMCID: PMC5602172 [Available on 2018-06-01]

PMID: 28362522 [Indexed for MEDLINE]

56. Pharmacoepidemiol Drug Saf. 2017 Jun;26(6):702-711. doi: 10.1002/pds.4207. Epub

Excluded b/c no oral DMD results

2017 Apr 3.

Adherence to disease-modifying therapies for multiple sclerosis and subsequent

hospitalizations.

Evans C(1), Marrie RA(2), Zhu F(3), Leung S(4), Lu X(5), Kingwell E(3), Zhao

Y(3), Tremlett H(3).

Author information:

(1)College of Pharmacy & Nutrition, University of Saskatchewan, Saskatoon, SK,

Canada.

(2)Departments of Internal Medicine and Community Health Sciences, University of

Manitoba, Winnipeg, MB, Canada.

(3)Department of Medicine (Neurology), University of British Columbia, Vancouver,

BC, Canada.

(4)Department of Community Health Sciences, University of Manitoba, Winnipeg, MB,

Canada.

(5)Saskatchewan Health Quality Council, Saskatoon, SK, Canada.

PURPOSE: The aim of this study was to examine the association between optimal

adherence to first-line disease-modifying therapies (DMT) for multiple sclerosis

(MS) and hospitalizations.

METHODS: We used population-based administrative data from three Canadian

provinces. All individuals receiving DMT (interferon-B-1b, interferon-B-1a, or

glatiramer acetate) between January 1, 1996, and December 31, 2011 (British

Columbia); March 31, 2012 (Manitoba); or March 31, 2014, (Saskatchewan) were

included. Adherence was estimated for the first year of DMT (year 0), using the

medication possession ratio (MPR). The association between optimal adherence

(MPR ≥ 80%) and all-cause and MS-specific hospitalizations in the subsequent 1,

2, and 5 years was assessed using Hurdle Poisson and logistic regression. Rate

and odds ratios were adjusted (aRR and aOR) for sociodemographic factors and

prior health-care utilization.

RESULTS: Overall, 4746 subjects were followed for a mean 7.8 (SD 4.0) years; 3598

(76%) were women. Optimal DMT adherence was achieved in 3564/4746 (75.1%)

subjects. Subsequent all-cause and MS-specific hospitalizations were lower for

subjects with optimal versus suboptimal adherence, but none reached statistical

significance (1-year period, aRR = 0.77, 95%CI: 0.47-1.26; aOR = 0.80, 95%CI:

0.52-1.25). Similar findings were observed in the 2-year and 5-year periods.

Prior health-care utilization (hospitalizations and medications) was associated

with future hospitalizations; for every additional medication class, the 5-year

all-cause hospitalization rate and likelihood of an MS-specific hospitalization

increased by 5% and 11%, respectively (aRR = 1.05, 95%CI: 1.02-1.07; and

aOR = 1.11, 95%CI: 1.07-1.14).

CONCLUSIONS: Hospitalization rates were lower in subjects with optimal DMT

adherence, but findings were not statistically significant. Prior hospitalization

and polypharmacy were associated with increased risk for future hospitalizations

in MS. Copyright © 2017 John Wiley & Sons, Ltd.

Copyright © 2017 John Wiley & Sons, Ltd.

DOI: 10.1002/pds.4207

PMID: 28370875 [Indexed for MEDLINE]

57. Patient Prefer Adherence. 2017 May 29;11:995-999. doi: 10.2147/PPA.S129356.

Excluded b/c no primary data (narrative review)

eCollection 2017.

Using a multidimensional unfolding approach to assess multiple sclerosis patient

preferences for disease-modifying therapy: a pilot study.

Sempere AP(1), Vera-Lopez V(2), Gimenez-Martinez J(1), Ruiz-Beato E(3), Cuervo

J(4), Maurino J(5).

Author information:

(1)Department of Neurology, Hospital General Universitario de Alicante, Alicante.

(2)Department of Statistics, University of Salamanca, Salamanca.

(3)Health Economics and Outcomes Research Unit, Roche Farma SA, Madrid.

(4)Health Economics and Outcomes Research, Oviedo.

(5)Medical Department, Roche Farma SA, Madrid, Spain.

PURPOSE: Multidimensional unfolding is a multivariate method to assess

preferences using a small sample size, a geometric model locating individuals and

alternatives as points in a joint space. The objective was to evaluate

relapsing-remitting multiple sclerosis (RRMS) patient preferences toward key

disease-modifying therapy (DMT) attributes using multidimensional unfolding.

PATIENTS AND METHODS: A cross-sectional pilot study in RRMS patients was

conducted. Drug attributes included relapse prevention, disease progression

prevention, side-effect risk and route and schedule of administration. Assessment

of preferences was performed through a five-card game. Patients were asked to

value attributes from 1 (most preferred) to 5 (least preferred).

RESULTS: A total of 37 patients were included; the mean age was 38.6 years, and

78.4% were female. Disease progression prevention was the most important factor

(51.4%), followed by relapse prevention (40.5%). The frequency of administration

had the lowest preference rating for 56.8% of patients. Finally, 19.6% valued the

side-effect risk attribute as having low/very low importance.

CONCLUSION: Patients' perspective for DMT attributes may provide valuable

information to facilitate shared decision-making. Efficacy attributes were the

most important drug characteristics for RRMS patients. Multidimensional unfolding

seems to be a feasible approach to assess preferences in multiple sclerosis

patients. Further elicitation studies using multidimensional unfolding with other

stated choice methods are necessary to confirm these findings.

DOI: 10.2147/PPA.S129356

PMCID: PMC5460668

PMID: 28615928

Conflict of interest statement: Disclosure The study was funded by Roche Farma

SA, Spain. Elena Ruiz-Beato and Jorge Maurino are employees of Roche Farma SA.

The other authors report no conflicts of interest in this work.

Excluded b/c no real-world adherence/persistence

58. Ann Clin Transl Neurol. 2017 May 17;4(7):506-511. doi: 10.1002/acn3.410.

eCollection 2017 Jul.

Cladribine to treat disease exacerbation after fingolimod discontinuation in

progressive multiple sclerosis.

Alvarez-Gonzalez C(1)(2), Adams A(3), Mathews J(4), Turner BP(1)(2), Giovannoni

G(1)(2), Baker D(1), Schmierer K(1)(2).

Author information:

(1)BartsMSBlizard Institute (Neuroscience)Queen Mary University of

LondonLondonUnited Kingdom.

(2)Emergency Care & Acute Medicine Neuroscience Clinical Academic GroupBarts

Health NHS TrustLondonUnited Kingdom.

(3)Department of NeuroradiologySt Bartholomew's HospitalBarts Health NHS

TrustLondonUnited Kingdom.

(4)PharmacyBarts Health NHS TrustLondonUnited Kingdom.

Rebound disease following cessation of disease modifying treatment (DMT) has been

reported in people with both relapsing and progressive multiple sclerosis (pwRMS,

pwPMS) questioning strict separation between these two phenotypes. While licensed

DMT is available for pwRMS to counter rebound disease, no such option exists for

pwPMS. We report on a pwPMS who developed rebound disease, with 45

Gadolinium-enhancing lesions on T1 weighted MRI brain, within 6 months after

fingolimod 0.5 mg/day was stopped. Treatment with a short course of subcutaneous

cladribine 60 mg led to effective suppression of inflammatory activity and

partial recovery with no short-term safety issues or adverse events.

DOI: 10.1002/acn3.410

PMCID: PMC5497536

PMID: 28695150

59. Mult Scler. 2017 May 1:1352458517709956. doi: 10.1177/1352458517709956. [Epub

ahead of print]

Three-year clinical outcomes of relapsing multiple sclerosis patients treated

with dimethyl fumarate in a United States community health center.

Smoot K(1), Spinelli KJ(2), Stuchiner T(1), Lucas L(1), Chen C(1), Grote L(1),

Baraban E(1), Kresa-Reahl K(1), Cohan S(1).

Author information:

(1)Providence Multiple Sclerosis Center, Providence Brain and Spine Institute,

Providence Health & Services, Portland, OR, USA.

(2)Regional Research Department, Providence Health & Services, Portland, OR, USA.

BACKGROUND: Following approval of dimethyl fumarate (DMF), we established a

registry of relapsing multiple sclerosis (RMS) patients taking DMF at our

community MS center.

OBJECTIVE: To track DMF patients' tolerability, disease progression, and

lymphopenia.

METHODS: Patients prescribed DMF for RMS from March 2013 to March 2016 were

prospectively enrolled ( N = 412). Baseline data, clinical relapses, magnetic

resonance imaging (MRI) activity, discontinuation, and lymphocyte counts were

captured through chart review.

RESULTS: The mean age of patients starting DMF was 49.4 ± 12.0 years and 70%

transitioned from a previous disease-modifying therapy (DMT). Of the patients,

38% discontinued DMF, 76% of whom discontinued due to side effects. Clinical

relapse and MRI activity were low. Comparing patients who transitioned from

interferon-β (IFN), glatiramer acetate (GA), or natalizumab (NTZ), patients

previously on NTZ had higher rates of relapse than those previously on GA

(annualized relapse rate p = 0.039, percent relapse p = 0.021). Grade III

lymphopenia developed in 11% of patients. Lymphopenia was associated with older

age ( p < 0.001) and longer disease duration ( p < 0.001).

CONCLUSION: Given the high rates of lymphopenia and discontinuation, it has

become our clinical practice to more closely scrutinize older patients and those

with a longer disease duration who are potential candidates for initiating DMF

therapy.

DOI: 10.1177/1352458517709956

PMID: 28537110

60. Clinicoecon Outcomes Res. 2017 Apr 28;9:251-260. doi: 10.2147/CEOR.S130334.

eCollection 2017.

Adherence to disease-modifying therapies and its impact on relapse, health

resource utilization, and costs among patients with multiple sclerosis.

Burks J(1), Marshall TS(2), Ye X(2).

Author information:

(1)Nova Southeastern University, Davie, FL.

(2)AbbVie Inc, Chicago, IL, USA.

PURPOSE: To evaluate adherence to disease-modifying therapies (DMTs) among

patients with multiple sclerosis (MS) initiating oral and injectable DMTs, and to

estimate the impact of adherence on relapse, health resource utilization, and

medical costs.

PATIENTS AND METHODS: Commercially insured MS patients (aged 18-65 years, two or

more MS diagnoses, one or more DMT claims) with continuous eligibility 12 months

before and after the first DMT claim date (index date) and no DMT claim during

the pre-index period were identified from a large commerical claims database for

the period from January 1, 2008, to September 30, 2015. Adherence to the index

DMT was measured by the 12-month post-index proportion of days covered (PDC) and

compared between oral and injectable DMT initiators. After adjustment for sex,

age at index DMT, and comorbidities, regression models examined the relationship

between adherence and relapse risk, MS-related health resource utilization, and

non-drug medical costs (2015 US$).

RESULTS: The study covered 12,431 patients and nine DMTs. Adherence to the index

DMT did not differ significantly between oral (n=1,018) and injectable (n=11,413)

DMTs when assessed by mean PDC (0.7257±0.2934 vs 0.7259±0.2869, respectively;

P=0.0787), or percentages achieving PDC ≥0.8 (61.4% vs 58.6%, respectively;

P=0.0806). Compared to non-adherence, adherence to DMT significantly reduced the

likelihood of relapse in the post-index 12 months by 42%, hospitalization by 52%,

and emergency visits by 38% (all, P<0.0001). Adherent patients would be expected

to have on average 0.7 fewer outpatient visits annually versus non-adherent

patients (P<0.0001). Based on the differences in predicted mean costs, adherence

(vs non-adherence) would decrease the total annual medical care costs by $5,816

per patient, including hospitalization costs by $1,953, emergency visits by $171,

and outpatient visits by $2,802.

CONCLUSION: Adherence remains suboptimal but comparable between oral and

injectable DMTs. Potential health and economic benefits underscore the importance

of improving adherence in MS.

DOI: 10.2147/CEOR.S130334

PMCID: PMC5417677

PMID: 28496344

Conflict of interest statement: Disclosure This study was supported by AbbVie.

AbbVie participated in study design, research, data collection, analysis and

interpretation of data, writing, reviewing and approving the publication. JB is a

professor and director of an MS program at Nova Southeastern University. He has

provided consulting services for AbbVie, Bayer, Biogen, Genzyme, and Novartis.

TSM and XY are employees of AbbVie, and may own stocks/stock options in the

company. The authors report no other conflicts of interest in this work.

Excluded b/c no real-world adherence/persistence

61. Cochrane Database Syst Rev. 2017 Apr 25;4:CD012200. doi:

10.1002/14651858.CD012200.pub2.

Treatment with disease-modifying drugs for people with a first clinical attack

suggestive of multiple sclerosis.

Filippini G(1), Del Giovane C(2), Clerico M(3), Beiki O(4)(5), Mattoscio M(6),

Piazza F(3), Fredrikson S(4), Tramacere I(1), Scalfari A(6), Salanti G(7).

Author information:

(1)Scientific Direction, Fondazione IRCCS, Istituto Neurologico Carlo Besta, via

Celoria, 11, Milan, Italy, 20133.

(2)Cochrane Italy, Department of Diagnostic, Clinical and Public Health Medicine,

University of Modena and Reggio Emilia, Via del Pozzo 71, Modena, Italy, 41124.

(3)University of Turin, Division of Neurology, AOU San Luigi Gonzaga, Regione

Gonzole, 13, Orbassano, Torino, Italy, 10043.

(4)Department of Clinical Neuroscience, Karolinska Institutet, Stockholm, Sweden,

17177.

(5)Department of Epidemiology and Biostatistics, Kermanshah University of Medical

Sciences, Kermanshah, Iran.

(6)Department of Medicine, Division of Brain Sciences, Centre for Neuroscience,

Wolfson Neuroscience Laboratories, Imperial College London, Du Cane Road, London,

UK, W12 0NN.

(7)Institute of Social and Preventive Medicine (ISPM), University of Bern,

Finkenhubelweg 11, Bern, Switzerland, 3005.

BACKGROUND: The treatment of multiple sclerosis has changed over the last 20

years. The advent of disease-modifying drugs in the mid-1990s heralded a period

of rapid progress in the understanding and management of multiple sclerosis. With

the support of magnetic resonance imaging early diagnosis is possible, enabling

treatment initiation at the time of the first clinical attack. As most of the

disease-modifying drugs are associated with adverse events, patients and

clinicians need to weigh the benefit and safety of the various early treatment

options before taking informed decisions.

OBJECTIVES: 1. to estimate the benefit and safety of disease-modifying drugs that

have been evaluated in all studies (randomised or non-randomised) for the

treatment of a first clinical attack suggestive of MS compared either with

placebo or no treatment;2. to assess the relative efficacy and safety of

disease-modifying drugs according to their benefit and safety;3. to estimate the

benefit and safety of disease-modifying drugs that have been evaluated in all

studies (randomised or non-randomised) for treatment started after a first attack

('early treatment') compared with treatment started after a second attack or at

another later time point ('delayed treatment').

SEARCH METHODS: We searched the Cochrane Multiple Sclerosis and Rare Diseases of

the CNS Group Trials Register, MEDLINE, Embase, CINAHL, LILACS,

clinicaltrials.gov, the WHO trials registry, and US Food and Drug Administration

(FDA) reports, and searched for unpublished studies (until December 2016).

SELECTION CRITERIA: We included randomised and observational studies that

evaluated one or more drugs as monotherapy in adult participants with a first

clinical attack suggestive of MS. We considered evidence on alemtuzumab,

azathioprine, cladribine, daclizumab, dimethyl fumarate, fingolimod, glatiramer

acetate, immunoglobulins, interferon beta-1b, interferon beta-1a (Rebif®,

Avonex®), laquinimod, mitoxantrone, natalizumab, ocrelizumab, pegylated

interferon beta-1a, rituximab and teriflunomide.

DATA COLLECTION AND ANALYSIS: Two teams of three authors each independently

selected studies and extracted data. The primary outcomes were

disability-worsening, relapses, occurrence of at least one serious adverse event

(AE) and withdrawing from the study or discontinuing the drug because of AEs.

Time to conversion to clinically definite MS (CDMS) defined by Poser diagnostic

criteria, and probability to discontinue the treatment or dropout for any reason

were recorded as secondary outcomes. We synthesized study data using

random-effects meta-analyses and performed indirect comparisons between drugs. We

calculated odds ratios (OR) and hazard ratios (HR) along with relative 95%

confidence intervals (CI) for all outcomes. We estimated the absolute effects

only for primary outcomes. We evaluated the credibility of the evidence using the

GRADE system.

MAIN RESULTS: We included 10 randomised trials, eight open-label extension

studies (OLEs) and four cohort studies published between 2010 and 2016. The

overall risk of bias was high and the reporting of AEs was scarce. The quality of

the evidence associated with the results ranges from low to very low. Early

treatment versus placebo during the first 24 months' follow-upThere was a small,

non-significant advantage of early treatment compared with placebo in

disability-worsening (6.4% fewer (13.9 fewer to 3 more) participants with

disability-worsening with interferon beta-1a (Rebif®) or teriflunomide) and in

relapses (10% fewer (20.3 fewer to 2.8 more) participants with relapses with

teriflunomide). Early treatment was associated with 1.6% fewer participants with

at least one serious AE (3 fewer to 0.2 more). Participants on early treatment

were on average 4.6% times (0.3 fewer to 15.4 more) more likely to withdraw from

the study due to AEs. This result was mostly driven by studies on interferon beta

1-b, glatiramer acetate and cladribine that were associated with significantly

more withdrawals for AEs. Early treatment decreased the hazard of conversion to

CDMS (HR 0.53, 95% CI 0.47 to 0.60). Comparing active interventions during the

first 24 months' follow-upIndirect comparison of interferon beta-1a (Rebif®) with

teriflunomide did not show any difference on reducing disability-worsening (OR

0.84, 95% CI 0.43 to 1.66). We found no differences between the included drugs

with respect to the hazard of conversion to CDMS. Interferon beta-1a (Rebif®) and

teriflunomide were associated with fewer dropouts because of AEs compared with

interferon beta-1b, cladribine and glatiramer acetate (ORs range between 0.03 and

0.29, with substantial uncertainty). Early versus delayed treatmentWe did not

find evidence of differences between early and delayed treatments for

disability-worsening at a maximum of five years' follow-up (3% fewer participants

with early treatment (15 fewer to 11.1 more)). There was important variability

across interventions; early treatment with interferon beta-1b considerably

reduced the odds of participants with disability-worsening during three and five

years' follow-up (OR 0.52, 95% CI 0.32 to 0.84 and OR 0.57, 95% CI 0.36 to 0.89).

The early treatment group had 19.6% fewer participants with relapses (26.7 fewer

to 12.7 fewer) compared to late treatment at a maximum of five years' follow-up

and early treatment decreased the hazard of conversion to CDMS at any follow-up

up to 10 years (i.e. over five years' follow-up HR 0.62, 95% CI 0.53 to 0.73). We

did not draw any conclusions on long-term serious AEs or discontinuation due to

AEs because of inadequacies in the available data both in the included OLEs and

cohort studies.

AUTHORS' CONCLUSIONS: Very low-quality evidence suggests a small and uncertain

benefit with early treatment compared with placebo in reducing

disability-worsening and relapses. The advantage of early treatment compared with

delayed on disability-worsening was heterogeneous depending on the actual drug

used and based on very low-quality evidence. Low-quality evidence suggests that

the chances of relapse are less with early treatment compared with delayed. Early

treatment reduced the hazard of conversion to CDMS compared either with placebo,

no treatment or delayed treatment, both in short- and long-term follow-up.

Low-quality evidence suggests that early treatment is associated with fewer

participants with at least one serious AE compared with placebo. Very low-quality

evidence suggests that, compared with placebo, early treatment leads to more

withdrawals or treatment discontinuation due to AEs. Difference between drugs on

short-term benefit and safety was uncertain because few studies and only indirect

comparisons were available. Long-term safety of early treatment is uncertain

because of inadequately reported or unavailable data.

DOI: 10.1002/14651858.CD012200.pub2

PMID: 28440858 [Indexed for MEDLINE]

62. ACS Chem Neurosci. 2017 Apr 19;8(4):860-865. doi: 10.1021/acschemneuro.6b00428.

Excluded b/c no primary data (narrative review)

Epub 2017 Jan 3.

Enhanced Brain Delivery of Dimethyl Fumarate Employing Tocopherol-Acetate-Based

Nanolipidic Carriers: Evidence from Pharmacokinetic, Biodistribution, and

Cellular Uptake Studies.

Kumar P(1), Sharma G, Kumar R, Malik R(1), Singh B, Katare OP, Raza K(1).

Author information:

(1)Department of Pharmacy, School of Chemical Sciences and Pharmacy, Central

University of Rajasthan , Bandar Sindri, Distt. Ajmer, Rajasthan, India -305817.

Dimethyl fumarate (DMF) is an approved drug for the management of relapsing

multiple sclerosis. Despite efficacy, DMF is also reported to be a challenging

drug owing to concerns like gastrointestinal tract flushing, multiple dosing,

lower brain permeability, less patient compliance, and economic hurdles. The

present study aims to develop DMF-tocopherol acetate nanolipidic carrier (NLCs)

to enhance brain permeability and improve the gastric tolerance. The developed

DMF-tocopherol acetate NLCs offered an average size of 69.70 nm, PDI of 0.317,

and a zeta potential of -9.71 mV. Higher drug entrapment (90.12%) and drug

loading (20.13%) assured controlled drug release behavior both in gastric and

intestinal pH. Cellular uptake studies on Caco-2 and SH-SY5Y monolayers confirmed

better intestinal absorption and neuronal uptake of the developed system, which

was further corroborated by the pharmacokinetic and biodistribution studies. The

oral bioavailability was enhanced by 4.09 times and brain availability was

substantially improved vis-à-vis plain drug. The findings are promising and offer

preclinical evidence for better brain availability of DMF, which can be exploited

in the better management of diseases like multiple sclerosis.

DOI: 10.1021/acschemneuro.6b00428

PMID: 27983793 [Indexed for MEDLINE]

63. Autoimmun Rev. 2017 Apr;16(4):355-376. doi: 10.1016/j.autrev.2017.02.007. Epub

Excluded b/c no primary data (narrative review)

2017 Feb 15.

Multiple sclerosis in the real world: A systematic review of fingolimod as a case

study.

Ziemssen T(1), Medin J(2), Couto CA(3), Mitchell CR(4).

Author information:

(1)Center of Clinical Neuroscience, University Hospital Carl Gustav Carus,

Dresden University of Technology, Dresden D-01307, Germany. Electronic address:

tjalf.ziemssen@uniklinikum-dresden.de.

(2)Novartis Pharma AG, CH-4002 Basel, Switzerland. Electronic address:

jennie.medin@novartis.com.

(3)Value Demonstration Practice, Oxford PharmaGenesis, Oxford OX13 5QJ, UK.

Electronic address: anne-marie.couto@pharmagenesis.com.

(4)Value Demonstration Practice, Oxford PharmaGenesis, Oxford OX13 5QJ, UK.

Electronic address: catherine.mitchell@pharmagenesis.com.

INTRODUCTION: The aim of our study was to systematically review the growing body

of published literature reporting on one specific multiple sclerosis (MS)

treatment, fingolimod, in the real world to assess its effectiveness in patients

with MS, evaluate methodologies used to investigate MS in clinical practice, and

describe the evidence gaps for MS as exemplified by fingolimod.

METHODS: We conducted a PRISMA-compliant systematic review of the literature

(cut-off date: 4 March 2016). Published papers reporting real-world data for

fingolimod with regard to clinical outcomes, persistence, adherence, healthcare

costs, healthcare resource use, treatment patterns, and patient-reported outcomes

that met all the eligibility criteria were included for data extraction and

quality assessment.

RESULTS AND DISCUSSION: Based on 34 included studies, this analysis found that

fingolimod treatment improved outcomes compared to the period before treatment

initiation and was more effective than interferons or glatiramer acetate.

However, among studies comparing fingolimod with natalizumab, overall trends were

inconsistent: some reported natalizumab to be more effective than fingolimod and

others reported similar effectiveness for natalizumab and fingolimod. These

studies illustrate the challenges of investigating MS in the real world,

including the subjectivity in evaluating some clinical outcomes and the

heterogeneity of methodologies used and patient populations investigated, which

limit comparisons across studies. Gaps in available real-world evidence for MS

are also highlighted, including those relating to patient-reported outcomes,

combined clinical outcomes (to measure overall treatment effectiveness), and

healthcare costs/resource use.

CONCLUSIONS: The included studies provide good evidence of the real-world

effectiveness of fingolimod and highlight the diversity of methodologies used to

assess treatment benefit in clinical practice. Future studies could address the

evidence gaps found in the literature and the challenges associated with

researching MS when designing real-world studies, assessing data, and comparing

evidence across studies.

Copyright © 2017 The Authors. Published by Elsevier B.V. All rights reserved.

DOI: 10.1016/j.autrev.2017.02.007

PMID: 28212923 [Indexed for MEDLINE]

64. Mult Scler. 2017 Apr;23(4):588-596. doi: 10.1177/1352458516657440. Epub 2016 Jul

11.

Excluded b/c no oral DMD results

Determinants of non-adherence to disease-modifying therapies in multiple

sclerosis: A cross-Canada prospective study.

McKay KA(1), Tremlett H(1), Patten SB(2), Fisk JD(3), Evans C(4), Fiest K(5),

Campbell T(6), Marrie RA(7); CIHR Team in the Epidemiology and Impact of

Comorbidity on Multiple Sclerosis (ECoMS).

Author information:

(1)Division of Neurology, Faculty of Medicine, Djavad Mowafaghian Centre for

Brain Health, University of British Columbia, Vancouver, BC, Canada.

(2)Departments of Psychiatry and Community Health Sciences, Cumming School of

Medicine, University of Calgary, Calgary, AB, Canada.

(3)Departments of Psychiatry, Psychology and Neuroscience, Dalhousie University,

Halifax, NS, Canada.

(4)College of Pharmacy and Nutrition, University of Saskatchewan, Saskatoon, SK,

Canada.

(5)Departments of Critical Care Medicine & Community Health Sciences, O'Brien

Institute for Public Health, and Hotchkiss Brain Institute, University of

Calgary, Canada.

(6)School of Nursing, Faculty of Health Professions, Dalhousie University,

Halifax, NS, Canada.

(7)Departments of Internal Medicine and Community Health Sciences, Health

Sciences Centre, Max Rady College of Medicine, Rady Faculty of Health Sciences,

University of Manitoba, Winnipeg, MB, Canada.

BACKGROUND: Poor adherence to the disease-modifying therapies (DMTs) for multiple

sclerosis (MS) may attenuate clinical benefit. A better understanding of

characteristics associated with non-adherence could improve outcomes.

OBJECTIVE: To evaluate characteristics associated with non-adherence to

injectable DMTs.

METHODS: Consecutive patients from four Canadian MS Clinics were assessed at

three time points over two years. Clinical and demographic information included

self-reported DMT use, missed doses in the previous 30 days, health behaviors,

and comorbidities. Non-adherence was defined as <80% of expected doses taken. We

employed generalized estimating equations to examine characteristics associated

with non-adherence at all time points with findings reported as adjusted odds

ratios (OR).

RESULTS: In all, 485 participants reported use of an injectable DMT, of whom 107

(22.1%) were non-adherent over the study period. Non-adherence was associated

with a lower Expanded Disability Status Scale score (0-2.5 vs 3.0-5.5, OR: 1.80;

95% confidence interval (CI): 1.06-3.04), disease duration (⩽5 vs <5 years, OR:

2.23; 95% CI: 1.10-4.52), alcohol dependence (OR: 2.14; 95% CI: 1.23-3.75), and

self-reported cognitive difficulties, measured by the Health Utilities Index-3

(OR: 1.55; 95% CI: 1.08-2.22).

CONCLUSIONS: Nearly one-quarter of participants were non-adherent during the

study. Alcohol dependence, perceived cognitive difficulties, longer disease

duration, and mild disability status were associated with non-adherence. These

characteristics may help healthcare professionals identify patients at greatest

risk of poor adherence.

DOI: 10.1177/1352458516657440

PMCID: PMC5407504

PMID: 27357507 [Indexed for MEDLINE]

65. Mult Scler Relat Disord. 2017 Apr;13:107-111. doi: 10.1016/j.msard.2017.02.016.

Excluded b/c no oral DMD results

Epub 2017 Feb 23.

Factors associated with adherence to disease modifying therapy in multiple

sclerosis: An observational survey from a referral center in Lithuania.

Duchovskiene N(1), Mickeviciene D(1), Jurkeviciene G(1), Dirziuviene B(2),

Balnyte R(1).

Author information:

(1)Neurology clinic, Lithuanian University of Health Sciences, Eiveniu str. 2,

Kaunas, Lithuania.

(2)Neurology clinic, Lithuanian University of Health Sciences, Eiveniu str. 2,

Kaunas, Lithuania. Electronic address: biruteveb@gmail.com.

AIM OF THE STUDY: To investigate adherence to disease modifying therapy (DMT) in

Lithuanian population of multiple sclerosis patients and factors associated to

it.

METHODS: Patients receiving one of the following DMT's: Interferon β 1a (Rebif)

44 micrograms three times a week subdermally (s/c) or Interferon β 1a (Avonex) 30

micrograms weekly intramuscularly (i/m), or Interferon β 1b (Betaferon, Extavia)

250 micrograms once in two days s/c, or Glatiramer acetate (Copaxone) 20mg daily

s/c, were presented with a questionnaire inquiring their demographic and clinical

characteristics and adherence to treatment profile, as well as HAD scale and

SF-36 questionnaire. Those who missed at least one dose of DMT during last three

months were considered non-adherent.

RESULTS: In total, 207 patients were enrolled, 73 (35.3%) of them were non

adherent during last three months. More patients with university education

(p=0.004, χ2 =8.466 high school/vocational vs. university) as well as consuming

>4 units/year of alcohol were non-adherent during last three months (p=0.005).

Average score for anxiety (6.69±4.03 vs. 6.92±4.24) and depression (4.74±3.9 vs.

4.7±3.83) in HAD scale did not differ significantly between adherent and

non-adherent groups. We did not find any significant difference in quality of

life scores (SF-36 v.2) between the groups. No significant difference of

adherence was found then comparing patients often suffering from drug side

effects with those who experience rare or no side effects. In logistic regression

model, patients consuming more than 4 alcohol units per year and patients with

university education were more likely to miss at least one dose during last three

months: 2.121 (95% CI: 1.143-3.937, p=0.017) and 2.409 (95% CI: 1.260-4.642,

p=0.008) times accordingly. Patients with better quality of life scores were

slightly less likely to be non adherent (OR 0.997 (95% CI: 0.994-0.999),

p=0.0017).

CONCLUSIONS: One third of patients were non-adherent during last three months.

Worse adherence rates were associated with higher education and higher alcohol

consumption. Education, alcohol consumption and quality of life scores were found

to be significant factors for predicting non-adherence. We found no associations

between adherence and anxiety, depression, or drugs side effects.

Copyright © 2017 Elsevier B.V. All rights reserved.

DOI: 10.1016/j.msard.2017.02.016

PMID: 28427690 [Indexed for MEDLINE]

66. Neurol Sci. 2017 Apr;38(4):589-594. doi: 10.1007/s10072-016-2806-4. Epub 2017 Jan

Excluded b/c no oral DMD results

11.

Changes in first-line injectable disease-modifying therapy for multiple

sclerosis: predictors of non-adherence, switching, discontinuation, and

interruption of drugs.

Degli Esposti L(1), Piccinni C(2), Sangiorgi D(3), Perrone V(3), Aledda L(3),

Marrosu MG(4), Lombardo F(5).

Author information:

(1)Clicon Srl, Health, Economics, and Outcomes Research, Via Salara, 36, 48100,

Ravenna, Italy. luca.degliesposti@clicon.it.

(2)Pharmacology Unit, Department of Medical and Surgical Sciences, University of

Bologna, Bologna, Italy.

(3)Clicon Srl, Health, Economics, and Outcomes Research, Via Salara, 36, 48100,

Ravenna, Italy.

(4)Dipartimento di Scienze Mediche, University of Cagliari, Cagliari, Italy.

(5)Farmacia Ospedale R. Binaghi, Azienda Sanitaria Locale di Cagliari, Cagliari,

Italy.

This study was aimed to describe changes of Disease-Modifying Treatments (DMT) in

an Italian cohort of patients with multiple sclerosis (MS) and to identify

predictors of therapeutic modifications. Patients with MS and treated with the

first-line injectable DMT (interferons-IFNs or glatiramer) between 1/7/2009 and

31/10/2012 were selected from administrative databases of the MS Center of

Cagliari (Sardinia, Italy). Socio-demographic, therapeutic, and clinical

information was collected in the 6 months preceding the index date. All patients

were followed for 36 months to evaluate therapeutic changes in terms of

non-adherence, switch, temporary discontinuation, and permanent interruption.

Predictors of changes were estimated by multivariable regression models. Data on

1698 patients were collected: glatiramer was prescribed in 27% of cases, IFNβ-1b

in 22%, IFNβ-1a-im in 20%, IFNβ-1a-sc-44mcg in 19%, and IFNβ-1a-sc-22mcg in 12%.

Non-adherence was observed in 25% of cases, therapeutic switch in 30%,

discontinuation in 37%, and permanent interruption in 28%. The risk of

non-adherence was higher for IFNβ-1b, compared with IFNβ-1a-im (adjOR = 1.73).

Therapeutic switch occurred especially in patients recently diagnosed (each year

from diagnosis causes a decrease of this risk adjHR = 0.97); the risk of

discontinuation was higher with EDSS = 4-6 and 7-9 (adjHR = 1.52 and 4.42,

respectively). The risk of permanent interruption increased with the augmentation

of disability (adjHR = 1.67 and 5.43 for EDSS 4-6 and 7-9). This study mirrored a

detailed framework of DMT prescription and identified factors related to changes

in the MS therapy. These findings could support healthcare providers in the

evaluation and maximization of benefits associated with a long-term DMT.

DOI: 10.1007/s10072-016-2806-4

PMID: 28078563 [Indexed for MEDLINE]

67. Mult Scler J Exp Transl Clin. 2017 Mar 17;3(1):2055217317696114. doi:

Excluded b/c no oral DMD results

10.1177/2055217317696114. eCollection 2017 Jan-Mar.

Assessment of treatment patterns associated with injectable disease-modifying

therapy among relapsing-remitting multiple sclerosis patients.

Nicholas J(1), Ko JJ, Park Y(2), Navaratnam P, Friedman HS(3), Ernst FR(4),

Herrera V(2).

Author information:

(1)OhioHealth MS Center, Riverside Methodist Hospital, USA.

(2)Novartis Pharmaceuticals Corporation, USA.

(3)DataMed Solutions LLC, USA.

(4)Indegene, USA.

BACKGROUND: Availability of oral disease-modifying therapy (DMT) for

relapsing-remitting multiple sclerosis (RRMS) may affect injectable DMT (iDMT)

treatment patterns.

OBJECTIVE: The objective of this paper is to evaluate iDMT persistency, reasons

for persistency lapses, and outcomes among newly diagnosed RRMS patients.

METHODS: Medical records of 300 RRMS patients initiated on iDMT between 2008 and

2013 were abstracted from 18 US-based neurology clinics. Eligible patients had ≥3

visits: pre-iDMT initiation, iDMT initiation (index), and ≥1 visit within 24

months post-index. MS-related symptoms, relapses, iDMT treatment patterns (i.e.

persistency, discontinuation, switching, and restart), and reasons for

non-persistency were tracked for 24 months.

RESULTS: At 24 months, iDMT persistency was 61.0%; 28.0% of patients switched to

another DMT, 8.0% discontinued, and 3.0% stopped and restarted the same iDMT. The

most commonly identified reasons for non-persistency were perceived lack of

efficacy (22.2%), adverse events (18.8%), and fear of needles/self-injecting

(9.4%). At 24 months, 38.0% of patients had experienced a relapse and 11.0% had

changes in MRI lesion counts. Patients without MS-related symptoms at index

reported increases in the incidence of these symptoms at 24 months.

CONCLUSIONS: Non-persistency with iDMT remains an issue in the oral DMT age. Many

patients still experienced relapses and disease progression, and should consider

switching to more effective therapies.

DOI: 10.1177/2055217317696114

PMCID: PMC5459267

PMID: 28607751

68. Brain Behav. 2017 Mar 14;7(4):e00671. doi: 10.1002/brb3.671. eCollection 2017

Excluded b/c no real-world adherence/persistence

Apr.

Catastrophic outcome of patients with a rebound after Natalizumab treatment

discontinuation.

González-Suarez I(1), Rodríguez de Antonio L(2), Orviz A(1), Moreno-García S(3),

Valle-Arcos MD(3), Matias-Guiu JA(1), Valencia C(1), Jorquera Moya M(4),

Oreja-Guevara C(1).

Author information:

(1)Neurology DepartmentMultiple Sclerosis CenterIdiSSCHospital Clinico San

CarlosMadridSpain.

(2)Neurology DepartmentHospital Universitario de FuenlabradaMadridSpain.

(3)Demyelinating Disease UnitNeurology DepartmentHospital Universitario 12 de

OctubreMadridSpain.

(4)Radiology DepartmentIdiSSCHospital Clinico San CarlosMadridSpain.

INTRODUCTION: Natalizumab (NTZ) is an effective drug for the treatment of

relapsing-remitting multiple sclerosis. In some patients discontinuation is

mandatory due to the risk of progressive multifocal leukoencephalopathy. However,

severe clinical and radiological worsening has been described after drug

cessation. Our aim was to describe the clinical and radiological features of the

rebound phenomenon.

MATERIAL AND METHODS: Patients switched from NTZ to Fingolimod (FTY) who had

presented a rebound after discontinuation were selected. Clinical and magnetic

resonance imaging (MRI) data were collected.

RESULTS: Four JC virus positive patients were included. The mean disease duration

was 9.5 years (SD: 4.12) with a mean time of 3.1 years on NTZ. All patients

started FTY within 3-4 months. Neurological deterioration started in a mean time

of 3.5 months (SD: 2.08) with multifocal involvement: 75% motor disturbances, 50%

cognitive impairment, 25% seizures. The average worsening in Expanded Disability

Status Scale [EDSS] was of 3.25 points (SD: 2.33). The MRI showed a very large

increase in T2 and gadolinium-enhanced lesions (mean: 23.67, SD: 18.58). All

patients received 5 days of IV methylprednisolone, one patient required plasma

exchange. All the patients presented neurological deterioration with an EDSS

worsening of 1.13 points (SD: 0.48). After the rebound three patients continued

treatment with FTY, only one patient restarted NTZ.

CONCLUSION: Discontinuation of NTZ treatment may trigger a severe rebound with

marked clinical and radiological worsening. A very careful evaluation of

benefit-risk should be considered before NTZ withdrawal, and a close monitoring

and a short washout period is recommended after drug withdrawal.

DOI: 10.1002/brb3.671

PMCID: PMC5390845

PMID: 28413713 [Indexed for MEDLINE]

69. Health Qual Life Outcomes. 2017 Mar 14;15(1):50. doi: 10.1186/s12955-017-0622-z.

Excluded b/c no oral DMD results

Glatiramer acetate treatment persistence - but not adherence - in multiple

sclerosis patients is predicted by health-related quality of life and

self-efficacy: a prospective web-based patient-centred study (CAIR study).

Jongen PJ(1)(2), Lemmens WA(3), Hoogervorst EL(4), Donders R(3).

Author information:

(1)University Medical Centre Groningen, Department of Community and Occupational

Medicine, University Groningen, Antonius Deusinglaan 1, 9713, AV, Groningen, The

Netherlands. p.j.h.jongen@rug.nl.

(2)MS4 Research Institute, Ubbergseweg 34, 6522, KJ, Nijmegen, The Netherlands.

p.j.h.jongen@rug.nl.

(3)Department for Health Evidence, Radboud University Medical Centre, P.O. Box

9101, 6500, HB, Nijmegen, The Netherlands.

(4)St. Antonius Hospital, P.O. Box 2500, 3430, EM, Nieuwegein, The Netherlands.

BACKGROUND: In patients with relapsing remitting multiple sclerosis (RRMS) the

persistence of and adherence to disease modifying drug (DMD) treatment is

inadequate. To take individualised measures there is a need to identify patients

with a high risk of non-persistence or non-adherence. As patient-related factors

have a major influence on persistence and adherence, we investigated whether

health-related quality of life (HRQoL) and self-efficacy could predict

persistence or adherence.

METHODS: In a prospective web-based patient-centred study in 203 RRMS patients,

starting treatment with glatiramer acatete (GA) 20 mg subcutaneously daily, we

measured physical and mental HRQoL (Multiple Sclerosis Quality of Life-54

questionnaire), functional and control self-efficacy (Multiple Sclerosis

Self-Efficacy Scale), the 12-month persistence rate and, in persistent patients,

the percentage of missed doses. HRQoL and self-efficacy were compared between

persistent and non-persistent patients, and between adherent and non-adherent

patients. Logistic regression analysis was used to assess whether persistence and

adherence were explained by HRQoL and self-efficacy.

RESULTS: Persistent patients had higher baseline physical (mean 58.1 [standard

deviation, SD] 16.9) and mental HRQoL (63.8 [16.8]) than non-persistent patients

(49.5 [17.6]; 55.9 [20.4]) (P = 0.001; P = 0.003) with no differences between

adherent and non-adherent patients (P = 0.46; P = 0.54). Likewise, in persistent

patients function (752 [156]) and control self-efficacy (568 [178]) were higher

than in non-persistent patients (689 [173]; 491 [192]) (P = 0.009; P = 0.004),

but not in adherent vs. non-adherent patients (P = 0.26; P = 0.82). Logistic

regression modelling identified physical HRQoL and control self-efficacy as

factors that explained persistence. Based on predicted scores from the model,

patients were classified into quartiles and the percentage of non-persistent

patients per quartile was calculated: non-persistence in the highest quartile was

23.4 vs. 53.2% in the lowest quartile. Risk differentiation with respect to

adherence was not possible. Based on these findings we propose a practical

work-up scheme to identify patients with a high risk of non-persistence and to

identify persistence-related factors.

CONCLUSIONS: Findings suggest that pre-treatment physical HRQoL and control

self-efficacy may identify RRMS patients with a high risk of early

discontinuation of injectable DMD treatment. Targeting of high-risk patients may

enable the efficient use of persistence-promoting measures.

TRIAL REGISTRATION: Nederlands Trial Register code: NTR2432 .

DOI: 10.1186/s12955-017-0622-z

PMCID: PMC5351176

PMID: 28292329 [Indexed for MEDLINE]

70. Health Qual Life Outcomes. 2017 Mar 9;15(1):47. doi: 10.1186/s12955-017-0614-z.

Excluded b/c no real-world adherence/persistence

The Performance Scales disability measure for multiple sclerosis: use and

sensitivity to clinically important differences.

Schwartz CE(1)(2), Powell VE(3).

Author information:

(1)DeltaQuest Foundation, Inc., 31 Mitchell Road, Concord, MA, 01742, USA.

carolyn.schwartz@deltaquest.org.

(2)Departments of Medicine and Orthopaedic Surgery, Tufts University Medical

School, Boston, MA, USA. carolyn.schwartz@deltaquest.org.

(3)DeltaQuest Foundation, Inc., 31 Mitchell Road, Concord, MA, 01742, USA.

BACKGROUND: In 1993, the Performance Scales© was created to assess

multi-dimensional disability in multiple sclerosis (MS). This tool has been used

in a variety of settings and study designs internationally. The present work

provides an overview of the history and psychometric characteristics of the

Performance Scales©, reviews its use over the past two decades, and summarizes

its responsiveness to subgroup differences.

METHODS: A Google Scholar and Ovid search yielded 230 articles citing the

Performance Scales©, of which 82 studies used the tool in empirical research.

Twelve articles provided sufficient information to enable computation of effect

sizes. Forest plots were used to show effect sizes for the overall summary score

and by domain by patient demographics, MS disease trajectory, and treatment

adherence.

RESULTS: The Performance Scales© evidenced sensitivity to clinically important

differences by disease trajectory and age (for selected domains). In contrast,

groups distinguished by patient adherence to disease-modifying therapies and

ethnicity were relatively small.

CONCLUSIONS: The Performance Scales© has been used in a large number of studies

since its development, suggesting that this psychometrically sound tool is

acknowledged to be a useful tool for MS clinical research. It is recommended that

future work include the entire measure, so that the whole-person impact of MS can

be characterized and considered in MS outcome research.

DOI: 10.1186/s12955-017-0614-z

PMCID: PMC5343380

PMID: 28274258 [Indexed for MEDLINE]

71. Clin Immunol. 2017 Mar;176:87-93. doi: 10.1016/j.clim.2017.01.001. Epub 2017 Jan

Excluded b/c no real-world adherence/persistence

17.

From natalizumab to fingolimod in eight weeks - Immunological, clinical, and

radiological data in quest of the optimal switch.

Harrer A(1), Pilz G(2), Oppermann K(2), Sageder M(3), Afazel S(4), Haschke-Becher

E(4), Rispens T(5), de Vries A(6), McCoy M(7), Stevanovic V(7), Hitzl W(8),

Trinka E(2), Kraus J(9), Sellner J(2), Wipfler P(2).

Author information:

(1)Department of Neurology, Paracelsus Medical University Salzburg, Salzburg,

Austria. Electronic address: a.harrer@salk.at.

(2)Department of Neurology, Paracelsus Medical University Salzburg, Salzburg,

Austria.

(3)Department of Neurology, Paracelsus Medical University Salzburg, Salzburg,

Austria; Department of Molecular Biology, Paris-Lodron University, Salzburg,

Austria.

(4)Department of Laboratory Medicine, Paracelsus Medical University Salzburg,

Salzburg, Austria.

(5)Department of Immunopathology, Sanquin Research and Academic Centre,

Amsterdam, The Netherlands.

(6)Laboratory for Monoclonal Therapeutics, Sanquin Diagnostics, Amsterdam, The

Netherlands.

(7)Division of Neuroradiology, Paracelsus Medical University Salzburg, Salzburg,

Austria.

(8)Research Office, Biostatistics, Paracelsus Medical University Salzburg,

Salzburg, Austria.

(9)Department of Laboratory Medicine, Paracelsus Medical University Salzburg,

Salzburg, Austria; Research Institute for Neurointervention, Paracelsus Medical

University Salzburg, Salzburg, Austria; Department of Neurology, Heinrich-Heine

University, Düsseldorf, Germany.

Natalizumab (NZB) discontinuation during a treatment change is associated with

recurrence of disease activity in a significant proportion of multiple sclerosis

(MS) patients. The immunological basis why disease reactivation occurs in

selected patients is unresolved. In search of a prognostic biomarker for a safe

and effective transition from NZB to fingolimod, we monitored five parameters

related to pharmacokinetic and pharmacodynamic effects of the two drugs in 12 MS

patients until six months on fingolimod. Clearance of free and cell-bound NZB,

re-expression of alpha-4, and fingolimod-mediated changes on CD8+ and CD4+ T cell

subsets showed pronounced interindividual variability. Higher frequencies of

memory CD8+ T cells after six months on fingolimod were the sole association with

disease reactivation. None of the investigated parameters thus had potential as

prognostic biomarker for the outcome of the switch. Our findings rather support

the thesis of broad interindividual differences in the immunopathogenesis of MS.

Copyright © 2017 Elsevier Inc. All rights reserved.

DOI: 10.1016/j.clim.2017.01.001

PMID: 28108364 [Indexed for MEDLINE]

72. J Med Econ. 2017 Mar;20(3):297-302. doi: 10.1080/13696998.2016.1258366. Epub 2016

Nov 21.

Excluded b/c no real-world adherence/persistence

The cost-effectiveness of disease-modifying therapies for the treatment of

relapsing-remitting multiple sclerosis.

Bozkaya D(1), Livingston T(2), Migliaccio-Walle K(1), Odom T(2).

Author information:

(1)a Xcenda , Palm Harbor , FL , USA.

(2)b Biogen , Weston , MA , USA.

BACKGROUND: The safety and efficacy of disease-modifying therapies (DMTs) for

relapsing-remitting multiple sclerosis (RRMS) has been established; however, it

is not clear which provides optimal value, given benefit-risk profiles and costs.

AIMS: To compare the cost-effectiveness of current DMTs for patients with RRMS in

the US.

MATERIALS AND METHODS: A Markov model predicting RRMS course following initiation

of a DMT was created comparing outcomes (e.g. relapses, disease progression) and

costs of natalizumab (NTZ), dimethyl fumarate (DMF), and peginterferon beta-1a

(PEG) with fingolimod (FIN), glatiramer acetate (GA, 20 mg daily), and

subcutaneous interferon beta-1a (IFN, 44 mcg), respectively, over 10 years. RRMS

and secondary-progressive MS (SPMS) EDSS state transitions were predicted in

3-month cycles in which patients were at risk of death, relapse, or

discontinuation. Upon DMT discontinuation, natural history progression and

relapse rates were applied. Incremental cost-effectiveness ratios (ICERs) were

estimated for the cost per relapse avoided, relapse-free years gained,

progression avoided, and progression-free years gained. The impact of model

parameters on outcomes was evaluated via one-way sensitivity analyses.

RESULTS: Costs ranged from $561,177 (NTZ) to $616,251 (GA). NTZ, DMF, and PEG

were dominant (less costly and more effective) compared to FIN, GA, and IFN,

respectively, for all ICERs. Variability in drug costs and parameters that

affected drug cost accrual (e.g. discontinuation rates and the decision to drop

out after SPMS conversion) had a considerable impact on ICERs.

LIMITATIONS: Several simplifying assumptions were made that may represent

potential limitations of this analysis (e.g. a constant treatment effect over

time was assumed).

CONCLUSIONS: The results from this analysis suggest that the NTZ, DMF, and PEG

are cost-effective DMT choices compared to FIN, GA, and IFN, respectively. The

actual impact on a particular plan will vary based on drug pricing and other

factors affecting drug cost accrual.

DOI: 10.1080/13696998.2016.1258366

PMID: 27822961 [Indexed for MEDLINE]

73. J Med Econ. 2017 Mar;20(3):228-238. doi: 10.1080/13696998.2016.1247712. Epub 2016

Nov 4.

Excluded b/c no real-world adherence/persistence

Peginterferon beta-1a versus other self-injectable disease-modifying therapies in

the treatment of relapsing-remitting multiple sclerosis in Scotland: a

cost-effectiveness analysis.

Hernandez L(1), Guo S(1), Toro-Diaz H(1), Carroll S(2), Syed Farooq SF(2).

Author information:

(1)a Evidera Inc. , Waltham , MA , USA.

(2)b Biogen , Maidenhead , Berkshire, UK.

AIMS: Peginterferon beta-1a 125 mcg administered subcutaneously every 2 weeks, a

new disease-modifying therapy (DMT) for relapsing-remitting multiple sclerosis

(RRMS), was approved in January 2015 by the Scottish Medicines Consortium. This

study assesses long-term clinical and economic outcomes of peginterferon beta-1a

compared with other self-injectable DMTs (interferon beta-1a [22 mcg, 30 mcg, and

44 mcg], interferon beta-1b, and glatiramer acetate 20 mg) in the treatment of

RRMS, from the National Health Service and Personal Social Services perspective

in Scotland.

METHODS: A previously published, validated Markov cohort model was adapted for

this analysis. The model estimates changes in patient disability, occurrence of

relapses, and other adverse events, and translates them into quality-adjusted

life years and costs. Natural history data came from the ADVANCE trial of

peginterferon beta-1a, the London Ontario (Canada) database, and a large

population-based MS survey in the UK. The comparative efficacy of each DMT vs

placebo was obtained from a network meta-analysis. Costs (2015 British Pounds)

were obtained from public databases and literature. Clinical and economic

outcomes were projected over 30 years and discounted at 3.5% per year.

RESULTS: Over 30 years, peginterferon beta-1a was dominant compared with

interferon beta-1a (22, 30, and 44 mcg), and interferon beta-1b, and

cost-effective compared with glatiramer acetate 20 mg. Results were most

sensitive to variations in each DMT's efficacy and acquisition costs.

Deterministic and probabilistic sensitivity analyses confirmed the robustness of

the results.

LIMITATIONS: The impact of improved adherence with peginterferon beta-1a on

clinical and economic outcomes and the impact of subsequent DMTs after treatment

discontinuation were not considered. Oral and infused DMTs were not included as

comparators. Conclusion Long-term treatment with peginterferon beta-1a improves

clinical outcomes, while its cost profile makes it either dominant or

cost-effective compared with other self-injectable DMTs for the treatment of RRMS

in Scotland.

DOI: 10.1080/13696998.2016.1247712

PMID: 27730845 [Indexed for MEDLINE]

Excluded b/c no primary data (systematic review)

74. Neurourol Urodyn. 2017 Mar;36(3):557-564. doi: 10.1002/nau.23025. Epub 2016 May

17.

Outcomes of intra-detrusor injections of botulinum toxin in patients with spina

bifida: A systematic review.

Hascoet J(1), Manunta A(2)(3), Brochard C(3)(4)(5), Arnaud A(1), Damphousse

M(3)(6), Menard H(3), Kerdraon J(3)(7), Journel H(3), Bonan I(3)(6), Odent

S(3)(8), Fremond B(1)(3), Siproudhis L(3)(4)(5), Gamé X(9), Peyronnet B(2)(3)(5);

French Referral Network of Spina Bifida.

Author information:

(1)Service de chirurgie pédiatrique, CHU Rennes, Rennes, France.

(2)Service d'urologie, CHU Rennes, Rennes, France.

(3)Centre de référence spina bifida, CHU Rennes, Rennes, France.

(4)Service de Gastro-Entérologie, CHU Rennes, Rennes, France.

(5)Equipe thématique INPHY CIC 1414 et INSERM UMR 991, CHU Rennes, Rennes,

France.

(6)Service de médecine physique et réadaptation, CHU Rennes, Rennes, France.

(7)Centre de rééducation de Kerpape, Ploemeur, France.

(8)Service de génétique, CHU Rennes, Rennes, France.

(9)Département d'Urologie, Transplantation Rénale et Andrologie, CHU Rangueil,

Toulouse, France.

CONTEXT: Bladder management in spina bifida patients relies on clean intermittent

catheterization and oral antimuscarinics with a significant failure rate. The

efficacy of intradetrusor injections of botulinum toxin has been confirmed in

patients with spinal cord injury or multiple sclerosis but not in patients with

myelomeningocele.

OBJECTIVE: To conduct a systematic review of current evidence regarding the

efficacy of intra-detrusor injections of Botulinum Toxin A (BTX-A) in spina

bifida patients with neurogenic detrusor overactivity (NDO) refractory to

antimuscarinics.

METHODS: A research has been conducted on Medline and Embase using the keywords:

("spina bifida" OR "myelomeningocele" OR "dysraphism") AND "toxin." The search

strategy and studies selection were performed using the PICOS method according to

the PRISMA statement.

RESULT: Twelve published series were included (n = 293 patients). All patients

were <18 years old. There was no randomized study comparing BTX-A versus placebo

and most studies had no control group. Most studies reported a clinical and

urodynamic improvement with resolution of incontinence in 32-100% of patients, a

decrease in maximum detrusor pressure from 32 to 54%, an increase of maximum

cystometric capacity from 27 to 162%, and an improvement in bladder compliance of

28-176%. Two studies suggested lower efficacy in patients with low compliance

bladder compared to those with isolated detrusor overactivity.

CONCLUSION: Intradetrusor injections of BTX-A could be effective in children with

spina bifida but this assumption is not supported by high level of evidence

studies. There is no data available in adult patients. Neurourol. Urodynam.

36:557-564, 2017. © 2016 Wiley Periodicals, Inc.

© 2016 Wiley Periodicals, Inc.

DOI: 10.1002/nau.23025

PMID: 27187872 [Indexed for MEDLINE]

75. Neurology. 2017 Feb 7;88(6):525-532. doi: 10.1212/WNL.0000000000003582. Epub 2017

Jan 11.

Excluded b/c no real-world adherence/persistence

Disease-modifying therapies modulate retinal atrophy in multiple sclerosis: A

retrospective study.

Button J(1), Al-Louzi O(1), Lang A(1), Bhargava P(1), Newsome SD(1), Frohman

T(1), Balcer LJ(1), Frohman EM(1), Prince J(1), Calabresi PA(1), Saidha S(2).

Author information:

(1)From the Departments of Neurology (J.B., O.A.-L., P.B., S.D.N., P.A.C., S.S.)

and Electrical and Computer Engineering (A.L., J.P.), Johns Hopkins University,

Baltimore, MD; Department of Internal Medicine (O.A.-L.), North Shore Medical

Center, Salem, MA; Department of Neurology and Ophthalmology (T.F., E.M.F.),

University of Texas Southwestern, Dallas; and Department of Neurology (L.J.B.),

New York University Langone Medical Center, New York.

(2)From the Departments of Neurology (J.B., O.A.-L., P.B., S.D.N., P.A.C., S.S.)

and Electrical and Computer Engineering (A.L., J.P.), Johns Hopkins University,

Baltimore, MD; Department of Internal Medicine (O.A.-L.), North Shore Medical

Center, Salem, MA; Department of Neurology and Ophthalmology (T.F., E.M.F.),

University of Texas Southwestern, Dallas; and Department of Neurology (L.J.B.),

New York University Langone Medical Center, New York. ssaidha2@jhmi.edu.

OBJECTIVE: To retrospectively investigate whether disease-modifying therapies

(DMTs) exert differential effects on rates of retinal atrophy in

relapsing-remitting multiple sclerosis (RRMS), as assessed using optical

coherence tomography (OCT).

METHODS: A total of 402 patients with RRMS followed at the Johns Hopkins MS

Center who underwent Cirrus-HD OCT were assessed for eligibility. Inclusion

criteria included at least 1 year of OCT follow-up and adherence to a single DMT

during the period of follow-up. Combined thickness of the ganglion cell + inner

plexiform (GCIP) and other retinal layers was computed utilizing automated

macular segmentation. Retinal thickness changes were analyzed using mixed-effects

linear regression.

RESULTS: The effects of glatiramer acetate (GA; n = 48), natalizumab (NAT; n =

46), and interferon-β-1a subcutaneously (IFNSC; n = 35) and intramuscularly

(IFNIM; n = 28) were assessed. Baseline analyses revealed no significant

differences between groups in terms of age, sex, optic neuritis history, or

follow-up duration. During follow-up, relative to NAT-treated patients, IFNSC-

and GA-treated patients exhibited 0.37 μm/y (p < 0.001) and 0.14 μm/y (p = 0.035)

faster rates of GCIP thinning, respectively, adjusting for the interval between

initiation of DMT and OCT monitoring (gap time), age, sex, relapses, and disease

duration. In the IFNSC group, GCIP thinning was 1.53 μm/y faster during the first

year of therapy vs during the time interval afterwards (p < 0.001).

CONCLUSIONS: Rates of GCIP atrophy in patients with RRMS vary according to DMT

utilization. Our findings support OCT for monitoring neurodegenerative treatment

effects in the retina, an easily accessible tissue, and as a practical outcome

measure in RRMS clinical trials.

© 2017 American Academy of Neurology.

DOI: 10.1212/WNL.0000000000003582

PMCID: PMC5304463

PMID: 28077493 [Indexed for MEDLINE]

Excluded b/c no primary data (narrative review)

76. Biomed Pharmacother. 2017 Feb;86:343-353. doi: 10.1016/j.biopha.2016.12.010. Epub

2016 Dec 21.

Multiple sclerosis: Therapeutic applications of advancing drug delivery systems.

Dolati S(1), Babaloo Z(2), Jadidi-Niaragh F(3), Ayromlou H(4), Sadreddini S(1),

Yousefi M(5).

Author information:

(1)Immunology Research Center, Tabriz University of Medical Sciences, Tabriz,

Iran; Drug Applied Research Center, Tabriz University of Medical Sciences,

Tabriz, Iran; Department of Immunology, Faculty of Medicine, Tabriz University of

Medical Sciences, Tabriz, Iran.

(2)Drug Applied Research Center, Tabriz University of Medical Sciences, Tabriz,

Iran; Department of Immunology, Faculty of Medicine, Tabriz University of Medical

Sciences, Tabriz, Iran.

(3)Drug Applied Research Center, Tabriz University of Medical Sciences, Tabriz,

Iran; Department of Immunology, Faculty of Medicine, Tabriz University of Medical

Sciences, Tabriz, Iran; Department of Immunology, School of Public Health, Tehran

University of Medical Sciences, Tehran, Iran.

(4)Department of Neurology, Faculty of Medicine, Tabriz University of Medical

Sciences, Tabriz, Iran.

(5)Drug Applied Research Center, Tabriz University of Medical Sciences, Tabriz,

Iran; Department of Immunology, Faculty of Medicine, Tabriz University of Medical

Sciences, Tabriz, Iran. Electronic address: Yousefime@tbzmed.ac.ir.

Multiple sclerosis (MS) is an inflammatory autoimmune disease of the central

nervous system, which is accompanying with demyelination, neurodegeneration and

sensibility to oxidative stress. In MS, auto-reactive lymphocytes cross the

blood-brain barrier (BBB) and reside in the perivenous demyelinating lesions

which create various distinct inflammatory demyelinated plaques situated

predominantly in the white matter. The current MS-related therapeutic approaches

can be classified into disease-modifying therapies (DMTs) and symptomatic

therapy. DMTs suppress circulating immune cells, inhibit passing the BBB and

decrease the inflammatory responses. Recent advances have remarkably delayed

disease development and improved the quality of life for numerous patients. In

spite of major improvements in therapeutic options, there are some limitations

regarding the routes of administration and the necessity for repeated and

long-term dosing in which cause to systemic disadvantageous consequences and

patient non-compliance. Nanotechnology presents promising approaches to improve

autoimmune disease treatment with the capability to overcome many of the

limitations common to the current immunosuppressive and biological therapies.

Here we emphasis on nanomedicine-based drug delivery approaches of biological

immunomodulatory mediators for the treatment of multiple sclerosis. This

comprehensive review details the most successful drugs in MS therapy and also

focuses on conceptions and clinical potential of novel nanomedicine attitudes for

inducing immunosuppression and immunological tolerance in MS to modulate abnormal

and pathologic immune responses.

Copyright © 2016 Elsevier Masson SAS. All rights reserved.

DOI: 10.1016/j.biopha.2016.12.010

PMID: 28011382 [Indexed for MEDLINE]

77. Clin Drug Investig. 2017 Feb;37(2):175-186. doi: 10.1007/s40261-016-0471-2.

Excluded b/c no real-world adherence/persistence

Real-World Outcomes in Fingolimod-Treated Patients with Multiple Sclerosis in the

Czech Republic: Results from the 12-Month GOLEMS Study.

Tichá V(1), Kodým R(2), Počíková Z(2), Kadlecová P(3).

Author information:

(1)MS Center, Department of Neurology and Center of Clinical Neuroscience, First

Faculty of Medicine and General University Hospital in Prague, Charles

University, Katerinska 30, 120 00, Prague, Czech Republic. vticha@post.cz.

(2)Novartis s.r.o., Na Pankraci 1724/129, 14000, Prague, Czech Republic.

(3)Aprova s.r.o., Brno, Czech Republic.

BACKGROUND AND OBJECTIVE: Once-daily oral fingolimod is approved in the EU as

escalation treatment for adult patients with highly active relapsing multiple

sclerosis (MS). The efficacy and safety profiles of fingolimod have been well

established in a large clinical development programme and several papers

reflecting the experience with fingolimod in real-world settings have been

published to date. The GOLEMS study was designed to evaluate the efficacy, safety

and tolerability of fingolimod and the impact of fingolimod treatment on

disability progression and work capability in patients with MS in routine

clinical practice in the Czech Republic.

METHODS: GOLEMS was a national, multicentre, non-interventional, single-arm study

conducted to analyse the outcomes of a minimum of 12 months of fingolimod therapy

on primary and secondary endpoints. The primary endpoint was to assess the

proportion of relapse-free patients and severity of MS relapses in patients

treated with fingolimod for 12 months. Secondary endpoints included assessment of

changes in disability progression evaluated by the Expanded Disability Status

Scale (EDSS) score and work capability assessment measured through voluntary

completion of the WPAI-GH questionnaire. The predictive factors for relapse-free

status during fingolimod treatment were also analysed.

RESULTS: Of the 240 enrolled patients, 219 completed the 12-month treatment

period at the time of final analysis. In the efficacy set (N = 237), the

proportion of relapse-free patients increased from 47 patients (19.6 %; 95 %

confidence interval [CI] 14.8-25.2) in the year before fingolimod initiation to

152 patients (64.1 %; 95 % CI 58.0-70.2) after 1 year of fingolimod treatment. Of

the 85 patients who experienced at least one relapse after 1 year of fingolimod

treatment, 53 (62.4 %; 95 % CI 51.7-71.9) reported only one relapse, while 25

(29.4 %; 95 % CI 20.8-39.8) and seven (8.2 %; 95 % CI 4.0-16.0) patients had ≥2

relapses, respectively. No significant changes were observed in EDSS scores over

the 12-month treatment period compared with baseline. The absolute number of

relapses during 2 years before initiation of fingolimod treatment and baseline

EDSS scores were identified as significant independent predictors for 'being

relapse-free' during the 12-month fingolimod treatment period. No trend was

established in work capability or number of missed days at work due to the large

proportion of missing data. Of 240 enrolled patients, 27 (11.3 %) patients

discontinued the study at or before the 12-month visit, 16 (6.7 %) discontinued

because of adverse events related to study drug. Only six (2.5 %) patients

reported serious adverse events related to the study drug.

CONCLUSION: The results confirm the favourable safety and efficacy profile of

fingolimod under real-world conditions, consistent with phase III trials.

DOI: 10.1007/s40261-016-0471-2

PMCID: PMC5250638

PMID: 27785735 [Indexed for MEDLINE]

Conflict of interest statement: Compliance with Ethical Standards Funding This

study was funded by Novartis s.r.o. Conflict of interest Veronika Tichá has

received financial support for conference travel, consultant fees and speaker

honoraria from Biogen Idec, Novartis, Merck Serono, Teva, Actelion and Receptos.

Veronika Tichá is an employee of MS Center, Department of Neurology and Center of

Clinical Neuroscience, Charles University in Prague, 1st Faculty of Medicine and

General University Hospital in Prague. Roman Kodým and Zuzana Počíková are

employees of Novartis Pharma Czech Republic. Pavla Kadlecová is an employee of

Aprova s.r.o. (contract research organisation). Ethical statement The GOLEMS

study was approved by the Ethics Committee of the General University Hospital,

Prague, CR. Data in this project were collected via a system of electronic data

capture OpenClinica®, a web-based software which supports Good Clinical Practice

(GCP), regulatory guidelines such as 21 CFR Part 11, and is built on a modern

architecture using leading open standards. Informed consent was obtained from all

individual participants included in the study.

78. Eye (Lond). 2017 Feb;31(2):232-240. doi: 10.1038/eye.2016.258. Epub 2016 Nov 25.

Excluded b/c no primary data (narrative review)

Fingolimod: therapeutic mechanisms and ocular adverse effects.

Mandal P(1), Gupta A(1), Fusi-Rubiano W(1), Keane PA(2)(3), Yang Y(1)(4).

Author information:

(1)Department of Ophthalmology, The Royal Wolverhampton NHS Trust, New Cross

Hospital, Wolverhampton, UK.

(2)NIHR Biomedical Research Centre for Ophthalmology, Moorfields Eye Hospital NHS

Foundation Trust, London, UK.

(3)Institute of Ophthalmology, University College London, London, UK.

(4)School of Life & Health Sciences, Aston University, Birmingham, UK.

Fingolimod is an oral immunomodulating drug used in the management of

relapsing-remitting multiple sclerosis (RRMS). We aim to review the published

literature on ocular manifestations of fingolimod therapy and their possible

underlying mechanisms. The therapeutic effects of fingolimod are mediated via

sphingosine receptors, which are found ubiquitously in various organs, including

lymphoid cells, central nervous system, cardiac myocytes, and smooth muscle

cells. Fingolimod-associated macular oedema (FAME) is the most common ocular side

effect but retinal haemorrhages and retinal vein occlusion can occur. The visual

consequences appear to be mild and, in cases of FAME, resolution is often

attained with discontinuation of therapy. However, in cases of retinal vein

occlusion, discontinuation of fingolimod alone may not be sufficient and

intra-vitreal therapy may be required. We also propose a pragmatic service

pathway for monitoring patients on fingolimod therapy, which includes stratifying

them by risk and visual acuity.

DOI: 10.1038/eye.2016.258

PMCID: PMC5306460

PMID: 27886183 [Indexed for MEDLINE]

79. J Neurol. 2017 Feb;264(2):304-315. doi: 10.1007/s00415-016-8341-7. Epub 2016 Nov

25.

Excluded b/c no real-world adherence/persistence

Lesion remyelinating activity of GSK239512 versus placebo in patients with

relapsing-remitting multiple sclerosis: a randomised, single-blind, phase II

study.

Schwartzbach CJ(1), Grove RA(2), Brown R(3), Tompson D(4), Then Bergh F(5),

Arnold DL(3)(6).

Author information:

(1)GSK, Research Triangle Park, Raleigh-Durham, NC, USA.

Schwartzbach@mindspring.com.

(2)GSK, Stockley Park, Uxbridge, UK.

(3)McGill University, Montreal, QC, Canada.

(4)GSK, Gunnels Wood Road, Stevenage, Hertfordshire, UK.

(5)University of Leipzig, Leipzig, Germany.

(6)Department of Neurology, NeuroRx Research, Montreal, QC, Canada.

Histamine H3 receptor blockade may enhance lesion remyelination in multiple

sclerosis (MS). The efficacy (using a magnetic resonance imaging marker of

myelination, magnetisation transfer ratio [MTR]), safety and pharmacokinetics of

GSK239512, a potent and brain penetrant H3 receptor antagonist/inverse agonist on

lesion remyelination in relapsing-remitting MS (RRMS) were assessed. This was a

phase II, randomised, parallel-group, placebo-controlled, double-blind

(sponsor-unblinded), international, multicentre study (NCT01772199). Patients

aged 18-50 with RRMS, receiving intramuscular interferon-β1a or glatiramer

acetate, were randomised 1:1 to once-daily oral GSK239512 or placebo, up-titrated

over 4-5 weeks to a maximum tolerable dose up to 80 µg and maintained until Week

48. The co-primary endpoints were mean changes in post-lesion MTR in

gadolinium-enhanced (GdE) or Delta-MTR defined lesions from pre-lesion values.

Adverse events (AE) and withdrawals were monitored. Of the 131 patients

randomised, 114 patients completed the study (GSK239512, n = 51; placebo, n = 63)

and 27 (GSK239512) and 28 (placebo) patients contributed lesions to the primary

analysis. GSK239512 was associated with positive effect sizes of 0.344 [90%

confidence interval (CI) 0.018, 0.671] and 0.243 (90% CI -0.112, 0.598) for

adjusted mean changes in the normalised MTR for GdE and Delta-MTR lesions,

respectively. The overall incidence of AEs was similar between GSK239512 and

placebo during the treatment phase although some AEs including insomnia were more

common with GSK239512, particularly during the titration period. A small but

positive effect of GSK239512 on remyelination was observed. MTR assessment

represents a promising method for detecting lesion remyelination in RRMS.

DOI: 10.1007/s00415-016-8341-7

PMCID: PMC5306088

PMID: 27888416 [Indexed for MEDLINE]

Conflict of interest statement: Compliance with ethical standards Conflicts of

interest CJS is a former employee of GSK and holds stocks/shares in GSK. RAG and

DT are employees of GSK and hold stocks/shares in GSK; RB has received personal

compensation from NeuroRx Research for consulting services. FTB has received an

honorarium from GSK for speaking at an investigators´ meeting for the trial

reported here; his institution received compensation from GSK for the treatment

and documentation of those patients included at his site; he has received

personal compensation for speaking or serving on advisory boards from

Bayer-Schering, Biogen, Genzyme, Novartis and Teva; through his institution, he

received grants from Actelion, Bayer, Novartis and Teva. DLA has an equity

interest in NeuroRx Research, has received personal fees from Biogen, EMD Serono,

Genentech, Genzyme, Hoffman LaRoche, Innate Immunotherapy, MedImmune, Mitsubishi,

Novartis, Receptos, Acorda, Sanofi-Aventis, and Teva, and Grants from Biogen and

Novartis. Ethical standards The study was conducted in accordance with the

International Conference on Harmonisation of Technical Requirements for

Registration of Pharmaceuticals for Human Use, Good Clinical Practice (ICH-GCP)

and the ethical principles outlined in the Declaration of Helsinki 2008 [17].

Ethics approval was obtained from respective countries’ Ethics Committees (Online

resource 1). The study protocol is available online at

http://www.gsk-clinicalstudyregister.com/files2/gsk-116477-protocol-redact.pdf.

80. Mult Scler Relat Disord. 2017 Feb;12:82-87. doi: 10.1016/j.msard.2017.01.009.

Excluded b/c no real-world adherence/persistence

Epub 2017 Jan 24.

Discontinuation of disease modifying treatments in middle aged multiple sclerosis

patients. First line drugs vs natalizumab.

Fagius J(1), Feresiadou A(2), Larsson EM(3), Burman J(2).

Author information:

(1)Department of Neuroscience, Uppsala University, Uppsala, Sweden. Electronic

address: jan.fagius@neuro.uu.se.

(2)Department of Neuroscience, Uppsala University, Uppsala, Sweden.

(3)Department of Surgical Sciences/Radiology, Uppsala University, Uppsala,

Sweden.

BACKGROUND: Several disease-modifying drugs (DMD) are available for the treatment

of MS, and most patients with relapsing-remitting disease are currently treated.

Data on when and how DMD treatment can be safely discontinued are scarce.

METHODS: Fifteen MS patients, treated with natalizumab for >5 years without

clinical and radiological signs of inflammatory disease activity, suspended

treatment and were monitored with MRI examinations and clinical follow-up to

determine recurrence of disease activity. This group was compared with a

retrospectively analysed cohort comprising 55 MS patients treated with first-line

DMDs discontinuing therapy in the time period of 1998-2015 after an analogous

stable course.

RESULTS: Natalizumab discontinuers were followed for on average 19 months, and

follow-up data for 56 months were available for first-line DMD quitters.

Two-thirds of natalizumab treated patients experienced recurrent inflammatory

disease activity, and one third had recurrence of rebound character. In contrast,

35% of first-line DMD quitters had mild recurrent disease activity, and no one

exhibited rebound.

CONCLUSIONS: Withdrawal of a first-line DMD after prolonged treatment in

middle-aged MS patients with stable disease appears to be relatively safe, while

natalizumab withdrawal in a similar group of patients cannot be safely done

without starting alternative therapy.

Copyright © 2017 Elsevier B.V. All rights reserved.

DOI: 10.1016/j.msard.2017.01.009

PMID: 28283113 [Indexed for MEDLINE]

81. Patient Prefer Adherence. 2017 Jan 31;11:175-180. doi: 10.2147/PPA.S124192.

Excluded b/c no real-world adherence/persistence

eCollection 2017.

Herding: a new phenomenon affecting medical decision-making in multiple sclerosis

care? Lessons learned from DIScUTIR MS.

Saposnik G(1), Maurino J(2), Sempere AP(3), Ruff CC(4), Tobler PN(4).

Author information:

(1)Division of Neurology, Department of Medicine, St Michael's Hospital,

University of Toronto, Toronto, ON, Canada; Laboratory for Social and Neural

Systems Research, Department of Economics, University of Zurich, Zurich,

Switzerland; Li Ka Shing Knowledge Institute, St Michael's Hospital, University

of Toronto, Toronto, ON, Canada.

(2)Neuroscience Area, Medical Department, Roche Farma, Madrid.

(3)Department of Neurology, Hospital General Universitario de Alicante, Alicante,

Spain.

(4)Laboratory for Social and Neural Systems Research, Department of Economics,

University of Zurich, Zurich, Switzerland.

PURPOSE: Herding is a phenomenon by which individuals follow the behavior of

others rather than deciding independently on the basis of their own private

information. A herding-like phenomenon can occur in multiple sclerosis (MS) when

a neurologist follows a therapeutic recommendation by a colleague even though it

is not supported by best practice clinical guidelines. Limited information is

currently available on the role of herding in medical care. The objective of this

study was to determine the prevalence (and its associated factors) of herding in

the management of MS.

METHODS: We conducted a study among neurologists with expertise in MS care

throughout Spain. Participants answered questions regarding the management of 20

case scenarios commonly encountered in clinical practice and completed 3 surveys

and 4 experimental paradigms based on behavioral economics. The herding

experiment consisted of a case scenario of a 40-year-old woman who has been

stable for 3 years on subcutaneous interferon and developed a self-limited

neurological event. There were no new magnetic resonance imaging (MRI) lesions.

Her neurological examination and disability scores were unchanged. She was

advised by an MS neurologist to switch from interferon to fingolimod against best

practice guidelines. Multivariable logistic regression analysis was conducted to

evaluate factors associated with herding.

RESULTS: Out of 161 neurologists who were invited to participate, 96 completed

the study (response rate: 60%). Herding was present in 75 (78.1%), having a

similar prevalence in MS experts and general neurologists (68.8% vs 82.8%;

P=0.12). In multivariate analyses, the number of MS patients seen per week was

positively associated with herding (odds ratio [OR] 1.08, 95% CI 1.01-1.14).

Conversely, physician's age, gender, years of practice, setting of practice, or

risk preferences were not associated with herding.

CONCLUSION: Herding was a common phenomenon affecting nearly 8 out of 10

neurologists caring for MS patients. Herding may affect medical decisions and

lead to poorer outcomes in the management of MS.

DOI: 10.2147/PPA.S124192

PMCID: PMC5293495

PMID: 28203061

Conflict of interest statement: Disclosure The study was sponsored by the

Sociedad Española de Neurologia (SEN) and funded by an operating grant from Roche

Farma Spain. The sponsors were not involved in the design, execution, analysis,

and interpretation or reporting of the results. Dr Gustavo Saposnik is supported

by the Distinguished Clinicians Scientist Award from HSFC. Dr Jorge Maurino is an

employee of Roche Farma Spain. Prof Philippe Tobler and Christian Ruff were

funded by the Swiss National Science Foundation (PNT: PP00P1_150739,

CRSII3_141965, and 00014_165884, CCR:105314_152891, CRSII3_141965, and

320030_143443). The authors report no other conflicts of interest in this work.

82. CNS Drugs. 2017 Jan;31(1):33-50. doi: 10.1007/s40263-016-0394-8.

Excluded b/c no primary data (narrative review)

Alemtuzumab Use in Clinical Practice: Recommendations from European Multiple

Sclerosis Experts.

Berger T(1), Elovaara I(2), Fredrikson S(3), McGuigan C(4), Moiola L(5), Myhr

KM(6), Oreja-Guevara C(7), Stoliarov I(8), Zettl UK(9).

Author information:

(1)Clinical Department of Neurology, Medical University of Innsbruck, Innsbruck,

Austria. thomas.berger@i-med.ac.at.

(2)Department of Neurology and Rehabilitation, University of Tampere Medical

School and Tampere University Hospital, Tampere, Finland.

(3)Department of Clinical Neuroscience, Karolinska Institute, Stockholm, Sweden.

(4)St Vincent's University Hospital, Dublin, Ireland.

(5)San Raffaele Scientific Institute, Milan, Italy.

(6)Haukeland University Hospital and University of Bergen, Bergen, Norway.

(7)Hospital Clínico San Carlos, Madrid, Spain.

(8)Institute of the Human Brain, Russian Academy of Sciences, St. Petersburg,

Russia.

(9)Department of Neurology, Neuroimmunological Section, University of Rostock,

Rostock, Germany.

Alemtuzumab (Lemtrada™) is a humanized monoclonal antibody approved in more than

50 countries. Within the European Union, alemtuzumab is indicated for the

treatment of adult patients with relapsing-remitting multiple sclerosis (RRMS)

with active disease defined by clinical or imaging features; in the USA, the

indication states that alemtuzumab should generally be reserved for the treatment

of patients with relapsing forms of multiple sclerosis who have had an inadequate

response to two or more disease-modifying therapies (DMTs). In clinical trials,

alemtuzumab demonstrated efficacy in treatment-naïve patients with active RRMS

and those relapsing on prior DMTs, with a consistent and manageable safety and

tolerability profile. The European Union indication provides physicians with

significant flexibility regarding treatment decisions, affording the opportunity

for individualized treatment. Thus, alemtuzumab may be an appropriate treatment

choice across a broad range of patients with RRMS, including, for example,

treatment-naïve patients with active disease, patients with highly active

disease, or for patients relapsing on prior DMTs. There are several

practicalities to consider when using alemtuzumab, including the unique dosing

regimen, administered via intravenous infusion on 5 consecutive days at baseline

and on 3 consecutive days 12 months later, and as-needed retreatment (3

consecutive days at least 12 months after the last course) in cases of disease

recurrence. Additionally, routine monthly monitoring is required for up to 48

months after the last infusion to promptly identify potentially serious

autoimmune adverse events. Given these considerations, it is beneficial to gain

insight into how alemtuzumab is being used in the real-world clinical setting.

Here, we report recommendations from European multiple sclerosis experts

regarding best practices for alemtuzumab treatment, including management of

adverse events and compliance with ongoing safety monitoring requirements.

DOI: 10.1007/s40263-016-0394-8

PMCID: PMC5225231

PMID: 27882532 [Indexed for MEDLINE]

Conflict of interest statement: Compliance with Ethical Standards Funding The

advisory board was funded by Sanofi Genzyme. All advisors received honoraria

payments for their participation. Open access fee was funded by the Medical

University of Innsbruck. Conflict of interest Thomas Berger has received

consulting fees or honorarium and payment for lectures including service on

speaker’s bureaus from Allergen, Bayer, Biogen, Genzyme, Merck, Novartis, Sanofi,

and Teva, and support for travel from Genzyme. Irina Elovaara has received

honoraria for participation in advisory boards and travel support from Genzyme.

Sten Fredrikson has received honoraria for lectures, educational activities, or

consultancy from Allergan, Bayer, Biogen, Genzyme, Merck, Novartis, Sanofi, and

Teva. Chris McGuigan (and/or department) has received research grants from

Biogen, Genzyme, Novartis, and Bayer, honoraria as a consultant for Biogen,

Genzyme, and Novartis, and travel support from Genzyme. Lucia Moiola has received

honoraria for speaking in a scientific meeting or advisory board from Biogen,

Sanofi-Genzyme, Teva, and Novartis. Kjell-Morten Myhr has received honoraria for

lecturing and participation in advisory boards or pharmaceutical

company-sponsored clinical trials, and travel support from Allergan, Almirall,

Bayer Schering, Biogen, Sanofi-Genzyme, Merck-Serono, Novartis, and Teva. Celia

Oreja-Guevara has received honoraria from Biogen, Sanofi-Genzyme, Bayer,

Novartis, Teva, and Merck-Serono. Igor Stoliarov has no conflicts of interest.

Uwe Zettl has received speaker honoraria, travel support, and research support

from Almirall, Bayer, Biogen, Genzyme, Merck Serono, Novartis, and Sanofi

Aventis.

83. Eur Neurol. 2017;77(3-4):130-136. doi: 10.1159/000453333. Epub 2017 Jan 5.

Excluded b/c no real-world adherence/persistence

Observational Study of Switching from Natalizumab to Immunomodulatory Drugs.

Villaverde-González R(1), Gracia Gil J, Pérez Sempere A, Millán Pascual J, Marín

Marín J, Carcelén Gadea M, Gabaldón Torres L, Moreno Escribano A, Candeliere

Merlicco A.

Author information:

(1)Department of Neurology, Complejo Hospitalario Universitario de Albacete,

Albacete, Spain.

OBJECTIVE: To determine the effect of disease-modifying drugs (DMDs) on disease

activity rebound in patients discontinuing natalizumab (NTZ).

METHODS: Twenty-one patients with relapsing-remitting multiple sclerosis (RRMS)

treated with NTZ for ≥1 year and who switched to DMDs (glatiramer acetate [GA] or

interferon) were followed up for 12 months in clinical practice. Clinical

outcomes after NTZ cessation were assessed every 3 months for 1 year and MRI was

performed at 12 months.

RESULTS: Twelve months after switching from NTZ to DMDs, there were no

significant differences in the annualized relapse rate (ARR) compared to the days

that NTZ was used (0.3 vs. 0.1; p = 0.083); and the ARR never reached similar

values to those prior to NTZ use (1.61; p < 0.001). The percentage of

relapse-free patients after switching from NTZ was 71.4%. These patients did not

have lower disease activity before NTZ compared with those with clinical relapses

(1.3 vs. 1.7; p = 0.302), but they had lower Expanded Disability Status Scale

scores (3.4 vs. 5.7; p = 0.001). DMDs had beneficial effects on MRI parameters,

as 10 of 16 patients (62.5%) presented no evidence of radiological activity 12

months after NTZ discontinuation.

CONCLUSIONS: Patients with RRMS and moderate disability who discontinued NTZ for

safety reasons may benefit from the DMDs GA and interferon with no known risk for

progressive multifocal leukoencephalopathy.

© 2017 S. Karger AG, Basel.

DOI: 10.1159/000453333

PMID: 28052269 [Indexed for MEDLINE]

84. Mult Scler Relat Disord. 2017 Jan;11:56-61. doi: 10.1016/j.msard.2016.12.002.

Excluded b/c no real-world adherence/persistence

Epub 2016 Dec 8.

The impact of betaplus program on patient treatment satisfaction with interferon

beta-1b in multiple sclerosis: Multicentric cross-sectional survey in the western

Balkan countries.

Drulovic J(1), Cukic M(2), Grgic S(3), Dincic E(4), Raicevic R(4), Nadj C(5),

Toncev G(6), Vojinovic S(7), Mesaros S(8), Kisic Tepavcevic D(9), Dujmovic I(8),

Tadic D(3), Miletic-Drakulic S(6), Dackovic J(8), Kostic S(4), Erakovic J(2),

Sakalas L(5), Savic D(7), Suknjaja V(5), Martinovic V(8), Maric G(9), Pekmezovic

T(9).

Author information:

(1)Clinic of Neurology, Clinical Center of Serbia, Faculty of Medicine,

University of Belgrade, Dr Subotica 6, Belgrade 11000, Serbia. Electronic

address: jelena60@eunet.rs.

(2)Clinic of Neurology, Clinical Center of Montenegro, Ljubljanska bb, Podgorica

81000, Montenegro.

(3)Clinic of Neurology, Clinical Center of Banja Luka, Dvanaest beba, Banja Luka

78000, Republika Srpska, Bosnia and Herzegovina.

(4)Clinic of Neurology, Military Medical Academy, Crnotravska 17, Belgrade 11000,

Serbia.

(5)Clinic of Neurology, Clinical Center of Vojvodina, Hajduk Veljkova 1-9, Novi

Sad 21000, Serbia.

(6)Clinic of Neurology, Clinical Center Kragujevac, Zmaj Jovina 30, Kragujevac

34000, Serbia.

(7)Clinic of Neurology, Clinical Center Nis, Bulevar Dr Zorana Djindjica 48, Nis

18000, Serbia.

(8)Clinic of Neurology, Clinical Center of Serbia, Faculty of Medicine,

University of Belgrade, Dr Subotica 6, Belgrade 11000, Serbia.

(9)Institute of Epidemiology, Faculty of Medicine, University of Belgrade,

Visegradska 26A, Belgrade 11000, Serbia.

BACKGROUND: Long-term treatment adherence to disease-modifying drugs (DMDs) may

have significant impact on clinical outcomes in multiple sclerosis (MS). It has

been recently emphasized that low treatment satisfaction (TS) may be an important

factor for achieving high rates of treatment adherence. Interferon (IFN) beta-1b

was the first DMD approved for the treatment of MS. The aims of our study were to

assess TS in subjects with relapsing-remitting (RR) MS treated with IFN beta-1b

in Serbia, Montenegro and the Republika Srpska, Bosnia and Herzegovina (B&H), and

additionally, to evaluate the impact of patient support program on TS and

adherence.

METHODS: This is a cross-sectional survey performed in order to examine TS and

adherence with IFN beta-1b in seven MS centers across three countries (Serbia,

Montenegro and B&H). Included in the study were 296 adult patients with RRMS

treated with IFN beta-1b for at least 6 months. They were invited to complete the

Treatment Satisfaction Questionnaire for Medication (TSQM). Additional two

treatment adherence questions were also asked. Patient support program

(Betaplus®) was available exclusively for patients in Serbia and not for those in

Montenegro and the Republika Srpska, B&H. In order to assess the potential impact

of this program on TSQM, we combined two groups of patients from Montenegro and

B&H and compared their results with those from patients in Serbia. Statistical

analysis includes multivariable linear regression analysis in order to assess the

differences between three MS patients groups in terms of the TSQM scores,

adjusted for potential confounders. For the evaluation of the effects of

Betaplus® program, multivariable logistic regression was used, controlling for

the same confounding factors.

RESULTS: Each of the TSQM summary scores in all three countries implicated high

level of patients' satisfaction. There was statistically significant group

difference on the Effectiveness summary score (p=0.001) and the Side effects

summary score (p=0.006) between the group of subjects from Serbia and the

combined group of subjects from Montenegro and B&H, in favor of the former

cohort. There was statistically significant group difference neither on the

Convenience summary score nor on the Overall satisfaction summary score. Results

of adjusted logistic regression analysis based on the availability of patient

support program (dependent variable) implicate that it had the most significant

impact on the Effectiveness summary score (p=0.008). According to the correlation

coefficients in the total patient cohort, all TSMQ summary scores except

Effectiveness significantly correlated with the decreased adherence (Side

effects: p=0.037; Convenience: p=0.016; Overall satisfaction: p=0.046).

CONCLUSION: TS with IFN beta-1b was high in our MS patients. Additionally, these

results have demonstrated that patient support program have significant impact on

TS with IFN beta-1b in the Balkan cohort of RRMS patients.

Copyright © 2016 Elsevier B.V. All rights reserved.

DOI: 10.1016/j.msard.2016.12.002

PMID: 28104258 [Indexed for MEDLINE]

85. Neuroepidemiology. 2017;48(3-4):124-130. doi: 10.1159/000477771. Epub 2017 Jul 7.

Excluded b/c no oral DMD results

Disease-Modifying Therapies and Adherence in Multiple Sclerosis: Comparing

Patient Self-Report with Pharmacy Records.

McKay KA(1), Evans C, Fisk JD, Patten SB, Fiest K, Marrie RA, Tremlett H.

Author information:

(1)Faculty of Medicine (Neurology), Djavad Mowafaghian Centre for Brain Health,

University of British Columbia, Vancouver, BC, Canada.

BACKGROUND: Self-report and pharmacy records are often used to measure adherence

rates to disease-modifying therapies (DMTs) in multiple sclerosis (MS), but

little is known about how the sources compare.

OBJECTIVE: Compare self-report and pharmacy records for assessing DMT use and

adherence rates.

METHODS: Demographic information, self-reported DMT use, and missed DMT doses in

the previous 30 days were obtained from consecutive MS patients attending an MS

clinic and linked to pharmacy records. A medication possession ratio (MPR) was

calculated using pharmacy records for the year before and after the visit; MPR

<80% defined nonadherence. Agreement between self-report and pharmacy records was

assessed using Cohen's kappa (κ).

RESULTS: Of 326 participants, 135 reported using an injectable DMT. There was

near-perfect and perfect agreement between self-report and pharmacy records for

DMT use (κ = 0.95; 95% CI 0.91-0.98) and DMT agent (κ = 1.00). Nonadherence was

estimated at 13% (17/128) from the 30-days self-report compared to 30% (34/113)

and 43% (53/123) in the year pre- and post-clinic visit from pharmacy records,

indicating moderate to fair agreement (year prior: κ = 0.41; 95% CI 0.22-0.59;

year post: κ = 0.22; 95% CI 0.09-0.36).

CONCLUSIONS: Patients self-reports closely reflected pharmacy records when

assessing DMT use and product. Moderate to fair agreement was found when

comparing adherence rates between sources.

© 2017 S. Karger AG, Basel.

DOI: 10.1159/000477771

PMID: 28683461

Excluded b/c no real-world adherence/persistence

86. Neurol Neurochir Pol. 2017 Mar - Apr;51(2):156-162. doi:

10.1016/j.pjnns.2017.01.006. Epub 2017 Feb 2.

Severe disease exacerbations in patients with multiple sclerosis after

discontinuing fingolimod.

Członkowska A(1), Smoliński Ł(2), Litwin T(2).

Author information:

(1)Second Department of Neurology, Institute of Psychiatry and Neurology, Warsaw,

Poland; Department of Clinical and Experimental Pharmacology, Medical University

of Warsaw, Warsaw, Poland. Electronic address: czlonkow@ipin.edu.pl.

(2)Second Department of Neurology, Institute of Psychiatry and Neurology, Warsaw,

Poland.

Discontinuation of fingolimod in patients with multiple sclerosis (MS) can lead

to disease reactivation. In this review, we describe cases of severe

exacerbations in patients with MS following discontinuation of fingolimod,

including three cases from our center. We consider potential mechanisms of

disease reactivation after cessation of fingolimod, and the evidence supporting

this rebound effect. We conclude that discontinuation of fingolimod results in

the return of disease activity, which then leads to severe exacerbations (i.e.,

rebounds) in a clinically significant proportion of patients. Lastly, we consider

disease-modifying treatment options for patients who discontinue fingolimod.

Copyright © 2017. Published by Elsevier Urban & Partner Sp. z o.o.

DOI: 10.1016/j.pjnns.2017.01.006

PMID: 28209440 [Indexed for MEDLINE]

Excluded b/c no real-world adherence/persistence

87. Perm J. 2017;21. doi: 10.7812/TPP/16-102.

Improving Patient-Centered Care by Assessing Patient Preferences for Multiple

Sclerosis Disease-Modifying Agents: A Stated-Choice Experiment.

Carlin CS(1), Higuera L(2), Anderson S(3).

Author information:

(1)Research Investigator for the Medica Research Institute in Minneapolis, MN.

caroline.carlin@medica.com.

(2)Research Associate for the Medica Research Institute in Minneapolis, MN.

lucas.higuera@medica.com.

(3)Pharmacist for Medica Pharmacy in Minneapolis, MN. sarah.anderson@medica.com.

CONTEXT: Long-term adherence to pharmaceutical treatment for multiple sclerosis

(MS) is poor. A focus on patient preferences when determining the patient's

therapeutic plan may improve this experience.

OBJECTIVE: To identify factors important to patients with MS when evaluating

their options for pharmaceutical agents that deliver disease-modifying therapy.

DESIGN: Stated-choice experiment to a sample of patients with MS from privately

and publicly insured enrollees in a regional health plan. The experiment

presented each respondent with a set of 8 drug choices for MS, asking them to

select their preferred disease-modifying agent (DMA). Each respondent was

randomized to 1 of 6 possible sets of 8 drug choices, for a total of 48 drug

pairings in the experiment. Each choice included 2 hypothetical DMAs and a "no

drug" option. Drug attributes included dosage type and modality, efficacy,

relapse risk, and drug side effects.

RESULTS: The "no drug" alternative was a stronger substitute than the alternative

drug when the focal drug characteristics changed, and the most important drivers

of choice were type of side effects and risk of severe relapse.

DISCUSSION: The heterogeneity of our sample and the inclusion of a "no drug"

alternative in the DMA choice scenarios make this study an important contribution

to this body of literature. The importance of the "no drug" alternative in our

results is consistent with poor long-term adherence to DMAs.

CONCLUSION: Patient-centered MS therapy using DMAs should include discussion of

side effects and relapse risk.

DOI: 10.7812/TPP/16-102

PMCID: PMC5391779

PMID: 28406788 [Indexed for MEDLINE]

88. Patient Prefer Adherence. 2016 Dec 30;11:55-62. doi: 10.2147/PPA.S118107.

eCollection 2017.

An evaluation of adherence in patients with multiple sclerosis newly initiating

treatment with a self-injectable or an oral disease-modifying drug.

Munsell M(1), Frean M(1), Menzin J(1), Phillips AL(2).

Author information:

(1)Boston Health Economics, Inc., Waltham, MA, USA.

(2)Health Economics & Outcomes Research, EMD Serono Inc., Rockland, MA, USA.

OBJECTIVE: As the multiple sclerosis (MS) disease-modifying drug (DMD) treatment

options have expanded to include oral therapies, it is important to understand

whether route of administration is associated with DMD adherence. The objective

of this study was to compare adherence to DMDs in patients with MS newly

initiating treatment with a self-injectable versus an oral DMD.

METHODS: This retrospective database study used IMS Health Real World Data

Adjudicated Claims - US data between July 1, 2010 and June 30, 2014. Adherence

was measured by medication possession ratio (MPR), calculated as the total number

of treated days divided by the total number of days from the first treated day

until the end of 12-month follow-up. A binary measure representing adherence (MPR

≥0.8) versus nonadherence (MPR <0.8) to therapy was used. Logistic regression

evaluated the likelihood of adherence to index DMD type (self-injectable vs

oral). Covariates included patient baseline characteristics (ie, age, sex,

comorbidities) and index DMD type.

RESULTS: The analysis included 7,207 self-injectable and 1,175 oral DMD-treated

patients with MS. In unadjusted analyses, the proportion of patients adherent to

therapy (MPR ≥0.8) did not differ significantly between the self-injectable

(54.1%) and the oral DMD cohorts (53.0%; P=0.5075). After controlling for

covariates, index DMD type was not a significant predictor of adherence (odds

ratio [OR] 1.062; 95% confidence interval [CI]: 0.937-1.202; P=0.3473). Higher

likelihood of adherence was associated with male sex (OR 1.20; 95% CI:

1.085-1.335; P=0.0005) and age groups older than 18-34 years (ORs 1.220-1.331;

P<0.01). Depression was associated with a lower likelihood of adherence (OR

0.618; 95% CI: 0.511-0.747; P<0.0001).

CONCLUSION: Male sex and age older than 18-34 years were significantly associated

with a higher likelihood of adherence, while depression was associated with a

lower likelihood of adherence. Index DMD type, stratified by the route of

administration (self-injectable vs oral DMD), was not a significant predictor of

DMD adherence.

DOI: 10.2147/PPA.S118107

PMCID: PMC5221550

PMID: 28115831

Conflict of interest statement: MM and JM are employees of Boston Health

Economics, Inc. MF is a former employee of Boston Health Economics, Inc. Boston

Health Economics, Inc., received funding from EMD Serono, Inc., Rockland, MA, USA

(a business of Merck KGaA, Darmstadt, Germany) to conduct the analyses. ALP is an

employee of EMD Serono, Inc., Rockland, MA, USA (a business of Merck KGaA,

Darmstadt, Germany). The authors received no funding for their participation in

the writing of the manuscript. The authors report no other conflicts of interest

in this work.

Excluded b/c no real-world adherence/persistence

89. Patient Prefer Adherence. 2016 Dec 22;11:33-45. doi: 10.2147/PPA.S115090.

eCollection 2017.

The state of multiple sclerosis: current insight into the patient/health care

provider relationship, treatment challenges, and satisfaction.

Tintoré M(1), Alexander M(2), Costello K(3), Duddy M(4), Jones DE(5), Law N(6),

O'Neill G(7), Uccelli A(8), Weissert R(9), Wray S(10).

Author information:

(1)Multiple Sclerosis Centre of Catalonia, Hospital Vall d'Hebron, Barcelona,

Spain.

(2)European Multiple Sclerosis Platform, Brussels, Belgium.

(3)National Multiple Sclerosis Society, Denver, CO, USA.

(4)Royal Victoria Infirmary, Newcastle-upon-Tyne, UK.

(5)Department of Neurology, University of Virginia, Charlottesville, VA, USA.

(6)Nancy Law Consulting LLC, Parker, CO, USA.

(7)Biogen, Cambridge, MA, USA.

(8)Centre of Excellence for Biomedical Research, University of Genoa, Genoa,

Italy.

(9)Department of Neurology, University of Regensburg, Regensburg, Germany.

(10)Hope Neurology Multiple Sclerosis Center, Knoxville, TN, USA.

BACKGROUND: Managing multiple sclerosis (MS) treatment presents challenges for

both patients and health care professionals. Effective communication between

patients with MS and their neurologist is important for improving clinical

outcomes and quality of life.

METHODS: A closed-ended online market research survey was used to assess the

current state of MS care from the perspective of both patients with MS (≥18 years

of age) and neurologists who treat MS from Europe and the US and to gain insight

into perceptions of treatment expectations/goals, treatment decisions, treatment

challenges, communication, and satisfaction with care, based on current clinical

practice.

RESULTS: A total of 900 neurologists and 982 patients completed the survey, of

whom 46% self-identified as having remitting-relapsing MS, 29% secondary

progressive MS, and 11% primary progressive MS. Overall, patients felt satisfied

with their disease-modifying therapy (DMT); satisfaction related to comfort in

speaking with their neurologist and participation in their DMT decision-making

process. Patients who self-identified as having relapsing-remitting MS were more

likely to be very satisfied with their treatment. Top challenges identified by

patients in managing their DMT were cost, side effects/tolerability of treatment,

and uncertainty if treatment was working. Half of the patients reported skipping

doses, but only 68% told their health care provider that they did so.

CONCLUSION: Several important differences in perception were identified between

patients and neurologists concerning treatment selection, satisfaction,

expectations, goals, and comfort discussing symptoms, as well as treatment

challenges and skipped doses. The study results emphasize that

patient/neurologist communication and patient input into the treatment

decision-making process likely influence patient satisfaction with treatment.

DOI: 10.2147/PPA.S115090

PMCID: PMC5189708

PMID: 28053511

Conflict of interest statement: Mar Tintoré received consulting and non-CME

service fees from Almirall, Biogen, EMD Merck Serono, Genzyme, Novartis, Roche,

Sanofi-Aventis, and Teva UK Limited; educational/research support from Bayer

HealthCare, Biogen, Genzyme, EMD Merck Serono, Novartis Pharma AG, and Teva UK

Limited. Martin Duddy received honoraria, educational support, consulting fees,

and research support from Bayer HealthCare, Biogen, Genzyme, Merck Serono,

Novartis Pharma AG, Roche, and Teva UK Limited; and personal compensation as

associate editor for the Multiple Sclerosis Journal. David E Jones received

consulting fees from Biogen, Genzyme, and Novartis; and research support from

Biogen. Nancy Law received consulting fees from Biogen in her current role; no

consulting fees were received in her former role as an employee of the National

Multiple Sclerosis Society (Denver, CO, USA). Gilmore O’Neill is a full-time

employee of and holds stock/stock options in Biogen. Antonio Uccelli received

consulting or speaker fees from Allergan, Bayer HealthCare, Biogen, Genzyme,

Merck Serono, Novartis, Roche, and Teva; and research support from Biogen, Merck

Serono, and Novartis. Robert Weissert received consulting fees from Biogen,

Genzyme, Merck Serono, Novartis, Roche, and Teva; served on speakers bureaus for

Ärztlicher Kreisverband Weiden and Biogen; and performed contracted research for

Novartis. Sibyl Wray received consulting fees from Acorda, Biogen, EMD Serono,

Genzyme, Novartis, Questcor, and Teva; served on speakers bureaus for Acorda,

Bayer HealthCare, Biogen, EMD Serono, Genzyme, Novartis, Questcor, and Teva; and

performed contracted research for Biogen, EMD Serono, Genzyme, Novartis,

Receptos, and Roche. The authors report no other conflicts of interest in this

work.

Excluded b/c no oral DMD results

90. J Manag Care Spec Pharm. 2016 Dec;22(12):1394-1401.

Adherence to Disease-Modifying Therapies for Multiple Sclerosis.

Higuera L(1), Carlin CS(1), Anderson S(2).

Author information:

(1)1 Medica Research Institute, Minnetonka, Minnesota.

(2)2 Medica Health Plans, Minnetonka, Minnesota.

BACKGROUND: Multiple sclerosis (MS) is a neurological degenerative chronic

condition without cure. However, long-term disease-modifying therapies (DMTs)

help reduce the severity of MS symptoms. Adherence to DMTs is key to their

success. Several studies have analyzed what makes patients adherent to their

DMTs. As new DMTs have entered the market, few studies have analyzed factors of

adherence using all currently available DMTs.

OBJECTIVE: To analyze different factors of adherence to DMTs for MS, in

particular how the type of DMT affects adherence.

METHODS: This retrospective cohort study used enrollment and claims data from an

upper Midwest health plan in the United States between 2011 and 2013. Patients

entered the study if they had any medical claim with an MS diagnosis and used

only 1 DMT during the study time frame. Medication possession ratios (MPRs) were

computed as the fraction of days with medication supplied during the year;

patients with MPRs of 0.8 or higher were considered adherent. Multivariate probit

models with patient-specific random effects were estimated, with controls for

demographic characteristics, type of DMT, health plan type, and measures of

health status.

RESULTS: Patients aged over 45 years were between 13.7 to 18.6 percentage points

more likely to be adherent than younger patients. Women had a 5.5

percentage-point lower probability of being adherent than men. Patients using

self-injectable DMTs with injection site reactions as the most likely side effect

were 9.1 percentage points less likely to be adherent than patients using oral,

infusible, and other self-injectable DMTs. Patients with depression had a 5.5

percentage-point lower probability of being adherent. These results were robust

to changes in controls for type of plan and neighborhood socioeconomic

characteristics.

CONCLUSIONS: This study found statistically significant differences in adherence

to DMTs by age, sex, type of DMT, and a depression diagnosis.

DISCLOSURES: TEVA provided funding for this study and had the option to review

the manuscript. The authors retained autonomy in the determination of the final

content of this work. Study concept and design were contributed by Carlin,

Anderson, and Higuera. Data interpretation was primarily performed by Higuera and

Carlin, along with Anderson. The manuscript was written and revised by Higuera,

Carlin, and Anderson.

DOI: 10.18553/jmcp.2016.22.12.1394

PMID: 27882830 [Indexed for MEDLINE]

Excluded b/c no real-world adherence/persistence

91. Mult Scler. 2016 Dec;22(14):1888-1890. Epub 2016 Apr 26.

Development of a primary cutaneous CD30(+) anaplastic large-cell T-cell lymphoma

during treatment of multiple sclerosis with fingolimod.

Papathemeli D(1), Gräfe R(2), Hildebrandt U(3), Zettl UK(4), Ulrich J(2).

Author information:

(1)Department of Dermatology and Venereology, Harzklinikum Dorothea Christiane

Erxleben, Quedlinburg, Germany dpapathem@live.com.

(2)Department of Dermatology and Venereology, Harzklinikum Dorothea Christiane

Erxleben, Quedlinburg, Germany.

(3)Department of Pathology, Harzklinikum Dorothea Christiane Erxleben,

Quedlinburg, Germany.

(4)Department of Neurology, University of Rostock, Rostock, Germany.

BACKGROUND: The appearance of solid tumors und lymphomas during treatment with

fingolimod was observed in studies and has been described in case reports.

OBJECTIVE: To report a case of primary cutaneous CD30(+) anaplastic large-cell

T-cell lymphoma during treatment of multiple sclerosis (MS) with fingolimod.

METHODS: Case study.

RESULTS: Our patient developed a lymphoma a few weeks after initialization of

therapy with fingolimod; 5 weeks after discontinuation of treatment the lesions

resolved.

CONCLUSION: Causality of fingolimod is indicated by the fact that the skin

lesions appeared after commencement of treatment and resolved after

discontinuation of therapy. This case serves as a reminder of the potential side

effects of fingolimod.

© The Author(s), 2016.

DOI: 10.1177/1352458516645868

PMID: 27207455 [Indexed for MEDLINE]

Excluded b/c no real-world adherence/persistence

92. Front Microbiol. 2016 Nov 29;7:1919. eCollection 2016.

Synthesis of Sphingolipids Impacts Survival of Porphyromonas gingivalis and the

Presentation of Surface Polysaccharides.

Moye ZD(1), Valiuskyte K(1), Dewhirst FE(2), Nichols FC(3), Davey ME(1).

Author information:

(1)Department of Oral Biology, College of Dentistry, University of Florida,

Gainesville FL, USA.

(2)Department of Microbiology, Forsyth Institute, CambridgeMA, USA; Department of

Oral Medicine, Infection and Immunity, Harvard School of Dental Medicine,

BostonMA, USA.

(3)Division of Periodontology, Department of Oral Health and Diagnostic Sciences,

School of Dental Medicine, University of Connecticut, Farmington CT, USA.

Bacteria alter the biophysical properties of their membrane lipids in response to

environmental cues, such as shifts in pH or temperature. In essence, lipid

composition determines membrane structure, which in turn influences many basic

functions, such as transport, secretion, and signaling. Like other members of the

phylum Bacteroidetes, the oral anaerobe Porphyromonas gingivalis possesses the

ability to synthesize a variety of novel membrane lipids, including species of

dihydroceramides that are distinct, yet similar in structure to sphingolipids

produced by the human host. The role of dihydroceramides in the physiology and

pathogenic potential of the human microbiota is only beginning to be explored;

yet there is increasing data indicating that these lipids play a role in human

diseases, such as periodontitis and multiple sclerosis. Here, we report on the

identification of a gene (PG1780) in the chromosome of P. gingivalis strain W83

encoding a putative serine palmitoyltransferase, the enzyme that catalyzes the

first step in sphingolipid biosynthesis. While we were able to detect

dihydroceramides in whole lipid extracts of P. gingivalis cells as well as crude

preparations of outer membrane vesicles, sphingolipids were absent in the PG1780

mutant strain. Moreover, we show that the synthesis of sphingolipids plays an

essential role in the long-term survival of the organism as well as its

resistance to oxidative stress. Further, a PG1780 mutant displayed much lower

activity of cell-associated arginine and lysine gingipains, yet slightly higher

activity in the corresponding culture supernates, which we hypothesize is due to

altered membrane properties and anchoring of these proteases to the cell surface.

In addition, we determined that sphingolipid production is critical to the

presentation of surface polysaccharides, with the mutant strain displaying less

K-antigen capsule and more anionic polysaccharide (APS). Overall, we have

discovered that, in addition to their role in pathogenicity, the synthesis of

sphingolipids is critical to the cellular homeostasis and persistence of this

important dental pathogen.

DOI: 10.3389/fmicb.2016.01919

PMCID: PMC5126122

PMID: 27965646

93. PLoS One. 2016 Nov 3;11(11):e0164862. doi: 10.1371/journal.pone.0164862.

Excluded b/c no real-world adherence/persistence

eCollection 2016.

Identification and Prioritization of Important Attributes of Disease-Modifying

Drugs in Decision Making among Patients with Multiple Sclerosis: A Nominal Group

Technique and Best-Worst Scaling.

Kremer IE(1), Evers SM(1)(2), Jongen PJ(3)(4), van der Weijden T(5), van de Kolk

I(6), Hiligsmann M(1).

Author information:

(1)Department of Health Services Research, CAPHRI School of Public Health and

Primary Care, Maastricht University, Maastricht, The Netherlands.

(2)Public Mental Health, Trimbos Institute, Netherlands Institute of Mental

Health and Addiction, Utrecht, The Netherlands.

(3)MS4 Research Institute, Nijmegen, The Netherlands.

(4)Department of Community & Occupational Medicine, University Medical Centre

Groningen, Groningen, The Netherlands.

(5)Department of Family Medicine, CAPHRI School of Public Health and Primary

Care, Maastricht University, Maastricht, The Netherlands.

(6)Department of Health Promotion, NUTRIM School of Nutrition and Translational

Research in Metabolism, Maastricht University, Maastricht, The Netherlands.

OBJECTIVES: Understanding the preferences of patients with multiple sclerosis

(MS) for disease-modifying drugs and involving these patients in clinical

decision making can improve the concordance between medical decisions and patient

values and may, subsequently, improve adherence to disease-modifying drugs. This

study aims first to identify which characteristics-or attributes-of

disease-modifying drugs influence patients´ decisions about these treatments and

second to quantify the attributes' relative importance among patients.

METHODS: First, three focus groups of relapsing-remitting MS patients were formed

to compile a preliminary list of attributes using a nominal group technique.

Based on this qualitative research, a survey with several choice tasks

(best-worst scaling) was developed to prioritize attributes, asking a larger

patient group to choose the most and least important attributes. The attributes'

mean relative importance scores (RIS) were calculated.

RESULTS: Nineteen patients reported 34 attributes during the focus groups and 185

patients evaluated the importance of the attributes in the survey. The effect on

disease progression received the highest RIS (RIS = 9.64, 95% confidence

interval: [9.48-9.81]), followed by quality of life (RIS = 9.21 [9.00-9.42]),

relapse rate (RIS = 7.76 [7.39-8.13]), severity of side effects (RIS = 7.63

[7.33-7.94]) and relapse severity (RIS = 7.39 [7.06-7.73]). Subgroup analyses

showed heterogeneity in preference of patients. For example, side effect-related

attributes were statistically more important for patients who had no experience

in using disease-modifying drugs compared to experienced patients (p < .001).

CONCLUSIONS: This study shows that, on average, patients valued effectiveness and

unwanted effects as most important. Clinicians should be aware of the average

preferences but also that attributes of disease-modifying drugs are valued

differently by different patients. Person-centred clinical decision making would

be needed and requires eliciting individual preferences.

DOI: 10.1371/journal.pone.0164862

PMCID: PMC5094791

PMID: 27812117 [Indexed for MEDLINE]

Conflict of interest statement: During the time of the study PJ Jongen has

received honoraria from Bayer, Merck, and Teva for contributions to symposia as a

speaker or for consultancy activities. This does not alter our adherence to PLOS

ONE policies on sharing data and materials. The other authors have declared that

no competing interest exists.

94. J Clin Neurosci. 2016 Nov;33:73-78. doi: 10.1016/j.jocn.2015.12.044. Epub 2016

Excluded b/c no oral DMD results

Aug 16.

Predictors of first-line treatment persistence in a Portuguese cohort of

relapsing-remitting multiple sclerosis.

Correia I(1), Marques IB(2), Sousa M(2), Batista S(2), Ferreira R(3), Nunes C(2),

Macário C(2), Cunha L(2), Sousa L(2).

Author information:

(1)Neurology Department, Centro Hospitalar e Universitário de Coimbra, Praceta

Prof. Mota Pinto, 3000-075 Coimbra, Portugal. Electronic address:

mcorreia.ines@gmail.com.

(2)Neurology Department, Centro Hospitalar e Universitário de Coimbra, Praceta

Prof. Mota Pinto, 3000-075 Coimbra, Portugal.

(3)Internal Medicine Department, Centro Hospitalar e Universitário de Coimbra,

Praceta Prof. Mota Pinto, 3000-075 Coimbra, Portugal.

Treatment persistence in first-line injectable disease-modifying therapies (DMT)

for relapsing-remitting multiple sclerosis (RRMS) is an important indicator of

effectiveness. Identifying predictors of treatment discontinuation is important

as there are other therapies currently available and a growing range of emerging

drugs. We report a retrospective study of RRMS and clinically isolated syndrome

patients followed in a University Hospital during a 13-year period with the

objective of identifying predictors of treatment persistence. An evaluation of

persistence on the first DMT, rates of DMT discontinuation, and reasons and

predictors of discontinuation was performed. A total of 410 patients were

included, 69% female, with mean disease duration of 37.8months, mean age of

34.2years and mean follow-up time of 6.1years. The first DMT was glatiramer

acetate (GA) in 27.56% of patients, interferon (IFN) β-1a intramuscular in

26.34%, IFNβ-1b in 26.10%, IFNβ-1a22 in 13.66% and IFNβ-1a44 in 6.34%. Treatment

was discontinued in 16.34% of patients after 1year of treatment and in 50.24% of

patients in the total follow-up time, with a mean time for discontinuation of

39.80months. Higher baseline Expanded Disability Status Scale score was an

independent predictor of treatment discontinuation (hazard ratio 1.35, p=0.002).

After the first year, treatment persistence was 90.74% for IFNβ-1a-IM, 88.46% for

IFNβ-1a44, 83.18% for IFNβ-1b, 83.19% for GA and 69.64% for IFNβ-1a22 (p=0.014).

Lower frequency of administration was associated with higher persistence rates.

The most common reason for treatment discontinuation was lack of efficacy in all

DMT subgroups.

Copyright © 2016 Elsevier Ltd. All rights reserved.

DOI: 10.1016/j.jocn.2015.12.044

PMID: 27542939 [Indexed for MEDLINE]

Excluded b/c no oral DMD results

95. Mult Scler Relat Disord. 2016 Nov;10:90-96. doi: 10.1016/j.msard.2016.09.011.

Epub 2016 Sep 28.

Predictors of long-term interferon discontinuation in newly diagnosed relapsing

multiple sclerosis.

Moccia M(1), Palladino R(2), Carotenuto A(3), Russo CV(3), Triassi M(4), Lanzillo

R(3), Brescia Morra V(3).

Author information:

(1)Multiple Sclerosis Clinical Care and Research Center, Department of

Neuroscience, Reproductive Science and Odontostomatology, Federico II University,

Naples, Italy. Electronic address: moccia.marcello@gmail.com.

(2)Department of Primary Care and Public Health, Imperial College, London, UK;

Department of Public Health, Federico II University, Naples, Italy.

(3)Multiple Sclerosis Clinical Care and Research Center, Department of

Neuroscience, Reproductive Science and Odontostomatology, Federico II University,

Naples, Italy.

(4)Department of Public Health, Federico II University, Naples, Italy.

BACKGROUND: Interferon-β has long-term safety and efficacy profiles for Relapsing

Remitting Multiple Sclerosis (RRMS). However, the increasing number of available

treatments requires to improve patient profiling and to perform individualized

clinical decisions. Therefore, the present study investigated predictors of

Interferon-β discontinuation.

METHODS: The present retrospective observational cohort study included 499 newly

diagnosed, drug naïve RRMS subjects receiving Interferon-β as first disease

modifying treatment (DMT), during a 7.9±3.8 year period, up to treatment

discontinuation. Possible markers of interest were recorded at the time of

diagnosis (age, gender, disease duration, baseline EDSS) or during follow-up as

variables of disease evolution (relapse occurrence, annualized relapse rate

-ARR-, 1-point EDSS progression, reaching of EDSS 4.0) or of treatment (high-dose

Interferon-β1a, low-dose Interferon-β1a, or Interferon-β1b).

RESULTS: 217 patients (43.5%) discontinued the treatment during the follow-up

period, with an incidence of 5% person-years (95%CI=4.6-5.9%). A multivariate Cox

regression model showed an increased rate of Interferon-β discontinuation for

female gender (p=0.019; HR=1.428), higher baseline EDSS (p=0.026; HR=1.346),

relapse occurrence (p=0.009; HR=1.618), higher ARR (p<0.001; HR=5.269), and

Interferon-β1b treatment (p=0.019; HR=1.506); and a reduced rate for occurrence

of EDSS progression (p<0.001; HR=0.299).

CONCLUSIONS: Most of the factors associated with Interferon-β discontinuation are

not modifiable, and are part of demographic features (i.e. gender), or of disease

characteristics (i.e. disability at diagnosis), but should be taken into account

when prescribing the first DMT for MS. Noteworthy, the use of Interferon-β1b is

associated with 50% increased risk of discontinuation, compared with high-dose

Interferon-β1a, highlighting the importance of drug formulations in treatment

persistence.

Copyright Â© 2016 Elsevier B.V. All rights reserved.

DOI: 10.1016/j.msard.2016.09.011

PMID: 27919507 [Indexed for MEDLINE]

96. Mult Scler Relat Disord. 2016 Nov;10:44-52. doi: 10.1016/j.msard.2016.08.002.

Epub 2016 Aug 8.

Comparative efficacy and discontinuation of dimethyl fumarate and fingolimod in

clinical practice at 12-month follow-up.

Hersh CM(1), Love TE(2), Cohn S(3), Hara-Cleaver C(4), Bermel RA(4), Fox RJ(4),

Cohen JA(4), Ontaneda D(4).

Author information:

(1)Lou Ruvo Center for Brain Health, Cleveland Clinic, 888 W. Bonneville Ave, Las

Vegas, NV, 89106 USA. Electronic address: hershc@ccf.org.

(2)Department of Epidemiology and Biostatistics, Case Western Reserve University,

10900 Euclid Ave, Cleveland, 44106 USA.

(3)Department of Neurology, Cleveland Clinic, 9500 Euclid Ave, Cleveland, OH,

44195 USA.

(4)Mellen Center for Multiple Sclerosis Treatment and Research, Cleveland Clinic,

9500 Euclid Ave, Cleveland, OH, 44195 USA.

BACKGROUND: Dimethyl fumarate (DMF) and fingolimod (FTY) are approved oral

disease modifying therapies (DMT) for relapsing multiple sclerosis (MS). Phase 3

trials established these agents as effective and generally well tolerated, though

comparative efficacy and discontinuation remain unknown.

OBJECTIVE: To assess real-world efficacy and discontinuation of DMF and FTY over

12 months in patients with MS.

METHODS: We identified 458 DMF-treated and 317 FTY-treated patients in a large

academic MS center. Measures of disease activity and discontinuation were

compared using propensity score (PS) weighting. Covariates in the PS model

included demographics and baseline clinical and MRI characteristics within 12

months of DMT initiation. The primary outcome measure was on-treatment annualized

relapse rate (ARR) ratio, which was analyzed using a Poisson regression model.

Other measures included time to first relapse, drug discontinuation, time to

discontinuation, and new brain MRI lesions at 12 months.

RESULTS: The on-treatment ARR for DMF was 0.16 (95% CI (0.12, 0.18)) and 0.13

(95% CI (0.08, 0.16)) for FTY. PS weighting, which demonstrated excellent

covariate balance, showed no differences between groups on ARR (rate ratio=1.56,

95% CI (0.78, 3.14)), overall brain MRI activity defined as new T2 and/or

gadolinium enhancing (GdE) lesions (OR=1.38, 95% CI (0.78, 2.42)), new T2 lesions

(OR=1.33, 95% CI (0.71, 2.49)), and discontinuation (OR=1.30, 95% CI (0.84,

1.99)). DMF had higher odds of GdE lesions (OR=2.19, 95% CI (1.10, 4.35)),

earlier time to discontinuation (HR=1.35, 95% CI (1.05, 1.74)), and earlier

relapses (HR=1.64, 95% CI (1.10, 2.46)) compared to FTY.

CONCLUSION: Assessment in our clinical practice cohort showed comparable clinical

efficacy, overall brain MRI activity, and discontinuation between DMF and FTY at

12 months. DMF had increased GdE lesions and intolerability early after treatment

initiation.

Copyright Â© 2016 Elsevier B.V. All rights reserved.

DOI: 10.1016/j.msard.2016.08.002

PMID: 27919497 [Indexed for MEDLINE]

97. Ther Adv Neurol Disord. 2016 Nov;9(6):454-461. Epub 2016 Aug 4.

Efficacy and tolerability of dimethyl fumarate in White-, African- and Hispanic-

Americans with multiple sclerosis.

Zhovtis Ryerson L(1), Green R(2), Confident G(2), Pandey K(3), Richter B(3),

Bacon T(2), Sammarco C(2), Laing L(2), Kalina J(2), Kister I(2).

Author information:

(1)NYU Multiple Sclerosis Comprehensive Care Center, 240 East 38 Street, New

York, NY 10016, USA.

(2)NYU Multiple Sclerosis Care Center, NYU Langone Medical Center, New York, NY,

USA.

(3)Barnabas Multiple Sclerosis Care Center, Livingston, NJ, USA.

BACKGROUND: Dimethyl fumarate (DMF) was approved by the US Food and Drug

Administration (FDA) for treatment of relapsing-remitting multiple sclerosis

(RRMS) based on two phase III randomized clinical trials (RCTs). There were not

enough non-White patients enrolled in these RCTs to allow for subgroup analysis

based on race. Efficacy and tolerability of DMF therapy across various racial

groups is unknown.

METHODS: Retrospective chart review was performed on all patients with RRMS who

were started on DMF in two tertiary multiple sclerosis (MS) clinics. Efficacy and

tolerability of DMF was compared across three self-identified racial groups:

White-American (WA), African-American (AA) and Hispanic-American (HA).

RESULTS: A total of 390 RRMS patients were included in the study: 261 (66.9%) WA,

69 (17.7%) AA and 52 (13.3%) HA. When comparing 'pre-DMF' (1 year) and 'on DMF'

(mean follow up of 14 months) periods, statistically significant reduction in

rates of annualized relapses (WA from 0.44 to 0.19, AA from 0.39 to 0.15, and HA

from 0.39 to 0.14; no differences between groups), new T2 lesions (WA from 45% to

23%, AA from 39% to 23%, HA from 52% to 26%; no difference between groups), and

Gd+ lesions (WA from 25% to 13%, AA from 24% to 7%, HA from 23% to 12%; no

difference between groups) were seen. DMF was relatively well tolerated across

all groups, with an overall discontinuation rate of 20% (no difference between

the three groups).

CONCLUSION: Efficacy of DMF in our clinic population did not differ across three

major ethnic groups, WA, AA and HA, and was comparable with results observed in

the pivotal studies. These 'real-life' data suggest that race is not a factor

that needs to be taken into account when initiating DMF.

DOI: 10.1177/1756285616661929

PMCID: PMC5066529

PMID: 27800021

Conflict of interest statement: The author(s) declared the following potential

conflicts of interest with respect to the research, authorship, and/or

publication of this article: LZR has received research support from Biogen Idec.

She has received compensation for advisory board and speaker activities for

Biogen Idec and Teva. KP has received speaker and consulting fees from Acorda ,

TEVA, and Biogen Idec. CS has received compensation for advisory board and

speaker activities for Biogen Idec. IK has served on scientific advisory board

for Biogen Idec and Genentech. Received research support from Guthy-Jackson

Charitable Foundation, National Multiple Sclerosis Society, Biogen-Idec, Serono,

and Novartis. The other authors have no conflicts of interest to declare.

98. CNS Drugs. 2016 Oct;30(10):909-29. doi: 10.1007/s40263-016-0377-9.

Excluded b/c no primary data (meta-analysis)

Benefit-Risk of Therapies for Relapsing-Remitting Multiple Sclerosis: Testing the

Number Needed to Treat to Benefit (NNTB), Number Needed to Treat to Harm (NNTH)

and the Likelihood to be Helped or Harmed (LHH): A Systematic Review and

Meta-Analysis.

Mendes D(1)(2), Alves C(3)(4), Batel-Marques F(3)(4).

Author information:

(1)AIBILI-Association for Innovation and Biomedical Research on Light and Image,

CHAD-Centre for Health Technology Assessment and Drug Research, Azinhaga de Santa

Comba, Celas, 3000-548, Coimbra, Portugal. diogomendes26@gmail.com.

(2)School of Pharmacy, University of Coimbra, Coimbra, Portugal.

diogomendes26@gmail.com.

(3)AIBILI-Association for Innovation and Biomedical Research on Light and Image,

CHAD-Centre for Health Technology Assessment and Drug Research, Azinhaga de Santa

Comba, Celas, 3000-548, Coimbra, Portugal.

(4)School of Pharmacy, University of Coimbra, Coimbra, Portugal.

OBJECTIVE: This study aimed to test the number needed to treat to benefit (NNTB)

and to harm (NNTH), and the likelihood to be helped or harmed (LHH) when

assessing benefits, risks, and benefit-risk ratios of disease-modifying

treatments (DMTs) approved for relapsing-remitting multiple sclerosis (RRMS).

METHODS: In May 2016, we conducted a systematic review using the PubMed and

Cochrane Central Register of Controlled Trials databases to identify phase III,

randomized controlled trials with a duration of ≥2 years that assessed first-line

(dimethyl fumarate [DMF], glatiramer acetate [GA], β-interferons [IFN], and

teriflunomide) or second-line (alemtuzumab, fingolimod, and natalizumab) DMTs in

patients with RRMS. Meta-analyses were performed to estimate relative risks (RRs)

on annualized relapse rate (ARR), proportion of relapse-free patients (PPR-F),

disability progression (PP-F-CDPS3M), and safety outcomes. NNTB and NNTH values

were calculated applying RRs to control event rates. LHH was calculated as

NNTH/NNTB ratio.

RESULTS: The lowest NNTBs on ARR, PPR-F, and PP-F-CDPS3M were found with

IFN-β-1a-SC (NNTB 3, 95 % CI 2-4; NNTB 7, 95 % CI 4-18; NNTB 4, 95 % CI 3-7,

respectively) and natalizumab (NNTB 2, 95 % CI 2-3; NNTB 4, 95 % CI 3-6; NNTB 9,

95 % CI 6-19, respectively). The lowest NNTH on adverse events leading to

treatment discontinuation was found with IFN-β-1b (NNTH 14, 95 % 2-426) versus

placebo; a protective effect was noted with alemtuzumab versus IFN-β-1a-SC (NNTB

22, 95 % 17-41). LHHs >1 were more frequent with IFN-β-1a-SC and natalizumab.

CONCLUSIONS: These metrics may be valuable for benefit-risk assessments, as they

reflect baseline risks and are easily interpreted. Before making treatment

decisions, clinicians must acknowledge that a higher RR reduction with drug A as

compared with drug B (versus a common comparator in trial A and trial B,

respectively) does not necessarily mean that the number of patients needed to be

treated for one patient to encounter one aditional outcome of interest over a

defined period of time is lower with drug A than with drug B. Overall,

IFN-β-1a-SC and natalizumab seem to have the most favorable benefit-risk ratios

among first- and second-line DMTs, respectively.

DOI: 10.1007/s40263-016-0377-9

PMID: 27538416 [Indexed for MEDLINE]

99. J Neurol Neurosurg Psychiatry. 2016 Oct;87(10):1133-7. doi:

Excluded b/c no real-world adherence/persistence

10.1136/jnnp-2016-313760. Epub 2016 Jun 13.

Discontinuing disease-modifying therapy in MS after a prolonged relapse-free

period: a propensity score-matched study.

Kister I(1), Spelman T(2), Alroughani R(3), Lechner-Scott J(4), Duquette P(5),

Grand'Maison F(6), Slee M(7), Lugaresi A(8), Barnett M(9), Grammond P(10),

Iuliano G(11), Hupperts R(12), Pucci E(13), Trojano M(14), Butzkueven H(2);

MSBase Study Group.

Author information:

(1)Department of Neurology, NYU Multiple Sclerosis Care Center, NYU School of

Medicine, New York, New York, USA.

(2)Department of Neurology, Royal Melbourne Hospital, Parkville, Victoria,

Australia Department of Medicine (RMH), The University of Melbourne, Parkville,

Victoria, Australia.

(3)Amiri Hospital, Kuwait City, Kuwait.

(4)John Hunter Hospital, Newcastle, New South Wales, Australia.

(5)Hôpital Notre Dame, Montreal, Quebec, Canada.

(6)Neuro Rive-Sud, Hôpital Charles LeMoyne, Québec, Quebec, Canada.

(7)Flinders University and Flinders Medical Centre, Adelaide, South Australia,

Australia.

(8)Department of Biomedical and NeuroMotor Sciences (DIBINEM), Mater Studiorum -

Université di Bologna, Italy and IRCCS Istituto delle Scienze Neurologiche -

"UOSI Riabilitazione Sclerosi Multipla" - Bologna, Italy.

(9)Brain and Mind Research Institute, University of Sydney, Sydney, New South

Wales, Australia.

(10)Centre de réadaptation déficience physique Chaudière-Appalache, Levis,

Quebec, Canada.

(11)Ospedali Riuniti di Salerno, Salerno, Italy.

(12)Orbis Medical Centre, Sittard-Geleen, The Netherlands.

(13)UOC Neurologia, ASUR Marche, Area Vasta 3, Macerata, Italy.

(14)Department of Basic Medical Sciences, Neuroscience and Sense Organs,

University of Bari, Bari, Italy.

BACKGROUND: Discontinuation of injectable disease-modifying therapy (DMT) for

multiple sclerosis (MS) after a long period of relapse freedom is frequently

considered, but data on post-cessation disease course are lacking.

OBJECTIVES: (1) To compare time to first relapse and disability progression among

'DMT stoppers' and propensity-score matched 'DMT stayers' in the MSBase Registry;

(2) To identify predictors of time to first relapse and disability progression in

DMT stoppers.

METHODS: Inclusion criteria for DMT stoppers were: age ≥18 years; no relapses for

≥5 years at DMT discontinuation; follow-up for ≥3 years after stopping DMT; not

restarting DMT for ≥3 months after discontinuation. DMT stayers were required to

have no relapses for ≥5 years at baseline, and were propensity-score matched to

stoppers for age, sex, disability (Expanded Disability Status Score), disease

duration and time on treatment. Relapse and disability progression events in

matched stoppers and stayers were compared using a marginal Cox model. Predictors

of first relapse and disability progression among DMT stoppers were investigated

using a Cox proportional hazards model.

RESULTS: Time to first relapse among 485 DMT stoppers and 854 stayers was similar

(adjusted HR, aHR=1.07, 95% CI 0.84 to 1.37; p=0.584), while time to confirmed

disability progression was significantly shorter among DMT stoppers than stayers

(aHR=1.47, 95% CI 1.18 to 1.84, p=0.001). The difference in hazards of

progression was due mainly to patients who had not experienced disability

progression in the prebaseline treatment period.

CONCLUSIONS: Patients with MS who discontinued injectable DMT after a long period

of relapse freedom had a similar relapse rate as propensity score-matched

patients who continued on DMT, but higher hazard for disability progression.

Published by the BMJ Publishing Group Limited. For permission to use (where not

already granted under a licence) please go to

http://www.bmj.com/company/products-services/rights-and-licensing/

DOI: 10.1136/jnnp-2016-313760

PMID: 27298148 [Indexed for MEDLINE]

100. BMC Neurol. 2016 Sep 29;16(1):187.

Persistency, medication prescribing patterns, and medical resource use associated

with multiple sclerosis patients receiving oral disease-modifying therapies: a

retrospective medical record review.

Nazareth T(1), Friedman HS(2), Navaratnam P(2), Herriott DA(3), Ko JJ(4), Barr

P(3), Sasane R(1).

Author information:

(1)Novartis Pharmaceutical Corporation, East Hanover, NJ, USA.

(2)DataMed Solutions LLC, New York, NY, USA.

(3)Indegene TTM, Atlanta, GA, USA.

(4)Novartis Pharmaceutical Corporation, East Hanover, NJ, USA.

john.ko@novartis.com.

BACKGROUND: In the US, the approved multiple sclerosis (MS) oral

disease-modifying therapies (ODMTs) are fingolimod (FTY), teriflunomide (TFN),

and dimethyl fumarate (DMF). FTY and TFN are recommended with once-daily doses

with no up-titration, whereas DMF treatment is recommended twice-daily (BID) and

is initiated with a 7-day starter dose of 120 mg BID before up-titration to the

maintenance dose of 240 mg BID. Limited information exists regarding real-world

ODMT prescribing patterns to aid physician/patient decision-making.

METHODS: Eligible patients for this retrospective medical record review were

≥18 years, had one visit related to ODMT initiation (index visit), and ≥1 visit

within 12 months before and after the index visit. Primary objectives were to

assess post-index ODMT persistency (i.e., discontinuation), prescribing patterns

(medication switching, dose up-titrations, dose reduction, re-starts, and

add-ons) and medical resource utilization (office-visits, MRI procedures, and

mobility indicators) at distinct time windows of 3, 6, 9, and 12 months.

Chi-square or Wilcoxon Rank Sum tests were used for 3-way ODMT group comparisons.

RESULTS: Medical records of 293 MS-diagnosed patients using ODMTs were abstracted

from 19 US-based neurology clinics between December 31, 2010 and June 30, 2014

(FTY: 101; DMF: 133; TFN: 59). Persistency rates among ODMT groups were similar.

MS-related medication switching, dose reduction, re-starts, and add-ons were

infrequently observed and were similar across ODMT groups. Of DMF patients with a

confirmed starting dose of 120 mg BID with ≥12 months follow-up (n = 26), the

percentage who were prescribed dose up-titrations to the recommended maintenance

DMF dose was 23.1 % at 1-3 months, 26.9 % at 4-6 months, 42.3 % at 7-9 months,

and 0 % at 10-12 months. There were no significant differences at any time window

among the ODMT groups in the number of office visits or percent of patients

receiving MRIs. Mobility indicator patterns (proportion of patients with abnormal

gait, wheelchair use, etc.) were consistent over time.

CONCLUSIONS: There was no difference in persistency, prescribing patterns

(medication switching, dose reduction, re-starts, and add-ons) or medical

resource utilization (office-visits, MRI procedures, and mobility indicators)

among the ODMTs. However, in a small sub-group of patients, delays of up to

9 months in DMF dose-up titration to the recommended maintenance dose were

observed.

DOI: 10.1186/s12883-016-0698-9

PMCID: PMC5041514

PMID: 27683214

101. PLoS One. 2016 Sep 29;11(9):e0163296. doi: 10.1371/journal.pone.0163296.

Excluded b/c no real-world adherence/oersistence

eCollection 2016.

The Efficacy of Natalizumab versus Fingolimod for Patients with

Relapsing-Remitting Multiple Sclerosis: A Systematic Review, Indirect Evidence

from Randomized Placebo-Controlled Trials and Meta-Analysis of Observational

Head-to-Head Trials.

Tsivgoulis G(1)(2)(3), Katsanos AH(1)(4), Mavridis D(5)(6), Grigoriadis N(7),

Dardiotis E(8), Heliopoulos I(9), Papathanasopoulos P(10), Karapanayiotides T(7),

Kilidireas C(11), Hadjigeorgiou GM(8), Voumvourakis K(1); HELANI (Hellenic

Academy of Neuroimmunology).

Author information:

(1)Second Department of Neurology, "Attikon" Hospital, School of Medicine,

University of Athens, Athens, Greece.

(2)Department of Neurology, The University of Tennessee Health Science Center,

Memphis, Tennessee, United States of America.

(3)International Clinical Research Center, Department of Neurology, St. Anne's

University Hospital in Brno, Brno, Czech Republic.

(4)Department of Neurology, School of Medicine, University of Ioannina, Ioannina,

Greece.

(5)Department of Primary Education, University of Ioannina, Ioannina, Greece.

(6)Department of Hygiene and Epidemiology, School of Medicine, University of

Ioannina, Ioannina, Greece.

(7)Second Department of Neurology, "AHEPA" University Hospital, Aristotelion

University of Thessaloniki, Thessaloniki, Macedonia, Greece.

(8)Department of Neurology, University Hospital of Larissa, University of

Thessaly, Larissa, Greece.

(9)Department of Neurology, Alexandroupolis University Hospital, Democritus

University of Thrace, Alexandroupolis, Greece.

(10)Department of Neurology, University of Patras Medical School, Patras, Greece.

(11)First Department of Neurology, "Eginition" Hospital, School of Medicine,

University of Athens, Athens, Greece.

BACKGROUND: Although Fingolimod (FGD) and Natalizumab (NTZ) appear to be

effective in relapsing-remitting multiple sclerosis (RRMS), they have never been

directly compared in a randomized clinical trial (RCT).

METHODS AND FINDINGS: We evaluated the comparative efficacy of FGD vs. NTZ using

a meta-analytical approach. Data from placebo-controlled RCTs was used for

indirect comparisons and observational data was utilized for head-to-head

comparisons. We identified 3 RCTs (2498 patients) and 5 observational studies

(2576 patients). NTZ was associated with a greater reduction in the 2-year

annualized relapse rate (ARR; SMDindirect = -0.24;95% CI: from -0.44 to -0.04; p

= 0.005) and with the probability of no disease activity at 2 years

(ORindirect:1.82, 95% CI: from 1.05 to 3.15) compared to FGD, while no

differences between the two therapies were found in the proportion of patients

who remained relapse-free (ORindirect = 1.20;95% CI: from 0.84 to 1.71) and those

with disability progression (ORindirect = 0.76;95% CI: from 0.48 to 1.21) at 2

years. In the analysis of observational data, we found no significant differences

between NTZ and FGD in the 2-year ARR (SMD = -0.05; 95% CI: from -0.26 to 0.16),

and 2-year disability progression (OR:1.08;95% CI: from 0.77 to 1.52). However,

NTZ-treated patients were more likely to remain relapse-free at 2-years compared

to FGD (OR: 2.19;95% CI: from 1.15 to 4.18; p = z0.020).

CONCLUSIONS: Indirect analyses of RCT data and head-to-head comparisons of

observational findings indicate that NTZ may be more effective than FGD in terms

of disease activity reduction in patients with RRMS. However, head-to-head RCTs

are required to independently confirm this preliminary observation.

DOI: 10.1371/journal.pone.0163296

PMCID: PMC5042498

PMID: 27684943

Conflict of interest statement: Dr. Tsivgoulis has received research support (not

related to this project) by Teva Pharmaceutical Hellas, Genesis Pharma, Merck

Serono and Novartis Hellas. Dr. Grigoriadis has received research support (not

related to this project) by Biogen Idec, Novartis, TEVA, Merck Serono, and

Genesis Pharma. Dr. Dardiotis has received research support (not related to this

project) from Novartis Hellas SA, Genesis Pharma, Bayer Hellas AG, Genzyme, Teva

and Merck-Serono. Dr. Dardiotis has also received research support (not related

to this project) by Novartis. Dr. Papathanasopoulos has received research support

(not related to this project) by Biogen Idec, Novartis, TEVA, Merck Serono, and

Genesis Pharma. Dr. Voumvourakis has received research support (not related to

this project) by Teva Pharmaceutical Hellas, Merck Serono, Genzyme, Genesis

Pharma and Novartis Hellas. All authors have no other relevant declarations

relating to employment, consultancy, patents, products in development, marketed

products, etc. This does not alter our adherence to PLOS ONE policies on sharing

data and materials.

102. Am J Ophthalmol Case Rep. 2016 Sep 28;4:67-70. doi: 10.1016/j.ajoc.2016.09.005.

Excluded b/c no real-world adherence/persistence

eCollection 2016 Dec.

Acute onset of fingolimod-associated macular edema.

Soliman MK(1)(2), Sarwar S(1), Sadiq MA(1), Jack L(3), Jouvenat N(4), Zabad

RK(4), Kedar S(3)(4), Nguyen QD(1)(3).

Author information:

(1)Ocular Imaging Research and Reading Center, Omaha, NE, 68131, USA.

(2)Department of Ophthalmology, Assiut University Hospital, Assiut University,

Assuit, Egypt.

(3)Stanley M. Truhlsen Eye Institute, University of Nebraska Medical Center,

Omaha, NE, 168198-5540, USA.

(4)Department of Neurological Sciences, University of Nebraska Medical Center,

Omaha, NE, 168198, USA.

Purpose: Fingolimod is among the first oral disease-modifying agents for the

treatment of relapsing-remitting multiple sclerosis (MS). Despite its favorable

safety profile, fingolimod may cause macular edema, a significant adverse event,

which occurs within the first 4 months of therapy. Macular edema usually resolves

upon discontinuation of fingolimod; however, the time required for resolution of

this condition is unknown.

Observations: A 42-year-old white male with a history of relapsing-remitting MS

presented with blurring of vision in his left eye 24 h after the first dose of

fingolimod. Dilated fundus examination of the left eye revealed an increased

retinal thickness and mild optic disc pallor. Spectral domain optical coherence

tomography (SD-OCT) confirmed the diagnosis of cystoid macular edema. Topical

nonsteroidal anti-inflammatory drug (NSAID) was initiated immediately after the

diagnosis, and fingolimod therapy was discontinued shortly thereafter. Seven

weeks after the initial presentation, intermediate uveitis was noted in the

inferior periphery of the left eye, and SD-OCT revealed worsening of macular

edema. Acetazolamide therapy was added to the topical NSAID to control the edema.

Three weeks after initiation of acetazolamide, macular thickness reduced

significantly. The patient then stopped all medications, and 3 weeks later

macular edema rebounded. Systemic steroid was employed to control both the

intermediate uveitis and macular edema.

Conclusions and importance: We report a case of acute and very rapid onset of

fingolimod-associated macular edema (FAME). Acetazolamide may have a beneficial

effect on macular edema secondary to fingolimod. It is unclear if intermediate

uveitis is associated with the rapid development of FAME.

DOI: 10.1016/j.ajoc.2016.09.005

PMCID: PMC5757484

PMID: 29503930

103. Patient Prefer Adherence. 2016 Sep 26;10:1945-1956. eCollection 2016.

Excluded b/c no real-world adherence/persistence

Patient preferences for treatment of multiple sclerosis with disease-modifying

therapies: a discrete choice experiment.

Garcia-Dominguez JM(1), Muñoz D(2), Comellas M(3), Gonzalbo I(3), Lizán L(3),

Polanco Sánchez C(4).

Author information:

(1)Multiple Sclerosis Unit, Hospital General Universitario Gregorio Marañon,

Madrid.

(2)Neurology Department, Hospital Universitario Alvaro Cunqueiro, Vigo.

(3)Outcomes'10, Jaime I University, Castellón.

(4)Health Economics & Outcomes Research, Merck, Madrid, Spain.

OBJECTIVES: To assess disease-modifying therapy (DMT) preferences in a population

of patients with multiple sclerosis (MS) and to estimate the association between

sociodemographic and clinical factors and these preferences.

METHODS: Preferences for DMTs attributes were measured using a discrete choice

experiment. Analysis of preferences was assessed using mixed-logit hierarchical

Bayes regression. A multilinear regression was used to evaluate the association

between the preferences for each attribute and patients' demographic and clinical

characteristics. A Student's t-test or Welch's t-test was used for subgroup

comparisons.

RESULTS: A total of 125 patients were included in the final analysis (62.9%

female, mean age 44.5 years, 71.5% with relapsing-remitting MS diagnosis). The

most important factor for patients was the possibility of suffering from the side

effects of the treatment (relative importance [RI] =50%), followed by a delay in

disease progression (RI =19.4%), and route and frequency of administration (RI

=14.3%). According to maximum acceptable risk, patients were willing to accept an

increase of 3.8% in severity of side effects, for a delay of 1 year in disease

progression. Treatment duration was the most prevalent factor affecting

preferences, followed by the age of patients, type of MS, level of education, and

the type of current treatment. Patients treated orally were significantly more

concerned about the route and frequency of administration (P=0.026) than patients

on injectable therapy. Naïve patients stated significantly less importance to

prevention of relapses (P=0.021) and deterioration of the capacity for performing

usual daily life activities (P=0.015). Finally, patients with >5 years since

diagnosis were significantly less concerned about preventing disease progression

(P=0.021), and more concerned about treatment side effects (P=0.052) than

compared with patients with <5 years of MS history.

CONCLUSION: The most important attribute for MS patients was side effects of

DMTs, followed by delay in disability progression. Experience with DMTs and time

since MS diagnosis changed patients' preferences. These results give information

to adjust new DMT treatment in order to satisfy patients' preferences and

therefore, improve adherence to treatment.

DOI: 10.2147/PPA.S114619

PMCID: PMC5045232

PMID: 27713622

104. BMC Neurol. 2016 Sep 21;16(1):181.

Excluded b/c no real-world adherence/persistence

Comparative evaluation of patients' and physicians' satisfaction with interferon

beta-1b therapy.

Zettl UK(1), Bauer-Steinhusen U(2), Glaser T(2), Hechenbichler K(3), Hecker M(4);

Study Group.

Author information:

(1)Department of Neurology, Neuroimmunology Section, University of Rostock,

Gehlsheimer Str. 20, 18147, Rostock, Germany.

(2)Neurology, Immunology, and Ophthalmology, Bayer Vital GmbH, Leverkusen,

Germany.

(3)Institute Dr. Schauerte, Munich, Germany.

(4)Department of Neurology, Neuroimmunology Section, University of Rostock,

Gehlsheimer Str. 20, 18147, Rostock, Germany. michael.hecker@rocketmail.com.

BACKGROUND: Due to the preventive nature of disease-modifying therapies for

multiple sclerosis, treatment success particularly depends on adherence to

therapeutic regimens and patients' perception of treatment efficacy. The latter

is strongly influenced by the confidence in the involved health care

professionals and the relationship to the treating physician.

METHODS: In this report, we considered physicians' and patients' evaluation of

satisfaction with interferon beta-1b treatment efficacy for assessing the

congruence in ratings. Data were queried in a study conducted between 2009 and

2013.

RESULTS: After 6 months of therapy, > 80 % of the patients and physicians

(N = 445) showed high degrees of satisfaction regarding interferon beta-1b

treatment, with only few physicians and patients (≤2.0 %) rating "not satisfied".

The proportion of patients rating with the same category as their physicians was

similar after 6 months (47 % congruence) and at the 24 months/study end visit

(49 %). Discrepancies between ratings were observed with respect to study end:

for patients with premature study end, more patients and physicians rated being

not satisfied with the therapy, accompanied by a considerably lower congruence of

33 % compared to 54 % for patients receiving the therapy for at least 2 years and

completing the study regularly.

CONCLUSIONS: Regular communication between physicians and patients about their

perception of therapy might improve alignment of treatment evaluation and could

result in increased therapy persistence. In addition, patients' willingness to

perform a long-term therapy - even in the absence of disease symptoms - might be

promoted by repeated exchange between health care providers and patients with

regard to realistic treatment expectations.

TRIAL REGISTRATION: ClinicalTrials.gov NCT00902135 (registered May 13, 2009).

DOI: 10.1186/s12883-016-0705-1

PMCID: PMC5031257

PMID: 27653529

105. Biologics. 2016 Sep 12;10:119-38. doi: 10.2147/BTT.S89218. eCollection 2016.

Excluded b/c no primary data (narrative review)

Therapeutic efficacy of monthly subcutaneous injection of daclizumab in relapsing

multiple sclerosis.

Cohan S(1).

Author information:

(1)Providence Multiple Sclerosis Center; Providence Brain and Spine Institute;

Providence Health & Services, Portland, OR, USA.

Despite the availability of multiple disease-modifying therapies for relapsing

multiple sclerosis (MS), there remains a need for highly efficacious targeted

therapy with a favorable benefit-risk profile and attributes that encourage a

high level of treatment adherence. Daclizumab is a humanized monoclonal antibody

directed against CD25, the α subunit of the high-affinity interleukin 2 (IL-2)

receptor, that reversibly modulates IL-2 signaling. Daclizumab treatment leads to

antagonism of proinflammatory, activated T lymphocyte function and expansion of

immunoregulatory CD56(bright) natural killer cells, and has the potential to, at

least in part, rectify the imbalance between immune tolerance and autoimmunity in

relapsing MS. The clinical pharmacology, efficacy, and safety of subcutaneous

daclizumab have been evaluated extensively in a large clinical study program. In

pivotal studies, daclizumab demonstrated superior efficacy in reducing clinical

and radiologic measures of MS disease activity compared with placebo or

intramuscular interferon beta-1a, a standard-of-care therapy for relapsing MS.

The risk of hepatic disorders, cutaneous events, and infections was modestly

increased. The monthly subcutaneous self-injection dosing regimen of daclizumab

may be advantageous in maintaining patient adherence to treatment, which is

important for optimal outcomes with MS disease-modifying therapy. Daclizumab has

been approved in the US and in the European Union and represents an effective new

treatment option for patients with relapsing forms of MS, and is currently under

review by other regulatory agencies.

DOI: 10.2147/BTT.S89218

PMCID: PMC5026217

PMID: 27672308

106. PLoS One. 2016 Sep 9;11(9):e0162162. doi: 10.1371/journal.pone.0162162.

Excluded b/c no real-world adherence/persistence

eCollection 2016.

Preclinical Metabolism, Pharmacokinetics and In Vivo Analysis of New

Blood-Brain-Barrier Penetrant Fingolimod Analogues: FTY720-C2 and FTY720-Mitoxy.

Enoru JO(1), Yang B(2), Krishnamachari S(2), Villanueva E(2), DeMaio W(1),

Watanyar A(1), Chinnasamy R(3), Arterburn JB(3), Perez RG(2).

Author information:

(1)In Vitro and Molecular Metabolism Laboratory, Ricerca Biosciences LLC,

Concord, Ohio, United States of America.

(2)Department of Biomedical Sciences, Graduate School of Biomedical Sciences,

Center of Emphasis in Neurosciences, Paul L. Foster School of Medicine, Texas

Tech University Health Sciences Center El Paso, El Paso, Texas, United States of

America.

(3)Department of Chemistry and Biochemistry, New Mexico State University, Las

Cruces, New Mexico, United States of America.

Parkinson's disease (PD) is a neurodegenerative aging disorder in which

postmortem PD brain exhibits neuroinflammation, as well as

synucleinopathy-associated protein phosphatase 2A (PP2A) enzymatic activity loss.

Based on our translational research, we began evaluating the

PD-repurposing-potential of an anti-inflammatory, neuroprotective, and PP2A

stimulatory oral drug that is FDA-approved for multiple sclerosis, FTY720

(fingolimod, Gilenya®). We also designed two new FTY720 analogues, FTY720-C2 and

FTY720-Mitoxy, with modifications that affect drug potency and mitochondrial

localization, respectively. Herein, we describe the metabolic stability and

metabolic profiling of FTY720-C2 and FTY720-Mitoxy in liver microsomes and

hepatocytes. Using mouse, rat, dog, monkey, and human liver microsomes the

intrinsic clearance of FTY720-C2 was 22.5, 79.5, 6.0, 20.2 and 18.3 μL/min/mg;

and for FTY720-Mitoxy was 1.8, 7.8, 1.4, 135.0 and 17.5 μL/min/mg, respectively.

In hepatocytes, both FTY720-C2 and FTY720-Mitoxy were metabolized from the octyl

side chain, generating a series of carboxylic acids similar to the parent FTY720,

but without phosphorylated metabolites. To assess absorption and distribution, we

gave equivalent single intravenous (IV) or oral doses of FTY720-C2 or

FTY720-Mitoxy to C57BL/6 mice, with two mice per time point evaluated. After IV

delivery, both FTY720-C2 and FTY720-Mitoxy were rapidly detected in plasma and

brain; and reached peak concentrations at the first sampling time points. After

oral dosing, FTY720-C2 was present in plasma and brain, although FTY720-Mitoxy

was not orally bioavailable. Brain-to-plasma ratio of both compounds increased

time-dependently, suggesting a preferential partitioning to the brain. PP2A

activity in mouse adrenal gland increased ~2-fold after FTY720-C2 or

FTY720-Mitoxy, as compared to untreated controls. In summary, FTY720-C2 and

FTY720-Mitoxy both (i) crossed the blood-brain-barrier; (ii) produced metabolites

similar to FTY720, except without phosphorylated species that cause

S1P1-mediated-immunosuppression; and (iii) stimulated in vivo PP2A activity, all

of which encourage additional preclinical assessment.

DOI: 10.1371/journal.pone.0162162

PMCID: PMC5017749

PMID: 27611691 [Indexed for MEDLINE]

Conflict of interest statement: Julius O. Enoru, William DeMaio and Adiba

Watanyar are employed by Ricerca Biosciences LLC. Our relationship with the

commercial entity does not alter adherence to PLOS ONE policies on sharing data

and materials. The corresponding author has filed a patent, “Compositions and

Methods for the Treatment of Parkinson's Disease”, US 20150290145, CA 2888634,

which does not alter adherence to PLOS ONE policies on sharing data and

materials. However, some restrictions exist with regard to material sharing, as

that would require additional synthesis and related expenses. We will share with

anyone who is willing to pay for new synthesis and shipping.

107. BMC Res Notes. 2016 Sep 7;9(1):434. doi: 10.1186/s13104-016-2243-8.

The real-world patient experience of fingolimod and dimethyl fumarate for

multiple sclerosis.

Wicks P(1), Rasouliyan L(2), Katic B(3), Nafees B(4), Flood E(4), Sasané R(5).

Author information:

(1)PatientsLikeMe, 160 Second Street, Cambridge, MA, 02142, USA.

pwicks@patientslikeme.com.

(2)ICON Plc, Medical Affairs Statistical Analysis, Torre Diagonal Mar, Josep Pla,

2, Planta 11, Módulo A1, 08019, Barcelona, Spain.

(3)PatientsLikeMe, 160 Second Street, Cambridge, MA, 02142, USA.

(4)ICON Plc, Clinical Outcomes Assessments, 820 W Diamond Ave Ste 100,

Gaithersburg, MD, 20878, USA.

(5)Novartis Pharmaceuticals Corporation, 1 Health Plaza, East Hanover, NJ, 07936,

USA.

BACKGROUND: Oral disease-modifying therapies offer equivalent or superior

efficacy and greater convenience versus injectable options.

OBJECTIVES: To compare patient-reported experiences of fingolimod and dimethyl

fumarate.

METHODS: Adult relapsing-remitting multiple sclerosis patients treated with

fingolimod or dimethyl fumarate were recruited from an online patient community

and completed an online survey about treatment side effects, discontinuation, and

satisfaction.

RESULTS: 281 patients in four groups completed the survey: currently receiving

fingolimod (CF, N = 61), currently receiving dimethyl fumarate (CDMF, N = 129),

discontinued fingolimod (DF, N = 32) and discontinued dimethyl fumarate (DDMF,

N = 59). Reasons for treatment switch were to take oral treatment (CF: 63.3 %,

CDMF: 61.8 %), side effects of prior medication (CF: 67.3 %, CDMF: 44.1 %) and

lack of effectiveness of prior medication (CF: 38.8 %, CDMF: 31.4 %). Main

reasons for discontinuation were side effects (DF: 46.9 %, DDMF: 67.8 %) and lack

of effectiveness (DF: 25.0 %, DDMF: 15.3 %). CDMF patients had an increased risk

of abdominal pain, flushing, diarrhea, and nausea. Treatment satisfaction was

highest among CF patients followed by CDMF, DF, and then DDMF patients.

CONCLUSIONS: Discontinuation was driven by experience of side effects. Patients

currently taking dimethyl fumarate were more likely to experience a side effect

versus patients currently taking fingolimod. Examination of the relationship

between tolerability and adherence/persistence is needed.

DOI: 10.1186/s13104-016-2243-8

PMCID: PMC5015319

PMID: 27604188 [Indexed for MEDLINE]

Excluded b/c no primary data (narrative review)

108. Expert Opin Biol Ther. 2016 Sep;16(9):1151-62. doi:

10.1080/14712598.2016.1213810. Epub 2016 Jul 27.

Use of natalizumab in multiple sclerosis: current perspectives.

Gandhi S(1), Jakimovski D(1), Ahmed R(1), Hojnacki D(2), Kolb C(2),

Weinstock-Guttman B(2), Zivadinov R(1)(3).

Author information:

(1)a Buffalo Neuroimaging Analysis Center, Department of Neurology , University

at Buffalo, State University of New York , Buffalo , NY , USA.

(2)b Jacobs MS Center, Department of Neurology , University at Buffalo, State

University of New York , Buffalo , NY , USA.

(3)c MR Imaging Clinical Translational Research Center, School of Medicine and

Biomedical Sciences , University at Buffalo, State University of New York ,

Buffalo , NY , USA.

INTRODUCTION: Natalizumab is an efficacious monoclonal antibody approved for use

in relapsing-remitting multiple sclerosis (RRMS). Multiple studies have

demonstrated reduced relapse rate, decreased disability progression and prolonged

disease-free intervals with natalizumab use. However, natalizumab is associated

with an increased risk of progressive multifocal leukoencephalopathy (PML), thus

restricting its widespread use with populations at high risk for developing PML.

Recently, the effect of natalizumab in secondary-progressive (SPMS) population

has been explored.

AREAS COVERED: This review highlights the pathophysiology behind disease

progression in MS and summarizes various attributes of natalizumab including: its

pharmacological properties and global economic impact, results of clinical

efficacy studies, its role in SPMS, pregnancy and its adverse events profile

including PML and discontinuation protocols.

EXPERT OPINION: Despite an established role in reducing RRMS disease activity,

natalizumab has found limited use in SPMS due to insufficient evidence of

efficacy. Current disease-modifying therapies exert modest overall benefit in

SPMS owing to its complex pathophysiology, higher prevalence of comorbidities and

increased PML risk with age and lack of reliable outcome measures. Finding more

appropriate MRI and clinical outcome measures is quintessential for designing

future randomized trials and possibly exploring primary neuroprotective agents

for treating SPMS.

DOI: 10.1080/14712598.2016.1213810

PMID: 27413840 [Indexed for MEDLINE]

109. J Neurol Neurosurg Psychiatry. 2016 Sep;87(9):944-51. doi:

Excluded b/c no real-world adherence/persistence

10.1136/jnnp-2015-312591. Epub 2016 May 9.

Efficacy and safety of cannabinoid oromucosal spray for multiple sclerosis

spasticity.

Patti F(1), Messina S(1), Solaro C(2), Amato MP(3), Bergamaschi R(4), Bonavita

S(5), Bruno Bossio R(6), Brescia Morra V(7), Costantino GF(8), Cavalla P(9),

Centonze D(10), Comi G(11), Cottone S(12), Danni M(13), Francia A(14), Gajofatto

A(15), Gasperini C(16), Ghezzi A(17), Iudice A(18), Lus G(19), Maniscalco GT(20),

Marrosu MG(21), Matta M(22), Mirabella M(23), Montanari E(24), Pozzilli C(25),

Rovaris M(26), Sessa E(27), Spitaleri D(28), Trojano M(29), Valentino P(30),

Zappia M(1); SA.FE. study group.

Collaborators: Benedetti M, Bertolotto A, Berra E, Bianco A, Buttari F, Cerqua R,

Florio C, Fuiani A, Guareschi A, Ippolito D, Nuara A, Palmieri V, Paolicelli D,

Petrucci L, Pontecorvo S, Saccà F, Salamone G, Signoriello E, Spinicci G, Russo

M, Tavazzi E, Trabucco E, Trotta M, M Z.

Author information:

(1)Department of Medical, Surgical Science and Advanced Technology "GF

Ingrassia", University of Catania, Catania, Italy.

(2)Neurology Unit, Department Head and Neck, ASL3 Genova, Genova, Italy.

(3)Department NEUROFARBA, University of Florence, Florence, Italy.

(4)Department of Neurology, Neurology Institute C Mondino, Pavia, Italy.

(5)I Clinic Neurology, II University of Naples, Naples, Italy.

(6)Neurology Operating Unit, Multiple Sclerosis Center, Provincial Health

Authority of Cosenza, Cosenza, Italy.

(7)Multiple Sclerosis Centre, University Federico II, Naples, Italy.

(8)Demyelinating Diseases Centre, Foggia Hospital, Foggia, Italy.

(9)Multiple Sclerosis Centre, S. Giovanni Battista, Molinette Hospital, Turin,

Italy.

(10)Department of Systems Medicine, Multiple Sclerosis Clinical and Research

Center, Tor Vergata University, Rome, Italy Unit of Neurology and of

Neurorehabilitation, IRCCS Neuromed, Pozzilli (IS), Italy.

(11)Department of Neurology, San Raffaele Hospital, Milan, Italy.

(12)Neuroimmunology Unit, Villa Sofia-Cervello Hospital, Palermo, Italy.

(13)Neurology Clinic, Ancona Hospital, Ancona, Italy.

(14)Department Neurol Psich, Multiple Sclerosis Center, Sapienza University,

Rome, Italy.

(15)Multiple Sclerosis Centre, University of Verona, Verona, Italy.

(16)Neurology Division, San Camillo Hospital, Rome, Italy.

(17)Multiple Sclerosis Centre, Sant'Antonio Abate Hospital, Gallarate, Italy.

(18)Multiple Sclerosis Centre, University Hospital Pisa, Pisa, Italy.

(19)Multiple Sclerosis Center, Second University of Naples, Naples, Italy.

(20)Multiple Sclerosis Centre, Cardarelli Hospital, Naples, Italy.

(21)Department of Medical Sciences, University of Cagliari, Cagliari, Italy.

(22)Multiple Sclerosis Centre (CRESM), San Luigi Gonzaga Hospital, Orbassano,

Italy.

(23)Multiple Sclerosis Centre, Cattolica University, Rome, Italy.

(24)Multiple Sclerosis Centre, Vaio Hospital, Fidenza, Italy.

(25)Multiple Sclerosis Centre, S. Andrea Hospital, Rome, Italy.

(26)Multiple Sclerosis Centre, IRCCS Don Gnocchi Foundation, Milan, Italy.

(27)Multiple Sclerosis Centre, IRCCS-Bonino Pulejo Centre, Messina, Italy.

(28)Multiple Sclerosis Centre, San G. Moscati Hospital, Avellino, Italy.

(29)Department of Basic Medical Sciences, Neuroscience and Sense Organs,

University of Bari "Aldo Moro", Bari, Italy.

(30)Department of Medical Sciences, Institute of Neurology, University "Magna

Graecia", Catanzaro, Italy.

BACKGROUND: The approval of 9-δ-tetrahydocannabinol and cannabidiol (THC:CBD)

oromucosal spray (Sativex) for the management of treatment-resistant multiple

sclerosis (MS) spasticity opened a new opportunity for many patients. The aim of

our study was to describe Sativex effectiveness and adverse events profile in a

large population of Italian patients with MS in the daily practice setting.

METHODS: We collected data of all patients starting Sativex between January 2014

and February 2015 from the mandatory Italian medicines agency (AIFA) e-registry.

Spasticity assessment by the 0-10 numerical rating scale (NRS) scale is available

at baseline, after 1 month of treatment (trial period), and at 3 and 6 months.

RESULTS: A total of 1615 patients were recruited from 30 MS centres across Italy.

After one treatment month (trial period), we found 70.5% of patients reaching a

≥20% improvement (initial response, IR) and 28.2% who had already reached a ≥30%

improvement (clinically relevant response, CRR), with a mean NRS score reduction

of 22.6% (from 7.5 to 5.8). After a multivariate analysis, we found an increased

probability to reach IR at the first month among patients with primary and

secondary progressive MS, (n=1169, OR 1.4 95% CI 1.04 to 1.9, p=0.025) and among

patients with >8 NRS score at baseline (OR 1.8 95% CI 1.3-2.4 p<0.001). During

the 6 months observation period, 631(39.5%) patients discontinued treatment. The

main reasons for discontinuation were lack of effectiveness (n=375, 26.2%) and/or

adverse events (n=268, 18.7%).

CONCLUSIONS: Sativex can be a useful and safe option for patients with MS with

moderate to severe spasticity resistant to common antispastic drugs.

Published by the BMJ Publishing Group Limited. For permission to use (where not

already granted under a licence) please go to

http://www.bmj.com/company/products-services/rights-and-licensing/

DOI: 10.1136/jnnp-2015-312591

PMCID: PMC5013116

PMID: 27160523 [Indexed for MEDLINE]

Excluded b/c no real-world adherence/persistence

110. J Neurol Neurosurg Psychiatry. 2016 Sep;87(9):937-43. doi:

10.1136/jnnp-2015-312221. Epub 2016 Jan 18.

Randomised natalizumab discontinuation study: taper protocol may prevent disease

reactivation.

Weinstock-Guttman B(1), Hagemeier J(2), Kavak KS(3), Saini V(3), Patrick K(3),

Ramasamy DP(2), Nadeem M(3), Carl E(2), Hojnacki D(3), Zivadinov R(4).

Author information:

(1)Jacobs Comprehensive MS Treatment and Research Center, University at Buffalo,

Buffalo, New York, USA Department of Neurology, State University of New York at

Buffalo, Buffalo, New York, USA.

(2)Department of Neurology, Buffalo Neuroimaging Analysis Center, School of

Medicine and Biomedical Sciences, University at Buffalo, State University of New

York, Buffalo, New York, USA.

(3)Jacobs Comprehensive MS Treatment and Research Center, University at Buffalo,

Buffalo, New York, USA.

(4)Department of Neurology, State University of New York at Buffalo, Buffalo, New

York, USA Department of Neurology, Buffalo Neuroimaging Analysis Center, School

of Medicine and Biomedical Sciences, University at Buffalo, State University of

New York, Buffalo, New York, USA MR Imaging Clinical Translational Research

Center, School of Medicine and Biomedical Sciences, University at Buffalo, State

University of New York, Buffalo, New York, USA.

OBJECTIVES: To compare two modes of natalizumab cessation interventions:

immediate versus tapered down, as measured by serial MRI and the occurrence of

relapses during a 12-month period.

BACKGROUND: Weighing progressive multifocal encephalopathy risk associated with

≥24 months of natalizumab therapy against the benefits of disease control, we

initiated a natalizumab discontinuation study.

METHODS: A phase IV, 12-month, single-blinded randomised (MRI) study. Fifty

relapsing patients with multiple sclerosis (MS) who had been on natalizumab

therapy ≥24 months and were contemplating natalizumab discontinuation were

enrolled. Participants were randomised to either the immediate discontinuation

group (IDG) or the tapered group (TG). IDG discontinued natalizumab at once and

initiated another disease modifying therapy (DMT) following the last natalizumab

infusion, while the TG received two more natalizumab infusions, at 6 and 8 weeks

(14 weeks from study entry) before initiating another DMT. Standardised MRI was

performed at baseline, 6 and 12 months from the last natalizumab infusion.

RESULTS: A higher rate of relapses in the IDG (n=28) compared to the TG (n=8)

over 12 months from the last infusion (p=0.007) was observed, most relapses

occurred within 3 months of discontinuation (20 vs 7 relapses, p=0.012). The IDG

showed a higher number of new T2 lesions within 6-12 months of discontinuation

(p=0.025), a higher mean absolute T2-LV change from 0 to 12 months (1.1 vs

0.1 mL, p=0.024) and a higher number of new T1-hypointense lesions over

0-12 months (p=0.005) as well as from baseline to 6 months (p=0.026) compared to

the TG.

CONCLUSIONS: Natalizumab discontinuation therapy was associated with development

of new disease activity. Our tapered protocol showed benefits, as patients in the

TG experienced less relapses and lower accumulation of MRI lesions compared to

those in the IDG.

Published by the BMJ Publishing Group Limited. For permission to use (where not

already granted under a licence) please go to

http://www.bmj.com/company/products-services/rights-and-licensing/

DOI: 10.1136/jnnp-2015-312221

PMID: 26780938

111. Mult Scler Relat Disord. 2016 Sep;9:80-90. doi: 10.1016/j.msard.2016.07.001. Epub

Excluded b/c no real-world adherence/persistence

2016 Jul 6.

Low-fat, plant-based diet in multiple sclerosis: A randomized controlled trial.

Yadav V(1), Marracci G(2), Kim E(2), Spain R(2), Cameron M(2), Overs S(3),

Riddehough A(4), Li DK(4), McDougall J(5), Lovera J(6), Murchison C(7), Bourdette

D(2).

Author information:

(1)Department of Neurology, Oregon Health & Science University, USA; Department

of Veterans Affairs MS Center of Excellence-West, Portland, OR, USA. Electronic

address: yadavv@ohsu.edu.

(2)Department of Neurology, Oregon Health & Science University, USA; Department

of Veterans Affairs MS Center of Excellence-West, Portland, OR, USA.

(3)Novant Medical Group, Charlotte, NC, USA.

(4)MS/MRI Research Group, University of British Columbia, Vancouver, BC, Canada.

(5)The McDougall Research and Education Foundation, Santa Rosa, CA, USA.

(6)Department of Neurology, Louisiana State University, New Orleans, LA, USA.

(7)Department of Neurology, Oregon Health & Science University, USA.

BACKGROUND: The role that dietary interventions can play in multiple sclerosis

(MS) management is of huge interest amongst patients and researchers but data

evaluating this is limited. Possible effects of a very-low-fat, plant-based

dietary intervention on MS related progression and disease activity as measured

by brain imaging and MS related symptoms have not been evaluated in a

randomized-controlled trial. Despite use of disease modifying therapies (DMT),

poor quality of life (QOL) in MS patients can be a significant problem with

fatigue being one of the common disabling symptoms. Effective treatment options

for fatigue remain limited. Emerging evidence suggests diet and vascular risk

factors including obesity and hyperlipidemia may influence MS disease progression

and improve QOL.

OBJECTIVES: To evaluate adherence, safety and effects of a very-low-fat,

plant-based diet (Diet) on brain MRI, clinical [MS relapses and disability, body

mass index (BMI)] and metabolic (blood lipids and insulin) outcomes, QOL [Short

Form-36 (SF-36)], and fatigue [Fatigue Severity Scale (FSS) and Modified Fatigue

Impact Scale (MFIS)], in relapsing-remitting MS (RRMS).

METHODS: This was a randomized-controlled, assessor-blinded, one-year long study

with 61 participants assigned to either Diet (N=32) or wait-listed (Control,

N=29) group.

RESULTS: The mean age (years) [Control-40.9±8.48; Diet-40.8±8.86] and the mean

disease duration (years) [Control -5.3±3.86; Diet-5.33±3.63] were comparable

between the two groups. There was a slight difference between the two study

groups in the baseline mean expanded disability status scale (EDSS) score

[Control-2.22±0.90; Diet-2.72±1.05]. Eight subjects withdrew (Diet, N=6; Control,

N=2). Adherence to the study diet based on monthly Food Frequency Questionnaire

(FFQ) was excellent with the diet group showing significant difference in the

total fat caloric intake compared to the control group [total fat intake/total

calories averaged ~15% (Diet) versus ~40% (Control)]. The two groups showed no

differences in brain MRI outcomes, number of MS relapses or disability at 12

months. The diet group showed improvements at six months in low-density

lipoprotein cholesterol (Δ=-11.99mg/dL; p=0.031), total cholesterol

(Δ=-13.18mg/dL; p=0.027) and insulin (Δ=-2.82mg/dL; p=0.0067), mean monthly

reductions in BMI (Rate=-1.125kg/m2 per month; p<0.001) and fatigue [FSS

(Rate=-0.0639 points/month; p=0.0010); MFIS (Rate=-0.233 points/month; p=0.0011)]

during the 12-month period.

CONCLUSIONS: While a very-low fat, plant-based diet was well adhered to and

tolerated, it resulted in no significant improvement on brain MRI, relapse rate

or disability as assessed by EDSS scores in subjects with RRMS over one year. The

diet group however showed significant improvements in measures of fatigue, BMI

and metabolic biomarkers. The study was powered to detect only very large effects

on MRI activity so smaller but clinically meaningful effects cannot be excluded.

The diet intervention resulted in a beneficial effect on the self-reported

outcome of fatigue but these results should be interpreted cautiously as a

wait-list control group may not completely control for a placebo effect and there

was a baseline imbalance on fatigue scores between the groups. If maintained, the

improved lipid profile and BMI could yield long-term vascular health benefits.

Longer studies with larger sample sizes are needed to better understand the

long-term health benefits of this diet.

Published by Elsevier B.V.

DOI: 10.1016/j.msard.2016.07.001

PMID: 27645350 [Indexed for MEDLINE]

112. Mult Scler Relat Disord. 2016 Sep;9:47-9. doi: 10.1016/j.msard.2016.06.007. Epub

Excluded b/c no real-world adherence/persistence

2016 Jun 23.

Cryptococcal meningitis after fingolimod discontinuation in a patient with

multiple sclerosis.

Ward MD(1), Jones DE(2), Goldman MD(3).

Author information:

(1)Department of Neurology, University of Virginia, PO Box 800394,

Charlottesville, VA 22908, USA. Electronic address: mdw8u@virginia.edu.

(2)Department of Neurology, University of Virginia, PO Box 800394,

Charlottesville, VA 22908, USA. Electronic address: dj9d@virginia.edu.

(3)Department of Neurology, University of Virginia, PO Box 800394,

Charlottesville, VA 22908, USA. Electronic address: mdg3n@virginia.edu.

Fingolimod (Gilenya, Novartis) is an oral sphingosine-1-phosphate analogue used

in the treatment of relapsing multiple sclerosis (MS). Fingolimod treatment is

associated with relative lymphopenia and was associated with an increased risk of

herpes infection in clinical trials. In the post-marketing setting, fingolimod

has been associated with several cases of cryptococcal meningitis, recently

prompting an update to its prescribing information. To date, all cases have been

associated with active treatment with fingolimod. In this report, we describe the

first case of cryptococcal meningitis diagnosed after fingolimod discontinuation.

Copyright © 2016 Elsevier B.V. All rights reserved.

DOI: 10.1016/j.msard.2016.06.007

PMID: 27645342 [Indexed for MEDLINE]

113. Neurol Sci. 2016 Sep;37(9):1557-9. doi: 10.1007/s10072-016-2621-y. Epub 2016 May

Excluded b/c no real-world adherence/persistence

25.

Mobitz type I and II atrioventricular blocks during fingolimod therapy.

Saccà F(1), Puorro G(2), Marsili A(2), Pane C(2), Russo CV(2), Lanzillo R(2), de

Rosa A(2), Cittadini A(3), De Angelis G(4), Brescia Morra V(2).

Author information:

(1)Department of Neurosciences, Reproductive and Odontostomatological Sciences,

University Federico II, Via Pansini, 5, 80131, Naples, Italy.

francesco.sacca@unina.it.

(2)Department of Neurosciences, Reproductive and Odontostomatological Sciences,

University Federico II, Via Pansini, 5, 80131, Naples, Italy.

(3)Department of Translational Medical Sciences, University Federico II, Naples,

Italy.

(4)U.O.C. Cardiologia e UTIC, ASST Rhodense, Garbagnate Milanese, Italy.

We investigated patients who showed a second-degree atrioventricular block

(S-AVB) after the first fingolimod administration. We observed six patients with

S-AVB, three Mobitz type I, and three type II. Monitoring continued on the second

day for all patients. Three patients showed persistence of the S-AVB, with

resolution on the second or third day. One patient had a persistent S-AVB up to

the fourth day when fingolimod was discontinued. We conclude that Mobitz type II

S-AVB is possible during fingolimod therapy. Patients with S-AVB could be

monitored until resolution of the S-AVBs, as these may persist several days after

the first fingolimod administration.

DOI: 10.1007/s10072-016-2621-y

PMID: 27225279 [Indexed for MEDLINE]

114. Patient Prefer Adherence. 2016 Aug 26;10:1647-56. doi: 10.2147/PPA.S109520.

Excluded b/c no real-world adherence/persistence

eCollection 2016.

Self-reported quality of life in multiple sclerosis patients: preliminary results

based on the Polish MS Registry.

Brola W(1), Sobolewski P(2), Fudala M(1), Flaga S(3), Jantarski K(4), Ryglewicz

D(5), Potemkowski A(6).

Author information:

(1)Department of Neurology, Specialist Hospital, Końskie.

(2)Depsartment of Neurology, Holy Spirit Specialist Hospital, Sandomierz.

(3)AGH University of Science and Technology, Krakow.

(4)Swietokrzyski Regional Branch of the Polish National Health Fund (NFZ),

Kielce.

(5)First Department of Neurology, Institute of Psychiatry and Neurology, Warsaw.

(6)Department of Psychology, University of Szczecin, Szczecin, Poland.

BACKGROUND: The aim of the study was to analyze selected clinical and

sociodemographic factors and their effects on the quality of life (QoL) of

multiple sclerosis (MS) patients registered in the Polish MS Registry.

METHODS: This was a cross-sectional observational study performed in Poland. Data

on personal and disease-specific factors were collected between January 1, 2011,

and December 31, 2015, via the web portal of the Polish MS Registry. All patients

were assessed by a physician and asked to complete the Polish language versions

of the following self-evaluation questionnaires: EuroQol 5-Dimensions, EuroQoL

Visual Analog Scale, and Multiple Sclerosis Impact Scale. Univariate analysis and

logistic regression were performed to determine the factors associated with QoL.

RESULTS: The study included 2,385 patients (female/male ratio 2.3:1) with

clinically confirmed MS (mean age 37.8±9.2 years). Average EuroQol 5-Dimensions

index was 0.72±0.24, and the mean EuroQoL Visual Analog Scale score was

64.2±22.8. The average Multiple Sclerosis Impact Scale score was 84.6±11.2

(62.2±18.4 for physical condition and 23.8±7.2 for mental condition). Lower QoL

scores were significantly associated with higher level of disability (odds ratio

[OR], 0.932; 95% confidence interval [CI], 0.876-0.984; P=0.001), age >40 years

(OR, 1.042; 95% CI, 0.924-1.158; P=0.012), longer disease duration (OR, 0.482;

95% CI, 0.224-0.998; P=0.042), and lack of disease modifying therapies (OR,

0.024; 95% CI, 0.160-0.835; P=0.024). No significant associations were found

between QoL, sex, type of MS course, patient's education, and marital status.

CONCLUSION: The Polish MS Registry is the first national registry for long-term

observation that allows for self-evaluation of the QoL. QoL of Polish patients

with MS is significantly lower compared with the rest of the population. The

parameter is mainly affected by the level of disability, duration of the disease,

and limited access to immunomodulatory therapy.

DOI: 10.2147/PPA.S109520

PMCID: PMC5008638

PMID: 27616882

115. PLoS One. 2016 Aug 25;11(8):e0161701. doi: 10.1371/journal.pone.0161701.

Excluded b/c no real-world adherence/persistence

eCollection 2016.

Associations of Lifestyle, Medication, and Socio-Demographic Factors with

Disability in People with Multiple Sclerosis: An International Cross-Sectional

Study.

Jelinek GA(1), De Livera AM(1), Marck CH(1), Brown CR(1), Neate SL(1), Taylor

KL(1), Weiland TJ(1).

Author information:

(1)Neuroepidemiology Unit, Melbourne School of Population and Global Health, The

University of Melbourne, Melbourne, VIC, Australia.

OBJECTIVE: Emerging evidence links modifiable lifestyle risk factors to disease

progression in multiple sclerosis (MS). We sought further evidence around this

hypothesis through detailed analysis of the association with disability of

lifestyle behaviours of a large international sample of people with MS.

MATERIALS AND METHODS: A total of 2469 people with MS from 57 countries provided

self-reported data via cross-sectional online survey on lifestyle (mostly with

validated tools) and the primary outcome measure, disability (Patient Determined

Disease Steps), categorised from 8 steps into 3 categories, mild, moderate and

major disability. Multinomial logistic regression modelling derived relative risk

ratios (RRRs) for disability categories.

RESULTS: RRRs of having moderate vs mild disability were: diet (per 30 points on

100 point scale) 0.72 (95%CI 0.52-0.98), ever smoking 1.32 (1.06-1.65), exercise

(moderate/high vs low) 0.35 (0.28-0.44), latitude (per degree from the equator)

1.02 (1.01-1.04), and number of comorbidities (2 vs none) 1.43 (1.04-1.95), (3 vs

none) 1.56 (1.13-2.16). RRRs of having major vs mild disability were: exercise

(moderate/high vs low) 0.07 (0.04-0.11), alcohol consumption (moderate vs low)

0.45 (0.30-0.68), plant-based omega 3 supplementation 0.39 (0.18-0.86), and

disease-modifying medication use 0.45 (0.29-0.70).

CONCLUSIONS: Healthier lifestyle has strong associations with disability in our

large international sample of people with MS, supporting further investigation

into the role of lifestyle risk factors in MS disease progression.

DOI: 10.1371/journal.pone.0161701

PMCID: PMC4999178

PMID: 27560626 [Indexed for MEDLINE]

Conflict of interest statement: GJ receives royalties from his books Overcoming

Multiple Sclerosis and Recovering from Multiple Sclerosis. GJ, SN and KT have

received payments for conducting live-in educational workshops for people with

MS. No other authors have conflicts of interest. This does not alter our

adherence to PLOS ONE policies on sharing data and materials.

116. PLoS One. 2016 Aug 15;11(8):e0160313. doi: 10.1371/journal.pone.0160313.

Excluded b/c no oral DMD results

eCollection 2016.

Long-Term Adherence to IFN Beta-1a Treatment when Using RebiSmart® Device in

Patients with Relapsing-Remitting Multiple Sclerosis.

Fernández O(1), Arroyo R(2), Martínez-Yélamos S(3), Marco M(4), Merino JA(5),

Muñoz D(6), Merino E(7), Roque A(7); RELOAD Study Group.

Author information:

(1)Hospitales Universitarios Regional de Málaga y Virgen de la Victoria,

Universidad de Málaga, IBIMA.

(2)Hospital U. Clínico San Carlos, Madrid.

(3)Hospital Universitario de Bellvitge, Barcelona.

(4)Corporació Sanitaria Parc Taulí, Barcelona.

(5)Hospital Universitario Puerta de Hierro, Madrid.

(6)Hospital Xeral Cies, Vigo.

(7)Departamento Médico, Merck S.L.

The effectiveness of disease-modifying drugs in the treatment of multiple

sclerosis is associated with adherence. RebiSmart® electronic device provides

useful information about adherence to the treatment with subcutaneous (sc)

interferon (IFN) β-1a (Rebif®). The aim of the study was to determine long-term

adherence to this treatment in patients with relapsing-remitting multiple

sclerosis (RRMS). This retrospective multicentre observational study analysed 258

patients with RRMS who were receiving sc IFN β-1a (Rebif®) treatment by using

RebiSmart® until replacement (36 months maximum lifetime) or treatment

discontinuation. Adherence was calculated with data (injection dosage, time, and

date) automatically recorded by RebiSmart®. Patients in the study had a mean age

of 41 years with a female proportion of 68%. Mean EDSS score at start of

treatment was 1.8 (95% CI, 1.6-1.9). Overall adherence was 92.6% (95% CI,

90.6-94.5%). A total of 30.2% of patients achieved an adherence rate of 100%,

80.6% at least 90%, and only 13.2% of patients showed a suboptimal adherence

(<80%). A total of 59.9% of subjects were relapse-free after treatment

initiation. Among 106 subjects (41.1%) who experienced, on average, 1.4 relapses,

the majority were mild (40.6%) or moderate (47.2%). Having experienced relapses

from the beginning of the treatment was the only variable significantly related

to achieving an adherence of at least 80% (OR = 3.06, 1.28-7.31). Results of this

study indicate that sc IFN β-1a administration facilitated by RebiSmart® could

lead to high rates of adherence to a prescribed dose regimen over 36 months.

DOI: 10.1371/journal.pone.0160313

PMCID: PMC4985132

PMID: 27526201 [Indexed for MEDLINE]

117. Clin Rheumatol. 2016 Aug;35(8):1985-1991. doi: 10.1007/s10067-016-3300-3. Epub

2016 May 17.

Excluded b/c no real-world adherence/persistence

Outcome of a glucocorticoid discontinuation regimen in patients with inactive

systemic sclerosis.

Iudici M(1), Vettori S(1), Russo B(1), Giacco V(1), Capocotta D(1), Valentini

G(2).

Author information:

(1)Department of Clinical and Experimental Medicine, Rheumatology Section, Second

University of Naples, II Policlinico, Via Pansini 5, 80131, Naples, Italy.

(2)Department of Clinical and Experimental Medicine, Rheumatology Section, Second

University of Naples, II Policlinico, Via Pansini 5, 80131, Naples, Italy.

gabriele.valentini@unina2.it.

Glucocorticoids (GC) are widely used to treat systemic sclerosis (SSc). The lack

of efficacy data and patient/physician concerns may prompt therapy

discontinuation. The aim of this study is to identify factors hampering GC

discontinuation in patients with stable disease on oral GC for longer than

12 months. Consecutive patients fulfilling the 2013 ACR/EULAR criteria for SSc

and with stable disease were prescribed a slow tapering GC regimen to achieve

discontinuation. At study entry and 6 months later (T6), patients were assessed

for disease activity and severity. Moreover, the Short-Form-36; the Health

Assessment Questionnaire Disability Index (HAQ-DI); and visual analog scales for

fatigue, pain, and general health were completed. Reasons for stopping the

discontinuation regimen were recorded. Forty-eight patients (46 females, 9

diffuse SSc), with a mean ± SD age of 56±14 years and a median disease duration

of 10 years (range 2-22), were enrolled. The median daily GC dose was 5 mg (range

5-10; all patients treated with prednisone). At T6, 33 (68.7 %) patients had

discontinued GC. The remaining 15 patients could not discontinue GC because of

arthralgia in eight, arthritis in two, puffy fingers in two, increased

creatine-kinase in two, and bursitis in one patient. At multiple logistic

analysis, a higher baseline HAQ-DI was the only independent factor associated

with GC need (OR 2.98, 95 % CI 1.20-7.41; p = 0.01). About one third of SSc

patients did not achieve a GC-free regimen. Disability as assessed by HAQ-DI was

the leading factor hindering GC discontinuation. A low HAQ-DI score can identify

candidates for GC discontinuation.

DOI: 10.1007/s10067-016-3300-3

PMID: 27184047 [Indexed for MEDLINE]

118. Mult Scler. 2016 Aug;22(9):1235-41. doi: 10.1177/1352458516638558. Epub 2016 Mar

15.

Excluded b/c no real-world adherence/persistence

Simultaneous early-onset immune thrombocytopenia and autoimmune thyroid disease

following alemtuzumab treatment in relapsing-remitting multiple sclerosis.

Obermann M(1), Ruck T(2), Pfeuffer S(2), Baum J(3), Wiendl H(2), Meuth SG(2).

Author information:

(1)Center for Neurology, Asklepios Hospitals Schildautal, Seesen,

Germany/Departments of Neurology and Haematology, University of Duisburg-Essen,

Duisburg, Germany mark.obermann@uni-due.de.

(2)Department of Neurology, University of Münster, Münster, Germany.

(3)Departments of Neurology and Haematology, University of Duisburg-Essen,

Duisburg, Germany.

OBJECTIVE: We report two cases of patients with relapsing-remitting multiple

sclerosis with early-onset thrombocytopenia and autoimmune thyroid disease after

the first treatment course with 60-mg alemtuzumab.

METHODS: Case series and review of the literature.

RESULTS: Both patients showed severe thrombocytopenia with platelet counts of

2 × 10(9) and 11 × 10(9)/L, respectively, as well as increased thyroid antibodies

within only a few months after initiating alemtuzumab treatment (11 and

9 months). Both patients responded considerably well to medical therapy including

corticosteroids and intravenous immunoglobulins with slow platelet recovery over

several weeks. Interestingly, both patients were previously treated with

fingolimod and showed a marked lymphocytopenia that led to discontinuation.

CONCLUSION: These cases emphasize the necessity of careful clinical surveillance

and proper education of patients treated with alemtuzumab as proposed by the

safety-monitoring program. Previous severe lymphocytopenia under therapy with

other disease-modifying therapies may be a risk factor for the development of

immune thrombocytopenia.

© The Author(s), 2016.

DOI: 10.1177/1352458516638558

PMID: 26980848 [Indexed for MEDLINE]

119. Neuromodulation. 2016 Aug;19(6):607-15. doi: 10.1111/ner.12447. Epub 2016 Jul 19.

Excluded b/c no real-world adherence/persistence

Best Practices for Intrathecal Baclofen Therapy: Patient Selection.

Saulino M(1), Ivanhoe CB(2)(3), McGuire JR(4), Ridley B(5), Shilt JS(6), Boster

AL(7).

Author information:

(1)Intrathecal Therapy Services, MossRehab, Elkins Park, PA, USA.

(2)Baylor College of Medicine, Houston, TX, USA.

(3)Mentis Neuro Health Brain Injury and Stroke Program at TIRR-Memorial Hermann,

Houston, TX, USA.

(4)Physical Medicine and Rehabilitation, Medical College of Wisconsin, Milwaukee,

WI, USA.

(5)Spasticity Management Program, Alta Bates Summit Medical Center, Berkeley, CA,

USA.

(6)Department of Orthopedic and Scoliosis Surgery, Baylor College of Medicine and

Texas Children's Hospital, Houston, TX, USA.

(7)Systems Medical Chief, Neuroimmunology, OhioHealth Multiple Sclerosis Program,

Columbus, OH, USA.

INTRODUCTION: When spasticity interferes with comfort, function, activities of

daily living, mobility, positioning, or caregiver assistance, patients should be

considered for intrathecal baclofen (ITB) therapy.

METHODS: An expert panel consulted on best practices.

RESULTS: ITB can be considered for problematic spasticity involving

muscles/muscle groups during all phases of diseases, including progressive

neurologic diseases. ITB alone or with other treatments should not be exclusively

reserved for individuals who have failed other approaches. ITB combined with

rehabilitation can be effective in certain ambulatory patients. ITB is also

highly effective in managing spasticity in children, who may suffer limb

deformity, joint dislocation, and poor motor function from spasticity and muscle

tightness on the growing musculoskeletal system. Spasticity management often

allows individuals to achieve higher function. When cognition is impaired, ITB

controls spasticity without the cognitive side effects of some oral medications.

Goal setting addresses expectations and treatment in the framework of pathology,

impairment, and disability. ITB is contraindicated in patients with

hypersensitivity to baclofen, which is rare, or active infection. Some patients

with an adverse reaction to oral baclofen may be mistakenly classified as having

an allergic reaction and may benefit from ITB. Relative contraindications include

unrealistic goals, unmanageable mental health issues, psychosocial factors

affecting compliance, and financial burden. Vascular shunting for hydrocephalus

is not a contraindication, but concurrent use may affect cerebrospinal fluid

flow. Seizures or prior abdominal or pelvic surgery should be discussed before

proceeding to an ITB screening test.

CONCLUSIONS: ITB should be considered when spasticity interferes with comfort or

function.

© 2016 International Neuromodulation Society.

DOI: 10.1111/ner.12447

PMID: 27434197 [Indexed for MEDLINE]

120. Medicine (Baltimore). 2016 Jul;95(29):e4180. doi: 10.1097/MD.0000000000004180.

Excluded b/c no real-world adherence/persistence

Improvement of macular edema without discontinuation of fingolimod in a patient

with multiple sclerosis: A case report.

Akiyama H(1), Suzuki Y, Hara D, Shinohara K, Ogura H, Akamatsu M, Hasegawa Y.

Author information:

(1)Department of Neurology, St. Marianna University School of Medicine, Kawasaki,

Kanagawa, Japan.

INTRODUCTION: Generally, fingolimod administration is simply discontinued when

fingolimod-associated macular edema (ME) appears, and the majority of cases are

said to recover spontaneously. However, to the best of our knowledge, this is the

1st report regarding improvement of ME without discontinuation of fingolimod

administration.

CASE PRESENTATION: The patient was a 66-year-old woman with relapsing-remitting

multiple sclerosis. She was started on treatment with fingolimod to prevent

recurrence, after which she developed ME that was probably due to fingolimod. The

patient expressed a strong fear of recurrence if fingolimod was discontinued, so

we continued fingolimod therapy and followed up the patient frequently. The ME

improved after approximately 1 year without any need for concomitant treatment.

CONCLUSION: We believe that the continuation of fingolimod therapy with strict

follow-up examination is one option for treatment, though strategies for managing

rapid deterioration of ME should be borne in mind.

DOI: 10.1097/MD.0000000000004180

PMCID: PMC5265758

PMID: 27442641 [Indexed for MEDLINE]

Conflict of interest statement: The authors have no funding and conflicts of

interest to disclose.

121. Mult Scler Relat Disord. 2016 Jul;8:78-85. doi: 10.1016/j.msard.2016.05.006. Epub

Excluded b/c no oral DMD results

2016 May 7.

Adherence and persistence to drug therapies for multiple sclerosis: A

population-based study.

Evans C(1), Marrie RA(2), Zhu F(3), Leung S(4), Lu X(5), Melesse DY(6), Kingwell

E(7), Zhao Y(8), Tremlett H(9).

Author information:

(1)College of Pharmacy & Nutrition, University of Saskatchewan, 104 Clinic Place,

Saskatoon, SK S7N 5E5, Canada. Electronic address: charity.evans@usask.ca.

(2)Departments of Internal Medicine and Community Health Sciences, University of

Manitoba, Health Sciences Centre, GF 543-820 Sherbrook Street, Winnipeg, MB,

Canada R3A 1R9. Electronic address: rmarrie@hsc.mb.ca.

(3)Department of Medicine (Neurology), University of British Columbia, UBC

Hospital, 2211 Wesbrook Mall, Vancouver, BC, Canada V6T 2B5. Electronic address:

f.zhu@stat.ubc.ca.

(4)Department of Community Health Sciences, University of Manitoba, Health

Sciences Centre, GF 543-820 Sherbrook Street, Winnipeg, MB, Canada R3A 1R9.

Electronic address: stellaleung1@gmail.com.

(5)Saskatchewan Health Quality Council, 241-111 Research Drive, Saskatoon, SK,

Canada S7N 3R2. Electronic address: xlu@hqc.sk.ca.

(6)Department of Community Health Sciences, University of Manitoba, Health

Sciences Centre, GF 543-820 Sherbrook Street, Winnipeg, MB, Canada R3A 1R9.

Electronic address: desyiz@gmail.com.

(7)Department of Medicine (Neurology), University of British Columbia, UBC

Hospital, 2211 Wesbrook Mall, Vancouver, BC, Canada V6T 2B5. Electronic address:

elainejk@mail.ubc.ca.

(8)Department of Medicine (Neurology), University of British Columbia, UBC

Hospital, 2211 Wesbrook Mall, Vancouver, BC, Canada V6T 2B5. Electronic address:

yinshan@msmri.medicine.ubc.ca.

(9)Department of Medicine (Neurology), University of British Columbia, UBC

Hospital, 2211 Wesbrook Mall, Vancouver, BC, Canada V6T 2B5. Electronic address:

helen.tremlett@ubc.ca.

OBJECTIVE: We aimed to estimate the prevalence and predictors of optimal

adherence and persistence to the disease-modifying therapies (DMT) for multiple

sclerosis (MS) in 3 Canadian provinces.

METHODS: We used population-based administrative databases in British Columbia

(BC), Saskatchewan, and Manitoba. All individuals receiving DMT (interferon-B-1b,

interferon-B-1a, and glatiramer acetate) between 1-January-1996 and

31-December-2011 (BC), 31-March-2014 (Saskatchewan), or 31-March-2012 (Manitoba)

were included. One-year adherence was estimated using the proportion of days

covered (PDC). Persistence was defined as time to DMT discontinuation. Regression

models were used to assess predictors of adherence and persistence; results were

pooled using random effects meta-analysis.

RESULTS: 4830 individuals were included. When results were combined, an estimated

76.4% (95% CI: 69.1-82.4%) of subjects exhibited optimal adherence (PDC ≥80%).

Median time to discontinuation of the initial DMT was 1.9 years (95% CI: 1.6-2.1)

in Manitoba, 2.8 years (95% CI: 2.5-3.0) in BC, and 4.0 years (95% CI: 3.5-4.6)

in Saskatchewan. Age, sex and socioeconomic status were not associated with

adherence or persistence. Individuals who had ≥4 physician visits during the year

prior to the first DMT dispensation were more likely to exhibit optimal adherence

compared to those with fewer (0-3) physician visits.

CONCLUSIONS: We observed adherence that is higher than what has been reported for

other chronic diseases, and other non-population-based MS cohorts. Closer

examination as to why adherence appears to be relatively better in MS and how

adherence influences disease outcomes could contribute to our understanding of

MS, and prove useful in the management of other chronic diseases.

Copyright © 2016 The Authors. Published by Elsevier B.V. All rights reserved.

DOI: 10.1016/j.msard.2016.05.006

PMID: 27456879 [Indexed for MEDLINE]

122. Ther Adv Neurol Disord. 2016 Jul;9(4):250-63. doi: 10.1177/1756285616634247. Epub

Excluded b/c no oral DMD results

2016 Mar 15.

Therapy satisfaction and adherence in patients with relapsing-remitting multiple

sclerosis: the THEPA-MS survey.

Haase R(1), Kullmann JS(2), Ziemssen T(3).

Author information:

(1)Center of Clinical Neuroscience, Neurological Clinic, University Clinic Carl

Gustav Carus Dresden, TU Dresden, Germany.

(2)Genzyme, a Sanofi company, Neu-Isenburg, Germany.

(3)Multiple Sklerose Zentrum, Zentrum für klinische Neurowissenschaften,

Universitätsklinik Carl Gustav Carus, Technische Universität Dresden,

Fetscherstr. 74, 01307 Dresden, Germany.

BACKGROUND: Improved clinical effectiveness and therefore positive modification

of multiple sclerosis (MS) with basic therapy can be achieved by long-term

regular intake of drugs as prescribed but investigations have shown that a high

percentage of patients do not take their medications as prescribed.

OBJECTIVES: We assessed the satisfaction and adherence of patients with MS with

their current disease-modifying treatment under clinical practice conditions. We

compared different facets of satisfaction as well as their internal relationship

and identified predictors in an exploratory manner.

METHODS: Therapy satisfaction in patients with relapsing-remitting multiple

sclerosis (THEPA-MS) was a noninterventional, prospective cross-sectional study

performed throughout Germany in 2013 and 2014, and included patients with

clinically isolated syndrome or relapsing-remitting MS. We applied a standardized

approach to document satisfaction and adherence by patient-reported outcomes

(Treatment Satisfaction Questionnaire for Medication) as well as by physician

ratings.

RESULTS: Of 3312 patients with a mean age of 43.7 years, 73.3% were women and the

mean level of disability according to the Expanded Disability Status Scale was

2.29; 13.3% did not receive any medication at the time of documentation, 21.3%

received interferon β1a intramuscularly, 20.7% had interferon β1a subcutaneously,

17.0% had interferon β1b subcutaneously and 23.7% had glatiramer acetate.

Adherence rates varied between 60% (lifetime) and 96.5% (current medication).

Differences between current medications were found for side effects and

convenience scores but not for effectiveness, satisfaction and adherence. Higher

global satisfaction and effectiveness were associated with fewer relapses, longer

duration of medication, lower disability score and the absence of several side

effects.

CONCLUSION: In a connected model of patient satisfaction, effectiveness, side

effects, convenience and adherence, patients' individual needs and concerns have

to be addressed. Most differences were found with respect to side effects and

convenience of treatment. Therefore, an improvement in these two domains seems to

be the most promising proximate approach to elevate adherence levels.

DOI: 10.1177/1756285616634247

PMCID: PMC4916516

PMID: 27366231

123. Am J Health Syst Pharm. 2016 Jun 1;73(11):811-9. doi: 10.2146/ajhp150723. Epub

Excluded b/c no primary data (narrative review)

2016 Apr 28.

Management of multiple sclerosis and the integration of related specialty

pharmacy programs within health systems.

Habibi M(1), Kuttab HM(2).

Author information:

(1)Department of Pharmacy Practice, University of Illinois at Chicago, Chicago,

IL mhabib1@uic.edu.

(2)Rush University Medical Center, Chicago, IL.

PURPOSE: The management of multiple sclerosis (MS) and the integration of related

specialty pharmacy programs within health systems are discussed.

SUMMARY: MS is a progressive immune-mediated inflammatory disease of the central

nervous system. Current treatment strategies include the use of disease-modifying

therapies (DMTs) that have various degrees of efficacy and tolerability. These

DMTs also differ with respect to frequency and route of administration, which can

significantly impact patient compliance and ultimately their response to therapy.

The introduction of oral and injectable DMTs requiring less-frequent injections

and having better adverse-effect profiles may help patients improve adherence to

therapy; however, access to these therapies is often restricted due to both their

high cost and limited distribution. These DMTs include fingolimod, teriflunomide,

dimethyl fumarate, and pegylated interferon beta-1a. All others, with the

exception of fingolimod, have limited distribution. Pharmacists in health-system

pharmacy programs can play a significant role in assisting patients with MS

manage their disease efficiently and safely by educating them about their

therapies, ensuring compliance with the associated risk evaluation and mitigation

strategy (REMS) program, and helping them access these therapies in a timely

manner.

CONCLUSION: MS is a progressive neurologic disorder that requires lifelong

treatment with DMTs. Good compliance, compliance with the associated REMS

program, and timely access to these drugs may positively influence patient care

and outcomes and provide an opportunity for the health-system pharmacists to have

a active role in caring for these patients.

Copyright © 2016 by the American Society of Health-System Pharmacists, Inc. All

rights reserved.

DOI: 10.2146/ajhp150723

PMID: 27126827 [Indexed for MEDLINE]

124. Ann Neurol. 2016 Jun;79(6):950-8. doi: 10.1002/ana.24651. Epub 2016 Apr 20.

Excluded b/c no real-world adherence/persistence

Rituximab versus fingolimod after natalizumab in multiple sclerosis patients.

Alping P(1), Frisell T(2), Novakova L(3), Islam-Jakobsson P(4), Salzer J(4),

Björck A(1), Axelsson M(3), Malmeström C(3), Fink K(1), Lycke J(3), Svenningsson

A(5)(4), Piehl F(1).

Author information:

(1)Department of Clinical Neuroscience, Karolinska Institute, Stockholm, Sweden.

(2)Department of Medicine Solna, Karolinska Institute, Stockholm, Sweden.

(3)Department of Neurology, Institute of Clinical Neuroscience and Physiology,

Sahlgrenska Academy at the University of Gothenburg, Gothenburg, Sweden.

(4)Department of Pharmacology and Clinical Neuroscience, Umeå University, Umeå,

Sweden.

(5)Department of Clinical Sciences, Danderyd Hospital, Karolinska Institutet,

Stockholm, Sweden.

OBJECTIVE: Many JC virus antibody-positive relapsing-remitting multiple sclerosis

(RRMS) patients who are stable on natalizumab switch to other therapies to avoid

progressive multifocal leukoencephalopathy.

METHODS: We compared outcomes for all RRMS patients switching from natalizumab

due to JC virus antibody positivity at 3 Swedish multiple sclerosis centers with

different preferential use of rituximab and fingolimod (Stockholm, n = 156,

fingolimod 51%; Gothenburg, n = 64, fingolimod 88%; Umeå, n = 36, fingolimod

19%), yielding a total cohort of N = 256 (fingolimod 55%).

RESULTS: Within 1.5 years of cessation of natalizumab, 1.8% (rituximab) and 17.6%

(fingolimod) of patients experienced a clinical relapse (hazard ratio for

rituximab = 0.10, 95% confidence interval [CI] = 0.02-0.43). The hazard ratio

(favoring rituximab) for adverse events (5.3% vs 21.1%) and treatment

discontinuation (1.8% vs 28.2%) were 0.25 (95% CI = 0.10-0.59) and 0.07 (95% CI =

0.02-0.30), respectively. Furthermore, contrast-enhancing lesions were found in

1.4% (rituximab) versus 24.2% (fingolimod) of magnetic resonance imaging

examinations (odds ratio = 0.05, 95% CI = 0.00-0.22). Differences remained when

adjusting for possible confounders (age, sex, disability status, time on

natalizumab, washout time, follow-up time, and study center).

INTERPRETATION: Our findings suggest an improved effectiveness and tolerability

of rituximab compared with fingolimod in stable RRMS patients who switch from

natalizumab due to JC virus antibody positivity. Although residual confounding

factors cannot be ruled out, the shared reason for switching from natalizumab and

the preferential use of either rituximab or fingolimod in 2 of the centers

mitigates these concerns. Ann Neurol 2016;79:950-958.

© 2016 American Neurological Association.

DOI: 10.1002/ana.24651

PMID: 27038238 [Indexed for MEDLINE]

125. Continuum (Minneap Minn). 2016 Jun;22(3):851-63. doi:

Excluded b/c no primary data (narrative review)

10.1212/CON.0000000000000327.

Switching or Discontinuing Disease-Modifying Therapies for Multiple Sclerosis.

Miller AE.

PURPOSE OF REVIEW: This article reviews the reasons for discontinuation or

switching of multiple sclerosis disease-modifying therapy as well as procedures

that might mitigate risk to the patient under such circumstances.

RECENT FINDINGS: Recent review of the literature, as well as the author's

extensive clinical experience, indicate that the discontinuation of multiple

sclerosis disease-modifying therapies occurs for many reasons. Often one

medication is stopped at the recommendation of the physician in order to switch

to another medication. However, often the decision to discontinue medication is

made by the patient. Unfortunately, in still other situations, treatment is

stopped because of circumstances beyond the control of either patient or

physician (eg, a loss of insurance coverage). Currently available data do not

permit a conclusion about whether it is ever safe to discontinue

disease-modifying therapy in a stable patient without the expectation of return

of disease activity.

SUMMARY: Clinicians must help patients avoid unnecessary and undesirable

cessation of disease-modifying therapy. While switches of therapy are often

necessary, steps to minimize both adverse events and the risk of recurrent

disease should be undertaken. Whether disease-modifying therapy can ever be

purposely discontinued without incurring a significant risk of disease recurrence

remains to be determined.

DOI: 10.1212/CON.0000000000000327

PMID: 27261686 [Indexed for MEDLINE]

126. Curr Opin Neurol. 2016 Jun;29(3):272-7. doi: 10.1097/WCO.0000000000000320.

Excluded b/c no primary data (narrative review)

Update on monitoring and adverse effects of first generation disease modifying

therapies and their recently approved versions in relapsing forms of multiple

sclerosis.

Dubey D(1), Cano CA, Stüve O.

Author information:

(1)Department of Neurology and Neurotherapeutics, University of Texas

Southwestern Medical Center, Dallas, Texas, USA.

PURPOSE OF REVIEW: As of April 2015, 13 disease modifying therapies (DMTs) have

been approved by the Food and Drug Administration. The older agents continue to

be utilized across the globe, especially in developing countries where many newer

DMTs are still not available. Even though first generation DMTs have modest

efficacy they have long term safety profile, and are considered safer than the

second generation DMTs.

RECENT FINDINGS: A PEGylated interferon beta-1a preparation that is administered

subcutaneously every 2 weeks was also recently approved. Less frequent

administration potentially reduced administration associated side effects and may

improve adherence and compliance. The polyethylene glycol is also thought to make

the drug less immunogenic. Glatopa (a glatiramer acetate bioequivalent), now

represents the first available generic alternative of a DMT for multiple

sclerosis. Its dosing, route of administration, and side effects are the same as

for Copaxone.

SUMMARY: In this article, we review the potential adverse effects and recommended

laboratory studies as part of the monitoring strategy following initiation of

various first generation DMTs and their recently approved versions.

DOI: 10.1097/WCO.0000000000000320

PMID: 27035896 [Indexed for MEDLINE]

127. Expert Opin Drug Deliv. 2016 Jun;13(6):799-805. doi:

Excluded b/c no oral DMD results

10.1517/17425247.2016.1158161. Epub 2016 Mar 12.

Exploratory analysis of predictors of patient adherence to subcutaneous

interferon beta-1a in multiple sclerosis: TRACER study.

Paolicelli D(1), Cocco E(2), Di Lecce V(1), Direnzo V(1), Moiola L(3), Lanzillo

R(4), Perini P(5), Malucchi S(6), Borriello G(7), Portaccio E(8), Panetta V(9),

Fenu G(2), Sangalli F(3), Cacciaguerra L(5), Trojano M(1); TRACER Group.

Author information:

(1)a Department of Basic Medical Sciences, Neurosciences and Sense Organs ,

University of Bari Aldo Moro , Bari , Italy.

(2)b Multiple Sclerosis Centre, Department of Public Health, Clinical Molecular

Medicine , University of Cagliari , Cagliari , Italy.

(3)c Department of Neurology, San Raffaele Scientific Institute , University of

Milan , Milan , Italy.

(4)d Department of Neurosciences, Reproductive and Odontostomatological Sciences

, Federico II University - School of Medicine , Naples , Italy.

(5)e Multiple Sclerosis Centre of the Veneto Region, Department of Neurosciences

, University Hospital of Padua , Padua , Italy.

(6)f Neurologia 2 - CRESM , A.O.U. San Luigi Gonzaga , Orbassano , Italy.

(7)g Multiple Sclerosis Centre, Department of Neurology and Psychiatry, S. Andrea

Hospital , Sapienza University , Rome , Italy.

(8)h Department of NEUROFARBA , University of Florence , Florence , Italy.

(9)i L'altrastatistica srl - Consultancy & Training , Biostatistics office , Rome

, Italy.

OBJECTIVE: The TRACER multicenter retrospective study aimed to collect data on

treatment adherence in a real-life setting, in order to identify predictors of

adherence at baseline.

METHODS: We recruited 384 relapsing-remitting (RR) multiple sclerosis patients

with at least 12 months of use of RebiSmart®. This electronic device records the

performed injections and assesses adherence as the percentage of 'not missing

doses', through the connection to the iMed database. Subjects with at least 80%

of completed doses at the 12 month of therapy were defined 'treatment adherents'.

RESULTS: After 12 months, 89.3% of patients were adherent; 93.2% of patients aged

26-40 years at baseline were adherent (vs 79% of the ≤25 and 87.5% of the

≥41 year olds; p = 0.006). Furthermore, 90.5% of patients with a baseline

Expanded Disability Status Scale (EDSS) score <4 showed ≥80% adherence (vs 71.4%

in those with EDSS score ≥4; p = 0.016). Fifty-four percent of the patients who

were not adherent after 3 months were also not adherent after 12 months (OR 16.8;

CI 95%:7.1-39.8).

CONCLUSIONS: Patients aged 26-40 years and with an EDSS score <4 at baseline were

the most adherent. The status of 'treatment adherent' in the first 3 months was

predictive of higher adherence in the long term.

DOI: 10.1517/17425247.2016.1158161

PMID: 26922837 [Indexed for MEDLINE]

Excluded b/c no primary data (narrative review)

128. Expert Opin Drug Metab Toxicol. 2016 Jun;12(6):701-9. doi:

10.1080/17425255.2016.1179279. Epub 2016 May 2.

Laquinimod in the treatment of relapsing remitting multiple sclerosis.

Hainke U(1), Thomas K(1), Ziemssen T(1).

Author information:

(1)a Department of Neurology, Multiple Sclerosis Center, Center of Clinical

Neuroscience, University Hospital Carl Gustav Carus , Dresden University of

Technology , Dresden , Germany.

INTRODUCTION: Laquinimod is a new once-daily oral administrable agent, which is

under investigation in a phase 3 clinical trial for relapsing remitting multiple

sclerosis (RRMS) and in a phase 2 clinical trial for primary progressive MS

(PPMS).

AREAS COVERED: The pharmacokinetic, pharmacodynamic and the safety profiles of

laquinimod are covered in this review. In preclinical studies, the ability to

prevent both experimental autoimmune encephalomyelitis and experimental

autoimmune neuritis has been demonstrated. Reduced cell infiltration,

demyelination, axonal damage and a shift of T-helper cell responses have been

shown. Accordingly, in human studies, a decrease of pro-inflammatory and an

increase of anti-inflammatory cytokines have been measured and a significant

reduction of disease progression and a decrease in brain volume loss has been

demonstrated. During all clinical studies a favorable safety profile was observed

for 0.6mg laquinimod. New information about cardiovascular events is prompting

the discontinuation of higher dosing regimens in both ongoing trials.

EXPERT OPINION: Laquinimod is a first in class oral agent with high potential to

reduce disease progression in RRMS and PPMS. Owing to its favorable safety

profile, a combination with 0.6mg laquinimod and other disease modifying

therapies could be an option in future MS therapy.

DOI: 10.1080/17425255.2016.1179279

PMID: 27089834 [Indexed for MEDLINE]

129. Neurol Clin Pract. 2016 Jun;6(3):220-229.

Excluded b/c no real-world adherence/persistence

Characterizing absolute lymphocyte count profiles in dimethyl fumarate-treated

patients with MS: Patient management considerations.

Fox RJ(1), Chan A(1), Gold R(1), Phillips JT(1), Selmaj K(1), Chang I(1), Novas

M(1), Rana J(1), Marantz JL(1).

Author information:

(1)Mellen Center for Multiple Sclerosis Treatment and Research (RJF), Cleveland

Clinic, Cleveland, OH; St. Josef Hospital (AC, RG), Ruhr University, Bochum,

Germany; Multiple Sclerosis Program (JTP), Baylor Institute for Immunology

Research, Dallas, TX; Medical University of Lodz (KS), Lodz, Poland; and Biogen

(IC, MN, JR, JLM), Cambridge, MA. Dr. Novas is currently with Alexion

Pharmaceuticals, Chesire, CT; and Dr. Rana is currently with Sanofi-Genzyme,

Cambridge, MA.

BACKGROUND: Delayed-release dimethyl fumarate (DMF), indicated for the treatment

of patients with relapsing-remitting multiple sclerosis (MS), is a

disease-modifying therapy with potential immunomodulatory and neuroprotective

effects. In clinical trials, DMF was associated with reduced white blood cell and

absolute lymphocyte counts. Current US prescribing information recommends

obtaining a complete blood count, including absolute lymphocyte count (ALC),

before initiating and during DMF treatment.

METHODS: We conducted an integrated analysis of phase 2b/3/long-term extension

studies of DMF in MS (N = 2,470) to characterize ALC profiles.

RESULTS: Mean ALCs decreased by 30% during the first year and then plateaued,

remaining above the lower limit of normal (LLN). Among patients treated ≥6 months

(N = 2,099), 2.2% experienced ALCs <500 mm3 persisting ≥6 months. ALCs remained

≥LLN in 84% and 76% of patients during the first 6 and 12 months, respectively;

of these, 0.1% and 0%, respectively, developed ALCs <500 mm3 persisting ≥6 months

at any time. Evidence of ALC improvement following DMF discontinuation was

observed. DMF efficacy was not substantially different in patients with and

without lymphopenia.

CONCLUSION: Lymphocyte monitoring provides effective means for early

identification of patients at risk for developing severe, prolonged lymphopenia.

DOI: 10.1212/CPJ.0000000000000238

PMCID: PMC4909524

PMID: 27347439

130. Thyroid. 2016 Jun;26(6):860-3. doi: 10.1089/thy.2015.0664. Epub 2016 Apr 28.

Excluded b/c no real-world adherence/persistence

Misdiagnosis of Graves' Disease with Apparent Severe Hyperthyroidism in a Patient

Taking Biotin Megadoses.

Barbesino G(1).

Author information:

(1)Thyroid Unit, Massachusetts General Hospital-Harvard Medical School , Boston,

Massachusetts.

BACKGROUND: Accurate immunoassays measuring minute quantities of hormones are the

cornerstone of the practice of endocrinology. Despite tremendous advances in this

field, novel pitfalls in these tests emerge from time to time. Oral biotin can

interfere with immunoassays of several hormones. The purpose of this report is to

relate an extreme case of such interference.

PATIENT FINDINGS: A patient with progressive multiple sclerosis was found to have

extremely elevated free thyroxine, triiodothyronine, and suppressed thyrotropin

(TSH) levels. His TSH receptor binding inhibiting antibody level was also

elevated. This constellation of laboratory findings suggested a diagnosis of

severe Graves' disease. All of the assays yielding abnormal results employed the

biotin-streptavidin affinity in their design. The patient had no symptoms of

hyperthyroidism, and detailed review of his medications revealed intake of

megadoses of biotin. Temporary discontinuation of biotin treatment resulted in

complete resolution of the biochemical abnormalities.

CONCLUSIONS: Non-physiologic biotin supplementation may interfere with several

immunoassays, including thyroid hormones, TSH, thyroglobulin, and TSH receptor

binding inhibiting antibody, leading to erroneous diagnoses. Questioning for

biotin intake should be part of the evaluation for patients undergoing endocrine

tests. Interruption of biotin supplementation for at least two days prior to

biotin-sensitive tests should be sufficient to avoid major misdiagnoses.

DOI: 10.1089/thy.2015.0664

PMID: 27043844 [Indexed for MEDLINE]

131. Mult Scler. 2016 May;22(6):753-60. doi: 10.1177/1352458515600248. Epub 2015 Aug

18.

Excluded b/c no real-world adherence/persistence

Multiple sclerosis patients have a diminished serologic response to vitamin D

supplementation compared to healthy controls.

Bhargava P(1), Steele SU(1), Waubant E(2), Revirajan NR(2), Marcus J(2), Dembele

M(1), Cassard SD(1), Hollis BW(3), Crainiceanu C(4), Mowry EM(5).

Author information:

(1)Department of Neurology, Johns Hopkins University, Baltimore, MD, USA.

(2)Department of Neurology, University of California San Francisco, San

Francisco, CA, USA.

(3)Department of Pediatrics, Medical University of South Carolina, Charleston,

SC, USA.

(4)Department of Biostatistics, Johns Hopkins School of Public Health, Baltimore,

MD, USA.

(5)Department of Neurology, Johns Hopkins University, Baltimore, MD, USA

emowry1@jhmi.edu.

BACKGROUND: Vitamin D insufficiency is a risk factor for multiple sclerosis (MS),

and patients do not always show the expected response to vitamin D

supplementation.

OBJECTIVE: We aimed to determine if vitamin D supplementation leads to a similar

increase in serum 25-hydroxyvitamin-D (25(OH)D) levels in patients with MS and

healthy controls (HCs).

METHODS: Participants in this open-label study were female, white, aged 18-60

years, had 25(OH)D levels ⩽ 75 nmol/l at screening, and had relapsing-remitting

MS (RRMS) or were HCs. Participants received 5000 IU/day of vitamin D3 for 90

days. Utilizing generalized estimating equations we examined the relationship

between the primary outcome (serum 25(OH)D level) and the primary (MS versus HC

status) and secondary predictors.

RESULTS: For this study 27 MS patients and 30 HCs were enrolled. There was no

significant difference in baseline 25(OH)D level or demographics except for

higher body mass index (BMI) in the MS group (25.3 vs. 23.6 kg/m(2), p=0.035). In

total, 24 MS subjects and 29 HCs completed the study. In a multivariate model

accounting for BMI, medication adherence, and oral contraceptive use, MS patients

had a 16.7 nmol/l (95%CI: 4.2, 29.2, p=0.008) lower increase in 25(OH)D levels

compared with HCs.

CONCLUSIONS: Patients with MS had a lower increase in 25(OH)D levels with

supplementation, even after accounting for putative confounders.

© The Author(s), 2015.

DOI: 10.1177/1352458515600248

PMCID: PMC4758937

PMID: 26286698 [Indexed for MEDLINE]

132. Mult Scler Relat Disord. 2016 May;7:53-7. doi: 10.1016/j.msard.2016.03.004. Epub

Excluded b/c no real-world adherence/persistence

2016 Mar 16.

Fingolimod-induced leukoencephalopathy in a patient with neuromyelitis optica

spectrum disorder.

Yoshii F(1), Moriya Y(2), Ohnuki T(2), Ryo M(2), Takahashi W(2).

Author information:

(1)Department of Neurology, Tokai University Oiso Hospital, 21-1 Gakkyou, Oiso,

Naka-gun, Kanagawa 259-0198, Japan. Electronic address:

yoshii@is.icc.u-tokai.ac.jp.

(2)Department of Neurology, Tokai University Oiso Hospital, 21-1 Gakkyou, Oiso,

Naka-gun, Kanagawa 259-0198, Japan.

Fingolimod (FTY720) is used for reducing the annualized relapse rate and slowing

progression of neurological disability in relapsing-remitting forms of multiple

sclerosis (MS). However, its safety is not confirmed in patients with

neuromyelitis optica spectrum disorder (NMOSD), who characteristically have

positive aquaporin-4 (AQP-4) antibody. A 54-year-old female with a

relapsing-remitting course of optic neuritis and myelitis for six years,

diagnosed initially as MS, had been treated with interferon beta-1b and oral

corticosteroid. Magnetic resonance imaging (MRI) consistently revealed lesions on

the optic nerve and spinal cord, but never on the brainstem or cerebral white

matter during acute exacerbation. After treatment was switched to fingolimod from

interferon beta-1b, multiple new lesions appeared at the brainstem and cerebral

white matter. Following discontinuation of fingolimod, these lesions completely

cleared, concomitantly with clinical improvement. During fingolimod treatment,

she was recognized to be positive for AQP-4 antibody. Fingolimod may be

contraindicated in patients with NMOSD.

Copyright © 2016 Elsevier B.V. All rights reserved.

DOI: 10.1016/j.msard.2016.03.004

PMID: 27237757 [Indexed for MEDLINE]

133. Patient Prefer Adherence. 2016 Apr 26;10:659-67. doi: 10.2147/PPA.S106155.

Excluded b/c no real-world adherence/persistence

eCollection 2016.

Perspectives and experiences of Dutch multiple sclerosis patients and multiple

sclerosis-specialized neurologists on injectable disease-modifying treatment.

Visser LH(1), Heerings MA(2), Jongen PJ(3), van der Hiele K(4).

Author information:

(1)Department of Neurology, Elisabeth-TweeSteden Hospital, Tilburg, the

Netherlands; Ethics of Care, University of Humanistic Studies, Utrecht, the

Netherlands.

(2)National Multiple Sclerosis Foundation, Rotterdam, the Netherlands.

(3)Department of Community and Occupational Medicine, University Medical Center

Groningen, University of Groningen, Groningen, the Netherlands; MS4 Research

Institute, Nijmegen, the Netherlands.

(4)Department of Neurology, Elisabeth-TweeSteden Hospital, Tilburg, the

Netherlands; National Multiple Sclerosis Foundation, Rotterdam, the Netherlands;

Section Health, Medical and Neuropsychology, Department of Psychology, Leiden

University, Leiden, the Netherlands.

BACKGROUND: The adherence to treatment with injectable disease-modifying drugs

(DMDs) in multiple sclerosis (MS) may benefit from adequate information provision

and management of expectations. The communication between patients and physicians

is very important in this respect. The current study investigated the

perspectives and experiences of the MS patients and neurologists concerning the

choice and course of treatment with DMDs in the Netherlands.

METHODS: The MS patients (aged 18-60 years; diagnosed with MS at least a year

ago, currently treated with injectable DMD treatment) and MS-specialized

neurologists (practicing for ≥3 years, treating ≥15 MS patients/month on average,

and spending >60% of their time in clinical practice) were asked to complete

semistructured Internet-based questionnaires. The neurologists in this study were

not necessarily the treating neurologists of the participating MS patients.

RESULTS: In all, 107 MS patients and 18 MS-specialized neurologists completed the

questionnaires. The MS-specialized neurologists in this study reported discussing

most of the suggested treatment goals with their patients. The MS patients

indicated that certain important treatment goals, ie, reduction in disease

progression, reduction or prolongation of time to long-term disability, and

reduction in new magnetic resonance imaging lesions, were not discussed with

them. More than one-quarter of the patients (27%) would appreciate more

information about their treatment. We found evidence for suboptimal patient

adherence to MS therapy (23% indicated taking a treatment break) due to diverse

side effects, lack of efficacy, or practical issues. As compared to these patient

reports, the scale of poor adherence was overestimated by more than half of the

neurologists (on average, 30% estimated treatment breaks).

CONCLUSION: The MS patients and MS-specialized neurologists in this study differ

in their experiences and perspectives on information provision and adherence to

DMDs. Education programs and up-to-date information on MS treatments for both

neurologists and patients may be helpful in improving patient involvement and

patient-physician communication.

DOI: 10.2147/PPA.S106155

PMCID: PMC4854242

PMID: 27175066

134. Cochrane Database Syst Rev. 2016 Apr 19;4:CD009371. doi:

Excluded b/c no primary data (meta-analysis)

10.1002/14651858.CD009371.pub2.

Fingolimod for relapsing-remitting multiple sclerosis.

La Mantia L(1), Tramacere I, Firwana B, Pacchetti I, Palumbo R, Filippini G.

Author information:

(1)Unit of Neurorehabilitation - Multiple Sclerosis Center, I.R.C.C.S. Santa

Maria Nascente - Fondazione Don Gnocchi, Via Capecelatro, 66, Milano, Italy,

20148.

BACKGROUND: Fingolimod was approved in 2010 for the treatment of patients with

the relapsing-remitting (RR) form of multiple sclerosis (MS). It was designed to

reduce the frequency of exacerbations and to delay disability worsening. Issues

on its safety and efficacy, mainly as compared to other disease modifying drugs

(DMDs), have been raised.

OBJECTIVES: To assess the safety and benefit of fingolimod versus placebo, or

other disease-modifying drugs (DMDs), in reducing disease activity in people with

relapsing-remitting multiple sclerosis (RRMS).

SEARCH METHODS: We searched the Cochrane Multiple Sclerosis and Rare Diseases of

the Central Nervous System (CNS) Group's Specialised Trials Register and US Food

and Drug Administration reports (15 February 2016).

SELECTION CRITERIA: Randomised controlled trials (RCTs) assessing the beneficial

and harmful effects of fingolimod versus placebo or other approved DMDs in people

with RRMS.

DATA COLLECTION AND ANALYSIS: We used standard methodological procedures as

expected by Cochrane.

MAIN RESULTS: Six RCTs met our selection criteria. The overall population

included 5152 participants; 1621 controls and 3531 treated with fingolimod at

different doses; 2061 with 0.5 mg, 1376 with 1.25 mg, and 94 with 5.0 mg daily.

Among the controls, 923 participants were treated with placebo and 698 with

others DMDs. The treatment duration was six months in three, 12 months in one,

and 24 months in two trials. One study was at high risk of bias for blinding,

three studies were at high risk of bias for incomplete outcome reporting, and

four studies were at high risk of bias for other reasons (co-authors were

affiliated with the pharmaceutical company). We retrieved 10 ongoing trials; four

of them have been completed.Comparing fingolimod administered at the approved

dose of 0.5 mg to placebo, we found that the drug at 24 months increased the

probability of being relapse-free (risk ratio (RR) 1.44, 95% confidence interval

(CI) (1.28 to 1.63); moderate quality of evidence), but it might lead to little

or no difference in preventing disability progression (RR 1.07, 95% CI 1.02 to

1.11; primary clinical endpoints; low quality evidence). Benefit was observed for

other measures of inflammatory disease activity including clinical (annualised

relapse rate): rate ratio 0.50, 95% CI 0.40 to 0.62; moderate quality evidence;

and magnetic resonance imaging (MRI) activity (gadolinium-enhancing lesions): RR

of being free from (MRI) gadolinium-enhancing lesions: 1.36, 95% CI 1.27 to 1.45;

low quality evidence.The mean change of MRI T2-weighted lesion load favoured

fingolimod at 12 and 24 months.No significant increased risk of discontinuation

due to adverse events was observed for fingolimod 0.5 mg compared to placebo at

six and 24 months. The risk of fingolimod discontinuation was significantly

higher compared to placebo for the dose 1.25 mg at 24 months (RR 1.93, 95% CI

1.48 to 2.52).No significant increased risk of discontinuation due to serious

adverse events was observed for fingolimod 0.5 mg compared to placebo at six and

24 months. A significant increased risk of discontinuation due to serious adverse

events was found for fingolimod 5.0 mg (RR 2.77, 95% CI 1.04 to 7.38) compared to

placebo at six months.Comparing fingolimod 0.5 mg to intramuscular interferon

beta-1a, we found moderate quality evidence that the drug at one year slightly

increased the number of participants free from relapse (RR 1.18, 95% CI 1.09 to

1.27) or from gadolinium-enhancing lesions (RR 1.12, 95% CI 1.05 to 1.19), and

decreased the relapse rate (rate ratio 0.48, 95% CI 0.34 to 0.70). We did not

detect any advantage for preventing disability progression (RR 1.02, 95% CI 0.99

to 1.06; low quality evidence). We did not detect any significant difference for

MRI T2-weighted lesion load change.We found a greater likelihood of participants

discontinuing fingolimod, as compared to other DMDs, due to adverse events in the

short-term (six months) (RR 3.21, 95% CI 1.16 to 8.86), but there was no

significant difference versus interferon beta-1a at 12 months (RR 1.51, 95% CI

0.81 to 2.80; moderate quality evidence). A higher incidence of adverse events

was suggestive of the lower tolerability rate of fingolimod compared to

interferon-beta 1a.Quality of life was improved in participants after switching

from a different DMD to fingolimod at six months, but this effect was not found

compared to placebo at 24 months.All studies were sponsored by Novartis Pharma.

AUTHORS' CONCLUSIONS: Treatment with fingolimod compared to placebo in RRMS

patients is effective in reducing inflammatory disease activity, but it may lead

to little or no difference in preventing disability worsening. The risk of

withdrawals due to adverse events requires careful monitoring of patients over

time. The evidence on the risk/benefit profile of fingolimod compared with

intramuscular interferon beta-1a was uncertain, based on a low number of

head-to-head RCTs with short follow-up duration. The ongoing trial results will

possibly satisfy these issues.

DOI: 10.1002/14651858.CD009371.pub2

PMID: 27091121 [Indexed for MEDLINE]

135. J Behav Med. 2016 Apr;39(2):276-87. doi: 10.1007/s10865-015-9694-6. Epub 2015 Nov

Excluded b/c no real-world adherence/persistence

12.

A pilot study to improve adherence among MS patients who discontinue treatment

against medical advice.

Bruce J(1), Bruce A(2)(3), Lynch S(4), Strober L(5), O'Bryan S(6), Sobotka D(7),

Thelen J(8), Ness A(8), Glusman M(8), Goggin K(9)(10), Bradley-Ewing A(9), Catley

D(8).

Author information:

(1)Department of Psychology, University of Missouri-Kansas City, Kansas City, MO,

USA. Brucejm@umkc.edu.

(2)Department of Pediatrics, University of Kansas Medical Center, Kansas City,

KS, USA.

(3)Children's Mercy Hospital, Kansas City, MO, USA.

(4)Department of Neurology, University of Kansas Medical Center, Kansas City, KS,

USA.

(5)Kessler Foundation, West Orange, NJ, USA.

(6)Texas Tech University, Lubbock, TX, USA.

(7)Saint Luke's Health System, Kansas City, MO, USA.

(8)Department of Psychology, University of Missouri-Kansas City, Kansas City, MO,

USA.

(9)Health Services and Outcome Research, Children's Mercy Hospitals and Clinics,

Kansas City, MO, USA.

(10)Schools of Medicine and Pharmacy, University of Missouri-Kansas City, Kansas

City, MO, USA.

Between 30 and 50% of MS patients may prematurely discontinue disease modifying

therapies. Little research has examined how to best talk with patients who have

discontinued treatment against medical advice. The aim of this pilot study was to

determine whether telephone counseling increases disease modifying therapy (DMT)

re-initiation among nonadherent patients with multiple sclerosis (MS).

Participants were eligible if they had relapsing-remitting disease, had stopped

taking a DMT, and had no plan to re-initiate treatment despite a provider

recommendation. Following a baseline assessment, 81 patients were randomly

assigned to either five 20 min, weekly sessions of Motivational

Interviewing/Cognitive Behavioral Therapy (MI-CBT) or Treatment as Usual (TAU)

with brief education. At 10 weeks, patients initially assigned to TAU switched

over to MI-CBT. Compared to patients in the TAU group, patients undergoing MI-CBT

were significantly more likely to indicate they were re-initiating DMT (41.7 vs.

14.3%). These significant results were replicated among patients crossing over

from TAU to MI-CBT. Treatment satisfaction was high, with 97% of participants

reporting that they would recommend MI-CBT to other patients with MS. Results of

this pilot study provide initial support for the use of MI-CBT among MS patients

who have discontinued treatment against medical advice.Clinicaltrials.gov:

NCT01925690.

DOI: 10.1007/s10865-015-9694-6

PMID: 26563147 [Indexed for MEDLINE]

136. Mult Scler. 2016 Apr;22(4):520-32. doi: 10.1177/1352458515594041. Epub 2015 Jul

Ex-US

21.

The effect of oral immunomodulatory therapy on treatment uptake and persistence

in multiple sclerosis.

Warrender-Sparkes M(1), Spelman T(2), Izquierdo G(3), Trojano M(4), Lugaresi

A(5), Grand'Maison F(6), Havrdova E(7), Horakova D(7), Boz C(8), Oreja-Guevara

C(9), Alroughani R(10), Iuliano G(11), Duquette P(12), Girard M(12), Terzi M(13),

Hupperts R(14), Grammond P(15), Petersen T(16), Fernandez-Bolaños R(17), Fiol

M(18), Pucci E(19), Lechner-Scott J(20), Verheul F(21), Cristiano E(22), Van

Pesch V(23), Petkovska-Boskova T(24), Moore F(25), Kister I(26), Bergamaschi

R(27), Saladino ML(28), Slee M(29), Barnett M(30), Amato MP(31), Shaw C(32),

Shuey N(33), Young C(34), Gray O(35), Kappos L(36), Butzkueven H(37), Kalincik

T(38), Jokubaitis V(39); MSBase study group.

Collaborators: Zwanikken C, Roger E, Despault P, Kister I, Marriott M, Van der

Walt A, King J, Byron J, Morgan L, Hansen E, Skibina O, Haartsen J, Hunter J,

Williams D, Dark L, De Luca G, Di Tommaso V, Travaglini D, Pietrolongo E, Ioia

Md, Farina D, Mancinelli L, Roullet E, Correale J, Ysrraelit C, Caceres F,

Cartechini E, Giuliani G, Rojas JI, Patrucco L, Paine M, Hughes S, Mechati S,

Corageoud M, Bulla A.

Author information:

(1)Department of Medicine, University of Melbourne, Melbourne, Australia.

(2)Department of Neurology, Royal Melbourne Hospital, Melbourne, Australia.

(3)Hospital Universitario Virgen Macarena, Sevilla, Spain.

(4)Department of Basic Medical Sciences, Neuroscience and Sense Organs,

University of Bari, Italy.

(5)MS Center, Department of Neuroscience, Imaging and Clinical Sciences,

University 'G. d'Annunzio', Chieti, Italy.

(6)Neuro Rive-Sud, Hôpital Charles LeMoyne, Quebec, Canada.

(7)Department of Neurology and Center of Clinical Neuroscience, 1st Faculty of

Medicine, General University Hospital and Charles University in Prague, Czech

Republic.

(8)Karadeniz Technical University, Trabzon, Turkey.

(9)University Hospital San Carlos, IdISSC, Madrid, Spain.

(10)Amiri Hospital, Kuwait City, Kuwait.

(11)Ospedali Riuniti di Salerno, Salerno, Italy.

(12)Hôpital Notre-Dame, Montreal, Canada.

(13)Ondokuz Mayis Üniversitesi, Samsun, Turkey.

(14)Orbis Medicle Center, Sittard, The Netherlands.

(15)Hotel-Dieu de Levis, Quebec, Canada.

(16)Aarhus University Hospital, Aarhus C, Denmark.

(17)Hospital Universitario Virgen de Valme, Seville, Spain.

(18)FLENI, Buenos Aires, Argentina.

(19)Ospedale di Macerata, Macerata, Italy.

(20)John Hunter Hospital, Newcastle, Australia.

(21)Groen Hart Ziekenhuis, Gouda, The Netherlands.

(22)Hospital Italiano, Buenos Aires, Argentina.

(23)Cliniques Universitaires Saint-Luc, Brussels, Belgium.

(24)Neurology Clinical Center, Skopje, Macedonia.

(25)Jewish General Hospital, Montreal, Canada.

(26)New York University Langone Medical Center, New York, USA.

(27)National Neurological Institute C. Mondino, Pavia, Italy.

(28)INEBA, Buenos Aires, Argentina.

(29)Flinders University and Medical Centre, Adelaide, Australia.

(30)Brain and Mind Research Institute, Sydney, Australia.

(31)Department NEUROFARBA, Section of Neurosciences, University of Florence,

Florence, Italy.

(32)Geelong Hospital, Geelong, Australia.

(33)St Vincent's Hospital, Melbourne, Australia.

(34)The Walton Centre for Neurology and Neurosurgery, Liverpool, United Kingdom.

(35)Craigavon Area Hospital, Portadown, United Kingdom.

(36)University Hospital Basel, Neurology, Departments of Medicine, Clinical

Research and Biomedicine, Basel, Switzerland.

(37)Department of Medicine, University of Melbourne, Melbourne, Australia/

Department of Neurology, Box Hill Hospital, Monash University, Box Hill,

Australia.

(38)Department of Medicine, University of Melbourne, Melbourne,

Australia/Department of Neurology, Royal Melbourne Hospital, Melbourne,

Australia.

(39)Department of Medicine, University of Melbourne, Melbourne, Australia

vilija.jokubaitis@unimelb.edu.au.

OBJECTIVE: We aimed to analyse the effect of the introduction of fingolimod, the

first oral disease-modifying therapy, on treatment utilisation and persistence in

an international cohort of patients with multiple sclerosis (MS).

METHODS: MSBASIS, a prospective, observational sub-study of the MSBase registry,

collects demographic, clinical and paraclinical data on patients followed from MS

onset (n=4718). We conducted a multivariable conditional risk set survival

analysis to identify predictors of treatment discontinuation, and to assess if

the introduction of fingolimod has altered treatment persistence.

RESULTS: A total of 2640 patients commenced immunomodulatory therapy. Following

the introduction of fingolimod, patients were more likely to discontinue all

other treatments (hazard ratio 1.64, p<0.001) while more patients switched to

fingolimod than any other therapy (42.3% of switches). Patients switched to

fingolimod due to convenience. Patients treated with fingolimod were less likely

to discontinue treatment compared with other therapies (p<0.001). Female sex,

country of residence, younger age, a high Expanded Disability Status Scale score

and relapse activity were all independently associated with higher rates of

treatment discontinuation.

CONCLUSION: Following the availability of fingolimod, patients were more likely

to discontinue injectable treatments. Those who switched to fingolimod were more

likely to do so for convenience. Persistence was improved on fingolimod compared

to other medications.

© The Author(s), 2015.

DOI: 10.1177/1352458515594041

PMID: 26199347 [Indexed for MEDLINE]

137. Patient. 2016 Apr;9(2):171-80. doi: 10.1007/s40271-015-0136-x.

Excluded b/c no real-world adherence/persistence

Patient Preferences for Injectable Treatments for Multiple Sclerosis in the

United States: A Discrete-Choice Experiment.

Poulos C(1), Kinter E(2), Yang JC(3), Bridges JF(4), Posner J(5), Reder AT(6).

Author information:

(1)RTI Health Solutions, 200 Park Offices Drive, Research Triangle Park, PO Box

12194, Durham, NC, 27709, USA. cpoulos@rti.org.

(2)Biogen, Cambridge, MA, USA.

(3)Pacific Economic Research, LLC, Bellevue, WA, USA.

(4)Johns Hopkins University, Baltimore, MD, USA.

(5)RTI Health Solutions, 200 Park Offices Drive, Research Triangle Park, PO Box

12194, Durham, NC, 27709, USA.

(6)University of Chicago Medicine, Chicago, IL, USA.

BACKGROUND AND OBJECTIVE: Patients' perceptions and experiences of medication

efficacy, medication adverse events, dosing frequency, and dosing complexity have

been found to influence adherence to injectable disease-modifying treatments

(DMTs) in patients with multiple sclerosis (MS). The aim of this study was to

quantify patient preferences for features of injectable DMTs for MS.

METHODS: Adult patients in the United States (US) with a self-reported diagnosis

of MS completed an online discrete-choice experiment survey to assess preference

for a number of features of a hypothetical injectable DMT. Patients chose

hypothetical treatments in paired comparisons, where each treatment was described

by features or attributes, including the number of years until disability

progression, the number of relapses in the next 4 years, injection time, the

frequency of injections, the occurrence of flu-like symptoms (FLS), and severity

of injection-site reactions. Random-parameters logit regression parameters were

used to calculate preference weights of attribute levels and the relative

importance of changes in treatment features.

RESULTS: Of the 205 patients who completed the survey, 192 provided sufficient

data for analysis. The results indicated a broad range of tradeoffs that patients

would be willing to make. With regard to this, the relative importance of an

improvement in the number of years until disability progression from 1 to 2

(i.e., vertical distance between preference weights for these attribute levels)

was 0.9 [95% confidence interval (CI) 0.5-1.2], the relative importance of this

change was approximately equivalent to that of an improvement from 12 injections

per month to two (mean 0.8, 95% CI 0.4-1.2), or approximately equivalent to a

decrease from four to one relapses in the next 4 years (mean 0.8, 95% CI

0.5-1.2), or FLS 3 days after every injection to 3 days after some injections

(mean 1.0, 95% CI 0.6-1.4).

CONCLUSIONS: These results suggest that an improvement in treatment efficacy may

be as important as a reduction in injection frequency or a reduction in some

adverse events for patients who self-administer injectable DMTs for MS.

Understanding the preferences of patients who use injectable treatments will

inform the development of such treatments, which may in turn improve patient

medication adherence and well-being.

DOI: 10.1007/s40271-015-0136-x

PMCID: PMC4796329

PMID: 26259849 [Indexed for MEDLINE]

138. Cochrane Database Syst Rev. 2016 Mar 22;3:CD009882. doi:

Excluded b/c no primary data (meta-analysis)

10.1002/14651858.CD009882.pub3.

Teriflunomide for multiple sclerosis.

He D(1), Zhang C, Zhao X, Zhang Y, Dai Q, Li Y, Chu L.

Author information:

(1)Department of Neurology, Affiliated Hospital of Guizhou Medical University,

No. 28, Gui Yi Street, Guiyang, Guizhou Province, China, 550004.

Update of

Cochrane Database Syst Rev. 2012;12:CD009882.

BACKGROUND: This is an update of the Cochrane review "Teriflunomide for multiple

sclerosis" (first published in The Cochrane Library 2012, Issue 12).Multiple

sclerosis (MS) is a chronic immune-mediated disease of the central nervous

system. It is clinically characterized by recurrent relapses or progression, or

both, often leading to severe neurological disability and a serious decline in

quality of life. Disease-modifying therapies (DMTs) for MS aim to prevent

occurrence of relapses and disability progression. Teriflunomide is a pyrimidine

synthesis inhibitor approved by both the US Food and Drug Administration (FDA)

and the European Medicines Agency (EMA) as a DMT for adults with

relapsing-remitting MS (RRMS).

OBJECTIVES: To assess the absolute and comparative effectiveness and safety of

teriflunomide as monotherapy or combination therapy versus placebo or other

disease-modifying drugs (DMDs) (interferon beta (IFNβ), glatiramer acetate,

natalizumab, mitoxantrone, fingolimod, dimethyl fumarate, alemtuzumab) for

modifying the disease course in people with MS.

SEARCH METHODS: We searched the Cochrane Multiple Sclerosis and Rare Diseases of

the CNS Group Specialised Trials Register (30 September 2015). We checked

reference lists of published reviews and retrieved articles and searched reports

(2004 to September 2015) from the MS societies in Europe and America. We also

communicated with investigators participating in trials of teriflunomide and the

pharmaceutical company, Sanofi-Aventis.

SELECTION CRITERIA: We included randomized, controlled, parallel-group clinical

trials with a length of follow-up of one year or greater evaluating

teriflunomide, as monotherapy or combination therapy, versus placebo or other

approved DMDs for people with MS without restrictions regarding dose,

administration frequency and duration of treatment.

DATA COLLECTION AND ANALYSIS: We used the standard methodological procedures of

Cochrane. Two review authors independently assessed trial quality and extracted

data. Disagreements were discussed and resolved by consensus among the review

authors. We contacted the principal investigators of included studies for

additional data or confirmation of data.

MAIN RESULTS: Five studies involving 3231 people evaluated the efficacy and

safety of teriflunomide 7 mg and 14 mg, alone or with add-on IFNβ, versus placebo

or IFNβ-1a for adults with relapsing forms of MS and an entry Expanded Disability

Status Scale score of less than 5.5.Overall, there were obvious clinical

heterogeneities due to diversities in study designs or interventions and

methodological heterogeneities across studies. All studies had a high risk of

detection bias for relapse assessment and a high risk of bias due to conflicts of

interest. Among them, three studies additionally had a high risk of attrition

bias due to a high dropout rate and two studies had an unclear risk of attrition

bias. The studies of combination therapy with IFNβ (650 participants) and the

study with IFNβ-1a as controls (324 participants) also had a high risk for

performance bias and a lack of power due to the limited sample.Two studies

evaluated the benefit and the safety of teriflunomide as monotherapy versus

placebo over a period of one year (1169 participants) or two years (1088

participants). A meta-analysis was not conducted. Compared to placebo,

administration of teriflunomide at a dose of 7 mg/day or 14 mg/day as monotherapy

reduced the number of participants with at least one relapse over one year (risk

ratio (RR) 0.72, 95% confidence interval (CI) 0.59 to 0.87, P value = 0.001 with

7 mg/day and RR 0.60, 95% CI 0.48 to 0.75, P value < 0.00001 with 14 mg/day) or

two years (RR 0.85, 95% CI 0.74 to 0.98, P value = 0.03 with 7 mg/day and RR

0.80, 95% CI 0.69 to 0.93, P value = 0.004 with 14 days). Only teriflunomide at a

dose of 14 mg/day reduced the number of participants with disability progression

over one year (RR 0.55, 95% CI 0.36 to 0.84, P value = 0.006) or two years (RR

0.74, 95% CI 0.56 to 0.96, P value = 0.02). When taking the effect of drop-outs

into consideration, the likely-case scenario analyses still showed a benefit in

reducing the number of participants with at least one relapse, but not for the

number of participants with disability progression. Both doses also reduced the

annualized relapse rate and the number of gadolinium-enhancing T1-weighted

lesions over two years. Quality of evidence for relapse outcomes at one year or

at two years was low, while for disability progression at one year or at two

years was very low.When compared to IFNβ-1a, teriflunomide at a dose of 14 mg/day

had a similar efficacy to IFNβ-1a in reducing the proportion of participants with

at least one relapse over one year, while teriflunomide at a dose of 7 mg/day was

inferior to IFNβ-1a (RR 1.52, 95% CI 0.87 to 2.67, P value = 0.14; 215

participants with 14 mg/day and RR 2.74, 95% CI 1.66 to 4.53, P value < 0.0001;

213 participants with 7 mg/day). However, the quality of evidence was very low.In

terms of safety profile, the most common adverse events associated with

teriflunomide were diarrhoea, nausea, hair thinning, elevated alanine

aminotransferase, neutropenia and lymphopenia. These adverse events had a

dose-related effects and rarely led to treatment discontinuation.

AUTHORS' CONCLUSIONS: There was low-quality evidence to support that

teriflunomide at a dose of 7 mg/day or 14 mg/day as monotherapy reduces both the

number of participants with at least one relapse and the annualized relapse rate

over one year or two years of treatment in comparison with placebo. Only

teriflunomide at a dose of 14 mg/day reduced the number of participants with

disability progression and delayed the progression of disability over one year or

two years, but the quality of the evidence was very low. The quality of available

data was too low to evaluate the benefit teriflunomide as monotherapy versus

IFNβ-1a or as combination therapy with IFNβ. The common adverse effects were

diarrhoea, nausea, hair thinning, elevated alanine aminotransferase, neutropenia

and lymphopenia. These adverse effects were mostly mild-to-moderate in severity,

but had a dose-related effect. New studies of high quality and longer follow-up

are needed to evaluate the comparative benefit of teriflunomide on these outcomes

and the safety in comparison with other DMTs.

DOI: 10.1002/14651858.CD009882.pub3

PMID: 27003123 [Indexed for MEDLINE]

139. Can J Neurol Sci. 2016 Mar;43(2):278-83. doi: 10.1017/cjn.2015.325. Epub 2016 Jan

Ex-US

25.

Canadian Experience with Fingolimod: Adherence to Treatment and Monitoring.

Lapierre Y(1), O'Connor P(2), Devonshire V(3), Freedman MS(4), Kremenchutzky

M(5), Yeung M(6), Schecter R(7).

Author information:

(1)1McGill University Health Centre,Montreal,Quebec.

(2)2St. Michael's Hospital,Toronto,Ontario.

(3)3University of British Columbia Hospital,Vancouver,British Columbia.

(4)4The University of Ottawa and the Ottawa Hospital Research

Institute,Ottawa,Ontario.

(5)5London Health Sciences Centre,London,Ontario.

(6)6Foothills Medical Centre,Calgary,Alberta.

(7)7Novartis Pharmaceuticals Canada Inc.,Dorval,Quebec.

BACKGROUND: The Canadian GILENYA® Go ProgramTM provides education and support to

people with relapsing-remitting multiple sclerosis during fingolimod treatment.

METHODS: Data were collected and analyzed from the time of the first individual

enrolled in March 2011 to March 31, 2014. Individuals were excluded if they

withdrew from the program prior to receiving the first dose, or had not completed

the first dose observation (FDO) at the time of data cut-off. Reports of adverse

effects were validated with a database of adverse events reported to Novartis

Pharmaceuticals Canada Inc.

RESULTS: A total of 2,399 individuals had completed FDO at the end of the

three-year observation period. Mean age was 41.2 years; 75.2% were female. The

most recent prior therapies reported were interferon-β agents (50.2%), glatiramer

acetate (31.1%), natalizumab (14.2%), no prior therapy (3.3%), and other agent

(1.1%). Reasons for switching to fingolimod were lack of efficacy (34.9%), side

effects (34.6%), and dissatisfaction with injections/infusion (30.4%).

Continuation rates with fingolimod at 12, 24 and 30 months were 80.7%, 76.6% and

76.0%, respectively. The discontinuation rate due to reported lack of efficacy

during the three-year period was 1.3%. There was 94.4% adherence to the scheduled

ophthalmic examination.

CONCLUSIONS: The GILENYA® Go ProgramTM captures data for virtually all

fingolimod-treated patients in Canada, enabling the evaluation of fingolimod use

in routine practice. Ongoing patient support and reminders to take the

medication, in conjunction with physicians' and/or patients' perception of the

efficacy and tolerability of fingolimod, resulted in a high rate of continuation

during longer-term therapy.

DOI: 10.1017/cjn.2015.325

PMID: 26890887 [Indexed for MEDLINE]

140. J Neurol. 2016 Mar;263(3):606-10. doi: 10.1007/s00415-015-7999-6. Epub 2016 Feb

Excluded b/c no real-world adherence/persistence

25.

Acute relapse after initiation of Siponimod in a patient with secondary

progressive MS.

Hussain R(1)(2), O'Leary S(1), Pacheco FM(3)(4), Zacharias TE(2), Litvak P(2),

Sguigna P(2), Marder E(1)(2), Kia K(5)(6), Kooner K(7)(8), Stüve O(9)(10)(11).

Author information:

(1)Neurology Section, VA North Texas Health Care System, Medical Service, 4500

South Lancaster Rd., Dallas, TX, 75216, USA.

(2)Department of Neurology and Neurotherapeutics, University of Texas

Southwestern Medical Center at Dallas, Dallas, TX, USA.

(3)Spinal Cord Injury Unit, VA North Texas Health Care System, Medical Service,

Dallas, TX, USA.

(4)Department of Physical Medicine and Rehabilitation, University of Texas

Southwestern Medical Center at Dallas, Dallas, TX, USA.

(5)Dermatology Section, VA North Texas Health Care System, Medical Service,

Dallas, TX, USA.

(6)Department of Dermatology, University of Texas Southwestern Medical Center at

Dallas, Dallas, TX, USA.

(7)Ophthalmology Section, VA North Texas Health Care System, Medical Service,

Dallas, TX, USA.

(8)Department of Ophthalmology, University of Texas Southwestern Medical Center

at Dallas, Dallas, TX, USA.

(9)Neurology Section, VA North Texas Health Care System, Medical Service, 4500

South Lancaster Rd., Dallas, TX, 75216, USA. olaf.stuve@utsouthwestern.edu.

(10)Department of Neurology and Neurotherapeutics, University of Texas

Southwestern Medical Center at Dallas, Dallas, TX, USA.

olaf.stuve@utsouthwestern.edu.

(11)Department of Neurology, Klinikum rechts der Isar, Technische Universität

München, Munich, Germany. olaf.stuve@utsouthwestern.edu.

Sphingosine 1-phosphate (S1P) is a signaling molecule that binds to five G

protein-coupled receptors (Proc Natl Acad Sci USA 108:751-756, 2011). Modulation

of these receptors has been associated with pleiotropic biological effects in the

immune, cardiovascular, and central nervous systems (CNS). The functional S1P

receptor antagonist fingolimod was the first member of this class of

pharmacotherapeutics to be approved for treatment of relapsing multiple sclerosis

(MS). Siponimod is currently in clinical trial in patients with secondary

progressive (SP) MS, a clinical trial for which there is an unmet need for

disease-modifying agents. 10 weeks into the trial, the patient awoke with blurry

vision in his left eye, and was subsequently diagnosed with an acute optic

neuritis. Despite discontinuation of siponimod and treatment with pulse

corticosteroids, the patient did not regain visual function in the affected eye.

This is the first report of disease reactivation shortly after initiating

siponimod in a patient with SPMS. This case illustrates that the known changes in

lymphocyte numbers and composition in the CNS associated with S1P receptor

antagonism during the SPMS disease stage may have adverse outcomes in some

patients during treatment initiation, and that close clinical and paraclinical

monitoring is advised.

DOI: 10.1007/s00415-015-7999-6

PMID: 26914924 [Indexed for MEDLINE]

141. Stem Cells Transl Med. 2016 Mar;5(3):379-91. doi: 10.5966/sctm.2015-0103. Epub

Excluded b/c no real-world adherence/persistence

2016 Jan 7.

Human Cortical Neural Stem Cells Expressing Insulin-Like Growth Factor-I: A Novel

Cellular Therapy for Alzheimer's Disease.

McGinley LM(1), Sims E(1), Lunn JS(1), Kashlan ON(2), Chen KS(2), Bruno ES(1),

Pacut CM(1), Hazel T(3), Johe K(3), Sakowski SA(4), Feldman EL(5).

Author information:

(1)Department of Neurology, University of Michigan, Ann Arbor, Michigan, USA.

(2)Department of Neurosurgery, University of Michigan, Ann Arbor, Michigan, USA.

(3)Neuralstem, Inc., Germantown, Maryland, USA.

(4)A. Alfred Taubman Medical Research Institute, University of Michigan, Ann

Arbor, Michigan, USA.

(5)Department of Neurology, University of Michigan, Ann Arbor, Michigan, USA A.

Alfred Taubman Medical Research Institute, University of Michigan, Ann Arbor,

Michigan, USA efeldman@med.umich.edu.

Alzheimer's disease (AD) is the most prevalent age-related neurodegenerative

disorder and a leading cause of dementia. Current treatment fails to modify

underlying disease pathologies and very little progress has been made to develop

effective drug treatments. Cellular therapies impact disease by multiple

mechanisms, providing increased efficacy compared with traditional single-target

approaches. In amyotrophic lateral sclerosis, we have shown that transplanted

spinal neural stem cells (NSCs) integrate into the spinal cord, form synapses

with the host, improve inflammation, and reduce disease-associated pathologies.

Our current goal is to develop a similar "best in class" cellular therapy for AD.

Here, we characterize a novel human cortex-derived NSC line modified to express

insulin-like growth factor-I (IGF-I), HK532-IGF-I. Because IGF-I promotes

neurogenesis and synaptogenesis in vivo, this enhanced NSC line offers additional

environmental enrichment, enhanced neuroprotection, and a multifaceted approach

to treating complex AD pathologies. We show that autocrine IGF-I production does

not impact the cell secretome or normal cellular functions, including

proliferation, migration, or maintenance of progenitor status. However,

HK532-IGF-I cells preferentially differentiate into gamma-aminobutyric acid-ergic

neurons, a subtype dysregulated in AD; produce increased vascular endothelial

growth factor levels; and display an increased neuroprotective capacity in vitro.

We also demonstrate that HK532-IGF-I cells survive peri-hippocampal

transplantation in a murine AD model and exhibit long-term persistence in

targeted brain areas. In conclusion, we believe that harnessing the benefits of

cellular and IGF-I therapies together will provide the optimal therapeutic

benefit to patients, and our findings support further preclinical development of

HK532-IGF-I cells into a disease-modifying intervention for AD.

©AlphaMed Press.

DOI: 10.5966/sctm.2015-0103

PMCID: PMC4807660

PMID: 26744412 [Indexed for MEDLINE]

142. JMIR Res Protoc. 2016 Feb 4;5(1):e23. doi: 10.2196/resprot.4473.

Excluded b/c no real-world adherence/persistence

Multiple Sclerosis Therapy With Disease-Modifying Treatments in Germany: The

PEARL (ProspEctive phArmacoeconomic cohoRt evaluation) Noninterventional Study

Protocol.

Vormfelde SV(1), Ortler S, Ziemssen T.

Author information:

(1)Novartis Pharma GmbH, Clinical Research in Neuroscience, Nuremberg, Germany.

BACKGROUND: Patients with multiple sclerosis (MS) require long-term therapy and

have a wide variety of needs for health-related support. The efficacy and safety

of MS therapy, as assessed by both clinicians and patients, are important

parameters that need to be considered. However, few studies combine data on

efficacy and safety outcomes with pharmacoeconomic data.

OBJECTIVE: Here, we present the study design of the ProspEctive phArmacoeconomic

cohoRt evaluation (PEARL), a prospective, multicenter, noninterventional cohort

study on patients with relapsing-remitting MS (RRMS) treated with

disease-modifying treatments (DMTs).

METHODS: During a prospective observational phase of 24 months per patient, PEARL

evaluated clinical and patient-perceived efficacy and safety measures, as well as

pharmacoeconomic data on RRMS patients treated with DMTs-interferon beta and

glatiramer acetate. Measurements of the patients' perceptions included the

assessment of patient-reported quality of life, treatment satisfaction, and

compliance. The study was planned to include 1800 outpatients from 180 German

neurological practices who had continuously been treated with an approved DMT for

at least 30 days. The primary statistical analyses of the PEARL study will be

descriptive. Particular focus will be on specific subgroups, such as patients who

switched DMTs during therapy and patients with disease worsening or disease

activity. Subgroups will be compared using stratified analyses.

RESULTS: Data collection for PEARL started in September 2010 and ended in July

2013. As of July 2015, the study is completed and is currently being analyzed and

written up.

CONCLUSIONS: PEARL is evaluating both the health status and resource utilization

of RRMS patients treated with DMTs in Germany. The combination of

pharmacoeconomic data with clinical and patients' self-perceived efficacy and

safety outcomes will add useful information to the currently incomplete picture

of the overall RRMS burden in Germany.

DOI: 10.2196/resprot.4473

PMCID: PMC4759453

PMID: 26846334

Excluded b/c no real-world adherence/persistence

143. Clin Kidney J. 2016 Feb;9(1):82-9. doi: 10.1093/ckj/sfv114. Epub 2015 Nov 10.

Drug-induced Fanconi syndrome associated with fumaric acid esters treatment for

psoriasis: a case series.

Balak DM(1), Bouwes Bavinck JN(2), de Vries AP(3), Hartman J(4), Neumann HA(1),

Zietse R(5), Thio HB(1).

Author information:

(1)Department of Dermatology , Erasmus Medical Center , Rotterdam , The

Netherlands.

(2)Department of Dermatology , Leiden University Medical Center , Leiden , The

Netherlands.

(3)Division of Nephrology, Department of Medicine , Leiden University Medical

Center , Leiden , The Netherlands.

(4)Netherlands Pharmacovigilance Centre Lareb , 's-Hertogenbosch , The

Netherlands.

(5)Division of Nephrology and Transplantation, Department of Internal Medicine ,

Erasmus Medical Center , Rotterdam , The Netherlands.

BACKGROUND: Fumaric acid esters (FAEs), an oral immunomodulating treatment for

psoriasis and multiple sclerosis, have been anecdotally associated with proximal

renal tubular dysfunction due to a drug-induced Fanconi syndrome. Few data are

available on clinical outcomes of FAE-induced Fanconi syndrome.

METHODS: Descriptive case series with two cases of Fanconi syndrome associated

with FAE treatment diagnosed at two Dutch university nephrology departments,

three cases reported at the Dutch and German national pharmacovigilance databases

and six previously reported cases.

RESULTS: All 11 cases involved female patients with psoriasis. The median age at

the time of onset was 38 years [interquartile range (IQR) 37-46]. Patients

received long-term FAEs treatment with a median treatment duration of 60 months

(IQR 28-111). Laboratory tests were typically significant for low serum levels of

phosphate and uric acid, while urinalysis showed glycosuria and proteinuria.

Eight (73%) patients had developed a hypophosphataemic osteomalacia and three

(27%) had pathological bone fractures. All patients discontinued FAEs, while four

(36%) patients were treated with supplementation of phosphate and/or vitamin D.

Five (45%) patients had persisting symptoms despite FAEs discontinuation.

CONCLUSIONS: FAEs treatment can cause drug-induced Fanconi syndrome, but the

association has been reported infrequently. Female patients with psoriasis

treated long term with FAEs seem to be particularly at risk. Physicians treating

patients with FAEs should be vigilant and monitor for the potential occurrence of

Fanconi syndrome. Measurement of the urinary albumin:total protein ratio is a

suggested screening tool for tubular proteinuria in Fanconi syndrome.

DOI: 10.1093/ckj/sfv114

PMCID: PMC4720194

PMID: 26798466

144. J Neurol. 2016 Feb;263(2):327-333. doi: 10.1007/s00415-015-7970-6. Epub 2015 Dec

Ex-US

8.

Efficacy of fingolimod is superior to injectable disease modifying therapies in

second-line therapy of relapsing remitting multiple sclerosis.

Braune S(1), Lang M(2), Bergmann A(2); NeuroTransData Study Group.

Author information:

(1)Neurozentrum Prien, Bernauer Str. 12, 83209, Prien, Germany.

braune@neurozentrum-prien.de.

(2)Neurozentrum Prien, Bernauer Str. 12, 83209, Prien, Germany.

Although fingolimod is registered in Europe for treatment of relapsing-remitting

multiple sclerosis (RRMS) if earlier disease modifying therapy (DMT) has failed,

no data regarding its efficacy in this patient group are available. This

observational cohort study of the NeuroTransData network includes German RRMS

outpatients with failure of earlier therapy with injectable DMT (iDMT), therefore

switching to either another iDMT (n = 133) or to fingolimod (n = 300).

Statistical comparison of clinical baseline characteristics showed more severely

affected patients in the fingolimod group. A propensity-score matched group

comparison was performed (n = 99 in each group) covering more than 2-year

observation time. Fingolimod showed statistically significant superior efficacy

in comparison to iDMT regarding annualized relapse rate (0.21 versus 0.33 per

year), time-to-relapse and likelihood of relapse (iDMT hazard ratio 1.7),

proportion and likelihood of patients with EDSS progression (15.10 versus 31.00%;

iDMT hazard ratio 1.7), persistence on medication and likelihood of

discontinuation (iDMT hazard ratio 3.0). Significantly more patients were free of

relapse and EDSS progression with fingolimod than with their second iDMT (64.4

versus 46.5%, p < 0.03). This real-life evidence in German RRMS outpatients

support data from controlled clinical studies and can quantitatively support

clinical decision finding processes if iDMT therapy fails in RRMS.

DOI: 10.1007/s00415-015-7970-6

PMCID: PMC4751192

PMID: 26645389 [Indexed for MEDLINE]

Excluded b/c no primary data (NMA)

145. JAMA. 2016 Jan 26;315(4):409-10. doi: 10.1001/jama.2015.18984.

Association of Immunotherapies With Outcomes in Relapsing-Remitting Multiple

Sclerosis.

Tramacere I(1), Del Giovane C(2), Filippini G(3).

Author information:

(1)Neuroepidemiology Unit, Fondazione IRCCS Istituto Neurologico Carlo Besta,

Milan, Italy.

(2)Italian Cochrane Centre, Department of Diagnostic, Clinical and Public Health

Medicine, University of Modena and Reggio Emilia, Modena, Italy.

(3)Scientific Direction, Fondazione IRCCS Istituto Neurologico Carlo Besta,

Milan, Italy.

CLINICAL QUESTION: What immunotherapies for multiple sclerosis are associated

with the greatest benefit and highest risk of discontinuation due to adverse

events in patients with relapsing-remitting multiple sclerosis?

BOTTOM LINE: Alemtuzumab, natalizumab, and fingolimod were associated with the

greatest benefit with regard to relapse prevention. Their association with

prevention of disability worsening was unclear. Fingolimod was associated with a

high risk of treatment discontinuation due to adverse events.

DOI: 10.1001/jama.2015.18984

PMID: 26813214 [Indexed for MEDLINE]

Excluded b/c no real-world adherence/persistence

146. Case Rep Neurol Med. 2016;2016:5876798. Epub 2016 Nov 22.

Progressive Multifocal Leukoencephalopathy in a Multiple Sclerosis Patient

Diagnosed after Switching from Natalizumab to Fingolimod.

Sinnecker T(1), Othman J(2), Kühl M(2), Metz I(3), Niendorf T(4), Kunkel A(2),

Paul F(5), Wuerfel J(6), Faiss J(2).

Author information:

(1)Department of Neurology, Asklepios Fachklinikum Teupitz, Teupitz, Germany;

NeuroCure Clinical Research Center, Charité-Universitätsmedizin Berlin, Berlin,

Germany; Department of Neurology, Universitätsspital Basel, Basel, Switzerland.

(2)Department of Neurology, Asklepios Fachklinikum Teupitz, Teupitz, Germany.

(3)Department of Neuropathology, Universitätsmedizin Göttingen, Göttingen,

Germany.

(4)Berlin Ultrahigh Field Facility, Max Delbrück Center for Molecular Medicine,

Berlin, Germany; Experimental and Clinical Research Center,

Charité-Universitätsmedizin Berlin and Max Delbrück Center for Molecular

Medicine, Berlin, Germany.

(5)NeuroCure Clinical Research Center, Charité-Universitätsmedizin Berlin,

Berlin, Germany; Experimental and Clinical Research Center,

Charité-Universitätsmedizin Berlin and Max Delbrück Center for Molecular

Medicine, Berlin, Germany; Clinical and Experimental Multiple Sclerosis Research

Center, Charité-Universitätsmedizin Berlin, Berlin, Germany; Department of

Neurology, Charité-Universitätsmedizin Berlin, Berlin, Germany.

(6)NeuroCure Clinical Research Center, Charité-Universitätsmedizin Berlin,

Berlin, Germany; Medical Imaging Analysis Center AG, Basel, Switzerland.

Background. Natalizumab- (NTZ-) associated progressive multifocal

leukoencephalopathy (PML) is a severe and often disabling infectious central

nervous system disease that can become evident in multiple sclerosis (MS)

patients after NTZ discontinuation. Recently, novel diagnostic biomarkers for the

assessment of PML risk in NTZ treated MS patients such as the anti-JC virus

antibody index have been reported, and the clinical relevance of milky-way

lesions detectable by MRI has been discussed. Case Presentation and Conclusion.

We report a MS patient in whom PML was highly suspected solely based on MRI

findings after switching from NTZ to fingolimod despite repeatedly negative

(ultrasensitive) polymerase chain reaction (PCR) testing for JC virus DNA in

cerebrospinal fluid. The PML diagnosis was histopathologically confirmed by brain

biopsy. The occurrence of an immune reconstitution inflammatory syndrome (IRIS)

during fingolimod therapy, elevated measures of JCV antibody indices, and the

relevance of milky-way-like lesions detectable by (7 T) MRI are discussed.

DOI: 10.1155/2016/5876798

PMCID: PMC5138460

PMID: 27994897

Conflict of interest statement: The authors declare that they have no competing

interests.

147. Eur J Neurol. 2016 Jan;23 Suppl 1:18-27. doi: 10.1111/ene.12883.

Excluded b/c no primary data (narrative review)

Disease-modifying treatments for multiple sclerosis - a review of approved

medications.

Torkildsen Ø(1)(2), Myhr KM(1)(3), Bø L(1)(2).

Author information:

(1)Department of Clinical Medicine, KG Jebsen MS Research Centre, University of

Bergen, Bergen, Norway.

(2)Department of Neurology, Norwegian Multiple Sclerosis Competence Centre,

Haukeland University Hospital, Bergen, Norway.

(3)Department of Neurology, Norwegian Multiple Sclerosis Registry and Biobank,

Haukeland University Hospital, Bergen, Norway.

BACKGROUND AND PURPOSE: There is still no curative treatment for multiple

sclerosis (MS), but during the last 20 years eight different disease-modifying

compounds have been approved for relapsing-remitting MS (RRMS).

METHODS: A literature search was conducted on published randomized controlled

phase III trials indexed in PubMed on the approved medications until 21 May 2015.

RESULTS: In this review the mode of action, documented treatment effects and side

effects of the approved MS therapies are briefly discussed.

CONCLUSIONS: Based on current knowledge of risk-benefit of the approved MS

medications, including factors influencing adherence, it is suggested that oral

treatment with dimethyl fumarate or teriflunomide should be preferred as a

starting therapy amongst the first-line preparations for de novo RRMS. In the

case of breakthrough disease on first-line therapy, or rapidly evolving severe

RRMS, second-line therapy with natalizumab, fingolimod or alemtuzumab should be

chosen based on careful risk-benefit stratification.

© 2015 The Authors. European Journal of Neurology published by John Wiley & Sons

Ltd on behalf of European Academy of Neurology.

DOI: 10.1111/ene.12883

PMCID: PMC4670697

PMID: 26563094 [Indexed for MEDLINE]

Excluded b/c no oral DMD results

148. J Med Econ. 2016;19(5):497-505. doi: 10.3111/13696998.2015.1134546. Epub 2016 Jan

18.

The impact of persistence with therapy on inpatient admissions and emergency room

visits in the US among patients with multiple sclerosis.

Thomas NP(1), Curkendall S(2), Farr AM(2), Yu E(1), Hurley D(1).

Author information:

(1)a a Genentech, Inc., Health Economics , South San Francisco , CA , USA.

(2)b b Truven Health Analytics, Health Economics , Ann Arbor , MI , USA.

OBJECTIVE: Disease-modifying therapy (DMT) for multiple sclerosis (MS) can reduce

relapses and delay progression; however, poor adherence and persistence with DMT

can result in sub-optimal outcomes. The associations between DMT adherence and

persistence and inpatient admissions and emergency room (ER) visits were

investigated.

METHODS: Patients with MS who initiated a DMT in a US administrative claims

database were followed for 1 year. Persistence to initiated DMT was measured as

the time from DMT initiation to discontinuation (a gap of >60 days without drug

'on hand') or end of 1-year follow-up. Adherence to initiated DMT was measured

during the persistent period and was operationalized as the medication possession

ratio (MPR). Patients with an MPR <0.80 were considered non-adherent. Claims

during the 1-year follow-up period were evaluated for the presence of an

all-cause inpatient admission or an ER visit. Adjusted odds ratios (AORs) for

inpatient admission or ER visit comparing persistent vs non-persistent and

adherent vs non-adherent patients were estimated using logistic regression models

adjusted for patient characteristics.

RESULTS: The final sample included 16,218 patients. During the 1-year follow-up

period, 35.3% of patients discontinued their initiated DMT and 13.9% were not

adherent while on therapy. During that same period, 10.0% of patients had an

inpatient admission and 24.9% had an ER visit. The likelihoods of inpatient

admission and ER visit were significantly decreased in persistent patients (AOR

[95% CI] = 0.50 [0.45, 0.56] and 0.65 [0.60, 0.69], respectively) and in adherent

patients (AOR [95% CI] = 0.83 [0.71, 0.97] and 0.86 [0.77, 0.95], respectively).

CONCLUSIONS: Persistence and adherence with initiated DMT are associated with

decreased likelihoods of inpatient admission or ER visit, which may translate to

improved clinical outcomes.

DOI: 10.3111/13696998.2015.1134546

PMID: 26706292 [Indexed for MEDLINE]

Ex-US

149. Mult Scler. 2016 Jan;22(1):85-93. doi: 10.1177/1352458515579216. Epub 2015 Apr

28.

Comparative analysis of first-year fingolimod and natalizumab drug

discontinuation among Swedish patients with multiple sclerosis.

Frisell T(1), Forsberg L(2), Nordin N(2), Kiesel C(2), Alfredsson L(3), Askling

J(4), Hillert J(2), Olsson T(2), Piehl F(2).

Author information:

(1)Clinical Epidemiology Unit, Department of Medicine, Karolinska Institutet,

Solna, Sweden thomas.frisell@ki.se.

(2)Department of Clinical Neuroscience, Karolinska Institutet, Solna, Sweden.

(3)Institute of Environmental Medicine, Karolinska Institutet, Solna, Sweden.

(4)Clinical Epidemiology Unit, Department of Medicine, Karolinska Institutet,

Solna, Sweden/Rheumatology Unit, Department of Medicine, Karolinska Institutet,

Solna, Sweden.

BACKGROUND: Natalizumab (NTZ) and fingolimod (FGL) are mainly used second line in

relapsing-remitting multiple sclerosis (MS), although pivotal trials included

mainly treatment-naïve patients.

OBJECTIVE: This study aims to provide real-world data on safety and

discontinuation rates.

METHODS: Using IMSE, a drug monitoring registry for all newer MS drugs in Sweden,

we analysed differences in baseline characteristics and 1-year drug survival for

patients registered 2011-2013, initiating treatment with NTZ (n=640) or FGL

(n=876). Among FGL initiators, n=383 (44%) had previously used NTZ

(FGL(afterNTZ)).

RESULTS: Compared with NTZ, the FGL cohort was older and more often male (36/38

years, 24%/33% males). Baseline Expanded Disability Status Scale was similar

across groups, but MS Severity Score was higher in NTZ patients, and Symbol Digit

Modalities Test and MS Impact Scale (MSIS-29) was higher in FGL(afterNTZ) versus

FGL(NTZ-naïve) patients. Proportion on drug after 1 year was high, NTZ=87%,

FGL(NTZ-naïve)=83% and FGL(afterNTZ)=76%. Adverse events was the most frequent

reason for discontinuing FGL (FGL(NTZ-naïve)=9%, FGL(afterNTZ)=12%), and was

significantly higher than on NTZ (3%). In contrast, the proportion of patients

stopping treatment due to lack of effect was more similar: NTZ=4%,

FGL(NTZ-naïve)=3%, FGL(afterNTZ)=8%.

CONCLUSION: FGL and NTZ were both well tolerated, but FGL less so than NTZ,

especially in patients switching to FGL from NTZ. Group differences were not

explained by differences in recorded baseline characteristics.

© The Author(s), 2015.

DOI: 10.1177/1352458515579216

PMID: 25921036 [Indexed for MEDLINE]

150. Mult Scler J Exp Transl Clin. 2016 Jan-Dec;2. doi: 10.1177/2055217316677868. Epub

2016 Nov 1.

Efficacy and tolerability of oral versus injectable disease-modifying therapies

for multiple sclerosis in clinical practice.

Longbrake EE(1), Cross AH(2), Salter A(3).

Author information:

(1)Department of Neurology, Yale University, USA; Department of Neurology,

Washington University in St. Louis, USA.

(2)Department of Neurology, Washington University in St. Louis, USA.

(3)Division of Biostatistics, Washington University in St. Louis, USA.

BACKGROUND: The advent of oral disease-modifying therapies fundamentally changed

the treatment of multiple sclerosis. Nevertheless, impressions of their relative

efficacy and tolerability are primarily founded on expert opinion.

OBJECTIVE: The purpose of this study was to determine whether oral

disease-modifying therapies were better tolerated and/or more effective for

controlling multiple sclerosis compared to injectable therapies in clinical

practice.

METHODS: Single-center, retrospective cohort study. 480 patients initiated oral

(fingolimod, teriflunomide, or dimethyl fumarate) or injectable therapy between

March 2013-March 2015 and follow-up data was collected for 5-31 months. Outcomes

included on-drug multiple sclerosis activity and drug discontinuation. Cox

proportional hazards models were used to control for baseline differences and

sensitivity analyses using propensity-weighted matching were performed.

RESULTS: A higher proportion of teriflunomide-treated patients experienced

multiple sclerosis activity compared to those treated with injectable therapies

(p = 0.0053) in the adjusted model. Breakthrough multiple sclerosis was equally

prevalent among fingolimod and dimethyl fumarate-treated compared to injectable

therapy-treated patients. Of patients initiating a disease-modifying therapy,

32-46% discontinued or switched treatments during the study. After controlling

for baseline differences, discontinuation rates were comparable across treatment

groups.

CONCLUSIONS: In this cohort, oral and injectable disease-modifying therapies were

equally well tolerated, but teriflunomide appeared less effective for controlling

multiple sclerosis activity than injectable therapies. Further study is needed.

PMCID: PMC5340186

PMID: 28280599

Excluded b/c no real-world adherence/persistence

151. Mult Scler Relat Disord. 2016 Jan;5:97-104. doi: 10.1016/j.msard.2015.11.006.

Epub 2015 Nov 10.

Pooled safety and tolerability data from four placebo-controlled teriflunomide

studies and extensions.

Comi G(1), Freedman MS(2), Kappos L(3), Olsson TP(4), Miller AE(5), Wolinsky

JS(6), O'Connor PW(7), Benamor M(8), Dukovic D(9), Truffinet P(10), Leist TP(11).

Author information:

(1)University Vita-Salute San Raffaele, Via Olgettina, 60, 20132 Milan, Italy.

Electronic address: g.comi@hsr.it.

(2)University of Ottawa and the Ottawa Hospital Research Institute, The Ottawa

Hospital - General Campus, 501 Smyth Road, Ottawa, ON, Canada K1H 8L6. Electronic

address: mfreedman@toh.on.ca.

(3)Neurology, Departments of Medicine and Clinical Research, University Hospital

Basel, Petersgraben 4, CH-4031 Basel, Switzerland. Electronic address:

Ludwig.Kappos@usb.ch.

(4)Department of Clinical Neurosciences, Karolinska Hospital, 17176 Stockholm,

Sweden. Electronic address: Tomas.Olsson@ki.se.

(5)Icahn School of Medicine at Mount Sinai-The Corinne Goldsmith Dickinson Center

for Multiple Sclerosis, 5 East 98th Street, New York, NY 10029, USA. Electronic

address: aaron.miller@mssm.edu.

(6)University of Texas Health Science Center at Houston, 6431 Fannin Street,

Houston, TX 77030, USA. Electronic address: Jerry.S.Wolinsky@uth.tmc.edu.

(7)University of Toronto, St Michael's Hospital, 30 Bond Street, Toronto, ON,

Canada M5B 1W8. Electronic address: opaul362@gmail.com.

(8)Genzyme, a Sanofi company, 1 Avenue Pierre Brossolette, 91385 Chilly-Mazarin,

France. Electronic address: Myriam.Benamor@sanofi.com.

(9)Sanofi, 55 Corporate Drive, Bridgewater, NJ 08807, USA. Electronic address:

Deborah.Dukovic@sanofi.com.

(10)Genzyme, a Sanofi company, 1 Avenue Pierre Brossolette, 91385 Chilly-Mazarin,

France. Electronic address: Philippe.Truffinet@sanofi.com.

(11)Comprehensive Multiple Sclerosis Center, Thomas Jefferson University

Hospital, 900 Walnut Street, Ste. 200, Philadelphia, PA 19107, USA. Electronic

address: Thomas.Leist@jefferson.edu.

BACKGROUND: Teriflunomide, a once-daily oral immunomodulator for the treatment of

relapsing-remitting multiple sclerosis, has demonstrated consistent efficacy on

clinical and MRI parameters in clinical trials.

OBJECTIVE: To summarize the safety and tolerability profile of teriflunomide

based on data from four placebo-controlled trials.

METHODS: Safety and tolerability were assessed using two teriflunomide clinical

program data pools. Pool 1 contained 3044 patients randomized to teriflunomide

(14 mg or 7 mg) or placebo in the core studies of one phase 2 trial and three

phase 3 trials, with cumulative treatment exposure >1500 patient-years per group.

Pool 2 comprised 2338 patients who received teriflunomide treatment in the above

trials, including those continuing in extension studies, with a duration of

treatment up to 12 years, representing >6800 patient-years. Safety assessments

included adverse events, laboratory parameters, and physical examinations.

RESULTS: In Pool 1, the number of patients experiencing adverse events and

serious adverse events was similar in the three treatment groups. Common events

occurring in ≥ 10% of patients in either teriflunomide group, and with an

incidence ≥ 2% compared with placebo, were alanine aminotransferase (ALT)

increase, headache, diarrhea, hair thinning, and nausea. Overall, the nature of

events observed in Pool 2 was similar to Pool 1. The majority of events in both

pools were of mild-to-moderate intensity, were self-limiting, and infrequently

resulted in discontinuation of therapy. The most common reason for treatment

discontinuation in all treatment groups was ALT elevation, reflecting the

protocol requirement to discontinue treatment on confirmation of ALT > 3 × the

upper limit of normal.

CONCLUSIONS: No new or unexpected safety signals beyond those detected in

individual trials were identified in this pooled analysis with treatment duration

exceeding 12 years and a cumulative exposure to teriflunomide exceeding 6800

patient-years. Overall, both doses of teriflunomide had consistent and manageable

safety profiles.

Copyright © 2015 The Authors. Published by Elsevier B.V. All rights reserved.

DOI: 10.1016/j.msard.2015.11.006

PMID: 26856952 [Indexed for MEDLINE]

152. Ther Adv Chronic Dis. 2016 Jan;7(1):18-33. doi: 10.1177/2040622315617354.

Excluded b/c no primary data (narrative review)

Ponesimod, a selective S1P1 receptor modulator: a potential treatment for

multiple sclerosis and other immune-mediated diseases.

D'Ambrosio D(1), Freedman MS(2), Prinz J(3).

Author information:

(1)Actelion Pharmaceuticals - Global Clinical Science and Epidemiology,

Gewerbestrasse 16, Basel 4056, Switzerland.

(2)Multiple Sclerosis Research Clinic, University of Ottawa, Ottawa, Canada.

(3)Dermatology, University of Munich, Munich, Germany.

The first oral treatment for relapsing multiple sclerosis, the nonselective

sphingosine-1-phosphate receptor (S1PR) modulator fingolimod, led to

identification of a pivotal role of sphingosine-1-phosphate and one of its five

known receptors, S1P1R, in regulation of lymphocyte trafficking in multiple

sclerosis. Modulation of S1P3R, initially thought to cause some of fingolimod's

side effects, prompted the search for novel compounds with high selectivity for

S1P1R. Ponesimod is an orally active, selective S1P1R modulator that causes

dose-dependent sequestration of lymphocytes in lymphoid organs. In contrast to

the long half-life/slow elimination of fingolimod, ponesimod is eliminated within

1 week of discontinuation and its pharmacological effects are rapidly reversible.

Clinical data in multiple sclerosis have shown a dose-dependent therapeutic

effect of ponesimod and defined 20 mg as a daily dose with desired efficacy, and

acceptable safety and tolerability. Phase II clinical data have also shown

therapeutic efficacy of ponesimod in psoriasis. These findings have increased our

understanding of psoriasis pathogenesis and suggest clinical utility of S1P1R

modulation for treatment of various immune-mediated disorders. A gradual dose

titration regimen was found to minimize the cardiac effects associated with

initiation of ponesimod treatment. Selectivity for S1P1R, rapid onset and

reversibility of pharmacological effects, and an optimized titration regimen

differentiate ponesimod from fingolimod, and may lead to better safety and

tolerability. Ponesimod is currently in phase III clinical development to assess

efficacy and safety in relapsing multiple sclerosis. A phase II study is also

ongoing to investigate the potential utility of ponesimod in chronic graft versus

host disease.

DOI: 10.1177/2040622315617354

PMCID: PMC4707431

PMID: 26770667

153. BMC Neurol. 2015 Dec 7;15:252. doi: 10.1186/s12883-015-0512-0.

Excluded b/c no real-world adherence/persistence

Clinical and radiologic rebound after discontinuation of natalizumab therapy in a

highly active multiple sclerosis patient was not halted by dimethyl-fumarate: a

case report.

Patti F(1), Leone C(2), Zappia M(3).

Author information:

(1)Department of Medical Sciences, Surgicals and Advanced Technologies G.F.

Ingrassia, Section of Neuroscience, University of Catania, Via Santa Sofia 78,

95123, Catania, Italy. patti@unict.it.

(2)Department of Medical Sciences, Surgicals and Advanced Technologies G.F.

Ingrassia, Section of Neuroscience, University of Catania, Via Santa Sofia 78,

95123, Catania, Italy. carmelaleone84@yahoo.it.

(3)Department of Medical Sciences, Surgicals and Advanced Technologies G.F.

Ingrassia, Section of Neuroscience, University of Catania, Via Santa Sofia 78,

95123, Catania, Italy. m.zappia@unict.it.

BACKGROUND: The evidence on the use of the oral dimethyl-fumarate after the

discontinuation of treatment with natalizumab in people with Multiple Sclerosis

is still little. Natalizumab discontinuation may induce the recurrence or rebound

of the clinical and neuroradiological disease activity. Currently no therapeutic

approach has been established to abolish disease reactivation and rebound after

natalizumab interruption.

CASE PRESENTATION: We describe a case of a 21-year-old woman affected from a

highly active relapsing-remitting Multiple Sclerosis who developed a clinical and

radiological rebound 5 months after the last infusion of natalizumab, while she

was being treated with dimethyl-fumarate 240 mg twice daily. She had received a

bridge "therapy" with Cyclophosphamide before staring dimethyl-fumarate.

CONCLUSION: We report on this case to stimulate further research to establish

whether new current and future drugs available for multiple sclerosis are able to

halt the disease rebound after the natalizumab interruption.

DOI: 10.1186/s12883-015-0512-0

PMCID: PMC4672501

PMID: 26643473 [Indexed for MEDLINE]

154. Immunol Res. 2015 Dec;63(1-3):58-69. doi: 10.1007/s12026-015-8719-0.

Excluded b/c no primary data (narrative review)

Advancing drug delivery systems for the treatment of multiple sclerosis.

Tabansky I(1), Messina MD(2)(3), Bangeranye C(3), Goldstein J(2)(3),

Blitz-Shabbir KM(2), Machado S(2)(3), Jeganathan V(4), Wright P(2), Najjar S(2),

Cao Y(4), Sands W(5)(6), Keskin DB(7), Stern JN(8)(9)(10)(11).

Author information:

(1)Department of Neurobiology and Behavior, The Rockefeller University, New York,

NY, USA.

(2)Department of Neurology, Hofstra North Shore-LIJ School of Medicine,

Hempstead, NY, USA.

(3)Department of Science Education, Hofstra North Shore-LIJ School of Medicine,

Hempstead, NY, USA.

(4)Department of Autoimmunity, The Feinstein Institute for Medical Research,

North Shore-LIJ Health System, Manhasset, NY, USA.

(5)School of Engineering and Applied Sciences, Harvard University, Cambridge, MA,

USA.

(6)Department of Medicine, University of Pittsburgh Medical Center, Pittsburgh,

PA, USA.

(7)Department of Cancer Immunology and AIDS, Dana Farber-Harvard Cancer

Institute, Boston, MA, USA.

(8)Department of Neurobiology and Behavior, The Rockefeller University, New York,

NY, USA. jstern01@rockefeller.edu.

(9)Department of Neurology, Hofstra North Shore-LIJ School of Medicine,

Hempstead, NY, USA. jstern01@rockefeller.edu.

(10)Department of Science Education, Hofstra North Shore-LIJ School of Medicine,

Hempstead, NY, USA. jstern01@rockefeller.edu.

(11)Department of Autoimmunity, The Feinstein Institute for Medical Research,

North Shore-LIJ Health System, Manhasset, NY, USA. jstern01@rockefeller.edu.

Erratum in

Immunol Res. 2016 Apr;64(2):640.

Multiple sclerosis (MS) is a chronic inflammatory autoimmune disease of the

central nervous system. It is characterized by demyelination of neurons and loss

of neuronal axons and oligodendrocytes. In MS, auto-reactive T cells and B cells

cross the blood-brain barrier (BBB), causing perivenous demyelinating lesions

that form multiple discrete inflammatory demyelinated plaques located primarily

in the white matter. In chronic MS, cortical demyelination and progressive axonal

transections develop. Treatment for MS can be stratified into disease-modifying

therapies (DMTs) and symptomatic therapy. DMTs aim to decrease circulating immune

cells or to prevent these cells from crossing the BBB and reduce the inflammatory

response. There are currently 10 DMTs approved for the relapsing forms of MS;

these vary with regard to their efficacy, route and frequency of administration,

adverse effects, and toxicity profile. Better drug delivery systems are being

developed in order to decrease adverse effects, increase drug efficacy, and

increase patient compliance through the direct targeting of pathologic cells.

Here, we address the uses and benefits of advanced drug delivery systems,

including nanoparticles, microparticles, fusion antibodies, and liposomal

formulations. By altering the properties of therapeutic particles and enhancing

targeting, breakthrough drug delivery technologies potentially applicable to

multiple disease treatments may rapidly emerge.

DOI: 10.1007/s12026-015-8719-0

PMID: 26475738 [Indexed for MEDLINE]

155. J Neurol. 2015 Dec;262(12):2627-34. doi: 10.1007/s00415-015-7834-0. Epub 2015 Sep

Excluded b/c no real-world adherence/persistence

4.

Long-term results from a phase 2 extension study of fingolimod at high and

approved dose in relapsing multiple sclerosis.

Montalban X(1), Comi G(2), Antel J(3), O'Connor P(4), de Vera A(5), Cremer M(5),

Sfikas N(5), von Rosenstiel P(5), Kappos L(6).

Author information:

(1)Neurology-Neuroimmunology Department & Neurorehabilitation Unit, Edif. Cemcat,

Vall d'Hebron University Hospital, 119-129, 08035, Barcelona, Spain.

xavier.montalban@cem-cat.org.

(2)Department of Neurology, Vita-Salute San Raffaele, University, Milan, Italy.

(3)Department of Neurology and Neurosurgery, Montreal Neurological Institute

University, 3801, Room 111, Montreal, Canada.

(4)St. Michael's Hospital, 30 Bond St, Toronto, ON, M5B 1W8, Canada.

(5)Novartis Pharma AG, Basel, Switzerland.

(6)Departments of Medicine, Clinical Research, Biomedicine and Biomedical

Engineering, University Hospital, Basel, Switzerland.

Fingolimod safety and efficacy data in relapsing-remitting multiple sclerosis

(RRMS) are available up to 5 years, from an extension of a randomized,

placebo-controlled, double-blind, phase 2 study, at a dose higher (5.0/1.25 mg)

than the approved dose of 0.5 mg. The objective of the study is to present the

end-of-study data (>7 years) from the open-label extension of the phase 2 study.

In the core phase (6 months), patients (N = 281) were randomized to placebo or

fingolimod 1.25/5 mg. In the extension, placebo patients were randomized to

fingolimod 1.25/5 mg. All patients received open-label 1.25 mg fingolimod after

month 24 and 0.5 mg after month 60. Clinical visits were performed every 3

months, expanded disability status scale (EDSS) every 6 months and magnetic

resonance imaging (MRI) annually. 122 (48.8%) patients completed the extension

study; overall fingolimod exposure was 1230.7 patient-years. The most common

(>10%) reasons for study discontinuation were adverse events (19.6%) and consent

withdrawal (16.4%). Fingolimod treatment for >7 years was associated with

sustained low clinical and MRI disease activity. Over 60% of patients remained

relapse free and about 80% were free from any MRI activity. Overall annualized

relapse rate was 0.18. Long-term fingolimod treatment was not associated with new

safety concerns. Long-term fingolimod was well tolerated and associated with a

sustained low level of disease activity.

DOI: 10.1007/s00415-015-7834-0

PMID: 26338810 [Indexed for MEDLINE]

Excluded b/c no oral DMD results

156. Neurol Ther. 2015 Dec;4(2):147-57. doi: 10.1007/s40120-015-0038-9. Epub 2015 Dec

8.

Natalizumab Discontinuation and Treatment Strategies in Patients with Multiple

Sclerosis (MS): A Retrospective Study from Two Italian MS Centers.

Lo Re M(1), Capobianco M(2), Ragonese P(3), Realmuto S(3), Malucchi S(2),

Berchialla P(4), Salemi G(3), Bertolotto A(2).

Author information:

(1)Department of Experimental Biomedicine and Clinical Neurosciences, University

of Palermo, Palermo, Italy. marianna.lore@gmail.com.

(2)Regional Multiple Sclerosis Centre, San Luigi Gonzaga Hospital, Orbassano,

Italy.

(3)Department of Experimental Biomedicine and Clinical Neurosciences, University

of Palermo, Palermo, Italy.

(4)Department of Clinical and Biological Sciences, University of Turin, Torino,

Italy.

INTRODUCTION: Natalizumab (NTZ) discontinuation can be followed by multiple

sclerosis (MS) disease reactivation. Currently no disease-modifying drug (DMD)

has been shown to be able to abolish disease reactivation. The aims of the

current study were: (1) to determine the frequency of MS reactivation after NTZ

discontinuation; (2) to evaluate predictors of reactivation risk, and (3) to

compare the effect of different treatments in reducing this risk.

METHODS: Data from 132 patients with MS followed-up for 2 years before NTZ

treatment and 1 year after interruption were collected from two Italian MS

centers and retrospectively evaluated.

RESULTS: Overall, 72 of 132 patients (54.5%) had relapses after NTZ

discontinuation and 60 of 125 patients (48%), who had magnetic resonance imaging,

had radiological reactivation. Rebound was observed in 28 of 132 patients

(21.2%). A higher number of relapses in the 2 years before NTZ treatment, a

longer washout period, and a lower number NTZ infusions correlated with

reactivation and rebound. Untreated patients (n = 37) had higher clinical and

radiological activity and rebound in comparison to patients receiving DMDs.

Moreover, a lower risk of relapses was found in patients treated with second-line

therapies (NTZ and fingolimod) than in those treated with first-line therapies

(interferon beta, glatiramer acetate, teriflunomide, azathioprine).

Interestingly, no disease reactivation in off-label treatment (rituximab,

autologous hematopoietic stem cell transplantation) was observed.

CONCLUSION: NTZ discontinuation is a risk for MS reactivation and rebound. An

alternative treatment should be promptly resumed mainly in patients with a

previous very active disease course and with a shorter NTZ therapy. Second-line

therapies demonstrate superiority in preventing relapses after NTZ

discontinuation.

DOI: 10.1007/s40120-015-0038-9

PMCID: PMC4685862

PMID: 26647006

157. Brain. 2015 Nov;138(Pt 11):3275-86. doi: 10.1093/brain/awv260. Epub 2015 Sep 11.

Excluded b/c no real-world adherence/persistence

Fingolimod versus interferon beta/glatiramer acetate after natalizumab suspension

in multiple sclerosis.

Iaffaldano P(1), Lucisano G(2), Pozzilli C(3), Brescia Morra V(4), Ghezzi A(5),

Millefiorini E(6), Patti F(7), Lugaresi A(8), Zimatore GB(9), Marrosu MG(10),

Amato MP(11), Bertolotto A(12), Bergamaschi R(13), Granella F(14), Coniglio

G(15), Tedeschi G(16), Sola P(17), Lus G(18), Ferrò MT(19), Iuliano G(20), Corea

F(21), Protti A(22), Cavalla P(23), Guareschi A(24), Rodegher M(25), Paolicelli

D(1), Tortorella C(1), Lepore V(26), Prosperini L(3), Saccà F(4), Baroncini D(5),

Comi G(25), Trojano M; Italian iMed-Web database.

Author information:

(1)1 Department of Basic Medical Sciences, Neurosciences and Sense Organs,

University of Bari "Aldo Moro", Piazza G. Cesare 11, 70124, Bari, Italy.

(2)1 Department of Basic Medical Sciences, Neurosciences and Sense Organs,

University of Bari "Aldo Moro", Piazza G. Cesare 11, 70124, Bari, Italy 2

Department of Clinical Pharmacology and Epidemiology, Mario Negri Sud Foundation,

Via Nazionale per Lanciano 8, 66030, Santa Maria Imbaro (CH), Italy.

(3)3 Multiple Sclerosis Center, S.Andrea Hospital, Dept. of Neurology and

Psychiatry, La Sapienza University, Via di Grottarossa, 1035, 00189, Rome, Italy.

(4)4 Department of Neurosciences, Reproductive and Odontostomatological Sciences,

University "Federico II", Via Pansini 5, 80131 Napoli, Italy.

(5)5 Multiple Sclerosis Center, S.Antonio Abate Hospital, Via Pastori 4, 21013

Gallarate (VA), Italy.

(6)6 Multiple Sclerosis Center, Policlinico Umberto I, La Sapienza University,

Viale dell'Università 30, 00185, Rome, Italy.

(7)7 Dipartimento di Scienze Mediche e Chirurgiche e Tecnologie Avanzate, GF

Ingrassia, Sez. Neuroscienze, Centro Sclerosi Multipla, Università di Catania,

Via Santa Sofia 78, 95123 Catania, Italy.

(8)8 Department of Neuroscience, Imaging and Clinical Sciences, Multiple

Sclerosis Center, "SS. Annunziata" Hospital, University "G. D'Annunzio", Via dei

Vestini snc, 66100, Chieti, Italy.

(9)9 Operative Unit of Neurology, "Dimiccoli" General Hospital, Viale Ippocrate

15, 76121, Barletta, Italy.

(10)10 Multiple Sclerosis Center, University of Cagliari, Via Is Guadazzonis 2,

09126, Cagliari, Italy.

(11)11 Department of NEUROFARBA, University of Florence, Viale Pieraccini 6,

50139, Florence, Italy.

(12)12 Neurologia 2, CRESM (Centro Riferimento Regionale Sclerosi Multipla), AOU

S. Luigi, Regione Gonzole 10, 10043 Orbassano (TO), Italy.

(13)13 Inter-department Multiple Sclerosis Research Centre, C. Mondino National

Institute of Neurology Foundation, Via Mondino 2, 27100, Pavia, Italy.

(14)14 Department of Neurosciences, University of Parma, Via Volturno 39, 43125,

Parma, Italy.

(15)15 Neurology Unit, "Madonna delle Grazie" Hospital, Contrada Cattedra

Ambulante snc, 75100, Matera, Italy.

(16)16 Division of Neurology, Second University of Naples, Via Costantinopoli

104, 80138, Naples, Italy.

(17)17 Department of Neurosciences, Neurology Unit, University of Modena and

Reggio Emilia, Nuovo Ospedale Civile S. Agostino/Estense, Via Giardini 1355,

41126, Modena, Italy.

(18)18 Multiple Sclerosis Center, II Division of Neurology, Department of

Clinical and Experimental Medicine, Second University of Naples, Via Luciano

Armanni 5, 80138, Napoli, Italy.

(19)19 Neurological Department, "Maggiore" Hospital, Largo Dossena 2, 26013,

Crema, Italy.

(20)20 Department of Neurosciences, "S.Giovanni di Dio" Hospital, Largo Città di

Ippocrate, 84131, Salerno, Italy.

(21)21 Neurology Unit, "S.Giovanni Battista" Hospital, Via Arcamone, 06124,

Foligno, Italy.

(22)22 Multiple Sclerosis Center, Neurological Department, "Niguarda Ca' Granda"

Hospital, Piazza Ospedale Maggiore 3, 20162, Milan, Italy.

(23)23 Multiple Sclerosis Center, Department of Neuroscience, University of Turin

& City of Health and Science University Hospital of Turin, Via Verdi 8, 10124,

Turin, Italy.

(24)24 Multiple Sclerosis Center, Medicine Department, Fidenza Hospital, Via Don

Enrico Tincati 5, 43125 Fidenza, Italy.

(25)25 Department of Neurology, Vita-Salute San Raffaele University, San Raffaele

Scientific Institute, Via Olgettina, 48 20132, Milan, Italy.

(26)2 Department of Clinical Pharmacology and Epidemiology, Mario Negri Sud

Foundation, Via Nazionale per Lanciano 8, 66030, Santa Maria Imbaro (CH), Italy.

The comparative effectiveness of fingolimod versus interferon beta/glatiramer

acetate was assessed in a multicentre, observational, prospectively acquired

cohort study including 613 patients with relapsing multiple sclerosis

discontinuing natalizumab in the Italian iMedWeb registry. First, after

natalizumab suspension, the relapse risk during the untreated wash-out period and

during the course of switch therapies was estimated through Poisson regression

analyses in separated models. During the wash-out period an increased risk of

relapses was found in patients with a higher number of relapses before

natalizumab treatment (incidence rate ratio = 1.31, P = 0.0014) and in patients

discontinuing natalizumab due to lack of efficacy (incidence rate ratio = 2.33, P

= 0.0288), patient's choice (incidence rate ratio = 2.18, P = 0.0064) and adverse

events (incidence rate ratio = 2.09, P = 0.0084). The strongest independent

factors influencing the relapse risk after the start of switch therapies were a

wash-out duration longer than 3 months (incidence rate ratio = 1.78, P < 0.0001),

the number of relapses experienced during and before natalizumab treatment

(incidence rate ratio = 1.61, P < 0.0001; incidence rate ratio = 1.13, P =

0.0118, respectively) and the presence of comorbidities (incidence rate ratio =

1.4, P = 0.0097). Switching to fingolimod was associated with a 64% reduction of

the adjusted-risk for relapse in comparison with switching to interferon

beta/glatiramer acetate (incidence rate ratio = 0.36, P < 0.0001). Secondly,

patients who switched to fingolimod or to interferon beta/glatiramer acetate were

propensity score-matched on a 1-to-1 basis at the switching date. In the

propensity score-matched sample a Poisson model showed a significant lower

incidence of relapses in patients treated with fingolimod in comparison with

those treated with interferon beta/glatiramer acetate (incidence rate ratio =

0.52, P = 0.0003) during a 12-month follow-up. The cumulative probability of a

first relapse after the treatment switch was significantly lower in patients

receiving fingolimod than in those receiving interferon beta/glatiramer acetate

(P = 0.028). The robustness of this result was also confirmed by sensitivity

analyses in subgroups with different wash-out durations (less or more than 3

months). Time to 3-month confirmed disability progression was not significantly

different between the two groups (Hazard ratio = 0.58; P = 0.1931). Our results

indicate a superiority of fingolimod in comparison to interferon beta/glatiramer

acetate in controlling disease reactivation after natalizumab discontinuation in

the real life setting.

© The Author (2015). Published by Oxford University Press on behalf of the

Guarantors of Brain. All rights reserved. For Permissions, please email:

journals.permissions@oup.com.

DOI: 10.1093/brain/awv260

PMID: 26362907 [Indexed for MEDLINE]

158. Brain. 2015 Nov;138(Pt 11):3287-98. doi: 10.1093/brain/awv258. Epub 2015 Sep 10.

Excluded b/c no real-world adherence/persistence

Defining reliable disability outcomes in multiple sclerosis.

Kalincik T(1), Cutter G(2), Spelman T(3), Jokubaitis V(3), Havrdova E(4),

Horakova D(4), Trojano M(5), Izquierdo G(6), Girard M(7), Duquette P(7), Prat

A(7), Lugaresi A(8), Grand'Maison F(9), Grammond P(10), Hupperts R(11),

Oreja-Guevara C(12), Boz C(13), Pucci E(14), Bergamaschi R(15), Lechner-Scott

J(16), Alroughani R(17), Van Pesch V(18), Iuliano G(19), Fernandez-Bolaños R(20),

Ramo C(21), Terzi M(22), Slee M(23), Spitaleri D(24), Verheul F(25), Cristiano

E(26), Sánchez-Menoyo JL(27), Fiol M(28), Gray O(29), Cabrera-Gomez JA(30),

Barnett M(31), Butzkueven H(32).

Author information:

(1)1 Department of Medicine, University of Melbourne, Melbourne, Australia 2

Department of Neurology, Royal Melbourne Hospital, Melbourne, Australia

tomas.kalincik@unimelb.edu.au.

(2)3 Department of Biostatistics, University of Alabama, Tuscaloosa, AL, USA.

(3)1 Department of Medicine, University of Melbourne, Melbourne, Australia.

(4)4 Department of Neurology and Center of Clinical Neuroscience, General

University Hospital and Charles University in Prague, Czech Republic.

(5)5 Department of Basic Medical Sciences, Neuroscience and Sense Organs,

University of Bari, Bari, Italy.

(6)6 Department of Neurology, Hospital Universitario Virgen Macarena, Sevilla,

Spain.

(7)7 Department of Neurology, Hôpital Notre Dame, Montreal, Canada.

(8)8 MS Center, Department of Neuroscience, Imaging and Clinical Sciences,

University 'G. d'Annunzio', Chieti, Italy.

(9)9 Neuro Rive-Sud, Hôpital Charles LeMoyne, Quebec, Canada.

(10)10 Department of Neurology, Hotel-Dieu de Levis, Quebec, Canada.

(11)11 Zuyderland Ziekenhus, Sittard-Geleen, The Netherlands.

(12)12 Multiple Sclerosis Unit, University Hospital San Carlos, Madrid, Spain.

(13)13 Karadeniz Technical University, Trabzon, Turkey.

(14)14 Neurology Unit, Azienda Sanitaria Unica Regionale Marche - AV3, Macerata,

Italy.

(15)15 C. Mondino National Neurological Institute, Pavia, Italy.

(16)16 Hunter Medical Research Institute, University Newcastle, Newcastle,

Australia.

(17)17 Amiri Hospital, Kuwait City, Kuwait.

(18)18 Cliniques Universitaires Saint-Luc, Brussels, Belgium.

(19)19 Ospedali Riuniti di Salerno, Salerno, Italy.

(20)20 Hospital Universitario Virgen de Valme, Seville, Spain.

(21)21 Hospital Germans Trias i Pujol, Badalona, Spain.

(22)22 Medical Faculty, Department of Neurology, Ondokuz Mayis University,

Samsun, Turkey.

(23)23 Flinders University and Medical Centre, Adelaide, Australia.

(24)24 Azienda Ospedaliera di Rilievo Nazionale, San Giuseppe Moscati, Avellino,

Italy.

(25)25 Groene Hart Ziekenhuis, Gouda, The Netherlands.

(26)26 Hospital Italiano, Buenos Aires, Argentina.

(27)27 Department of Neurology, Hospital de Galdakao-Usansolo, Galdakao, Spain.

(28)28 Fundacion para la Lucha contra las Enfermedades Neurologicas de la

Infancia, Buenos Aires, Argentina.

(29)29 Craigavon Area Hospital, Portadown, UK.

(30)30 Centro Internacional de Restauracion Neurologica, Havana, Cuba.

(31)31 Brain and Mind Centre, Sydney, Australia.

(32)1 Department of Medicine, University of Melbourne, Melbourne, Australia 2

Department of Neurology, Royal Melbourne Hospital, Melbourne, Australia 32

Department of Neurology, Box Hill Hospital, Monash University, Melbourne,

Australia.

Prevention of irreversible disability is currently the most important goal of

disease modifying therapy for multiple sclerosis. The disability outcomes used in

most clinical trials rely on progression of Expanded Disability Status Scale

score confirmed over 3 or 6 months. However, sensitivity and stability of this

metric has not been extensively evaluated. Using the global MSBase cohort study,

we evaluated 48 criteria of disability progression, testing three definitions of

baseline disability, two definitions of progression magnitude, two definitions of

long-term irreversibility and four definitions of event confirmation period. The

study outcomes comprised the rates of detected progression events per 10 years

and the proportions of the recorded events persistent at later time points. To

evaluate the ratio of progression frequency and stability for each criterion, we

calculated the proportion of events persistent over the five subsequent years

once progression was achieved. Finally, we evaluated the clinical and demographic

determinants characterising progression events and, for those that regressed back

to baseline, determinants of their subsequent regression. The study population

consisted of 16 636 patients with the minimum of three recorded disability

scores, totalling 112 584 patient-years. The progression rates varied between

0.41 and 1.14 events per 10 years, with the length of required confirmation

interval as the most important determinant of the observed variance. The

concordance among all tested progression criteria was only 17.3%. Regression of

disability occurred in 11-34% of the progression events over the five subsequent

years. The most important determinant of progression stability was the length of

the confirmation period. For the most accurate set of the progression criteria,

the proportions of 3-, 6-, 12- or 24-month confirmed events persistent over 5

years reached 70%, 74%, 80% and 89%, respectively. Regression post progression

was more common in younger patients, relapsing-remitting disease course, and

after a smaller change in disability, and was inflated by higher visit frequency.

These results suggest that the disability outcomes based on 3-6-month confirmed

disability progression overestimate the accumulation of permanent disability by

up to 30%. This could lead to spurious results in short-term clinical trials, and

the issue may be magnified further in cohorts consisting predominantly of younger

patients and patients with relapsing-remitting disease. Extension of the required

confirmation period increases the persistence of progression events.

© The Author (2015). Published by Oxford University Press on behalf of the

Guarantors of Brain. All rights reserved. For Permissions, please email:

journals.permissions@oup.com.

DOI: 10.1093/brain/awv258

PMID: 26359291 [Indexed for MEDLINE]

Excluded b/c abstract only

159. Value Health. 2015 Nov;18(7):A766. doi: 10.1016/j.jval.2015.09.2514. Epub 2015

Oct 20.

Adherence to Glatiramer Acetate 40 Mg Versus Oral Disease-Modifying Therapies for

Multiple Sclerosis.

Halpern R(1), Wolbeck R(1), Blauer-Peterson C(1), Wu Y(2), Gandhi SK(2).

Author information:

(1)Optum, Eden Prairie, MN, USA.

(2)Teva Pharmaceuticals Inc, Malvern, PA, USA.

DOI: 10.1016/j.jval.2015.09.2514

PMID: 26534289

160. Value Health. 2015 Nov;18(7):A764. doi: 10.1016/j.jval.2015.09.2504. Epub 2015

Excluded b/c abstract only

Oct 20.

Differences In Multiple Sclerosis Relapse Rates Based On Patient Adherence,

Average Daily Dose, And Persistence With Disease-Modifying Therapy: Observations

Based on Real-World Data.

Irwin DE(1), Cappell KA(2), Davis BM(2), Wu Y(3), Grinspan A(4), Gandhi SK(3).

Author information:

(1)Truven Health Analytics, Chapel Hill, NC, USA.

(2)Truven Health Analytics, Ann Arbor, MI, USA.

(3)Teva Pharmaceuticals Inc, Malvern, PA, USA.

(4)Teva Pharmaceuticals, Weston, FL, USA.

DOI: 10.1016/j.jval.2015.09.2504

PMID: 26534280

161. Value Health. 2015 Nov;18(7):A760. doi: 10.1016/j.jval.2015.09.2481. Epub 2015

Oct 20.

Excluded b/c abstract only

Comparison of Compliance And Discontinuation Rates Among Ms Patients Treated With

Fingolimod and Other Disease-Modifying Therapies: A Canadian Retrospective Claims

Analysis.

Haddad P(1), Duquette P(2), Yeung M(3), Lamarche A(1), Fraelic A(4), Chan S(4).

Author information:

(1)Novartis, Dorval, QC, Canada.

(2)Notre Dame Hospital, Montréal, QC, Canada.

(3)University of Calgary Multiple Sclerosis Clinic, Calgary, NS, Canada.

(4)IMS Health Canada inc., Montreal, QC, Canada.

DOI: 10.1016/j.jval.2015.09.2481

PMID: 26534256

162. Mult Scler J Exp Transl Clin. 2015 Oct 5;1:2055217315609629. doi:

Excluded b/c no real-world adherence/persistence

10.1177/2055217315609629. eCollection 2015 Jan-Dec.

Remotely-delivered cognitive remediation in multiple sclerosis (MS): protocol and

results from a pilot study.

Charvet LE, Shaw MT, Haider L, Melville P, Krupp LB(1).

Author information:

(1)Department of Neurology, Stony Brook Medicine, Stony Brook, NY, USA.

BACKGROUND: Cognitive impairment represents a critical unmet treatment need in

multiple sclerosis (MS). Cognitive remediation is promising but traditionally

requires multiple clinic visits to access treatment. Computer-based programs

provide remote access to intensive and individually-adapted training.

OBJECTIVE: Our goal was to develop a protocol for remotely-supervised cognitive

remediation that enables individuals with MS to participate from home while

maintaining the standards for clinical study.

METHODS: MS participants (n = 20) were randomized to either an active cognitive

remediation program (n = 11) or a control condition of ordinary computer games

(n = 9). Participants were provided study laptops to complete training for five

days per week over 12 weeks, targeting a total of 30 hours. Treatment effects

were measured with composite change via scores of a repeated neuropsychological

battery.

RESULTS: Compliance was high with an average of 25.0 hours of program use (80% of

the target) and did not differ between conditions (25.7 vs. 24.2 mean hours,

p = 0.80). The active vs. control participants significantly improved in both the

cognitive measures (mean composite z-score change of 0.46 ± 0.59 improvement

vs. -0.14 ± 0.48 decline, p = 0.02) and motor tasks (mean composite z-score

change of 0.40 ± 0.71improvement vs. -0.64 ± 0.73 decline, p = 0.005).

CONCLUSIONS: Remotely-supervised cognitive remediation is feasible for clinical

study with potential for meaningful benefit in MS.

DOI: 10.1177/2055217315609629

PMCID: PMC5433334

PMID: 28607707

163. BMC Neurol. 2015 Sep 22;15:170. doi: 10.1186/s12883-015-0426-x.

Excluded b/c no oral DMD results

Adherence and cost in multiple sclerosis patients treated with IM IFN beta-1a:

impact of the CARE patient management program.

Katsarava Z(1)(2), Ehlken B(3), Limmroth V(4), Taipale K(5), Patel SN(6),

Niemczyk G(7), Rehberg-Weber K(8), Wernsdörfer C(9); C.A.R.E. Study Group.

Author information:

(1)Department of Neurology, Evangelic Hospital Unna, Holbeinstr. 10, 59423, Unna,

Germany. Zaza.Katsarava@uk-essen.de.

(2)Department of Neurology, University Hospital Essen, Essen, Germany.

Zaza.Katsarava@uk-essen.de.

(3)IMS HEALTH, Munich, Germany. BEhlken@de.imshealth.com.

(4)Department of Neurology, Cologne City Hospitals, University of Cologne,

Cologne, Germany. LimmrothV@kliniken-koeln.de.

(5)Biogen GmbH, Ismaning, Germany. kirsi.taipale@biogen.com.

(6)Biogen GmbH, Ismaning, Germany. sarita.noemi.patel@biogen.com.

(7)Biogen GmbH, Ismaning, Germany. gabriele.niemczyk@biogen.com.

(8)Biogen GmbH, Ismaning, Germany. karin.rehberg-weber@biogen.com.

(9)Biogen GmbH, Ismaning, Germany. colin.wernsdoerfer@biogen.com.

BACKGROUND: Disease modifying treatments (DMT) for MS such as interferon beta

(IFNβ) have been shown to reduce the risk for disease progression. Therefore

adherence to treatment is essential for treatment outcome.Here we want to

evaluate if participation in a patient management program (PMP) improves

adherence to DMT as well as health and cost outcomes associated with MS.

METHODS: In this open-label multicentre prospective observational study, German

MS patients treated with once weekly intramuscular (IM) IFNβ-1a (Avonex), were

offered participation in a PMP and followed for up to 12 months. The PMP included

injection trainings, support and quarterly visits for up to 12 months after

initiation of therapy. Utilisation of health care services was evaluated. The

primary endpoint was to evaluate the direct and indirect cost associated with MS

from payer, patient and societal perspective, in patients who participate in the

PMP. Secondary endpoint was the clinical outcome in patients who participate in

the PMP (differentiated in adherent versus non-adherent patients).

RESULTS: In total 731 patients (mean age: 38.2, 73.7% female) were enrolled, 640

(88%) were observed for twelve months. After six months 34% of patients had

participated in the PMP continuously and 21% temporarily; 39% had not

participated. After twelve months, the proportions of participants were: 37%

continuously and 19% temporarily; 40% had not participated. After 6 months, mean

reduction in cost per patient in the participants group (€ 2151) was almost twice

as high as the cost reduction amongst non-participants (€ 1131). After twelve

months, the annual relapse rate was reduced by 58% compared to baseline in both

the participant and non-participant groups.

CONCLUSIONS: In a real-world-setting, participation in a patient management

program was associated with improved medication adherence and lower total

MS-related direct and indirect cost over time.

DOI: 10.1186/s12883-015-0426-x

PMCID: PMC4580346

PMID: 26395989 [Indexed for MEDLINE]

164. Int J Mol Sci. 2015 Sep 10;16(9):21832-45. doi: 10.3390/ijms160921832.

Excluded b/c no real-world adherence/persistence

Adaptive Immune Responses in a Multiple Sclerosis Patient with Acute

Varicella-Zoster Virus Reactivation during Treatment with Fingolimod.

Harrer A(1), Wipfler P(2), Pilz G(3), Oppermann K(4), Haschke-Becher E(5), Afazel

S(6), Kraus J(7)(8), Trinka E(9), Sellner J(10)(11).

Author information:

(1)Department of Neurology, Christian Doppler Medical Center, Paracelsus Medical

University, 5020 Salzburg, Austria. a.harrer@salk.at.

(2)Department of Neurology, Christian Doppler Medical Center, Paracelsus Medical

University, 5020 Salzburg, Austria. p.wipfler@salk.at.

(3)Department of Neurology, Christian Doppler Medical Center, Paracelsus Medical

University, 5020 Salzburg, Austria. georg.pilz@salk.at.

(4)Department of Neurology, Christian Doppler Medical Center, Paracelsus Medical

University, 5020 Salzburg, Austria. k.oppermann@salk.at.

(5)Department of Laboratory Medicine, Paracelsus Medical University, 5020

Salzburg, Austria.

(6)Department of Laboratory Medicine, Paracelsus Medical University, 5020

Salzburg, Austria. s.afazel@salk.at.

(7)Department of Neurology, A.ö. Krankenhaus Zell am See, Teaching Hospital of

the Paracelsus Medical University, 5700 Zell am See, Austria.

joerg.kraus@kh-zellamsee.at.

(8)Research Institute of Neurointervention, Paracelsus Medical University, 5020

Salzburg, Austria. joerg.kraus@kh-zellamsee.at.

(9)Department of Neurology, Christian Doppler Medical Center, Paracelsus Medical

University, 5020 Salzburg, Austria. e.trinka@salk.at.

(10)Department of Neurology, Christian Doppler Medical Center, Paracelsus Medical

University, 5020 Salzburg, Austria. j.sellner@salk.at.

(11)Department of Neurology, Klinikum rechts der Isar, Technische Universität,

81675 München, Germany. j.sellner@salk.at.

Fingolimod, an oral sphingosine 1-phosphate (S1P) receptor modulator, is approved

for the treatment of relapsing forms of multiple sclerosis (MS). The interference

with S1P signaling leads to retention particularly of chemokine receptor-7 (CCR7)

expressing T cells in lymph nodes. The immunological basis of varicella zoster

virus (VZV) infections during fingolimod treatment is unclear. Here, we studied

the dynamics of systemic and intrathecal immune responses associated with

symptomatic VZV reactivation including cessation of fingolimod and initiation of

antiviral therapy. Key features in peripheral blood were an about two-fold

increase of VZV-specific IgG at diagnosis of VZV reactivation as compared to the

previous months, a relative enrichment of effector CD4+ T cells (36% versus mean

12% in controls), and an accelerated reconstitution of absolute lymphocytes

counts including a normalized CD4+/CD8+ ratio and reappearance of CCR7+ T cells.

In cerebrospinal fluid (CSF) the lymphocytic pleocytosis and CD4+/CD8+ ratios at

diagnosis of reactivation and after nine days of fingolimod discontinuation

remained unchanged. During this time CCR7+ T cells were not observed in CSF.

Further research into fingolimod-associated VZV reactivation and immune

reconstitution is mandatory to prevent morbidity and mortality associated with

this potentially life-threatening condition.

DOI: 10.3390/ijms160921832

PMCID: PMC4613283

PMID: 26378517 [Indexed for MEDLINE]

165. Arthritis Res Ther. 2015 Sep 8;17:244. doi: 10.1186/s13075-015-0739-6.

Excluded b/c no real-world adherence/persistence

IL2RA is associated with persistence of rheumatoid arthritis.

van Steenbergen HW(1), van Nies JA(2), Ruyssen-Witrand A(3), Huizinga TW(4),

Cantagrel A(5), Berenbaum F(6), van der Helm-van Mil AH(7).

Author information:

(1)Department of Rheumatology, Leiden University Medical Center, P.O. Box 9600,

2300, RC, Leiden, The Netherlands. h.w.van_steenbergen@lumc.nl.

(2)Department of Rheumatology, Leiden University Medical Center, P.O. Box 9600,

2300, RC, Leiden, The Netherlands. j.a.b.van_nies@lumc.nl.

(3)Department of Rheumatology, Toulouse University Hospital, Toulouse, 31059

Toulouse cedex 9, France. adruyssen@hotmail.com.

(4)Department of Rheumatology, Leiden University Medical Center, P.O. Box 9600,

2300, RC, Leiden, The Netherlands. t.w.j.huizinga@lumc.nl.

(5)Department of Rheumatology, Toulouse University Hospital, Toulouse, 31059

Toulouse cedex 9, France. cantagrel.a@chu-toulouse.fr.

(6)University of Paris 06 UPMC, UMR_S-938, 75005 Paris France, Department of

Rheumatology, AP-HP, Saint-Antoine Hospital, 184, rue du Faubourg-Saint-Antoine,

75012, Paris, France. francis.berenbaum@sat.aphp.fr.

(7)Department of Rheumatology, Leiden University Medical Center, P.O. Box 9600,

2300, RC, Leiden, The Netherlands. a.h.m.van_der_helm@lumc.nl.

INTRODUCTION: Although rheumatoid arthritis (RA) is generally a chronic disease,

a proportion of RA-patients achieve disease-modifying antirheumatic drug

(DMARD)-free sustained remission, reflecting loss of disease-persistence. To

explore mechanisms underlying RA-persistence, we performed a candidate gene

study. We hypothesized that variants associating with lack of radiographic

progression also associate with DMARD-free sustained remission.

METHODS: 645 Dutch RA-patients were studied on DMARD-free sustained remission

during a maximal follow-up duration of 10-years. Variants associated with

radiographic progression under an additive model in the total RA-population

(Human Leukocyte Antigens (HLA)-DRB1-shared epitope (SE), Dickkopf-1

(DKK1)-rs1896368, DKK1-rs1896367, DKK1-rs1528873, C5Orf30-rs26232, Interleukin-2

receptor-α (IL2RA)-rs2104286, Matrix metalloproteinase-9 (MMP-9)-rs11908352,

rs451066 and Osteoprotegerin (OPG)-rs1485305) were studied. Cox-regression

analyses were performed and Bonferroni correction applied. Soluble IL2Rα

(sIL2Rα)-levels were studied. For replication, 622 RA-patients included in the

French Evaluation et Suivi de POlyarthrites Indifférenciées Récentes cohort

(ESPOIR)-cohort were investigated. Results were combined in inverse-variance

weighted meta-analysis.

RESULTS: Similar as previously reported, the SE-alleles associated with less

remission (hazard ratio (HR) = 0.57, 95 % confidence interval (95 %

CI) = 0.42-0.77, p = 2.72×10(-4)). Variants in DKK-1, C5orf30, MMP-9 and OPG were

not associated with remission. The IL2RA-rs2104286 minor allele associated with a

higher chance on remission (HR = 1.52, 95 % CI = 1.16-1.99, p = 2.44×10(-3)). The

rs2104286 minor allele associated with lower sIL2Rα-levels (p = 1.44×10(-3));

lower sIL2Rα-levels associated with a higher chance on remission (HR per 100

pg/L = 0.81, 95 % CI = 0.68-0.95, p = 0.012). When including rs2104286 and

sIL2Rα-levels in one analysis, the HR for rs2104286 was 2.27 (95 %

CI = 1.06-4.84, p = 0.034) and for sIL2Rα 0.83 (95 % CI = 0.70-0.98, p = 0.026).

Within ESPOIR, the HR of rs2104286 was 1.31 (95 % CI = 0.90-1.90). The

meta-analysis revealed a p-value of 1.01×10(-3).

CONCLUSION: IL2RA-rs2104286 and sIL2Rα-level associated with RA-persistence.

IL2RA variants are known to protect against multiple sclerosis, diabetes mellitus

and RA. Besides HLA-SE, IL2RA-rs2104286 is thus far the only known genetic

variant associated with both joint destruction and RA-persistence. This

underlines the relevance of IL2RA for RA.

DOI: 10.1186/s13075-015-0739-6

PMCID: PMC4563834

PMID: 26350950 [Indexed for MEDLINE]

166. Int J MS Care. 2015 Sep-Oct;17(5):236-43. doi: 10.7224/1537-2073.2014-069.

Excluded b/c no real-world adherence/persistence

Clinical Significance of Gastrointestinal and Flushing Events in Patients with

Multiple Sclerosis Treated with Delayed-Release Dimethyl Fumarate.

Phillips JT(1), Selmaj K(1), Gold R(1), Fox RJ(1), Havrdova E(1), Giovannoni

G(1), Abourjaily H(1), Pace A(1), Novas M(1), Hotermans C(1), Viglietta V(1),

Meltzer L(1).

Author information:

(1)Multiple Sclerosis Program, Baylor Institute for Immunology Research, Dallas,

TX, USA (JTP); Medical University of Lodz, Lodz, Poland (KS); St. Josef Hospital,

Ruhr University, Bochum, Germany (RG); Mellen Center for Multiple Sclerosis

Treatment and Research, Cleveland Clinic, Cleveland, OH, USA (RJF); Department of

Neurology, First Faculty of Medicine, Charles University in Prague, Prague, Czech

Republic (EH); Queen Mary University of London, Blizard Institute, Barts and the

London School of Medicine and Dentistry, London, UK (GG); and Biogen, Cambridge,

MA, USA (HA, AP, MN, CH, VV, LM).

BACKGROUND: In the phase 3 DEFINE and CONFIRM trials, flushing and

gastrointestinal (GI) events were associated with delayed-release dimethyl

fumarate (DMF; also known as gastroresistant DMF) treatment in people with

relapsing-remitting multiple sclerosis (MS). To investigate these events, a post

hoc analysis of integrated data from these trials was conducted, focusing on the

initial treatment period (months 0-3) with the recommended DMF dosage (240 mg

twice daily).

METHODS: Eligibility criteria included age 18 to 55 years, relapsing-remitting MS

diagnosis, and Expanded Disability Status Scale score 0 to 5.0. Patients were

randomized and received treatment with placebo (n = 771) or DMF (n = 769) for up

to 2 years. Adverse events were recorded at scheduled clinic visits every 4

weeks.

RESULTS: The incidence of GI and flushing events was highest in the first month

of treatment. In months 0 to 3, the incidence of GI events was 17% in the placebo

group and 27% in the DMF group and the incidence of flushing and related symptoms

was 5% in the placebo group and 37% in the DMF group. Most GI and flushing events

were of mild or moderate severity and resolved during the study. The events were

temporally associated with the use of diverse symptomatic therapies (efficacy not

assessed) and infrequently led to DMF discontinuation.

CONCLUSIONS: This integrated analysis indicates that in a clinical trial setting,

GI and flushing events associated with DMF treatment are generally transient and

mild or moderate in severity and uncommonly lead to treatment discontinuation.

DOI: 10.7224/1537-2073.2014-069

PMCID: PMC4599361

PMID: 26472945

167. Int J MS Care. 2015 Sep-Oct;17(5):231-5. doi: 10.7224/1537-2073.2014-078.

Excluded b/c no real-world adherence/persistence

Plasma Exchange in a Patient with Tumefactive, Corticosteroid-Resistant Multiple

Sclerosis.

Ikeda KM(1), Lee DH(1), Fraser JA(1), Mirsattari S(1), Morrow SA(1).

Author information:

(1)Schulich School of Medicine and Dentistry, Western Uni-versity, London,

Ontario, Canada (KMI, DHL, JAF, SMM, SAM); and Department of Clinical

Neurological Sciences (KMI, JAF, SM, SAM) and Department of Radiology (DHL),

London Health Sciences Centre, London, Ontario, Canada.

Tumefactive multiple sclerosis (MS) is an aggressive form of MS that can be

difficult to treat with standard therapies. In severe MS relapses, plasma

exchange (PLEX) has shown some benefit, but reports of its use in patients with

tumefactive MS are limited. This article describes the successful use of PLEX in

a patient with tumefactive MS. A 46-year-old right-handed woman with a recent

diagnosis of MS presented with drowsiness, dysarthria, horizontal nystagmus, and

quadriparesis. Her brain magnetic resonance images demonstrated multiple

tumefactive demyelinating lesions in the medulla, bilateral periventricular white

matter, and corona radiata white matter. She was initially treated with a 10-day

course of intravenous methylprednisolone without benefit; therefore, PLEX was

initiated. After the second exchange, the patient started to improve and was

discharged initially to rehabilitation and then home. She was started on

disease-modifying therapy with natalizumab and did not experience further

relapses but had slow clinical decline during the next year, which led to

discontinuation of natalizumab treatment. PLEX may be used as second-line

treatment in corticosteroid-resistant MS relapses, but there are limited reports

of its use in patients with tumefactive MS. This patient presented with

aggressive disease with multiple tumefactive lesions and did not respond to

standard treatment with corticosteroids. PLEX was successful in improving her

symptoms, allowing her to return home, although the disease progressed during the

next year.

DOI: 10.7224/1537-2073.2014-078

PMCID: PMC4599360

PMID: 26472944

Excluded b/c no real-world adherence/persistence

168. Medsurg Nurs. 2015 Sep-Oct;24(5):336-41, 355.

Relation of Demographics and Reported Symptoms and Medication Use in Persons with

Multiple Sclerosis.

Newland P, Riley M, Foerster LA, Thomas FP.

A study using a cross-sectional descriptive design examined symptoms, race, sex,

and use of disease-modifying therapies (DMT) in persons with relapsing remitting

multiple sclerosis. Significant differences in symptoms and DMT use were found

among women, men, and African Americans.

PMID: 26665870 [Indexed for MEDLINE]

169. Mult Scler Relat Disord. 2015 Sep;4(5):444-450. doi: 10.1016/j.msard.2015.06.010.

Excluded b/c no real-world adherence/persistence

Epub 2015 Jun 19.

Optimizing treatment initiation: Effects of a patient education program about

fingolimod treatment on knowledge, self-efficacy and patient satisfaction.

Zimmer A(1), Bläuer C(2), Coslovsky M(3), Kappos L(4), Derfuss T(5).

Author information:

(1)Department of Neurology, University Hospital Basel, Petersgraben 4, 4031

Basel, Switzerland. Electronic address: andrea.zimmer@usb.ch.

(2)Department of Special Clinics, University Hospital Basel, Hebelstrasse 2, 4031

Basel, Switzerland. Electronic address: cornelia.blaeuer@usb.ch.

(3)Clinical Trial Unit, Department of Clinical Research, University Hospital

Basel, Schanzenstrasse 55, 4031 Basel, Switzerland. Electronic address:

michael.coslovsky@usb.ch.

(4)Departments of Neurology and Biomedicine, University Hospital Basel,

Petersgraben 4, 4031 Basel, Switzerland. Electronic address:

ludwig.kappos@usb.ch.

(5)Departments of Neurology and Biomedicine, University Hospital Basel,

Petersgraben 4, 4031 Basel, Switzerland. Electronic address:

tobias.derfuss@usb.ch.

BACKGROUND: Satisfaction with information on medication is linked to adherence,

but patients are often dissatisfied with information about medication.

Information about treatment with fingolimod is important for MS patients since an

active role in managing treatment is required from them. To facilitate optimal

treatment initiation, a nurse-led patient education program on fingolimod was

implemented in a Swiss MS center.

OBJECTIVE: To evaluate the impact of the evidence-based comprehensive patient

education program on knowledge, self-efficacy and patient satisfaction.

METHODS: Knowledge gain, subjective perception of being informed about the new

treatment, self-efficacy in handling it and satisfaction with the program were

evaluated in a pretest-posttest design. Patient reported outcomes were collected

before and after an educational session on the first-dose day at the MS Center in

a consecutive sample of 98 people with MS. Data was analyzed descriptively, score

comparisons were done by Wilcoxon tests, and associations were estimated with

Spearman's correlation coefficient.

RESULTS: Knowledge increased significantly from pre- to posttest. Similarly,

perception of being informed and self-efficacy increased significantly.

Satisfaction with the program was high. Pretest-differences in knowledge

concerning gender and marital status were balanced after the educational session.

Results did not differ between patients with fingolimod as first treatment and

those switching from other MS treatments. At posttest perception of being

informed and self-efficacy were significantly related to satisfaction.

CONCLUSIONS: An evidence-based comprehensive treatment education program is

suitable to satisfy MS patients' information needs at treatment initiation. It

enhances short-term treatment knowledge and self-efficacy in handling a new

treatment in daily life. MS Nurses can thus contribute to effective treatment

education and potentially to medication safety and adherence.

Copyright © 2015 Elsevier B.V. All rights reserved.

DOI: 10.1016/j.msard.2015.06.010

PMID: 26346793 [Indexed for MEDLINE]

Excluded b/c no primary data (narrative review)

170. Mult Scler Relat Disord. 2015 Sep;4(5):437-443. doi: 10.1016/j.msard.2015.07.004.

Epub 2015 Jul 8.

Stopping immunomodulatory medications in MS: Frequency, reasons and consequences.

Tobin WO(1), Weinshenker BG(2).

Author information:

(1)Department of Neurology, College of Medicine, Mayo Clinic, 200 First Street

Southwest, Rochester, MN 55905, USA.

(2)Department of Neurology, College of Medicine, Mayo Clinic, 200 First Street

Southwest, Rochester, MN 55905, USA. Electronic address: weinb@mayo.edu.

Erratum in

Mult Scler Relat Disord. 2016 Jan;5():105.

Prolonged periods of disease quiescence during long term treatment of patients

with clinically isolated syndrome and MS may prompt consideration of

discontinuing disease-modifying therapy (DMT). We reviewed pertinent studies to

identify the frequency and reasons for discontinuation of DMT.

Copyright © 2015 Elsevier B.V. All rights reserved.

DOI: 10.1016/j.msard.2015.07.004

PMID: 26346792 [Indexed for MEDLINE]

171. Mult Scler Relat Disord. 2015 Sep;4(5):406-408. doi: 10.1016/j.msard.2015.06.015.

Excluded b/c no real-world adherence/persistence

Epub 2015 Jul 2.

Acetazolamide therapy in a case of fingolimod-associated macular edema: early

benefits and long-term limitations.

Schröder K(1), Finis D(2), Harmel J(3), Ringelstein M(4), Hartung HP(5), Geerling

G(6), Aktas O(7), Guthoff R(8).

Author information:

(1)Department of Ophthalmology, Medical Faculty, Heinrich-Heine-University

Duesseldorf, Duesseldorf, Germany. Electronic address:

Katharina.Schroeder@med.uni-duesseldorf.de.

(2)Department of Ophthalmology, Medical Faculty, Heinrich-Heine-University

Duesseldorf, Duesseldorf, Germany. Electronic address:

David.Finis@med.uni-duesseldorf.de.

(3)Department of Neurology, Medical Faculty, Heinrich-Heine-University

Duesseldorf, Duesseldorf, Germany. Electronic address:

Jens.Harmel@med.uni-duesseldorf.de.

(4)Department of Neurology, Medical Faculty, Heinrich-Heine-University

Duesseldorf, Duesseldorf, Germany. Electronic address:

Marius.Ringelstein@med.uni-duesseldorf.de.

(5)Department of Neurology, Medical Faculty, Heinrich-Heine-University

Duesseldorf, Duesseldorf, Germany. Electronic address:

Hans-Peter.Hartung@med.uni-duesseldorf.de.

(6)Department of Ophthalmology, Medical Faculty, Heinrich-Heine-University

Duesseldorf, Duesseldorf, Germany. Electronic address:

Geerling@med.uni-duesseldorf.de.

(7)Department of Neurology, Medical Faculty, Heinrich-Heine-University

Duesseldorf, Duesseldorf, Germany. Electronic address:

orhan.aktas@med.uni-duesseldorf.de.

(8)Department of Ophthalmology, Medical Faculty, Heinrich-Heine-University

Duesseldorf, Duesseldorf, Germany. Electronic address:

Rainer.Guthoff@med.uni-duesseldorf.de.

Fingolimod is a potent drug in relapsing forms of multiple sclerosis. Visual

impairment due to fingolimod-associated macular edema (FAME) usually leads to

discontinuation of fingolimod therapy.METHODS: We report on a 24-year old woman

with bilateral FAME.

RESULTS: We continued fingolimod and added oral acetazolamide, which led to

recovery of visual acuity and regression of macular edema. However, fingolimod

had to be discontinued when fluorescein angiography revealed an enlarged foveal

avascular zone.

DISCUSSION AND CONCLUSION: Oral acetazolamide might be a treatment option for

FAME, while ischemic conversion may be limiting. Ophthalmologic assessments are

mandatory for follow-up when fingolimod therapy is continued after onset of FAME.

Copyright © 2015 Elsevier B.V. All rights reserved.

DOI: 10.1016/j.msard.2015.06.015

PMID: 26346788 [Indexed for MEDLINE]

Excluded b/c no real-world adherence/persistence

172. Ther Adv Neurol Disord. 2015 Sep;8(5):233-8. doi: 10.1177/1756285615594575.

Tumefactive multiple sclerosis lesions in two patients after cessation of

fingolimod treatment.

Faissner S(1), Hoepner R(2), Lukas C(3), Chan A(2), Gold R(2), Ellrichmann G(2).

Author information:

(1)Department of Neurology, St Josef-Hospital, Ruhr-University Bochum,Gudrunstr.

56, 44791 Bochum, Germany.

(2)Department of Neurology, St Josef-Hospital, Ruhr-University Bochum, Bochum,

Germany.

(3)Department of Radiology, St Josef-Hospital, Ruhr-University Bochum, Bochum,

Germany.

BACKGROUND: Fingolimod (FTY) is the first oral medication approved for multiple

sclerosis therapy. Until now, little has been known about the effects of FTY

withdrawal regarding disease activity and development of tumefactive

demyelinating lesions (TDLs), as already described in patients who discontinue

natalizumab.

METHODS: In this study we present the clinical and radiological findings of two

patients who had a severe rebound after FTY withdrawal and compare these with

patients identified by a PubMed data bank analysis using the search term

'fingolimod rebound'. In total, 10 patients, of whom three developed TDLs, are

presented.

RESULTS: Patients suffering from TDLs were free of clinical and radiological

signs of disease activity under FTY therapy (100% versus 57%, compared with

patients without TDLs) and had rebounds after a mean of 14.6 weeks (standard

deviation 11.5) [patients without TDLs 11.7 (standard deviation 3.4)].

CONCLUSION: We propose that a good therapeutic response to FTY might be

predisposing for a severe rebound after withdrawal. Consequently, therapy

switches should be planned carefully with a short therapy free interval.

DOI: 10.1177/1756285615594575

PMCID: PMC4622113

PMID: 26557898

173. BMC Neurol. 2015 Aug 19;15:141. doi: 10.1186/s12883-015-0399-9.

Excluded b/c no oral DMD results

Differential glatiramer acetate treatment persistence in treatment-naive patients

compared to patients previously treated with interferon.

Fernández-Fournier M(1), Tallón-Barranco A(2), Chamorro B(3), Martínez-Sánchez

P(4), Puertas I(5).

Author information:

(1)Clinical Neuroimmunology and Multiple Sclerosis Unit, La Paz University

Hospital, Madrid, Spain. fernandezfournier@hotmail.com.

(2)Clinical Neuroimmunology and Multiple Sclerosis Unit, La Paz University

Hospital, Madrid, Spain. atb1958@gmail.com.

(3)Clinical Neuroimmunology and Multiple Sclerosis Unit, La Paz University

Hospital, Madrid, Spain. beatrizlapaz2@gmail.com.

(4)Department of Neurology, La Paz University Hospital, Madrid, Spain.

patrindalo@hotmail.com.

(5)Clinical Neuroimmunology and Multiple Sclerosis Unit, La Paz University

Hospital, Madrid, Spain. inmapuertas@hotmail.com.

BACKGROUND: In the treatment of multiple sclerosis, a change of therapy is

considered after treatment failure or adverse events. Although disease modifying

drugs' (DMD) efficacy and side effects have been fully analysed in clinical

trials, the effects of previous therapy use are less well studied. We aimed to

study medication persistence with glatiramer acetate in treatment-naive patients

and in patients previously treated with interferon.

METHODS: A retrospective study of relapsing-remitting multiple sclerosis patients

treated with glatiramer acetate in an MS Unit of a Spanish University Hospital

(January 2004--September 2013). Treatment time on glatiramer acetate was studied.

Reasons for treatment discontinuation were considered as follows: lack of

efficacy, serious adverse event, injection-related side effect, pregnancy and

lost to follow-up. Use of prior DMD was registered and analysed. Homogeneity of

groups was analysed using Fisher's and Mann-Whitney's tests. The Kaplan Meier

method and Cox regression model were used to estimate time to and risk of

treatment discontinuation.

RESULTS: In total, 155 relapsing-remitting multiple sclerosis patients were

treated with glatiramer acetate: 100 treatment-naive patients and 55 treated

previously with interferon. At the end of the study, 76 patients (49.0%)

continued on glatiramer acetate (with an average treatment time (ATT) of 50.4

months, s.d.32.8) and 50 patients (32.3%) had switched therapy: 27 patients

(17.4%) for inefficacy (ATT 29.2 months, s.d.17.5), 20 patients (12.9%) for

injection site reactions (ATT 16.5 months, s.d.20.3) and 3 patients (1.9%) after

serious adverse events (ATT 15.7 months, s.d.15.1). ATT in our cohort was 39

months (s.d.30.0), median follow-up 34 months. Six months after glatiramer

acetate initiation, probability of persisting on GA was 91.4%, 82.5% after 12

months and 72.5% after 2 years. The risk of glatiramer acetate treatment

discontinuation was 2.8 [1.7 - 4.8] times greater for treatment-naive patients

than for patients treated previously with interferon and this was hardly modified

after adjusting for sex and age.

CONCLUSIONS: Glatiramer acetate was safe and useful with low rates of serious

adverse events and low rates of break-through disease. Injection intolerance

proved a major limitation to glatiramer acetate use. Patients who had been

previously treated with interferons presented a lower probability of glatiramer

acetate discontinuation than treatment-naive patients.

DOI: 10.1186/s12883-015-0399-9

PMCID: PMC4545781

PMID: 26286576 [Indexed for MEDLINE]

174. BMC Neurol. 2015 Aug 4;15:128. doi: 10.1186/s12883-015-0379-0.

Excluded b/c no real-world adherence/persistence

A prospective web-based patient-centred interactive study of long-term

disabilities, disabilities perception and health-related quality of life in

patients with multiple sclerosis in The Netherlands: the Dutch Multiple Sclerosis

Study protocol.

Jongen PJ(1)(2), Heerings M(3)(4), Lemmens WA(5), Donders R(6), van der Zande

A(7), van Noort E(8), Kool A(9).

Author information:

(1)Department of Community & Occupational Medicine, University Medical Centre

Groningen, Antonius Deusinglaan 1, 9713 AV, Groningen, The Netherlands.

p.j.h.jongen@rug.nl.

(2)MS4 Research Institute, Ubbergseweg 34, 6522 KJ, Nijmegen, The Netherlands.

p.j.h.jongen@rug.nl.

(3)MH-Advies & organisatiebureau, IJselstraat 81, 9406 TR, Assen, The

Netherlands. m.a.p.heerings@gmail.com.

(4)National MS Foundation The Netherlands, Mathenesserlaan 378, 3023 HB,

Rotterdam, The Netherlands. m.a.p.heerings@gmail.com.

(5)Department for Health Evidence, Radboud University Nijmegen Medical Centre,

P.O. Box 9101, 6500 HB, Nijmegen, The Netherlands. Wim.Lemmens@radboudumc.nl.

(6)Department for Health Evidence, Radboud University Nijmegen Medical Centre,

P.O. Box 9101, 6500 HB, Nijmegen, The Netherlands. Rogier.Donders@radboudumc.nl.

(7)National MS Foundation The Netherlands, Mathenesserlaan 378, 3023 HB,

Rotterdam, The Netherlands. anneke@nationaalmsfonds.nl.

(8)Curavista bv, Markt 9, 4931 BR, Geertruidenberg, The Netherlands.

Esther.vanNoort@curavista.nl.

(9)Curavista bv, Markt 9, 4931 BR, Geertruidenberg, The Netherlands.

anton.kool@curavista.nl.

BACKGROUND: In the past two decades the widespread use of disease modifying drugs

with moderate to strong efficacy has changed the natural course of multiple

sclerosis (MS). Health care professionals, researchers, patient organizations and

health authorities are in need of recent information about the objectified and

subjective long-term clinical outcomes in MS patients. Such information is

scarce.

METHODS/DESIGN: We started a prospective, web-based, patient-centred, interactive

study of long-term disabilities, disabilities perception and health-related

quality of life (HRQoL) in MS patients in The Netherlands (Dutch Multiple

Sclerosis Study). The study has an on online patient-driven inclusion and online

acquisition of patient-reported outcomes (PROs). At six-months intervals

participants complete the Multiple Sclerosis Impact Profile (MSIP) (disabilities

and disabilities perception in seven domains and four symptoms), the Multiple

Sclerosis Quality of Life-54 items (MSQoL-54), the Modified Fatigue Impact

Scale-5 items (MFIS-5) and the Leeds Multiple Sclerosis Quality of Life-8 items

(LMSQoL) questionnaires, and a Medication and Adherence Inventory. Every three

years the Expanded Disability Status Scale (EDSS) score is assessed by phone. The

monthly completion of the MFIS-5, LMSQoL and Medication and Adherence Inventory

is optional. Completed questionnaires and inventories, and automatically

generated scores are made available online to patients for self-monitoring and

self-management purposes, and to authorized health care professionals for the

evaluation of disease activity and of the effectiveness of treatments. Study

duration is planned to be 15 years. Results will be analyzed periodically using

means and standard deviations for continuous variables, and frequencies for

categorical variables. Relations between time points, variables, patient and

treatment characteristics will be evaluated in random effects repeated measures

models.

DISCUSSION: The Dutch Multiple Sclerosis Study is characterized by online

patient-driven inclusion; online data acquisition; the use of PROs; the optional

monthly completion of short questionnaires; the interactive use of personal study

data by patients and authorized health care professionals for self-monitoring,

self-management and multidisciplinary care; the expected representativeness of

the study sample; and a long-term time horizon. The study will provide valuable

data on long-term disabilities, disabilities perceptions and HRQoL in MS patients

in The Netherlands.

DOI: 10.1186/s12883-015-0379-0

PMCID: PMC4524214

PMID: 26238866 [Indexed for MEDLINE]

175. BMC Neurol. 2015 Jul 31;15:125. doi: 10.1186/s12883-015-0377-2.

Excluded b/c no real-world adherence/persistence

Dramatic recovery of steroid-refractory relapsed multiple sclerosis following

Fingolimod discontinuation using selective immune adsorption.

De Masi R(1)(2)(3), Accoto S(4), Orlando S(5), De Blasi V(6), Pasca S(7)(8),

Scarpello R(9), Spagnolo L(10), Idolo A(11), De Donno A(12).

Author information:

(1)Laboratory of Neuroproteomics, Multiple Sclerosis Centre, "F. Ferrari"

Hospital, 73042, Casarano, Lecce, Italy. dmsrrt@gmail.com.

(2)Multiple Sclerosis Centre, "F. Ferrari" Hospital, 73042, Casarano, Lecce,

Italy. dmsrrt@gmail.com.

(3)Complex Operative Unit of Neurology-Stroke Unit, "F. Ferrari" Hospital, 73042,

Casarano, Lecce, Italy. dmsrrt@gmail.com.

(4)Complex Operative Unit of Nephrology and Dialysis, "F. Ferrari" Hospital,

73042, Casarano, Lecce, Italy. salva.accoto@gmail.com.

(5)Laboratory of Neuroproteomics, Multiple Sclerosis Centre, "F. Ferrari"

Hospital, 73042, Casarano, Lecce, Italy. stephaniaorlando@gmail.com.

(6)Complex Operative Unit of Nephrology and Dialysis, "F. Ferrari" Hospital,

73042, Casarano, Lecce, Italy. vincenzo.deblasi@tin.it.

(7)Laboratory of Neuroproteomics, Multiple Sclerosis Centre, "F. Ferrari"

Hospital, 73042, Casarano, Lecce, Italy. sergio.pasca@virgilio.it.

(8)Multiple Sclerosis Centre, "F. Ferrari" Hospital, 73042, Casarano, Lecce,

Italy. sergio.pasca@virgilio.it.

(9)Complex Operative Unit of Neurology-Stroke Unit, "F. Ferrari" Hospital, 73042,

Casarano, Lecce, Italy. rocco.scarpello@gmail.com.

(10)Division of Neuroradiology, "F. Ferrari" Hospital, 73042, Casarano, Lecce,

Italy. leospagnolo@gmail.com.

(11)Department of Biological and Environmental Sciences and Technologies,

Laboratory of Hygiene, University of the Salento, Lecce, Italy.

adele.idolo@unisalento.it.

(12)Department of Biological and Environmental Sciences and Technologies,

Laboratory of Hygiene, University of the Salento, Lecce, Italy.

antonella.dedonno@unisalento.it.

BACKGROUND: Selective immune adsorption (SIA) is an emerging method for treating

immune-mediated neurological diseases, given its superior safety profile compared

to plasma exchange (PEX). However, the available literature concerning Multiple

Sclerosis includes no cases of SIA applied to steroid-refractory rebound after

Fingolimod discontinuation.

CASE PRESENTATION: Here we report the case of a 32-year-old woman suffering from

multiple sclerosis treated with Fingolimod and admitted to a Multiple Sclerosis

Centre after drug discontinuation due to the occurrence of lymphopenia. During

the few weeks preceding admission, the patient experienced progressive and severe

neurological deterioration that did not respond to an initial cycle of pulsed

high doses of intravenous 6-methyl prednisolone (IVMP). Given the ineffectiveness

of a second cycle of IVMP, the patient was treated with plasma immunoadsorption,

leading to dramatic functional recovery. The patient then started a

neuro-rehabilitation program. About one month after the final SIA procedure the

patient started Natalizumab-based therapy, while maintaining a stable

neurological condition. We noted significant modification of C3/C4 complement

components and total gamma globulin concentrations (IgG) during SIA.

CONCLUSIONS: Our observations show that however serious, steroid-refractory

neurological deterioration occurring after Fingolimod discontinuation in multiple

sclerosis can be treated with selective immune-adsorption therapy which thus

represents a good alternative in these cases. It could be speculated that this

clinical condition was associated with pattern II of demyelination, given the

good response to a form of treatment that acts on autoantibodies. Thus, SIA

represented an effective therapeutic strategy for this case of relapsed MS as

steroid-resistent rebound post Fingolimod cessation.

DOI: 10.1186/s12883-015-0377-2

PMCID: PMC4521477

PMID: 26227815 [Indexed for MEDLINE]

176. PLoS One. 2015 Jul 27;10(7):e0133279. doi: 10.1371/journal.pone.0133279.

Excluded b/c no oral DMD results

eCollection 2015.

Adherence to Disease Modifying Drugs among Patients with Multiple Sclerosis in

Germany: A Retrospective Cohort Study.

Hansen K(1), Schüssel K(2), Kieble M(2), Werning J(2), Schulz M(2), Friis R(3),

Pöhlau D(4), Schmitz N(5), Kugler J(6).

Author information:

(1)TU Dresden, Medizinische Fakultät, Lehrstuhl für Gesundheitswissenschaften /

Public Health, Dresden, Germany; TU Dresden, Medizinische Fakultät, Bereich

Allgemeinmedizin, Dresden, Germany.

(2)Deutsches Arzneiprüfungsinstitut e. V. (DAPI), Berlin, Germany.

(3)Department of Health Science, California State University, Long Beach,

California, United States of America.

(4)Kamillus-Klinik, Asbach, Germany.

(5)Douglas Mental Health University Institute, FBC Building, Montreal, Canada.

(6)TU Dresden, Medizinische Fakultät, Lehrstuhl für Gesundheitswissenschaften /

Public Health, Dresden, Germany.

BACKGROUND: Long-term therapies such as disease modifying therapy for Multiple

Sclerosis (MS) demand high levels of medication adherence in order to reach

acceptable outcomes. The objective of this study was to describe adherence to

four disease modifying drugs (DMDs) among statutorily insured patients within two

years following treatment initiation. These drugs were interferon beta-1a i.m.

(Avonex), interferon beta-1a s.c. (Rebif), interferon beta-1b s.c. (Betaferon)

and glatiramer acetate s.c. (Copaxone).

METHODS: This retrospective cohort study used pharmacy claims data from the data

warehouse of the German Institute for Drug Use Evaluation (DAPI) from 2001

through 2009. New or renewed DMD prescriptions in the years 2002 to 2006 were

identified and adherence was estimated during 730 days of follow-up by analyzing

the medication possession ratio (MPR) as proxy for compliance and persistence

defined as number of days from initiation of DMD therapy until discontinuation or

interruption.

FINDINGS: A total of 52,516 medication profiles or therapy cycles (11,891 Avonex,

14,060 Betaferon, 12,353 Copaxone and 14,212 Rebif) from 50,057 patients were

included into the analysis. Among the 4 cohorts, no clinically relevant

differences were found in available covariates. The Medication Possession Ratio

(MPR) measured overall compliance, which was 39.9% with a threshold MPR≥0.8.

There were small differences in the proportion of therapy cycles during which a

patient was compliant for the following medications: Avonex (42.8%), Betaferon

(40.6%), Rebif (39.2%), and Copaxone (37%). Overall persistence was 32.3% at the

end of the 24 months observation period, i.e. during only one third of all

included therapy cycles patients did not discontinue or interrupt DMD therapy.

There were also small differences in the proportion of therapy cycles during

which a patient was persistent as follows: Avonex (34.2%), Betaferon (33.4%),

Rebif (31.7%) and Copaxone (29.8%).

CONCLUSIONS: Two years after initiating MS-modifying therapy, only 30-40% of

patients were adherent to DMDs.

DOI: 10.1371/journal.pone.0133279

PMCID: PMC4516264

PMID: 26214805 [Indexed for MEDLINE]

177. Neurology. 2015 Jul 7;85(1):29-39. doi: 10.1212/WNL.0000000000001706. Epub 2015

Excluded b/c no real-world adherence/persistence

May 29.

Switching from natalizumab to fingolimod: A randomized, placebo-controlled study

in RRMS.

Kappos L(1), Radue EW(2), Comi G(2), Montalban X(2), Butzkueven H(2), Wiendl

H(2), Giovannoni G(2), Hartung HP(2), Derfuss T(2), Naegelin Y(2), Sprenger T(2),

Mueller-Lenke N(2), Griffiths S(2), von Rosenstiel P(2), Gottschalk R(2), Zhang

Y(2), Dahlke F(2), Tomic D(2); TOFINGO study group.

Author information:

(1)From Neurology, the Departments of Medicine, Clinical Research and Biomedicine

(L.K., T.D., Y.N., T.S.), the Medical Image Analysis Center (MIAC) (E.-W.R.,

T.S., N.M.-L.), and the Department of Radiology, Division of Neuroradiology

(T.S.), University Hospital, University of Basel, Switzerland; the Department of

Neuroscience (G.C.), Scientific Institute H. San Raffaele, University of Milan,

Italy; Vall d'Hebron University Hospital (X.M.), Barcelona, Spain; Royal

Melbourne Hospital (H.B.), Parkville, Australia; the Department of Neurology

(H.W.), University of Münster, Germany; the Neuroscience and Trauma Centre

(G.G.), Blizard Institute, Barts and The London School of Medicine and Dentistry,

London, UK; the Department of Neurology (H.-P.H.), Heinrich Heine University,

Düsseldorf, Germany; Oxford PharmaGenesis Ltd. (S.G.), Tubney Warren Barns,

Oxford, UK; Novartis Pharma AG (P.v.R., Y.Z., F.D., D.T.), Basel, Switzerland;

and Novartis Pharmaceutical Corporation (R.G.), East Hanover, NJ.

lkappos@uhbs.ch.

(2)From Neurology, the Departments of Medicine, Clinical Research and Biomedicine

(L.K., T.D., Y.N., T.S.), the Medical Image Analysis Center (MIAC) (E.-W.R.,

T.S., N.M.-L.), and the Department of Radiology, Division of Neuroradiology

(T.S.), University Hospital, University of Basel, Switzerland; the Department of

Neuroscience (G.C.), Scientific Institute H. San Raffaele, University of Milan,

Italy; Vall d'Hebron University Hospital (X.M.), Barcelona, Spain; Royal

Melbourne Hospital (H.B.), Parkville, Australia; the Department of Neurology

(H.W.), University of Münster, Germany; the Neuroscience and Trauma Centre

(G.G.), Blizard Institute, Barts and The London School of Medicine and Dentistry,

London, UK; the Department of Neurology (H.-P.H.), Heinrich Heine University,

Düsseldorf, Germany; Oxford PharmaGenesis Ltd. (S.G.), Tubney Warren Barns,

Oxford, UK; Novartis Pharma AG (P.v.R., Y.Z., F.D., D.T.), Basel, Switzerland;

and Novartis Pharmaceutical Corporation (R.G.), East Hanover, NJ.

Comment in

Neurology. 2015 Jul 7;85(1):14-5.

OBJECTIVE: To investigate the effect of different natalizumab washout (WO)

periods on recurrence of MRI and clinical disease activity in patients switching

from natalizumab to fingolimod.

METHODS: In this multicenter, double-blind, placebo-controlled trial (TOFINGO),

patients with relapsing-remitting multiple sclerosis (RRMS) were randomized 1:1:1

to 8-, 12-, or 16-week WO followed by fingolimod treatment over 32 weeks from

last natalizumab infusion (LNI). Brain MRI was performed at baseline and weeks 8,

12, 16, 20, and 24.

RESULTS: Of 142 enrolled and randomized patients, 112 (78.9%) completed the study

(8 weeks, n = 41/50; 12 weeks, n = 31/42; 16 weeks, n = 40/50). Number (95%

confidence interval [CI]) of active (new/newly enlarged T2) lesions from LNI

through 8 weeks of fingolimod treatment (primary outcome) was similar in the

8-week (2.1 [1.7-2.6]) and 12-week WO groups (1.7 [1.3-2.2]) and higher in the

16-week WO group (8.2 [7.3-9.1]). During the WO period only, the number (95% CI)

of active lesions increased with increasing WO duration (8 weeks, 0.4 [0.2-0.6];

12 weeks, 2.1 [1.6-2.6]; 16 weeks, 3.6 [3.0-4.2]). Over the 24 weeks from LNI,

gadolinium-enhancing T1 lesion counts were lower in the 8-week WO group (14.1

[5.67-22.53]) than in the 12-week (21.3 [1.41-41.19]) or 16-week (18.5

[8.40-28.60]) WO groups. More patients were relapse-free in the 8-week (88%) and

12-week (91%) WO groups than the 16-week WO group (84%). Sixty-eight percent of

patients experienced adverse events (mostly mild/moderate), with similar

incidence across groups. No unusually severe relapses or opportunistic infections

occurred.

CONCLUSIONS: Initiating fingolimod therapy 8-12 weeks after natalizumab

discontinuation is associated with a lower risk of MRI and clinical disease

reactivation than initiation after 16-week WO.

CLASSIFICATION OF EVIDENCE: This study provides Class II evidence that for

patients with RRMS switching from natalizumab to fingolimod, shorter natalizumab

WO periods are associated with less MRI disease activity than are longer WO

periods.

© 2015 American Academy of Neurology.

DOI: 10.1212/WNL.0000000000001706

PMCID: PMC4501941

PMID: 26024899 [Indexed for MEDLINE]

178. Adv Ther. 2015 Jul;32(7):626-35. doi: 10.1007/s12325-015-0224-2. Epub 2015 Jul

Excluded b/c no real-world adherence/persistence

14.

Safety and Tolerability of Fingolimod in Latin American Patients with

Relapsing-Remitting Multiple Sclerosis: The Open-Label FIRST LATAM Study.

Ordoñez-Boschetti L, Rey R, Cruz A, Sinha A, Reynolds T, Frider N, Alvarenga R.

Erratum in

Adv Ther. 2015 Jul;32(7):636.

INTRODUCTION: Fingolimod 0.5 mg is an orally active sphingosine 1-phosphate

receptor modulator approved for use in adults with relapsing multiple sclerosis

(MS). The efficacy and safety profile of fingolimod has been well characterized

in a large clinical development program. Here, we report the safety and

tolerability of fingolimod in relapsing-remitting MS (RRMS) patients from Latin

America.

METHODS: A total of 162 patients with RRMS, predominantly from Latin American

countries (138/162), were enrolled in this 16-week, single treatment arm,

open-label, multi-center study. Unlike the phase III pivotal studies, this study

permitted enrollment of patients with controlled diabetes, certain cardiac and

pulmonary conditions, older age, and higher baseline Expanded Disability Status

Scale. All patients were monitored clinically for a minimum of 6 hours after the

first dose. Safety and tolerability assessments were based on adverse events,

clinically notable laboratory abnormalities, vital signs, ophthalmic

examinations, and electrocardiograms.

RESULTS: Overall, the safety and tolerability profile was consistent with that

reported previously in phase 3 studies and the FIRST study. Adverse events (AEs)

were predominantly mild (n = 49, 35.5%) or moderate (n = 27, 19.6%). Three

patients (2.2%) discontinued fingolimod due to AEs. Infections were reported in

33 patients (23.9%) and were predominantly mild in nature (n = 28, 20.3%).

Increases in alanine aminotransferase enzymes of ≥3, ≥5 and ≥10 upper limit of

normal were reported in five (3.7%), three (2.2%) and one (0.7%) patients,

respectively. Hypertension cases (n = 3; 2.2%) did not result in treatment

discontinuation and were controlled with antihypertensive therapy. Following

first-dose administration, the majority of patients (90.6%) were discharged at

6 h. During the first-dose monitoring, 5 cases of bradycardia were reported; none

required extended monitoring or treatment for symptomatic bradycardia.

CONCLUSION: The first dose of fingolimod 0.5 mg was well tolerated in RRMS

patients from Latin America. The overall safety profile was clinically manageable

and consistent with previous fingolimod studies.

FUNDING: Novartis.

TRIAL REGISTRATION: ClinicalTrials.gov #NCT01497262.

DOI: 10.1007/s12325-015-0224-2

PMCID: PMC4522027

PMID: 26170105 [Indexed for MEDLINE]

Excluded b/c no real-world adherence/persistence

179. Retin Cases Brief Rep. 2015 Summer;9(3):195-7. doi: 10.1097/ICB.0000000000000136.

Acute retinal necrosis and immune reconstitution inflammatory syndrome in a

natalizumab-treated patient with multiple sclerosis.

Saraiva VS(1).

Author information:

(1)Department of Ophthalmology and Visual Sciences, Federal University of São

Paulo, São Paulo, Brazil.

PURPOSE: To describe a case of acute retinal necrosis and subsequent immune

reconstitution inflammatory syndrome in a patient with multiple sclerosis treated

with natalizumab.

METHODS: Case report.

RESULTS: A 51-year-old man with multiple sclerosis developed acute retinal

necrosis during treatment with natalizumab. The patient was successfully managed

with natalizumab discontinuation, oral valacyclovir, and prophylactic laser

retinal photocoagulation. A few months after natalizumab interruption and 1 month

after valacyclovir completion, the patient presented with an episode of presumed

noninfectious anterior and intermediate uveitis, which responded well to oral

steroid mini-pulse therapy.

CONCLUSION: Natalizumab is a potent immune suppressor used in the treatment of

multiple sclerosis and Crohn disease. It has already been linked to herpetic

infections and immune reconstitution inflammatory syndrome in the central nervous

system. These complications may also affect ocular tissues.

DOI: 10.1097/ICB.0000000000000136

PMID: 25933353 [Indexed for MEDLINE]

Excluded b/c no primary data (study design)

180. BMC Neurol. 2015 Jun 18;15:93. doi: 10.1186/s12883-015-0342-0.

The PANGAEA study design - a prospective, multicenter, non-interventional,

long-term study on fingolimod for the treatment of multiple sclerosis in daily

practice.

Ziemssen T(1), Kern R(2), Cornelissen C(3).

Author information:

(1)Zentrum für klinische Neurowissenschaften, Klinik und Poliklinik für

Neurologie, Universitätsklinikum Carl Gustav Carus Dresden, Technische

Universität Dresden, Fetscherstr. 43, D-01307, Dresden, Germany.

tjalf.ziemssen@uniklinikum-dresden.de.

(2)Zentrum für klinische Neurowissenschaften, Klinik und Poliklinik für

Neurologie, Universitätsklinikum Carl Gustav Carus Dresden, Technische

Universität Dresden, Fetscherstr. 43, D-01307, Dresden, Germany.

Raimar.Kern@uniklinikum-dresden.de.

(3)Novartis Pharma GmbH, Roonstr. 25, D-90429, Nuernberg, Germany.

christian.cornelissen@novartis.com.

BACKGROUND: Fingolimod (Gilenya) is an oral medication for patients with highly

active relapsing-remitting Multiple Sclerosis (RRMS). Clinical trials and

post-marketing experience on more than 114,000 patients have established a

detailed safety profile. Total patient exposure now exceeds 195,000 patient-years

as stated in the last financial report (Dec 2014) of the Novartis Pharma AG,

Basel, Switzerland. However, less is known about the safety of long-term

fingolimod use in daily practice. Here, we describe the study design of PANGAEA

(Post-Authorization Non-interventional German sAfety of GilEnyA in RRMS

patients), a prospective, multicenter, non-interventional, long-term study to

collect safety, efficacy, and pharmacoeconomic data on RRMS patients treated with

fingolimod (0.5 mg/daily) under real-world conditions in Germany.

METHODS: PANGAEA is striving to assess a real-world safety and efficacy profile

of fingolimod, based on data from 4,000 RRMS patients, obtained during a 60-month

observational phase. A pharmacoeconomic sub-study of 800 RRMS patients further

collects patient-reported outcome measures of disability, quality of life,

compliance, treatment satisfaction, and usage of resources during a 24-month

observational phase. Descriptive statistical analyses of the safety set as well

as of stratified subgroups such as patients with concomitant diabetes mellitus

and pretreated patients (e.g., natalizumab) will be conducted.

DISCUSSION: PANGAEA seeks to confirm the current safety profile of fingolimod

obtained in phase I-III clinical trials. The study design presented here will

additionally provide guidance on the therapeutic use of fingolimod in clinical

practice and possibly assists physicians in making evidence-based decisions.

DOI: 10.1186/s12883-015-0342-0

PMCID: PMC4472406

PMID: 26084334 [Indexed for MEDLINE]

181. Curr Treat Options Neurol. 2015 Jun;17(6):354. doi: 10.1007/s11940-015-0354-5.

Excluded b/c no primary data (narrative review)

The transition from first-line to second-line therapy in multiple sclerosis.

Dörr J(1), Paul F.

Author information:

(1)NeuroCure Clinical Research Center and Clinical and Experimental Multiple

Sclerosis Research Center, Charité-Universitätsmedizin Berlin, Charitéplatz 1,

10117, Berlin, Germany, jan-markus.doerr@charite.de.

OPINION STATEMENT: Sufficient control of disease activity in multiple sclerosis

(MS) patients, particularly in the early phase of the disease, is crucial for the

prevention of an unfavorable outcome. While currently available disease modifying

drugs are generally clearly assigned as first-line or second-line treatment, no

universal guidelines exist that help in the real world setting to decide when and

how exactly a transition from first-line to second-line therapy should be

initiated. Furthermore, the concept of first and second-line therapies is

constantly evolving. In order to facilitate evidence-based decision making in

this common situation, we here summarize existing data on the optimization of

treatment when the first-line drug needs to be switched. Obviously, a switch of

treatment starts with an exploration of the motivation to switch, which usually

may be ascribed to either inadequate treatment response or tolerability, safety,

or adherence issues. In the latter situation, intra class switching, e.g., from

interferon (IFN) beta to glatiramer acetate (GA) or, in case of aversion against

injectables, from GA/IFN beta to one of the new orals dimethylfumarate or

teriflunomide can be a reasonable option. If treatment failure is the reason for

a switch, existing data suggest that escalation to a more powerful drug such as

natalizumab, fingolimod or even alemtuzumab is more appropriate. Of note, in some

drugs, different formal approvals apply in different countries. For example,

while fingolimod is approved as second-line therapy in the European Union, it can

be used as first-line drug in the United States and in Switzerland. The flip side

of these more powerful drugs might be a less favorable risk-benefit ratio. As

long as data are not yet sufficient to allow a direct comparison of efficacy

among second-line drugs, the treatment decision should be primarily based on the

individual situation and risk profile of the patient.

DOI: 10.1007/s11940-015-0354-5

PMID: 25912246

Excluded b/c no primary data (narrative review)

182. Patient Prefer Adherence. 2015 May 21;9:685-93. doi: 10.2147/PPA.S57354.

eCollection 2015.

Patient experience and practice trends in multiple sclerosis - clinical utility

of fingolimod.

Lee JM(1), Han MH(2).

Author information:

(1)Stanford Healthcare, Multiple Sclerosis Center, Stanford University School of

Medicine, Stanford University, Stanford, CA, USA.

(2)Stanford Healthcare, Multiple Sclerosis Center, Stanford University School of

Medicine, Stanford University, Stanford, CA, USA ; Department of Neurology and

Neurological Sciences, Stanford University School of Medicine, Stanford

University, Stanford, CA, USA.

Targeting sphingosine-1-phosphate pathway with orally available immune-modulatory

fingolimod (Gilenya™) therapy ameliorates relapsing-remitting multiple sclerosis

(RRMS) by decreasing relapse rate as shown in FREEDOMS and TRANSFORMS. Fingolimod

has also been shown to be superior to interferon-beta therapy as evidenced by

TRANSFORMS. Albeit multiple benefits in treatment of multiple sclerosis including

high efficacy and ease of administration, potential untoward effects such as

cardiotoxicity, risk of infection, and cancer exist, thus mandating careful

screening and frequent monitoring of patients undergoing treatment with

fingolimod. This review outlines mechanism of action, observations, side effects,

and practice guidelines on use of fingolimod in treatment of RRMS.

DOI: 10.2147/PPA.S57354

PMCID: PMC4446999

PMID: 26056436

183. J Neuroimmunol. 2015 May 15;282:118-22. doi: 10.1016/j.jneuroim.2015.03.022. Epub

Excluded b/c no real-world adherence/persistence

2015 Apr 7.

Severe disease reactivation in four patients with relapsing-remitting multiple

sclerosis after fingolimod cessation.

Berger B(1), Baumgartner A(2), Rauer S(2), Mader I(3), Luetzen N(3), Farenkopf

U(4), Stich O(2).

Author information:

(1)University Hospital Freiburg, Department of Neurology and Neurophysiology,

Breisacher Strasse 64, D-79106 Freiburg, Germany. Electronic address:

benjamin.berger@uniklinik-freiburg.de.

(2)University Hospital Freiburg, Department of Neurology and Neurophysiology,

Breisacher Strasse 64, D-79106 Freiburg, Germany.

(3)University Hospital Freiburg, Department of Neuroradiology, Breisacher Strasse

64, D-79106 Freiburg, Germany.

(4)Medical practice, Leutkirchstrasse 37, D-77723 Gengenbach, Germany.

OBJECTIVES: Fingolimod is a well-established, highly effective immunomodulatory

treatment for patients with relapsing-remitting multiple sclerosis (RRMS).

However, little is known about disease course after its discontinuation.

METHODS: This is a case series on four patients with RRMS who had a severe

reactivation after fingolimod discontinuation.

RESULTS: One patient had pretreatment with glatiramer acetate, interferon-β 1b

and interferon-β 1a and another with interferon-β 1a, intravenous immunoglobulins

and natalizumab whereas the other two were therapy naïve before fingolimod

initiation. Patients were treated with fingolimod for two, thirty, forty-five and

seventy-eight months, respectively. Fingolimod had to be discontinued because of

persisting lymphopenia, severe varicella zoster virus infection, subarachnoid

hemorrhage, and increased liver function enzymes, respectively. Between two to

four months after fingolimod cessation these patients had a severe relapse.

Cerebral magnetic resonance imaging (MRI) at this point revealed multiple new

lesions, partially with contrast ring enhancement. Partial recovery was achieved

after steroid pulse therapy followed by plasma exchange in two patients.

CONCLUSIONS: Despite the limited evidence from our case series on potential

disease reactivation exceeding pre-fingolimod activity in a subgroup of RRMS

patients, particularly patients with previously high disease activity should

undergo frequent clinical as well as radiological monitoring after fingolimod

discontinuation.

Copyright © 2015 Elsevier B.V. All rights reserved.

DOI: 10.1016/j.jneuroim.2015.03.022

PMID: 25903738 [Indexed for MEDLINE]

Excluded b/c no primary data (narrative review)

184. Mult Scler J Exp Transl Clin. 2015 May 12;1:2055217315585333. doi:

10.1177/2055217315585333. eCollection 2015 Jan-Dec.

Adherence to behavioural interventions in multiple sclerosis: Follow-up meeting

report (AD@MS-2).

Heesen C(1), Bruce J(2), Gearing R(3), Moss-Morris R, Weinmann J(4), Hamalainen

P(5), Motl R(6), Dalgas U(7), Kos D(8), Visioli F(9), Feys P(10), Solari A(11),

Finlayson M(12), Eliasson L(13), Matthews V(14), Bogossian A(4), Liethmann K(15),

Köpke S(16), Bissell P(17).

Author information:

(1)Institute of Neuroimmunology and Clinical MS Research (inims) and Department

of Neurology, UMC Hamburg Eppendorf, Germany.

(2)University of Missouri - Kansas City, Department of Psychology, USA.

(3)Columbia University, New York, USA.

(4)Health Psychology Section, Psychology Department, Institute of Psychiatry,

Psychology and Neuroscience, KCL, Guy's Hospital Campus London, UK.

(5)Director Masku Neurological Rehabilitation Centre, Finland.

(6)Department of Kinesiology and Community Health, University of Illinois at

Urbana-Champaign, USA.

(7)Department of Public Health - Sport Science, Aarhus, Denmark.

(8)Department of Rehabilitation Sciences, KU Leuven and Department of Health and

Social Care, AP University College Antwerp, Belgium.

(9)IMDEA-Food, Madrid, Spain; University of Padova, Italy.

(10)REVAL, Biomedical Research Institute (BIOMED), Hasselt University, Belgium.

(11)Neuroepidemiology Unit, Foundation Neurological Institute C. Besta, Milan,

Italy.

(12)School of Rehabilitation Therapy, Faculty of Health Sciences, Queen's

University, Kingston, Canada.

(13)Atlantis Healthcare, Summit, USA; Centre for Haematology, Imperial College

London, Hammersmith Hospital, UK.

(14)MS Trust, Letchworth, UK.

(15)Institute of Neuroimmunology and Multiple Sclerosis and Department of

Neurology, UMC Hamburg Eppendorf, Germany.

(16)Institute for Social Medicine and Epidemiology, University of Lübeck,

Germany.

(17)Section of Public Health, School of Health and Related Research (ScHARR), The

University of Sheffield, UK.

After an initial meeting in 2013 that reviewed adherence to disease modifying

therapy, the AD@MS group conducted a follow-up meeting in 2014 that examined

adherence to behavioural interventions in MS (e.g. physical activity, diet,

psychosocial interventions). Very few studies have studied adherence to

behavioural interventions in MS. Outcomes beyond six months are lacking, as well

as implementation work in the community. Psychological interventions need to

overcome stigma and other barriers to facilitate initiation and maintenance of

behaviour change. A focus group concentrated on physical activity and exercise as

one major behavioural intervention domain in MS. The discussion revealed that

patients are confronted with multiple challenges when attempting to regularly

engage in physical activity. Highlighted needs for future research included an

improved understanding of patients' and health experts' knowledge and attitudes

towards physical activity as well as a need for longitudinal research that

investigates exercise persistence.

DOI: 10.1177/2055217315585333

PMCID: PMC5433389

PMID: 28607693

Excluded b/c no primary data (narrative review)

185. Ther Clin Risk Manag. 2015 May 8;11:759-66. doi: 10.2147/TCRM.S69123. eCollection

2015.

Profile of PEGylated interferon beta in the treatment of relapsing-remitting

multiple sclerosis.

Cocco E(1), Marrosu MG(1).

Author information:

(1)Multiple Sclerosis Center, Department of Public Health, Clinical and Molecular

Medicine, University of Cagliari, Cagliari, Italy.

Several treatments are currently available for relapsing-remitting multiple

sclerosis. Among them, interferon (IFN) beta remains a valid treatment approach

because of its good benefit/risk profile. Due to the need for frequent

administration (weekly, at a minimum), the use of IFN beta is limited by

uncomfortable side effects that could reduce adherence to and persistence with

the treatment. The use of subcutaneous polyethylene glycol (PEG)ylated interferon

beta-1a (PEG-IFN) has been proposed to offer a better combination of

pharmacokinetic and pharmacodynamic profiles and therapy-related side effects. A

125 μg dose of PEG-IFN given every 2 or 4 weeks was tested in two Phase I studies

and shown to be as safe and efficient as IFN beta-1a but with a longer half-life.

A Phase III trial (ADVANCE) comparing 125 μg of PEG-IFN given every 2 or 4 weeks

with placebo in 1,512 patients with relapsing-remitting multiple sclerosis showed

significant reductions in both the annualized relapse rate (ARR) and the

occurrence of new or newly enlarged T2 brain lesions in both experimental groups

versus placebo after the first year. Moreover, 38% fewer patients showed

progression of disability (P=0.04) in the PEG-IFN groups. During the second year,

the ARR was further reduced in the PEG-IFN 2-week treatment group (0.230 at 1

year versus 0.178 at 2 years) and was maintained in the 4-week treatment group.

Patients who received immediate PEG-IFN treatment showed improved clinical

efficacy (ARR, risk of relapse, 12-week disability progression) and magnetic

resonance imaging parameters (new T2 and newly enlarging lesions,

gadolinium-positive lesions) compared with those with delayed treatment. The

effects were more evident with the 2-week dose for all endpoints considered.

Furthermore, PEG-IFN was well tolerated, and no new safety concerns arose. In

conclusion, PEG-IFN has good efficacy and a good safety profile. The available

data support the use of PEG-IFN as a suitable therapeutic option in patients with

relapsing-remitting multiple sclerosis.

DOI: 10.2147/TCRM.S69123

PMCID: PMC4431469

PMID: 26056458

186. Neurologia. 2015 May;30(4):214-22. doi: 10.1016/j.nrl.2013.12.008. Epub 2014 Jan

Excluded b/c no oral DMD results

28.

Therapeutic compliance of first line disease-modifying therapies in patients with

multiple sclerosis. COMPLIANCE Study.

[Article in English, Spanish]

Saiz A(1), Mora S(2), Blanco J(2); en representación de los investigadores del

estudio COMPLIANCE.

Collaborators: Marzo ME, Lacruz F, Peña J, Fernández D, Oterino A, Llaneza MÁ,

Pego R, del Real MÁ, Ginestal R, Casanova LI, Rubio L, Escartín A, Massons J,

Blanco Y, Barrero F, Foronda J, Cancho ED, Fernández R, Carod FJ, García MR,

Flores JM, Villaverde R, López JM, Meca J, Araña VM, Croissier C, Meca V, Cacho

J, Bowakim W, Miralles A, Plaza JF, Romero B.

Author information:

(1)Servicio de Neurología, Hospital Clínic i provincial de Barcelona, Universitat

de Barcelona e Institut d́Investigació August Pi i Sunyer (IDIBAPS), Barcelona,

España. Electronic address: asaiz@clinic.ub.es.

(2)Departamento Médico, Novartis Farmacéutica S.A., Barcelona, España.

INTRODUCTION: Non-adherence to disease-modifying therapies (DMTs) in multiple

sclerosis may be associated with reduced efficacy. We assessed compliance, the

reasons for non-compliance, treatment satisfaction, and quality of life (QoL) of

patients treated with first-line therapies.

METHODS: A cross-sectional, multicenter study was conducted that included

relapsing multiple sclerosis patients. Compliance in the past month was assessed

using Morisky-Green test. Seasonal compliance and reasons for non-compliance were

assessed by an ad-hoc questionnaire. Treatment satisfaction and QoL were

evaluated by means of TSQM and PRIMUS questionnaires.

RESULTS: A total of 220 patients were evaluated (91% relapsing-remitting); the

mean age was 39.1 years, 70% were female, and the average time under treatment

was 5.4 years. Subcutaneous interferon (IFN) β-1b was used in 23% of the

patients, intramuscular IFN β-1a in 21%, subcutaneous IFN β-1a in 37%, and with

glatiramer acetate in 19%. The overall compliance was 75%, with no significant

differences related to the therapy, and 81% did not report any seasonal

variation. Compliant patients had significantly lower disability scores and time

of diagnosis, and greater satisfaction with treatment and its effectiveness.

Discomfort and flu-like symptoms were the most frequent reasons for

non-compliance. The satisfaction and QoL were associated with less disability and

number of therapeutic switches.

CONCLUSIONS: The rate of compliance, satisfaction and QoL in multiple sclerosis

patients under DMTs is high, especially for those newly diagnosed, less disabled,

and with fewer therapeutic switches. Discomfort and flu-like symptoms associated

with injected therapies significantly affect adherence.

Copyright © 2013 Sociedad Española de Neurología. Published by Elsevier España,

S.L.U. All rights reserved.

DOI: 10.1016/j.nrl.2013.12.008

PMID: 24484756 [Indexed for MEDLINE]

Excluded b/c no primary data (meta-analysis)

187. Cochrane Database Syst Rev. 2015 Apr 22;(4):CD011076. doi:

10.1002/14651858.CD011076.pub2.

Dimethyl fumarate for multiple sclerosis.

Xu Z(1), Zhang F, Sun F, Gu K, Dong S, He D.

Author information:

(1)Department of Neurology, Affiliated Hospital of Guiyang Medical College, No.

28, Gui Yi Street, Guiyang, Guizhou Procince, China, 550004.

BACKGROUND: Multiple sclerosis (MS) often leads to severe neurological disability

and a serious decline in quality of life. The ideal target of disease-modifying

therapy for MS is to prevent disability worsening and improve quality of life.

Dimethyl fumarate is considered to have an immunomodulatory activity and

neuroprotective effect. It has been approved by the U.S. Food and Drug

Administration (FDA) and the European Medicines Agency as a first-line therapy

for adult patients with relapsing-remitting MS (RMSS).

OBJECTIVES: To assess the benefit and safety of dimethyl fumarate as monotherapy

or combination therapy versus placebo or other approved disease-modifying drugs

(interferon beta, glatiramer acetate, natalizumab, mitoxantrone, fingolimod,

teriflunomide, alemtuzumab) for patients with MS.

SEARCH METHODS: The Trials Search Co-ordinator searched the Trials Specialised

Register of the Cochrane Multiple Sclerosis and Rare Diseases of the Central

Nervous System Group (4 June 2014). We checked reference lists of published

reviews and retrieved articles and searched reports (2004 to June 2014) from the

MS societies in Europe and America. We also communicated with investigators

participating in trials of dimethyl fumarate and the Biogen Idec Medical

Information.

SELECTION CRITERIA: We included randomised, controlled, parallel-group clinical

trials (RCTs) with a length of follow-up equal to or greater than one year

evaluating dimethyl fumarate, as monotherapy or combination therapy, versus

placebo or other approved disease-modifying drugs for patients with MS without

restrictions regarding dosage, administration frequency and duration of

treatment.

DATA COLLECTION AND ANALYSIS: We used the standard methodological procedures of

The Cochrane Collaboration. Two review authors independently assessed trial

quality and extracted data. Disagreements were discussed and resolved by

consensus among the review authors. We contacted the principal investigators of

included studies for additional data or confirmation of data.

MAIN RESULTS: Two RCTs were included, involving 2667 adult patients with RRMS to

evaluate the efficacy and safety of two dosages of dimethyl fumarate (240 mg

orally three times daily or twice daily) by direct comparison with placebo for

two years. Among them, a subsample of 1221 (45.8%) patients were selected to

participate in MRI evaluations by each study site with MRI capabilities itself.

No powered head-to-head study with an active treatment comparator has been found.

Meta-analyses showed that dimethyl fumarate both three times daily and twice

daily reduced the number of patients with a relapse (risk ratio (RR) 0.57, 95%

confidence interval (CI) 0.50 to 0.66, P < 0.00001 and 0.64, 95% CI 0.54 to 0.77,

P < 0.00001, respectively) or disability worsening (RR 0.70, 95% CI 0.57 to 0.87,

P = 0.0009 and 0.65, 95% CI 0.53 to 0.81, P = 0.0001, respectively) over two

years, compared to placebo. The treatment effects were decreased in the

likely-case scenario analyses taking the effect of dropouts into consideration.

Both dosages also reduced the annualised relapse rate. Data of active lesions on

MRI scans were not combined because there was a high risk of selection bias for

MRI outcomes and imprecision of MRI data in both studies, as well as an obvious

heterogeneity between the studies. In terms of safety profile, both dosages

increased the risk for adverse events and the risk for drug discontinuation due

to adverse events. The most common adverse events included flushing and

gastrointestinal events (upper abdominal pain, nausea and diarrhoea). Uncommon

adverse events included lymphopenia and leukopenia, but they were more likely to

happen with dimethyl fumarate than with placebo (high dosage: RR 5.25, 95% CI

2.20 to 12.51, P = 0.0002 and 5.23, 95% CI 2.47 to 11.07, P < 0.0001,

respectively; low dosage: RR 5.69, 95% CI 2.40 to 13.46, P < 0.0001 and 6.53, 95%

CI 3.13 to 13.64, P < 0.00001, respectively). Both studies had a high attrition

bias resulting from the unbalanced reasons for dropouts among groups. Quality of

evidence for relapse outcome was moderate, but for disability worsening was low.

AUTHORS' CONCLUSIONS: There is moderate-quality evidence to support that dimethyl

fumarate at a dose of 240 mg orally three times daily or twice daily reduces both

the number of patients with a relapse and the annualised relapse rate over two

years of treatment in comparison with placebo. However, the quality of the

evidence to support the benefit in reducing the number of patients with

disability worsening is low. There is no high-quality data available to evaluate

the benefit on MRI outcomes. The common adverse effects such as flushing and

gastrointestinal events are mild-to-moderate for most patients. Lymphopenia and

leukopenia are uncommon adverse events but significantly associated with dimethyl

fumarate. Both dosages of dimethyl fumarate have similar benefit and safety

profile, which supports the option of low-dose administration. New studies of

high quality and long-term follow-up are needed to evaluate the benefit of

dimethyl fumarate on prevention of disability worsening and to observe the

long-term adverse effects including progressive multifocal leukoencephalopathy.

DOI: 10.1002/14651858.CD011076.pub2

PMID: 25900414 [Indexed for MEDLINE]

188. PLoS One. 2015 Apr 13;10(4):e0123824. doi: 10.1371/journal.pone.0123824.

Excluded b/c no oral DMD results

eCollection 2015.

Long-term persistence with injectable therapy in relapsing-remitting multiple

sclerosis: an 18-year observational cohort study.

Zhornitsky S(1), Greenfield J(1), Koch MW(1), Patten SB(2), Harris C(1), Wall

W(1), Alikhani K(1), Burton J(1), Busche K(1), Costello F(1), Davenport JW(1),

Jarvis SE(1), Lavarato D(2), Parpal H(1), Patry DG(1), Yeung M(1), Metz LM(1).

Author information:

(1)Department of Clinical Neurosciences, Faculty of Medicine, University of

Calgary; Calgary, Canada; Hotchkiss Brain Institute, Faculty of Medicine,

University of Calgary, Calgary, Canada.

(2)Department of Psychiatry, Faculty of Medicine, University of Calgary, Calgary,

Canada; Hotchkiss Brain Institute, Faculty of Medicine, University of Calgary,

Calgary, Canada.

Disease modifying therapies (DMTs) reduce the frequency of relapses and

accumulation of disability in multiple sclerosis (MS). Long-term persistence with

treatment is important to optimize treatment benefit. This long-term, cohort

study was conducted at the Calgary MS Clinic. All consenting adults with

relapsing-remitting MS who started either glatiramer acetate (GA) or interferon-β

1a/1b (IFN-β) between January 1st, 1996 and July 1st, 2011 were included.

Follow-up continued to February 1st, 2014. Time-to-discontinuation of the initial

and subsequently-prescribed DMTs (switches) was analysed using Kaplan-Meier

survival analyses. Group differences were compared using log-rank tests and

multivariable Cox regression models. Analysis included 1471 participants; 906

were initially prescribed GA and 565 were initially prescribed IFN-β. Follow-up

information was available for 87%; 29 (2%) were lost to follow-up and 160 (11%)

moved from Southern Alberta while still using DMT. Median time-to-discontinuation

of all injectable DMTs was 11.1 years. Participants with greater disability at

treatment initiation, those who started treatment before age 30, and those who

started between 2006 and 2011 were more likely to discontinue use of all

injectable DMTs. Median time-to-discontinuation of the initial DMT was 8.6 years.

Those initially prescribed GA remained on treatment longer. Of 610 participants

who discontinued injectable DMT, 331 (54%) started an oral DMT, or a second-line

DMT, or resumed injectable DMT after 90 days. Persistence with injectable DMTs

was high in this long-term population-based study. Most participants who

discontinued injectable DMT did not remain untreated. Further research is

required to understand treatment outcomes and outcomes after stopping DMT.

DOI: 10.1371/journal.pone.0123824

PMCID: PMC4395027

PMID: 25867095 [Indexed for MEDLINE]

Excluded b/c no primary data (systematic review)

189. Decisional Dilemmas in Discontinuing Prolonged Disease-Modifying Treatment for

Multiple Sclerosis [Internet].

Butler M(1), Forte ML(1), Schwehr N(1), Carpenter A(1), Kane RL(1).

Rockville (MD): Agency for Healthcare Research and Quality (US); 2015 Apr. Report

No.: 15-EHC012-EF.

AHRQ Comparative Effectiveness Reviews.

Author information:

(1)Minnesota Evidence-based Practice Center

OBJECTIVE: We conducted a systematic review to examine the long-term consequences

of discontinuing disease-modifying treatment (DMT) for multiple sclerosis (MS) by

examining the long-term benefits and harms, and the reasons for discontinuing

treatment. We also examined the evidence for people's values, beliefs, and

preferences regarding discontinuing DMT.

DATA SOURCES: We searched Medline®, PsycInfo®, Scopus, and the Cochrane Clinical

Trials Registry through August 2014 plus reference lists of included studies and

recent systematic reviews.

METHODS: Two investigators screened abstracts and full texts of identified

references for eligibility. Eligible studies included studies of over 3 years

that examined Food and Drug Administration–approved DMTs compared with placebo,

other active DMT, or no DMT for adults with clinically isolated syndrome or MS in

outpatient settings for patient-centered outcomes. We excluded studies of

mitoxantrone, since it has a maximum lifetime dosage. Timing was relaxed for

women who were considering pregnancy or already pregnant or patients

discontinuing natalizumab due to risk factor changes. We extracted data, assessed

risk of bias of individual studies, and evaluated strength of the body of

evidence for each comparison and outcome. We also evaluated, using Technical

Brief methods, studies of any design that examined individuals' attitudes,

values, and preferences for discontinuing treatments and health states, or

factors and processes patients with MS and clinicians use in shared

decisionmaking.

RESULTS: We identified 27 unique studies with discontinuation information: 16 of

these contained complete information to allow full analysis of long-term benefits

and harms. Evidence was insufficient for long-term benefits of DMTs for secondary

progressive MS patients and most outcomes for relapsing-remitting MS (RRMS)

patients. Low-strength evidence suggests higher long-term all-cause survival for

treatment-naïve RRMS patients who did not delay starting interferon beta-1b by 2

years and used DMTs for a longer duration than for those who started later.

Low-strength evidence suggests that interferon did not change RRMS patients'

disability progression. Limited low-strength evidence suggests that long-term

harms do not differ from short-term harms. The majority of discontinuation tends

to occur within 2 to 3 years. Another 25 unique studies provided intrapersonal,

interpersonal, and shared decisionmaking information. No study directly asked why

people may be reluctant to discontinue when treatment no longer seems effective;

taken as a whole, the literature set provides some insight. The preferences

literature underscores the complexity of the topic and the processes underlying

decisionmaking.

CONCLUSIONS: MS patients and providers have little information to guide decisions

to discontinue DMT.

PMID: 25996027

190. BMC Pharmacol Toxicol. 2015 Apr 1;16:7. doi: 10.1186/s40360-015-0006-0.

Excluded b/c no real-world adherence/persistence

A Swiss real world best practice experience in three different clinical settings

of the 6 hour fingolimod first dose observation procedure.

Ramseier SP(1), Roth S(2), Czaplinski A(3).

Author information:

(1)Cantonal Hospital Aarau, Aarau, Switzerland. Simon.Ramseier@ksa.ch.

(2)Clinique de Carouge SA, Carouge, Switzerland. serge.roth@latour.ch.

(3)Neurocentre Bellevue, Theaterstrasse 8, Zurich, CH-8001, Switzerland.

mc@neurozentrumbellevue.ch.

BACKGROUND: The Swiss label of oral fingolimod (0.5 mg once daily) requires a

6-hour first dose observation (FDO) including an ECG prior to and 6 hours after

the first intake but in comparison to other countries such as Austria, Australia

and Canada there are no restrictions regarding the clinical settings of the FDO

procedure in Switzerland. We present here our real-world experience of the 6 hour

FDO procedure in three different clinical settings, following fingolimod

treatment initiation. This is the first report on the FDO of fingolimod in these

real-world clinical settings in Swiss patients with multiple sclerosis (MS).

METHODS: This was a retrospective, multi-clinic, observational study of 136

patients with relapsing-remitting multiple sclerosis. Summary statistics have

been used to present the data.

RESULTS: Only two patients (<1.5% [2/136]) experienced symptoms after the first

dose of fingolimod. Atrioventricular conduction abnormalities were reported in 3%

(4/136) of patients, which resolved spontaneously within 24 hours of treatment

initiation. During the average 6.8 months follow-up, 96% (131/136) of the

patients remained on therapy

CONCLUSIONS: These findings support the safety and feasibility of FDO and

tolerability of fingolimod in real-world clinical settings.

DOI: 10.1186/s40360-015-0006-0

PMCID: PMC4405834

PMID: 25889400 [Indexed for MEDLINE]

191. J Med Econ. 2015 Apr;18(4):295-302. doi: 10.3111/13696998.2014.991786. Epub 2014

Excluded b/c no real-world adherence/persistence

Dec 10.

Cost-effectiveness of natalizumab vs fingolimod for the treatment of

relapsing-remitting multiple sclerosis: analyses in Sweden.

O'Day K(1), Meyer K, Stafkey-Mailey D, Watson C.

Author information:

(1)Xcenda, Global Health Economics & Outcomes Research , Palm Harbor, FL , USA.

OBJECTIVE: To assess the cost-effectiveness of natalizumab vs fingolimod over 2

years in relapsing-remitting multiple sclerosis (RRMS) patients and patients with

rapidly evolving severe disease in Sweden.

METHODS: A decision analytic model was developed to estimate the incremental cost

per relapse avoided of natalizumab and fingolimod from the perspective of the

Swedish healthcare system. Modeled 2-year costs in Swedish kronor of treating

RRMS patients included drug acquisition costs, administration and monitoring

costs, and costs of treating MS relapses. Effectiveness was measured in terms of

MS relapses avoided using data from the AFFIRM and FREEDOMS trials for all

patients with RRMS and from post-hoc sub-group analyses for patients with rapidly

evolving severe disease. Probabilistic sensitivity analyses were conducted to

assess uncertainty.

RESULTS: The analysis showed that, in all patients with MS, treatment with

fingolimod costs less (440,463 Kr vs 444,324 Kr), but treatment with natalizumab

results in more relapses avoided (0.74 vs 0.59), resulting in an incremental

cost-effectiveness ratio (ICER) of 25,448 Kr per relapse avoided. In patients

with rapidly evolving severe disease, natalizumab dominated fingolimod. Results

of the sensitivity analysis demonstrate the robustness of the model results. At a

willingness-to-pay (WTP) threshold of 500,000 Kr per relapse avoided, natalizumab

is cost-effective in >80% of simulations in both patient populations.

LIMITATIONS: Limitations include absence of data from direct head-to-head studies

comparing natalizumab and fingolimod, use of relapse rate reduction rather than

sustained disability progression as the primary model outcome, assumption of 100%

adherence to MS treatment, and exclusion of adverse event costs in the model.

CONCLUSIONS: Natalizumab remains a cost-effective treatment option for patients

with MS in Sweden. In the RRMS patient population, the incremental cost per

relapse avoided is well below a 500,000 Kr WTP threshold per relapse avoided. In

the rapidly evolving severe disease patient population, natalizumab dominates

fingolimod.

DOI: 10.3111/13696998.2014.991786

PMID: 25422991 [Indexed for MEDLINE]

192. JAMA Neurol. 2015 Apr;72(4):405-13. doi: 10.1001/jamaneurol.2014.4147.

Comparison of switch to fingolimod or interferon beta/glatiramer acetate in

active multiple sclerosis.

He A(1), Spelman T(2), Jokubaitis V(2), Havrdova E(3), Horakova D(3), Trojano

M(4), Lugaresi A(5), Izquierdo G(6), Grammond P(7), Duquette P(8), Girard M(8),

Pucci E(9), Iuliano G(10), Alroughani R(11), Oreja-Guevara C(12),

Fernandez-Bolaños R(13), Grand'Maison F(14), Sola P(15), Spitaleri D(16),

Granella F(17), Terzi M(18), Lechner-Scott J(19), Van Pesch V(20), Hupperts

R(21), Sánchez-Menoyo JL(22), Hodgkinson S(23), Rozsa C(24), Verheul F(25),

Butzkueven H(26), Kalincik T(27); MSBase Study Group.

Collaborators: Giuliani G, Cartechini E, Rio ME, Zwanikken C, Flechter S, Kasa K,

Amato MP, Shaygannejad V, Bergamaschi R, Slee M, Boz C, Moore F, Barnett M, Vucic

S, McCombe P, Cabrera-Gomez JA, Petersen T, Ramo C, Roullet E, Den

Braber-Moerland L, Paolicelli D, Iaffaldano P, Direnzo V, D'Onghia M, Piroska I,

Erdelyi T, Iljicsov A, Kovacs K, Sas A, Skibina O, Haartsen J, van Munster E,

Fiol M, Correale J, Ysrraelit C, Herbert J, Kister I, Sirbu CA, Shaw C, Vetere S,

Petkovska-Boskova T, Singhal B, Bacile EA, Arruda WO, Roger E, Despault P,

Marriott M, Van der Walt A, King J, Byron J, Morgan L, Hinson E, De Luca G, Di

Tommaso V, Travaglini D, Pietrolongo E, di Ioia M, Farina D, Mancinelli L, Rojas

JI, Patrucco L, Williams D, Dark L, Savino A, Chapman J, Cristiano E, Saladino

ML, Deri N, Gray O, Hughes S, Paine M, Vella N, Mechati S, Corageoud M, Bulla A.

Author information:

(1)Department of Neurology, Royal Melbourne Hospital, Melbourne, Australia.

(2)Department of Medicine, University of Melbourne, Melbourne, Australia.

(3)Department of Neurology and Center of Clinical Neuroscience, General

University Hospital, Prague, Czech Republic4Department of Neurology and Center

for Clinical Neuroscience, Charles University, Prague, Czech Republic.

(4)Department of Basic Medical Sciences, Neuroscience, and Sense Organs,

University of Bari, Bari, Italy.

(5)MS Center, Department of Neuroscience, Imaging, and Clinical Sciences,

University G. d'Annunzio, Chienti, Italy.

(6)Department of Neurology, Hospital Universitario Virgen Macarena, Sevilla,

Spain.

(7)Department of Neurology, Hotel-Dieu de Lévis, Lévis, Quebec, Canada.

(8)Department of Neurology, Hôpital Notre Dame, Montreal, Quebec, Canada.

(9)Neurology Unit, Azienda Sanitaria Unica Regionale Marche, Macerata, Italy.

(10)Department of Neurology, Ospedali Riuniti di Salerno, Salerno, Italy.

(11)Department of Neurology, Amiri Hospital, Kuwait City, Kuwait.

(12)Multiple Sclerosis Unit, University Hospital San Carlos, Madrid, Spain.

(13)Department of Neurology, Hospital Universitario Virgen de Valme, Seville,

Spain.

(14)Neuro Rive-Sud, Hôpital Charles LeMoyne, Quebec City, Quebec, Canada.

(15)Department of Neurology, Nuovo Ospedale Civile San Agostino, Modena, Italy.

(16)Department of Neurology, Azienda Ospedaliera di Rilievo Nazionale, San

Giuseppe Moscati, Avellino, Italy.

(17)Institute of Neurology, University of Parma, Parma, Italy.

(18)Medical Faculty, Department of Neurology, Ondokuz Mayis University, Samsun,

Turkey.

(19)Department of Medicine, John Hunter Hospital, Newcastle, Australia21Hunter

Medical Research Institute, University of Newcastle, Newcastle, Australia.

(20)Department of Neurology, Cliniques Universitaires Saint-Luc, Brussels,

Belgium.

(21)Department of Neurology, Orbis Medical Center, Sittard, the Netherlands.

(22)Department of Neurology, Hospital de Galdakao-Usansolo, Galdakao, Spain.

(23)Department of Nephrology, Liverpool Hospital, Liverpool,

Australia26Department of Neurology, Liverpool Hospital, Liverpool, Australia.

(24)Department of Neurology, Jahn Ferenc Teaching Hospital, Budapest, Hungary.

(25)Neurology Unit, Groen Hart Ziekenhuis, Gouda, the Netherlands.

(26)Department of Neurology, Royal Melbourne Hospital, Melbourne,

Australia2Department of Medicine, University of Melbourne, Melbourne,

Australia29Department of Neurology, Box Hill Hospital, Monash University,

Melbourne, Australia.

(27)Department of Neurology, Royal Melbourne Hospital, Melbourne,

Australia2Department of Medicine, University of Melbourne, Melbourne, Australia.

Erratum in

JAMA Neurol. 2015 Aug;72(8):950.

Comment in

JAMA Neurol. 2015 Apr;72(4):387-9.

Nat Rev Neurol. 2015 Jun;11(6):316-7.

IMPORTANCE: After multiple sclerosis (MS) relapse while a patient is receiving an

injectable disease-modifying drug, many physicians advocate therapy switch, but

the relative effectiveness of different switch decisions is often uncertain.

OBJECTIVE: To compare the effect of the oral immunomodulator fingolimod with that

of all injectable immunomodulators (interferons or glatiramer acetate) on relapse

rate, disability, and treatment persistence in patients with active MS.

DESIGN, SETTING, AND PARTICIPANTS: Matched retrospective analysis of data

collected prospectively from MSBase, an international, observational cohort

study. The MSBase cohort represents a population of patients with MS monitored at

large MS centers. The analyzed data were collected between July 1996 and April

2014. Participants included patients with relapsing-remitting MS who were

switching therapy to fingolimod or injectable immunomodulators up to 12 months

after on-treatment clinical disease activity (relapse or progression of

disability), matched on demographic and clinical variables. Median follow-up

duration was 13.1 months (range, 3-80). Indication and attrition bias were

controlled with propensity score matching and pairwise censoring, respectively.

Head-to-head analyses of relapse and disability outcomes used paired, weighted,

negative binomial models or frailty proportional hazards models adjusted for

magnetic resonance imaging variables. Sensitivity analyses were conducted.

EXPOSURES: Patients had received fingolimod, interferon beta, or glatiramer

acetate for a minimum of 3 months following a switch of immunomodulatory therapy.

MAIN OUTCOMES AND MEASURES: Annualized relapse rate and proportion of

relapse-free patients, as well as the proportion of patients without sustained

disability progression.

RESULTS: Overall, 379 patients in the injectable group were matched to 148

patients in the fingolimod group. The fingolimod group had a lower mean

annualized relapse rate (0.31 vs 0.42; 95% CI, 0.02-0.19; P=.009), lower hazard

of first on-treatment relapse (hazard ratio [HR], 0.74; 95% CI, 0.56-0.98;

P=.04), lower hazard of disability progression (HR, 0.53; 95% CI, 0.31-0.91;

P=.02), higher rate of disability regression (HR, 2.0; 95% CI, 1.2-3.3; P=.005),

and lower hazard of treatment discontinuation (HR, 0.55; P=.04) compared with the

injectable group.

CONCLUSIONS AND RELEVANCE: Switching from injectable immunomodulators to

fingolimod is associated with fewer relapses, more favorable disability outcomes,

and greater treatment persistence compared with switching to another injectable

preparation following on-treatment activity of MS.

DOI: 10.1001/jamaneurol.2014.4147

PMID: 25665031 [Indexed for MEDLINE]

193. Interact J Med Res. 2015 Mar 5;4(1):e6. doi: 10.2196/ijmr.3776.

Excluded b/c no real-world adherence/persistence

Preferred features of oral treatments and predictors of non-adherence: two

web-based choice experiments in multiple sclerosis patients.

Wicks P(1), Brandes D, Park J, Liakhovitski D, Koudinova T, Sasane R.

Author information:

(1)PatientsLikeMe, Cambridge, MA, United States. pwicks@patientslikeme.com.

BACKGROUND: Oral disease modifying therapies (DMTs) for multiple sclerosis (MS)

differ in efficacy, tolerability, and safety.

OBJECTIVE: We sought to understand how these attributes impact patient preference

and predicted DMT non-adherence among oral-naïve MS patients.

METHODS: Adult MS patients from the "PatientsLikeMe" Web-based health

data-sharing platform completed a discrete choice exercise where they were asked

to express their preference for one of three hypothetical oral DMTs, each with a

certain combination of levels of tested attributes. Another Web-based exercise

tested a number of possible drivers of non-adherence, mainly side effects. Data

from an MS clinic were used to adjust for sample bias. Respondents' preferences

were analyzed using Hierarchical Bayesian estimation.

RESULTS: A total of 319 patients completed all questions. Most respondents were

female (77.7%, 248/319) with mean age 48 years (SD 10). Liver toxicity was the

attribute that emerged as the most important driver of patient preference (25.8%,

relative importance out of 100%), followed by severe side effects (15.3%), delay

to disability progression (10.7%), and common side effects (10.4%). The most

important drivers of predicted non-adherence were frequency of daily dosing

(17.4% out of 100%), hair thinning (14.8%), use during pregnancy (14.1%), severe

side effects (13.8%), and diarrhea (13.0%).

CONCLUSIONS: Understanding the important concerns expressed by patients may help

health care providers to understand and educate their patients more completely

about these concerns. This knowledge may therefore improve both choices of

appropriate therapy and adherence to therapy over time.

DOI: 10.2196/ijmr.3776

PMCID: PMC4376178

PMID: 25749630

194. Curr Treat Options Neurol. 2015 Mar;17(3):336. doi: 10.1007/s11940-014-0336-z.

Excluded b/c no primary data (narrative review)

Treatment of pediatric multiple sclerosis.

Narula S(1), Hopkins SE, Banwell B.

Author information:

(1)Division of Neurology, Children's Hospital of Philadelphia, Perelman School of

Medicine at the University of Pennsylvania, 3501 Civic Center Boulevard, Colket

Translational Research Building, 10th floor, Philadelphia, PA, 19104, USA,

narulas@email.chop.edu.

OPINION STATEMENT: The past 10 years have borne witness to increased recognition

and diagnosis of pediatric multiple sclerosis (MS). Additionally, during this

time period, the number of treatment options available for MS patients has

increased significantly, as has the number of studies evaluating the use of these

therapies in children. Though the U.S. Food and Drug Administration has not

formally approved any of these therapies for use in pediatric MS, a number of

injectable, oral, and intravenous treatments are currently being used off-label

in these children. Disease modifying therapy should be initiated promptly

following a diagnosis of MS. The patient and family should be engaged in the

choice of therapy as this is likely to promote adherence. First-line options

include any of the injectable therapies (glatiramer acetate, interferon beta),

which have roughly similar efficacy (approximately 30 % reduction of clinical

relapses). If a patient has breakthrough disease or persistent, unmanageable side

effects, transition to a different first-line therapy or escalation to a

second-line therapy, such as natalizumab, should be considered. Though the

efficacy of second-line agents is higher, the potential risk of serious adverse

effects also increases. New therapies, including oral agents, are now being

rigorously studied with pediatric clinical trials and may provide safe

alternatives for patients that are either unresponsive or intolerant to currently

available medications. When necessary, acute exacerbations can be treated with

corticosteroids. Intravenous methylprednisolone at a dosage of 30 mg/kg/day

(maximum dose 1000 mg/day) for 3-5 days is recommended with severe attacks. If

patients are unresponsive to corticosteroids, treatment with either intravenous

immunoglobulin or plasma exchange may be required. Fatigue, spasticity, and pain

can also occur in pediatric patients with MS. Medications are needed if symptoms

are severe and impact quality of life.

DOI: 10.1007/s11940-014-0336-z

PMID: 25680425

195. Eur J Neurol. 2015 Mar;22(3):585-7. doi: 10.1111/ene.12487. Epub 2014 Jul 3.

Excluded b/c no real-world adherence/persistence

No impact of current therapeutic strategies on disease reactivation after

natalizumab discontinuation: a comparative analysis of different approaches

during the first year of natalizumab discontinuation.

Capobianco M(1), di Sapio A, Malentacchi M, Malucchi S, Matta M, Sperli F,

Bertolotto A.

Author information:

(1)Regional Multiple Sclerosis Centre, San Luigi Gonzaga Hospital, Orbassano,

Italy.

BACKGROUND AND PURPOSE: Natalizumab discontinuation induces the recurrence of

multiple sclerosis disease activity: currently no therapeutic approach has been

found able to abolish disease reactivation.

METHODS: The recurrence of disease activity after natalizumab discontinuation was

retrospectively evaluated in 79 patients who had been treated with

immunomodulating agents, other first-line therapies, fingolimod or not treated.

RESULTS: No differences have been found in clinical or magnetic resonance imaging

recurrence of disease activity amongst the groups. Interestingly, no disease

reactivation was observed only in one patient treated for 6 months with monthly

pulses of cyclophosphamide.

CONCLUSION: Disease modifying treatment or 'no treatment' is unable to abolish

disease activity reactivation after natalizumab discontinuation.

© 2014 The Author(s) European Journal of Neurology © 2014 EAN.

DOI: 10.1111/ene.12487

PMID: 24995482 [Indexed for MEDLINE]

196. Int J MS Care. 2015 Mar-Apr;17(2):57-64. doi: 10.7224/1537-2073.2013-051.

Excluded b/c no real-world adherence/persistence

Walgreens connected care: impact of managed therapy on adherence to medications

used to treat multiple sclerosis and related comorbid conditions.

DuChane J(1), Clark B(1), Staskon F(1), Miller R(1), Love K(1), Duncan I(1).

Author information:

(1)Health Services and Outcomes Research, Walgreen Co, Deerfield, IL, USA (JD,

BC, FS, ID); and Specialty Solutions Group, Walgreen Co, Pittsburgh, PA, USA (RM,

KL).

BACKGROUND: The Walgreens Connected Care Multiple Sclerosis (CCMS) treatment

management program provides enhanced levels of monitoring, oversight, and care

for patients taking MS disease-modifying agents. This study compared rates of

adherence to MS medications for patients participating in the CCMS program for at

least 6 months with those for patients participating for less than 6 months. For

a subsample of patients, we also examined the relationship between adherence and

the presence of fatigue or depression.

METHODS: This was a retrospective study of patients new to the CCMS program and

followed up for 1 year of participation. Adherence to MS medications was measured

as the proportion of days covered, with propensity scores used to match the CCMS

intervention group to the less-managed comparison group. The impact of program

participation on the relationship between depression or fatigue and adherence

over time was a separate analysis.

RESULTS: Mean proportion of days covered rates improved significantly in the

group managed for at least 6 months compared with those who were less managed.

Positive screenings for fatigue and depression reduced adherence in the

less-managed group but not in patients with longer participation in the program.

CONCLUSIONS: Overall, the CCMS program significantly increased adherence to MS

medications. This improved adherence was not negatively impacted by positive

screenings for fatigue and depression.

DOI: 10.7224/1537-2073.2013-051

PMCID: PMC4399768

PMID: 25892975

197. J Clin Neurosci. 2015 Mar;22(3):598-600. doi: 10.1016/j.jocn.2014.08.016. Epub

Excluded b/c no real-world adherence/persistence

2014 Dec 15.

Treatment of progressive multifocal leukoencephalopathy-immune reconstitution

inflammatory syndrome with intravenous immunoglobulin in a patient with multiple

sclerosis treated with fingolimod after discontinuation of natalizumab.

Calic Z(1), Cappelen-Smith C(2), Hodgkinson SJ(1), McDougall A(1), Cuganesan

R(3), Brew BJ(4).

Author information:

(1)Department of Neurology and Neurophysiology, Liverpool Hospital, Locked Bag

7103, Liverpool BC, NSW 1871, Australia; South Western Sydney Clinical School,

University of New South Wales, Sydney, NSW, Australia.

(2)Department of Neurology and Neurophysiology, Liverpool Hospital, Locked Bag

7103, Liverpool BC, NSW 1871, Australia; South Western Sydney Clinical School,

University of New South Wales, Sydney, NSW, Australia. Electronic address:

Cecilia.Cappelen-Smith@swsahs.nsw.gov.au.

(3)Department of Radiology, Liverpool Hospital, Liverpool BC, NSW, Australia.

(4)Department of Neurology, St Vincent's Hospital, Darlinghurst, NSW, Australia.

We report a case of progressive multifocal leukoencephalopathy-immune

reconstitution inflammatory syndrome in a multiple sclerosis (MS) patient 3.5

months after fingolimod commencement and 4.5 months after natalizumab (NTZ)

cessation. Three cerebrospinal fluid analyses were required before a definitive

diagnosis of progressive multifocal leukoencephalopathy was reached. Intravenous

immunoglobulin (IVIG) was subsequently given as the sole MS treatment along with

mirtazapine and mefloquine. There has been improvement and subsequent clinical

stabilization. The notable features are the difficult timing of fingolimod

commencement in the context of previous NTZ therapy, the role of repeated

cerebrospinal fluid John Cunningham virus analyses in progressive multifocal

leukoencephalopathy diagnosis, and the role of IVIG.

Crown Copyright © 2014. Published by Elsevier Ltd. All rights reserved.

DOI: 10.1016/j.jocn.2014.08.016

PMID: 25523125 [Indexed for MEDLINE]

198. J Manag Care Spec Pharm. 2015 Mar;21(3):210-8b.

Excluded b/c no real-world adherence/persistence

Increased relapse activity for multiple sclerosis natalizumab users who become

nonpersistent: a retrospective study.

McQueen RB(1), Livingston T, Vollmer T, Corboy J, Buckley B, Allen RR, Nair K,

Campbell JD.

Author information:

(1)University of Colorado Skaggs School of Pharmacy and Pharmaceutical Sciences,

Anschutz Medical Campus, Mail Stop C238, 12850 E. Montview Blvd., Aurora, CO

80045. Tel.: 303.709.9264; FAX: 303.724.0979. Robert.mcqueen@ucdenver.edu.

BACKGROUND: Natalizumab disease-modifying therapy for relapsing-remitting

multiple sclerosis (MS) is efficacious in randomized controlled trials. Few

studies have estimated the association between real-world natalizumab persistence

behavior and relapse-related outcomes.

OBJECTIVES: To (a) examine the impact of using natalizumab consistently (i.e.,

persistent) on relapse-related outcomes as compared with transitioning to

inconsistent natalizumab use (i.e., nonpersistent) and (b) examine the impact of

other treatment patterns on relapse-related outcomes for those who initiated

natalizumab.

METHODS: Using the IMS PharMetrics Plus claims database (years 2006-2012), we

identified MS subjects who initiated natalizumab (no natalizumab claims in year

prior) and had at least 2 years of follow-up. Persistence in annual follow-up

periods was defined as no 90-day or greater gap in natalizumab therapy. Relapse

was an MS-related hospitalization or outpatient visit with intravenous or oral

steroid burst claim within 7 days. Analyses compared observations based on

changes in natalizumab persistence and natalizumab nonpersistence status from 1

year to the next (e.g., transitioning from persistent to nonpersistent),

estimating differences in mean annual relapses and mean annual relapse-related

costs.

RESULTS: A total of 2,407 natalizumab initiators had at least 2 years of

follow-up, yielding 4,770 year-to-year natalizumab treatment patterns where each

subject contributed 1, 2, or 3 year-to-year treatment patterns. In the year

prior, 3,187 treatment patterns were persistent; 731 (22.9%) of these

transitioned to nonpersistence. The remaining 1,583 treatment patterns were

nonpersistent in the year prior; 132 (8.3%) of these transitioned to persistence.

Persistent to nonpersistent treatment patterns were associated with a mean

relapse-rate increase of 0.23 (95% CI = 0.12, 0.35), and a mean increase in

relapse-related costs of $1,346 (95% CI = $97, $2,595). Nonpersistent to

persistent treatment patterns were associated with a mean relapse-rate decrease

of -0.15 (95% CI = -0.32, 0.017) and a mean decrease in relapse-related costs of

-$1,369 (95% CI = -$2,761, $23).

CONCLUSIONS: Findings suggest that real-world persistent natalizumab users who

become nonpersistent have statistically significant increases in annual relapses

and relapse-related costs. Those who transition from nonpersistent to persistent

have nonsignificant reductions in relapses and their associated costs.

DOI: 10.18553/jmcp.2015.21.3.210

PMID: 25726030 [Indexed for MEDLINE]

Excluded b/c no primary data (systematic review)

199. Mult Scler. 2015 Mar;21(3):305-17. doi: 10.1177/1352458514564487. Epub 2015 Jan

12.

The incidence and prevalence of psychiatric disorders in multiple sclerosis: a

systematic review.

Marrie RA(1), Reingold S(2), Cohen J(3), Stuve O(4), Trojano M(5), Sorensen

PS(6), Cutter G(7), Reider N(8).

Author information:

(1)Department of Internal Medicine, University of Manitoba, Canada/Department of

Community Health Sciences, University of Manitoba, Canada rmarrie@hsc.mb.ca.

(2)Scientific and Clinical Review Associates, LLC, USA.

(3)Mellen Center for MS Treatment and Research, Cleveland Clinic, USA.

(4)Department of Neurology and Neurotherapeutics, University of Texas

Southwestern, USA.

(5)Department of Basic Medical Sciences, Neurosciences and Sense Organs,

University of Bari, Italy.

(6)Department of Neurology, Copenhagen University Hospital Rigshospitalet,

Denmark.

(7)Department of Biostatistics, University of Alabama at Birmingham, USA.

(8)Department of Community Health Sciences, University of Manitoba, Canada.

BACKGROUND: Psychiatric comorbidity is associated with lower quality of life,

more fatigue, and reduced adherence to disease-modifying therapy in multiple

sclerosis (MS).

OBJECTIVES: The objectives of this review are to estimate the incidence and

prevalence of selected comorbid psychiatric disorders in MS and evaluate the

quality of included studies.

METHODS: We searched the PubMed, PsychInfo, SCOPUS, and Web of Knowledge

databases and reference lists of retrieved articles. Abstracts were screened for

relevance by two independent reviewers, followed by full-text review. Data were

abstracted by one reviewer, and verified by a second reviewer. Study quality was

evaluated using a standardized tool. For population-based studies we assessed

heterogeneity quantitatively using the I² statistic, and conducted meta-analyses.

RESULTS: We included 118 studies in this review. Among population-based studies,

the prevalence of anxiety was 21.9% (95% CI: 8.76%-35.0%), while it was 14.8% for

alcohol abuse, 5.83% for bipolar disorder, 23.7% (95% CI: 17.4%-30.0%) for

depression, 2.5% for substance abuse, and 4.3% (95% CI: 0%-10.3%) for psychosis.

CONCLUSION: This review confirms that psychiatric comorbidity, particularly

depression and anxiety, is common in MS. However, the incidence of psychiatric

comorbidity remains understudied. Future comparisons across studies would be

enhanced by developing a consistent approach to measuring psychiatric

comorbidity, and reporting of age-, sex-, and ethnicity-specific estimates.

© The Author(s), 2015.

DOI: 10.1177/1352458514564487

PMCID: PMC4429164

PMID: 25583845 [Indexed for MEDLINE]

200. J Control Release. 2015 Feb 10;199:37-44. doi: 10.1016/j.jconrel.2014.12.001.

Excluded b/c no real-world adherence/persistence

Epub 2014 Dec 4.

Buccal mucosal delivery of a potent peptide leads to therapeutically-relevant

plasma concentrations for the treatment of autoimmune diseases.

Jin L(1), Boyd BJ(1), White PJ(2), Pennington MW(3), Norton RS(4), Nicolazzo

JA(5).

Author information:

(1)Drug Delivery, Disposition and Dynamics, Monash Institute of Pharmaceutical

Sciences, Monash University, Parkville, Victoria, Australia.

(2)Drug Discovery Biology, Monash Institute of Pharmaceutical Sciences, Monash

University, Parkville, Victoria, Australia.

(3)Peptides International, Louisville, KY, USA.

(4)Medicinal Chemistry, Monash Institute of Pharmaceutical Sciences, Monash

University, Parkville, Victoria, Australia.

(5)Drug Delivery, Disposition and Dynamics, Monash Institute of Pharmaceutical

Sciences, Monash University, Parkville, Victoria, Australia. Electronic address:

joseph.nicolazzo@monash.edu.

Stichodactyla helianthus neurotoxin (ShK) is an immunomodulatory peptide

currently under development for the treatment of autoimmune diseases, including

multiple sclerosis and rheumatoid arthritis by parenteral administration. To

overcome the low patient compliance of conventional self-injections, we have

investigated the potential of the buccal mucosa as an alternative delivery route

for ShK both in vitro and in vivo. After application of fluorescent 5-Fam-ShK to

untreated porcine buccal mucosa, there was no detectable peptide in the receptor

chamber using an in vitro Ussing chamber model. However, the addition of the

surfactants sodium taurodeoxycholate hydrate or cetrimide, and formulation of ShK

in a chitosan mucoadhesive gel, led to 0.05-0.13% and 1.1% of the applied dose,

respectively, appearing in the receptor chamber over 5h. Moreover, confocal

microscopic studies demonstrated significantly enhanced buccal mucosal retention

of the peptide (measured by mucosal fluorescence associated with 5-Fam-ShK) when

enhancement strategies were employed. Administration of 5-Fam-ShK to mice

(10mg/kg in a mucoadhesive chitosan-based gel (3%, w/v) with or without cetrimide

(5%, w/w)) resulted in average plasma concentrations of 2.6-16.2nM between 2 and

6h, which were substantially higher than the pM concentrations required for

therapeutic activity. This study demonstrated that the buccal mucosa is a

promising administration route for the systemic delivery of ShK for the treatment

of autoimmune diseases.

Copyright © 2014 Elsevier B.V. All rights reserved.

DOI: 10.1016/j.jconrel.2014.12.001

PMID: 25482338 [Indexed for MEDLINE]

Excluded b/c no primary data (narrative review)

201. Patient Prefer Adherence. 2015 Feb 9;9:265-74. doi: 10.2147/PPA.S61651.

eCollection 2015.

Teriflunomide for the treatment of relapsing-remitting multiple sclerosis:

patient preference and adherence.

Bayas A(1), Mäurer M(2).

Author information:

(1)Department of Neurology, Klinikum Augsburg, Augsburg, Germany.

(2)Department of Neurology, Caritas Krankenhaus Bad Mergentheim, Bad Mergentheim,

Germany.

Multiple sclerosis (MS), a chronic demyelinating neuroinflammatory disease of the

central nervous system, is the most common neurological disorder leading to

disability in young adulthood. In the last 2 decades, numerous treatments for

relapsing-remitting MS have been approved with eleven treatment options available

worldwide. One of the determinants in treatment selection is disease activity in

the individual patient. However, patient preferences play an increasingly major

role in treatment decision making. With teriflunomide, a reversible inhibitor of

the enzyme dihydroorotate dehydrogenase, a new oral therapeutic option, given

once daily, has been approved within the last 2 years by the regulatory agencies.

The current review focuses on characteristics of the drug relevant for patients'

preferences in the treatment decision process in the light of the available

medications. Perceiving and considering patients' preferences will have an effect

on treatment adherence, which is known to be often low in MS patients.

Teriflunomide-related adherence issues will also be discussed regarding mode of

application, dosing, and potential side effects.

DOI: 10.2147/PPA.S61651

PMCID: PMC4332317

PMID: 25709412

202. J Drug Assess. 2015 Feb 7;4(1):1-6. doi: 10.3109/21556660.2015.1010650.

Excluded b/c no oral DMD results

eCollection 2015.

Results of sustained long-term use of interferon beta-1a in a community-based

cohort of patients with relapsing multiple sclerosis.

Cohan S(1), Chen C(2), Baraban E(2), Stuchiner T(2), Grote L(2), Rodriguez M(2).

Author information:

(1)Providence Health and Services, Multiple Sclerosis Center, Portland, OR, USA.

(2)Providence Health and Services, Brain and Spine Institute, Portland, OR, USA.

BACKGROUND: Few studies have evaluated long-term efficacy of interferon beta-1a

in large community-based cohorts.

OBJECTIVE: Evaluate time to relapse, relapse rate, and disability progression in

patients treated with intramuscular interferon beta-1a.

METHODS: A retrospective review of medical records from 2000-2010 was performed.

Adult patients with relapsing-remitting MS or clinically isolated syndrome

treated with interferon beta-1a were included. Primary outcomes were time to

relapse, annualized relapse rate, and changes in Expanded Disability Status Scale

score. Other outcomes included factors associated with time to first relapse,

risk of having a relapse while receiving interferon beta-1a, and discontinuation

of therapy.

RESULTS: In total, 364 of 696 patients screened were enrolled, with a mean age of

51 ± 12.1 years, disease duration of 9.39 ± 7.02 years, and duration of therapy

of 4.03 ± 2.56 years. Mean time to first on-therapy relapse was 5.58 ± 0.26

years, annualized relapse rate was 0.30 ± 0.55 years, and mean increase in

sustained Expanded Disability Status Scale score was 0.018. Relapse risk was

associated with higher baseline Expanded Disability Status Scale score, age at

disease onset, and number of relapses in the 12 months prior to therapy

initiation.

CONCLUSIONS: This study demonstrates favorable clinical outcomes observed in a

large community-based cohort, and serves to emphasize the continued therapeutic

importance of interferon beta-1a, despite the development of newer agents with

greater convenience of use, but also more potential risk of serious morbidity.

DOI: 10.3109/21556660.2015.1010650

PMCID: PMC4937628

PMID: 27536455

203. Expert Opin Drug Metab Toxicol. 2015 Feb;11(2):295-306. doi:

Excluded b/c no primary data (narrative review)

10.1517/17425255.2015.993315. Epub 2014 Dec 16.

Pharmacology and clinical efficacy of dalfampridine for treating multiple

sclerosis.

Lugaresi A(1).

Author information:

(1)University "G. d'Annunzio" of Chieti-Pescara, Department of Neuroscience,

Imaging and Clinical Sciences , Via dei Vestini 31, Chieti, 66100 , Italy

lugaresi@unich.it.

INTRODUCTION: Multiple sclerosis (MS) is the most frequent cause of neurological

impairment and sustained disability in young adults. Currently approved

disease-modifying drugs do not directly ameliorate the most common symptoms, such

as walking impairment. Dalfampridine (DAL), currently approved in all forms of

MS, might represent an answer to unmet needs in the symptomatic treatment of MS.

AREAS COVERED: The main pharmacological and clinical properties and safety issues

of DAL, an extended-release formulation of 4-aminopyridine (4-AP), a

broad-spectrum voltage-dependent potassium channel blocker, are described.

Relevant publications were identified from a search of PubMed from 1966 to June

2014 (search terms 'dalfampridine OR fampridine OR 4-aminopyridine). DAL, 10 mg

twice daily, improves walking ability in approximately one-third and walking

speed in about 25% of patients, independently from disease course, compared with

placebo; it also improves leg strength. Treatment is generally well tolerated,

although there is a dose-dependent increased risk of seizures, especially with

dosages > 10 mg twice daily.

EXPERT OPINION: DAL represents an option in the symptomatic treatment of MS.

Improved ambulation can impact quality of life, motivation and adherence,

enhancing the successful management of MS. It has still to be established whether

this favorably impacts costs associated with MS.

DOI: 10.1517/17425255.2015.993315

PMID: 25510833 [Indexed for MEDLINE]

Excluded b/c no real-world adherence/persistence

204. Health Technol Assess. 2015 Feb;19(12):vii-viii, xxv-xxxi, 1-187. doi:

10.3310/hta19120.

The Cannabinoid Use in Progressive Inflammatory brain Disease (CUPID) trial: a

randomised double-blind placebo-controlled parallel-group multicentre trial and

economic evaluation of cannabinoids to slow progression in multiple sclerosis.

Ball S(1), Vickery J(2), Hobart J(2), Wright D(1), Green C(3), Shearer J(3), Nunn

A(4), Cano MG(1), MacManus D(5), Miller D(5), Mallik S(5), Zajicek J(2).

Author information:

(1)Centre for Biostatistics, Bioinformatics and Biomarkers, Plymouth University

Peninsula Schools of Medicine and Dentistry, Plymouth, UK.

(2)Peninsula Clinical Trials Unit, Plymouth University Peninsula Schools of

Medicine and Dentistry, Plymouth, UK.

(3)Health Economics Group, University of Exeter Medical School, Exeter, UK.

(4)Medical Research Council Clinical Trials Unit, London, UK.

(5)University College London's Institute of Neurology, London, UK.

Comment in

Evid Based Med. 2015 Aug;20(4):124.

BACKGROUND: The Cannabinoid Use in Progressive Inflammatory brain Disease (CUPID)

trial aimed to determine whether or not oral Δ(9)-tetrahydrocannabinol (Δ(9)-THC)

slowed the course of progressive multiple sclerosis (MS); evaluate safety of

cannabinoid administration; and, improve methods for testing treatments in

progressive MS.

OBJECTIVES: There were three objectives in the CUPID study: (1) to evaluate

whether or not Δ(9)-THC could slow the course of progressive MS; (2) to assess

the long-term safety of Δ(9)-THC; and (3) to explore newer ways of conducting

clinical trials in progressive MS.

DESIGN: The CUPID trial was a randomised, double-blind, placebo-controlled,

parallel-group, multicentre trial. Patients were randomised in a 2 : 1 ratio to

Δ(9)-THC or placebo. Randomisation was balanced according to Expanded Disability

Status Scale (EDSS) score, study site and disease type. Analyses were by

intention to treat, following a pre-specified statistical analysis plan. A

cranial magnetic resonance imaging (MRI) substudy, Rasch measurement theory (RMT)

analyses and an economic evaluation were undertaken.

SETTING: Twenty-seven UK sites.

PARTICIPANTS: Adults aged 18-65 years with primary or secondary progressive MS,

1-year evidence of disease progression and baseline EDSS 4.0-6.5.

INTERVENTIONS: Oral Δ(9)-THC (maximum 28 mg/day) or matching placebo.

ASSESSMENT VISITS: Three and 6 months, and then 6-monthly up to 36 or 42 months.

MAIN OUTCOME MEASURES: Primary outcomes were time to EDSS progression, and change

in Multiple Sclerosis Impact Scale-29 version 2 (MSIS-29v2) 20-point physical

subscale (MSIS-29phys) score. Various secondary patient- and clinician-reported

outcomes and MRI outcomes were assessed. RMT analyses examined performance of

MS-specific rating scales as measurement instruments and tested for a symptomatic

or disease-modifying treatment effect. Economic evaluation estimated mean

incremental costs and quality-adjusted life-years (QALYs).

RESULTS: Effectiveness - recruitment targets were achieved. Of the 498 randomised

patients (332 to active and 166 to placebo), 493 (329 active and 164 placebo)

were analysed.

PRIMARY OUTCOMES: no significant treatment effect; hazard ratio EDSS score

progression (active : placebo) 0.92 [95% confidence interval (CI) 0.68 to 1.23];

and estimated between-group difference in MSIS-29phys score (active-placebo) -0.9

points (95% CI -2.0 to 0.2 points). Secondary clinical and MRI outcomes: no

significant treatment effects. Safety - at least one serious adverse event: 35%

and 28% of active and placebo patients, respectively. RMT analyses - scale

evaluation: MSIS-29 version 2, MS Walking Scale-12 version 2 and MS Spasticity

Scale-88 were robust measurement instruments. There was no clear symptomatic or

disease-modifying treatment effect. Economic evaluation - estimated mean

incremental cost to NHS over usual care, over 3 years £27,443.20 per patient. No

between-group difference in QALYs.

CONCLUSIONS: The CUPID trial failed to demonstrate a significant treatment effect

in primary or secondary outcomes. There were no major safety concerns, but

unwanted side effects seemed to affect compliance. Participants were more

disabled than in previous studies and deteriorated less than expected, possibly

reducing our ability to detect treatment effects. RMT analyses supported

performance of MS-specific rating scales as measures, enabled group- and

individual person-level examination of treatment effects, but did not influence

study inferences. The intervention had significant additional costs with no

improvement in health outcomes; therefore, it was dominated by usual care and not

cost-effective. Future work should focus on determining further factors to

predict clinical deterioration, to inform the development of new studies, and

modifying treatments in order to minimise side effects and improve study

compliance. The absence of disease-modifying treatments in progressive MS

warrants further studies of the cannabinoid pathway in potential neuroprotection.

TRIAL REGISTRATION: Current Controlled Trials ISRCTN62942668.

FUNDING: The National Institute for Health Research Health Technology Assessment

programme, the Medical Research Council Efficacy and Mechanism Evaluation

programme, Multiple Sclerosis Society and Multiple Sclerosis Trust. The report

will be published in full in Health Technology Assessment; Vol. 19, No. 12. See

the NIHR Journals Library website for further project information.

DOI: 10.3310/hta19120

PMCID: PMC4781163

PMID: 25676540 [Indexed for MEDLINE]

205. J Neurosci Nurs. 2015 Feb;47(1):E31-9. doi: 10.1097/JNN.0000000000000100.

Excluded b/c no real-world adherence/persistence

Acceptance of the extracare program by Beta interferon-treated patients with

multiple sclerosis: results of the explore study.

Freidel M(1), Ortler S, Fuchs A, Seibert S, Schuh K.

Author information:

(1)Matthias Freidel, MD, is a Neurologist and Psychiatrist at Neurologische

Praxis, Kaltenkirchen, Germany. Sonja Ortler, PhD, is an Employee at Clinical

Research Neuroscience, Novartis Pharma GmbH, Nuremberg, Germany. Questions or

comments about this article may be directed to Alexander Fuchs, PhD, at

alexander.fuchs@novartis.com. He is an Employee at Clinical Research

Neuroscience, Novartis Pharma GmbH, Nuremberg, Germany. Sabine Seibert, PhD, is

an Employee at Clinical Research Neuroscience, Novartis Pharma GmbH, Nuremberg,

Germany. Katrin Schuh, PhD, is an Employee at Clinical Research Neuroscience,

Novartis Pharma GmbH, Nuremberg, Germany.

BACKGROUND: To gain full benefit from disease-modifying therapies such as

interferon β-1b, patients with multiple sclerosis (MS) need to adhere to

treatment in the long term. Treatment adherence requires high patient

satisfaction with treatment and care.

OBJECTIVES: Our aim was to evaluate the satisfaction of patients with MS

receiving interferon β-1b Extavia with the patient care program Extracare.

Efficacy and safety of treatment were evaluated as secondary objectives.

METHODS: In this prospective, noninterventional 1-year study, data on the

satisfaction of 174 patients with MS with Extracare were obtained by

questionnaires. Disability and symptom severity as well as patients' reported

activity limitations, quality of life, and fatigue were recorded.

RESULTS: We observed high levels of patients' satisfaction with MS nurses,

telephonic care, and information provided by Extracare (values ≤ 1.53 on a Likert

scale ranging from 1 [very good] to 6 [insufficient]). Patient reported quality

of life (Patient Reported Indices for MS QoL) improved from 11.82 ± 11.36 at

baseline to 9.74 ± 10.94 at the end of the study (p = .02), whereas clinical

parameters of disease progression remained unchanged. Rate of adverse events was

as expected.

CONCLUSIONS: This study provides the basis for further improvements of care

programs to increase treatment adherence of patients with MS.

DOI: 10.1097/JNN.0000000000000100

PMCID: PMC4334305

PMID: 25285595 [Indexed for MEDLINE]

206. JAMA Neurol. 2015 Feb;72(2):152-8. doi: 10.1001/jamaneurol.2014.3537.

Excluded b/c no real-world adherence/persistence

Evaluation of no evidence of disease activity in a 7-year longitudinal multiple

sclerosis cohort.

Rotstein DL(1), Healy BC(2), Malik MT(1), Chitnis T(1), Weiner HL(1).

Author information:

(1)Partners Multiple Sclerosis Center, Brigham and Women's Hospital, Boston,

Massachusetts.

(2)Partners Multiple Sclerosis Center, Brigham and Women's Hospital, Boston,

Massachusetts2Biostatistics Center, Massachusetts General Hospital, Brookline,

Massachusetts.

Comment in

JAMA Neurol. 2015 Feb;72(2):145-7.

JAMA Neurol. 2015 Jul;72(7):835-6.

IMPORTANCE: With multiple and increasingly effective therapies for relapsing

forms of multiple sclerosis (MS), disease-free status or no evidence of disease

activity (NEDA) has become a treatment goal and a new outcome measure. However,

the persistence of NEDA over time and its predictive power for long-term

prognosis are unknown.

OBJECTIVE: To investigate NEDA during 7 years as measured by relapses, disability

progression, and yearly magnetic resonance imaging (MRI).

DESIGN, SETTING, AND PARTICIPANTS: Patients were selected from the 2200-patient

Comprehensive Longitudinal Investigation of Multiple Sclerosis at Brigham and

Women's Hospital (CLIMB) cohort study. Patients were required to have an initial

diagnosis of clinically isolated syndrome or relapsing-remitting MS and a minimum

of 7 years of prospective follow-up that included yearly brain MRI and biannual

clinical visits (n = 219). Patients were analyzed independent of

disease-modifying therapy. Patients were classified as having early

(recent-onset) MS if they were 5 years or less from their first MS symptom at

enrollment or otherwise considered to have established MS (>5 years from onset).

MAIN OUTCOMES AND MEASURES: NEDA was defined as a composite that consisted of

absence of relapses, no sustained Expanded Disability Status Scale score

progression, and no new or enlarging T2 or T1 gadolinium-enhancing lesions on

annual MRI. Relapses, progression, and MRI changes were also investigated as

individual outcomes.

RESULTS: A total of 99 of 215 patients (46.0%) had NEDA for clinical and MRI

measures at 1 year, but only 17 of 216 (7.9%) maintained NEDA status after 7

years. No differences were found in NEDA status between patients with early vs

established MS. A dissociation was found between clinical and MRI disease

activity. Each year, 30.6% (64 of 209) to 42.9% (93 of 217) of the cohort had

evidence of either clinical or MRI disease activity but not both. NEDA at 2 years

had a positive predictive value of 78.3% for no progression (Expanded Disability

Status Scale score change ≤0.5) at 7 years. Only minor improvement was found in

the positive predictive values with additional follow-up of 1 to 3 years.

CONCLUSIONS AND RELEVANCE: NEDA is difficult to sustain long term even with

treatment. NEDA status at 2 years may be optimal in terms of prognostic value in

the longer term. Our results provide a basis for investigating NEDA as an outcome

measure and treatment goal and for evaluating the effect of new MS drugs on NEDA.

DOI: 10.1001/jamaneurol.2014.3537

PMID: 25531931 [Indexed for MEDLINE]

Excluded b/c no primary data (narrative review)

207. Antiinflamm Antiallergy Agents Med Chem. 2015;14(1):15-25.

Oral agents in multiple sclerosis.

Lorefice L(1), Fenu G, Frau J, Coghe GC, Marrosu MG, Cocco E.

Author information:

(1)Multiple Sclerosis Center, Department of Public Health and Clinical and

Molecular Medicine, University of Cagliari, Cagliari, Italy.

lorena.lorefice@hotmail.it.

Multiple sclerosis (MS) is a complex autoimmune disease of the central nervous

system. Disease-modifying drugs licensed for MS treatment have been developed to

reduce relapse rates and halt disease progression. The majority of current MS

drugs involve regular, parenteral administration, affecting long-term adherence

and thus reducing treatment efficacy. Over the last two decades great progress

has been made towards developing new MS therapies with different modes of action

and biologic effects. In particular, oral drugs have generated much interest

because of their convenience and positive impact on medication adherence.

Fingolimod was the first launched oral treatment for relapsing-remitting MS;

recently, Teriflunomide and Dimethyl fumarate have also been approved as oral

disease-modifying agents. In this review, we summarize and discuss the history,

pharmacodynamics, efficacy, and safety of oral agents that have been approved or

are under development for the selective treatment of MS.

PMID: 25924620 [Indexed for MEDLINE]

208. Dermatology. 2015;230(1):82-6. doi: 10.1159/000368351. Epub 2014 Dec 5.

Excluded b/c no real-world adherence/persistence

Extracorporeal shock wave therapy for injection site panniculitis in multiple

sclerosis patients.

Stieger M(1), Schmid JP, Yawalkar N, Hunziker T.

Author information:

(1)Department of Dermatology, Bern University Hospital and University of Bern,

Bern, Switzerland.

BACKGROUND: Painful cutaneous injection site reactions may hamper treatment with

interferon β (IFN-β) and glatiramer acetate (GA) in multiple sclerosis (MS)

patients.

OBJECTIVE: To maintain therapy adherence, efficient therapeutic modalities for

these subcutaneous inflammatory lesions are urgently needed. We tested the

application of local extracorporeal shock wave therapy (ESWT).

METHODS: We applied 5 sessions of ESWT to 8 patients suffering from MS who had

developed painful panniculitis at the injection sites of either IFN-β or GA.

Clinical outcomes, i.e. pain reduction and regression of induration, were

assessed 3 and 6 months after completion of the ESWT using a visual analogue

score.

RESULTS: All patients showed both significant pain reduction and reduction of the

skin induration in the treated lesions, while in untreated control lesions there

was no improvement.

CONCLUSION: ESWT proved to be a non-invasive, safe and efficient physical

treatment modality for injection-induced painful cutaneous side effects of

disease-modifying drugs in MS.

© 2014 S. Karger AG, Basel.

DOI: 10.1159/000368351

PMID: 25502016 [Indexed for MEDLINE]

Excluded b/c no real-world adherence/persistence

209. Eur Neurol. 2015;73(1-2):57-65. doi: 10.1159/000365968. Epub 2014 Nov 7.

Second-line therapy with fingolimod for relapsing-remitting multiple sclerosis in

clinical practice: the effect of previous exposure to natalizumab.

Bianco A(1), Patanella AK, Nociti V, Marti A, Frisullo G, Plantone D, De Fino C,

Fetta A, Batocchi AP, Rossini PM, Mirabella M.

Author information:

(1)Institute of Neurology, Catholic University, Rome, Italy.

BACKGROUND: To evaluate efficacy and safety of fingolimod for relapsing-remitting

multiple sclerosis, particularly in patients previously exposed to natalizumab.

METHOD: Prospective observational single-centre second-line cohort study.

RESULTS: Among 71 patients treated with fingolimod 0.5 mg/day for a mean duration

of 21.75 ± 12.60 months, the annualized relapse rate was 0.66 (C.I. 95%

0.27-1.05) with a significant difference between 26 patients with prior

natalizumab exposure (1.15; C.I. 95% 0.12-2.17) and 45 not exposed (0.38; C.I.

95% 0.18-0.57; p = 0.002). In a multivariate negative regression model, only

previous exposure to natalizumab (p = 0.049) and duration of fingolimod treatment

(p < 0.001) significantly correlated with the annualized relapse rate. Previous

exposure to natalizumab (p = 0.028) and duration of treatment with fingolimod (p

< 0.001) were confirmed by restricting the analysis to the first 12 months of

treatment with fingolimod, but were no longer statistically significant by

analysing only patients (n = 51) with at least 12 months of treatment with

fingolimod (0.32; C.I. 95% 0.08-0.55 vs. 0.22; C.I. 95% 0.11-0.32; p = NS). No

differences were observed in neuroradiological outcomes and disability

progression in patients exposed to natalizumab and not exposed. The rate of

discontinuation due to adverse events was 11.3%, with no differences between the

two groups.

CONCLUSIONS: Our study confirms efficacy and side effects of fingolimod in a

second-line clinical practice cohort. Prior natalizumab exposure and duration of

treatment with fingolimod are independent predictors of annualized relapse rate

during the first 12 months of treatment with fingolimod, but not in the

long-term, and may be influenced by the 3 months washout period between the two

drugs.

DOI: 10.1159/000365968

PMID: 25402749 [Indexed for MEDLINE]

210. Expert Opin Drug Deliv. 2015;12(12):1829-35. doi: 10.1517/17425247.2015.1078789.

Excluded b/c no oral DMD results

Epub 2015 Sep 15.

How many injections did you miss last month? A simple question to predict

interferon β-1a adherence in multiple sclerosis.

Moccia M(1), Palladino R(2)(3), Russo C(1), Massarelli M(1), Nardone A(3),

Triassi M(3), Lugaresi A(4), Brescia Morra V(1).

Author information:

(1)a 1 "Federico II" University, Multiple Sclerosis Clinical Care and Research

Centre, Department of Neuroscience, Reproductive Science and Odontostomatology ,

Naples, Italy +39 08 17 46 37 64 ; +39 08 15 46 36 63 ;

moccia.marcello@gmail.com.

(2)b 2 Department of Primary Care and Public Health, Imperial College , London,

UK.

(3)c 3 Federico II University, Department of Public Health , Naples, Italy.

(4)d 4 "Gabriele d'Annunzio" University of Chieti-Pescara, Department of

Neuroscience, Imaging and Clinical Sciences , Chieti, Italy.

OBJECTIVES: Adherence to treatment is of utmost importance in multiple sclerosis

(MS) to achieve full benefits from disease-modifying treatments. Thus, we

investigated predictors of adherence to interferon β-1a.

METHODS: 114 relapsing-remitting MS subjects were recruited and followed-up

during 1.536 ± 0.961 years. RebiSmart® (Ares Trading SA, Coinsins, Switzerland),

an electronic auto-injector, allows real-time recording of adherence which was

retrospectively evaluated, and subjects were categorized accordingly: fully

adherent (if no doses were missed), early or late missing (if missing the first

dose during the first month of observation or later). The occurrence of clinical

relapses and the annualized relapse rate (ARR) were prospectively recorded.

RESULTS: Adherence was 95.0 ± 9.0%. Early missing (n = 17, 14.9%) was more likely

to be associated with the occurrence of a clinical relapse (OR = 4.155; p =

0.018), but not late missing (n = 54, 47.4%) (OR = 1.454; p = 0.408), as compared

to fully adherent (n = 43, 37.7%). Adherence was lower in early missing, as

compared to late missing (p < 0.001). The ARR was higher in early missing, as

compared to late missing and to fully adherent (p < 0.001).

CONCLUSION: MS subjects missing an injection early presented lower adherence, and

a fourfold chance of having a relapse, suggesting a simple way to assess and

categorize adherence in a clinical, real-life setting, where lack of time often

prevents more thorough evaluations.

DOI: 10.1517/17425247.2015.1078789

PMID: 26371561 [Indexed for MEDLINE]

211. Expert Rev Neurother. 2015;15(7):803-24. doi: 10.1586/14737175.2015.1058162. Epub

Excluded b/c no primary data (narrative review)

2015 Jun 22.

Oral drugs in multiple sclerosis therapy: an overview and a critical appraisal.

D'Amico E(1), Leone C, Caserta C, Patti F.

Author information:

(1)Multiple Sclerosis Center, Policlinico G. Rodolico, Rodolico, Catania, 95100,

Italy.

Multiple sclerosis (MS) is characterized by demyelination and axonal loss that

results in progressive disability. Recent advances in understanding the immune

pathogenesis of MS resulted in the introduction of numerous effective drugs with

diverse mechanisms of actions, routes of administration and benefit-risk

profiles. New oral drugs recently approved for MS treatment has led to

significant achievements in MS management. The oral route of administration

promotes patient satisfaction and increases therapeutic compliance; but their

introduction has raised concerns regarding safety and tolerability; and a

thorough analysis of the benefit/risk ratio is required. This article reviews the

mechanisms of action, safety and efficacy of the licensed and experimental oral

drugs in MS. Moreover, we put into perspective the disease, drug and

patient-related factors that should be taken into account when considering the

appropriate oral drug and treatment strategy to the appropriate patient, thus

paving the road for personalized medicine in MS.

DOI: 10.1586/14737175.2015.1058162

PMID: 26098146 [Indexed for MEDLINE]

Excluded b/c no primary data (narrative review)

212. Expert Rev Pharmacoecon Outcomes Res. 2015;15(6):869-72. doi:

10.1586/14737167.2015.1081060. Epub 2015 Aug 20.

Incorporating real-world clinical practice in multiple sclerosis economic

evaluations.

McQueen RB(1), Nair KV(1), Vollmer TL(2), Campbell JD(1).

Author information:

(1)a 1 University of Colorado Anschutz Medical Campus - Skaggs School of Pharmacy

and Pharmaceutical Sciences, 12850 E. Montview Blvd Aurora, Aurora, CO 80045,

USA.

(2)b 2 University of Colorado Anschutz Medical Campus - Neurology, Aurora, CO,

USA.

Using evidence from short-term randomized controlled trials, decision-analytic

models project costs, risks and benefits of disease-modifying therapies (DMTs)

for multiple sclerosis (MS). Such trial-informed models lack the breadth needed

to generalize to clinical practice or policy due to limitations: lack of DMT

switching/discontinuation, limited head-to-head DMT comparisons and efficacy, not

effectiveness, designs. We present an illustrative example that incorporates

treatment switching and discontinuation by estimating the cost-effectiveness

(value) of first-line natalizumab versus second-line natalizumab treatment for

relapsing-remitting MS patients negative for anti-JC virus antibodies. Treating

JC virus-negative relapsing-remitting MS patients with natalizumab as first-line

provided better value compared with second-line. Decision-makers should consider

this evidence for treatment step-edit policies through modeling scenarios closer

to clinical practice.

DOI: 10.1586/14737167.2015.1081060

PMID: 26295727 [Indexed for MEDLINE]

213. Inflamm Allergy Drug Targets. 2015;13(6):351-66.

Excluded b/c no primary data (systematic review)

A systematic review of drugs in late-stage development for the treatment of

multiple sclerosis: a focus on oral synthetic drugs.

Safavi M, Nikfar S, Abdollahi M(1).

Author information:

(1)Department of Toxicology and Pharmacology, Faculty of Pharmacy and

Pharmaceutical Sciences Research Center, and Endocrinology & Metabolism Research

Center, Institute of Clinical Endocrine Sciences, Tehran University of Medical

Sciences, Tehran 1417614411, Iran. Mohammad@TUMS.Ac.Ir.

Multiple sclerosis (MS) is a heterogeneous, chronic, debilitating immune-mediated

disease of the central nervous system (CNS). There are four types of MS according

to their relapsing or progressive pattern that include relapsing-remitting

(RRMS), secondary progressive (SPMS), primary progressive (PPMS), and progressive

relapsing (PRMS). There is no definite cure for MS, thus medications typically

focus on slowing the progression of the disease, managing symptoms and improving

the quality of life. There is no specific medication for the management of PPMS

and thus these patients are often neglected. New medicines in this phase of the

disease are needed. On the other hand injectable immunomodulatory medicines,

which dominated the MS market for over the past two decades, raise the issues of

adherence and tolerance while oral therapies do offer a step forward in

convenience. This systematic review article discusses the emerging synthetic

small molecule that administered orally for MS treatment. We searched PubMed, Web

of Science and Google Scholar to summaries the present knowledge on mechanism of

action, and completed and current clinical trial of laquinimod, masitinib and

siponimod. Data were collected from 1985 to January 2015. The development of

effective medicines for MS is critically dependent upon understanding the

biological basis of this complex multifactorial disease. The current

pharmacotherapeuetic options for its treatment are mainly immunomodulators which

were developed on the basis that MS is an autoimmune disease. The new synthetic

small molecule agents such as laquinimod, masitinib and siponimod with different

mechanism of actions can be administered orally rather than by injection.

PMID: 26021323 [Indexed for MEDLINE]

214. Int J Neurosci. 2015;125(9):678-85. doi: 10.3109/00207454.2014.969839. Epub 2014

Oct 29.

Experience with fingolimod in clinical practice.

Hersh CM(1), Hara-Cleaver C, Rudick RA, Cohen JA, Bermel RA, Ontaneda D.

Author information:

(1)1Mellen Center for Multiple Sclerosis Treatment and Research, Neurological

Institute, Cleveland Clinic, Cleveland, OH, USA.

AIM: To report experience with fingolimod in clinical practice.

DESIGN/METHODS: Patients in an academic medical center who were prescribed

fingolimod from October 2010 to August 2011 were identified through the

electronic medical record and followed for 12 months after fingolimod initiation.

Adverse effects (AEs), clinical measures, MRI data, and quality of life measures

were assessed.

RESULTS: Three hundred seventeen patients started fingolimod. Eleven patients

were treatment naïve (3.5%) and 76 (24.0%) had remote disease modifying therapy

(DMT) use prior to fingolimod. One hundred fifty-one (47.6%) switched because of

patient preference and 79 (24.9%) switched because of breakthrough disease. About

11.6% transitioned from natalizumab. Follow-up data were available for 306

patients (96.5%) with mean follow-up time 332 days. Fingolimod was discontinued

in 76 of 306 patients (24.8%) at mean 248 days after fingolimod start.

Discontinuation most often was due to AEs (n = 40) or breakthrough disease (n =

22). Among patients who started fingolimod with available 12 month follow-up

data, 267 (87.3%) remained relapse free and 256 (83.7%) had no relapses or

gadolinium enhancement. Time to first relapse occurred at mean 282 days after

fingolimod initiation. Quality of life measures remained stable at follow-up.

CONCLUSIONS: Fingolimod was discontinued at a higher rate in clinical practice

than in clinical trials. Discontinuation was primarily due to AEs or breakthrough

disease. Disease activity was adequately controlled in most patients who started

fingolimod. This clinical practice cohort is consistent with efficacy data from

phase 3 trials and describes the most common tolerability issues in clinical

practice.

DOI: 10.3109/00207454.2014.969839

PMCID: PMC4414718

PMID: 25271798 [Indexed for MEDLINE]

215. J Clin Neurol. 2015 Jan;11(1):9-19. doi: 10.3988/jcn.2015.11.1.9. Epub 2015 Jan

Excluded b/c no primary data (narrative review)

2.

Oral disease-modifying therapies for multiple sclerosis.

Kim W(1), Zandoná ME(2), Kim SH(3), Kim HJ(3).

Author information:

(1)Department of Neurology, College of Medicine, The Catholic University of

Korea, Seoul, Korea.

(2)Pontifical Catholic University of Rio Grande do Sul, Science Without Borders,

Porto Alegre, Brazil. ; Department of Neurology, Research Institute and Hospital

of National Cancer Center, Goyang, Korea.

(3)Department of Neurology, Research Institute and Hospital of National Cancer

Center, Goyang, Korea.

Classical multiple sclerosis (MS) treatments using first-line injectable drugs,

although widely applied, remain a major concern in terms of therapeutic adherence

and efficacy. New oral drugs recently approved for MS treatment represent

significant advances in therapy. The oral route of administration clearly

promotes patient satisfaction and increases therapeutic compliance. However,

these drugs may also have safety and tolerability issues, and a thorough analysis

of the risks and benefits is required. Three oral drugs have been approved by

regulatory agencies for MS treatment: fingolimod, teriflunomide, and dimethyl

fumarate. This article reviews the mechanisms of action, safety, and efficacy of

these drugs and two other drugs that have yielded positive results in phase III

trials: cladribine and laquinimod.

DOI: 10.3988/jcn.2015.11.1.9

PMCID: PMC4302185

PMID: 25628732

Excluded b/c no real-world adherence/persistence

216. J Med Econ. 2015;18(12):1085-91. doi: 10.3111/13696998.2015.1076826. Epub 2015

Sep 21.

The budget impact of introducing delayed-release dimethyl fumarate for treatment

of relapse-remitting multiple sclerosis in Canada.

Dorman E(1), Kansal AR(1), Sarda S(2).

Author information:

(1)a a Evidera , Bethesda , MD , USA.

(2)b b Biogen Idec , Cambridge , MA , USA.

OBJECTIVE: Multiple sclerosis (MS) causes significant disability globally and is

especially prevalent in Canada. Delayed-release dimethyl fumarate (DMF; also

known as gastro-resistant DMF) is an orally administered disease-modifying

treatment (DMT) for patients with relapsing-remitting MS (RRMS) that is currently

on the market in the US, Australia, Canada, and Europe. A budget impact model

(BIM) was developed to assess the financial consequences of introducing DMF for

treatment of RRMS in Canada.

METHODS: A BIM calculated the financial consequences of introducing DMF in Canada

over 3 years based on RRMS prevalence, treatment market share, and clinical

effects. RRMS prevalence in Canada was derived from published literature and

natural relapse rates, and disease state distribution from clinical trial data.

It was conservatively assumed that 100% of RRMS patients were treated with a DMT.

DMF was assumed to absorb market share proportionally from the following current

treatments: interferon beta-1a-IM, interferon beta-1a-SC, interferon beta-1b, and

glatiramer acetate. Treatment efficacy, in terms of relapse rate reductions and

treatment discontinuation rates, was determined from mixed treatment comparison.

Treatment costs (including costs of acquisition, monitoring, and administration)

and cost of relapse were considered. Deterministic one-way sensitivity analyses

were conducted to assess the most sensitive input parameters.

RESULTS: Over 3 years, the introduction of DMF resulted in an average annual

increase of CAD417 per treated patient per year, with reductions in costs

associated with relapses (CAD192/patient/year) partially offsetting increased

drug acquisition costs (CAD602/patient/year). On a population level, the average

annual cost increase was CAD24,654,237, a CAD 0.68 increase per population

covered by the Canadian healthcare system. The main drivers of budget impact were

drop-out rates, proportion of RRMS patients treated, and market share

assumptions.

CONCLUSIONS: The acquisition costs of DMF for treatment of RRMS are predicted to

be partially offset by reduced costs of relapses in the Canadian healthcare

system.

DOI: 10.3111/13696998.2015.1076826

PMID: 26390149 [Indexed for MEDLINE]

217. J Med Econ. 2015;18(9):711-20. doi: 10.3111/13696998.2015.1044276. Epub 2015 Jun

Excluded b/c no oral DMD results

1.

Impact of increasing adherence to disease-modifying therapies on healthcare

resource utilization and direct medical and indirect work loss costs for patients

with multiple sclerosis.

Yermakov S(1), Davis M, Calnan M, Fay M, Cox-Buckley B, Sarda S, Duh MS, Iyer R.

Author information:

(1)a a Analysis Group , Boston , MA , USA.

OBJECTIVES: To estimate the effect of adherence to disease-modifying therapies

(DMTs) among patients with multiple sclerosis (MS) on healthcare resource

utilization (HRU) and costs, and model the impact of a 10 percentage point

increase in adherence on these outcomes.

METHODS: Employed patients, 18-64 years old, with ≥2 MS diagnoses and ≥1 DMT

claim during January 1, 2002 to September 30, 2012 were identified from a large

commercially-insured US claims database. Adherence was measured as proportion of

days covered (PDC) during follow-up. Multivariate regression analyses were

conducted to estimate the effect of adherence on HRU related to urgent care

(i.e., inpatient or emergency room visit), days of work loss, direct medical

cost, and indirect work loss costs. Model coefficients were used to evaluate the

impact of a 10 percentage point increase in adherence on the outcomes.

RESULTS: A total of 1510 patients were included (mean age = 43.4 years, 64%

female). Patients with higher adherence had lower HRU, fewer days of work loss,

and lower direct and indirect costs. A 10 percentage point increase in adherence

significantly decreased the likelihood of an inpatient or emergency room visit by

9-19%, days of work loss by 3-8%, and direct and indirect costs by 3-5%,

depending on the follow-up period (all p < 0.01).

CONCLUSIONS: Increasing DMT adherence was found to significantly decrease

urgent-care HRU, days of work loss, and direct and indirect costs among patients

with MS.

DOI: 10.3111/13696998.2015.1044276

PMID: 25903661 [Indexed for MEDLINE]

218. Mult Scler Int. 2015;2015:809252. doi: 10.1155/2015/809252. Epub 2015 Sep 21.

Excluded b/c no primary data (narrative review)

Neuroimaging of Natalizumab Complications in Multiple Sclerosis: PML and Other

Associated Entities.

Honce JM(1), Nagae L(1), Nyberg E(1).

Author information:

(1)Division of Neuroradiology, University of Colorado School of Medicine, 12700 E

19th Avenue Mail Stop C278, Aurora, CO 80045, USA.

Natalizumab (Tysabri) is a monoclonal antibody (α4 integrin antagonist) approved

for treatment of multiple sclerosis, both for patients who fail therapy with

other disease modifying agents and for patients with aggressive disease.

Natalizumab is highly effective, resulting in significant decreases in rates of

both relapse and disability accumulation, as well as marked decrease in MRI

evidence of disease activity. As such, utilization of natalizumab is increasing,

and the presentation of its associated complications is increasing accordingly.

This review focuses on the clinical and neuroimaging features of the major

complications associated with natalizumab therapy, focusing on the rare but

devastating progressive multifocal leukoencephalopathy (PML). Associated entities

including PML associated immune reconstitution inflammatory syndrome (PML-IRIS)

and the emerging phenomenon of rebound of MS disease activity after natalizumab

discontinuation are also discussed. Early recognition of neuroimaging features

associated with these processes is critical in order to facilitate prompt

diagnosis, treatment, and/or modification of therapies to improve patient

outcomes.

DOI: 10.1155/2015/809252

PMCID: PMC4592919

PMID: 26483978

219. Mult Scler Relat Disord. 2015 Jan;4(1):75-82. doi: 10.1016/j.msard.2014.09.214.

Excluded b/c no oral DMD results

Epub 2014 Oct 7.

Therapy Optimization in Multiple Sclerosis: a cohort study of therapy adherence

and risk of relapse.

Cohen BA(1), Coyle PK(2), Leist T(3), Oleen-Burkey MA(4), Schwartz M(5), Zwibel

H(6).

Author information:

(1)Department of Neurology, Northwestern University, 710 North Lake Shore Drive,

Abbott Hall 1121, Chicago, IL 60611, USA. Electronic address:

bac106@northwestern.edu.

(2)Department of Neurology, Stony Brook University, Health Sciences Center,

T12-020, Stony Brook, NY 11794, USA. Electronic address:

Patricia.Coyle@stonybrookmedicine.edu.

(3)Comprehensive MS Center, Thomas Jefferson University, 900 Walnut St. #200,

Philadelphia, PA 19107, USA. Electronic address: thomas.leist@jefferson.edu.

(4)Health Outcomes Research Consulting and Writing, Outcomes Scribe, LLC, 664

Wynding Oaks, Kalamazoo, MI 49006, USA. Electronic address:

OutcomesScribe@gmail.com.

(5)Biostatistics, MedNet Solutions, Inc., 110 Cheshire Lane, Ste. 300,

Minnetonka, MN 55305, USA. Electronic address: mschwartz@mednetstudy.com.

(6)Neuroscience Consultants, Comprehensive Multiple Sclerosis Center, 4601 Ponce

de Leon Blvd, Ste. 100, Coral Gables, FL 33146, USA. Electronic address:

zwibelmdms@aol.com.

OBJECTIVES: The objective of the Therapy Optimization in MS (TOP MS) Study was to

prospectively assess the relationship between MS disease-modifying therapy (DMT)

adherence and MS relapse risk over 2 years.

METHODS: Potential participants were recruited for TOP MS by specialty pharmacies

who dispensed glatiramer acetate and beta interferons for MS nationwide. Signed

IRB-approved informed consents were returned to the pharmacies. TOP MS used

electronic data capture with monthly patient entries. Adherence, measured by

medication possession ratio (MPR), was derived from pharmacy shipment records.

Logistic regression examined the association between protocol-defined relapses

and DMT MPR (<0.5; >0.5-<0.9; >0.9).

RESULTS: TOP MS enrolled 3151 persons with MS, and 2410 completed the full 2

years. Across all therapies, the mean MPR for the 2-year completer cohort of 2049

who maintained the same DMT was 0.9+0.2 (range: 0.1-1.0), with 63.8% reaching a

2-year MPR >0.9. Evaluated by categories of MPR, the proportion of participants

remaining relapse-free for 24 months increased with increasing MPR, and the

proportion with >1 relapses declined with increasing levels of MPR (p<0.0008).

Regression analysis revealed the odds of relapse for a patient in the MPR >0.9

MPR group was 64% that of a patient in the MPR <0.5 category (p=0.02). Use of >1

DMT prior to the current one was an independent predictor of relapse.

CONCLUSIONS: The study provides class III evidence that improvement in adherence

to DMT for MS is associated with improved clinical outcomes as measured by

relapse reduction.

Copyright © 2014 Elsevier B.V. All rights reserved.

DOI: 10.1016/j.msard.2014.09.214

PMID: 25787057 [Indexed for MEDLINE]

220. Neurosci J. 2015;2015:389360. doi: 10.1155/2015/389360. Epub 2015 Nov 26.

Excluded b/c no primary data (narrative review)

Fingolimod Real World Experience: Efficacy and Safety in Clinical Practice.

Fonseca J(1).

Author information:

(1)University of Aveiro, 3810-193 Aveiro, Portugal ; Novartis Farma SA, 2710-693

Sintra, Portugal.

Fingolimod is a multiple sclerosis treatment licensed in Europe since 2011. Its

efficacy has been demonstrated in three large phase III trials, used in the

regulatory submissions throughout the world. As usual, in these trials the

inclusion and exclusion criteria were designed to obtain a homogeneous

population, with interchangeable characteristics in the different treatment arms.

Although this is the best strategy to achieve a robust answer to the

investigation question, it does not guaranty the treatment efficacy in the

clinical practice, since in the real world there are concomitant treatments,

comorbidities, adherence, and persistence challenges. But, to make informed

treatment decision for a real life patient, we need to have evidence of the

treatment efficacy, what has been called treatment effectiveness. This work aims

to review fingolimod effectiveness, using, as source of information, abstracts,

posters, and manuscripts. This unorthodox strategy was developed because more

than half of the published experience with fingolimod is still on abstracts and

posters. Only a small part of the studies reviewed are already published in peer

reviewed journals. Fingolimod seems to be, at least, as effective and safe as it

was on clinical trials, and with its long-term experience no new safety signals

were observed.

DOI: 10.1155/2015/389360

PMCID: PMC4674597

PMID: 26693475

221. Oral Surg Oral Med Oral Pathol Oral Radiol. 2015 Jan;119(1):e13-9. doi:

Excluded b/c no real-world adherence/persistence

10.1016/j.oooo.2014.08.023. Epub 2014 Sep 16.

mTOR inhibitor-associated stomatitis (mIAS) in three patients with cancer treated

with everolimus.

Kalogirou EM(1), Tosios KI(2), Piperi EP(3), Sklavounou A(4).

Author information:

(1)Postgraduate Student, Department of Oral Pathology and Medicine, Dental

School, University of Athens, Athens, Greece.

(2)Assistant Professor, Department of Oral Pathology and Medicine, Dental School,

University of Athens, Athens, Greece. Electronic address: ktosios@dent.uoa.gr.

(3)Lecturer, Department of Oral Pathology and Medicine, Dental School, University

of Athens, Athens, Greece.

(4)Professor, Department of Oral Pathology and Medicine, Dental School,

University of Athens, Athens, Greece.

Mammalian targets of rapamycin inhibitors (mTOR inhibitors, mTORI) are indicated

for the management of several cancer types, including hormone receptor--positive

or HER2-negative breast cancer, advanced renal cell carcinoma, advanced

neuroendocrine tumors of pancreatic origin, and tuberous sclerosis

complex-related tumors. Among the most common adverse events of mTORI medication

are discrete, large, solitary or multiple, superficial ulcers, almost exclusively

situated on nonkeratinized oral mucosa, described as mTORI-associated stomatitis

(mIAS). We describe the clinical presentation, course, and management of mIAS in

three patients receiving the mTORI everolimus (Afinitor, Novartis, East Hanover,

NJ). In two patients, mIAS manifested 9 and 30 days after first using everolimus,

respectively, whereas in the third patient, it recurred 3 months after

re-introduction of everolimus. Oral rinses with a "magic mouthwash" solution

(dexamethasone oral drops solution 2 mg/mL × 10 mL, lidocaine gel 2% × 30 g,

doxycycline suspension 50 mg/5 mL × 60 mL, and sucralfate oral suspension 1000

mg/5 mL × 150 mL, dissolved in sodium chloride 0.9% × 2000 mL) four times daily

proved helpful in alleviating the symptoms, and the ulcers healed in 4 to 15

days. No side effects were recorded, and dose reduction or discontinuation of

everolimus was not necessitated in two cases.

Copyright © 2015 Elsevier Inc. All rights reserved.

DOI: 10.1016/j.oooo.2014.08.023

PMID: 25442249 [Indexed for MEDLINE]

222. Pneumologia. 2015 Jan-Mar;64(1):40-5.

Excluded b/c no real-world adherence/persistence

Mediastinal fibrosis and Hodgkin lymphoma mimicking bronchiolitis obliterans

organizing pneumonia.

Nedelcu RE, Kiss E, Ciorba M, Galbenu P, Ulmeanu R.

Bronchiolitis obliterans organizing pneumonia (BOOP) represents a kaleidoscope of

concepts and morphologies, often being confused with a series of conditions,

among which the most feared are Hodgkin's lymphoma and bronchioloalveolar

carcinoma. We shall present the case of a 56-year-old patient, diagnosed in

August 2013 with a pulmonary tumour of the right upper lobe, which was CTstaged -

T4N0M0 (IIIA), who underwent a video-assisted thoracotomy for histopathological

confirmation. A mediastino-pulmonary formation had been detected intraoperatively

and multiple biopsies had been collected. The information brought by the

histopathological examination suggested the presence of 2 synchronous

pathologies, namely: the mediastinal biopsy showed an advanced degree of dense,

compact fibrosis and the pulmonary biopsy highlighted the presence of granulation

tissue and Masson bodies in the distal airspace with destruction of vascular and

alveolar structures, an aspect which was compatible with organizing pneumonia

(BOOP). Once a diagnosis was established, an oral corticosteroid therapy was

initiated (Prednisone 30 mg/day) over a period of one month, but the

symptomatology of the patient worsened. A new thoracic CT carried out in November

2013 highlighted the progression and extension of the paramediastinal tumoral

formation, exhibiting central necrosis and invading the mediastinal vessels,

causing their compression (superior vena cava syndrome) associated with multiple

mediastinal and hilar adenopathies. The non-favorable evolution and the extensive

array of conditions that may mimic the BOOP histopathological pattern have been

the key elements, which were the basis of our persistence in getting a real

diagnosis. Therefore, in this respect, the biopsy parts performed by thoracotomy

were sent for immunohistochemical testing. The CD30 and CD15 positive markers

distinctive for Reed-Sternberg cells allowed the diagnosis of Hodgkin's lymphoma.

PMID: 26016055 [Indexed for MEDLINE]

Excluded b/c no primary data (systematic review)

223. Patient Prefer Adherence. 2014 Dec 4;8:1653-64. doi: 10.2147/PPA.S67253.

eCollection 2014.

0078xx	Treatment adherence and other patient-reported outcomes as cost determinants in

multiple sclerosis: a review of the literature.

Lizán L(1), Comellas M(1), Paz S(1), Poveda JL(2), Meletiche DM(3), Polanco C(4).

Author information:

(1)Outcomes'10, Castellón, Spain.

(2)Hospital Universitario La Fe, Valencia, Spain.

(3)EMD Serono Inc., Rockland, MA, USA.

(4)Merck S.L., Madrid, Spain.

BACKGROUND: Treatment adherence is one of the key factors for achieving optimal

clinical outcomes. In order to assess costs related to adherence to, and

persistence and compliance with, disease-modifying therapies (DMTs) in patients

with multiple sclerosis (MS), a narrative review of the literature was performed.

Satisfaction with and preference for DMTs and their delivery devices were also

assessed, as both can have an influence on patients' adherence and persistence.

METHODS: Electronic databases (MEDLINE, PubMed, Google Scholar, congress

proceedings) were searched to identify publications analyzing MS costs related to

adherence, persistence, satisfaction, and preferences for MS treatments.

Bibliographic references were hand searched. English or Spanish studies published

between January 2007 and January 2013 were selected.

RESULTS: A total of 398 titles were identified, of which 12 met the inclusion

criteria. Six studies evaluated the impact of adherence, persistence, and

compliance on treatment costs; four publications analyzed satisfaction with DMTs;

and two assessed treatment preferences based on attributes of the delivery

device. Increased adherence and persistence were associated with better clinical

outcomes, leading to lower relapse risk (odds ratio [OR]: 0.71; 95% confidence

interval [CI]: 0.59-0.85) and a decrease in health care resource use, such as

MS-related hospitalizations (OR: 0.63; 95% CI: 0.47-0.83) and emergency

department visits (OR: 0.80; 95% CI: 0.60-1.07). This reduction in resource use

led to a patient/year total cost reduction (excluding DMT costs) of up to 22%.

CONCLUSION: This review highlights the importance of ensuring adequate adherence

in MS patients through treatments and devices better tailored to patients' needs

that could enhance clinical outcomes and reduce MS costs. Understanding the

factors underlying satisfaction and compliance with treatment and patients'

preference for certain therapies could help in the development of strategies that

can improve adherence.

DOI: 10.2147/PPA.S67253

PMCID: PMC4262214

PMID: 25525341

224. Expert Opin Drug Deliv. 2014 Dec;11(12):1969-78. doi:

Excluded b/c no primary data (narrative review)

10.1517/17425247.2014.943181. Epub 2014 Sep 26.

Single-use autoinjector for once-weekly intramuscular injection of IFNβ-1a.

Limmroth V(1), Gerbershagen K.

Author information:

(1)Klinikum Köln-Merheim, Klinik für Neurologie und Palliativmedizin ,

Ostmerheimer Str. 200 D-51109 Köln , Germany LimmrothV@kliniken-koeln.de.

INTRODUCTION: IFNβ products and glatiramer acetate are established treatment

first-line options in long-term disease-modifying therapy of multiple sclerosis

(MS). These self-injectable medications are used once weekly to once daily.

Injection-related issues are common patient-cited reasons for nonadherence.

Autoinjectors have been shown to support long-term adherence to injectable

medications. The ability to self-inject in MS patients has been associated with a

reduced risk of missed injections and drug discontinuation, and a beneficial

effect on patient independence.

AREAS COVERED: The recently introduced easy-to-use prefilled once-weekly pen is a

safe and effective device for intramuscular (IM) IFNβ-1a application and provides

a convenient method for self-injection. We reviewed the available published

evidence on the characteristics of this device. The once-weekly pen facilitates

self-injection and was preferred over prefilled syringes by patients in a

prospective open-label, multicenter Phase IIIb trial in MS patients who had been

using IM IFNβ-1a in prefilled syringes.

EXPERT OPINION: The simple and safe handling, shielded short needle, single-use

disposable design and virtually painless injection by the device may contribute

to adherence, quality of life and independence in patients using IM IFNβ-1a.

DOI: 10.1517/17425247.2014.943181

PMID: 25255732 [Indexed for MEDLINE]

225. Expert Rev Neurother. 2014 Nov;14(11):1243-50. doi: 10.1586/14737175.2014.971758.

Excluded b/c no real-world adherence/persistence

Evaluation of the tolerability and efficacy of Sativex in multiple sclerosis.

Moreno Torres I(1), Sanchez AJ, Garcia-Merino A.

Author information:

(1)Hospital Universitario Puerta de Hierro, Laboratorio de Neuroinmunologia,

Majadahonda, 28222, Spain.

Refractory spasticity, central neuropathic pain and bladder dysfunction are

common clinical problems in patients with multiple sclerosis (MS). None of the

currently available oral medications has proven to be reliably effective and can

be limited by toxicity. Cannabinoids have shown therapeutic effects on those

MS-associated symptoms. Delta-9-tetrahydrocannabinol (THC)/cannabidiol (CBD)

Sativex (nabiximols) is an oromucosal spray formulation that contains THC and CBD

in an approximate 1:1 ratio and is described as an endocannabinoid system

modulator. The efﬁcacy of THC/CBD on MS-associated spasticity, pain and bladder

dysfunction has been studied in clinical trials as well as in clinical practice

studies. Adverse effects are usually mild or moderate and the low rate of drug

discontinuation provides good evidence of long-term tolerability. This article

focuses on the pharmacological properties, clinical efﬁcacy and tolerability of

THC/CBD in MS patients.

DOI: 10.1586/14737175.2014.971758

PMID: 25331416 [Indexed for MEDLINE]

226. Mult Scler. 2014 Nov;20(13):1714-20. doi: 10.1177/1352458514533398. Epub 2014 May

19.

Excluded b/c no real-world adherence/persistence

Predictors for multiple sclerosis relapses after switching from natalizumab to

fingolimod.

Hoepner R(1), Havla J(2), Eienbröker C(3), Tackenberg B(3), Hellwig K(1), Meinl

I(2), Hohlfeld R(4), Gold R(1), Kümpfel T(2), Kleiter I(5).

Author information:

(1)St Josef-Hospital, Ruhr-University, Germany.

(2)Institute of Clinical Neuroimmunology, Ludwig-Maximilians-University, Germany.

(3)Clinical Neuroimmunology Group, Philipps-University, Germany.

(4)Institute of Clinical Neuroimmunology, Ludwig-Maximilians-University,

GermanyMunich Cluster for Systems Neurology (SyNergy), Germany.

(5)St Josef-Hospital, Ruhr-University, Germany ingo.kleiter@rub.de.

BACKGROUND: Risks of natalizumab (NAT) therapy have to be weighed against disease

recurrence after stopping NAT.

OBJECTIVES: The objective of this paper is to identify risk factors for

recurrence of relapses after switching from NAT to fingolimod (FTY) in

relapsing-remitting multiple sclerosis (RRMS).

METHODS: Patients (n = 33) were treated with NAT for ≥1 year, and then switched

to FTY within 24 weeks (mean follow-up on FTY 81.1 (SD 26.5) weeks). Annual

relapse rates (ARR) and Expanded Disability Status Scale scores (EDSS) were

assessed. Descriptive statistics, univariate logistic regression analysis, and

receiver operating characteristic curves were conducted.

RESULTS: Overall, 20 patients (61%) had relapses after discontinuation of NAT and

16 (48%) during FTY therapy. The maximum incidence of relapses occurred between

weeks 13-24 post-NAT. The last EDSS during the switching period predicted

relapses during subsequent FTY therapy. EDSS >3 separated most powerfully between

the groups (sensitivity 64%, specificity 88%) and significantly predicted

relapses (relative risk 3.27, 95% CI: 1.5-7.3). Seventy-five percent of patients

with EDSS ≤ 3 remained free of relapses, compared to 18% of patients with EDSS

>3.

CONCLUSIONS: There was an increase of the ARR in the first year after switching

from NAT to FTY. Last EDSS during the switching period was a predictor of

relapses during FTY.

© The Author(s), 2014.

DOI: 10.1177/1352458514533398

PMID: 24842961 [Indexed for MEDLINE]

227. Mult Scler. 2014 Nov;20(13):1795-8. doi: 10.1177/1352458514531348. Epub 2014 Apr

Excluded b/c no real-world adherence/persistence

22.

Adherence in multiple sclerosis (ADAMS): classification, relevance, and research

needs. A meeting report.

Heesen C(1), Bruce J(2), Feys P(3), Sastre-Garriga J(4), Solari A(5), Eliasson

L(6), Matthews V(7), Hausmann B(8), Ross AP(9), Asano M(10), Imonen-Charalambous

K(11), Köpke S(12), Clyne W(13), Bissell P(14).

Author information:

(1)University Medical Center, Martinistrasse 52, Hamburg, 20246, Germany

heesen@uke.uni-hamburg.de.

(2)University of Missouri-Kansas City, Kansas City, Missouri, USA.

(3)Hasselt University, Belgium.

(4)Servei de Neurologia / Neuroimmunologia, Multiple Sclerosis Centre of

Catalonia (Cemcat), Hospital Universitari Vall d'Hebron, Universitat Autònoma de

Barcelona, Barcelona, Spain.

(5)Istituto Neurologico C. Besta, Milan, Italy.

(6)Alantis Healthcare, USA.

(7)MS Trust, UK.

(8)European MS Platform, Belgium.

(9)International Organization of MS Nurses, USA.

(10)Queens University, Canada.

(11)European Patient's Forum, Belgium.

(12)University Medical Center, Lübeck, Germany.

(13)Coventry University, UK.

(14)Section of Public Health, Sheffield, UK.

BACKGROUND: Adherence to medical interventions is a global problem. With an

increasing amount of partially effective but expensive drug treatments adherence

is increasingly relevant in multiple sclerosis (MS). Perceived lack of efficacy

and side effects as well as neuropsychiatric factors such as forgetfulness,

fatigue and depression are major determinants. However, research on adherence to

behavioural interventions as part of rehabilitative interventions has only rarely

been studied.

METHODS: In a one-day meeting health researchers as well as patient

representatives and other stakeholders discussed adherence issues in MS and

developed a general draft research agenda within a focus group session.

RESULTS: The focus group addressed four major areas: (1) focussing patients and

their informal team; (2) studying health care professionals; (3) comparing

practice across cultures; and (4) studying new adherence interventions.

CONCLUSIONS: A focus on patient preferences as well as a non-judgmental

discussion on adherence issues with patients should be at the core of adherence

work.

© The Author(s), 2014.

DOI: 10.1177/1352458514531348

PMID: 24756569 [Indexed for MEDLINE]

228. Mult Scler Relat Disord. 2014 Nov;3(6):689-95.

Excluded b/c pediatric patients

Treatment adherence and transitioning youth in pediatric multiple sclerosis.

Lulu S(1), Julian L, Shapiro E, Hudson K, Waubant E.

Author information:

(1)University of California, San Francisco, CA, UnitedStates

BACKGROUND: Transitioning youth with multiple sclerosis (MS) represent a

vulnerable group to potentially poor outcomes. It is unknown how pediatric MS

patients transition into adult care.

OBJECTIVES: To describe self-management skills that include adherence to

disease-modifying therapies, quality of life measures, illness perception,

transition readiness and healthcare skills assessments in patients with pediatric

MS and associations with clinical and cognitive outcomes.

METHODS: This is a prospective cross-sectional study at the pediatric MS center

and transitional MS clinic at the University of California, San Francisco.

Patients and one of their parents completed validated surveys for self-management

skills. Non-adherence is defined as not taking their medication more than 20% of

the time in the past 1 month. Wilcoxin matched-pairs rank test and McNemar's

tests were used for comparison of patient and parent responses. Univariate and

multivariate regression models were used for analyses adjusting for disease

duration and socio-economic status.

RESULTS: Thirty patients were enrolled with a mean (+/-SD) age of 15.8

years+/-2.8, 53% was female and 47% Hispanic. The rate of non-adherence was 37%.

The most common reason for non-adherence was forgetting to take their medication

reported in 50% of patients. In adjusted regression models, higher EDSS was

associated with a lower score on patient's quality of life (13 points decrease,

95% CI 6–18, p<0.0001), and lower healthcare skills (15 points decrease, 95%

CI5–26, p=0.006). Four points increase in Symbol Digit Modalities Test score was

associated a 0.1 increase in transition readiness score (95% CI0.07–0.2, p=0.001)

and 3.9 points increase in health care skills scores (95% CI 1.7–6, p=0.008).

DOI: 10.1016/j.msard.2014.09.088

PMCID: PMC4361811

PMID: 25798373 [Indexed for MEDLINE]

229. Value Health. 2014 Nov;17(7):A401. doi: 10.1016/j.jval.2014.08.913. Epub 2014 Oct

Excluded b/c abstract only

26.

Persistence in Open and Closed Data Sources: A Study of Fingolimod Verse

Interferons/Glatiramer Acetate in Patients with Multiple Sclerosis.

Lahoz R(1), Bergvall N(1), Nazareth T(2), Korn JR(3).

Author information:

(1)Novartis Pharma AG, Basel, Switzerland.

(2)Novartis Pharmaceuticals Corporation, East Hanover, NJ, USA.

(3)IMS Health, Waltham, MA, USA.

DOI: 10.1016/j.jval.2014.08.913

PMID: 27200959

230. Value Health. 2014 Nov;17(7):A400. doi: 10.1016/j.jval.2014.08.911. Epub 2014 Oct

Excluded b/c abstract only

26.

Modelling the Persistence of Disease-Modifying Drug Treatment (DMT) and its

Independent Drivers in Finnish Multiple Sclerosis (MS) Patients: Parametric

Survival Modelling.

Soini E(1), Holmberg M(2), Asseburg C(1), Sumelahti ML(2).

Author information:

(1)ESiOR Oy, Kuopio, Finland.

(2)School of Medicine, University of Tampere, Finland.

DOI: 10.1016/j.jval.2014.08.911

PMID: 27200954

231. Value Health. 2014 Nov;17(7):A400-1. doi: 10.1016/j.jval.2014.08.912. Epub 2014

Excluded b/c abstract only

Oct 26.

Persistence with Fingolimod Versus Dimethyl Fumarate in Patients with Multiple

Sclerosis: Retrospective Analysis of us Open-Source Pharmacy Data.

Bergvall N(1), Lahoz R(1), Nazareth T(2), Korn JR(3).

Author information:

(1)Novartis Pharma AG, Basel, Switzerland.

(2)Novartis Pharmaceuticals Corporation, East Hanover, NJ, USA.

(3)IMS Health, Waltham, MA, USA.

DOI: 10.1016/j.jval.2014.08.912

PMID: 27200952

232. Value Health. 2014 Nov;17(7):A400. doi: 10.1016/j.jval.2014.08.910. Epub 2014 Oct

Excluded b/c abstract only

26.

Using a Panel Survey to Identify Predictors of Disease-Modifying Drug Adherence

in Patients with Multiple Sclerosis.

Phillips AL(1), Kozma CM(2), Locklear JC(1).

Author information:

(1)EMD Serono, Inc., Rockland, MA, USA.

(2)Independent Research Consultant, Saint Helena Island, SC, USA.

DOI: 10.1016/j.jval.2014.08.910

PMID: 27200951

233. Eur J Pharm Sci. 2014 Oct 15;63:147-53. doi: 10.1016/j.ejps.2014.07.005. Epub

Excluded b/c no real-world adherence/persistence

2014 Jul 19.

Clinical pharmacology of ponesimod, a selective S1P₁ receptor modulator, after

uptitration to supratherapeutic doses in healthy subjects.

Hoch M(1), D'Ambrosio D(2), Wilbraham D(3), Brossard P(2), Dingemanse J(2).

Author information:

(1)Department of Clinical Pharmacology, Actelion Pharmaceuticals Ltd., Allschwil,

Switzerland. Electronic address: matthias.hoch@actelion.com.

(2)Department of Clinical Pharmacology, Actelion Pharmaceuticals Ltd., Allschwil,

Switzerland.

(3)Quintiles Drug Research Unit at Guy's Hospital, London, United Kingdom.

PURPOSE: The aim of this study was to assess in healthy subjects the safety,

tolerability, pharmacokinetics, and pharmacodynamics of ponesimod, an oral

selective sphingosine-1-phosphate receptor 1 (S1P1) modulator in development for

multiple sclerosis, by using an uptitration scheme up to supratherapeutic doses.

METHODS: This was a double-blind, placebo-controlled, randomised, parallel group,

uptitration study. Male and female subjects received ascending oral doses of

ponesimod (n=12) or placebo (n=4) once daily for 3 days at each dose level

(10-20-40-60-80-100mg).

RESULTS: The most frequent adverse events were chest discomfort, headache,

dizziness, dyspnoea, abdominal pain, and night sweats. Chest discomfort and

dyspnoea were considered dose-limiting. A transient decrease in heart rate was

observed following the first 10-mg ponesimod dose (maximum mean decrease of 9

beats per minute (bpm) (placebo: 2 bpm)). After uptitration, effects on heart

rate were indistinguishable from placebo. A dose-dependent effect on pulmonary

function tests was observed and reached a plateau with 60-80 mg ponesimod

(maximum mean decrease from baseline of 1.24l (-30.5%) in forced expiratory

volume in 1s). A plateau in mean lymphocyte count reduction of approximately 70%

from baseline was reached at the 40 mg dose level. Observed effects were fully

reversible within 10days after treatment discontinuation. No relevant sex

differences were observed.

CONCLUSIONS: At supratherapeutic doses, symptoms of chest discomfort and dyspnoea

were dose-limiting. An uptitration dosing scheme is to be preferred in clinical

studies in patients in order to limit effects of ponesimod on heart rate and

atrioventricular (AV) conduction.

Copyright © 2014. Published by Elsevier B.V.

DOI: 10.1016/j.ejps.2014.07.005

PMID: 25046167 [Indexed for MEDLINE]

234. J Med Econ. 2014 Oct;17(10):696-707. doi: 10.3111/13696998.2014.940422. Epub 2014

Jul 23.

Persistence with and adherence to fingolimod compared with other

disease-modifying therapies for the treatment of multiple sclerosis: a

retrospective US claims database analysis.

Bergvall N(1), Petrilla AA, Karkare SU, Lahoz R, Agashivala N, Pradhan A, Capkun

G, Makin C, McGuiness CB, Korn JR.

Author information:

(1)Novartis Pharma AG , Basel , Switzerland.

OBJECTIVE: Achieving therapeutic goals in multiple sclerosis (MS) requires strict

adherence to treatment schedules. This retrospective study analyzed persistence

with, and adherence to, fingolimod compared with injectable/infusible

disease-modifying therapies (DMTs) in patients with MS.

METHODS: Patients in the PharMetrics Plus™ US administrative claims database with

at least one prescription for, or administration of, fingolimod, glatiramer

acetate (GA), interferon (IFN), or natalizumab (index DMT) between October 1,

2010 and September 30, 2011 were included. Patients were naïve to index DMT (no

claim in the previous 360 days) and had an MS diagnosis code within 360 days of

the first index DMT prescription. Outcomes were persistence, risk of

discontinuing index DMT (evaluated by a Cox proportional hazards model),

adherence (measured using the medication possession ratio [MPR] and proportion of

days covered [PDC] in patients with at least two index DMT prescriptions), and

the risk of being non-adherent (MPR <80% and PDC <80%, assessed using a logistic

regression model).

RESULTS: The study included 3750 patients (fingolimod, n = 889; GA, n = 1233; any

IFN, n = 1341; natalizumab, n = 287). Discontinuation rates (fingolimod, 27.9%;

GA, 39.5%; IFN, 43.7%; natalizumab, 39.5%; all p < 0.001) and risk of

discontinuation were significantly higher (hazard ratios vs fingolimod [95%

confidence interval]: GA, 1.75 [1.49-2.07]; IFN, 2.01 [1.71-2.37]; natalizumab,

1.53 [1.22-1.91]) for patients receiving other DMTs compared with fingolimod. The

risk of being non-adherent was also lower for patients in the fingolimod cohort

than the other treatment cohorts, irrespective of whether non-adherence was

defined as MPR <80% (p < 0.05 for all) or PDC <80% (p < 0.05 for GA and IFN).

LIMITATIONS: As with all studies assessing real-world treatment patterns it is

unclear if medications were used as prescribed.

CONCLUSIONS: In a real-world setting, persistence with, and adherence to, oral

fingolimod was higher than for injectable and infusible DMTs.

DOI: 10.3111/13696998.2014.940422

PMID: 25019581 [Indexed for MEDLINE]

235. Mult Scler. 2014 Oct;20(12):1662-4. doi: 10.1177/1352458514523497. Epub 2014 Feb

Excluded b/c no real-world adherence/persistence

10.

Fingolimod-associated amenorrhea: a report of three cases.

Alroughani R(1).

Author information:

(1)Division of Neurology, Department of Medicine, Amiri Hospital, Sharq, Kuwait

Neurology Clinic, Dasman Diabetes Institute, Kuwait alroughani@gmail.com.

Amenorrhea has not been reported as an adverse event in fingolimod phase III

clinical trials in patients with multiple sclerosis (MS) with either 0.5 mg or

1.25 mg dosages. Here we report three cases of young women with MS who developed

amenorrhea within 6 months of initiation of fingolimod. They experienced

irregularities in their menstrual cycles in the first 3 months, which progressed

to amenorrhea by 5(th) or 6(th) month. Gynecology evaluations showed no other

etiologies. Menses returned to baseline after discontinuation of fingolimod for

2-3 months. Amenorrhea could be associated with fingolimod in the first year.

Future surveillance is advised to determine the incidence rate of this adverse

event.

© The Author(s) 2014.

DOI: 10.1177/1352458514523497

PMID: 24515730 [Indexed for MEDLINE]

236. J Neurol Sci. 2014 Sep 15;344(1-2):80-7. doi: 10.1016/j.jns.2014.06.030. Epub

Excluded b/c no real-world adherence/persistence

2014 Jun 23.

Patient centered decision making: use of conjoint analysis to determine

risk-benefit trade-offs for preference sensitive treatment choices.

Wilson L(1), Loucks A(2), Bui C(3), Gipson G(3), Zhong L(3), Schwartzburg A(4),

Crabtree E(5), Goodin D(6), Waubant E(7), McCulloch C(8).

Author information:

(1)Health Policy and Economics, University of California San Francisco,

Departments of Medicine and Pharmacy, 3333 California Street, San Francisco, CA

94143, USA. Electronic address: wilsonL@pharmacy.ucsf.edu.

(2)University of California San Francisco, Department of Clinical Pharmacy, 3333

California Street, San Francisco, CA 94143, USA. Electronic address:

Aimee.Loucks@gmail.com.

(3)University of California San Francisco, Department of Clinical Pharmacy, 3333

California Street, San Francisco, CA 94143, USA.

(4)University of California San Francisco, Department of Neurology, 1500 Owens

Street, Suite 320, San Francisco, CA 94158, USA. Electronic address:

amy.outdoor@ucsfmedctr.org.

(5)UCSF Multiple Sclerosis Center, 1500 Owens Street, Suite 320, San Francisco,

CA 94158, USA. Electronic address: Liz.Crabtree-Hartman@ucsf.edu.

(6)UCSF Multiple Sclerosis Center, 1500 Owens Street, Suite 320, San Francisco,

CA 94158, USA. Electronic address: Douglas.Goodin@ucsf.edu.

(7)University of California San Francisco, Regional Pediatric MS Center Director,

1500 Owens Street, Suite 320, San Francisco, CA 94158, USA. Electronic address:

Emmanuelle.Waubant@ucsf.edu.

(8)Division of Biostatistics, University of California San Francisco, Department

of Epidemiology and Biostatistics, 185 Berry Street, Suite 5700, Box 0560, San

Francisco, CA 94107-1762, USA. Electronic address: chuck@biostat.ucsf.edu.

Understanding patient preferences facilitates shared decision-making and focuses

on patient-centered outcomes. Little is known about relapsing-remitting multiple

sclerosis (RRMS) patient preferences for disease modifying therapies (DMTs). We

use choice based conjoint (CBC) analysis to calculate patient preferences for

risk/benefit trade-offs for hypothetical DMTs.METHODS: Patients with RRMS were

surveyed between 2012 and 2013. Our CBC survey mimicked the decision-making

process and trade-offs of patients choosing DMTs, based on all possible DMT

attributes. Mixed-effects logistic regression analyzed preferences. We estimated

maximum acceptable risk trade-offs for various DMT benefits.

RESULTS: Severe side-effect risks had the biggest impact on patient preference

with a 1% risk, decreasing patient preference five-fold compared to no risk.

(OR=0.22, p<0.001). Symptom improvement was the most preferred benefit (OR=3.68,

p<0.001), followed by prevention of progression of 10 years (OR=2.4, p<0.001).

Daily oral administration had the third highest DMT preference rating (OR=2.08,

p<0.001). Patients were willing to accept 0.08% severe risk for a year delayed

relapse, and 0.22% for 4 vs 2 year prevented progression.

CONCLUSION: We provided patient preferences and risk-benefit trade-offs for

attributes of all available DMTs. Evaluation of patient preferences is a key step

in shared decision making and may significantly impact early drug initiation and

compliance.

Copyright © 2014 Elsevier B.V. All rights reserved.

DOI: 10.1016/j.jns.2014.06.030

PMID: 25037284 [Indexed for MEDLINE]

237. J Neurol Sci. 2014 Sep 15;344(1-2):193-7. doi: 10.1016/j.jns.2014.06.013. Epub

Excluded b/c no real-world adherence/persistence

2014 Jun 17.

Tumefactive demyelination and a malignant course in an MS patient during and

following fingolimod therapy.

Hellmann MA(1), Lev N(1), Lotan I(1), Mosberg-Galili R(1), Inbar E(2), Luckman

J(2), Fichman-Horn S(3), Yakimov M(3), Steiner I(4).

Author information:

(1)Department of Neurology, Rabin Medical Center, Beilinson Campus, Petach Tikva,

Israel; Sackler Faculty of Medicine, Tel Aviv University, Tel Aviv, Israel.

(2)Department of Radiology, Rabin Medical Center, Beilinson Campus, Petach Tikva,

Israel; Sackler Faculty of Medicine, Tel Aviv University, Tel Aviv, Israel.

(3)Department of Pathology, Rabin Medical Center, Beilinson Campus, Petach Tikva,

Israel; Sackler Faculty of Medicine, Tel Aviv University, Tel Aviv, Israel.

(4)Department of Neurology, Rabin Medical Center, Beilinson Campus, Petach Tikva,

Israel; Sackler Faculty of Medicine, Tel Aviv University, Tel Aviv, Israel.

Electronic address: isteiner@cc.huji.ac.il.

Comment in

J Neurol Sci. 2014 Sep 15;344(1-2):1-2.

Finglimod, a sphingosine 1-phosphate receptor modulator, is the first orally

administered therapy approved for prophylaxis in multiple sclerosis (MS). Several

reports in the last two years suggested that it might be associated with severe

augmentation of disease activity upon initiation or discontinuation of therapy.

We present an MS patient who developed a giant cavitating brain lesion under

fingolimod and in whom cessation of therapy was associated with a very active

course. Brain biopsy revealed the lesion to be due to an active demyelinating

inflammatory process. With the current wave of immunosuppressive treatments for

MS, there is a need to be vigilant to side effects and risks not identified in

large multicenter trials, collect the data and set guidelines and precautions for

present and future medications.

Copyright © 2014 Elsevier B.V. All rights reserved.

DOI: 10.1016/j.jns.2014.06.013

PMID: 25001515 [Indexed for MEDLINE]

238. Age Ageing. 2014 Sep;43(5):592-5. doi: 10.1093/ageing/afu093. Epub 2014 Jul 28.

Excluded b/c no primary data (narrative review)

National Osteoporosis Society vitamin D guideline summary.

Aspray TJ(1), Bowring C(2), Fraser W(3), Gittoes N(4), Javaid MK(5), Macdonald

H(6), Patel S(7), Selby P(8), Tanna N(9), Francis RM(10); National Osteoporosis

Society.

Author information:

(1)The Bone Clinic, Musculoskeletal Unit, Freeman Hospital, Newcastle upon Tyne,

UK Institute for Ageing and Health, Newcastle University, Newcastle upon Tyne,

UK.

(2)National Osteoporosis Society, Bath BA2 0PJ, UK.

(3)Norwich Medical School, University of East Anglia, Norwich NR4 7TJ, UK.

(4)Department of Medicine, University Hospitals Birmingham, Birmingham B15 2TH,

UK.

(5)NIHR BRU, NDORMS, University of Oxford, Nuffield Orthopaedic Centre, OXFORD,

OX3 7HE, UK.

(6)Musculoskeletal Research, University of Aberdeen, Aberdeen AB25 2ZD, UK.

(7)Department of Rheumatology, Epsom & St Helier University Hospital, Carshalton,

Surrey SM5 3AA, UK.

(8)Department of Medicine, Manchester Royal Infirmary, Oxford Road, Manchester,

UK.

(9)Women's Services & Arthritis Centre, N W London Hospitals NHS Trust, Harrow,

Middlesex, UK.

(10)Institute for Ageing and Health, Newcastle University, Newcastle upon Tyne,

UK.

The National Osteoporosis Society (NOS) published its document, Vitamin D and

Bone Health: A Practical Clinical Guideline for Patient Management, in 2013 as a

practical clinical guideline on the management of vitamin D deficiency in adult

patients with, or at risk of developing, bone disease. There has been no clear

consensus in the UK on vitamin D deficiency its assessment and treatment, and

clinical practice is inconsistent. This guideline is aimed at clinicians,

including doctors, nurses and dieticians. It recommends the measurement of serum

25 (OH) vitamin D (25OHD) to estimate vitamin D status in the following clinical

scenarios: bone diseases that may be improved with vitamin D treatment; bone

diseases, prior to specific treatment where correcting vitamin D deficiency is

appropriate; musculoskeletal symptoms that could be attributed to vitamin D

deficiency. The guideline also states that routine vitamin D testing is

unnecessary where vitamin D supplementation with an oral antiresorptive treatment

is already planned and sets the following serum 25OHD thresholds: <30 nmol/l is

deficient; 30-50 nmol/l may be inadequate in some people; >50 nmol/l is

sufficient for almost the whole population. For treatment, oral vitamin D3 is

recommended with fixed loading doses of oral vitamin D3 followed by regular

maintenance therapy when rapid correction of vitamin D deficiency is required,

although loading doses are not necessary where correction of deficiency is less

urgent or when co-prescribing with an oral antiresorptive agent. For monitoring,

serum calcium (adjusted for albumin) should be checked 1 month after completing a

loading regimen, or after starting vitamin D supplementation, in case primary

hyperparathyroidism has been unmasked. However, routine monitoring of serum 25OHD

is generally unnecessary but may be appropriate in patients with symptomatic

vitamin D deficiency or malabsorption and where poor compliance with medication

is suspected. The guideline focuses on bone health as, although there are

numerous putative effects of vitamin D on immunity modulation, cancer prevention

and the risks of cardiovascular disease and multiple sclerosis, there remains

considerable debate about the evaluation of extraskeletal factors and optimal

vitamin D status in these circumstances.

© The Author 2014. Published by Oxford University Press on behalf of the British

Geriatrics Society. All rights reserved. For Permissions, please email:

journals.permissions@oup.com.

DOI: 10.1093/ageing/afu093

PMID: 25074538 [Indexed for MEDLINE]

239. CNS Drugs. 2014 Sep;28(9):817-24. doi: 10.1007/s40263-014-0185-z.

Real-world use of fingolimod in patients with relapsing remitting multiple

sclerosis: a retrospective study using the national multiple sclerosis registry

in Kuwait.

Al-Hashel J(1), Ahmed SF, Behbehani R, Alroughani R.

Author information:

(1)Department of Neurology, Ibn Sina Hospital, P.O. Box 25427, 13115, Safat,

Kuwait.

BACKGROUND: Fingolimod is an oral sphingosine-1-phosphate-receptor modulator,

which has demonstrated efficacy in clinical trials and has recently been approved

for multiple sclerosis (MS) treatment in Kuwait. Post-marketing studies are

important to demonstrate real-life efficacy and safety.

OBJECTIVE: The objective of this study was to examine the efficacy and safety of

fingolimod treatment in a clinical setting.

METHODS: Using the national Kuwait MS registry, relapsing remitting MS patients

who had been prescribed fingolimod for ≥6 months were retrospectively identified.

Three-monthly clinical evaluations and 6-monthly magnetic resonance imagings

(MRIs) were performed. Patient status pre- and post-treatment was compared using

chi-square and Student t-tests.

RESULTS: A total of 175 patients were included: 75.4 % female (n = 132); mean age

33.3 ± 9.2 years; mean disease duration 7.2 ± 5.2 years; mean fingolimod use

21.7 ± 9.1 months. Most had used previous disease-modifying therapy (78.9 %;

n = 138), mainly interferons (66.9 %; n = 117). Twenty-three patients (11.4 %)

discontinued/withdrew fingolimod; of whom eight had relapses. The proportion of

relapse-free patients improved significantly (86.3 % vs. 32.6 %; p < 0.001),

while the proportion of patients with MRI activity decreased (18.3.6 % vs.

77.7 %; p < 0.001). Mean expanded disability status scale (EDSS) score at the

last visit improved when compared with pre-treatment (2.26 ± 1.49 vs.

2.60 ± 1.44; p = 0.03). Forty-three (24.6 %) patients experienced adverse events;

headaches and lymphopenia were the most commonly reported adverse events.

CONCLUSION: Fingolimod treatment was associated with reduced relapse and MRI

activity, and an improved EDSS score. Discontinuation/withdrawal rates and

adverse events were low. Fingolimod presents a promising treatment for MS in

Kuwait.

DOI: 10.1007/s40263-014-0185-z

PMID: 25011422 [Indexed for MEDLINE]

Excluded b/c no primary data (narrative review)

240. Expert Rev Neurother. 2014 Sep;14(9):1029-42. doi: 10.1586/14737175.2014.945523.

Epub 2014 Aug 11.

Fostering adherence to injectable disease-modifying therapies in multiple

sclerosis.

Lugaresi A(1), Rottoli MR, Patti F.

Author information:

(1)Department of Neuroscience, Imaging and Clinical Sciences, Multiple Sclerosis

Center, University "G. d'Annunzio", Ospedale Clinicizzato "SS Annunziata", VII

livello, Corpo A, Via dei Vestini snc, 66100 Chieti, Italy.

Multiple sclerosis requires long-term management, often with disease-modifying

therapies. Poor medication adherence, especially to injectables, can increase

relapse and hospitalisation rates and consume healthcare resources. We discuss

adherence definitions and terminology and its prevalence in multiple sclerosis

(MS). Typical causes of poor adherence in patients with MS include: insufficient

efficacy or tolerability, concurrent disorders, and consequences of MS (e.g.,

forgetfulness, depression, fatigue and poor motor skills). Ways to improve

adherence rates are reviewed, focusing on interdisciplinary healthcare teams,

good communication between healthcare workers and patients (and their families),

ongoing support and digital tools to promote adherence. We consider open

communication and continuing education to be key, and that MS nurses have a

pivotal role in ensuring patients' adherence to MS medicines.

DOI: 10.1586/14737175.2014.945523

PMID: 25109614 [Indexed for MEDLINE]

241. Int J Neurosci. 2014 Sep;124(9):652-6. doi: 10.3109/00207454.2013.877460. Epub

Excluded b/c no real-world adherence/persistence

2014 Jan 23.

Clinical experience with THC:CBD oromucosal spray in patients with multiple

sclerosis-related spasticity.

Koehler J(1), Feneberg W, Meier M, Pöllmann W.

Author information:

(1)Marianne Strauss Klinik , Berg , Germany.

This detailed medical charts' data collection study conducted at a multiple

sclerosis (MS) clinic in Germany evaluated the effectiveness of

tetrahydrocannabinol (THC)/cannabidiol (CBD) oromucosal spray in patients with

resistant MS spasticity. Over a 15-month timeframe, THC:CBD spray was initiated

in 166 patients. Mean follow-up was 9 months. In all, 120 patients remained on

treatment for a response rate of 72%. THC:CBD spray was used as add-on therapy in

95 patients and as monotherapy in 25 patients to achieve best-possible

therapeutic results. Among responders, the mean spasticity 0-10 numerical rating

scale (NRS) score decreased by 57%, from 7.0 before treatment to 3.0 within 10

days of starting THC:CBD spray. The mean dosage was 4 sprays/day. Most patients

who withdrew from treatment (40/46) had been receiving THC:CBD spray for less

than 60 days. Main reasons for treatment discontinuation were: adverse drug

reactions, mainly dizziness, fatigue and oral discomfort (23 patients; 13.9%);

lack of efficacy (14 patients; 8.4%); or need for a baclofen pump (9 patients;

5.4%). No new safety signals were noted with THC:CBD spray during the evaluation

period. In this routine clinical practice setting at an MS clinic in Germany,

THC:CBD spray was effective and well tolerated as add-on therapy or as

monotherapy in a relevant proportion of patients with resistant MS spasticity.

DOI: 10.3109/00207454.2013.877460

PMID: 24392812 [Indexed for MEDLINE]

242. Mult Scler Relat Disord. 2014 Sep;3(5):620-8. doi: 10.1016/j.msard.2014.06.006.

Excluded b/c no real-world adherence/persistence

Epub 2014 Jul 3.

First-dose effects of fingolimod after switching from injectable therapies in the

randomized, open-label, multicenter, Evaluate Patient OutComes (EPOC) study in

relapsing multiple sclerosis.

Hughes B(1), Cascione M(2), Freedman MS(3), Agius M(4), Kantor D(5), Gudesblatt

M(6), Goldstick LP(7), Agashivala N(8), Schofield L(9), McCague K(10), Hashmonay

R(11), Barbato L(12); EPOC study investigators.

Author information:

(1)Mercy Ruan Neuroscience Center, Des Moines, IA, USA. Electronic address:

brucehughes@mac.com.

(2)Tampa Neurology Associates, Tampa, FL, USA. Electronic address:

me@markcascione.com.

(3)University of Ottawa and the Ottawa Hospital Research Institute, Ottawa, ON,

Canada. Electronic address: MFREEDMAN@Ottawahospital.on.ca.

(4)University of California Davis, Veterans Affairs Northern California Health

Care System (VANCHCS), CA, USA. Electronic address: markaagius@hotmail.com.

(5)Neurologique Foundation, Inc., Jacksonville, FL, USA. Electronic address:

dkantor@neurologique.org.

(6)Multiple Sclerosis Comprehensive Care Center, St Islip, NY, USA. Electronic

address: mgudesbl@southshoreneurologic.com.

(7)Neurology Specialists Inc, Dayton, OH, USA. Electronic address:

lgoldstick@hotmail.com.

(8)Novartis Pharmaceuticals Corporation, East Hanover, NJ, USA. Electronic

address: neetu.agashivala@novartis.com.

(9)Novartis Pharmaceuticals Corporation, East Hanover, NJ, USA. Electronic

address: lesley.schofield@novartis.com.

(10)Novartis Pharmaceuticals Corporation, East Hanover, NJ, USA. Electronic

address: kevin.mccague@novartis.com.

(11)Novartis Pharmaceuticals Corporation, East Hanover, NJ, USA. Electronic

address: ronny.hashmonay@novartis.com.

(12)Novartis Pharmaceuticals Corporation, East Hanover, NJ, USA. Electronic

address: lou.barbato@novartis.com.

BACKGROUND: In pivotal phase 3 studies, fingolimod treatment initiation was

associated with a transient reduction in heart rate (HR). Atrioventricular (AV)

conduction delays, which were typically asymptomatic, were detected in a small

minority of patients.

OBJECTIVE: We report the first-dose effects of fingolimod in patients who

switched from injectable therapies during the Evaluate Patient OutComes (EPOC)

study (ClinicalTrials.gov Identifier: NCT01216072).

METHODS: This was a phase 4, 6-month, randomized, active-comparator, open-label,

multicenter study. It included over 900 fingolimod-treated patients with

relapsing multiple sclerosis, with subgroups of individuals who were receiving

common concomitant HR-lowering medications or had pre-existing cardiac conditions

(PCCs). Vital signs were recorded hourly for 6h post-dose. A 12-lead

electrocardiogram was obtained at baseline and at 6h post-dose.

RESULTS: A transient decrease in mean HR and blood pressure occurred within 6h of

the first fingolimod dose. The incidence of symptomatic bradycardia was low (1%);

eight patients reported dizziness and there was one case each of fatigue,

palpitations, dyspnea, cardiac discomfort, and gait disturbance. These

symptomatic events were typically mild or moderate in severity and all resolved

spontaneously, without intervention or fingolimod discontinuation.

CONCLUSION: First-dose effects in patients with PCCs and in those receiving

concomitant HR-lowering medications were consistent with effects observed in the

overall study population and with results from previous clinical trials. The EPOC

study provides additional data demonstrating the transient and generally benign

nature of fingolimod first-dose effects on HR and AV conduction in a large

population that is more representative of patients encountered in routine

clinical practice than in the pivotal trials.

Copyright © 2014 Elsevier B.V. All rights reserved.

DOI: 10.1016/j.msard.2014.06.006

PMID: 26265274 [Indexed for MEDLINE]

243. Neurol Sci. 2014 Sep;35(9):1485-6. doi: 10.1007/s10072-014-1800-y. Epub 2014 Apr

Excluded b/c no real-world adherence/persistence

23.

Multiple sclerosis rebound after fingolimod discontinuation for lymphopenia.

La Mantia L(1), Prone V, Marazzi MR, Erminio C, Protti A.

Author information:

(1)Department of Clinical Neurology and Stroke Unit, Multiple Sclerosis Center,

Niguarda Ca' Granda Hospital, Piazza Ospedale Maggiore 3, 20162, Milan, Italy,

lamantialore@gmail.com.

DOI: 10.1007/s10072-014-1800-y

PMID: 24756193 [Indexed for MEDLINE]

244. Ther Adv Neurol Disord. 2014 Sep;7(5):232-8. doi: 10.1177/1756285614544466.

Excluded b/c no real-world adherence/persistence

Fumarate treatment in progressive forms of multiple sclerosis: first results of a

single-center observational study.

Strassburger-Krogias K(1), Ellrichmann G(1), Krogias C(1), Altmeyer P(2), Chan

A(1), Gold R(3).

Author information:

(1)Department of Neurology, Ruhr University Bochum, St Josef-Hospital, Bochum,

Germany.

(2)Department of Dermatology, Ruhr University Bochum, St Josef-Hospital, Bochum,

Germany.

(3)Department of Neurology, Ruhr University Bochum, St Josef-Hospital,

Gudrunstraße 56, 44791 Bochum, Germany.

OBJECTIVES: Therapeutic options in progressive forms of multiple sclerosis (MS)

are still limited. Dimethyl fumarate (DMF) has immunomodulatory properties but

may also exert antioxidative cytoprotective effects. Hence, it may be a

therapeutic option for progressive MS. The aim of this observational study was to

evaluate safety, adherence and efficacy of fumarates in patients with primary

progressive MS (PPMS) or secondary progressive MS.

METHODS: Patients with progressive MS whose condition had failed to respond to

standard therapies and had worsened received the fumarate mixture Fumaderm,

licensed for psoriasis therapy in Germany, or DMF by pharmaceutical preparation

(Bochum ethics approval no. 4797-13). At regular follow-up visits, tolerability

and disease course were assessed.

RESULTS: Twenty-six patients [age 54 ± 7.8 years; female = 13 (50%); PPMS = 12

(46.2%); Expanded Disability Status Scale (EDSS) = 6.0 ± 0.4 (range 3.5-8.0);

disease duration = 14.1 ± 8.7 years] were initiated on treatment with Fumaderm (n

= 18) or pharmacy-prepared DMF (n=8). During a mean follow-up period of 13.2 ±

7.5 months (range 6-30) only five patients (19.2%) reported minor complaints. In

15 patients (57.7%) EDSS remained stable. In five cases (19.2%) there was even a

decrease in EDSS while in six patients (23.1%) there was an increase in EDSS of

more than 0.5 points, reflecting deterioration. Laboratory values were controlled

for lymphopenia, renal and hepatic values, without any safety problems. We

observed no significant differences between the two pharmaceutical forms.

CONCLUSION: Our pilot data indicate that fumarate therapy appears to be safe and

well tolerated by patients with progressive MS. In more than 75% of cases no

further disease progression was evident. However, controlled studies are

warranted to evaluate the detailed therapeutic potential of fumarates and their

long-term effects in progressive MS.

DOI: 10.1177/1756285614544466

PMCID: PMC4206620

PMID: 25342977

245. Patient Prefer Adherence. 2014 Aug 21;8:1123-34. doi: 10.2147/PPA.S68698.

Excluded b/c no primary data (narrative review)

eCollection 2014.

Two decades of subcutaneous glatiramer acetate injection: current role of the

standard dose, and new high-dose low-frequency glatiramer acetate in

relapsing-remitting multiple sclerosis treatment.

Caporro M(1), Disanto G(1), Gobbi C(1), Zecca C(1).

Author information:

(1)Neurocenter of Southern Switzerland, Ospedale Regionale di Lugano, Lugano,

Switzerland.

Glatiramer acetate, a synthetic amino acid polymer analog of myelin basic

protein, is one of the first approved drugs for the treatment of

relapsing-remitting multiple sclerosis. Several clinical trials have shown

consistent and sustained efficacy of glatiramer acetate 20 mg subcutaneously

daily in reducing relapses and new demyelinating lesions on magnetic resonance

imaging in patients with relapsing-remitting multiple sclerosis, as well as

comparable efficacy to high-dose interferon beta. Some preclinical and clinical

data suggest a neuroprotective role for glatiramer acetate in multiple sclerosis.

Glatiramer acetate is associated with a relatively favorable side-effect profile,

and importantly this was confirmed also during long-term use. Glatiramer acetate

is the only multiple sclerosis treatment compound that has gained the US Food and

Drug Administration pregnancy category B. All these data support its current use

as a first-line treatment option for patients with clinical isolated syndrome or

relapsing-remitting multiple sclerosis. More recent data have shown that

high-dose glatiramer acetate (ie, 40 mg) given three times weekly is effective,

safe, and well tolerated in the treatment of relapsing-remitting multiple

sclerosis, prompting the approval of this dosage in the US in early 2014. This

high-dose, lower-frequency glatiramer acetate might represent a new, more

convenient regimen of administration, and this might enhance patients' adherence

to the treatment, crucial for optimal disease control.

DOI: 10.2147/PPA.S68698

PMCID: PMC4144933

PMID: 25170258

246. Patient Prefer Adherence. 2014 Aug 19;8:1093-9. doi: 10.2147/PPA.S67334.

Excluded b/c no primary data (narrative review)

eCollection 2014.

Managing treatment fatigue in patients with multiple sclerosis on long-term

therapy: the role of multiple sclerosis nurses.

Crawford A(1), Jewell S(1), Mara H(1), McCatty L(1), Pelfrey R(1).

Author information:

(1)The Lash Group; Frisco, TX, USA.

This article discusses the many ways that nurses can address the factors that

lead to treatment fatigue in patients with multiple sclerosis (MS) on long-term

disease-modifying therapy, ultimately helping to preserve the patient's health

and quality of life. Patients with MS on long-term therapy may suffer from

treatment fatigue and poor adherence due to a variety of different factors,

including difficulties with injections, anxiety/depression, financial problems,

and inaccurate beliefs about the MS disease process. Because MS nurses have

regular interactions with patients, they are ideally situated to help patients

cope with these and other factors that may limit adherence.

DOI: 10.2147/PPA.S67334

PMCID: PMC4144841

PMID: 25170254

247. Curr Med Res Opin. 2014 Aug;30(8):1461-71. doi: 10.1185/03007995.2014.915802.

Epub 2014 May 2.

Excluded b/c no real-world adherence/persistence

Healthcare resource use and relapses with fingolimod versus natalizumab for

treating multiple sclerosis: a retrospective US claims database analysis.

Bergvall N(1), Lahoz R, Reynolds T, Korn JR.

Author information:

(1)Novartis Pharma AG , Basel , Switzerland.

OBJECTIVE: Healthcare resource utilization in patients with multiple sclerosis

(MS) is linked to relapses and disease progression. This retrospective cohort

database analysis compared healthcare resource use and proxy measures of relapse

outcomes in patients with active disease who switched to fingolimod or

natalizumab.

METHODS: Using administrative claims data from the US PharMetrics Plus database,

we identified patients with an MS diagnosis and a claim for fingolimod or

natalizumab between 1 October 2010 and 30 June 2012 (index period) who had

experienced a relapse (identified using a claims-based algorithm) and used other

disease-modifying therapies (DMTs) in the previous year. Patients in the

fingolimod and natalizumab cohorts were propensity score matched (1:1).

MS-related inpatient stays, corticosteroid use and the proportion of patients

experiencing claims-based relapses were assessed in the pre-index and post-index

persistence periods. Time to first claims-based relapse in the post-index

persistence period was assessed using a Kaplan-Meier curve.

RESULTS: The study included 623 unmatched patients (299 and 324 patients in the

fingolimod and natalizumab cohorts, respectively) and 370 matched patients (185

in each cohort). In the matched analysis, MS-related inpatient stays and

corticosteroid use were similar in the fingolimod and natalizumab cohorts during

the post-index persistence period, and were significantly reduced versus the

pre-index period (p < 0.01). A similar proportion of patients in the fingolimod

and natalizumab cohorts were free from claims-based relapses in the persistence

period (68.1% and 68.6%, respectively). There was no significant difference in

the likelihood of experiencing a claims-based relapse (p = 0.8696).

LIMITATION: Identification of relapses is based on database claims rather than on

clinical assessment.

CONCLUSIONS: In analyses of patients with MS with a history of relapse and DMT

use, fingolimod and natalizumab reduce healthcare resource utilization and have

similar effectiveness in a real-world setting.

DOI: 10.1185/03007995.2014.915802

PMID: 24754349 [Indexed for MEDLINE]

Excluded b/c no oral DMD results

248. J Manag Care Spec Pharm. 2014 Aug;20(8):800-7.

Use of an early disease-modifying drug adherence measure to predict future

adherence in patients with multiple sclerosis.

Kozma CM(1), Phillips AL, Meletiche DM.

Author information:

(1)CK Consulting Associates, LLC, 84 Tomahawk Trail, Saint Helena Island, SC

29920. ckozma@c-k-consulting.com.

BACKGROUND: Patients with multiple sclerosis (MS) who are adherent to their

treatment regimens are less likely to experience relapses and the cost associated

with relapse. Pharmacists whose practice involves these specialty pharmaceuticals

used to treat MS are striving for ways to improve outcomes by achieving treatment

adherence in their patients. Specialty pharmacies have reported higher adherence

rates than traditional pharmacies, which may translate to improved outcomes.

Identifying patients who warrant increased adherence intervention is critical.

Models using administrative health care claims to predict adherence have

typically included demographic characteristics, comorbidities, and/or previous

consumption of health care resources. Addition of a measure of early adherence

may improve the ability to predict future adherence outcomes.

OBJECTIVE: To evaluate early adherence with disease-modifying drugs (DMDs) as a

predictor of future adherence in patients with MS.

METHODS: The first DMD claim (i.e., index event) for adult MS patients (aged ≥18

years and aged ≤ 65 years) who received self-injected DMDs between January 1,

2006, and May 31, 2010, was identified in a national U.S. managed care database.

Patients were required to have continuous eligibility for 12 months pre- and 24

months post-index. Multiple regression models were used to predict future

adherence as measured by the proportion of days covered (PDC). The base model

included age, gender, a medication intensity measure, presence of a

non-MS-related hospitalization pre-index, and markers for physical difficulty,

forgetfulness, or depression/stress. Models adding early DMD adherence as a

covariate were analyzed using incrementing 30-day periods predicting the

subsequent 360 days.

RESULTS: There were 4,606 patients included with an average age of 46.0 (SD 9.4)

years, and 78.7% were female. Average PDC in the first 360 days post-index was

80.0% (SD 26.0). Using the first 60 days of early adherence as the only predictor

in the model showed an R2 of 20.6%. The base model (i.e., no early adherence

measure but other covariates included) yielded an adjusted R2 of only 2.3%. As

the time period of early adherence is increased (from 60 to 360 days), the

explained variance as measured by adjusted R2 values increased from 20.6% to

53.5% (early adherence-only models). Addition of the covariates, other than early

adherence, increased the R2 by 1% to 2%.

CONCLUSIONS: Statistical predictive models that include early adherence with DMDs

were able to explain the variance in future adherence outcomes to a greater

extent than models based solely on baseline characteristics. The efficiency of an

adherence intervention in reaching its intended target can be improved by using

models such as these with enhanced specificity and selectivity.

DOI: 10.18553/jmcp.2014.20.8.800

PMID: 25062073 [Indexed for MEDLINE]

Excluded b/c no real-world adherence/persistence

249. JAMA Neurol. 2014 Aug;71(8):954-60. doi: 10.1001/jamaneurol.2014.1200.

Treatment of relapsing-remitting multiple sclerosis after 24 doses of

natalizumab: evidence from an Italian spontaneous, prospective, and observational

study (the TY-STOP Study).

Clerico M(1), Schiavetti I(2), De Mercanti SF(1), Piazza F(1), Gned D(3), Brescia

Morra V(4), Lanzillo R(4), Ghezzi A(5), Bianchi A(5), Salemi G(6), Realmuto S(6),

Sola P(7), Vitetta F(7), Cavalla P(8), Paolicelli D(9), Trojano M(9), Sormani

MP(2), Durelli L(1).

Author information:

(1)Division of Neurology, Department of Clinical and Biological Sciences,

University of Turin, San Luigi Gonzaga University Hospital, Orbassano, Italy.

(2)Biostatistics Unit, Department of Health Sciences, University of Genoa, Genoa,

Italy.

(3)Department of Oncology, University of Turin, San Luigi Gonzaga University

Hospital, Orbassano, Italy.

(4)Department of Neurosciences, Reproductive, and Odontostomatological Sciences,

Federico II University of Naples, Naples, Italy.

(5)Department of Neurology II, Center of Multiple Sclerosis, Azienda Ospedaliera

Sant'Antonio Abate Hospital, Gallarate, Italy.

(6)Section of Neurology, Department of Experimental Biomedicine and Clinical

Neuroscience, University of Palermo, Palermo, Italy.

(7)Neurology Unit, Department of Neurosciences, University of Modena and Reggio

Emilia, Modena, Italy.

(8)Division of Neurology and Multiple Sclerosis Center, Department of

Neurosciences, University of Turin, Città della Salute e della Scienza University

Hospital, Turin, Italy.

(9)Department of Neuroscience and Sense Organs, University of Bari, Bari, Italy.

IMPORTANCE: The evaluation of therapeutic choices is needed after 24 doses of

natalizumab in patients with multiple sclerosis (MS).

OBJECTIVE: To evaluate the effect of therapeutic choices on the mean annualized

relapse rate and on magnetic resonance imaging MS activity after 24 doses of

natalizumab in patients with relapsing-remitting MS.

DESIGN, SETTING, AND PARTICIPANTS: The TY-STOP study, which recruited

participants between October 22, 2010, and October 22, 2012, at 8 Italian MS

centers (secondary care outpatient clinics) among 124 adult patients who

demonstrated no clinical or magnetic resonance imaging MS activity after 24 doses

of natalizumab.

INTERVENTIONS: Natalizumab, no treatment, interferon beta, glatiramer acetate, or

fingolimod.

MAIN OUTCOMES AND MEASURES: The primary end point was the mean annualized relapse

rate. Statistical analyses were performed in 124 patients with complete follow-up

data among 130 patients who were recruited and stratified into study groups. In

the intent-to-treat group, the decision was made to continue or interrupt

natalizumab after 24 doses. In the as-treated group, natalizumab continuers

received natalizumab, natalizumab switchers changed to different therapies, and

natalizumab quitters discontinued natalizumab during the study year.

RESULTS: No significant differences in demographic or baseline clinical

characteristics were found among the study participants. In the intent-to-treat

group (n = 124), clinical (P = .004) and radiologic (P = .02) MS activity was

significantly lower in patients continuing natalizumab (n = 43) than in patients

interrupting natalizumab (n = 81), with a protective effect of natalizumab

continuation on both outcomes (odds ratio [OR], 0.33; 95% CI, 0.15-0.70 for

clinical activity and OR, 0.35; 95% CI, 0.15-0.79 for radiologic activity). In

the as-treated group (n = 124), clinical (P = .003) and radiologic (P = .03) MS

activity was significantly lower in natalizumab continuers than in natalizumab

switchers or quitters, confirming a protective effect of natalizumab on the risk

of relapse in natalizumab continuers compared with natalizumab quitters (OR,

4.40; 95% CI, 1.72-11.23) and natalizumab switchers (OR, 3.28; 95% CI,

0.99-10.79). No disease rebound was observed in natalizumab quitters. After

natalizumab discontinuation, 1 patient developed progressive multifocal

leukoencephalopathy during the observation period, with complete recovery.

CONCLUSIONS AND RELEVANCE: This study provides class III evidence of an increased

risk of MS activity resumption after natalizumab discontinuation. Therapy

discontinuation after 24 doses in natalizumab-responding patients should be

considered only if the risk of progressive multifocal leukoencephalopathy is high

and outweighs the benefits of continuing the drug.

TRIAL REGISTRATION: Osservatorio Nazionale Sulla Sperimentazione Clinica dei

Medicinali No. 131/2010.

DOI: 10.1001/jamaneurol.2014.1200

PMID: 24977406 [Indexed for MEDLINE]

250. Patient Prefer Adherence. 2014 Jul 14;8:987-9. doi: 10.2147/PPA.S65708.

Excluded b/c no real-world adherence/persistence

eCollection 2014.

Life-threatening asthma attack during prolonged fingolimod treatment: case

report.

Zecca C(1), Caporro M(1), Györik S(2), Gobbi C(1).

Author information:

(1)Neurocenter of Southern Switzerland, Department of Neurology, Ospedale

Regionale di Lugano, Lugano, Switzerland.

(2)Department of Internal Medicine, Ospedale Regionale di Bellinzona, Bellinzona,

Switzerland.

BACKGROUND: Fingolimod (FTY) mediates bronchoconstriction by interacting with

sphingosine-1-phosphate receptors. The majority of the reported adverse

respiratory events occur during the first weeks of treatment.

CASE PRESENTATION: A 49-year-old woman developed a life-threatening asthma attack

after 6 months of continuous FTY treatment. The adverse event required prolonged

hospitalization, and the patient recovered without sequelae after FTY

interruption. A history of previous airway hyperreactivity and a concurrent viral

respiratory infection possibly acted as predisposing factors.

CONCLUSION: This first description of a severe, life-threatening asthma attack

during prolonged FTY treatment suggests the need for long-term clinical

surveillance, especially in patients with known predisposing factors.

DOI: 10.2147/PPA.S65708

PMCID: PMC4105229

PMID: 25053881

251. Iran J Neurol. 2014 Jul 4;13(3):119-26.

Excluded b/c no primary data (narrative review)

Cardiovascular effects of fingolimod: A review article.

Behjati M(1), Etemadifar M(2), Abdar Esfahani M(3).

Author information:

(1)Department of Cardiology, School of Medicine, Cardiovascular Research Center

AND Heart Failure Research Center, Isfahan Cardiovascular Research Institute,

Isfahan University of Medical Sciences, Isfahan, Iran.

(2)Department of Neurology, School of Medicine, Isfahan University of Medical

Sciences, Isfahan, Iran.

(3)Department of Cardiology, School of Medicine, Isfahan University of Medical

Sciences, Isfahan, Iran.

Multiple sclerosis (MS) is a chronic auto-immune disease. Most therapeutic

strategies for treatment of this disease direct immune modulation and control of

inflammatory processes. First-line therapeutic agents showed moderate efficacy

and frequent side-effects with moderate efficacy in trials. Their parental

administration and limited long-term adherence restrict their efficacy compared

with second-line therapies. Fingolimod as a second-line therapeutic agent has

been shown to reduce annualized relapse rate, risk of disability progression and

inflammatory activity of relapsing MS. Safety and efficacy FTY720: Safety and

efficacy issues are the main metrics for judgment of drug efficacy. In this

article, we focus on cardiovascular effects of FTY720 treatment. Effect of FTY720

on rate and rhythm, impact of FTY720 on endothelial cells, its atheroprotective

effects, its effects on cardiac transplantation outcomes, vascular complications

of FTY720, effects of FTY720 on endocrine functions and interaction of FTY720

with cardioactive agents are explained in this review article.

PMCID: PMC4240927

PMID: 25422729

252. Patient Prefer Adherence. 2014 Jul 4;8:971-9. doi: 10.2147/PPA.S49903.

Excluded b/c no real-world adherence/persistence

eCollection 2014.

Patient perspectives on switching disease-modifying therapies in the NARCOMS

registry.

Salter AR(1), Marrie RA(2), Agashivala N(3), Belletti DA(3), Kim E(3), Cutter

GR(1), Cofield SS(1), Tyry T(4).

Author information:

(1)Department of Biostatistics, University of Alabama at Birmingham, Birmingham,

AL, USA.

(2)Department of Internal Medicine, University of Manitoba, Winnipeg, MB, Canada

; Department of Community Health Sciences, University of Manitoba, Winnipeg, MB,

Canada.

(3)Novartis Pharmaceutical Corporation, East Hanover, NJ, USA.

(4)Division of Neurology, St. Joseph's Hospital and Medical Center, Phoenix, AZ,

USA.

INTRODUCTION: The evolving landscape of disease-modifying therapies (DMTs) for

multiple sclerosis raises important questions about why patients change DMTs.

Physicians and patients could benefit from a better understanding of the reasons

for switching therapy.

PURPOSE: To investigate the reasons patients switch DMTs and identify

characteristics associated with the decision to switch.

METHOD: The North American Research Committee on Multiple Sclerosis (NARCOMS)

Registry conducted a supplemental survey among registry participants responding

to the 2011 update survey. The supplemental survey investigated reasons for

switching DMT, origin of the discussion of DMT change, and which factors

influenced the decision. Chi-square tests, Fisher's exact tests, and logistic

regression were used for the analyses.

RESULTS: Of the 691 eligible candidates, 308 responded and met the inclusion

criteria (relapsing disease course, switched DMT after September 2010). The

responders were 83.4% female, on average 52 years old, with a median

(interquartile range) Patient-Determined Disease Steps score of 4 (2-5). The most

recent prior therapy included first-line injectables (74.5%), infusions (18.1%),

an oral DMT (3.4%), and other DMTs (4.0%). The discussion to switch DMT was

initiated almost equally by physicians and participants. The primary reason for

choosing the new DMT was based most frequently on physician's recommendation

(24.5%) and patient perception of efficacy (13.7%).

CONCLUSION: Participants frequently initiated the discussion regarding changing

DMT, although physician recommendations regarding the specific therapy were still

weighed highly. Long-term follow-up of these participants will provide valuable

information on their disease trajectory, satisfaction with, and effectiveness of

their new medication.

DOI: 10.2147/PPA.S49903

PMCID: PMC4094626

PMID: 25045254

Excluded b/c no real-world adherence/persistence

253. Int J MS Care. 2014 Summer;16(2):68-75. doi: 10.7224/1537-2073.2013-021.

Treatment satisfaction in multiple sclerosis.

Glanz BI(1), Musallam A(1), Rintell DJ(1), Chitnis T(1), Weiner HL(1), Healy

BC(1).

Author information:

(1)Department of Neurology, Harvard Medical School, Boston, MA, USA (BIG, DJR,

TC, HLW, BCH); Partners Multiple Sclerosis Center, Brigham and Women's Hospital,

Boston, MA, USA (BIG, AM, DJR, TC, HLW, BCH); and Biostatistics Center,

Massachusetts General Hospital, Boston, MA, USA (BCH).

BACKGROUND: Disease-modifying therapies (DMTs) for the treatment of multiple

sclerosis (MS) are associated with inconvenient methods of administration,

significant side effects, and low adherence rates. This study was undertaken to

compare treatment satisfaction in MS patients treated with interferon beta-1a

intramuscular (IFNβ-1a IM), interferon beta-1a subcutaneous (IFNβ-1a SC),

glatiramer acetate (GA), and natalizumab (NTZ), and to examine the associations

between treatment satisfaction ratings and adherence to therapy.

METHODS: Two hundred twenty-six treated MS patients completed the Treatment

Satisfaction Questionnaire for Medicine. Multivariable models were used to

compare treatment satisfaction across groups.

RESULTS: There were no statistically significant differences in overall treatment

satisfaction. The NTZ group reported greater satisfaction with the ability of the

medication to treat or prevent MS than the IFNβ-1a IM group. The NTZ group also

reported higher overall convenience scores than the IFNβ-1a IM group and greater

satisfaction with ease of use of the medication than the interferon and GA

groups. Patients in the IFNβ-1a IM group reported less satisfaction with ease of

planning when to use the medication than those in the other groups. Convenience

was associated with adherence in IFNβ-1a SC- and GA-treated patients, with lower

convenience scores associated with lower adherence.

CONCLUSIONS: These results may be useful to MS patients and health-care providers

facing decisions about DMT use.

DOI: 10.7224/1537-2073.2013-021

PMCID: PMC4106393

PMID: 25061430

Excluded b/c no real-world adherence/persistence

254. Mult Scler Relat Disord. 2014 Jul;3(4):520-6. doi: 10.1016/j.msard.2014.04.003.

Epub 2014 Apr 13.

Long-term management of natalizumab discontinuation in a large monocentric cohort

of multiple sclerosis patients.

Sangalli F(1), Moiola L(2), Ferrè L(2), Radaelli M(2), Barcella V(2), Rodegher

M(2), Colombo B(2), Martinelli Boneschi F(2), Martinelli V(2), Comi G(2).

Author information:

(1)Department of Neurology, San Raffaele Scientific Institute, Via Olgettina 48,

20132 Milan, Italy. Electronic address: sangalli.francesca@hsr.it.

(2)Department of Neurology, San Raffaele Scientific Institute, Via Olgettina 48,

20132 Milan, Italy.

BACKGROUND: Pivotal and post-marketing studies demonstrated the impressive

efficacy and the good tolerability profile of natalizumab in Multiple Sclerosis

patients. On the other hand long-term safety of natalizumab therapy is burdened

by the risk of progressive multifocal leukoencephalopathy, especially in anti-JCV

seropositive patients treated for more than two years. Some of these patients

must stop the drug at the risk of disease reactivation.

OBJECTIVES: To evaluate the effects of natalizumab discontinuation in a

monocentric cohort of multiple sclerosis patients followed for a mean time of

22.4 months.

METHODS: One hundred and ten patients, who stopped therapy after at least 12

infusions, were followed with periodic clinical and magnetic resonance imaging

evaluations. One hundred patients started either immunomodulant therapy (n=90) or

fingolimod (n=10) while 10 remained without any drug.

RESULTS: "Disease-activity free" patients were 25% at one year after

discontinuation and annualized relapse rate significantly increased from 0.06 to

0.84 (p<0.0001). We found that the risk of reactivation peaked despite

alternative treatments between the second and the eighth month after suspension,

a so-called "high risk period". During this period the majority of patients

showed a return to pre-natalizumab disease activity while 10% of patients

presented a "rebound activity". A higher pre-natalizumab disease activity was

correlated with an increased risk of reactivation (p=0.004).

CONCLUSIONS: Our data suggest that disease reactivation peaked during a "high

risk period" between the second and the eighth month since stopping the drug.

During this period no alternative treatments seemed to provide an adequate

protection from disease reactivation. Though transient, this phase could be

potentially dangerous, therefore we need to develop more effective strategies to

deal with this challenge.

Copyright © 2014 Elsevier B.V. All rights reserved.

DOI: 10.1016/j.msard.2014.04.003

PMID: 25877065

Excluded b/c no primary data (narrative review)

255. Br J Nurs. 2014 Jun 12-25;23(11):582-9.

Fingolimod for multiple sclerosis: a review for the specialist nurse.

Harrison K(1).

Author information:

(1)Neurology Nursing Specialist, Department of Neurology, Tergooiziekenhuizen,

Blaricum, Netherlands.

The availability of treatments for multiple sclerosis (MS) has increased

substantially over the past decade. Once-daily fingolimod 0.5 mg capsules

(Gilenya, Novartis Pharma) were approved in the European Union in March 2011 as

the first oral disease-modifying therapy for patients with relapsing MS. This

review summarises the efficacy and safety of fingolimod, and discusses practical

considerations for MS specialist nurses. Fingolimod has demonstrated efficacy in

the treatment of relapsing-remitting MS, as assessed by relapse measures,

inflammatory disease activity and brain volume loss. Evaluation of its safety

profile suggests a need for monitoring procedures for specific adverse events,

including transient, mostly asymptomatic, reductions in heart rate, blood

pressure increases, macular oedema and liver enzyme elevations. The MS nurse is

likely to be involved in monitoring treatment initiation, providing support in

the case of adverse events and promoting patient adherence to the prescribed

treatment regimen.

DOI: 10.12968/bjon.2014.23.11.582

PMID: 24933548 [Indexed for MEDLINE]

256. Patient Prefer Adherence. 2014 Jun 11;8:805-11. doi: 10.2147/PPA.S59496.

Excluded b/c no real-world adherence/persistence

eCollection 2014.

Enhanced patient support services improve patient persistence with multiple

sclerosis treatment.

Roche J(1), McCarry Y(2), Mellors K(3).

Author information:

(1)Beaumont Hospital, Dublin, Ireland.

(2)Quintiles Ireland Ltd, Fairview, Dublin, Ireland.

(3)Merck Serono Ltd, Feltham, UK.

BACKGROUND: Subcutaneous interferon beta-1a (sc IFN β-1a) therapy (44 µg or 22

µg, three times weekly) improves relapse rates and disability progression in

patients with relapsing multiple sclerosis (MS). While early treatment with

disease-modifying drugs may maximize therapeutic benefit, patients with low

adherence or long treatment gaps are at increased risk of relapse. MySupport is

an industry-sponsored program that provides support to patients with MS who have

been prescribed sc IFN β-1a in the UK or Republic of Ireland (ROI), via telephone

and text messaging, website access, and (in some cases) face-to-face support from

a dedicated MySupport Nurse. The aim of this audit was to assess if the MySupport

program in the ROI could improve persistence to sc IFN β-1a therapy.

METHODS: Anonymized data were supplied retrospectively from the MySupport

program, for ROI patients who were registered in January 2010 to receive sc IFN

β-1a three times weekly. Patients were recorded as "new" at their first drug

delivery; "active", if they continued to receive scheduled deliveries;

"interrupted", if their medication delivery was halted; or "stopped", if no

deliveries were made for 12 months. The number of "active" patients was recorded

monthly for 24 months. Results were compared with data from UK patients with MS,

who were receiving National Health Service (NHS) support only, or this support

plus MySupport.

RESULTS: A greater proportion of ROI patients receiving MySupport (compared

against UK patients receiving NHS support only) were on treatment at 12 months

(87.8% versus 79.3%) and at 24 months (76.2% versus 61.8%). The odds of being on

treatment were significantly greater, at all time points, for ROI patients

receiving MySupport, versus UK patients receiving NHS support only (P<0.0001).

CONCLUSION: A personalized support program, utilizing one-to-one nursing support

and additional support materials, can increase the probability of patients with

MS remaining on disease-modifying drug treatment.

DOI: 10.2147/PPA.S59496

PMCID: PMC4062553

PMID: 24966668

257. Mult Scler. 2014 Jun;20(7):889-91. doi: 10.1177/1352458513519839. Epub 2014 Jan

Excluded b/c no real-world adherence/persistence

20.

Interferon beta-related nephropathy and interstitial lung disease: a new

association and a long-term warning.

Capobianco M(1), Piccoli G(2), Neve Vigotti F(2), Scapoli P(3), Deagostini MC(2),

Albera C(4), Roccatello D(5), Bertolotto A(6).

Author information:

(1)SCDO Neurologia 2 - Centro di Riferimento Regionale Sclerosi Multipla,

Orbassano, Italy m.capobianco@alice.it.

(2)SS Nefrologia, Dipartimento di Scienze Cliniche e Biologiche, Università degli

Studi di Torino, Orbassano, Italy.

(3)Medicina Nucleare, Istituto per la Ricerca e la Cura del Cancro, Candiolo,

Italy.

(4)SCDU Pneumologia, Dipartimento di Scienze Cliniche e Biologiche, Univeristà

degli Studi di Torino, Orbassano, Italy.

(5)Centro Interreggionale Malattie Rare, Università degli Studi di Torino,

Torino, Italy.

(6)SCDO Neurologia 2 - Centro di Riferimento Regionale Sclerosi Multipla,

Orbassano, Italy.

We report on a so-far never described association between glomerulonephritis and

sarcoid-like lung disease after long-term interferon beta (IFNb) treatment for

relapsing-remitting multiple sclerosis. The interest in this case resides in the

documented remission after IFNb discontinuation. The history of IFNb-related

adverse events is probably not yet completely written. The rapid reversal of the

pathological signs in our patient underlines the importance of careful clinical

and laboratory surveillance, including kidney functional parameters, for an early

diagnosis of IFNb-related diseases.

© The Author(s) 2014.

DOI: 10.1177/1352458513519839

PMID: 24446386 [Indexed for MEDLINE]

Excluded b/c no primary data (systematic review)

258. Cochrane Database Syst Rev. 2014 May 24;(5):CD004927. doi:

10.1002/14651858.CD004927.pub4.

Surgical management of functional bladder outlet obstruction in adults with

neurogenic bladder dysfunction.

Utomo E(1), Groen J, Blok BF.

Author information:

(1)Department of Urology, Erasmus Medical Center, Room Na-1708, 's-Gravendijkwal

230, Rotterdam, Zuid-Holland, Netherlands, 3015 CE.

BACKGROUND: The most common type of functional bladder outlet obstruction in

patients with neurogenic bladder is detrusor-sphincter dyssynergia (DSD). The

lack of co-ordination between the bladder and the external urethral sphincter

muscle (EUS) in DSD can result in poor bladder emptying and high bladder

pressures, which may eventually lead to progressive renal damage.

OBJECTIVES: To assess the effectiveness of different surgical therapies for the

treatment of functional bladder outlet obstruction (i.e. DSD) in adults with

neurogenic bladder dysfunction.

SEARCH METHODS: We searched the Cochrane Incontinence Group Specialised Register,

which contains trials identified from the Cochrane Central Register of Controlled

Trials (CENTRAL), MEDLINE, MEDLINE In-Process, and handsearching of journals and

conference proceedings (searched 20 February 2014), and the reference lists of

relevant articles.

SELECTION CRITERIA: Randomised controlled trials (RCTs) or quasi-RCTs comparing a

surgical treatment of DSD in adults suffering from neurogenic bladder

dysfunction, with no treatment, placebo, non-surgical treatment, or other

surgical treatment, alone or in combination.

DATA COLLECTION AND ANALYSIS: Two review authors independently assessed trial

quality and extracted data.

MAIN RESULTS: We included five trials (total of 199 participants, average age of

40 years). The neurological diseases causing DSD were traumatic spinal cord

injury (SCI), multiple sclerosis (MS), or congenital malformations.One trial

compared placement of sphincteric stent prosthesis with sphincterotomy. For

urodynamic measurements, results for postvoid residual urine volume (PVR) and

cystometric bladder capacity were inconclusive and consistent with benefit of

either sphincteric stent prosthesis or sphincterotomy at three, six, 12, and 24

months. Results for maximum detrusor pressure (Pdet.max) were also inconclusive

at three, six, and 12 months; however, after two years, the Pdet.max after

sphincterotomy was lower than after stent placement (mean difference (MD) -30

cmH2O, 95% confidence interval (CI) 8.99 to 51.01).Four trials considered

botulinum A toxin (BTX-A) injection in the EUS, either alone or in combination

with other treatments. The comparators included oral baclofen, oral alpha

blocker, lidocaine, and placebo. The BTX-A trials all differed in protocols, and

therefore we did not undertake meta-analysis. A single 100 units transperineal

BTX-A injection (Botox®) in patients with MS resulted in higher voided urine

volumes (MD 69 mL, 95% CI 11.87 to 126.13), lower pre-micturition detrusor

pressure (MD -10 cmH2O, 95% CI -17.62 to -2.38), and lower Pdet.max (MD -14

cmH2O, 95% CI -25.32 to -2.68) after 30 days, compared to placebo injection.

Results for PVR using catheterisation, basal detrusor pressure, maximal bladder

capacity, maximal urinary flow, bladder compliance at functional bladder

capacity, maximal urethral pressure, and closure urethral pressure at 30 days

were inconclusive and consistent with benefit of either BTX-A injection or

placebo injections. In participants with SCI, treatment with 200 units of Chinese

manufactured BTX-A injected at eight different sites resulted in better bladder

compliance (MD 7.5 mL/cmH2O, 95% CI -10.74 to -4.26) than participants who

received the same injections with the addition of oral baclofen. Results for

maximum uroflow rate, maximal cystometric capacity, and volume per voiding were

inconclusive and consistent with benefit of either BTX-A injection or BTX-A

injection with the addition of oral baclofen. However, the poor quality of

reporting in this trial caused us to question the relevance of bladder compliance

as an adequate outcome measure.In participants with DSD due to traumatic SCI, MS,

or congenital malformation, the results for PVRs after one day were inconclusive

and consistent with benefit of either a single 100 units transperineal BTX-A

(Botox®) injection or lidocaine injection. However, after seven and 30 days of

BTX-A injection, PVRs were lower (MD -163 and -158 mL, 95% CI -308.65 to -17.35

and 95% CI -277.57 to -39.03, respectively) compared to participants who received

lidocaine injections. Results at one month for Pdet.max on voiding, EUS activity

in electromyography, and maximal urethral pressure were inconclusive and

consistent with benefit of either BTX-A or lidocaine injections.Finally, one

small trial consisting of five men with SCI compared weekly BTX-A injections with

normal saline as placebo. The placebo had no effect on DSD in the two

participants allocated to the placebo treatment. Their urodynamic parameters were

unchanged from baseline values until subsequent injections with BTX-A once a week

for three weeks. These subsequent injections resulted in similar responses to

those of the three participants who were allocated to the BTX-A treatment.

Unfortunately, the report presented no data on placebo treatment.Only the trial

that compared sphincterotomy with stent placement reported outcome measures renal

function and urologic complications related to DSD. Results for renal function at

12 and 24 months, and urologic complications related to DSD at three, six, 12,

and 24 months were inconclusive and consistent with benefit of either sphincteric

stent prosthesis or sphincterotomy.Adverse effects reported were haematuria due

to the cystoscopic injection and muscle weakness, of which the latter may be

related to the BTX-A dose used.All trials had some methodological shortcomings,

so insufficient information was available to permit judgement of risk of bias. At

least half of the trials had an unclear risk of selection bias and reporting

bias. One trial had a high risk of attrition bias, and another trial had a high

risk of reporting bias.

AUTHORS' CONCLUSIONS: Results from small studies with a high risk of bias have

identified evidence of limited quality that intraurethral BTX-A injections

improve some urodynamic measures after 30 days in the treatment of functional

bladder outlet obstruction in adults with neurogenic bladder dysfunction. The

necessity of reinjection of BTX-A is a significant drawback; a sphincterotomy

might therefore be a more effective treatment option for lowering bladder

pressure in the long-term.However, because of the limited availability of

eligible trials, this review was unable to provide robust evidence in favour of

any of the surgical treatment options. More RCTs are needed, measuring

improvement on quality of life, and on other types of surgical treatment options

for DSD since these are lacking. Future RCTs assessing the effectiveness of BTX-A

injections also need to address the uncertainty about the optimal dose and mode

of injection for this specific type of urological condition.

DOI: 10.1002/14651858.CD004927.pub4

PMID: 24859260 [Indexed for MEDLINE]

259. Neurol Ther. 2014 May 6;3(1):29-39. doi: 10.1007/s40120-014-0017-6. eCollection

Excluded b/c no primary data (narrative review)

2014 Jun.

Laquinimod therapy in multiple sclerosis: a comprehensive review.

Kolb-Sobieraj C(1), Gupta S(1), Weinstock-Guttman B(1).

Author information:

(1)Department of Neurology, School of Medicine and Biomedical Sciences, State

University of New York at Buffalo, New York, USA ; Baird MS Center, Jacobs

Neurological Institute, Buffalo General Medical Center, New York, USA.

INTRODUCTION: Multiple sclerosis (MS) is considered an autoimmune disease with

inflammatory and neurodegenerative underlying processes that affect the central

nervous system. The available disease-modifying therapies (DMTs) approved to

treat MS have only shown partial benefit in controlling the disease progression,

primarily impeding its inflammatory component, while the parenteral

administration of most of these therapies has shown to affect patient compliance.

Laquinimod is a promising new oral drug recently evaluated in a third phase III

clinical trial that demonstrated beneficial effects in delaying disease

progression and preventing brain atrophy, suggesting a potential neuroprotective

effect and a favorable safety profile.

AREAS COVERED: This is a comprehensive review covering clinical efficacy and

safety data obtained from two phase III clinical trials, as well as the presumed

beneficial mechanism of action, of laquinimod. This article also provides a short

overview of the oral DMTs recently approved for the treatment of relapsing MS, as

well as challenges that still remain to be overcome to fully control the

relentless course of MS.

CONCLUSION: Laquinimod has been shown to have a novel immunomodulatory and

potential neuroprotective mechanism of action as suggested from animal models and

in vitro experimental data. Phase III clinical trials ALLEGRO (Clinicaltrials.gov

#NCT00509145) and BRAVO (Clinicaltrials.gov #NCT00605215) have demonstrated

clinical efficacy and tolerability, while the third phase III study is currently

evaluating the safety and efficacy of laquinimod at a higher dosage. Emerging

oral treatments like laquinimod will provide new options for patients to consider

that can lead to better patient adherence and improved outcomes.

DOI: 10.1007/s40120-014-0017-6

PMCID: PMC4381916

PMID: 26000222

Excluded b/c no primary data (narrative review)

260. Expert Opin Pharmacother. 2014 May;15(7):1019-27. doi:

10.1517/14656566.2014.902936. Epub 2014 Apr 17.

Teriflunomide: a novel oral treatment for relapsing multiple sclerosis.

Sartori A(1), Carle D, Freedman MS.

Author information:

(1)University of Trieste, Ospedale di Cattinara - Azienda

Ospedaliero-Universitaria Ospedali Riuniti, Department of Medical, Surgical and

Health Sciences (Neurology) , 447 Strada di Fiume, 34149 Trieste , Italy +39 040

399 4321 ; +39 040 910 861 ; sartoriarianna23@gmail.com.

INTRODUCTION: Multiple sclerosis is a disabling chronic inflammatory disease of

the CNS. New emerging oral treatments can offer efficacy with higher levels of

therapeutic adherence. Teriflunomide is one such oral agent that has recently

been approved for the treatment of relapsing multiple sclerosis (RMS).

AREAS COVERED: The aim of this review is to describe the pharmacological profile

of teriflunomide and review the vast clinical development program that paved the

way for its approval, with emphasis on its safety and tolerability.

EXPERT OPINION: Teriflunomide is a safe new oral medication for treating RMS. It

is effective at reducing relapses, MRI activity and slowing disability

progression. It is well tolerated, with mild and transitory side effects.

Although teriflunomide is given a pregnancy category 'X' by the FDA and an

effective contraception is needed, to date, there has been no evidence of

teratogenicity in humans and a rapid washout procedure can lead to a virtually

complete elimination. Its effectiveness appeared to be at least comparable to

that of high-dose IFN-β-1a, and although direct comparisons with other orals are

still lacking, its tolerability and encouraging safety data suggest that

teriflunomide could be considered an ideal first-line medication for RMS.

DOI: 10.1517/14656566.2014.902936

PMID: 24742277 [Indexed for MEDLINE]

261. Mult Scler. 2014 May;20(6):705-16. doi: 10.1177/1352458513507821. Epub 2013 Oct

Excluded b/c no real-world adherence/persistence

14.

Teriflunomide versus subcutaneous interferon beta-1a in patients with relapsing

multiple sclerosis: a randomised, controlled phase 3 trial.

Vermersch P(1), Czlonkowska A, Grimaldi LM, Confavreux C, Comi G, Kappos L,

Olsson TP, Benamor M, Bauer D, Truffinet P, Church M, Miller AE, Wolinsky JS,

Freedman MS, O'Connor P; TENERE Trial Group.

Author information:

(1)Neurology, University of Lille Nord de France, Lille, France.

BACKGROUND: In previous studies, teriflunomide significantly reduced the

annualised relapse rate (ARR) and disability progression.

OBJECTIVE: This phase 3, rater-blinded study (NCT00883337) compared teriflunomide

with interferon-beta-1a (IFNβ-1a).

METHODS: Patients with relapsing multiple sclerosis were randomised (1:1:1) to

oral teriflunomide 7-or 14 mg, or subcutaneous IFNβ-1a 44 µg. The primary

composite endpoint was time to failure, defined as first occurrence of confirmed

relapse or permanent treatment discontinuation for any cause. Secondary endpoints

included ARR, Fatigue Impact Scale (FIS) and Treatment Satisfaction Questionnaire

for Medication (TSQM). The study was completed 48 weeks after the last patient

was randomised.

RESULTS: Some 324 patients were randomised (IFNβ-1a: 104; teriflunomide 7 mg:

109; teriflunomide 14 mg: 111). No difference in time to failure was observed.

There was no difference in ARR between teriflunomide 14 mg and IFNβ-1a, but ARR

was significantly higher with teriflunomide 7 mg. FIS scores indicated more

frequent fatigue with IFNβ-1a, though differences were only significant with

teriflunomide 7 mg. TSQM scores were significantly higher with teriflunomide.

There were no unexpected safety findings.

CONCLUSION: Effects on time to failure were comparable between teriflunomide and

IFNβ-1a. There was no difference between teriflunomide 14 mg and IFNβ-1a on ARR,

though ARR was higher with teriflunomide 7 mg. The teriflunomide safety profile

was consistent with previous studies.

DOI: 10.1177/1352458513507821

PMID: 24126064 [Indexed for MEDLINE]

262. Mult Scler Relat Disord. 2014 May;3(3):408-12. doi: 10.1016/j.msard.2013.11.001.

Excluded b/c no real-world adherence/persistence

Epub 2013 Nov 28.

Abnormal rhythms in patients without known cardiac disease after a first dose of

fingolimod.

Schurmann P(1), Basra S(2), Awar OG(3), Aguilar D(4), Basant A(5), Dragan E(6),

Hutton GJ(7), Birnbaum Y(8).

Author information:

(1)The Section of Cardiology, The Department of Medicine, Baylor College of

Medicine, One Baylor Plaza, Houston, TX 77030, USA. Electronic address:

schurman@bcm.edu.

(2)The Section of Cardiology, The Department of Medicine, Baylor College of

Medicine, One Baylor Plaza, Houston, TX 77030, USA. Electronic address:

basra@bcm.edu.

(3)The Section of Cardiology, The Department of Medicine, Baylor College of

Medicine, One Baylor Plaza, Houston, TX 77030, USA. Electronic address:

awar@bcm.edu.

(4)The Section of Cardiology, The Department of Medicine, Baylor College of

Medicine, One Baylor Plaza, Houston, TX 77030, USA. Electronic address:

daguilar@bcm.edu.

(5)The Section of Cardiology, The Department of Medicine, Baylor College of

Medicine, One Baylor Plaza, Houston, TX 77030, USA. Electronic address:

barya@bcm.edu.

(6)The Department of Neurology, Baylor College of Medicine, One Baylor Plaza,

Houston, TX 77030, USA. Electronic address: dragan@bcm.edu.

(7)The Department of Neurology, Baylor College of Medicine, One Baylor Plaza,

Houston, TX 77030, USA. Electronic address: ghutton@bcm.edu.

(8)The Section of Cardiology, The Department of Medicine, Baylor College of

Medicine, One Baylor Plaza, Houston, TX 77030, USA. Electronic address:

ybirnbau@bcm.edu.

BACKGROUND: Fingolimod is used to reduce the rates of relapse and slow the

progression of disability in relapsing-remitting multiple sclerosis (RRMS).

In-office monitoring of patients for 6h after the first dose of fingolimod is

currently recommended due to rare cardiac rhythm disturbances. The objective of

this paper is to describe our experience with continuous electrocardiographic

monitoring of patients with RRMS starting on fingolimod.

METHODS: Since changes to the FDA recommendations for first dose observation, a

total of 59 patients with RRMS began treatment with fingolimod. After the first

dose, all patients were observed for 6h with continuous electrocardiographic

telemetry, vital signs were checked every hour, and 12 lead ECG performed before

and after the 6-h period.

RESULTS: Three out of 59 (5%) patients developed arrhythmia that led to

discontinuation of fingolimod. The first patient had a sinus bradycardia with

idioventricular escape rhythm that lasted 45s and two patients developed

second-degree atrio-ventricular block Mobitz type I. None of the patients had a

history of prior cardiacc disease or was taking other medications that may cause

arrhythmia or bradycardia.

CONCLUSION: Continuous on-line electrocardiographic telemetry may detect abnormal

rhythms in a small number of patients started on fingolimod. The clinical

significance of these is unclear and warrants further study.

© 2013 Elsevier B.V. All rights reserved.

DOI: 10.1016/j.msard.2013.11.001

PMID: 25876482

263. Mult Scler Relat Disord. 2014 May;3(3):355-63. doi: 10.1016/j.msard.2013.11.006.

Excluded b/c no real-world adherence/persistence

Epub 2013 Dec 12.

Effect of prior treatment status and reasons for discontinuation on the efficacy

and safety of fingolimod vs. interferon β-1a intramuscular: Subgroup analyses of

the Trial Assessing Injectable Interferon vs. Fingolimod Oral in

Relapsing-Remitting Multiple Sclerosis (TRANSFORMS).

Khatri BO(1), Pelletier J(2), Kappos L(3), Hartung HP(4), Comi G(5), Barkhof

F(6), von Rosenstiel P(7), Meng X(8), Grinspan A(9), Hashmonay R(10), Cohen

JA(11); TRANSFORMS Study Group.

Author information:

(1)The Regional MS Center, Center for Neurological Disorders, Wheaton Franciscan

Health Care, 3237 S. 16th Street, Milwaukee, WI 53215, USA. Electronic address:

bokhatri@aol.com.

(2)CHU La Timone, CRMBM-CNRS 6612, Marseille, France. Electronic address:

jean.pelletier@mail.ap-hm.fr.

(3)University Hospital, Petersgraben 4, Basel 4031, Switzerland. Electronic

address: lkappos@uhbs.ch.

(4)Heinrich Heine University, Moorenstr 5, Dusseldorf 40225, Germany. Electronic

address: Hans-Peter.Hartung@uniduesseldorf.de.

(5)Università Vita-Salute San Raffaele, Milan 20123, Italy. Electronic address:

comi.giancarlo@unisr.it.

(6)VU Medical Center, P.O. Box 7057, Amsterdam 1007 MB, The Netherlands.

Electronic address: f.barkhof@vumc.nl.

(7)Novartis Pharma AG, Kirschgarten Site, Basel 4002, Switzerland. Electronic

address: philipp.von_rosenstiel@novartis.com.

(8)Novartis Pharmaceuticals Corporation, One Health Plaza, East Hanover, NJ

07936, USA. Electronic address: Xiangyi.meng@novartis.com.

(9)Novartis Pharmaceuticals Corporation, One Health Plaza, East Hanover, NJ

07936, USA. Electronic address: augustogrinspan@hotmail.com.

(10)Novartis Pharmaceuticals Corporation, One Health Plaza, East Hanover, NJ

07936, USA. Electronic address: ronny.hashmonay@novartis.com.

(11)Mellen Center, Cleveland Clinic Foundation, 9500 Euclid Avenue, Cleveland, OH

44195, USA. Electronic address: cohenj@ccf.org.

BACKGROUND: Fingolimod demonstrated superior efficacy compared with interferon

β-1a intramuscular in relapsing multiple sclerosis. The impact of treatment

history on fingolimod efficacy is unknown.

OBJECTIVES: This post-hoc analysis of phase 3 TRANSFORMS data compared the

efficacy and safety of fingolimod and interferon β-1a intramuscular among patient

subgroups defined by prior treatment history.

METHODS: Annualized relapse rate and safety of once-daily oral fingolimod 0.5mg,

1.25mg, or once-weekly interferon β-1a 30μg intramuscular for 12 months were

analyzed in 1292 patients with relapsing multiple sclerosis according to prior

disease-modifying therapy, reason for prior disease-modifying therapy

discontinuation (adverse events or unsatisfactory therapeutic effect), and prior

disease-modifying therapy duration.

RESULTS: Compared with interferon β-1a intramuscular, fingolimod 0.5mg

significantly reduced annualized relapse rate in patients who were treatment

naive, received prior interferon-β treatment, discontinued prior

disease-modifying therapy for unsatisfactory therapeutic effect, or had prior

disease-modifying therapy duration of ≥1 year (P≤0.05, all comparisons). Similar

trends were observed in patients with prior glatiramer acetate treatment.

Significant reductions were also seen with fingolimod 1.25mg for treatment-naive

and prior interferon-β-treated patients.

CONCLUSIONS: This analysis demonstrates superiority of fingolimod over interferon

β-1a intramuscular regardless of prior (interferon-β) treatment and prior

treatment efficacy and duration. ClinicalTrials.gov identifier: NCT00340834.

© 2013 Published by Elsevier B.V.

DOI: 10.1016/j.msard.2013.11.006

PMID: 25876473

264. Mult Scler Relat Disord. 2014 May;3(3):341-9. doi: 10.1016/j.msard.2013.10.006.

Excluded b/c no real-world adherence/persistence

Epub 2013 Nov 5.

Impact of prior treatment status and reasons for discontinuation on the efficacy

and safety of fingolimod: Subgroup analyses of the Fingolimod Research Evaluating

Effects of Daily Oral Therapy in Multiple Sclerosis (FREEDOMS) study.

Kremenchutzky M(1), O'Connor P(2), Hohlfeld R(3), Zhang-Auberson L(4), von

Rosenstiel P(5), Meng X(6), Grinspan A(7), Hashmonay R(8), Kappos L(9).

Author information:

(1)London (Ont) MS Clinic, Western University, 339 Windermere Road, London, ON,

Canada N6A6A5. Electronic address: marcelo.kremenchutzky@LHSC.ON.CA.

(2)St. Michael's Hospital, 30 Bond Street, Suite 3 007 Shuter Wing, Toronto, ON,

Canada M5B1W8. Electronic address: oconnorp@smh.toronto.on.ca.

(3)Institut fur Klinische Neuroimmunologie, University of Munich, Marchioninistr

15, Munich D-81377, Germany. Electronic address:

reinhard.hohlfeld@med.uni-muenchen.de.

(4)Novartis Pharma AG, Basel 4002, Switzerland. Electronic address:

lixin.zhang_auberson@novartis.com.

(5)Novartis Pharma AG, Basel 4002, Switzerland. Electronic address:

philipp.von_rosenstiel@novartis.com.

(6)Novartis Pharmaceuticals Corporation, One Health Plaza, East Hanover, NJ

07936, USA. Electronic address: Xiangyi.meng@novartis.com.

(7)Novartis Pharmaceuticals Corporation, One Health Plaza, East Hanover, NJ

07936, USA. Electronic address: augustogrinspan@hotmail.com.

(8)Novartis Pharmaceuticals Corporation, One Health Plaza, East Hanover, NJ

07936, USA. Electronic address: ronny.hashmonay@novartis.com.

(9)University Hospital, Petersgraben 4, Basel 4031, Switzerland. Electronic

address: lkappos@uhbs.ch.

BACKGROUND: Fingolimod is a once-daily, oral sphingosine 1-phosphate receptor

modulator approved for the treatment of relapsing multiple sclerosis.

OBJECTIVE: This post-hoc analysis of phase 3 FREEDOMS data assessed whether the

effects of fingolimod are consistent among subgroups of patients defined by prior

treatment history.

METHODS: Annualized relapse rate and safety profile of treatment with fingolimod

0.5mg, 1.25mg, or placebo once-daily for 24 months were analyzed in 1272

relapsing multiple sclerosis patients, by subgroups based on disease-modifying

therapy history (treatment-naive; prior interferon-β or glatiramer acetate),

reason for discontinuation of prior disease-modifying therapy (unsatisfactory

therapeutic response or adverse events), and prior disease-modifying therapy

duration.

RESULTS: Both fingolimod doses significantly reduced annualized relapse rate in

patients that received prior interferon-β or glatiramer acetate, discontinued

prior disease-modifying therapy owing to unsatisfactory therapeutic effect, were

treatment-naive, or had prior disease-modifying therapy duration of >1-3 years

(P≤0.0301 for all comparisons vs placebo). Fingolimod 1.25mg resulted in greater

reductions in annualized relapse rate in patients that discontinued prior

disease-modifying therapy for adverse events or had prior disease-modifying

therapy duration of ≤1 year or >3 years (P≤0.0194 vs placebo).

CONCLUSIONS: Fingolimod demonstrated similar efficacy in relapsing multiple

sclerosis patients regardless of prior treatment history. Clinicaltrials.gov

identifier: NCT00289978.

© 2013 The Authors. Published by Elsevier B.V. All rights reserved.

DOI: 10.1016/j.msard.2013.10.006

PMID: 25876471

265. Rehabil Psychol. 2014 May;59(2):136-46. doi: 10.1037/a0036322. Epub 2014 Apr 7.

Excluded b/c no real-world adherence/persistence

Telephone counseling and home telehealth monitoring to improve medication

adherence: results of a pilot trial among individuals with multiple sclerosis.

Turner AP(1), Sloan AP(1), Kivlahan DR(1), Haselkorn JK(1).

Author information:

(1)VA Puget Sound Health Care System.

OBJECTIVE: To evaluate the impact upon medication adherence of brief

telephone-based counseling using principles of motivational interviewing and

telehealth home monitoring.

DESIGN: Randomized controlled pilot trial of 19 veterans with multiple sclerosis

(MS) currently prescribed disease modifying therapy (DMT) who endorsed missing

doses. Follow-up was conducted at 1, 3, and 6 months.

RESULTS: Participants in the intervention condition reported better adherence

relative to controls at 6-month follow-up [M (SD) = 1.3 (2.1) vs. 8.2 (12.3) past

month missed doses]. All participants in the intervention condition completed all

3 telephone counseling sessions and 90% or greater rated the program as highly

successful.

CONCLUSION: Brief telephone counseling represents a promising mechanism for

improving medication adherence. The primary components, motivational interviewing

and home telehealth monitoring, provided complementary mechanisms for initiating

and sustaining behavior change over time. The intervention was well tolerated and

provided an opportunity to extend access and reduce barriers to care by bringing

it into the homes of participants.

(c) 2014 APA, all rights reserved.

DOI: 10.1037/a0036322

PMID: 24708232 [Indexed for MEDLINE]

Excluded b/c no real-world adherence/persistence

266. Patient Prefer Adherence. 2014 Apr 3;8:415-22. doi: 10.2147/PPA.S53140.

eCollection 2014.

Treatment selection and experience in multiple sclerosis: survey of neurologists.

Hanson KA(1), Agashivala N(2), Wyrwich KW(3), Raimundo K(2), Kim E(2), Brandes

DW(4).

Author information:

(1)UBC: An Express Scripts Company, Dorval, QC, Canada.

(2)Novartis Pharmaceuticals Corporation East Hanover, NJ, USA.

(3)Evidera, Bethesda, MD, USA.

(4)Hope MS Center, Knoxville, TN, USA.

BACKGROUND: Multiple sclerosis (MS) is a complex disease with many therapeutic

options. Little is known about how neurologists select particular

disease-modifying therapies (DMTs) for their patients.

OBJECTIVE: To understand how neurologists make decisions regarding the

prescription of DMTs for patients with MS, and to explore neurologists'

experiences with individual DMTs.

METHODS: From December 2012 to January 2013, members of a nationwide physician

market research panel were sent an online study invitation with a link to a

survey website. Eligible neurologists were included if they currently practice

medicine in the United States, and if they treat ≥20 patients with MS.

RESULTS: A total of 102 neurologists (n=63 general neurologists; n=39 MS

specialists; 81.4% male) completed the survey. The mean (standard deviation)

number of years in practice since completing medical training was 16.4 (8.6)

years. Overall, the most commonly prescribed DMTs were subcutaneous interferon

(IFN) β-1a and glatiramer acetate; approximately 5.5% of patients were untreated.

The most important attributes of DMT medication selection were (in order of

importance) efficacy, safety, tolerability, patient preference, and convenience.

The DMT with the highest neurologist-reported percentage of patients who were

"Very/Extremely Satisfied" with their therapy was fingolimod (31.0%), followed by

glatiramer acetate (13.9%; P=0.017). Compared with fingolimod (94.0%),

significantly fewer (P<0.05) neurologists reported that "All/Most" of their

patients were adherent to treatment with glatiramer acetate (78.0%), subcutaneous

IFN β-1a (84.0%), and IFN β-1b (75.0%); no significant differences were observed

with intramuscular IFN β-1a (92.9%; P=0.75). Patients' calls to neurologists'

offices were most commonly related to side effects for all self-injectable DMTs,

whereas calls about fingolimod primarily involved insurance coverage issues.

CONCLUSION: Our survey results showed that very few patients with MS did not

received any DMT. Among the DMTs available at the time of the survey,

neurologists reported that patients were most satisfied with, and adherent to,

fingolimod, but these patients also faced more problems with insurance coverage

when compared with those taking self-injectable DMTs.

DOI: 10.2147/PPA.S53140

PMCID: PMC3979792

PMID: 24729689

Excluded b/c no primary data (narrative review)

267. Int J MS Care. 2014 Spring;16(1):55-60. doi: 10.7224/1537-2073.2013-006.

Nurses' perspective on approaches to limit flu-like symptoms during interferon

therapy for multiple sclerosis.

Filipi ML(1), Beavin J(1), Brillante RT(1), Costello K(1), Hartley GC(1), Hartley

K(1), Namey M(1), O'Leary S(1), Remington G(1).

Author information:

(1)College of Nursing, University of Nebraska Medical Center, Omaha, NE, USA

(MLF); Biogen Idec, Weston, MA, USA (substantial portion of contributions made

while employed at Forget-Me-Not Home Memory Care, Raleigh, NC, USA) (JB); Biogen

Idec, Weston, MA, USA (substantial portion of contributions made while employed

at Rush Multiple Sclerosis Center, Chicago, IL, USA) (RTB); Department of

Neurology, Johns Hopkins Hospital, Baltimore, MD, USA (KC); Acadia Neurology

Center, Acadia, CA, USA (GCH); Providence Multiple Sclerosis Center, Providence

St. Vincent Medical Center, Portland, OR, USA (KH); Mellen Center for Multiple

Sclerosis Treatment and Research, Cleveland Clinic Foundation, Cleveland, OH, USA

(MN); Novartis Pharmaceuticals Corporation, East Hanover, NJ, USA (substantial

portion of contributions made while employed at Texas Neurology, Dallas, TX, USA)

(SO); and Clinical Center for Multiple Sclerosis, Department of Neurology,

University of Texas Southwestern Medical Center, Dallas, TX, USA (GR). Kay

Hartley is now with Providence Home Health, Portland, OR, USA.

BACKGROUND: Several interferon beta (IFNβ) formulations are approved for

first-line use as disease-modifying therapies to treat patients with multiple

sclerosis (MS). Systemic post-injection reactions, often termed flu-like symptoms

(FLS), occur in approximately half of all patients treated with IFNβs and can

affect adherence to therapy. These symptoms, which include pyrexia, chills,

malaise, myalgia, and headaches, usually resolve within 24 hours or persist

intermittently following each injection. Because FLS, which usually occur early

in the treatment course and diminish over time, are a primary cause of

nonadherence to IFNβ therapy, it is important to employ strategies that can

attenuate these side effects.

METHODS: To identify interventions effective in limiting FLS, a panel of United

States-based nurses with expertise in MS patient care was convened and a

literature review completed.

RESULTS: Panel consensus was reached on specific interventions that can attenuate

FLS. These prevention and mitigation strategies include dose titration,

analgesia, and optimal injection timing, as well as other techniques that panel

members have found useful in their clinical practice experience.

CONCLUSIONS: These measures, in addition to effective patient education, will

help to reduce the incidence of FLS secondary to IFNβ therapy, improve patient

medication adherence, and positively affect long-term clinical outcomes.

DOI: 10.7224/1537-2073.2013-006

PMCID: PMC3967704

PMID: 24688355

268. J Intern Med. 2014 Apr;275(4):350-63. doi: 10.1111/joim.12203. Epub 2014 Mar 11.

Excluded b/c no primary data (narrative review)

Established and novel disease-modifying treatments in multiple sclerosis.

Cross AH(1), Naismith RT.

Author information:

(1)Department of Neurology, Washington University, St. Louis, MO, USA.

Comment in

J Intern Med. 2014 Apr;275(4):382-6.

Multiple sclerosis (MS) is a presumed autoimmune disorder of the central nervous

system, resulting in inflammatory demyelination and axonal and neuronal injury.

New diagnostic criteria that incorporate magnetic resonance imaging have resulted

in earlier and more accurate diagnosis of MS. Several immunomodulatory and

immunosuppressive therapeutic agents are available for relapsing forms of MS,

which allow individualized treatment based upon the benefits and risks.

Disease-modifying therapies introduced in the 1990s, the beta-interferons and

glatiramer acetate, have an established track record of efficacy and safety,

although they require administration via injection. More recently, monoclonal

antibodies have been engineered to act through specific mechanisms such as

blocking alpha-4 integrin interactions (natalizumab) or lysing cells bearing

specific markers, for example CD52 (alemtuzumab) or CD20 (ocrelizumab and

ofatumumab). These agents can be highly efficacious, but sometimes have serious

potential complications (natalizumab is associated with progressive multifocal

leukoencephalopathy; alemtuzumab is associated with the development of new

autoimmune disorders). Three new oral therapies (fingolimod, teriflunomide and

dimethyl fumarate, approved for MS treatment from 2010 onwards) provide efficacy,

tolerability and convenience; however, as yet, there are no long-term

postmarketing efficacy and safety data in a general MS population. Because of

this lack of long-term data, in some cases, therapy is currently initiated with

the older, safer injectable medications, but patients are monitored closely with

the plan to switch therapies if there is any indication of a suboptimal response

or intolerance or lack of adherence to the initial therapy. For patients with MS

who present with highly inflammatory and potentially aggressive disease, the

benefit-to-risk ratio may support initiating therapy using a drug with greater

potential efficacy despite greater risks (e.g. fingolimod or natalizumab if JC

virus antibody-negative). The aim of this review is to discuss the clinical

benefits, mechanisms of action, safety profiles and monitoring strategies of

current MS disease-modifying therapies in clinical practice and of those expected

to enter the market in the near future.

© 2014 The Association for the Publication of the Journal of Internal Medicine.

DOI: 10.1111/joim.12203

PMID: 24444048 [Indexed for MEDLINE]

269. JAMA Neurol. 2014 Apr;71(4):436-41. doi: 10.1001/jamaneurol.2013.6240.

Excluded b/c no real-world adherence/persistence

Switching from natalizumab to fingolimod in multiple sclerosis: a French

prospective study.

Cohen M(1), Maillart E(2), Tourbah A(3), De Sèze J(4), Vukusic S(5), Brassat

D(6), Anne O(7), Wiertlewski S(8), Camu W(9), Courtois S(10), Ruet A(11),

Debouverie M(12), Le Page E(13), Casez O(14), Heinzlef O(15), Stankoff B(16),

Bourre B(17), Castelnovo G(18), Rico A(19), Berger E(20), Camdessanche JP(21),

Defer G(22), Clavelou P(23), Al Khedr A(24), Zephir H(25), Fromont A(26), Papeix

C(2), Brochet B(10), Pelletier J(18), Lebrun C(1); Club Francophone de la

Sclérose en Plaques Investigators.

Collaborators: Barth P, Blanc F, Boulay C, Camu W, Chambaud L, Clavelou P,

Collongues N, Coustans M, Creange A, Derouiche F, Edan G, Fleury M, Gout O,

Guennoc AM, Kopf A, Labauge P, Lallement F, Laplaud D, Louiset P, Malikova I,

Moreau T, Ouallet JC, Pittion S, Rouhart F, Rumbach L, Seeldrayers P, Taithe F,

Taurin G, Thouvenot E, Vermersch P, Zaenker C.

Author information:

(1)Department of Neurology, University Hospital of Nice, Nice, France.

(2)Department of Neurology, Salpêtrière Hospital, Paris, France.

(3)Department of Neurology, University Hospital of Reims, Reims, France.

(4)Department of Neurology, University Hospital of Strasbourg, Strasbourg,

France.

(5)Department of Neurology, University Hospital of Lyon, Lyon, France.

(6)Department of Neurology, University Hospital of Toulouse, Toulouse, France.

(7)Department of Neurology, University Hospital of La Rochelle, La Rochelle,

France.

(8)Department of Neurology, University Hospital of Nantes, Nantes, France.

(9)Department of Neurology, University Hospital of Montpellier, Montpellier,

France.

(10)Department of Neurology, University Hospital of Mulhouse, Mulhouse, France.

(11)Department of Neurology, University Hospital of Bordeaux, Bordeaux, France.

(12)Department of Neurology, University Hospital of Nancy, Nancy, France.

(13)Department of Neurology, University Hospital of Rennes, Rennes, France.

(14)Department of Neurology, University Hospital of Grenoble, Grenoble, France.

(15)Department of Neurology, University Hospital of Poissy, Poissy, France.

(16)Department of Neurology, Tenon Hospital, Paris, France.

(17)Department of Neurology, University Hospital of Rouen, Rouen, France.

(18)Department of Neurology, University Hospital of Nimes, Nimes, France.

(19)Department of Neurology, University Hospital of Marseille, Marseille, France.

(20)Department of Neurology, University Hospital of Besançon, Besançon, France.

(21)Department of Neurology, University Hospital of Saint Etienne, Saint Etienne,

France.

(22)Department of Neurology, University Hospital of Caen, Caen, France.

(23)Department of Neurology, University Hospital of Clermont Ferrand, Clermont

Ferrand, France.

(24)Department of Neurology, University Hospital of Amiens, Amiens, France.

(25)Department of Neurology, University Hospital of Lille, Lille, France.

(26)Department of Neurology, University Hospital of Dijon, Dijon, France.

Comment in

JAMA Neurol. 2014 Jul 1;71(7):925.

JAMA Neurol. 2014 Jul 1;71(7):924-5.

Nat Rev Neurol. 2014 Jun;10(6):311-3.

IMPORTANCE: The safety and efficacy of switching from natalizumab to fingolimod

have not yet been evaluated in a large cohort of patients with multiple sclerosis

(MS) to our knowledge.

OBJECTIVE: To collect data from patients with MS switching from natalizumab to

fingolimod.

DESIGN, SETTING, AND PARTICIPANTS: The Enquête Nationale sur l'Introduction du

Fingolimod en Relais au Natalizumab (ENIGM) study, a survey-based, observational

multicenter cohort study among MS tertiary referral centers. Participants were

patients for whom a switch from natalizumab to fingolimod was planned. Clinical

data were collected on natalizumab treatment, duration and management of the

washout period (WP), and relapse or adverse events during the WP and after the

initiation of fingolimod.

MAIN OUTCOMES AND MEASURES: Occurrence of MS relapse during the WP or during a

6-month follow-up period after the initiation of fingolimod.

RESULTS: Thirty-six French MS tertiary referral centers participated. In total,

333 patients with MS switched from natalizumab to fingolimod after a mean of 31

natalizumab infusions (female to male ratio, 2.36; mean age, 41 years; and

Expanded Disability Status Scale score at the initiation of natalizumab, 3.6).

Seventy-one percent were seropositive for the JC polyomavirus. The Expanded

Disability Status Scale score remained stable for patients receiving natalizumab.

Twenty-seven percent of patients relapsed during the WP. A WP shorter than 3

months was associated with a lower risk of relapse (odds ratio, 0.23; P = .001)

and with less disease activity before natalizumab initiation (P = .03). Patients

who stopped natalizumab because of poor tolerance or lack of efficacy also had a

higher risk of relapse (odds ratio, 3.20; P = .004). Twenty percent of patients

relapsed during the first 6 months of fingolimod therapy. Three percent stopped

fingolimod for efficacy, tolerance, or compliance issues. In the multivariate

analysis, the occurrence of relapse during the WP was the only significant

prognostic factor for relapse during fingolimod therapy (odds ratio, 3.80;

P = .05).

CONCLUSIONS AND RELEVANCE: In this study, switching from natalizumab to

fingolimod was associated with a risk of MS reactivation during the WP or shortly

after fingolimod initiation. The WP should be shorter than 3 months.

DOI: 10.1001/jamaneurol.2013.6240

PMID: 24566807 [Indexed for MEDLINE]

270. Mult Scler. 2014 Apr;20(4):505-9. doi: 10.1177/1352458513516530. Epub 2013 Dec

Excluded b/c no real-world adherence/persistence

23.

Fingolimod to treat severe multiple sclerosis after natalizumab-associated

progressive multifocal leukoencephalopathy: a valid option?

Maillart E(1), Louapre C, Lubetzki C, Papeix C.

Author information:

(1)APHP, Hôpital Pitié Salpêtrière, France.

Comment in

Mult Scler. 2014 Apr;20(4):510-1.

We report two cases of multiple sclerosis (MS) patients with

natalizumab-associated progressive multifocal leukoencephalopathy. Severe MS

relapses occurred between four and five months after natalizumab discontinuation.

After failure of immunomodulatory treatment, both patients were treated with

fingolimod. The outcome was positive on clinical and MRI disease activity,

without worsening of the progressive multifocal leukoencephalopathy. These

observations suggest that using fingolimod for severe multiple sclerosis after

natalizumab-associated progressive multifocal leukoencephalopathy may be an

option, under close clinical and radiological monitoring.

DOI: 10.1177/1352458513516530

PMID: 24367037 [Indexed for MEDLINE]

271. Mult Scler. 2014 Apr;20(4):471-80. doi: 10.1177/1352458513500551. Epub 2013 Aug

Excluded b/c no real-world adherence/persistence

15.

Temporal profile of lymphocyte counts and relationship with infections with

fingolimod therapy.

Francis G(1), Kappos L, O'Connor P, Collins W, Tang D, Mercier F, Cohen JA.

Author information:

(1)Novartis Pharmaceuticals Corporation-NJ, USA.

BACKGROUND: Reduction in peripheral blood lymphocytes is an expected

pharmacodynamic outcome of fingolimod therapy.

OBJECTIVE: The objective of this article is to evaluate lymphocyte dynamics

during and after fingolimod therapy and assess the relationship between

lymphocyte counts and infections.

METHODS: Lymphocyte counts and their relationship with infections were evaluated

in three multiple sclerosis (MS) populations: (Group A) FREEDOMS phase 3 core

study group (n = 1272); (Group B) All Studies group (one phase 2 and two phase 3

studies, plus their extensions; n = 2315); and (Group C) Follow-up group (after

fingolimod discontinuation; n = 538).

RESULTS: Administration of fingolimod 0.5 mg led to reductions in lymphocyte

counts to a steady-state of 24%-30% of baseline values within two weeks, which

remained stable while on therapy. Following fingolimod discontinuation, average

counts exceeded the lower limit of normal range within six to eight weeks, and

were 80% of baseline values by three months. In Group A, infection rates per

patient-year were 1.4 with placebo and 1.0 in fingolimod-treated patients who had

the lowest lymphocyte counts (< 0.2 × 10(9)/l). No evidence was seen for an

increase in serious or opportunistic infections.

CONCLUSIONS: Fingolimod induces a rapid and reversible reduction in lymphocyte

counts without an increase in infections relative to placebo. Because fingolimod

reduces blood lymphocyte counts via redistribution in secondary lymphoid organs,

peripheral blood lymphocyte counts cannot be utilized to evaluate the lymphocyte

subset status of a patient.

DOI: 10.1177/1352458513500551

PMID: 23950550 [Indexed for MEDLINE]

Excluded b/c no primary data (narrative review)

272. Ther Clin Risk Manag. 2014 Mar 27;10:229-39. doi: 10.2147/TCRM.S53285.

eCollection 2014.

Pharmacology and clinical efficacy of dimethyl fumarate (BG-12) for treatment of

relapsing-remitting multiple sclerosis.

Ruggieri S(1), Tortorella C(2), Gasperini C(3).

Author information:

(1)Department of Neurology and Psychiatry, Sapienza University of Rome

"Sapienza", Rome, Italy.

(2)Department of Basic Sciences, Neurosciences and Sense Organs, University of

Bari, Bari, Italy.

(3)Department of Neurosciences, S Camillo Forlanini Hospital, Rome, Italy.

The last two decades have seen the introduction of several therapies for multiple

sclerosis (MS). These therapies are intended to work at different levels of the

disease, typically targeting direct symptom management, brief corticosteroid

administration for acute exacerbations, and the regular use of disease-modifying

drugs. Nevertheless, in clinical practice, disease-modifying drugs or

immunosuppressive treatments are frequently associated with suboptimal response

in terms of efficacy and several side effects leading to poor patient adherence,

so the proportion of relapsing-remitting MS patients not adequately responding to

disease-modifying therapy have been reported to range from 7% to 49%. Natalizumab

and fingolimod are the newest US Food and Drug Administration-approved agents

that have been added to the MS treatment armamentarium, but their use is limited

by a less known safety profile and recognized specific risk. Thus, there is an

important need for new therapeutic strategies, especially those that may offer

greater patient satisfaction and safer risk profile in order to optimize

therapeutic outcomes. A number of potential therapies for MS are now in

late-stage development. Effective, safe, and well-tolerated therapies may improve

compliance and empower patients with a level of independence not presently

possible. To meet these characteristics, most of these therapies are oral

compounds. Herein, we review the pharmacology and efficacy of dimethyl fumarate

(BG-12) to date and its role in the evolving marketplace.

DOI: 10.2147/TCRM.S53285

PMCID: PMC3972027

PMID: 24707183

273. Am J Health Syst Pharm. 2014 Mar 15;71(6):463-9. doi: 10.2146/ajhp130495.

Excluded b/c no real-world adherence/persistence

Integrated clinical and specialty pharmacy practice model for management of

patients with multiple sclerosis.

Hanson RL(1), Habibi M, Khamo N, Abdou S, Stubbings J.

Author information:

(1)Rebekah L. Hanson, Pharm.D., BCPS, BCACP, is Clinical Liaison Pharmacist,

Specialty Pharmacy Services, and Clinical Assistant Professor, Department of

Pharmacy Practice, College of Pharmacy, University of Illinois at Chicago (UIC),

Chicago. Mitra Habibi, Pharm.D., is Clinical Pharmacist, Department of Neurology,

University of Illinois Hospital and Health Sciences System, Chicago, and Clinical

Assistant Professor, College of Pharmacy, UIC. Nehrin Khamo, Pharm.D., is

Clinical Staff Pharmacist, Specialty Pharmacy Services, and Clinical Instructor,

Department of Pharmacy Practice, College of Pharmacy, UIC. Sherif Abdou, B.S., is

Pharm.D. Candidate and Student Pharmacist, Specialty Pharmacy Services; and JoAnn

Stubbings, B.S.Pharm., M.H.C.A., is Assistant Director, Specialty Pharmacy

Services, and Clinical Associate Professor, Department of Pharmacy Practice,

College of Pharmacy, UIC.

PURPOSE: An integrated clinical and specialty pharmacy practice model for the

management of patients with multiple sclerosis (MS) is described.

SUMMARY: Specialty medications, such as disease-modifying therapies (DMTs) used

to treat MS, are costly and typically require special administration, handling,

and storage. DMTs are associated with high rates of nonadherence and may have

associated safety risks. The University of Illinois Hospital and Health Sciences

System developed an MS pharmacy practice model that sought to address the many

challenges of coordinating care with multiple entities outside the health system.

Several key features of the integrated model include a dedicated clinical

pharmacist on the MS specialty team, an integrated specialty pharmacy service,

direct access to the electronic medical record, and face-to-face interaction with

patients. Through the active involvement of the neurology clinical pharmacist and

an onsite specialty pharmacy service, targeted assessments and medication and

disease education are provided to the patient before DMT initiation and

maintained throughout therapy. In addition, the regular point of contact and

refill coordination encourages improved compliance, appropriate medication use,

ongoing safety monitoring, and improved communication with the provider for

quicker interventions. This fosters increased accessibility, convenience, and

patient confidence. Improving patient outcomes--the priority goal of this service

model--will be assessed in future planned studies. Through this new practice

model, providers are empowered to incorporate specialty medication management

into transitions in care, admission and discharge quality indicators,

readmissions, and other core measures.

CONCLUSION: An integrated pharmacy practice model that includes an

interdisciplinary team of physicians, nurses, and pharmacists improved patient

compliance with MS therapies.

DOI: 10.2146/ajhp130495

PMID: 24589537 [Indexed for MEDLINE]

274. BMC Neurol. 2014 Mar 13;14:49. doi: 10.1186/1471-2377-14-49.

Excluded b/c no real-world adherence/persistence

Therapy optimization in multiple sclerosis: a prospective observational study of

therapy compliance and outcomes.

Coyle PK, Cohen BA, Leist T, Markowitz C, Oleen-Burkey M(1), Schwartz M, Tullman

MJ, Zwibel H.

Author information:

(1)Health Outcomes Research Consulting and Writing, Outcomes Scribe, LLC, 664

Wynding Oaks, Kalamazoo, MI 49006, USA. OutcomesScribe@gmail.com.

BACKGROUND: Data sources for MS research are numerous but rarely provide an

objective measure of drug therapy compliance coupled with patient-reported health

outcomes. The objective of this paper is to describe the methods and baseline

characteristics of the Therapy Optimization in MS (TOP MS) study designed to

investigate the relationship between disease-modifying therapy compliance and

health outcomes.

METHODS: TOP MS was designed as a prospective, observational, nationwide

patient-focused study using an internet portal for data entry. The protocol was

reviewed and approved by Sterling IRB. The study was registered with

ClinicalTrials.gov. It captured structured survey data monthly from MS patients

recruited by specialty pharmacies. Data collection included the clinical

characteristics of MS such as MS relapses. Disability, quality of life and work

productivity and activity impairment were assessed quarterly with well-validated

scales. When events like severe fatigue or new or worsening depression were

reported, feedback was provided to treating physicians. The therapy compliance

measure was derived from pharmacy drug shipment records uploaded to the study

database. The data presented in this paper use descriptive statistics.

RESULTS: The TOP MS Study enrolled 2966 participants receiving their

disease-modifying therapy (DMT) from specialty pharmacies. The mean age of the

sample was 49 years, 80.4% were female, 89.9% were Caucasian and 55.7% were

employed full or part time. Mean time since first symptoms was 11.5 years; mean

duration since diagnosis was 9.5 years. Patient-reported EDSS was 3.5; 72.2% had

a relapsing-remitting disease course. The most commonly reported symptoms at the

time of enrollment were fatigue (74.7%), impaired coordination or balance (61.8%)

and numbness and tingling (61.2%). Half of the sample was using glatiramer

acetate and half was using beta-interferons.

CONCLUSION: Demographic and clinical characteristics of the TOP MS sample at

enrollment are consistent with other community-based MS samples, and the sample

appears to be representative of DMT users in the US. TOP MS data can be used to

explore the associations between disease-modifying therapy compliance and health

outcomes.

TRIAL REGISTRATION: ClinicalTrials.gov (NCT00819000).

DOI: 10.1186/1471-2377-14-49

PMCID: PMC3984676

PMID: 24624979 [Indexed for MEDLINE]

275. Biomed J. 2014 Mar-Apr;37(2):41-9. doi: 10.4103/2319-4170.130440.

Excluded b/c no primary data (narrative review)

Advances in the treatment of relapsing-remitting multiple sclerosis.

Tanasescu R(1), Ionete C, Chou IJ, Constantinescu CS.

Author information:

(1)Academic Clinical Neurology, Division of Clinical Neuroscience, School of

Medicine, University of Nottingham, Nottingham, England, UK; Department of

Neurology, Neurosurgery and Psychiatry, Carol Davila University of Medicine and

Pharmacy, Colentina Hospital, Bucharest, Romania, .

This article reviews and discusses the approved and emerging therapies for

multiple sclerosis (MS). MS is a chronic and disabling immune-mediated disease of

the central nervous system (CNS) that affects mainly young adults. MS imposes a

huge economic burden on healthcare systems and the society. Although the last 20

years have brought a continuous expansion in therapeutic options, there are still

unmet needs in MS management. Available MS drugs have varying degrees of efficacy

in reducing relapse risk. The long-term term effects of these treatments are

incompletely known. New therapies, along with variations of currently available

treatments, may prove more effective and tolerable than the available drugs.

Treatments for MS differ with respect to the mode of administration, tolerability

and likelihood of treatment adherence, side effects, and risk of major toxicity.

The armamentarium of approved disease-modifying therapies in MS and those in

development include: (1) the first approved, moderately effective, injectable

interferon-β and glatiramer acetate; (2) oral drugs (fingolimod, laquinimod,

teriflunomide, dimethyl fumarate); (3) monoclonal antibodies (rituximab,

ocrelizumab, ofatumumab, daclizumab, alemtuzumab); and (4) immunosuppressive

agents (e.g. mitoxantrone). The place of each drug in the therapeutic algorithm

is dependent on its specific risk-benefit profile. Patients' clinical and

paraclinical phenotypes and biomarker profile may help to elucidate disease

subtypes and response to therapy in the future, thus allowing treatment

individualization.

DOI: 10.4103/2319-4170.130440

PMID: 24732658 [Indexed for MEDLINE]

276. Neurology. 2014 Feb 25;82(8):674-80. doi: 10.1212/WNL.0000000000000137. Epub 2014

Excluded b/c no real-world adherence/persistence

Jan 24.

Pregnancy outcomes in the clinical development program of fingolimod in multiple

sclerosis.

Karlsson G(1), Francis G, Koren G, Heining P, Zhang X, Cohen JA, Kappos L,

Collins W.

Author information:

(1)From Novartis Pharma AG (G. Karlsson, P.H., W.C.), Basel, Switzerland;

Novartis Pharmaceuticals Corporation (G.F., X.Z.), East Hanover, NJ; Motherisk

Program (G. Koren), Hospital for Sick Children, Toronto, Canada; Neurological

Institute (J.A.C.), Cleveland Clinic, OH; and Neurology and Department of

Biomedicine (L.K.), University Hospital, Basel, Switzerland.

Comment in

Nat Rev Neurol. 2014 Mar;10(3):125.

Neurology. 2014 Feb 25;82(8):654-5.

OBJECTIVE: To report outcomes of pregnancies that occurred during the fingolimod

clinical development program.

METHODS: Pregnancy outcomes from phase II, phase III, and phase IV clinical

studies (with optional extensions) were reported by clinical trial investigators.

Fingolimod exposure in utero was defined as fingolimod treatment at the time of

conception or in the 6 weeks before conception.

RESULTS: As of October 31, 2011, 89 pregnancies were reported in completed or

ongoing clinical studies, with 74 in fingolimod treatment arms. Of 66 pregnancies

with in utero exposure to fingolimod, there were 28 live births, 9 spontaneous

abortions, 24 elective abortions, 4 ongoing pregnancies, and 1 pregnancy with an

unknown outcome (patient lost to follow-up). Two infants were born with

malformations: 1 with congenital unilateral posteromedial bowing of the tibia and

1 with acrania. Elective abortions were performed for 1 case each of tetralogy of

Fallot, spontaneous intrauterine death, and failure of fetal development. There

were 5 cases (7.6%; 95% confidence interval 3%-17%) of abnormal fetal development

in the 66 pregnancies that had in utero exposure to fingolimod. In all 5 cases,

fetal exposure to the drug took place in the first trimester of pregnancy.

CONCLUSIONS: The number of patients becoming pregnant during fingolimod therapy

remains small and does not permit firm conclusions to be drawn about fetal safety

of fingolimod in humans. Given the known risks of teratogenicity in animals and

the present data, women of childbearing potential should use effective

contraception during fingolimod therapy and for 2 months after discontinuation.

DOI: 10.1212/WNL.0000000000000137

PMCID: PMC3945658

PMID: 24463630 [Indexed for MEDLINE]

Excluded b/c no real-world adherence/persistence

277. PLoS One. 2014 Feb 6;9(2):e88472. doi: 10.1371/journal.pone.0088472. eCollection

2014.

Relapse rates in patients with multiple sclerosis switching from interferon to

fingolimod or glatiramer acetate: a US claims database study.

Bergvall N(1), Makin C(2), Lahoz R(1), Agashivala N(3), Pradhan A(3), Capkun

G(1), Petrilla AA(2), Karkare SU(2), McGuiness CB(2), Korn JR(2).

Author information:

(1)Novartis Pharma AG, Basel, Switzerland.

(2)IMS Health, Plymouth Meeting, Pennsylvania, United States of America.

(3)Novartis Pharmaceuticals Corporation, East Hanover, New Jersey, United States

of America.

BACKGROUND: Approximately one-third of patients with multiple sclerosis (MS) are

unresponsive to, or intolerant of, interferon (IFN) therapy, prompting a switch

to other disease-modifying therapies. Clinical outcomes of switching therapy are

unknown. This retrospective study assessed differences in relapse rates among

patients with MS switching from IFN to fingolimod or glatiramer acetate (GA) in a

real-world setting.

METHODS: US administrative claims data from the PharMetrics Plus™ database were

used to identify patients with MS who switched from IFN to fingolimod or GA

between October 1, 2010 and March 31, 2012. Patients were matched 1∶1 using

propensity scores within strata (number of pre-index relapses) on demographic

(e.g. age and gender) and disease (e.g. timing of pre-index relapse,

comorbidities and symptoms) characteristics. A claims-based algorithm was used to

identify relapses while patients were persistent with therapy over 360 days

post-switch. Differences in both the probability of experiencing a relapse and

the annualized relapse rate (ARR) while persistent with therapy were assessed.

RESULTS: The matched sample population contained 264 patients (n = 132 in each

cohort). Before switching, 33.3% of patients in both cohorts had experienced at

least one relapse. During the post-index persistence period, the proportion of

patients with at least one relapse was lower in the fingolimod cohort (12.9%)

than in the GA cohort (25.0%), and ARRs were lower with fingolimod (0.19) than

with GA (0.51). Patients treated with fingolimod had a 59% lower probability of

relapse (odds ratio, 0.41; 95% confidence interval [CI], 0.21-0.80; p = 0.0091)

and 62% fewer relapses per year (rate ratio, 0.38; 95% CI, 0.21-0.68; p = 0.0013)

compared with those treated with GA.

CONCLUSIONS: In a real-world setting, patients with MS who switched from IFNs to

fingolimod were significantly less likely to experience relapses than those who

switched to GA.

DOI: 10.1371/journal.pone.0088472

PMCID: PMC3916439

PMID: 24516663 [Indexed for MEDLINE]

278. CNS Drugs. 2014 Feb;28(2):89-94. doi: 10.1007/s40263-013-0131-5.

Excluded b/c no primary data (narrative review)

A review of safety-related pregnancy data surrounding the oral disease-modifying

drugs for multiple sclerosis.

Lu E(1), Wang BW, Alwan S, Synnes A, Dahlgren L, Sadovnick AD, Tremlett H.

Author information:

(1)Division of Neurology, Brain Research Centre and Department of Medicine,

Faculty of Medicine, University of British Columbia, Room S178, 2211 Wesbrook

Mall, Vancouver, BC, V6T 2B5, Canada.

The recent approval of several oral disease-modifying drugs (DMDs) for multiple

sclerosis (MS) brings promise of improved clinical effectiveness as well as

greater drug compliance compared to the existing non-oral DMDs, and substantially

increases patient choice and therapeutic options in the effective management of

MS. However, for men and women with MS of childbearing age, concerns about the

effect of oral DMDs on pregnancy and the fetus may arise. Some limited data from

animal reproductive studies of oral DMDs suggest a potential increased risk of

early pregnancy loss, impaired growth and birth defects. Although active

surveillance mechanisms exist, there is limited data to inform clinical practice.

Using existing information from published clinical trials and drug monographs, as

well as recent conference proceedings, this review summarizes the mechanism of

action (in relation to embryogenesis and pregnancy) and existing animal or human

pregnancy-related data for approved (fingolimod, teriflunomide and dimethyl

fumarate) and investigational (laquinimod and firategrast) oral DMDs for MS.

DOI: 10.1007/s40263-013-0131-5

PMID: 24343726 [Indexed for MEDLINE]

279. Curr Med Res Opin. 2014 Feb;30(2):251-62. doi: 10.1185/03007995.2013.849236. Epub

Excluded b/c no primary data (narrative review)

2013 Oct 22.

BG-12 (dimethyl fumarate): a review of mechanism of action, efficacy, and safety.

Fox RJ(1), Kita M, Cohan SL, Henson LJ, Zambrano J, Scannevin RH, O'Gorman J,

Novas M, Dawson KT, Phillips JT.

Author information:

(1)Mellen Center for Multiple Sclerosis Treatment and Research , Cleveland

Clinic, Cleveland, OH , USA.

BACKGROUND: Multiple sclerosis (MS) is a chronic inflammatory disease, affecting

more than 2.5 million people worldwide with more 400,000 cases in the United

States alone. There has been considerable improvement in the treatment of MS,

with the introduction of disease-modifying drugs; however, new oral therapies may

provide additional benefit by providing an alternative treatment modality and the

potential for improved adherence by avoiding the injection-associated side

effects and anxiety encountered with some first-line agents. BG-12 (dimethyl

fumarate) is an oral agent approved in the United States for the treatment of

relapsing forms of MS.

SCOPE: We review published literature about what is known about the mechanism of

action of BG-12, and key efficacy and safety findings from three clinical studies

in patients with relapsing-remitting MS (RRMS).

FINDINGS: Data from preclinical studies have demonstrated that BG-12 may promote

anti-inflammatory and cytoprotective activities that are mediated, at least in

part, by the nuclear factor (erythroid-derived 2)-like 2 (Nrf2) antioxidant

response pathway. Studies in animals have shown a protective effect of BG-12 on

neuronal, axonal and myelin integrity. Results from a phase 2 study and two

randomized double-blind placebo-controlled phase 3 studies, CONFIRM and DEFINE,

have shown that BG-12 provides clinical and radiologic efficacy in patients with

RRMS. At 2 years, BG-12 240 mg twice and three times daily reduced annualized

relapse rate (CONFIRM primary endpoint) by 44% and 51% and the risk of relapse

(DEFINE primary endpoint) by 49% and 50%, respectively, compared with placebo

(all p < 0.001). BG-12 was generally well tolerated and had an acceptable safety

profile, with a similar incidence of adverse events across treatment groups.

CONCLUSIONS: BG-12 may have cytoprotective and anti-inflammatory properties that

contribute to its efficacy among patients with RRMS. Findings from phase 2 and 3

studies further support BG-12 as an effective initial therapy. ClinicalTrials.gov

ID: NCT00168701; NCT00420212: NCT00451451.

DOI: 10.1185/03007995.2013.849236

PMID: 24131282 [Indexed for MEDLINE]

280. J Neurol. 2014 Feb;261(2):363-72. doi: 10.1007/s00415-013-7204-8. Epub 2013 Dec

Excluded b/c no real-world adherence/persistence

10.

Randomized controlled trial of physical activity, cognition, and walking in

multiple sclerosis.

Sandroff BM(1), Klaren RE, Pilutti LA, Dlugonski D, Benedict RH, Motl RW.

Author information:

(1)Department of Kinesiology and Community Health, University of Illinois at

Urbana-Champaign, 233 Freer Hall, 906 South Goodwin Ave., Urbana, IL, 61801, USA.

The present study adopted a randomized controlled trial design and examined the

effect of a physical activity behavioral intervention on cognitive and walking

performance among persons with MS who have mild or moderate disability status. A

total of 82 MS patients were randomly allocated into intervention or wait-list

control conditions. The intervention condition received a theory-based program

for increasing physical activity behavior that was delivered via the Internet,

and one-on-one video chat sessions with a behavior-change coach. Participants

completed self-report measures of physical activity and disability status, and

underwent the oral Symbol Digit Modalities Test (SDMT) and 6-minute walk (6MW)

test before and after the 6-month period. Analysis using mixed-model ANOVA

indicated a significant time × condition × disability group interaction on SDMT

scores (p = 0.02, partial-η (2) = 0.08), such that persons with mild disability

in the intervention condition demonstrated a clinically meaningful improvement in

SDMT scores (~6 point change). There was a further significant time × condition

interaction on 6MW distance (p = 0.02, partial-η (2) = 0.07), such that those in

the intervention condition demonstrated an increase in 6MW distance relative to

those in the control group. The current study supports physical activity as a

promising tool for managing cognitive impairment and impaired walking performance

in persons with MS, and suggests that physical activity might have specific

effects on cognition and non-specific effects on walking performance in this

population.

DOI: 10.1007/s00415-013-7204-8

PMID: 24323244 [Indexed for MEDLINE]

281. Mayo Clin Proc. 2014 Feb;89(2):225-40. doi: 10.1016/j.mayocp.2013.11.002.

Excluded b/c no primary data (narrative review)

Multiple sclerosis: current and emerging disease-modifying therapies and

treatment strategies.

Wingerchuk DM(1), Carter JL(2).

Author information:

(1)Department of Neurology, Mayo Clinic, Scottsdale, AZ. Electronic address:

wingerchuk.dean@mayo.edu.

(2)Department of Neurology, Mayo Clinic, Scottsdale, AZ. Electronic address:

carter.jonathan@mayo.edu.

Multiple sclerosis (MS) is a chronic inflammatory demyelinating central nervous

system disease that typically strikes young adults, especially women. The

pathobiology of MS includes inflammatory and neurodegenerative mechanisms that

affect both white and gray matter. These mechanisms underlie the relapsing, and

often eventually progressive, course of MS, which is heterogeneous; confident

prediction of long-term individual prognosis is not yet possible. However,

because revised MS diagnostic criteria that incorporate neuroimaging data

facilitate early diagnosis, most patients are faced with making important

long-term treatment decisions, most notably the use and selection of

disease-modifying therapy (DMT). Currently, there are 10 approved MS DMTs with

varying degrees of efficacy for reducing relapse risk and preserving neurological

function, but their long-term benefits remain unclear. Moreover, available DMTs

differ with respect to the route and frequency of administration, tolerability

and likelihood of treatment adherence, common adverse effects, risk of major

toxicity, and pregnancy-related risks. Thorough understanding of the benefit-risk

profiles of these therapies is necessary to establish logical and safe treatment

plans for individuals with MS. We review the available evidence supporting

risk-benefit profiles for available and emerging DMTs. We also assess the place

of individual DMTs within the context of several different MS management

strategies, including those currently in use (sequential monotherapy, escalation

therapy, and induction and maintenance therapy) and others that may soon become

feasible (combination approaches and "personalized medicine"). We conducted this

review using a comprehensive search of MEDLINE, PubMed, EMBASE, Cochrane Database

of Systematic Reviews, and Cochrane Central Register of Controlled Trials, from

January 1, 1990, to August 31, 2013. The following search terms were used:

multiple sclerosis, randomized controlled trials, interferon-beta, glatiramer

acetate, mitoxantrone, natalizumab, fingolimod, teriflunomide, dimethyl fumarate,

BG-12, alemtuzumab, rituximab, ocrelizumab, daclizumab, neutralizing antibodies,

progressive multifocal leukoencephalopathy.

Copyright © 2014 Mayo Foundation for Medical Education and Research. Published by

Elsevier Inc. All rights reserved.

DOI: 10.1016/j.mayocp.2013.11.002

PMID: 24485135 [Indexed for MEDLINE]

282. ASN Neuro. 2014 Jan 30;6(1):e00136. doi: 10.1042/AN20130037.

Excluded b/c no primary data (narrative review)

Iron chelation and multiple sclerosis.

Weigel KJ(1), Lynch SG(2), LeVine SM(3).

Author information:

(1)*Department of Biological Sciences, University of Notre Dame, Notre Dame, IN,

U.S.A.

(2)†Department of Neurology, University of Kansas Medical Center, Kansas City,

KS, U.S.A.

(3)‡Department of Molecular and Integrative Physiology, University of Kansas

Medical Center, Kansas City, KS, U.S.A.

Histochemical and MRI studies have demonstrated that MS (multiple sclerosis)

patients have abnormal deposition of iron in both gray and white matter

structures. Data is emerging indicating that this iron could partake in

pathogenesis by various mechanisms, e.g., promoting the production of reactive

oxygen species and enhancing the production of proinflammatory cytokines. Iron

chelation therapy could be a viable strategy to block iron-related pathological

events or it can confer cellular protection by stabilizing hypoxia inducible

factor 1α, a transcription factor that normally responds to hypoxic conditions.

Iron chelation has been shown to protect against disease progression and/or limit

iron accumulation in some neurological disorders or their experimental models.

Data from studies that administered a chelator to animals with experimental

autoimmune encephalomyelitis, a model of MS, support the rationale for examining

this treatment approach in MS. Preliminary clinical studies have been performed

in MS patients using deferoxamine. Although some side effects were observed, the

large majority of patients were able to tolerate the arduous administration

regimen, i.e., 6-8 h of subcutaneous infusion, and all side effects resolved upon

discontinuation of treatment. Importantly, these preliminary studies did not

identify a disqualifying event for this experimental approach. More recently

developed chelators, deferasirox and deferiprone, are more desirable for possible

use in MS given their oral administration, and importantly, deferiprone can cross

the blood-brain barrier. However, experiences from other conditions indicate that

the potential for adverse events during chelation therapy necessitates close

patient monitoring and a carefully considered administration regimen.

DOI: 10.1042/AN20130037

PMCID: PMC3906635

PMID: 24397846 [Indexed for MEDLINE]

283. BMC Neurol. 2014 Jan 14;14:11. doi: 10.1186/1471-2377-14-11.

Excluded b/c no oral DMD results

Retrospective US database analysis of persistence with glatiramer acetate vs.

available disease-modifying therapies for multiple sclerosis: 2001-2010.

Oleen-Burkey M(1), Cyhaniuk A, Swallow E.

Author information:

(1)Outcomes Scribe, LLC, 664 Wynding Oaks, Kalamazoo, MI 49006, USA.

OutcomesScribe@gmail.com.

BACKGROUND: Long-term persistence to treatment for chronic disease is difficult

for patients to achieve, regardless of the disease or medication being used. The

objective of this investigation was to examine treatment persistence with

glatiramer acetate (GA) relative to available disease-modifying therapies (DMT)

for multiple sclerosis (MS) over 12-, 24- and 36-month periods.

METHODS: Data from Clinformatics™ for DataMart affiliated with OptumInsight was

used to identify patients using DMT between 2001 and 2010. Patients with 12, 24,

and 36 months of follow-up were included. Persistence was defined as continuous

use of the same DMT for the duration of follow-up regardless of treatment gaps.

Regimen changes including re-initiation of therapy following gaps of 15 days or

more, switching therapy, and DMT discontinuation were investigated. Descriptive

statistics were used to summarize the results.

RESULTS: Cohorts of GA users with 12 months (n = 12,144), 24 months (n = 7,386)

and 36 months (n = 4,693) of follow-up were identified. Persistence rates with GA

were 80% for all time periods; discontinuation rates declined over time while

switching increased modestly. In contrast, the full DMT-treated cohorts showed

persistent rates of 68.3% at 12 months (n = 35,312), 53.9% at 24 months (n =

21,927), and 70.1% at 36 months (n = 14,343). As with these full DMT-treated

cohorts, the proportion of GA users remaining on their initial therapy without a

gap of 15 days or more decreased with length of follow-up. However, the

proportion of GA users with a gap in treatment who re-initiated GA increased over

time (64.4% at 12 months; 75.1% at 24 months, and 80.1% at 36 months) while those

in the full DMT-treated cohorts re-initiated therapy at rates of only 50-60%.

CONCLUSIONS: Persistence rates for GA were 80% for the 12-, 24- and 36-month time

periods in contrast with the full DMT-treated cohorts whose persistence rates

never exceeded 70.0%. Although there were more gaps in therapy of 15 days or more

with all DMT over time, the proportion of GA users re-initiating therapy

increased with follow-up contributing to the steady persistence. Therapy

persistence is essential to achieve the desired outcomes in MS.

DOI: 10.1186/1471-2377-14-11

PMCID: PMC3897905

PMID: 24423119 [Indexed for MEDLINE]

284. Case Rep Neurol Med. 2014;2014:307872. doi: 10.1155/2014/307872. Epub 2014 Nov

Excluded b/c no real-world adherence/persistence

23.

PML-IRIS during Fingolimod Diagnosed after Natalizumab Discontinuation.

Killestein J(1), Vennegoor A(1), van Golde AE(2), Bourez RL(3), Wijlens ML(2),

Wattjes MP(4).

Author information:

(1)Department of Neurology, MS Center Amsterdam, VU University Medical Center,

P.O. Box 7057, 1007 MB Amsterdam, The Netherlands.

(2)Department of Neurology, ZGT, Almelo, The Netherlands.

(3)Department of Radiology, ZGT, Almelo, The Netherlands.

(4)Department of Radiology, Nuclear Medicine & PET Research, MS Center Amsterdam,

VU University Medical Center, Amsterdam, The Netherlands.

Background. Natalizumab treatment is frequently discontinued and replaced by

alternative medication in multiple sclerosis (MS) patients having a high risk of

progressive multifocal leukoencephalopathy (PML). Case Presentation. We report a

PML case that was missed on magnetic resonance imaging (MRI) at the time

Natalizumab treatment was discontinued. The patient subsequently developed a

PML-immune reconstitution inflammatory syndrome after the initiation of

Fingolimod treatment, suggesting that immune reconstitution may occur even during

Fingolimod induced lymphopenia. Conclusion. This report highlights the need for

strict drug surveillance using MRI of Natalizumab-associated MS patients at the

time of drug discontinuation and beyond. This is important with respect to

pharmacovigilance purposes not only for Natalizumab, but also for alternative

drugs used after Natalizumab discontinuation.

DOI: 10.1155/2014/307872

PMCID: PMC4258922

PMID: 25506447

Excluded b/c no real-world adherence/persistence

285. Mult Scler Int. 2014;2014:752318. doi: 10.1155/2014/752318. Epub 2014 Aug 26.

Multiple Sclerosis State of the Art (SMART): A Qualitative and Quantitative

Analysis of Therapy's Adherence, Hospital Reliability's Perception, and Services

Provided Quality.

Di Battista G(1), Bertolotto A(2), Gasperini C(3), Ghezzi A(4), Maimone D(5),

Solaro C(6).

Author information:

(1)Neurology Unit, Department of Neuroscience and Sense Organs, A.C.O San Filippo

Neri, Via G. Martinotti 20, 00135 Rome, Italy.

(2)Clinical Neurobiology Unit, Regional Referring Multiple Sclerosis Centre

(CReSM), University Hospital San Luigi Gonzaga, Orbassano, 10043 Turin, Italy.

(3)Department of Neurology, A.O. San Camillo Forlanini Hospital, Circonvallazione

Gianicolense 87, 00152 Rome, Italy.

(4)Multiple Sclerosis Study Centre, Gallarate Hospital, Via Pastori 4, 21013

Gallarate, Italy.

(5)Neurology Unit, A.O. Garibaldi Nesima, Piazza Santa Maria Del Gesù 5, 95122

Catania, Catania, Italy.

(6)Neurology Unit, Department Head and Neck, ASL3 Genovese, Via D. Oliva 22,

16154 Genoa, Italy.

The purpose of this study was to assess the adherence to therapy in patients with

relapsing remitting multiple sclerosis (RR-MS) and to analyze the possible

influence of factors such as hospital care and patients socioeconomic status. Two

hundred eighty-five patients with RR-MS according to Mc Donald's criteria and

naïve disease-modifying drugs (DMDs) naïve were enrolled. Two self-administered

questionnaires addressing the management of patients at therapy prescription and

the personal perception of the daily life changes caused by DMDs were

administered at months 3 and 12. Full adherence, considered as correct use of the

therapy prescribed, was observed in a very high percentage of subjects (97.3% and

93.9% at 3 and 12 months). The main cause for reduced adherence was single dose

forgetfulness, followed by anxiety, pain at the injection site, and tiredness of

"doing all injections." Nurses and neurologists of MS Center were identified as

the major resource in coping with the disease at 3 and 12 months by patients. The

neurologist was the health professional involved in MS management in 95% of cases

and the nurse appeared to play a central role in patient training and drug

administration management (50.3%).

DOI: 10.1155/2014/752318

PMCID: PMC4158464

PMID: 25215236

286. J Neurol Sci. 2013 Dec 15;335(1-2):1-4. doi: 10.1016/j.jns.2013.08.025. Epub 2013

Excluded b/c no real-world adherence/persistence

Aug 30.

A patient's perspective of partnership in the treatment of multiple sclerosis: MS

regimes--an orchestrated approach.

von Pückler A(1).

Author information:

(1)Mendelssohnstr. 85, D-60325 Frankfurt, Germany. Electronic address:

a.pueckler@gmx.de.

Having been an MS patient for over 15 years, I will give you a short picture of

what I expect from my doctors with regard to an equilibrated

doctor-patient-relationship. Though such partnership also involves soft

components like "My doctor's understanding of my personal situation" as well as

"His understanding of my personality", this speech is limited to the hard

components "Consideration of my individual course of disease" as well as

"Provision of information and thereby educating me comprehensively". Also, I will

point to some important legal aspects of such partnership and finally will give

you some thoughts about some kind of "public partnership" in the internet.

Consideration of individual course of disease: The doctor's choice of an

appropriate treatment based on a careful and sound analysis and evaluation of the

patient's personal individual course of disease may be impaired by strict

compliance with guidelines. While guidelines may be helpful to ensure a broad

high-level patient-centered care, they bear the risk that patients are treated

according to standard programs just for the doctor to be on the safe side.

Education of the patient: I will explain how I would like to be educated by my

doctor in a perfect world. I will then elaborate that a comprehensive patient

education may prevent loss of confidence as well as allegations like biased

attitude of doctors, and may also positively influence cost pressure and

adherence to treatments. Legal aspects of partnership: It is a physician's legal

duty to educate his patients comprehensively. The right of the patients to be

educated derives from their right of self-determination, which in turn arises

directly from the patients' human dignity and personal rights. Forums and Medical

Councils of MS-Societies: Internet communities do not seem to be too happy with

their MS-Societies and experts. Statements of MS-Societies or their Medical

Councils on hot topics have sometimes been slow. Via the forums, MS-Societies may

get an idea of the questions and problems MS patients have. They could inform the

Medical Council accordingly, so that reactions regarding new developments or

surprising trial results do not take too long.

© 2013.

DOI: 10.1016/j.jns.2013.08.025

PMID: 24041734 [Indexed for MEDLINE]

287. PLoS One. 2013 Dec 10;8(12):e82796. doi: 10.1371/journal.pone.0082796.

Excluded b/c no real-world adherence/persistence

eCollection 2013.

Risk acceptance in multiple sclerosis patients on natalizumab treatment.

Tur C(1), Tintoré M, Vidal-Jordana Á, Bichuetti D, Nieto González P, Arévalo MJ,

Arrambide G, Anglada E, Galán I, Castilló J, Nos C, Río J, Martín MI, Comabella

M, Sastre-Garriga J, Montalban X.

Author information:

(1)Multiple Sclerosis Centre of Catalonia (Cemcat), Department of

Neurology-Neuroimmunology, Vall d'Hebron University Hospital, Barcelona, Spain.

OBJECTIVE: We aimed to investigate the ability of natalizumab (NTZ)-treated

patients to assume treatment-associated risks and the factors involved in such

risk acceptance.

METHODS: From a total of 185 patients, 114 patients on NTZ as of July 2011

carried out a comprehensive survey. We obtained disease severity perception

scores, personality traits' scores, and risk-acceptance scores (RAS) so that

higher RAS indicated higher risk acceptance. We recorded JC virus status

(JCV+/-), prior immunosuppression, NTZ treatment duration, and clinical

characteristics. NTZ patients were split into subgroups (A-E), depending on their

individual PML risk. Some 22 MS patients on first-line drugs (DMD) acted as

controls.

RESULTS: No differences between treatment groups were observed in disease

severity perception and personality traits. RAS were higher in NTZ than in DMD

patients (p<0.01). Perception of the own disease as a more severe condition

tended to predict higher RAS (p=0.07). Higher neuroticism scores predicted higher

RAS in the NTZ group as a whole (p=0.04), and in high PML-risk subgroups (A-B)

(p=0.02). In low PML-risk subgroups (C-E), higher RAS were associated with a JCV+

status (p=0.01). Neither disability scores nor pre-treatment relapse rate

predicted RAS in either group.

CONCLUSIONS: Risk acceptance is a multifactorial phenomenon, which might be

partly explained by an adaptive process, in light of the higher risk acceptance

amongst NTZ-treated patients and, especially, amongst those who are JCV

seropositive but still have low PML risk, but which seems also intimately related

to personality traits.

DOI: 10.1371/journal.pone.0082796

PMCID: PMC3858305

PMID: 24340060 [Indexed for MEDLINE]

Excluded b/c no primary data (narrative review)

288. Ann Pharmacother. 2013 Dec;47(12):1697-702. doi: 10.1177/1060028013509232. Epub

2013 Oct 23.

Dimethyl fumarate (Tecfidera): a new oral agent for multiple sclerosis.

Venci JV(1), Gandhi MA.

Author information:

(1)University of Rochester Medical Center/Strong Memorial Hospital, Rochester,

NY, USA.

Erratum in

Ann Pharmacother. 2014 May;48(5):668. Dosage error in article text.

Comment in

Ann Pharmacother. 2014 Nov;48(11):1537.

OBJECTIVE: To describe the clinical evidence supporting the safety, efficacy, and

clinical utility of oral dimethyl fumarate for the treatment of multiple

sclerosis (MS).

DATA SOURCES: A comprehensive PubMed search was conducted in July 2013 using the

search terms dimethyl fumarate and Tecfidera. Reference lists of abstracted

publications were reviewed to identify relevant works that were not retrieved via

the electronic search. Additional information was obtained from the FDA Web site,

manufacturer prescribing information, and Clinicaltrials.gov.

STUDY SELECTION AND DATA ABSTRACTION: Clinical trials and review articles that

included the use of dimethyl fumarate in the treatment of MS and were available

in English were abstracted for review.

DATA SYNTHESIS: The safety and efficacy of dimethyl fumarate for the treatment of

relapsing remitting MS was confirmed in 2 phase III trials, DEFINE and CONFIRM.

Relative to placebo, twice-daily dimethyl fumarate was found to reduce the

proportion of patients with relapses at 2 years by 34% to 49% and the annualized

relapse rate by 44% to 53%. Although the incidence of serious adverse effects did

not differ from that of placebo, intolerable flushing and gastrointestinal

adverse effects prompted discontinuation in 3% and 4% of patients, respectively.

CONCLUSIONS: In March 2013, dimethyl fumarate was approved as a third oral option

for patients with relapsing forms of MS. Although no head-to-head trials have

been conducted, a comparison of data from phase III trials suggests that the

efficacy of dimethyl fumarate is comparable to that of existing oral agents and

may offer a preferable safety profile.

DOI: 10.1177/1060028013509232

PMID: 24259625 [Indexed for MEDLINE]

289. Clin Neurol Neurosurg. 2013 Dec;115 Suppl 1:S6-11. doi:

Excluded b/c no oral DMD results

10.1016/j.clineuro.2013.09.013.

Adherence to disease-modifying therapies and attitudes regarding disease in

patients with multiple sclerosis.

Ožura A(1), Kovač L, Sega S.

Author information:

(1)Division of Neurology, University Medical Centre Ljubljana, Zaloška 2, 1000

Ljubljana, Slovenia.

Although currently there is no cure for MS the course of the disease can be

influenced by disease modifying therapy (DMT). For therapy to be sufficiently

efficient, it is crucial that patients take their medication regularly as

prescribed. Adherence describes the extent to which a patient acts in accordance

with the prescribed timing, dosing, and frequency of medication administration.

To date, there are no known data about adherence rates among patients with MS in

Slovenia. We wanted to assess adherence in patients with MS, who are treated with

first line DMTs and discover reasons for non-adherence. A number of 451 patients

were invited to participate. They received two questionnaires via post mail. The

adherence rate and putative reasons for non-adherence were assessed by the use of

standardized self-report Multiple Sclerosis Treatment Experience Questionnaire

(MSTEQ). Patients' attitudes regarding disease, therapy and relationship with

their physician were assessed by another questionnaire. The analysis of results

included 299 patients. Among the patients 18.5% missed at least one medication

dose in the past 28 days. Patients taking Avonex were significantly more adherent

then patients on other DMTs (p=0.005). Our study showed a higher then expected

adherence among Slovenian patients with MS (81.5%). Our research did not confirm

the influence of side effects or patients' attitudes regarding illness and

therapy on adherence. However we found unexpectedly high percentage (71.8%) of

patients belief that psychological factors are involved in MS aetiology.

Copyright © 2013 Elsevier B.V. All rights reserved.

DOI: 10.1016/j.clineuro.2013.09.013

PMID: 24321157 [Indexed for MEDLINE]

Excluded b/c no real-world adherence/persistence

290. Curr Med Res Opin. 2013 Dec;29(12):1647-56. doi: 10.1185/03007995.2013.847411.

Epub 2013 Oct 1.

Comparative effectiveness of fingolimod versus interferons or glatiramer acetate

for relapse rates in multiple sclerosis: a retrospective US claims database

analysis.

Bergvall N(1), Makin C, Lahoz R, Agashivala N, Pradhan A, Capkun G, Petrilla A,

Karkare SU, Balderston McGuiness C, Korn JR.

Author information:

(1)Novartis Pharma AG , Basel , Switzerland.

OBJECTIVE: Disease-modifying therapies, such as fingolimod, interferon (IFN) and

glatiramer acetate (GA), have differing effects on relapse rates in patients with

multiple sclerosis (MS), but little is known about the real-world differences in

relapse rates with these treatments. This retrospective study assessed relapse

rates in patients with active MS initiating fingolimod, IFN or GA therapy in a

real-world setting.

METHODS: Using administrative claims data from the US PharMetrics Plus database,

we identified previously treated and untreated patients with MS who initiated

fingolimod, IFN or GA treatment between 1 October 2010 and 31 March 2011 and had

experienced a relapse in the previous year. A claims-based algorithm was used to

identify relapses over the persistence period in patients with 540 days of

post-index continuous enrolment. A logistic regression model assessed the

probability of having at least one relapse and a generalized linear model

estimated differences in annualized relapse rates (ARRs).

RESULTS: The study enrolled 525 patients (fingolimod, n = 128; combined IFN/GA

cohort, n = 397) of the 31,041 initially identified. Similar findings for

fingolimod and IFN/GA were observed for the unadjusted proportion of patients

experiencing relapses (31.3% vs. 34.0%, respectively; p = 0.5653) and ARRs (0.50

vs. 0.55, respectively) while persistent to treatment. After adjusting for

baseline differences, fingolimod was associated with a 52% reduction in the

probability of having a relapse (odds ratio, 0.48; 95% confidence interval [CI],

0.28-0.84; p = 0.0097) and a 50% reduction in ARR (rate ratio, 0.50; 95% CI,

0.34-0.75; p = 0.0006) compared with IFN/GA.

LIMITATIONS: Identification of relapses is based on the claims in the database

rather than on a clinical assessment.

CONCLUSIONS: In a real-world setting, fingolimod was shown to be associated with

significantly lower relapse rates than IFN/GA in patients with MS who had a

history of relapses.

DOI: 10.1185/03007995.2013.847411

PMID: 24059944 [Indexed for MEDLINE]

Excluded b/c no primary data (narrative review)

291. Expert Opin Pharmacother. 2013 Dec;14(18):2545-52. doi:

10.1517/14656566.2013.848855. Epub 2013 Nov 11.

Review of laquinimod and its therapeutic potential in multiple sclerosis.

Thöne J(1), Gold R.

Author information:

(1)Ruhr-University Bochum, Department of Neurology at St. Josef-Hospital Bochum ,

Gudrunstr. 56, D-44791 Bochum , Germany +49 234 509 2411 ; +49 234 509 2414 ;

ralf.gold@rub.de.

INTRODUCTION: Multiple sclerosis (MS) is a chronic immunological disease of the

central nervous system characterized by early inflammatory demyelination and

subsequent neurodegeneration. Although major progress has occurred, MS is still

an incurable disease. Further, parenteral application and/or safety issues of the

currently licensed drugs are associated with low patient compliance. Thus, there

remains an unmet need for the development of more effective and well-tolerated

oral therapies for the treatment of MS. At this point in time, different oral

available substances are under investigation and hold promise in the treatment of

relapsing-remitting MS (RRMS).

AREAS COVERED: The physical, chemical and pharmacological properties of

laquinimod , as well as its suggested mechanisms of action, clinical efficacy and

side-effect profile are reviewed.

EXPERT OPINION: Laquinimod is a new orally administered synthetic drug designed

as an immunomodulator. Its mechanisms of action are not yet fully elucidated.

Studies in mice and humans revealed different mechanisms of action, including

anti-inflammatory and neuroprotective effects. So far, Phase II and Phase III

clinical trials have shown its efficacy on magnetic resonance imaging based

measures of disease activity, annualized relapse rate and disability progression

in RRMS patients. Current data suggest a relatively modest efficacy by measures

of relapse rate and there seems to be no superiority in comparison to established

disease-modifying agents in relapsing-remitting MS. Further studies are necessary

to evaluate both neuroprotective efficacy and optimal dosage of laquinimod in

more detail.

DOI: 10.1517/14656566.2013.848855

PMID: 24215556 [Indexed for MEDLINE]

Excluded b/c no real-world adherence/persistence

292. Med Devices (Auckl). 2013 Nov 15;6:175-84. doi: 10.2147/MDER.S52590. eCollection

2013.

ExtaviJect® 30G device for subcutaneous self-injection of interferon beta-1b for

multiple sclerosis: a prospective European study.

Boeru G(1), Milanov I, De Robertis F, Kozubski W, Lang M, Rojas-Farreras S,

Tomlinson M.

Author information:

(1)Military Hospital, Bucharest, Romania.

BACKGROUND: The ExtaviJect® 30G autoinjector was developed to facilitate

parenteral self-administration of interferon beta-1b (Extavia®), a first-line

disease-modifying therapy in patients with multiple sclerosis. Our aim was to

assess patient compliance with treatment when using the autoinjector, patients'

and nurses' experiences of using the device, its tolerability, and patient

satisfaction.

METHODS: This was a 12-week, real-world, prospective, observational,

noninterventional study conducted in nine European countries. Questionnaires were

used to measure patient compliance and to assess patients' and nurses'

experiences. All adverse events were recorded by severity, including injection

site reactions or pain. Patient satisfaction and health-related quality of life

were assessed using the Treatment Satisfaction Questionnaire for Medication-9

(TSQM-9) and EuroQol-5 Dimension (EQ-5D) instruments, respectively.

RESULTS: Of 582 patients enrolled, 568 (98%) received at least one injection and

attended the first follow-up visit at 6 weeks, and 542 (93%) attended the second

follow-up visit at 12 weeks. For the whole study, 548 of 568 (97%) patients were

compliant with treatment. Among the various questions assessing whether the

device was easy and quick to use accurately, without fear of the needle, 56%-98%

of patients and 59%-98% of nurses were in agreement. There were nine serious

adverse events (four disease-related) reported among the 227 (39%) patients

reporting adverse events. Scores increased in the TSQM-9 convenience domain

between weeks 6 and 12 (P=0.0009), and in the EQ-5D visual analog scale between

baseline and week 12 (P<0.0001), indicating improvement in health-related quality

of life.

CONCLUSION: ExtaviJect 30G was convenient to use and was associated with high

levels of compliance.

DOI: 10.2147/MDER.S52590

PMCID: PMC3832381

PMID: 24255602

Excluded b/c no primary data (narrative review)

293. Am J Manag Care. 2013 Nov;19(17 Suppl):s343-54.

An update on new and emerging therapies for relapsing-remitting multiple

sclerosis.

Weinstock-Guttman B(1).

Author information:

(1)WBWeinstock-Guttman@KaleidaHealth.org.

Disease-modifying therapies (DMTs), known to actively reduce relapses and delay

disability progression, have been used for the treatment of relapsing-remitting

multiple sclerosis (RRMS) for over a decade. These well-known therapies include

intramuscular (IM) interferon (IFN) beta-1a (Avonex), subcutaneous (SC) IFN

beta-1a (Rebif), SC IFN beta- 1b (Betaseron; Extavia), and SC glatiramer acetate

(Copaxone). These first-line therapies have shown only partial benefits for

controlling multiple sclerosis (MS) disease activity and are often associated

with inadequate patient adherence. Low patient adherence to therapy may be

related to the mode of administration or to the side effects associated with

treatment. The intravenous DMT natalizumab (Tysabri; dosed monthly) provides high

therapeutic efficacy and good compliance but is considered a second-line

intervention because of the associated increased risk for progressive multifocal

leukoencephalopathy. In 2010, fingolimod (Gilenya), the first oral DMT, was

approved by the US Food and Drug Administration (FDA) for the treatment of MS.

Recently, 2 new oral DMTs received FDA approval for the treatment of RRMS:

teriflunomide (Aubagio) and dimethyl fumarate (Tecfidera). In addition, oral

laquinimod, several monoclonal antibodies (eg, alemtuzumab, daclizumab, and

ocrelizumab), and other agents have shown preliminary beneficial results in

relapsing MS in phase 3 clinical trials. These new and emerging DMTs may provide

a more efficacious individualized therapeutic approach, more favorable methods of

administration (eg, oral administration), and/or a lower frequency of infusions

(eg, annually, 3-5 daily infusions over a year for alemtuzumab) that may improve

patient adherence and clinical outcomes.

PMID: 24494635 [Indexed for MEDLINE]

Excluded b/c no primary data (narrative review)

294. Am J Manag Care. 2013 Nov;19(17 Suppl):s321-31.

Impact of diagnosis and early treatment on the course of multiple sclerosis.

Noyes K, Weinstock-Guttman B(1).

Author information:

(1)BWeinstock-Guttman@KaleidaHealth.org.

Multiple sclerosis (MS) is a progressive inflammatory disease of the central

nervous system that results in neurological dysfunction and disability. The

initiation of disease-modifying therapy (DMT) early in the course of MS may

improve the prognosis for patients with MS and reduce the occurrence of

neurological damage. In patients with relapsing-remitting MS (RRMS), DMT reduces

the rate of relapses, reduces the appearance of magnetic resonance imaging

markers of disease activity, and slows the course of disability progression. DMT

has been shown to be more effective when initiated early in the course of MS. In

patients who have not yet developed clinically definite MS (CDMS), but have had 1

attack of neurological symptoms consistent with MS (ie, clinically isolated

syndrome [CIS]), the initiation of DMT (specifically, interferon beta, glatiramer

acetate, and teriflunomide) following this attack has been shown to delay the

conversion to CDMS. Current guidelines have recognized the benefits of early

treatment of MS with DMTs. However, there are a number of barriers to

implementing early MS treatment. Early diagnosis and treatment of MS can be

hindered because patients may delay consulting a physician about their

neurological symptoms or may be reluctant to start DMT. Further, even after

initiating DMT, continued adherence to treatment is often poor. These delays in

treatment and a lack of adherence to treatment are associated with poor patient

outcomes. The objectives of this review are to highlight the importance of early

diagnosis and treatment of CIS or RRMS and discuss the favorable outcomes

associated with early initiation of DMT.

PMID: 24494633 [Indexed for MEDLINE]

295. BMC Neurol. 2013 Oct 4;13:138. doi: 10.1186/1471-2377-13-138.

Compliance to fingolimod and other disease modifying treatments in multiple

sclerosis patients, a retrospective cohort study.

Agashivala N(1), Wu N, Abouzaid S, Wu Y, Kim E, Boulanger L, Brandes DW.

Author information:

(1)Novartis Pharmaceuticals Corporation, One Health Plaza, 07936, East Hanover,

NJ USA. neetu.agashivala@novartis.com.

BACKGROUND: Adherence to disease-modifying therapies (DMTs) results in the

reduction of the number and severity of relapses and delays the progression of

multiple sclerosis (MS). Patients with lower adherence rates experience more

inpatient visits and higher MS-related medical costs. Fingolimod, the first oral

DMT approved by the US Food and Drug Administration, may improve the access and

compliance to MS treatment when compared to injectable DMTs.

METHODS: This retrospective cohort study used pharmacy claims from Medco Health

Solutions, Inc., of patients who initiated DMTs between October 2010 and February

2011. Initiation was defined as no prescription fills for the same DMT in the

prior 12 months. Patients without a DMT prescription fill 12 months before the

index date were considered naïve users. Compliance was measured via proportion of

days covered (PDC) and medication possession ratio (MPR) for 12 months

post-index. Discontinuation was defined as a ≥60-day gap of index DMT supply. Cox

proportional hazard models compared time to discontinuation between cohorts.

RESULTS: Of 1,891 MS patients (mean age: 45.7; female: 76.4%), 13.1% initiated

fingolimod, 10.7% interferon beta-1b, 20.0% intramuscular interferon beta-1a,

18.8% subcutaneous interferon beta-1a, and 37.4% glatiramer acetate. Patients

initiating fingolimod had highest average PDC and MPR in both experienced

(fingolimod: mean PDC=0.83, 73.7% with PDC≥0.8; mean MPR=0.92, 90.5% with

MPR≥0.8) and naïve DMT users (fingolimod: mean PDC=0.80, 66.7% with PDC≥0.8; mean

MPR=0.90, 87.4% with MPR≥0.8). The proportion of patients discontinuing index DMT

within 12 months was significantly lower for the fingolimod cohort (naïve: 31.3%;

experienced: 25.7%). Adjusted results found that patients receiving self-injected

DMTs discontinued significantly sooner than fingolimod users. This association

was generally stronger in experienced DMT users.

CONCLUSIONS: Fingolimod initiators were more compliant, less likely to

discontinue treatment, and discontinued later than patients who initiated

self-injected DMT.

DOI: 10.1186/1471-2377-13-138

PMCID: PMC3851325

PMID: 24093542 [Indexed for MEDLINE]

296. Clin Ther. 2013 Oct;35(10):1501-12. doi: 10.1016/j.clinthera.2013.07.330.

Excluded b/c no oral DMD results

Treatment patterns in disease-modifying therapy for patients with multiple

sclerosis in the United States.

Bonafede MM(1), Johnson BH, Wenten M, Watson C.

Author information:

(1)Truven Health Analytics, Cambridge, MA. Electronic address:

machaon.bonafede@truvenhealth.com.

BACKGROUND: Patients with multiple sclerosis (MS) whose disease activity is

inadequately controlled with a platform therapy (interferon beta or glatiramer

acetate [GA]) may switch to another platform therapy or escalate therapy to

natalizumab or fingolimod, which were approved in the US in 2006 and 2010,

respectively.

OBJECTIVE: The objective of this study was to describe treatment patterns in

patients with multiple sclerosis (MS) in the United States who were followed for

2 years after initiating a disease-modifying therapy (DMT).

METHODS: A retrospective observational cohort study was conducted to examine

treatment patterns of initial DMT use (on initial therapy for 2 years with and

without gaps of ≥ 60 days, medication switching, and discontinuation) among

patients with MS who initiated a platform therapy (interferon-β or glatiramer

acetate) or natalizumab between January 1, 2007, and September 30, 2009; the

first DMT claim was the index. Eligible patients were identified in the

MarketScan Commercial and Medicare Supplemental databases based on continuous

enrollment for 6 months before (preindex period) and 24 months after their index

date, with a diagnosis of MS and no claim for a previous DMT in the 6-month

preindex period. Demographics at index and clinical characteristics during the

preindex period were also analyzed.

RESULTS: A total of 6181 MS patients were included, with 5735 (92.8%) starting on

platform therapy. Natalizumab initiators were more likely to stay on index

therapy (32.3% vs 16.9%, P < 0.001) and have fewer treatment gaps of ≥ 60 days

(44.8% vs 55.3%, P < 0.001) compared with platform initiators. In addition,

natalizumab initiators were less likely to switch treatment (13.9% vs 19.1%, P =

0.007) and took longer to switch (400.9 days vs 330.7 days, P < 0.001) compared

with platform initiators. Nearly 79% of platform initiators who switched went to

another platform therapy. Approximately two thirds of patients who switched to a

third DMT (n = 130) switched to another platform therapy. A total of 9% of

natalizumab and platform initiators discontinued DMT within the 2 years.

CONCLUSIONS: Most MS patients initiating DMT started on platform therapy.

Natalizumab initiators tended to stay on index therapy, have fewer treatment

gaps, and switch less than platform initiators in the 2 years after treatment

initiation. Switching between platform therapies is common despite evidence that

MS patients on platform therapy may benefit from switching to natalizumab.

© 2013 Elsevier HS Journals, Inc. All rights reserved.

DOI: 10.1016/j.clinthera.2013.07.330

PMID: 24139422 [Indexed for MEDLINE]

297. Ann Pharmacother. 2013 Sep;47(9):1153-60. doi: 10.1177/1060028013500647.

Excluded b/c no primary data (systematic review)

Teriflunomide for the treatment of relapsing multiple sclerosis: a review of

clinical data.

Brunetti L(1), Wagner ML, Maroney M, Ryan M.

Author information:

(1)Rutgers, The State University of New Jersey, Piscataway, NJ, USA.

OBJECTIVE: To review the pharmacology and clinical data for teriflunomide in

relapsing multiple sclerosis (MS).

DATA SOURCES: A literature search from 1966 to May 2013 using PubMed/MEDLINE, Web

of Science, International Pharmaceutical Abstracts, Academic Search Premiere,

Science Citation Index, and the national clinical trials registry was performed

using the terms teriflunomide, HMR1726, and A771726. All articles containing

human clinical trial data and relevant pharmacologic information were reviewed.

STUDY SELECTION/DATA EXTRACTION: Phase 2 and phase 3 clinical trials for

teriflunomide were evaluated. All peer-reviewed articles with clinically relevant

information were reviewed. Priority for inclusion was placed on randomized

controlled trials.

DATA SYNTHESIS: Three phase 2 and three phase 3 clinical trials have evaluated

teriflunomide as monotherapy or as adjunctive therapy in approximately 3000

patients with relapsing forms of MS. The phase 3 studies used annualized relapse

rate, magnetic resonance imaging changes, and Expanded Disability Status Scale

scores as outcome measures. One additional Phase 3 clinical study is ongoing. The

annualized relapse rates and magnetic resonance imaging findings were improved

compared to those with placebo and similar to or improved compared with those

with subcutaneously administered interferon-β-1a 44 µg thrice weekly. Durability

of response is supported by open-label extension studies. Common adverse events

include increased liver function enzymes, alopecia, diarrhea, influenza, nausea,

and paresthesias. Treatment discontinuation was not common and occurred in

approximately 10% of patients in phase 3 studies.

CONCLUSIONS: Teriflunomide is an effective and safe oral treatment option for

relapsing MS. It can be used as monotherapy or added to an interferon or

glatiramer acetate. It reduces the rate of relapse and may slow disease

progression. The advantages of this drug are the convenience of oral

administration and good tolerability. The disadvantage is the lack of long-term

safety data and data about the benefit of combination therapy.

DOI: 10.1177/1060028013500647

PMID: 24259730 [Indexed for MEDLINE]

298. Clin Drug Investig. 2013 Sep;33(9):647-52. doi: 10.1007/s40261-013-0108-7.

Excluded b/c no real-world adherence/persistence

Impact of disease-modifying therapies on the survival of patients with multiple

sclerosis in Taiwan, 1997-2008.

Tsai CP(1), Lee CT.

Author information:

(1)Neurology, Neurological Institute, Taipei Veterans General Hospital and

National Yang-Ming University, Taipei, Taiwan, ROC. cptsai@vghtpe.gov.tw

BACKGROUND: Little is known about the impact of disease-modifying therapies

(DMTs) on the survival of patients with multiple sclerosis (MS) throughout the

world.

OBJECTIVE: We conducted this study to investigate the association between DMTs

and the survival of patients with MS in Taiwan.

METHODS: A total of 1,240 individuals who had a primary diagnosis of MS and a

seriously disabling disease certificate in Taiwan between 1 January 1997 and 1

December 2008 were followed up until 31 December 2009 to check what medical

services were provided to them and whether they had a date of death recorded in

the national mortality database. Disease-modifying therapies, including

interferon beta 1-a, interferon beta 1-b and glatiramer acetate, were included in

the analysis. Follow-up information was available on all individuals; the mean

follow-up time was 54.3 months (standard deviation [SD] 38.8 months). A Cox

regression model was utilized to reveal the effect of DMTs on MS mortality by

controlling for sex, age, residence, insurance amount and geographic region.

RESULTS: Eighty-eight of the 1,240 individuals (7.1 %) died. The risk of

mortality in the first year showed a 7-fold age- and sex-standardized mortality

rate increase over that of the general population in Taiwan. In the fully

adjusted model, the final independent risk factors were older age, rural

residence, lower economic status and lower adherence to DMTs.

CONCLUSION: The results of this study support the notion that DMTs can improve

the survival of patients with MS, and show that individuals with the risk factors

of older age, rural residence and lower economic status had a higher MS-related

mortality risk in Taiwan.

DOI: 10.1007/s40261-013-0108-7

PMID: 23861171 [Indexed for MEDLINE]

299. J Med Econ. 2013 Sep;16(9):1146-53. doi: 10.3111/13696998.2013.823868. Epub 2013

Excluded b/c no oral DMD results

Aug 8.

Using observational analysis of multiple sclerosis relapse to design

outcomes-based contracts for disease-modifying drugs: a feasibility assessment.

Lage MJ(1), Carroll CA, Fairman KA.

Author information:

(1)Health Metrics Outcomes Research , LLC, Delray Beach, FL, USA.

OBJECTIVE: To assess predictors and costs of multiple sclerosis (MS) relapse, a

potential outcome measure in payer-manufacturer risk-sharing agreements for

disease-modifying drugs (DMDs).

METHODS: A retrospective cohort analysis of medical/pharmacy claims was used.

Study patients had ≥1 DMD (interferon beta, glatiramer, natalizumab) claim,

without DMD claims in a 6-month pre-period before DMD initiation; were aged 18-64

years and continuously enrolled from the pre-period through a 24-month

post-period; and had ≥2 MS medical claims during the 30-month study period.

Post-period relapse cohorts included: (1) severe (hospitalization with MS

diagnosis); (2) moderate (outpatient services including intravenous

methylprednisolone); and (3) none. Poisson regression modeled severe relapse

frequency, logistic regression modeled ≥1 severe relapse, and generalized linear

modeling predicted healthcare costs. Tested predictors included demographics,

insurance type, index DMD, pre-period health status, and DMD medication

possession ratio (MPR).

RESULTS: Severe relapse was experienced by 14.5% and moderate relapse by 13.8% of

2291 patients. In logistic regression, severe relapse was predicted by plan type;

age (odds ratio [OR] = 1.018, 95% confidence interval [CI] = 1.005-1.031);

pre-period Charlson Comorbidity Index (OR = 1.307, 95% CI = 1.166-1.464);

pre-period proxy measure indicating impaired activities of daily living

(OR = 1.470, 95% CI = 1.134-1.905); pre-period MS hospitalization (OR = 2.174,

95% CI = 1.537-3.074); and DMD non-adherence (MPR OR = 0.101, 95%

CI = 0.068-0.151). Poisson regression results were similar. Predicted mean

[standard deviation] all-cause healthcare expenditures were tripled for patients

with severe compared with moderate relapse ($48,173 [$8665] and $13,334 [$1929],

respectively).

LIMITATIONS: Commercially insured patients from a single payer; use may have been

inconsistent with approved indications; proxy relapse measure may have

misclassified patients.

CONCLUSIONS: Severe MS relapses requiring hospitalization, although affecting

less than 15% of patients initiating DMD treatment, are associated with high

medical costs. The only actionable predictor of severe relapse identified in

observational analysis was MPR, raising questions about the feasibility of using

observational data to guide outcomes-based contracting.

DOI: 10.3111/13696998.2013.823868

PMID: 23844620 [Indexed for MEDLINE]

300. PM R. 2013 Sep;5(9):747-56. doi: 10.1016/j.pmrj.2013.04.022. Epub 2013 May 3.

Excluded b/c no real-world adherence/persistence

Adherence associated with oral medications in the treatment of spasticity.

Halpern R(1), Gillard P, Graham GD, Varon SF, Zorowitz RD.

Author information:

(1)OptumInsight, Health Economics and Outcomes Research, 12125 Technology Drive,

Eden Prairie, MN 55344(∗). Electronic address: rachel.halpern@optum.com.

OBJECTIVE: To examine adherence to baclofen, tizanidine, and dantrolene (U.S.

Food and Drug Administration-approved oral spasticity medications), and

identified determinants of adherence.

DESIGN: A retrospective administrative claims data analysis that used medical and

pharmacy claims data and enrollment information from a large, national U.S.

health plan.

SUBJECTS AND METHODS: The subjects were commercial health plan members who

initiated treatment on baclofen, tizanidine, or dantrolene from January 1, 2004,

through September 30, 2009, and who had stroke, spinal cord injury, traumatic

brain injury, cerebral palsy, or multiple sclerosis. Descriptive and logistic

regression statistical analyses were performed.

MAIN OUTCOME MEASUREMENTS: Outcomes were adherence, measured as continuous

medication possession ratio (MPR) and as a binary indicator (MPR ≥0.80, adherent;

MPR <0.80, nonadherent), change in oral spasticity medication, and use of nonoral

spasticity therapy.

RESULTS: The study population included 2840 subjects. Adherence overall was poor:

the range of mean unadjusted MPR values was 0.10-0.50, which indicated that, at

best, the subjects were adherent to their index spasticity medications for 50% of

their treatment periods. Unadjusted overall MPRs for baclofen and tizanidine were

20.4% and 9.1%, respectively. Fewer than 5% of subjects changed oral spasticity

medications. The results of logistic regression to identify determinants of

adherence showed that subjects treated with tizanidine versus baclofen had 37.4%

lower odds of adherence and that subjects with traumatic brain injury versus

stroke had 77.5% lower odds of adherence. The odds of adherence increased with

age and with preindex contracture or decubitus ulcer.

CONCLUSIONS: Adherence to oral spasticity medication was poor irrespective of

index spasticity medication or condition. Results from this study indicated that

physicians cannot assume that patients are adherent to prescribed oral spasticity

medications. A more complete understanding of the reasons behind nonadherence is

required.

Copyright © 2013 American Academy of Physical Medicine and Rehabilitation.

Published by Elsevier Inc. All rights reserved.

DOI: 10.1016/j.pmrj.2013.04.022

PMID: 23648652 [Indexed for MEDLINE]

301. Ther Adv Chronic Dis. 2013 Sep;4(5):192-205. doi: 10.1177/2040622313492810.

Excluded b/c no primary data (narrative review)

Teriflunomide in relapsing multiple sclerosis: therapeutic utility.

Freedman MS(1).

Author information:

(1)Department of Medicine (Neurology), University of Ottawa, Senior Scientist,

The Ottawa Hospital Research Institute, 501 Smyth Road, Ottawa, ON, Canada K1H

8L6.

Teriflunomide is an oral, once-daily disease-modifying therapy (DMT) approved in

the USA, Australia, and Argentina for the treatment of relapsing forms of

multiple sclerosis (RMS). Teriflunomide reversibly limits the expansion of

activated T and B cells associated with the inflammatory process purportedly

involved in multiple sclerosis pathogenesis, while preserving lymphocytes for

routine immune surveillance. In an extensive clinical development program,

teriflunomide demonstrated consistent benefits on both clinical and magnetic

resonance imaging outcomes. In long-term studies, teriflunomide treatment was

associated with low rates of relapse and disability progression for up to 8

years. The safety profile of teriflunomide has been well characterized, with

adverse events generally mild to moderate in nature and infrequently leading to

permanent treatment discontinuation. The evidence reviewed here indicates that

teriflunomide is an effective addition to the current DMTs used to treat RMS.

DOI: 10.1177/2040622313492810

PMCID: PMC3752181

PMID: 23997924

302. Acta Neurol Scand. 2013 Aug;128(2):e6-e10. doi: 10.1111/ane.12082. Epub 2013 Jan

Excluded b/c no real-world adherence/persistence

22.

Switching from natalizumab to fingolimod: an observational study.

Sempere AP(1), Martín-Medina P, Berenguer-Ruiz L, Pérez-Carmona N, Sanchez-Perez

R, Polache-Vengud J, Feliu-Rey E.

Author information:

(1)Neurology Department, Hospital General Universitario de Alicante, Alicante,

Spain. aperezs@mac.com

BACKGROUND: Multiple sclerosis patients who discontinue using natalizumab are at

risk of a rebound in disease activity. However, the optimal alternative therapy

is not currently known.

AIMS OF THE STUDY: We report on clinical and MRI data and patient safety in a

group of relapsing-remitting multiple sclerosis patients who tested seropositive

for the JC virus and who have switched from natalizumab to fingolimod because of

concerns regarding PML risks.

METHODS: The test for JC virus antibodies was performed in 18 relapsing-remitting

multiple sclerosis patients who were being treated with natalizumab for more than

1 year. Eight seropositive patients switched to fingolimod while the seronegative

patients continued with natalizumab.

RESULTS: After switching to fingolimod, five of eight patients (63%) experienced

clinical relapses, and MRI activity was detected in six of eight patients (75%).

Neither clinical relapses nor MRI activity was observed in the patients who

continued with natalizumab. No serious adverse effects were detected.

CONCLUSIONS: Natalizumab is an effective treatment for relapsing-remitting

multiple sclerosis, but its discontinuation continues to be a complex problem.

All of the therapies tried thus far, including fingolimod, have been unable to

control the reactivation of the disease. Further studies addressing alternative

therapies after natalizumab discontinuation are necessary.

© 2013 John Wiley & Sons A/S.

DOI: 10.1111/ane.12082

PMID: 23336398 [Indexed for MEDLINE]

Excluded b/c no primary data (narrative review)

303. Expert Opin Pharmacother. 2013 Aug;14(11):1559-67. doi:

10.1517/14656566.2013.804912. Epub 2013 May 22.

Dimethyl fumarate : a Janus-faced substance?

Kees F(1).

Author information:

(1)University of Regensburg, Faculty of Chemistry and Pharmacy, Department of

Pharmacology and Toxicology, Universitätsstraße 31, 93053 Regensburg, Germany.

frieder.kees@ur.de

INTRODUCTION: Dimethyl fumarate (DMF) has been used as fungicide, but oral DMF

activates anti-inflammatory and anti-oxidative pathways that are beneficial in

the treatment of psoriasis. BG-12, a specific formulation of DMF, has been

approved very recently for the treatment of relapsing-remitting multiple

sclerosis (RRMS), which is characterized by both autoimmune lymphocytes leading

to inflammation and mitochondrial alterations associated with oxidative stress.

AREAS COVERED: This review describes the pharmacokinetics and the mode of action

of DMF, with a focus on molecular and cellular pathways, and discusses clinical

results of DMF in RRMS treatment. To identify relevant publications, the author

searched the PubMed database by using appropriate keywords and by searching for

references cited within the obtained articles.

EXPERT OPINION: DMF demonstrated efficacy in several RRMS outcome measures

related to disease activity and severity, but results on disability progression

have been inconsistent. The overall safety profile might qualify DMF for

long-term use, the frequency of side effects such as gastrointestinal complaints

and flushing might hamper treatment adherence of MS patients. Since DMF

covalently binds to intracellular proteins, the fate of this molecule in the body

might need thorough long-term observation during clinical use.

DOI: 10.1517/14656566.2013.804912

PMID: 23697607 [Indexed for MEDLINE]

304. J Med Econ. 2013 Aug;16(8):1036-42. doi: 10.3111/13696998.2013.811079. Epub 2013

Excluded b/c no real-world adherence/persistence

Jun 12.

A discrete choice experiment to determine patient preferences for injection

devices in multiple sclerosis.

Shingler SL(1), Swinburn P, Ali S, Perard R, Lloyd AJ.

Author information:

(1)Oxford Outcomes, An ICON plc Company, Oxford, UK.

OBJECTIVE: Relapsing-remitting multiple sclerosis (MS) is usually managed with

disease modifying drugs (DMDs), most commonly administered via self-injection.

The aim of this study was to estimate the influence that different

treatment-related attributes have for MS patients on their choice of MS DMD

device. By establishing the relative importance of these characteristics for

patients it should be possible to better understand the acceptability of a given

device and to optimize the development of future devices.

METHODS: A discrete choice experiment (DCE) survey was developed on the basis of

a review of published literature. Attributes identified for inclusion in the

survey were: ease of use; comfort of use; presence of additional functions,

needle visibility; practicality and efficacy. Choice sets were presented as pairs

of hypothetical treatments based upon a fractional factorial design. One-hundred

device-using MS patients completed the survey online. Analysis was conducted

using a mixed-logit approach.

RESULTS: Analysis of the DCE data revealed that all attributes significantly

predicted treatment choice. Efficacy exhibited the largest effect on treatment

selection and this provided context for understanding the magnitude of impact for

the other attributes. Reducing the discomfort associated with device use and

eliminating the necessity for assembly or drug reconstitution were highly valued.

The addition of reminder and time-stamping functions, improved needlestick injury

prevention, and reduction in device size were secondary concerns but still deemed

desirable.

CONCLUSION: Efficacy is of primary importance to MS patients, but characteristics

of drug delivery devices can play an important role in treatment decision-making.

Not all device characteristics could be included, and results are based upon 100

participants only. Findings suggest there is significant potential value in

developing self-injection devices that are not only efficacious but also

convenient and comfortable to use. Reducing barriers to adherence could

potentially translate into improved treatment outcomes for patients with MS.

DOI: 10.3111/13696998.2013.811079

PMID: 23730944 [Indexed for MEDLINE]

305. Mult Scler Relat Disord. 2013 Jul;2(3):256-8. doi: 10.1016/j.msard.2012.11.004.

Excluded b/c no real-world adherence/persistence

Epub 2013 Jan 16.

Severe early bilateral macular edema following fingolimod therapy.

Coppes OJ(1), Gutierrez I(2), Reder AT(3), Ksiazek S(3), Bernard J(3).

Author information:

(1)Pritzker School of Medicine, University of Chicago, 5841 South Maryland

Avenue, Chicago Il, 60637, USA. Electronic address: ocoppes@uchicago.edu.

(2)School of Medicine, Ross University, Commonwealth of Dominica, West Indies.

(3)University of Chicago, Chicago, USA.

We report a case of bilateral macular edema (ME) within 10 days of starting

fingolimod 0.5mg therapy in a patient with Multiple Sclerosis (MS). The

complication resolved without treatment as demonstrated by sequential Optical

Coherence Tomography (OCT). Fingolimod is a sphingosine-1-phosphate receptor

modulator that reduces lymphocyte presence in the CNS. In pivotal trials, ME, a

known complication of fingolimod, typically occurred unilaterally with onset at

approximately 3 months. A 60y/o AA female, diagnosed with MS in 1977, started

oral fingolimod treatment on 05/31/2011. Baseline screening with OCT and

ophthalmology evaluation showed no ME. On 06/10, she developed bilateral blurry

vision and discontinued fingolimod. On 06/27, OCT revealed severe bilateral ME.

Later OCT exams showed a progressive decrease in Central Foveal Thickness (CFT)

and Macular Volume (MV), without specific treatment other than discontinuation of

fingolimod. On 7/27, CFT, MV, and Visual Acuity (VA) were similar to baseline.

This is the first reported case of bilateral, early onset ME following fingolimod

treatment at the current FDA-approved dose of 0.5mg. Diabetes, a known risk

factor for ME, may have contributed to her early, bilateral involvement. Our case

provides further support for earlier OCT, in conjunction with ophthalmic

examinations, for at-risk patients on fingolimod, and suggests that cessation of

fingolimod may be associated with resolution of ME.

Copyright © 2012 Elsevier B.V. All rights reserved.

DOI: 10.1016/j.msard.2012.11.004

PMID: 25877733

306. Patient Prefer Adherence. 2013 Jun 12;7:509-16. doi: 10.2147/PPA.S40736. Print

Excluded b/c no oral DMD results

2013.

Medication possession ratio: implications of using fixed and variable observation

periods in assessing adherence with disease-modifying drugs in patients with

multiple sclerosis.

Kozma CM(1), Dickson M, Phillips AL, Meletiche DM.

Author information:

(1)CK Consulting Associates, LLC St Helena Island, SC.

BACKGROUND: The purpose of this study was to compare two methods of adherence

calculation using administrative data for patients with multiple sclerosis (MS)

who are prescribed disease-modifying drugs.

METHODS: Pharmacy-billed disease-modifying drug prescription claims were selected

from the 2007-2008 LifeLink™ Health Plan Claims Database. The index date was the

first disease-modifying drug prescription claim. Two cohorts were created: all

patients with a disease-modifying drug claim in 2007 and a subset with continuous

eligibility for 12 months post-index. Adherence was calculated across all

disease-modifying drugs for 12 months post-index. Medication possession ratios

(MPRs) with variable (start to end of therapy) and fixed (365 days) duration

denominators were calculated. Variable MPR was calculated by summing days supply

from the first to the last prescription (inclusive) divided by time between the

last prescription date plus days supply and the first prescription date. Variable

MPR was evaluated for all patients and the continuously eligible cohort. Fixed

MPR used the same numerator but divided by 365 days of follow-up and evaluated

only for the continuously eligible cohort.

RESULTS: There were 3405 patients with MS and a disease-modifying drug claim in

2007 and 2145 in the continuously eligible cohort. Means for variable MPR ranged

from 87.5% ± 16.6% for the continuously eligible cohort to 90.5% ± 16.0% for the

2007 cohort. The comparable value for fixed MPR was 78.0% ± 28.2% for the

continuously eligible cohort. Fixed MPR gave a consistently lower rate of

adherence than variable MPR at an 80% adherence threshold.

CONCLUSION: Different adherence measures can yield different outcomes, especially

when using different eligibility criteria. These results demonstrate the

importance of full disclosure of methods used for calculations and specification

of the study population.

DOI: 10.2147/PPA.S40736

PMCID: PMC3685450

PMID: 23807840

307. PLoS One. 2013 May 21;8(5):e63480. doi: 10.1371/journal.pone.0063480. Print 2013.

Excluded b/c no oral DMD results

Persistence on therapy and propensity matched outcome comparison of two

subcutaneous interferon beta 1a dosages for multiple sclerosis.

Kalincik T(1), Spelman T, Trojano M, Duquette P, Izquierdo G, Grammond P,

Lugaresi A, Hupperts R, Cristiano E, Van Pesch V, Grand'maison F, La Spitaleri D,

Rio ME, Flechter S, Oreja-Guevara C, Giuliani G, Savino A, Amato MP, Petersen T,

Fernandez-Bolanos R, Bergamaschi R, Iuliano G, Boz C, Lechner-Scott J, Deri N,

Gray O, Verheul F, Fiol M, Barnett M, van Munster E, Santiago V, Moore F, Slee M,

Saladino ML, Alroughani R, Shaw C, Kasa K, Petkovska-Boskova T, den

Braber-Moerland L, Chapman J, Skromne E, Herbert J, Poehlau D, Needham M, Bacile

EA, Arruda WO, Paine M, Singhal B, Vucic S, Cabrera-Gomez JA, Butzkueven H;

MSBase Study Group.

Collaborators: Roger E, Despault P, Marriott M, Van der Walt A, King J,

Kilpatrick T, Buzzard K, Jokubaitis V, Byron J, Morgan L, Skibina O, Haartsen J,

De Luca G, Di Tommaso V, Travaglini D, Pietrolongo E, di Ioia M, Farina D,

Mancinelli L, Paolicelli D, Iaffaldano P, Ignacio Rojas J, Patrucco L, Roullet E,

Correale J, Ysrraelit C, Cartechini E, Pucci E, Williams D, Dark L, Shaygannejad

V, Zwanikken C, Vella N, Sirbu CA.

Author information:

(1)Departments of Medicine and Neurology, University of Melbourne and Royal

Melbourne Hospital, Melbourne, Australia. tomas.kalincik@unimelb.edu.au

OBJECTIVES: To compare treatment persistence between two dosages of interferon

β-1a in a large observational multiple sclerosis registry and assess disease

outcomes of first line MS treatment at these dosages using propensity scoring to

adjust for baseline imbalance in disease characteristics.

METHODS: Treatment discontinuations were evaluated in all patients within the

MSBase registry who commenced interferon β-1a SC thrice weekly (n = 4678).

Furthermore, we assessed 2-year clinical outcomes in 1220 patients treated with

interferon β-1a in either dosage (22 µg or 44 µg) as their first disease

modifying agent, matched on propensity score calculated from pre-treatment

demographic and clinical variables. A subgroup analysis was performed on 456

matched patients who also had baseline MRI variables recorded.

RESULTS: Overall, 4054 treatment discontinuations were recorded in 3059 patients.

The patients receiving the lower interferon dosage were more likely to

discontinue treatment than those with the higher dosage (25% vs. 20% annual

probability of discontinuation, respectively). This was seen in discontinuations

with reasons recorded as "lack of efficacy" (3.3% vs. 1.7%), "scheduled stop"

(2.2% vs. 1.3%) or without the reason recorded (16.7% vs. 13.3% annual

discontinuation rate, 22 µg vs. 44 µg dosage, respectively). Propensity score was

determined by treating centre and disability (score without MRI parameters) or

centre, sex and number of contrast-enhancing lesions (score including MRI

parameters). No differences in clinical outcomes at two years (relapse rate, time

relapse-free and disability) were observed between the matched patients treated

with either of the interferon dosages.

CONCLUSIONS: Treatment discontinuations were more common in interferon β-1a 22 µg

SC thrice weekly. However, 2-year clinical outcomes did not differ between

patients receiving the different dosages, thus replicating in a registry dataset

derived from "real-world" database the results of the pivotal randomised trial.

Propensity score matching effectively minimised baseline covariate imbalance

between two directly compared sub-populations from a large observational

registry.

DOI: 10.1371/journal.pone.0063480

PMCID: PMC3660604

PMID: 23704913 [Indexed for MEDLINE]

308. Drugs. 2013 May;73(7):625-50. doi: 10.1007/s40265-013-0030-6.

Excluded b/c no primary data (narrative review)

Drugs in development for relapsing multiple sclerosis.

Ali R(1), Nicholas RS, Muraro PA.

Author information:

(1)Division of Brain Sciences, Imperial College London, Burlington Danes

Building, 160 Du Cane Road, London W12 0NN, UK.

Drug development for multiple sclerosis (MS), as with any other neurological

disease, faces numerous challenges, with many drugs failing at various stages of

development. The disease-modifying therapies (DMTs) first introduced for MS are

only moderately effective, but given the lack of competition, they have been

widely accepted in clinical practice. Although safety and efficacy continue to be

the two main metrics by which drugs will be judged, the newer agents in the

market also face challenges of a more comparative nature-are they more

efficacious than the currently available drugs on the market? Are they safer or

better tolerated? Do they offer any practical advantages over current treatments?

Fingolimod represented a milestone following its approval as an oral drug for MS

in 2010, offering patients a far more convenient administration route. However,

association with cardiovascular complications has led to a more cautious approach

in its initial prescribing, now requiring cardiac monitoring for the first 6 h as

well as subsequent monitoring of blood pressure and for macular oedema.

Natalizumab, amongst licensed drugs, represents the current benchmark for

efficacy. The risk of progressive multifocal leukoencephalopathy during

natalizumab treatment is now more quantifiable. Other monoclonal antibodies are

in various phases of development. Marketing authorisation for alemtuzumab has

been filed, and whilst trial data suggest that its efficacy outperforms both

licensed drugs and others in development, there is a significant risk of

secondary autoimmunity. Its once-yearly administration, however, seems

particularly advantageous. Rituximab is unlikely to be developed further as its

license will expire, but ocrelizumab, another monoclonal antibody directly

targeting B cells, is currently in phase 2 development and looks promising.

Daclizumab is also moderately efficacious but may struggle to establish itself

given its monthly subcutaneous dosing. There are new oral drugs in development,

and it is likely that BG-12 will be licensed this year. This has been licensed

for psoriasis so there are good safety data in humans that may also hold true in

MS; however, its three times daily dosage will probably impact on patient

compliance. Laquinimod has lower efficacy than BG-12 but appears safe and could

find a place as a first-line agent. Teriflunomide has just been licensed by the

US FDA and may challenge the current injectable first-line therapies as it has a

similar efficacy but the advantage of being taken orally. However, risk of

teratogenicity may caution against its use in some women of child-bearing

potential. This review will examine drugs that have been recently approved as

well as those that are in late phase 2 or 3 development as treatment for

relapsing MS, highlighting their mechanism of action as well as the clinical

trial and safety data before discussing their potential for success in an

increasingly florid and complex DMT armamentarium.

DOI: 10.1007/s40265-013-0030-6

PMID: 23609782 [Indexed for MEDLINE]

Excluded b/c no primary data (narrative review)

309. Front Neurol. 2013 May 1;4:10. doi: 10.3389/fneur.2013.00010. eCollection 2013.

How does fingolimod (gilenya(®)) fit in the treatment algorithm for highly active

relapsing-remitting multiple sclerosis?

Fazekas F(1), Bajenaru O, Berger T, Fabjan TH, Ledinek AH, Jakab G, Komoly S,

Kobys T, Kraus J, Kurča E, Kyriakides T, Lisý L, Milanov I, Nehrych T, Moskovko

S, Panayiotou P, Jazbec SŠ, Sokolova L, Taláb R, Traykov L, Turčáni P, Vass K,

Vella N, Voloshyná N, Havrdová E.

Author information:

(1)Department of Neurology, Medical University of Graz Graz, Austria.

Multiple sclerosis (MS) is a neurological disorder characterized by inflammatory

demyelination and neurodegeneration in the central nervous system. Until

recently, disease-modifying treatment was based on agents requiring parenteral

delivery, thus limiting long-term compliance. Basic treatments such as

beta-interferon provide only moderate efficacy, and although therapies for

second-line treatment and highly active MS are more effective, they are

associated with potentially severe side effects. Fingolimod (Gilenya(®)) is the

first oral treatment of MS and has recently been approved as single

disease-modifying therapy in highly active relapsing-remitting multiple sclerosis

(RRMS) for adult patients with high disease activity despite basic treatment

(beta-interferon) and for treatment-naïve patients with rapidly evolving severe

RRMS. At a scientific meeting that took place in Vienna on November 18th, 2011,

experts from ten Central and Eastern European countries discussed the clinical

benefits and potential risks of fingolimod for MS, suggested how the new therapy

fits within the current treatment algorithm and provided expert opinion for the

selection and management of patients.

DOI: 10.3389/fneur.2013.00010

PMCID: PMC3640198

PMID: 23641231

310. Int J Neurosci. 2013 May;123(5):324-8. doi: 10.3109/00207454.2012.759569. Epub

Excluded b/c no real-world adherence/persistence

2013 Feb 4.

Endovascular treatment of chronic cerebrospinal venous insufficiency in multiple

sclerosis: a retrospective study.

Alroughani R(1), Lamdhade S, Thussu A.

Author information:

(1)Division of Neurology, Department of Medicine, Amiri Hospital, Kuwait City,

Kuwait. raed.alroughani@dasmaninstitute.org

BACKGROUND: Chronic cerebrospinal venous insufficiency (CCSVI) has been proposed

to be associated with multiple sclerosis (MS). Zamboni et al. reported

significant improvement in neurological outcomes in MS patients who underwent

percutaneous transluminal angioplasty (PTA).

OBJECTIVES: To retrospectively evaluate the neurological outcomes in MS patients

who underwent PTA.

METHOD: Relapsing remitting MS patients who underwent PTA and completed at least

1 year post-PTA were assessed. Patients with clinically isolated syndrome or

progressive forms of MS were excluded. Primary endpoint was the proportion of

relapse-free patients at 1 year. Secondary endpoints were change in mean Expanded

Disability Status Scale (EDSS) score and proportion of patients with new magnetic

resonance imaging (MRI) activity (defined as either gadolinium-enhancing or new

T2 lesions) at 1 year.

RESULTS: Forty-five patients satisfied the inclusion criteria. Females

constituted 71.1%. The mean age and mean disease duration were 33.76 and 7.16

years, respectively. At 1-year post-PTA, the proportion of relapse-free patients

decreased from 84.44% to 66.67% (p = 0.085), whereas the mean EDSS score

increased (p = 0.017). The proportion of patients with new MRI activity increased

significantly from 17.78% to 44.44% (p = 0.012). A total of 35.6% of patients

stopped their disease modifying therapies (DMTs). There was no difference among

the patients who stopped their DMTs with respect to relapses, EDSS score or new

MRI activity.

CONCLUSION: The study revealed that PTA in relapsing remitting MS patients was

not associated with any neurological improvement. However, there was an increase

in disease activity irrespective of the adherence to DMTs. Further evidence of

the association between CCSVI and MS is required.

DOI: 10.3109/00207454.2012.759569

PMID: 23301864 [Indexed for MEDLINE]

311. J Neurol. 2013 May;260(5):1382-7. doi: 10.1007/s00415-012-6808-8. Epub 2012 Dec

Excluded b/c no real-world adherence/persistence

25.

Fingolimod reduces recurrence of disease activity after natalizumab withdrawal in

multiple sclerosis.

Havla J(1), Tackenberg B, Hellwig K, Meinl I, Krumbholz M, Seitz F, Eienbröker C,

Gold R, Hohlfeld R, Kleiter I, Kümpfel T.

Author information:

(1)Institute of Clinical Neuroimmunology, Medical Campus Grosshadern,

Ludwig-Maximilians-University, Marchioninistr. 15, 81377 Munich, Germany.

joachim.havla@med.lmu.de

After discontinuation of natalizumab (NAT), multiple sclerosis (MS) disease

activity often recurs. We assessed the recurrence of clinical disease activity

during the first year after switching from NAT to fingolimod (FTY) in patients

with relapsing-remitting MS. The number of relapses and the annualized relapse

rate (ARR) before, during and after NAT discontinuation were determined and

compared between 26 MS patients who switched to FTY within 24 weeks, and 10 MS

patients who remained without disease modifying therapy (therapy free group =

TFG). Median follow-up post-NAT discontinuation was 55.1 weeks. In a subgroup (n

= 20), the occurrence of contrast-enhancing-lesions (Gd+) on magnetic resonance

imaging (MRI) was determined. Eleven patients (42 %) in the FTY group and seven

patients (70 %) in the TFG had one or more relapses after cessation of NAT during

follow-up (p < 0.05). One of the 11 (9 %) patients in the FTY group and 6/9 (67

%) patients in the TFG showed Gd+ lesions during follow-up (p < 0.05). Patients

who switched to FTY ≤ 12 weeks after NAT discontinuation (n = 9) showed a trend

for a lower post-NAT ARR compared to patients who started FTY therapy >12 weeks

after NAT was stopped (n = 17). Most relapses in the FTY group occurred just

before or within 8 weeks after starting FTY. Our observation suggests that

initiation of FTY treatment after NAT discontinuation reduces the recurrence of

disease activity compared to withdrawal without further immunomodulatory

treatment. In the FTY group the ARR tended to depend on the time interval between

discontinuation of NAT and initiation of FTY.

DOI: 10.1007/s00415-012-6808-8

PMID: 23266894 [Indexed for MEDLINE]

312. Patient Prefer Adherence. 2013 Apr 16;7:309-18. doi: 10.2147/PPA.S41992. Print

Excluded b/c no real-world adherence/persistence

2013.

A cross-sectional survey of patient satisfaction and subjective experiences of

treatment with fingolimod.

Hanson KA(1), Agashivala N, Stringer SM, Balantac Z, Brandes DW.

Author information:

(1)United BioSource Corporation, Dorval, QC, Canada.

BACKGROUND: Fingolimod is the first oral disease-modifying therapy indicated for

the treatment of patients with relapsing forms of multiple sclerosis (MS) to

reduce the frequency of clinical relapses and delay the progression of physical

disability caused by MS.

OBJECTIVE: To obtain data from MS patients who have taken fingolimod regarding

their treatment choice, first-dose observation (FDO) experience, and treatment

satisfaction.

METHODS: Patients ≥ 18 years old with physician-diagnosed MS in the United States

who had taken at least one dose of fingolimod for the treatment of MS were

invited to complete a web-based survey, which captured information on the reasons

for starting fingolimod, FDO experience, and treatment satisfaction as measured

by the Treatment Satisfaction Questionnaire for Medication (TSQM). A high TSQM

scale score denotes high satisfaction.

RESULTS: Survey respondents (n = 380; 55% female) had a mean (standard deviation)

age of 39.8 (12.6) years, and a mean (standard deviation) duration of MS of 9.8

(10.3) years. Overall, more than 80% of patients reported the first dose was

moderately/very/extremely manageable, convenient, and easy to take. Although 80%

of patients reported experiencing a side effect with the first dose, most were

highly tolerable and only eleven patients (2.9%) reported they were "Not at all"

satisfied with the FDO experience. TSQM scale scores were highest for the side

effect (79.4), followed by convenience (71.7), effectiveness (70.1), and global

satisfaction (68.9) domains; relatively higher scores were observed among

treatment-experienced patients. Both treatment-naïve and treatment-experienced

patients indicated physician recommendation as the primary reason for starting

fingolimod. Among treatment-experienced respondents (n = 273), 58% reported that

their first choice for MS treatment would be fingolimod if selecting today.

CONCLUSION: Most fingolimod patients were satisfied with their FDO experience.

Satisfaction with fingolimod was high and observed higher among

treatment-experienced compared to treatment-naïve patients. Additional research

is needed to understand key clinical and medication attributes underlying

treatment satisfaction with fingolimod and other disease-modifying therapies.

DOI: 10.2147/PPA.S41992

PMCID: PMC3633540

PMID: 23620662

313. Int J MS Care. 2013 Spring;15(1):36-45. doi: 10.7224/1537-2073.2011-038.

Excluded b/c no primary data (narrative review)

Facilitating medication adherence in patients with multiple sclerosis.

Remington G(1), Rodriguez Y(1), Logan D(1), Williamson C(1), Treadaway K(1).

Author information:

(1)UT Southwestern MS Center, Dallas, TX, USA.

This article reviews adherence to medication in multiple sclerosis (MS) patients

from the perspective of nurse and social worker authors. It reviews data on

patient adherence and offers practical, evidence-based strategies that

health-care providers can use to facilitate adherence. In addition, it examines

how emerging MS therapies may affect patient adherence and associated

interventions. To promote adherence, interventions need to incorporate new and

creative approaches. A proactive approach includes assessing patient needs and

lifestyle before the start of medication and selecting the most appropriate

disease-modifying therapy for each individual patient. Including

multidisciplinary expertise and services in the treatment plan can be part of a

comprehensive, holistic approach to helping patients and families. Optimization

of health-care provider roles is likely to facilitate improved adherence.

DOI: 10.7224/1537-2073.2011-038

PMCID: PMC3883032

PMID: 24453761

314. JAMA Neurol. 2013 Apr;70(4):469-75. doi: 10.1001/jamaneurol.2013.923.

Excluded b/c pediatric patients

Natalizumab therapy for highly active pediatric multiple sclerosis.

Kornek B(1), Aboul-Enein F, Rostasy K, Milos RI, Steiner I, Penzien J, Hellwig K,

Pitarokoili K, Storm van's Gravesande K, Karenfort M, Blaschek A, Meyer A, Seidl

R, Debelic D, Vass K, Prayer D, Kristoferitsch W, Bayas A.

Author information:

(1)Department ofNeurology, Medical University of Vienna, WaehringerGuertel 18-20,

A-1090 Vienna, Austria. barbara.bajer-kornek@meduniwien.ac.at

IMPORTANCE: Given the high frequency of failure of first-line therapies, there is

an urgent need for second-line treatment strategies for pediatric patients with

multiple sclerosis (MS).

OBJECTIVE: To report the use of natalizumab in pediatric MS. Natalizumab, a

humanized monoclonal antibody targeting α4 integrin, is effective against active

relapsing-remitting MS in adults.

DESIGN: Retrospective study.

SETTING: Eleven centers for neurology and pediatric neurology in Germany and

Austria.

PARTICIPANTS: A total of 20 pediatric patients with MS who started treatment with

natalizumab prior to 18 years of age. These patients underwent magnetic resonance

imaging as clinically indicated, despite the fact that 19 of these 20 patients

were undergoing first-line disease-modifying therapy. The mean (SD) age at

initiation of natalizumab therapy was 16.7 (1.1) years, and the mean (SD)

pretreatment period was 18 (10) months.

INTERVENTION: Natalizumab, 300 mg every 4 weeks.

MAIN OUTCOME MEASURES: Annualized relapse rates, Expanded Disability Status Scale

scores, number of new T2/fluid-attenuated inversion recovery lesions and

contrast-enhancing lesions on magnetic resonance imaging, number of adverse

events, the prevalence of neutralizing antibodies against natalizumab, and serum

JC virus-antibody status. RESULTS Treatment with natalizumab was associated with

reductions in mean annualized relapse rates (3.7 without treatment vs 0.4 with

treatment; P < .001), median Expanded Disability Status Scale scores (2 without

treatment vs 1 with treatment; P < .02), and mean number of new

T2/fluid-attenuated inversion recovery lesions per year (7.8 without treatment vs

0.5 with treatment; P < .001). Two patients developed high-titer neutralizing

antibodies against natalizumab and had to stop therapy. Adverse events included

headaches, asthenia, infections, and hypersensitivity. Abnormal laboratory

results were found for 8 patients. JC virus antibodies were found in 5 of 13

patients. After the discontinuation of natalizumab therapy, relapse activity

occurred in 6 of 8 patients within 6 months.

CONCLUSIONS AND RELEVANCE: Our data indicate that natalizumab may be safe and

effective against MS in pediatric patients with breakthrough disease.

DOI: 10.1001/jamaneurol.2013.923

PMID: 23420110 [Indexed for MEDLINE]

315. Expert Opin Drug Deliv. 2013 Mar;10(3):285-7. doi: 10.1517/17425247.2013.763793.

Excluded b/c no primary data (narrative review)

Epub 2013 Jan 23.

Improving adherence to injectable disease-modifying drugs in multiple sclerosis.

Bayas A(1).

Author information:

(1)Department of Neurology, D-86156 Augsburg, Germany.

antonios.bayas@klinikum-augsburg.de

Comment on

Expert Opin Drug Deliv. 2013 Feb;10(2):273-83.

Multiple sclerosis (MS) is the most common neurological disorder leading to

permanent disability in early adulthood and premature pension. Prophylactic

treatment should be initiated early in the disease process to prevent permanent

disability at best. Injectable drugs, i.e., interferon beta preparations and

glatiramer acetate, belong to the first-line therapeutics in MS. Autoinjection

devices, available for all disease-modifying drugs, can improve injection

tolerability and patient's satisfaction compared with manual injection. In one

study, the use of an autoinjector has been found to be the strongest predictor of

adherence at 24 months. Nevertheless, long-term adherence to injectable MS

therapies is generally suboptimal for various barriers with an impact on efficacy

of these treatments. Improvements concerning drug delivery by innovative features

could be achieved by the first electronic autoinjector RebiSmart(™) and may help

to increase adherence. Patients' rating of the new electronic device and

short-term adherence data convey some evidence that patients' treatment

satisfaction and adherence may increase by its use. Long-term studies, already

under way, are awaited to show, if long-term adherence will be positively

influenced. A further achievement of the new electronic device is the possibility

to monitor adherence objectively allowing a discussion between the patient and

health care provider about nonadherence reasons. The necessity of improving

adherence to injectable disease-modifying drugs within the growing field of oral

MS therapeutics will also be discussed.

DOI: 10.1517/17425247.2013.763793

PMID: 23339371 [Indexed for MEDLINE]

316. Acta Neurol Scand. 2013 Feb;127(2):133-40. doi: 10.1111/j.1600-0404.2012.01708.x.

Excluded b/c no oral DMD results

Epub 2012 Aug 25.

Stoppers and non-starters of disease-modifying treatment in multiple sclerosis.

Grytten N(1), Aarseth JH, Espeset K, Johnsen GB, Wehus R, Lund C, Haugstad RC.

Author information:

(1)National Multiple Sclerosis Competence Centre, Department of Neurology,

Haukeland University Hospital, Bergen, Norway.

nina.grytten.torkildsen@helse-bergen.no

OBJECTIVES: To explore the frequency of non-starters and stoppers of

disease-modifying therapy (DMT) in a cohort of people recently diagnosed with

multiple sclerosis (MS) and to identify reasons for non-starting or stopping DMT

measured by demographic variables, social support [The Interpersonal Support

Evaluation List (ISEL)] and disease-related stress [The Impact of Event Scale

(IES)].

MATERIALS AND METHODS: A multicentre retrospective cohort study using postal

surveys completed by people with MS was performed, comprising all patients

diagnosed with MS during 2000-2007 at four university clinics in Norway.

RESULTS: Of the 424 respondents, 180 (42%) were still using the first prescribed

DMT, 83 (20%) were using DMT after switching DMT at least once, 53 (12.5%) had

ended DMT, and 108 (25.5%) had never started DMT. The risk of non-starting DMT

was associated with increasing age at diagnosis, the region, disease-related

stress and avoidant trauma coping. The risk factors for stopping therapy after

the first prescribed DMT were adverse events and high education.

CONCLUSIONS: Disease-related stress, avoidant trauma coping, age at diagnosis and

education should be considered when motivating people with MS to use DMT. Hence,

the challenges to starting and continuing treatment will probably also remain a

problem with orally administered DMT.

© 2012 John Wiley & Sons A/S.

DOI: 10.1111/j.1600-0404.2012.01708.x

PMID: 22924678 [Indexed for MEDLINE]

317. Expert Opin Drug Deliv. 2013 Feb;10(2):273-83. doi: 10.1517/17425247.2013.746311.

Excluded b/c no primary data (narrative review)

Epub 2012 Dec 20.

RebiSmart™ (version 1.5) device for multiple sclerosis treatment delivery and

adherence.

Lugaresi A(1).

Author information:

(1)Department of Neuroscience and Imaging, University G. d'Annunzio, c/o Centro

Sclerosi Multipla, Ospedale Clinicizzato SS Annunziata, Via dei Vestini 31, 66100

Chieti, Italy. a.lugaresi@unich.it

Comment in

Expert Opin Drug Deliv. 2013 Mar;10(3):285-7.

INTRODUCTION: First-line disease-modifying drugs for the treatment of multiple

sclerosis (MS) are mostly administered by injection. Although these treatments

can control the symptoms and progression of the disease to some extent, patients

often fail to adhere to their therapy in the long term, so may not obtain the

maximum clinical benefits. As injection-related problems are common barriers to

adherence, autoinjectors have been developed to improve the ease and convenience

of self-injection.

AREAS COVERED: This article discusses RebiSmart™, an electronic autoinjector for

the subcutaneous administration of interferon β-1a, focusing on the device

features and data from clinical trials of the device. An overview of other

available autoinjectors for first-line MS therapies is provided for context.

EXPERT OPINION: The device is the first electronic autoinjector for use in MS and

offers several innovative features, including adjustable injection settings and

an electronic dosing log, which may improve adherence. The dosing log can be

reviewed with the patient, allowing the physician to open a dialogue to discuss

possible issues with treatment adherence. The use of multidose cartridges also

provides a softer impact on the environment and easier disposal. The hidden

needle helps avoid needle phobia and reduces the risk of needle stick injury.

DOI: 10.1517/17425247.2013.746311

PMID: 23252744 [Indexed for MEDLINE]

318. Drug Healthc Patient Saf. 2013;5:37-47. doi: 10.2147/DHPS.S28822. Epub 2013 Feb

Excluded b/c no primary data (narrative review)

26.

Oral available agents in the treatment of relapsing remitting multiple sclerosis:

an overview of merits and culprits.

Thöne J(1), Ellrichmann G.

Author information:

(1)Department of Neurology, St JosefHospital Bochum, Ruhr-University Bochum,

Bochum, Germany.

Multiple sclerosis (MS) is a chronic immunological disease of the central nervous

system characterized by early inflammatory demyelination and subsequent

neurodegeneration. Major therapeutic progress has occurred during the past

decade, in particular since the introduction of immunomodulatory agents, however,

MS is still an incurable disease. In addition, parenteral application of the

currently licensed drugs is associated with injection-related adverse events

(AEs) and low patient compliance. Thus, there remains an unmet need for the

development of more effective and well tolerated oral therapies for the treatment

of MS. A number of new orally administered agents including fingolimod,

laquinimod, teriflunomide, cladribine, and BG-12 have been licensed recently or

are currently under investigation in relapsing remitting MS patients. In

multi-center, randomized, placebo-controlled phase III clinical studies, all of

these agents have already shown their efficacy on both clinical disease

parameters and magnetic resonance imaging-based measures of disease activity in

patients with relapsing remitting MS. However, there are essential differences

concerning their clinical efficacy and side-effect profiles. Additionally, the

mechanisms by which these substances exert clinical efficacy have not been fully

elucidated. In this article, we review the pharmaceutical properties of

fingolimod, laquinimod, teriflunomide, cladribine, and BG-12; and their suggested

mechanisms of action, clinical efficacy, and side-effect profiles.

DOI: 10.2147/DHPS.S28822

PMCID: PMC3585507

PMID: 23459383

Excluded b/c no primary data (narrative review)

319. Expert Opin Pharmacother. 2013 Jan;14(1):123-36. doi:

10.1517/14656566.2013.754012. Epub 2012 Dec 21.

Cladribine tablets for the treatment of relapsing-remitting multiple sclerosis.

Comi G(1), Hartung HP, Kurukulasuriya NC, Greenberg SJ, Scaramozza M.

Author information:

(1)University Vita-Salute San Raffaele, Department of Neurology and Institute of

Experimental Neurology, Via Olgettina 60, 20132 Milan, Italy. g.comi@hsr.it

Comment in

Expert Opin Pharmacother. 2013 Jan;14(1):1-3.

INTRODUCTION: Multiple sclerosis (MS) is a chronic immune-mediated disorder of

the central nervous system leading to progressive neurodegeneration and

disability. Until 2010, all approved disease-modifying drugs for MS required

parenteral administration, which is associated with suboptimal adherence. It was

anticipated that new approaches to treatment, including oral agents such as

cladribine tablets, may improve adherence. In 2011, the development of cladribine

tablets was stopped following negative feedback from the EMA and FDA.

AREAS COVERED: This article provides an overview of the chemistry, mechanism of

action and pharmacological properties of cladribine tablets therapy, and

highlights the rationale for its development as an oral treatment for MS. Key

efficacy and safety data from the pivotal Phase III CLARITY study are presented,

providing context for the opinion received from the regulatory agencies.

EXPERT OPINION: Despite the promising efficacy data observed in the cladribine

tablets clinical trial program, regulatory agencies identified a potential risk

of increased malignancies, and raised concerns about the implications of

sustained lymphocyte depletion. Following the feedback received from the

regulatory agencies, Merck Serono made the decision to withdraw the agent from

the regulatory approval process. The experience gained will benefit other

research efforts to address the outstanding unmet treatment needs of patients

with relapsing MS.

DOI: 10.1517/14656566.2013.754012

PMID: 23256518 [Indexed for MEDLINE]

320. Int J MS Care. 2013 Winter;15(4):194-201. doi: 10.7224/1537-2073.2012-034.

Excluded b/c no oral DMD results

Treatment discontinuation and disease progression with injectable

disease-modifying therapies: findings from the north american research committee

on multiple sclerosis database.

Fox RJ(1), Salter AR(1), Tyry T(1), Sun J(1), You X(1), Laforet G(1), Campagnolo

D(1).

Author information:

(1)Mellen Center for Multiple Sclerosis, Neurological Institute, and the

Cleveland Clinic Lerner College of Medicine, Cleveland Clinic, Cleveland, OH, USA

(RJF); the University of Alabama at Birmingham, Birmingham, AL, USA (ARS); Barrow

Neurological Institute of St. Joseph's Hospital and Medical Center, Phoenix, AZ,

USA (TT); Biogen Idec Inc, Weston, MA, USA (JS, XY, GL, DC); the University of

Massachusetts Medical School, Worcester, MA, USA (GL); and the University of

Arizona College of Medicine, Phoenix, AZ, USA (DC).

Injectable first-line disease-modifying therapies (DMTs) for multiple sclerosis

(MS) are generally prescribed for continuous use. Accordingly, the various

factors that influence patient persistence with treatment and that can lead some

patients to switch medications or discontinue treatment may affect clinical

outcomes. Using data from the North American Research Committee on Multiple

Sclerosis (NARCOMS) database, this study evaluated participants' reasons for

discontinuation of injectable DMTs as well as the relationship between staying on

therapy and sustained patient-reported disease progression and annualized relapse

rates. Participants selected their reason(s) for discontinuation from among 16

possible options covering the categories of efficacy, safety, tolerability, and

burden, with multiple responses permitted. Both unadjusted data and data adjusted

for baseline age, disease duration, disability, and sex were evaluated.

Discontinuation profiles varied among DMTs. Participants on intramuscular

interferon beta-1a (IM IFNβ-1a) and glatiramer acetate (GA) reported the fewest

discontinuations based on safety concerns, although GA was associated with

reports of higher burden and lower efficacy than other therapies. Difficulties

with tolerability were more often reported as a reason for discontinuing

subcutaneous (SC) IFNβ-1a than as a reason for discontinuing IM IFNβ-1a, GA, or

SC IFNβ-1b. In the persistent therapy cohort, less patient-reported disability

progression was reported with IM IFNβ-1a treatment than with SC IFNβ-1a, IFNβ-1b,

or GA. These findings have relevance to clinical decision making and medication

compliance in MS patient care.

DOI: 10.7224/1537-2073.2012-034

PMCID: PMC3883017

PMID: 24453783

321. J Manag Care Pharm. 2013 Jan-Feb;19(1 Suppl A):S41-53.

Excluded b/c no primary data (narrative review)

Perspectives for managed care organizations on the burden of multiple sclerosis

and the cost-benefits of disease-modifying therapies.

Owens GM(1), Olvey EL, Skrepnek GH, Pill MW.

Author information:

(1)Gary Owens Associates, 5 Palmer Drive, Glen Mills, PA 19342. USA.

gowens99@comcast.net

Disease-modifying therapies (DMTs) are a core component of multiple sclerosis

(MS) management. Given current constraints on health care expenditures, the

relative cost-effectiveness of these therapies needs to be considered when making

treatment decisions. The objective of this article is to review the burden of

illness of MS, discuss the cost-effectiveness data for DMTs, and summarize the

implications for payers. For the burden of illness in MS, a retrospective

analysis of managed care administrative data from the IMS LifeLink Health Plan

Claims Database was performed. Data from claims submitted for patients with

confirmed MS (ICD-9-CM code 340) over a period of 1 year (2009) were analyzed. A

literature review was conducted to put these data into perspective. The

retrospective analysis determined that the mean annual cost of treating MS in the

United States in 2009 was $23,434, which varied according to the presence of

comorbidities/complications. Overall, DMTs accounted for 69% of the total costs

of managing the disease. According to the literature review, the typical

first-line DMTs (interferon beta [IFNβ] formulations and glatiramer acetate [GA])

are generally associated with incremental cost-utility or cost-effectiveness

ratios in excess of $100,000 per quality of life year gained. Natalizumab may

have cost benefits over other agents in patients with more aggressive disease.

According to the available data, studies indicate that DMT cost-effectiveness

(specifically cost per quality-adjusted life years) appears to improve with

treatment initiation during the early stages of the disease. In

relapsing-remitting MS, there is currently little evidence to differentiate

between the DMTs that are typically used first-line (IFNs and GA) based on

cost-effectiveness or cost-utility studies. Presently, optimal therapy decisions

for DMT-naïve patients are likely to be made individually based on patient and

provider preference, adherence, and medication risk-benefit profiles. For

patients with more advanced disease, natalizumab appears to have greater efficacy

and to be more cost-effective than other agents.

DOI: 10.18553/jmcp.2013.19.s1.S41

PMID: 23383732 [Indexed for MEDLINE]

322. J Manag Care Pharm. 2013 Jan-Feb;19(1 Suppl A):S24-40.

Excluded b/c no primary data (narrative review)

Narrative review of the literature on adherence to disease-modifying therapies

among patients with multiple sclerosis.

Menzin J(1), Caon C, Nichols C, White LA, Friedman M, Pill MW.

Author information:

(1)Boston Health Economics, Inc., 20 Fox Road, Waltham, MA 0245, USA.

jmenzin@bhei.com

While no curative treatment exists for multiple sclerosis (MS), several

disease-modifying therapies (DMTs) have been developed to reduce relapse rates,

slow disability progression, and modify the overall disease course. However,

because of the chronic nature of the disease, long-term therapy adherence can be

challenging for some patients with MS. Low adherence to DMTs has been shown to be

associated with higher rates of disease relapses and progression as well as with

an increase in medical resource utilization. As new MS treatments are developed,

a comprehensive understanding of current adherence rates and the impact of

adherence on clinical and economic outcomes is of particular interest. Our

objective was to conduct a review of the published literature to evaluate rates

of adherence to DMTs in MS and the impact of adherence on both clinical and

economic outcomes from the patient and payer perspectives. Systematic literature

searches were conducted using MEDLINE, EMBASE, and the Cochrane Central Register

for Controlled Trials. Studies were limited to those completed on human subjects,

written in the English language, and published between May 1, 2001, and May 1,

2011. Additional inclusion criteria required that studies involve a population of

patients with MS, utilize the administration of DMTs, and report a measurement of

adherence. Studies reporting persistence measures (e.g., treatment

discontinuation rates) or rates of switching between DMTs (with no other measure

of adherence reported) were excluded if they did not also assess adherence. Among

the 24 studies meeting inclusion criteria, adherence to DMTs ranged from 41% to

88%. Weighted mean adherence rates were higher for intramuscular (IM) interferon

beta-1a (IFNβ-1a) administered once a week (69.4%), and subcutaneous (SC) IFNβ-1b

administered every other day (63.8%) than for SC IFNβ-1a administered 3 times a

week (58.4%) and glatiramer acetate administered daily (56.8%). There was a

numerically greater risk of MS relapse or disease progression among patients

nonadherent to therapy versus adherent patients, with findings statistically

significant in 2 of 4 studies. Additionally, 2 studies showed statistically

significant reductions in inpatient or emergency room utilization and total

MS-related medical costs among patients adherent to therapy compared with

nonadherent patients. Higher patient out-of-pocket copayments and coinsurance

were significantly associated with lower adherence to DMTs, while the use of

interventional or disease therapy management programs were associated with

improved adherence. Lack of medication adherence remains a problem among patients

with MS. Improvements in adherence have the potential to improve patient and

payer burden in terms of improved clinical outcomes and lower nonpharmacy medical

resource utilization.

DOI: 10.18553/jmcp.2013.19.s1.S24

PMID: 23383731 [Indexed for MEDLINE]

323. J Manag Care Pharm. 2013 Jan-Feb;19(1):18-25.

Excluded b/c no real-world adherence/persistence

Dalfampridine prior authorization program: a cohort study.

Gleason PP(1), Phillips J, Fenrick BA, Delgado-Riley A, Starner CI.

Author information:

(1)Prime Therapeutics LLC, Eagan, MN 55121, USA. pgleason@primetherapeutics.com

BACKGROUND: Dalfampridine (Ampyra) is indicated to improve walking in patients

with multiple sclerosis (MS) and was found to be effective in 35%-43% of

individuals with MS in clinical trials. Dalfampridine may increase seizure risk,

particularly in patients with renal impairment. A U.S. managed care expert

consensus panel agreed that patient access to dalfampridine is best managed by a

prior authorization (PA) in accordance with the FDA-approved labeling. To ensure

safe and appropriate dalfampridine use, a health plan developed and implemented a

2-phase point-of-sale PA program.

OBJECTIVES: To evaluate dalfampridine PA review decisions, utilization, and

pharmacy expenditures following the implementation of a dalfampridine safety and

clinical PA program compared with a group of dalfampridine utilizers unexposed to

a PA program.

METHODS: The study utilized retrospective administrative pharmacy claims data

from a commercial health plan averaging 1.3 million members per month. The plan

implemented a 2-phase dalfampridine safety and effectiveness PA program on August

1, 2010. A comparison group that did not implement the dalfampridine PA program

was identified from a commercially insured population with approximately 350,000

members per month. Members in both groups were required to be continuously

enrolled from August 1, 2010, through January 31, 2011. A member's earliest paid

or rejected claim found from August 1, 2010, through October 31, 2010, was

defined as the index claim. Dalfampridine-weighted 30-day supply claims were

summed and compared between groups from index date through January 31, 2011. A

pharmacy cost avoidance estimate was calculated using the difference in average

claims per member from index claim through January 31, 2011, multiplied by

dalfampridine wholesale acquisition cost. Overall, dalfampridine utilization was

evaluated between the intervention and comparison populations from August 2010

(implementation of PA in intervention group) through December 2011. Linear

regression and Poisson models were used to test the trend differences.

RESULTS: The 60 PA-exposed dalfampridine members' average follow-up was 157 days.

Phase 1 approval was obtained by 32 (53.3%) members; 4 (6.7%) members received a

denial because of renal impairment; 8 (13.3%) members received a denial due to

inability to obtain walking time; 1 (1.7%) member with relapse-remitting MS was

denied a PA due to no concomitant disease-modifying agent; and 15 (25.0%) members

did not initiate the PA process. Phase 2 approval was obtained by 23 (38.3%) of

the 60 members. The 60 PA members had a total of 126 claims and an average

utilization of 2.1 (SD 1.8) claims per member. The 20 non-PA dalfampridine

members' average follow-up was 157 days. The comparison group members had a total

of 84 claims and an average utilization of 4.2 (SD 2.0) claims per member. The PA

program resulted in an average of 2.1 (P  less than  0.001) fewer claims per

member in the PA group. The total dalfampridine cost avoidance estimate was

$143,010 or $0.03 per member per month. The overall measure of a monthly claims

utilization difference over time was statistically significantly different at P

less than  0.001, using the linear regression slope trend test. The trend line

slope was not statistically significantly different, P = 0.841, between the

intervention and comparison populations.

CONCLUSIONS: The study indicates that a dalfampridine PA program potentially

improved safety and minimized dalfampridine costs. A PA program is effective in

selecting appropriate utilizers for initial therapy. Addition of care management

may further optimize use by encouraging adherence and tracking patient response.

DOI: 10.18553/jmcp.2013.19.1.18

PMID: 23383704 [Indexed for MEDLINE]

324. J Med Econ. 2013;16(4):547-51. doi: 10.3111/13696998.2013.774281. Epub 2013 Feb

Excluded b/c no real-world adherence/persistence

21.

Implications of real-world adherence on cost-effectiveness analysis in multiple

sclerosis.

Brandes DW(1), Raimundo K, Agashivala N, Kim E.

Author information:

(1)Hope MS Center, Knoxville, TN, USA.

OBJECTIVES: Adherence to medication is essential for optimal outcomes, especially

for chronic diseases such as multiple sclerosis (MS). Studies in MS indicate that

lower adherence is associated with an increased risk of relapse, hospitalization

or emergency room (ER) visits, and higher medical costs. A previous investigation

assessed the cost per relapse avoided for patients with MS receiving first-line

disease modifying therapies (DMTs); however, the model assumed 100% adherence.

METHODS: Because real-world utilization patterns influence the actual

effectiveness of medications, this analysis assessed the impact of real-world

adherence from a US commercial payer perspective, using updated costs.

RESULTS: As was seen in the original study, in this revised model, fingolimod was

associated with the lowest cost per relapse avoided ($90,566), followed by SC IFN

β-1b (Extavia: $127,024), SC IFN β-1b (Betaseron: $137,492), SC IFN β-1a

($144,016), glatiramer acetate ($160,314), and IM IFN β-1a ($312,629). The model

inputs that had the greatest impact on the results were adherence-adjusted

relative relapse rate reduction (RRR) of fingolimod, the wholesale acquisition

costs of fingolimod, and the average number of relapses in untreated patients

with MS.

LIMITATIONS: The estimates of DMT adherence are from a single claims database

study of a large national pharmacy benefit manager that only measured adherence,

not actual relapses, and the model does not incorporate manufacturer discounts

and rebates, which are not publicly available.

CONCLUSION: These results suggest that economic analyses of MS therapies should

incorporate real-world adherence rates where available, rather than relying

exclusively on trial-based efficacy estimates when considering the economic value

of treatment alternatives, and that highly efficacious therapies with low

adherence may yield real-world efficacy that is substantially lower than that

observed in closely monitored clinical trials.

DOI: 10.3111/13696998.2013.774281

PMID: 23391123 [Indexed for MEDLINE]

325. J Med Econ. 2013;16(3):349-57. doi: 10.3111/13696998.2012.755537. Epub 2012 Dec

Excluded b/c no real-world adherence/persistence

18.

Cost-minimization analysis of fingolimod compared with natalizumab for the

treatment of relapsing-remitting multiple sclerosis in Sweden.

Bergvall N(1), Tambour M, Henriksson F, Fredrikson S.

Author information:

(1)Novartis Pharma AG, Basel, Switzerland. niklas.bergvall@novartis.com

BACKGROUND: Fingolimod and natalizumab have the same European Union licence for

the treatment of relapsing multiple sclerosis, and are considered by the

Committee for Medicinal Products for Human Use (CHMP) to have broadly similar

efficacy.

OBJECTIVE: A cost-minimization analysis was performed to compare differences in

treatment costs between fingolimod and natalizumab from a societal perspective in

Sweden.

METHODS: This analysis included costs associated with initiating and following

treatment (physician visits and monitoring), continuing therapy (drugs and

administration), and lost patient productivity and leisure time. Unit costs (in

Swedish krona [SEK]) were based on regional data (median prices for physician

visits and monitoring sessions). Natalizumab infusion costs were obtained from

the national cost-per-patient database. Drug costs for both therapies were 15,651

SEK/28 days.

RESULTS: After 3 years, fingolimod use was associated with savings of

124,823 SEK/patient compared with natalizumab (total cost/patient: 566,718 SEK vs

691,542 SEK). Cost savings with fingolimod were 40,402 SEK/patient after 1 year

and 301,730 SEK/patient after 10 years. Treatment with natalizumab was 18% more

expensive than fingolimod therapy after 1 year and 23% more expensive after 10

years.

LIMITATIONS: Based on the CHMP assessment, it was assumed that fingolimod and

natalizumab have similar efficacy. The analysis was conducted for Sweden, and

caution is needed in extrapolating the results to other countries.

CONCLUSION: Fingolimod is cost-saving compared with natalizumab for the treatment

of relapsing-remitting multiple sclerosis in Sweden.

DOI: 10.3111/13696998.2012.755537

PMID: 23211038 [Indexed for MEDLINE]

326. J Neurol. 2013 Jan;260(1):327-9. doi: 10.1007/s00415-012-6744-7. Epub 2012 Nov

Excluded b/c no real-world adherence/persistence

16.

Disease reactivation after fingolimod discontinuation in two multiple sclerosis

patients.

Ghezzi A, Rocca MA, Baroncini D, Annovazzi P, Zaffaroni M, Minonzio G, Comi G,

Filippi M.

DOI: 10.1007/s00415-012-6744-7

PMID: 23161460 [Indexed for MEDLINE]

327. PLoS One. 2013;8(3):e59694. doi: 10.1371/journal.pone.0059694. Epub 2013 Mar 19.

Excluded b/c no oral DMD results

The Australian Multiple Sclerosis (MS) immunotherapy study: a prospective,

multicentre study of drug utilisation using the MSBase platform.

Jokubaitis VG(1), Spelman T, Lechner-Scott J, Barnett M, Shaw C, Vucic S, Liew D,

Butzkueven H, Slee M; Australian Msbase Study Group.

Collaborators: Kilpatrick TJ, King J, Marriott M, van der Walt A, Skibina O,

Haartsen J, Barlow S, Dark L, Williams D, Ribbons K, Burke MT.

Author information:

(1)Melbourne Brain Centre, RMH, Department of Medicine, The University of

Melbourne, Parkville, Victoria, Australia. vilija.jokubaitis@unimelb.edu.au

OBJECTIVE: To prospectively characterise treatment persistence and predictors of

treatment discontinuation in an Australian relapsing-remitting multiple sclerosis

(RRMS) population.

METHODS: Tertiary MS treatment centres participating in the MSBase registry

prospectively assessed treatment utilisation, persistence, predictors of

treatment discontinuation and switch rates. Multivariable survival analyses were

used to compare treatment persistence between drugs and to identify predictors of

treatment discontinuation.

RESULTS: 1113 RRMS patients were studied. Patients persisted on their first

disease-modifying therapy (DMT) for a median of 2.5 years. Treatment persistence

on GA was shorter than on all IFNβ products (p<0.03). Younger age at treatment

initiation and higher EDSS were predictive of DMT discontinuation. Patients

persisted on subsequent DMTs, for 2.3 years. Patients receiving natalizumab (NAT)

as a subsequent DMT persisted longer on treatment than those on IFNβ or GA

(p<0.000). The primary reason for treatment discontinuation for any drug class

was poor tolerability. Annualised switch or cessation rates were 9.5-12.5% for

individual IFNβ products, 11.6% for GA and 4.4% for NAT.

CONCLUSION: This multicentre MS cohort study is the first to directly compare

treatment persistence on IFNβ and GA to NAT. We report that treatment persistence

in our Australian RRMS population is short, although patients receiving IFNβ as a

first DMT persisted longer on treatment than those on GA. Additionally, patients

receiving NAT as a subsequent DMT were more likely to persist on treatment than

those switched to IFNβ or GA. EDSS and age at DMT initiation were predictive of

DMT discontinuation. Treatment intolerance was the principal reason for treatment

cessation.

DOI: 10.1371/journal.pone.0059694

PMCID: PMC3602083

PMID: 23527252 [Indexed for MEDLINE]

328. Ther Clin Risk Manag. 2013;9:361-9. doi: 10.2147/TCRM.S41552. Epub 2013 Oct 3.

Excluded b/c no primary data (narrative review)

Bridging, switching or drug holidays - how to treat a patient who stops

natalizumab?

Havla J(1), Kleiter I, Kümpfel T.

Author information:

(1)Institute of Clinical, Neuroimmunology, Medical Campus, Grosshadern, Ludwig

Maximilians, University, Munich, Germany.

Natalizumab (NAT) was the first monoclonal antibody to be approved for the

treatment of relapsing-remitting multiple sclerosis (RRMS). While pivotal and

postmarketing studies have showed considerable and sustained efficacy of NAT in

RRMS, the increasing incidence of therapy-associated progressive multifocal

leukoencephalopathy (PML), a brain infection caused by the John Cunningham virus

(JCV), is a risk associated with long-term therapy. The risk for

therapy-associated PML is highest in so-called "triple risk" patients. Therefore,

long-term NAT-treated, immunosuppressive-pretreated, and JCV antibody-positive

patients often discontinue NAT therapy. However, until now, it is not known which

treatment strategy should be followed after NAT cessation. Since disease activity

returns to pretreatment levels, or even above, within 4-7 months from the last

infusion of NAT, patients who stop NAT are at considerable risk of relapse and

worsening of multiple sclerosis (MS)-related disability. Several strategies have

been applied to prevent the recurrence of disease activity after discontinuation

of NAT. Of these, bridging with intravenous methylprednisolone, and switching to

glatiramer acetate or interferon beta (IFN-beta) do not seem to be effective

enough. More promising results have been obtained in retrospective studies and

case series with fingolimod (FTY), an alternative escalation therapy for RRMS,

although some patients have showed a severe disease rebound after starting FTY

treatment. The time interval between the discontinuation of NAT and the start of

FTY might affect the recurrence of disease activity. Long-term data about the

efficacy and safety of FTY treatment after cessation of NAT are urgently needed

and should be further investigated. Prospective studies are warranted, to

optimize treatment strategies for RRMS patients who discontinue NAT, especially

because new therapies will be available in the very near future.

DOI: 10.2147/TCRM.S41552

PMCID: PMC3794889

PMID: 24124371

329. Ther Clin Risk Manag. 2013;9:73-85. doi: 10.2147/TCRM.S17426. Epub 2013 Mar 3.

Excluded b/c no primary data (narrative review)

Advances in the treatment of relapsing-remitting multiple sclerosis - critical

appraisal of fingolimod.

Gasperini C(1), Ruggieri S, Mancinelli CR, Pozzilli C.

Author information:

(1)Department of Neurosciences, S Camillo Forlanini Hospital, Rome, Italy;

Multiple sclerosis (MS) is a chronic inflammatory disorder of the central nervous

system, traditionally considered to be an autoimmune, demyelinating disease.

Based on this understanding, initial therapeutic strategies were directed at

immune modulation and inflammation control. At present, there are five licensed

first-line disease-modifying drugs for MS in Europe, and two second-line

treatments. Currently available MS therapies have shown significant efficacy

throughout many trials, but they produce different side effects. Despite

disease-modifying drugs being well known and safe, they require regular and

frequent parenteral administration and are associated with limited long-term

treatment adherence. Therefore, the development of new therapeutic strategies is

warranted. Several oral compounds are in late stages of development for treating

MS. fingolimod is an oral sphingosine-1-phosphate receptor modulator that has

demonstrated superior efficacy compared with placebo and interferon β-1a in phase

III studies. It has already been approved in the treatment of MS. This review

focuses on advances in current and novel oral treatment approaches in MS. We

summarily review the oral compounds in this study, focusing on the recent

development, approval, and the clinical experience with fingolimod.

DOI: 10.2147/TCRM.S17426

PMCID: PMC3590932

PMID: 23483794

330. J Neurol Sci. 2012 Dec 15;323(1-2):167-72. doi: 10.1016/j.jns.2012.09.009. Epub

2012 Oct 3.

Early tolerability and safety of fingolimod in clinical practice.

Ontaneda D(1), Hara-Cleaver C, Rudick RA, Cohen JA, Bermel RA.

Author information:

(1)Mellen Center, Department of Neurology, Cleveland Clinic, Cleveland, OH, USA.

ontaned@ccf.org

BACKGROUND: Fingolimod is approved by the U.S. Food and Drug Administration to

reduce relapses and disability progression in relapsing forms of MS. Several

screening studies and a first-dose observation (FDO) period are recommended due

to adverse effects observed in clinical trials.

OBJECTIVE: The objective of this study is to describe the early experience with

fingolimod, including startup, tolerability and safety in a large academic

multiple sclerosis (MS) center.

METHODS: Patients prescribed fingolimod from September 2010 to July 2011 were

identified through electronic medical records. Demographics, MS disease history,

pre-treatment screening studies, FDO experience during shared medical visits and

three month follow-up data were analyzed.

RESULTS: Three hundred ninety-one patients were prescribed fingolimod of whom 317

started the medication and were included in the analysis. Fingolimod was most

frequently used in relapsing remitting MS (n=256, 80.8%) and was prescribed as a

first-line agent in 11 cases (3.5%). FDO was uneventful in 308 patients (96.8%).

Adverse events during FDO were self limited and included symptomatic bradycardia

(n=3), chest tightness (n=2) and hypertension (n=1). Fingolimod was discontinued

in 30 patients (9.5%) at three months. Adverse effects leading to discontinuation

by more than one patient included headache (n=4), macular edema (n=3), nausea

(n=3) and hypertension (n=2).

CONCLUSIONS: Fingolimod was well tolerated during FDO and adverse events were

self limited. The shared medical visit is an appropriate setting for FDO. Adverse

effects were similar to those described in clinical trials but the

discontinuation rate was higher.

Copyright © 2012 Elsevier B.V. All rights reserved.

DOI: 10.1016/j.jns.2012.09.009

PMCID: PMC3970706

PMID: 23040960 [Indexed for MEDLINE]

331. Cochrane Database Syst Rev. 2012 Dec 12;12:CD009882. doi:

Excluded b/c no primary data (systematic review)

10.1002/14651858.CD009882.pub2.

Teriflunomide for multiple sclerosis.

He D(1), Xu Z, Dong S, Zhang H, Zhou H, Wang L, Zhang S.

Author information:

(1)Department of Neurology, Affiliated Hospital of Guiyang Medical College,

Guiyang, China.

Update in

Cochrane Database Syst Rev. 2016;3:CD009882.

BACKGROUND: Disease-modifying therapies (DMTs) for multiple sclerosis aim to

specifically reduce inflammation in relapsing multiple sclerosis and promote

neuroprotection and neurorepair in progressive multiple sclerosis (MS). Most of

the currently available disease-modifying drugs (DMDs) require regular and

frequent parenteral administration, which imposes a burden on patients and leads

to reduced adherence. Not all MS patients respond adequately to current DMDs and,

therefore, alternative MS treatments with less invasive routes of administration

and new modes of action are required to expand the current treatment repertoire,

increase adherence, and thereby improve efﬁcacy. As one of the oral DMDs,

teriflunomide is a potentially promising new oral agent in the treatment of

relapsing MS. It inhibits dihydro-orotate dehydrogenase (DHODH) and the synthesis

of pyrimidine and has selective immunosuppressive and immunomodulatory

properties.

OBJECTIVES: To explore the potential benefits of teriflunomide and so expand the

available DMT options, the effectiveness and safety of teriflunomide, as

monotherapy or combination therapy, were assessed versus placebo or approved DMDs

(IFN-β, glatiramer acetate, natalizumab, mitoxantrone, fingolimod) for modifying

disease in patients with MS.

SEARCH METHODS: The Trials Search Co-ordinator searched the Cochrane Multiple

Sclerosis and Rare Diseases of the Central Nervous System Group Specialised

Register (27 June 2012). We checked references in identified trials and manually

searched the reports (2004 to June 2012) from neurological associations and MS

societies. We also communicated with researchers participating in trials on

teriflunomide and contacted Sanofi-Aventis.

SELECTION CRITERIA: All randomised, double-blind, controlled, parallel clinical

trials (RCTs) with a length of follow-up of at least one year evaluating

teriflunomide, as monotherapy or combination therapy, versus placebo or other

treatments (IFN-β, glatiramer acetate, natalizumab, mitoxantrone, fingolimod) for

patients with MS. Titles and abstracts of the citations retrieved by the

literature search were screened independently for inclusion or exclusion by two

review authors. Any disagreement regarding inclusion was resolved by discussion

or by referral to a third assessor if necessary.

DATA COLLECTION AND ANALYSIS: Two review authors independently extracted data and

assessed trial quality. Disagreements were discussed and resolved by consensus

among review authors. Principal investigators of included studies were contacted

for additional data or confirmation of information.

MAIN RESULTS: Two studies involving 1204 people evaluated the efficacy and safety

of teriflunomide 7 mg and 14 mg, alone or with add-on IFN-β, versus placebo for

adult patients with relapsing forms of MS (relapsing-remitting (RRMS), secondary

progressive (SPMS) with relapse, and progressive relapsing MS (PRMS)) and an

entry Expanded Disability Status Scale (EDSS) score of ≤ 5.5. Both studies had

high attrition bias (26.8% and 36.4% attrition respectively). Teriflunomide 7 or

14 mg alone had potential benefits on reducing relapse rates, and alone or with

add-on IFN-β was safe for patients with relapsing forms of MS in the short term.

The most common adverse events included nasopharyngitis, headache, diarrhoea,

fatigue, elevated alanine aminotransferase levels, nausea, hair thinning or

decreased hair density, influenza, back pain, urinary tract infection, and pain

in the arms or legs. Four ongoing trials were identified.

AUTHORS' CONCLUSIONS: We found low-level evidence for the use of teriflunomide as

a disease-modifying therapy for MS, due to the limited quality of the available

RCTs. We did not conduct meta-analysis because of the clinical and methodological

diversity of the included studies. Short-term teriflunomide, 7 or 14 mg alone or

with add-on IFN-β, was safe for patients with relapsing MS. Both teriflunomide 7

and 14 mg alone had potential benefits for patients with relapsing forms of MS.

We are waiting for the publication of ongoing trials. RCTs with high

methodological quality and longer periods of observation are needed to assess

safety, disability progression, neuroprotection and quality of life.

DOI: 10.1002/14651858.CD009882.pub2

PMID: 23235682 [Indexed for MEDLINE]

332. Mult Scler. 2012 Dec;18(12):1705-17. doi: 10.1177/1352458512438239. Epub 2012 Feb

Excluded b/c no primary data (systematic review)

27.

Cutaneous adverse events associated with disease-modifying treatment in multiple

sclerosis: a systematic review.

Balak DM(1), Hengstman GJ, Çakmak A, Thio HB.

Author information:

(1)Department of Dermatology, Erasmus Medical Center, Rotterdam, The Netherlands.

Glatiramer acetate and interferon-beta are approved first-line disease-modifying

treatments (DMTs) for multiple sclerosis (MS). DMTs can be associated with

cutaneous adverse events, which may influence treatment adherence and patient

quality of life. In this systematic review, we aimed to provide an overview of

the clinical spectrum and the incidence of skin reactions associated with DMTs. A

systematic literature search was performed up to May 2011 in Medline, Embase, and

Cochrane databases without applying restrictions in study design, language, or

publishing date. Eligible for inclusion were articles describing any skin

reaction related to DMTs in MS patients. Selection of articles and data

extraction were performed by two authors independently. One hundred and six

articles were included, of which 41 (39%) were randomized controlled trials or

cohort studies reporting incidences of mainly local injection-site reactions. A

large number of patients had experienced some form of localized injection-site

reaction: up to 90% for those using subcutaneous formulations and up to 33% for

those using an intramuscular formulation. Sixty-five case-reports involving 106

MS patients described a wide spectrum of cutaneous adverse events, the most

frequently reported being lipoatrophy, cutaneous necrosis and ulcers, and various

immune-mediated inflammatory skin diseases. DMTs for MS are frequently associated

with local injection-site reactions and a wide spectrum of generalized cutaneous

adverse events, in particular, the subcutaneous formulations. Although some of

the skin reactions may be severe and persistent, most of them are mild and do not

require cessation of DMT.

DOI: 10.1177/1352458512438239

PMID: 22371220 [Indexed for MEDLINE]

333. J Manag Care Pharm. 2012 Nov-Dec;18(9):1-17.

Excluded b/c no primary data (narrative review)

Evaluating risks, costs, and benefits of new and emerging therapies to optimize

outcomes in multiple sclerosis.

Bandari DS(1), Sternaman D, Chan T, Prostko CR, Sapir T.

Author information:

(1)Hoag Neurosciences Institute, Newport Beach, CA, USA. dbandari@mscscal.com

BACKGROUND: Multiple sclerosis (MS) is a complex, chronic, and often

disablingneurological disease. Despite the recent incorporation of new

treatmentapproaches early in the disease course, care providers still face

difficultdecisions as to which therapy will lead to optimal outcomes and whento

initiate or escalate therapies. Such decisions require proper assessmentof

relative risks, costs, and benefits of new and emerging therapies, as wellas

addressing challenges with adherence to achieve optimal managementand outcomes.At

the 24th Annual Meeting Expo of the Academy of Managed CarePharmacy (AMCP), held

in San Francisco on April 18, 2012, a 4-hour activitytitled "Analyzing and

Applying the Evidence to Improve Cost-Benefit andRisk-Benefit Outcomes in

Multiple Sclerosis" was conducted in associationwith AMCP's Continuing

Professional Education Partner Program (CPEPP).The practicum, led by the primary

authors of this supplement, featureddidactic presentations, a roundtable session,

and an expert panel discussiondetailing research evidence, ideas, and discussion

topics central to MSand its applications to managed care.

OBJECTIVES: To review (a) recent advances in MS management, (b) strategiesto

optimize the use of disease-modifying therapies for MS, (c) costs ofcurrent MS

therapies, (d) strategies to promote adherence and complianceto disease-modifying

therapies, and (e) potential strategies for managedcare organizations to improve

care of their MS patient populations and optimizeclinical and economic outcomes.

SUMMARY: Advances in magnetic resonance imaging and newer therapieshave allowed

earlier diagnosis and reduction of relapses, reduction in progressionof

disability, and reduction in total cost of care in the long term.Yet, even with

the incorporation of new disease-modifying therapies intothe treatment

armamentarium of MS, challenges remain for patients, providers,caregivers, and

managed care organizations as they have to makeinformed decisions based on the

properties, risks, costs, and benefits ofeach individual drug as part of an

individualized shared decision-makingprocess. Case management and collaborative

practice models, which incorporateself-management, medication therapy, formulary

management, andcontinuous education, while promoting symptom management,

medicationadherence, and a health-promoting lifestyle, are important in the

overallmanagement of MS and can provide outcomes-based interventions aimedat

controlling costs while maximizing treatment efficacy.

DOI: 10.18553/jmcp.2012.18.S9-B.1

PMID: 23206263 [Indexed for MEDLINE]

334. Mult Scler. 2012 Nov;18(11):1636-9. doi: 10.1177/1352458512454773. Epub 2012 Jul

Excluded b/c no real-world adherence/persistence

24.

Withdrawal of fingolimod treatment for relapsing-remitting multiple sclerosis:

report of six cases.

Hakiki B(1), Portaccio E, Giannini M, Razzolini L, Pastò L, Amato MP.

Author information:

(1)Department of Neurology, University of Florence, Florence, Italy.

hakiki.bahia@unifi.it

The objective of this article is to report our experience on fingolimod

suspension in multiple sclerosis patients. We evaluated clinical and magnetic

resonance (MR) outcomes in six patients after fingolimod discontinuation. Within

three months from fingolimod suspension, five subjects returned to pre-treatment

disease activity; one patient, however, exhibited a clear rebound of clinical and

MR activity. Our findings suggest that clinical and MR outcomes after fingolimod

suspension can vary among patients. Systematic collection of clinical, laboratory

and imaging data is highly advisable to identify subjects who are at higher risk

of rebound and to define effective management strategies in these subjects.

DOI: 10.1177/1352458512454773

PMID: 22829326 [Indexed for MEDLINE]

335. Neurology. 2012 Sep 11;79(11):1130-5. doi: 10.1212/WNL.0b013e3182698c64. Epub

2012 Aug 29.

Excluded b/c no primary data (systematic review)

Disease-modifying drugs for multiple sclerosis in pregnancy: a systematic review.

Lu E(1), Wang BW, Guimond C, Synnes A, Sadovnick D, Tremlett H.

Author information:

(1)Department of Medicine, Faculty of Medicine, University of British Columbia,

Vancouver, Canada.

OBJECTIVE: To systematically review the literature regarding safety of

disease-modifying drug (DMD) use during pregnancy on perinatal and developmental

outcomes in offspring of patients with multiple sclerosis (MS).

METHODS: A PubMed and EMBASE search up to February 2012 was conducted with a

manual search of references from relevant articles. Selected studies were

evaluated using internationally accepted criteria.

RESULTS: Fifteen studies identified 761 interferon β-, 97 glatiramer acetate-,

and 35 natalizumab-exposed pregnancies. Study quality ranged from poor to good;

no study was rated excellent. Small sample sizes limited most studies. Compared

with data for unexposed pregnancies, fair- to good-quality prospective cohort

studies reported that interferon β exposure was associated with lower mean birth

weight, shorter mean birth length, and preterm birth (<37 weeks), but not low

birth weight (<2,500 g), cesarean delivery, congenital anomaly (including

malformation), or spontaneous abortion. Fewer studies of fair quality were

available for glatiramer acetate and natalizumab. Glatiramer acetate exposure was

not associated with lower mean birth weight, congenital anomaly, preterm birth,

or spontaneous abortion. Natalizumab exposure did not appear to be associated

with shorter mean birth length, lower mean birth weight, or lower mean

gestational age. No studies examined mitoxantrone or fingolimod exposure. One

study of paternal DMD use during conception found no effect on gestational age or

birth weight. Few studies examined longer-term developmental outcomes.

CONCLUSION: Further studies are needed to determine the potential risks

associated with preconceptional and in utero DMD exposure in patients with MS.

Discontinuation of DMDs before conception is still recommended.

DOI: 10.1212/WNL.0b013e3182698c64

PMCID: PMC3525300

PMID: 22933738 [Indexed for MEDLINE]

336. Mult Scler. 2012 Sep;18(9):1278-89. Epub 2012 Feb 3.

Excluded b/c no real-world adherence/persistence

Long-term follow-up of a phase 2 study of oral teriflunomide in relapsing

multiple sclerosis: safety and efficacy results up to 8.5 years.

Confavreux C(1), Li DK, Freedman MS, Truffinet P, Benzerdjeb H, Wang D, Bar-Or A,

Traboulsee AL, Reiman LE, O'Connor PW; Teriflunomide Multiple Sclerosis Trial

Group.

Author information:

(1)Hôpital Neurologique, Université Claude Bernard Lyon 1, France.

christian.confavreux@chu-lyon.fr

BACKGROUND: Teriflunomide, an oral disease-modifying therapy in development for

patients with relapsing forms of multiple sclerosis (RMS), was well tolerated and

effective in reducing magnetic resonance imaging (MRI) lesions in 179 RMS

patients in a phase 2 36-week, placebo-controlled study.

METHODS: A total of 147 patients who completed the core study entered an

open-label extension. Teriflunomide patients continued their assigned dose, and

placebo patients were re-allocated to teriflunomide, 7 mg/day or 14 mg/day. An

interim analysis was performed at a cut-off on January 8 2010.

RESULTS: The mean and median duration of study treatment, including both the core

and extension phase, from baseline to the interim cut-off, was 5.6 years

(standard deviation: 2.7 years) and 7.1 years (range: 0.05-8.5 years),

respectively. Of 147 patients, 62 (42.2%) discontinued (19% due to

treatment-emergent adverse events (TEAEs)). The most common TEAEs were mild

infections, fatigue, sensory disturbances and diarrhoea. No serious opportunistic

infections occurred, with no discontinuations due to infection. Asymptomatic

alanine aminotransferase increases (≤3× upper limit of normal (ULN)) were common

(7 mg, 64.2%; 14 mg, 62.1%); increases >3×ULN were similar across groups (7 mg,

12.3%; 14 mg, 12.1%). Mild decreases in neutrophil counts occurred; none led to

discontinuation. The incidence of malignancies was comparable to that of the

general population, and cases were not reminiscent of those observed in

immunocompromised patients. Annualised relapse rates remained low, minimal

disability progression was observed, with a dose-dependent benefit with

teriflunomide 14 mg for several MRI parameters.

CONCLUSION: Teriflunomide had a favourable safety profile for up to 8.5 years.

DOI: 10.1177/1352458512436594

PMCID: PMC3573681

PMID: 22307384 [Indexed for MEDLINE]

337. Epilepsia. 2012 Jul;53(7):1119-30. doi: 10.1111/j.1528-1167.2012.03506.x. Epub

Excluded b/c no primary data (narrative review)

2012 May 11.

Finding a better drug for epilepsy: the mTOR pathway as an antiepileptogenic

target.

Galanopoulou AS(1), Gorter JA, Cepeda C.

Author information:

(1)Saul R. Korey Department of Neurology, Dominick P. Purpura Department of

Neuroscience, Laboratory of Developmental Epilepsy, Montefiore/Einstein Epilepsy

Management Center, Albert Einstein College of Medicine, Bronx, New York, USA.

aristea.galanopoulou@einstein.yu.edu

The mammalian target of rapamycin (mTOR) signaling pathway regulates cell growth,

differentiation, proliferation, and metabolism. Loss-of-function mutations in

upstream regulators of mTOR have been highly associated with dysplasias,

epilepsy, and neurodevelopmental disorders. These include tuberous sclerosis,

which is due to mutations in TSC1 or TSC2 genes; mutations in phosphatase and

tensin homolog (PTEN) as in Cowden syndrome, polyhydramnios, megalencephaly,

symptomatic epilepsy syndrome (PMSE) due to mutations in the STE20-related kinase

adaptor alpha (STRADalpha); and neurofibromatosis type 1 attributed to

neurofibromin 1 mutations. Inhibition of the mTOR pathway with rapamycin may

prevent epilepsy and improve the underlying pathology in mouse models with

disrupted mTOR signaling, due to PTEN or TSC mutations. However the timing and

duration of its administration appear critical in defining the seizure and

pathology-related outcomes. Rapamycin application in human cortical slices from

patients with cortical dysplasias reduces the 4-aminopyridine-induced

oscillations. In the multiple-hit model of infantile spasms, pulse high-dose

rapamycin administration can reduce the cortical overactivation of the mTOR

pathway, suppresses spasms, and has disease-modifying effects by partially

improving cognitive deficits. In post-status epilepticus models of temporal lobe

epilepsy, rapamycin may ameliorate the development of epilepsy-related pathology

and reduce the expression of spontaneous seizures, but its effects depend on the

timing and duration of administration, and possibly the model used. The observed

recurrence of seizures and epilepsy-related pathology after rapamycin

discontinuation suggests the need for continuous administration to maintain the

benefit. However, the use of pulse administration protocols may be useful in

certain age-specific epilepsy syndromes, like infantile spasms, whereas

repetitive-pulse rapamycin protocols may suffice to sustain a long-term benefit

in genetic disorders of the mTOR pathway. In summary, mTOR dysregulation has been

implicated in several genetic and acquired forms of epileptogenesis. The use of

mTOR inhibitors can reverse some of these epileptogenic processes, although their

effects depend upon the timing and dose of administration as well as the model

used.

Wiley Periodicals, Inc. © 2012 International League Against Epilepsy.

DOI: 10.1111/j.1528-1167.2012.03506.x

PMCID: PMC3389589

PMID: 22578218 [Indexed for MEDLINE]

338. Mult Scler. 2012 Jul;18(7):932-46. doi: 10.1177/1352458511433302. Epub 2012 Jan

Excluded b/c no primary data (systematic review)

16.

Systematic review of disease-modifying therapies to assess unmet needs in

multiple sclerosis: tolerability and adherence.

Giovannoni G(1), Southam E, Waubant E.

Author information:

(1)Blizard Institute of Cell and Molecular Science, Barts and The London School

of Medicine and Dentistry, Queen Mary University of London, London, UK.

g.giovannoni@qmul.ac.uk

Reviews of therapeutic drugs usually focus on the highly selected and closely

monitored patient populations from randomized controlled trials. The objective of

this study was to review systematically the tolerability and adherence of

multiple sclerosis disease-modifying therapies, using data from both randomized

controlled trials and observational settings. Relevant literature was identified

using predefined search terms, and adverse event and study discontinuation data

were extracted and categorized according to study type (randomized controlled

trial or observational) and study duration. A total of 151 papers were selected

for analysis; 33% were classified as randomized controlled trials and 62% as

observational studies. Most of the papers concerned interferon preparations and

glatiramer acetate; the limited available information on mitoxantrone and

natalizumab precluded extensive examination of these. The most common adverse

events were flu-like symptoms (interferon therapies only) and injection-site

reactions. Mean discontinuation rates ranged from 16% to 27%. There were no

marked differences in tolerability or adherence data from randomized controlled

trials and observational studies, but the incidence of adverse events remained

high in lengthy studies and discontinuations accumulated with time. The present

systematic review of randomized clinical trial and observational data highlights

the tolerability and adherence issues associated with commonly used first-line

multiple sclerosis treatments.

DOI: 10.1177/1352458511433302

PMID: 22249762 [Indexed for MEDLINE]

339. Neurology. 2012 Jun 5;78(23):1877-85. doi: 10.1212/WNL.0b013e318258f7d4. Epub

Excluded b/c no real-world adherence/persistence

2012 May 23.

Teriflunomide added to interferon-β in relapsing multiple sclerosis: a randomized

phase II trial.

Freedman MS(1), Wolinsky JS, Wamil B, Confavreux C, Comi G, Kappos L, Olsson TP,

Miller A, Benzerdjeb H, Li H, Simonson C, O'Connor PW; Teriflunomide Multiple

Sclerosis Trial Group and the MRI Analysis Center.

Author information:

(1)University of Ottawa, Ottawa, Canada. mfreedman@toh.on.ca

OBJECTIVE: To evaluate teriflunomide as add-on therapy to ongoing stable-dosed

interferon-β (IFNβ) in patients with relapsing forms of multiple sclerosis (RMS).

METHODS: A total of 118 patients with RMS were randomly assigned 1:1:1 to receive

oral placebo or teriflunomide, 7 or 14 mg, once daily for 24 weeks; 86 patients

entered the 24-week extension. The primary objective was to evaluate safety;

secondary objectives were to evaluate the effects of treatment on disease

activity assessed by MRI and relapse rate.

RESULTS: Teriflunomide was well tolerated with a low and similar incidence of

treatment-emergent adverse events (TEAEs) across the 3 groups; TEAEs led to

treatment discontinuation of 4.9%, 8.1%, and 7.9% of patients in the placebo,

7-mg, and 14-mg groups, respectively. The number of gadolinium-enhancing T1

(T1-Gd) lesions was reduced in both teriflunomide groups, with relative risk

reductions (RRRs) of 84.6% (p = 0.0005) and 82.8% (p < 0.0001) for 7 and 14 mg,

respectively, compared with IFNβ alone at 48 weeks. T1-Gd lesion volume was also

reduced in the 7-mg group (RRR 72.1%, p = 0.1104) and 14-mg group (RRR 70.6%, p =

0.0154). A trend toward dose-dependent reduction in annualized relapse rate was

also noted (RRRs 32.6% [p = 0.4355] and 57.9% [p = 0.1005] for 7 and 14 mg,

respectively).

CONCLUSION: Teriflunomide as add-on therapy to IFNβ had acceptable safety and

tolerability and reduced MRI disease activity compared with IFNβ alone.

CLASSIFICATION OF EVIDENCE: This study provides Class II evidence that

teriflunomide, 7 and 14 mg, added to IFNβ, is safe. The T1-Gd lesion burden was

significantly reduced with both teriflunomide doses.

DOI: 10.1212/WNL.0b013e318258f7d4

PMID: 22622860 [Indexed for MEDLINE]

Excluded b/c no real-world adherence/persistence

340. Can J Neurol Sci. 2012 May;39(3):352-4.

MS patients report excellent compliance with oral prednisone for acute relapses.

Morrow SA(1), McEwan L, Alikhani K, Hyson C, Kremenchutzky M.

Author information:

(1)University of Western Ontario, LSHC-UH B10-118, 339 Windermere Rd, London,

Ontario, N6G 1W8, Canada. smorrow8@uwo.ca

BACKGROUND: Multiple Sclerosis is characterized by relapses separated by periods

of relative quiescence. High dose intravenous corticosteroid pulses for three to

five days is the current standard for the treatment of acute relapses, but recent

evidence supports the use of equivalent doses of oral therapy as an alternative.

The highest single dose preparation of oral prednisone is a 50mg tablet,

requiring patients to take 25 tablets a day. Questions regarding compliance with

this oral regimen have been raised.

OBJECTIVES: To determine whether MS patients are complaint with 1,250 mg of oral

prednisone daily for acute relapses.

METHODS: Between November 2008 and December 2009, all patients diagnosed with an

acute relapse in the London (Ontario) MS clinic were prospectively identified. If

treatment with oral prednisone was initiated, subjects were given a survey to be

mailed anonymously to the clinic.

RESULTS: Sixty eight MS relapses were diagnosed and treated with corticosteroids

in 66 patients of which 60 (58 subjects) were treated with 1,250 mg prednisone.

Fifty-three (91.4%) surveys were returned. The reported compliance rate was high

at 94.3% (50/53) with only one patient reporting being unable to take all the

required pills due to intolerance. Most subjects (43, 86.0%) encountered at least

one side effect, most commonly insomnia, mood changes and increased appetite. Two

thirds of subjects (69.8%) indicated a preference for oral medication for future

relapses.

CONCLUSION: High dose (1,250 mg) oral prednisone is an acceptable therapy to MS

patients for the treatment of acute relapses with a high rate of compliance.

PMID: 22547517 [Indexed for MEDLINE]

Excluded b/c no primary data (narrative review)

341. Orphanet J Rare Dis. 2012 Apr 12;7:20. doi: 10.1186/1750-1172-7-20.

Behçet's disease.

Saadoun D(1), Wechsler B.

Author information:

(1)Service de Médecine Interne 2, Centre de Référence Maladies Autoimmunes rares,

CHU Pitié-Salpêtrière, 83 Bd de l'Hôpital, 75013, Paris, France.

david.saadoun@psl.aphp.fr

DEFINITION OF THE DISEASE: Behçet disease (BD) is a chronic, relapsing,

multisystemic disorder characterized by mucocutaneous, ocular, vascular and

central nervous system manifestations.EPIDEMIOLOGY: BD seems to cluster along the

ancient Silk Road, which extends from eastern Asia to the Mediterranean basin.

European cases are often described, not exclusively in the migrant population.

CLINICAL DESCRIPTION: The clinical spectrum includes oral and genital

ulcerations, uveitis, vascular, neurological, articular, renal and

gastrointestinal manifestations.

ETIOLOGY: The etiopathogenesis of the disease remains unknown, although genetic

predisposition, environmental factors and immunological abnormalities have been

implicated.

DIAGNOSTIC METHODS: Diagnosis is only based on clinical criteria. DIFFERRENTIAL

DIAGNOSIS: It depends on the clinical presentation of BD, but sarcoidosis,

multiple sclerosis, Crohn's disease, Takayasu's arteritis, polychondritis or

antiphospholipid syndrome need to be considered.

MANAGEMENT: Treatment is symptomatic using steroids and immunomodulatory therapy.

It is efficient depending on the rapidity of initiation, the compliance, and the

duration of therapy.

PROGNOSIS: The prognosis is severe due to the ocular, neurological and arterial

involvement.

DOI: 10.1186/1750-1172-7-20

PMCID: PMC3472229

PMID: 22497990 [Indexed for MEDLINE]

Excluded b/c no real-world adherence/persistence

342. Int J MS Care. 2012 Spring;14(1):46-53. doi: 10.7224/1537-2073-14.1.46.

Injectable multiple sclerosis medications: a patient survey of factors associated

with injection-site reactions.

Stewart TM(1), Tran ZV(1).

Author information:

(1)Rocky Mountain MS Center, Westminster, CO, USA (TMS), and the Department of

Biostatistics & Informatics, Colorado School of Public Health, University of

Colorado Anschutz Medical Campus, Aurora, CO, USA (ZVT).

Although injection-site reactions (ISRs) occur with US Food and Drug

Administration-approved injectable disease-modifying therapies (DMTs) for

multiple sclerosis, there are currently few reports of real-world data on ISR

management strategies or possible correlations between ISRs and patient

demographics, disease characteristics, and missed injections. Patient-reported

data on the use of DMTs, patient demographic and disease characteristics, missed

injections, and ISR reduction strategies were collected via e-mail, a patient

registry (www.ms-cam.org), and a Web-based survey. Of the 1380 respondents, 1201

(87%) indicated that they had used injectable DMTs, of whom 377 (31%) had used

intramuscular (IM) interferon beta-1a (IFNβ-1a), 172 (14%) had used subcutaneous

(SC) IFNβ-1a, 183 (15%) had used SC IFNβ-1b, and 469 (39%) had used glatiramer

acetate (GA). The majority of respondents were older (73% were ≥40 years), female

(79%), married or living with a partner (72%), white (94%), and nonsmoking (82%).

Injection-site reaction incidence, grouped according to severity, varied among

DMTs, with IM IFNβ-1a causing significantly (P < .001) fewer mild, moderate, or

severe ISRs than the other therapies. Female sex and younger age were

significantly (P < .05) associated with more moderate ISRs among users of IM

IFNβ-1a, SC IFNβ-1b, and GA. Nonwhites reported severe ISRs more often than

whites. For all DMTs injection-site massage and avoidance of sensitive sites were

the most frequently used strategies to minimize ISRs. These data may help

identify patients with characteristics associated with a higher risk for ISRs,

allowing health-care professionals to provide anticipatory guidance to patients

at risk for decreased adherence or discontinuation.

DOI: 10.7224/1537-2073-14.1.46

PMCID: PMC3882978

PMID: 24453732

Excluded b/c no oral DMD results

343. Int J MS Care. 2012 Spring;14(1):17-24. doi: 10.7224/1537-2073-14.1.17.

Adherence to first-line disease-modifying therapy for multiple sclerosis in

kuwait.

Alroughani R(1), Thussu A(1).

Author information:

(1)Department of Medicine, Division of Neurology, Amiri Hospital, Kuwait City,

Kuwait.

The aim of this retrospective study was to determine the rate of nonadherence to

disease-modifying therapies (DMTs) among multiple sclerosis (MS) patients in

Kuwait and to identify reasons for patient discontinuation of long-term therapy.

Using a newly established MS registry at our institution, we collected data on MS

patients' demographics, clinical characteristics, disability measures, and

continuation or discontinuation of first-line DMTs. Reasons for nonadherence were

divided into four categories: adverse events, inconvenience, perceived lack of

efficacy, and physician-documented disease progression. Of 212 eligible patients,

40.1% were found to be nonadherent to first-line DMTs. In the nonadherent group,

the female-to-male ratio was 1.75:1 and the mean age at disease onset was 26.8

years. Of this group, 69.4% of patients had a relapsing-remitting course, 18.8%

had a secondary progressive course, and 11.8% had clinically isolated syndrome.

Compared with the adherent group, the nonadherent group had a shorter mean

disease duration (P = .014) and a greater likelihood of having Expanded

Disability Status Scale (EDSS) scores of 3 or lower (67.1% vs. 48.0%; P = .007).

Inconvenience was the most common reason for nonadherence (32.9%), followed by

perceived lack of efficacy (25.9%), adverse events (23.5%), and

physician-documented disease progression (17.7%). In summary, the rate of

nonadherence to first-line DMTs in MS patients at our institution is considered

high. Most nonadherent patients had a short disease duration and low EDSS scores.

Inconvenience and perceived lack of efficacy were the most common reasons for

nonadherence. The results demonstrate a need to improve treatment adherence among

MS patients in Kuwait through providing better patient education, improving

communication between patients and health-care providers, defining therapy

expectations, and instituting new therapeutic techniques.

DOI: 10.7224/1537-2073-14.1.17

PMCID: PMC3882977

PMID: 24453728

344. Arch Neurol. 2012 Feb;69(2):262-4. doi: 10.1001/archneurol.2011.1057.

Excluded b/c no real-world adherence/persistence

Rebound of disease activity after withdrawal of fingolimod (FTY720) treatment.

Havla JB(1), Pellkofer HL, Meinl I, Gerdes LA, Hohlfeld R, Kümpfel T.

Author information:

(1)Institute of Clinical Neuroimmunology, Ludwig-Maximilians-University,

Marchioninistr 15, 81377 Munich, Germany.

BACKGROUND: The oral sphingosine-1-phosphate receptor modulator fingolimod

(FTY720) was recently approved for the treatment of relapsing-remitting multiple

sclerosis. To date, data about a possible recurrence of disease activity after

discontinuation of fingolimod treatment are scarce.

OBJECTIVE: To describe a patient who discontinued fingolimod treatment after a

local malignant melanoma was diagnosed. Three months after cessation, he had a

striking rebound of multiple sclerosis activity.

DESIGN: Case report and review of literature.

SETTING: Institute of Clinical Neuroimmunology, Ludwig-Maximilians-University,

Munich, Germany.

PATIENT: A 45-year-old man diagnosed as having relapsing-remitting multiple

sclerosis.

MAIN OUTCOME MEASURES: Multiple sclerosis disease activity including annual

relapse rate, Expanded Disability Status Scale score, and number of

gadolinium-enhancing lesions on magnetic resonance imaging before, during, and

after treatment with fingolimod.

RESULTS: Three months after discontinuation of treatment with fingolimod, the

patient experienced a severe relapse, with Expanded Disability Status Scale score

progression from 2.5 to 4.5. On brain and spinal magnetic resonance imaging, he

showed a rebound of disease activity, with a drastic increase of

gadolinium-enhancing lesions (>20).

CONCLUSIONS: Two aspects relevant to any newly approved multiple sclerosis

treatment with immunomodulatory properties are highlighted with this case: first,

possible rebound of disease activity after discontinuation; second, the

occurrence of a tumor as a possible treatment-related complication.

DOI: 10.1001/archneurol.2011.1057

PMID: 22332194 [Indexed for MEDLINE]

345. J Neurol. 2012 Feb;259(2):386-8. doi: 10.1007/s00415-011-6367-4. Epub 2012 Jan

Excluded b/c no real-world adherence/persistence

10.

Does fingolimod in multiple sclerosis patients cause macular edema?

Turaka K(1), Bryan JS.

Author information:

(1)Associated Retina Consultants Ltd, 7600 N 15th Street, Phoenix, AZ 85020, USA.

keen_268@yahoo.com

Multiple sclerosis (MS) is a demyelinating disease of the central nervous system

and is the common cause of optic neuritis. Fingolimod, an immunosuppressive

agent, is used in MS to prevent acute exacerbations. We report a case of

relapsing-remitting MS treated with fingolimod. The patient presented with an

acute decrease in vision in the left eye. Eye examination showed clinical macular

edema (ME) in the left eye, which was confirmed on fluorescein angiogram and

optical coherence tomography (OCT). After discontinuation of fingolimod and

treatment with topical corticosteroid medication, there was complete resolution

of the ME. The ME as a side-effect of fingolimod is reversible after

discontinuing, which was seen on OCT.

DOI: 10.1007/s00415-011-6367-4

PMID: 22231867 [Indexed for MEDLINE]

Excluded b/c no primary data (narrative review)

346. Curr Pharm Des. 2012;18(29):4489-97.

Immune therapy of multiple sclerosis--future strategies.

Meuth SG(1), Göbel K, Wiendl H.

Author information:

(1)University of Münster, Department of Neurology - Inflammatory disorders of the

nervous system and neurooncology, Domagkstraße 13, 48149 Münster, Germany.

sven.meuth@ukmuenster

Baseline disease-modifying therapies (DMTs) for multiple sclerosis (MS) include

three different preparations of interferon-beta (IFN-β) and glatiramer acetate

(GA). These substances reduce relapse rates, side-effects are tolerated by most

patients and - after more than 15 years of experience - the long-term safety

profile for these drugs can be appraised as very good. In 2006, the therapeutic

tool kit was augmented by the first monoclonal antibody, natalizumab, approved as

monotherapy for treatment-refractory highly active MS. The restriction to these

patient groups results from the rare, but fatal risk of JC virus-induced

progressive multifocal leukoencephalopathy (PML). The first oral agent

(fingolimod) was approved in 2010 for the United States and in 2011 for Europe.

As a further option for therapy escalation the chemotherapeutic agent

mitoxantrone is approved for non-responding relapsing-remitting MS (RRMS) or

secondary progressive MS (SPMS). The use of mitoxantrone is limited by severe

cardiotoxicity and the risk of treatment related acute leukemia. However, despite

the fact that therapeutic options for MS have significantly been widened over the

past decade new treatment options and more convenient modes of application are

needed to enhance efficacy and improve adherence to therapy. This article will

review recent developments in MS treatments focusing on oral agents (cladribine,

fingolimod, BG00012, teriflunomide and laquinimod) and novel monoclonal

antibodies (alemtumzumab, daclizumab, ocrelizumab, ofatumumab).

PMID: 22612746 [Indexed for MEDLINE]

347. Drug Des Devel Ther. 2012;6:175-86. doi: 10.2147/DDDT.S8927. Epub 2012 Jul 19.

Excluded b/c no primary data (narrative review)

Development of oral agent in the treatment of multiple sclerosis: how the first

available oral therapy, fingolimod will change therapeutic paradigm approach.

Gasperini C(1), Ruggieri S.

Author information:

(1)Department of Neurosciences, S Camillo Forlanini Hospital, University of Rome

Sapienza, Rome, Italy. c.gasperini@libero.it

Multiple sclerosis (MS) is a chronic inflammatory disorder of the central nervous

system, traditionally considered to be an autoimmune, demyelinating disease.

Based on this understanding, the initial therapeutic strategies were directed at

immune modulation and inflammation control. At present, there are five licensed

first-line disease-modifying drugs and two second-line treatments in MS.

Currently available MS therapies have shown significant efficacy throughout many

trials, but they produce different side-effect profiles in patients. Since they

are well known and safe, they require regular and frequent parenteral

administration and are associated with limited long-term treatment adherence.

Thus, there is an important need for the development of new therapeutic

strategies. Several oral compounds are in late-stage development for treating MS.

Fingolimod (FTY720; Novartis, Basel, Switzerland) is an oral

sphingosine-1-phosphase receptor modulator which has demonstrated superior

efficacy compared with placebo and interferon β-1a in Phase III studies and has

been approved in the treatment of MS. We summarily review the oral compounds in

study, focusing on the recent development, approval and the clinical experience

with FTY720.

DOI: 10.2147/DDDT.S8927

PMCID: PMC3414371

PMID: 22888218 [Indexed for MEDLINE]

348. ISRN Neurol. 2012;2012:451457. doi: 10.5402/2012/451457. Epub 2012 Dec 22.

Excluded b/c no oral DMD results

Switching and escalating therapy in long-lasting multiple sclerosis: not always

necessary.

Carvalho AT(1), Sá MJ.

Author information:

(1)MS Clinic, Department of Neurology, Centro Hospitalar de São João, 4200-319

Porto, Portugal ; Department of Neurology, Centro Hospitalar de Vila Nova

Gaia/Espinho, 4434-502 Vila Nova de Gaia, Portugal.

Although therapy switch is common among patients with multiple sclerosis (MS),

sometimes the initial prescribed treatment is maintained for a long period with

clinical stability, low disability, and nonsignificant side effects. We aim to

describe demographic and clinical characteristics of patients treated in our MS

clinic with the same disease-modifying drug (DMD) lasting for >12 years. From the

cohort of 51 patients followed in our MS clinic with relapse-remitting MS who

started an DMD between 1996 and 1999, we found a high percentage (51%) of

patients who were efficiently treated with the first DMD. These patients were

mainly females, with low annualized relapse rate and Multiple Sclerosis Severity

Score (MSSS). Our results may be related to the open and multidisciplinary model

of our MS clinic organization. Identifying characteristics associated with

therapy persistence may be useful in developing strategies to improve therapy

effectiveness.

DOI: 10.5402/2012/451457

PMCID: PMC3539327

PMID: 23316389

349. J Med Econ. 2012;15(6):1149-58. doi: 10.3111/13696998.2012.707631. Epub 2012 Jul

Excluded b/c no real-world adherence/persistence

27.

Fingolimod reduces direct medical costs compared to natalizumab in patients with

relapsing-remitting multiple sclerosis in The Netherlands.

Heisen M(1), Treur MJ, van der Hel WS, Frequin ST, Groot MT, Verheggen BG.

Author information:

(1)Pharmerit Europe, Rotterdam, The Netherlands. mheisen@pharmerit.com

OBJECTIVE: To assess the costs of oral treatment with Gilenya® (fingolimod)

compared to intravenous infusion of Tysabri® (natalizumab) in patients with

relapsing-remitting multiple sclerosis (RRMS) in The Netherlands.

METHODS: A cost-minimization analysis was used to compare both treatments. The

following cost categories were distinguished: drug acquisition costs,

administration costs, and monitoring costs. Costs were discounted at 4%, and

incremental model results were presented over a 1, 2, 5, and 10 year time

horizon. The robustness of the results was determined by means of a number of

deterministic univariate sensitivity analyses. Additionally, a break-even

analysis was carried out to determine at which natalizumab infusion costs a

cost-neutral outcome would be obtained.

RESULTS: Comparing fingolimod to natalizumab, the model predicted discounted

incremental costs of -€2966 (95% CI: -€4209; -€1801), -€6240 (95% CI: -€8800;

-€3879), -€15,328 (95% CI: -€21,539; -€9692), and -€28,287 (95% CI: -€39,661;

-€17,955) over a 1, 2, 5, and 10-year time horizon, respectively. These

predictions were most sensitive to changes in the costs of natalizumab infusion.

Changing these costs of €255 within a range from €165-364 per infusion resulted

in cost savings varying from €4031 to €8923 after 2 years. The additional

break-even analysis showed that infusion costs-including aseptic preparation of

the natalizumab solution-needed to be as low as the respective costs of €94 and

€80 to obtain a cost neutral result after 2 and 10 years.

LIMITATIONS: Neither treatment discontinuation and subsequent re-initiation nor

patient compliance were taken into account. As a consequence of the applied

cost-minimization technique, only direct medical costs were included.

CONCLUSION: The present analysis showed that treatment with fingolimod resulted

in considerable cost savings compared to natalizumab: starting at €2966 in the

first year, increasing to a total of €28,287 after 10 years per RRMS patient in

the Netherlands.

DOI: 10.3111/13696998.2012.707631

PMID: 22737996 [Indexed for MEDLINE]

350. J Med Econ. 2012;15(3):601-9. doi: 10.3111/13696998.2012.667027. Epub 2012 Mar 1.

Excluded b/c no oral DMD results

Impact of medication adherence to disease-modifying drugs on severe relapse, and

direct and indirect costs among employees with multiple sclerosis in the US.

Ivanova JI(1), Bergman RE, Birnbaum HG, Phillips AL, Stewart M, Meletiche DM.

Author information:

(1)Analysis Group, Inc, New York, NY, USA. jivanova@analysisgroup.com

OBJECTIVE: To compare rates of severe relapse and total direct and indirect costs

over a 2-year period between US-based employees with multiple sclerosis (MS) who

were adherent and non-adherent to disease-modifying drugs (DMDs).

METHODS: Employees with ≥1 MS diagnosis (ICD-9-CM: 340.x) and ≥1 DMD pharmacy

claim between 1/1/2002-12/31/2007 were identified from a large US administrative

claims database. Patients had continuous coverage ≥6 months before (baseline) and

≥24 months after (study period) their index date (first DMD claim). Adherence was

measured using medication possession ratio (MPR) over the study period. Patients

with MPR ≥80% were considered adherent (n = 448) and those with MPR <80% as

non-adherent (n = 200). Multivariate analyses were used to compare rates of

severe relapse (inpatient or Emergency Department visit with MS diagnosis) and

costs in 2007 dollars between DMD adherent and non-adherent patients. Direct

costs were calculated as reimbursements to providers for medical services and

prescription drugs excluding DMDs. Indirect costs included disability and

medically-related absenteeism costs.

RESULTS: DMD adherent patients were on average older (43.5 vs 41.8 years,

p = 0.015) and more likely to be male (38.6% vs 26.0%, p = 0.002) compared with

non-adherent patients. Adherent patients had lower rates of depression, higher

rates of previous DMD use, and higher baseline MS-related costs. After adjusting

for differences in baseline characteristics, DMD adherent patients had a lower

rate of severe relapse (12.4% vs 19.9%, p = 0.013) and lower total (direct and

indirect) costs ($14,095 vs $16,638, p = 0.048) over the 2-year study period.

CONCLUSIONS: In this study, DMD adherence was associated with a significantly

lower rate of severe relapse and lower total costs over 2 years. Causality cannot

be inferred because adherence and outcomes were measured over the same period.

The study was subject to limitations associated with use of claims data and the

absence of clinical measures.

DOI: 10.3111/13696998.2012.667027

PMID: 22376190 [Indexed for MEDLINE]

351. Patient Prefer Adherence. 2012;6:55-61. doi: 10.2147/PPA.S26250. Epub 2012 Jan

Excluded b/c no oral DMDs

18.

Patient assessment of an electronic device for subcutaneous self-injection of

interferon β-1a for multiple sclerosis: an observational study in the UK and

Ireland.

D'Arcy C(1), Thomas D, Stoneman D, Parkes L.

Author information:

(1)West London Neuroscience Centre, Charing Cross Hospital, London, UK.

BACKGROUND: Injectable disease-modifying drugs (DMDs) reduce the number of

relapses and delay disability progression in patients with relapsing-remitting

multiple sclerosis (RRMS). Regular self-injection can be stressful and impeded by

MS symptoms. Auto-injection devices can simplify self-injection, overcome

injection-related issues, and increase treatment satisfaction. This study

investigated patient responses to an electronic auto-injection device.

METHODS: Patients with RRMS (n = 63), aged 18-65 years, naïve to subcutaneous

(sc) interferon (IFN) β-1a therapy, were recruited to a Phase IV, observational,

open-label, multicenter study (NCT01195870). Patients self-injected sc IFN β-1a

using the RebiSmart™ (Merck Serono S.A. - Geneva, Switzerland) electronic

auto-injector for 12 weeks, including an initial titration period if recommended

by the prescribing physician. In week 12, patients completed a questionnaire

comprising of a visual analog scale (VAS) to rate how much they liked using the

device, a four-point response question on ease of use ('very difficult',

'difficult', 'easy', or 'very easy'), and a list of ten device functions to rank,

based upon their experiences.

RESULTS: Six patients (9.5%) discontinued the study: one switched to manual

injection; two discontinued all treatment; three changed therapy. In total, 59

out of 63 patients (93.7%) completed the VAS; 54 out of 59 (91.5%; 95% confidence

interval: 81.3%-97.2%) 'liked' using the electronic auto-injector (score ≥6),

whereas 57 out of 59 (96.6%) rated the device overall as 'easy' or 'very easy' to

use. Device features rated as most useful were the hidden needle (mean [standard

deviation] score: 3.3 [3.01]; n = 56), confirmation sound (3.9 [2.45]), and

multidose cartridge (4.6 [2.32]). The least useful functions were the dose

history list (8.0 [2.57]) and dose history calendar (7.5 [2.30]).

CONCLUSIONS: These findings suggest that the electronic auto-injector may be

suitable for patients who are new to injectable DMD therapy. Devices that

simplify the injection process may help to ensure that patients receive the full

benefits of treatment.

DOI: 10.2147/PPA.S26250

PMCID: PMC3269318

PMID: 22298944

352. Expert Opin Emerg Drugs. 2011 Dec;16(4):697-712. doi:

Excluded b/c no primary data (narrative review)

10.1517/14728214.2011.642861. Epub 2011 Dec 7.

Emerging oral drugs for relapsing-remitting multiple sclerosis.

Gasperini C(1), Ruggieri S.

Author information:

(1)Dipartimento Testa Collo, Azienda Ospedaliera S. Camillo-Forlanini, Rome,

Italy. c.gasperini@libero.it

INTRODUCTION: Multiple sclerosis (MS) is a chronic inflammatory disease of the

central nervous system (CNS), traditionally considered to be an autoimmune,

demyelinating disease. The last two decades have witnessed the introduction of

several therapies for MS. At present, there are five licensed first-line,

disease-modifying drugs (DMDs) in MS and two second-line treatments.

Nevertheless, in clinical practice DMDs or immunosuppressive treatments are

frequently associated with suboptimal response in terms of efficacy and several

side effects leading to poor patient adherence.

AREAS COVERED: Since MS is a chronic disease, DMDs require long-term, regular

injection or monthly parenteral infusions, which may be uncomfortable and

inconvenient for the patient. Thus, there is an important need for new

therapeutic strategies, especially those that may offer greater patient

satisfaction in order to optimize therapeutic outcomes. Currently, five oral

therapies are in Phase III development or have recently been approved for the

treatment of relapsing-remitting MS: cladribine and fingolimod, the first

approved in Russia and Australia, the latter is more widespread. Fumaric acid

(BG-12), teriflunomide (A77126 or HMR1726) and laquinimod (ABR-215062) are in

Phase III trials. Details of these five drugs will be covered in this review.

EXPERT OPINION: Preliminary results indicate that oral medications are as

effective as, or possibly more effective than, current injectable formulations.

It is believable that improved outcomes will translate into higher real and

perceived efficacy rates and contribute to improved adherence. The decision to

switch established patients from injectable to oral medications will be made on

balancing the efficacy and tolerability of the patient's existing therapy and

their compliance history, even though safety is likely to become the most

important factor in the future development of MS drugs.

DOI: 10.1517/14728214.2011.642861

PMID: 22148963 [Indexed for MEDLINE]

353. Imaging Sci Dent. 2011 Dec;41(4):177-81. doi: 10.5624/isd.2011.41.4.177. Epub

Excluded b/c no real-world adherence/persistence

2011 Dec 19.

Pyknodysostosis: report of a rare case with review of literature.

Ramaiah KK(1), George GB, Padiyath S, Sethuraman R, Cherian B.

Author information:

(1)Department of Oral Medicine and Radiology, Mar Baselios Dental College,

Kothamangalam, India.

Pyknodysostosis is a rare autosomal recessive disorder characterized by the post

natal onset of short limbs, short stature, and generalized hyperostosis along

with acro-osteolysis with sclerosis of the terminal phalanges, a feature that is

considered essentially pathognomonic. Other features include persistence of

fontanelles, delayed closure of sutures, wormian bones, absence of frontal

sinuses, and obtuse mandibular gonial angle with relative mandibular prognathism.

We report a case of 17-year-old girl who presented with a chief complaint of

retention of deciduous teeth. General physical examination demonstrated short

stature, frontal and parietal bossing, depressed nasal bridge, beaked nose,

hypoplastic midface, wrinkled skin over the finger tips, and nail abnormalities.

Radiographs showed multiple impacted permanent and supernumerary teeth,

hypoplastic paranasal sinuses with acro-osteolysis of terminal phalanges, and

open fontanelles, and sutures along with wormian bones in the lambdoidal region.

DOI: 10.5624/isd.2011.41.4.177

PMCID: PMC3251792

PMID: 22232728

354. BMC Neurol. 2011 Nov 10;11:144. doi: 10.1186/1471-2377-11-144.

Excluded b/c no oral DMD results

The prevalence of injection-site reactions with disease-modifying therapies and

their effect on adherence in patients with multiple sclerosis: an observational

study.

Beer K(1), Müller M, Hew-Winzeler AM, Bont A, Maire P, You X, Foulds P, Mårlind

J, Curtius D.

Author information:

(1)Private practice, Obere Bahnhofstrasse, CH 9500, Wil, Switzerland.

praxis.beer@bluewin.ch

BACKGROUND: Interferon beta (IFNβ) and glatiramer acetate (GA) are administered

by subcutaneous (SC) or intramuscular (IM) injection. Patients with multiple

sclerosis (MS) often report injection-site reactions (ISRs) as a reason for

noncompliance or switching therapies. The aim of this study was to compare the

proportion of patients on different formulations of IFNβ or GA who experienced

ISRs and who switched or discontinued therapy because of ISRs.

METHODS: The Swiss MS Skin Project was an observational multicenter study.

Patients with MS or clinically isolated syndrome who were on the same therapy for

at least 2 years were enrolled. A skin examination was conducted at the first

study visit and 1 year later.

RESULTS: The 412 patients enrolled were on 1 of 4 disease-modifying therapies for

at least 2 years: IM IFNβ-1a (n = 82), SC IFNβ-1b (n = 123), SC IFNβ-1a (n =

184), or SC GA (n = 23). At first evaluation, ISRs were reported by fewer

patients on IM IFNβ-1a (13.4%) than on SC IFNβ-1b (57.7%; P < 0.0001), SC IFNβ-1a

(67.9%; P < 0.0001), or SC GA (30.4%; P = not significant [NS]). No patient on IM

IFNβ-1a missed a dose in the previous 4 weeks because of ISRs, compared with 5.7%

of patients on SC IFNβ-1b (P = 0.044), 7.1% of patients on SC IFNβ-1a (P =

0.011), and 4.3% of patients on SC GA (P = NS). Primary reasons for discontinuing

or switching therapy were ISRs or lack of efficacy. Similar patterns were

observed at 1 year.

CONCLUSIONS: Patients on IM IFNβ-1a had fewer ISRs and were less likely to switch

therapies than patients on other therapies. This study may have implications in

selecting initial therapy or, for patients considering switching or discontinuing

therapy because of ISRs, selecting an alternative option.

DOI: 10.1186/1471-2377-11-144

PMCID: PMC3227610

PMID: 22074056 [Indexed for MEDLINE]

355. Ther Deliv. 2011 Nov;2(11):1455-65.

Excluded b/c no primary data (narrative review)

Review of subcutaneous interferon β-1a, delivered via the electronic

self-injection device RebiSmart™, for the treatment of multiple sclerosis.

Devonshire VA(1), Verdun di Cantogno E.

Author information:

(1)Department of Neurology, University of British Columbia, Rm S159, 2211

Wesbrook Mall, Vancouver V6T 2B5, BC, Canada. vdev@shaw.ca

Multiple sclerosis is a chronic disease requiring lifelong treatment with

disease-modifying drugs that aim to prevent relapses and slow the progression of

disability. The established first-line treatments for multiple sclerosis all

require regular injections, and real-world observations suggest that long-term

adherence to treatment is low. Indeed, poor adherence to disease-modifying drug

treatment has been associated with poorer clinical outcomes. Autoinjectors have

been developed to improve the success of self-injection, whilst also making

injections more comfortable for patients, with the aim of minimizing obstacles to

treatment adherence. RebiSmart™ is a new electronic autoinjector for subcutaneous

administration of interferon β-1a (Rebif®) that includes several unique features

designed to further reduce barriers to adherence, including a dosing log and

adjustable comfort settings.

PMID: 22844681 [Indexed for MEDLINE]

356. BMC Neurol. 2011 Oct 6;11:122. doi: 10.1186/1471-2377-11-122.

Excluded b/c no oral DMD results

Disease-modifying drug initiation patterns in commercially insured multiple

sclerosis patients: a retrospective cohort study.

Margolis JM(1), Fowler R, Johnson BH, Kassed CA, Kahler K.

Author information:

(1)Thomson Reuters, 332 Bryn Mawr Ave, Bala Cynwyd, PA 19004, USA.

jay.margolis@thomsonreuters.com

BACKGROUND: The goal of this research was to compare the demographics, clinical

characteristics and treatment patterns for newly diagnosed multiple sclerosis

(MS) patients in a commercial managed care population who received

disease-modifying drug (DMD) therapy versus those not receiving DMD therapy.

METHODS: A retrospective cohort study using US administrative healthcare claims

identified individuals newly diagnosed with MS (no prior MS diagnosis 12 months

prior using ICD-9-CM 340) and ≥18 years old during 2001-2007 to characterize them

based on demographics, clinical characteristics, and pharmacologic therapy for

one year prior to and a minimum of one year post-index. The index date was the

first MS diagnosis occurring in the study period. Follow-up of subjects was done

by ICD-9-CM code identification and not by actual chart review. Multivariate

analyses were conducted to adjust for confounding variables.

RESULTS: Patients were followed for an average of 35.7±17.5 months after their

index diagnosis. Forty-three percent (n=4,462) of incident patients received

treatment with at least one of the DMDs during the post-index period. Treated

patients were primarily in the younger age categories of 18-44 years of age, with

DMD therapy initiated an average of 5.3±9.1 months after the index diagnosis.

Once treatment was initiated, 27.7% discontinued DMD therapy after an average of

17.6±14.6 months, and 16.5% had treatment gaps in excess of 60 days.

CONCLUSIONS: Nearly 60% of newly-diagnosed MS patients in this commercial managed

care population remained untreated while over a quarter of treated patients

stopped therapy and one-sixth experienced treatment gaps despite the risk of

disease progression or a return of pre-treatment disease activity.

DOI: 10.1186/1471-2377-11-122

PMCID: PMC3204236

PMID: 21974973 [Indexed for MEDLINE]

Excluded b/c no primary data (narrative review)

357. Int J MS Care. 2011 Fall;13(3):105-12. doi: 10.7224/1537-2073-13.3.105.

The evolving role of the multiple sclerosis nurse: an international perspective.

Burke T(1), Dishon S(1), McEwan L(1), Smrtka J(1).

Author information:

(1)Institute for Immunology & Allergy Research, Westmead Millennium Institute,

Westmead, Sydney, Australia (TB); Carmel Medical Centre, Haifa, Israel (SD);

London Health Sciences Centre, Multiple Sclerosis Clinic, London, Ontario, Canada

(LM); and Fort Lauderdale MS Center, Pompano Beach, FL, USA (JS).

A greater understanding of the pathogenesis of multiple sclerosis (MS) and the

need for treatments with increased efficacy, safety, and tolerability have led to

the ongoing development of new treatments. The evolution of treatments for MS is

expected to have a dramatic impact on the entire health-care team, especially MS

nurses, who build strong collaborative partnerships with their patients. MS

nurses help patients better understand their disease and treatment options,

facilitate the initiation and management of treatment, and encourage adherence.

With new oral therapies entering the market, the potential for increased

efficacy, tolerability, adherence, and convenience for patients is evident.

However, the resulting change in the treatment paradigm means that the skill set

required of an MS nurse will inevitably expand. There will be a growing need for

professional training and development to ensure that nurses are familiar with the

wider range of treatments and their specific modes of action, dosing schedules,

and benefit/risk profiles. In addition, the MS nurse's role will expand to

include management of the complex monitoring needs specific to each therapy. This

article explores how the role of the MS nurse is evolving with the development of

new MS therapies, including novel oral therapies.

DOI: 10.7224/1537-2073-13.3.105

PMCID: PMC3882972

PMID: 24453713

Excluded b/c no oral DMD results

358. J Behav Med. 2011 Oct;34(5):330-8. doi: 10.1007/s10865-011-9316-x. Epub 2011 Jan

23.

Exacerbation history is associated with medication and appointment adherence in

MS.

Hancock LM(1), Bruce JM, Lynch SG.

Author information:

(1)Department of Psychology, University of Missouri-Kansas City, Kansas City, MO

64110, USA.

Disease-modifying treatments are designed to prevent exacerbations in multiple

sclerosis (MS). To date, few studies have examined the relationship between

disease activity and treatment adherence in MS. The primary aim of this study was

to examine the association between disease activity (e.g., annualized relapse

rates), medication adherence, and appointment adherence in relapsing-remitting

MS. Retrospective exacerbation and appointment data were collected and used to

predict prospective medication adherence. Results indicated that patients with

higher annualized relapse rates missed fewer doses of medication and were less

likely to miss appointments. Conversely, patients with relatively stable disease

were more likely to demonstrate poor medication adherence and poor appointment

adherence. Patients who missed more appointments also missed more doses of their

disease modifying medication. Future studies may wish to examine clinical methods

designed to improve immunotherapy adherence among patients who are in relatively

symptom-free stages of relapsing-remitting MS.

DOI: 10.1007/s10865-011-9316-x

PMID: 21259038 [Indexed for MEDLINE]

359. Adv Ther. 2011 Sep;28(9):761-75. doi: 10.1007/s12325-011-0054-9. Epub 2011 Aug

Excluded b/c no oral DMD results

24.

Adherence and persistence among multiple sclerosis patients after one

immunomodulatory therapy failure: retrospective claims analysis.

Halpern R(1), Agarwal S, Borton L, Oneacre K, Lopez-Bresnahan MV.

Author information:

(1)Health Economics and Outcomes Research, OptumInsight, Eden Prairie, MN 55344,

USA.

INTRODUCTION: There are no published data on patient adherence to, and

persistence with, disease-modifying therapies (DMT) for multiple sclerosis (MS)

after one immunomodulatory failure. The present study compares secondline DMT

adherence and persistence among patients with MS.

METHODS: Patients with MS initiating a second-line treatment with natalizumab,

intramuscular interferon beta-1a (i.m.-IFNβ-1a), subcutaneous (s.c.) IFNβ-1a,

interferon beta-1b (IFNβ-1b), and glatiramer acetate (GA) from January 1, 2006 to

October 4, 2008 were identified from a retrospective claims database associated

with a large US health plan. Adherence was measured with medication possession

ratio (MPR); adherence indicated MPR ≥ 0.80. Persistence was measured as time

until a minimum 60-day gap in second-line therapy. Adherence and persistence were

modeled with logistic and Cox proportional hazard regressions, respectively.

RESULTS: The study population comprised 1381 patients. Multivariate analysis

showed that the odds of adherence were significantly higher in the natalizumab

cohort compared with all other second-line cohorts. The natalizumab cohort was

more likely to be persistent compared with the i.m.-IFNβ-1a and IFNβ-1b cohorts.

CONCLUSION: The natalizumab cohort was more adherent compared with the other

second-line DMT cohorts, likely due in large part to active physician involvement

and monitoring. Adherence to DMT, even after first-line failure, is critical to

achieving optimal therapeutic benefit.

DOI: 10.1007/s12325-011-0054-9

PMID: 21870169 [Indexed for MEDLINE]

360. Expert Rev Med Devices. 2011 Sep;8(5):543-53. doi: 10.1586/erd.11.29. Epub 2011

Excluded b/c no primary data (narrative review)

Jul 6.

A new electronic device for subcutaneous injection of IFN-β-1a.

Exell S(1), Verdun E, Driebergen R.

Author information:

(1)Merck Serono S.A.-Geneva*, 9 chemin des Mines, 1202 Geneva, Switzerland.

simon.exell@merckserono.net

Disease-modifying drugs (DMDs) can provide important benefits for patients with

multiple sclerosis (MS), but nonadherence to treatment is associated with an

increased risk of relapse. All first-line DMDs used in MS require regular

injection, but injection-related problems are common barriers to treatment

adherence. Autoinjectors that allow automatic injection at the press of a button

have increased the ease and convenience of injection, compared with manual

injection. A new electronic autoinjector has recently been introduced for the

administration of subcutaneous IFN-β-1a. This device is the first electronic

autoinjector for use with any MS therapy, and includes several innovative

features that may be advantageous to patients. One of these features is an

accurate electronic dosing log, which can be viewed by the patient and the

healthcare provider. This article discusses this new electronic device in the

context of other autoinjectors currently used to self-inject first-line DMDs in

MS.

DOI: 10.1586/erd.11.29

PMID: 21728909 [Indexed for MEDLINE]

361. J Neurol. 2011 Sep;258(9):1665-9. doi: 10.1007/s00415-011-5996-y. Epub 2011 Mar

Excluded b/c no real-world adherence/persistence

23.

De-escalation from natalizumab in multiple sclerosis: recurrence of disease

activity despite switching to glatiramer acetate.

Havla J(1), Gerdes LA, Meinl I, Krumbholz M, Faber H, Weber F, Pellkofer HL,

Hohlfeld R, Kümpfel T.

Author information:

(1)Institute of Clinical Neuroimmunology, Medical Campus Grosshadern,

Ludwig-Maximilians-University, Marchioninistr. 15, 81377 Munich, Germany.

Natalizumab (NAT) is an effective therapy for relapsing-remitting multiple

sclerosis (MS), but is associated with an increased risk of progressive

multifocal leucoencephalopathy after 2 years therapy. Thus, NAT treated patients

often decide to stop NAT therapy after 2 years. Reports on recurrence of disease

activity after NAT discontinuation are controversial. We studied disease activity

in 13 MS patients who stopped NAT therapy and either remained without disease

modifying therapy (no DMT, n = 6), or switched to glatiramer acetate (GLAT, n =

7). Annual relapse rate (ARR), expanded disability status scale (EDSS), and

number of patients with contrast-enhancing-lesions (Gd+) on MRI before, during

and within 1 year after NAT were determined. We observed recurrence of disease

activity in both groups (5/7 GLAT treated patients and 6/6 patients without DMT)

within 12 months after cessation of NAT (mean time to first relapse was 5.5

months for all patients). One of the GLAT treated patients and three patients

without DMT had severe relapses with sustained EDSS worsening. No differences in

ARR, EDSS and MRI parameters were seen between both groups. Patients with

relapses after NAT therapy, however, tended to show higher disease activity

(EDSS, ARR) before initiation of NAT therapy compared to patients without

relapses. Duration of NAT treatment was not associated with higher disease

activity after NAT discontinuation. In this observation the majority of patients

showed reappearance of disease activity after discontinuation of NAT regardless

of whether they switched to GLAT or remained without DMT. Further treatment

strategies are warranted for patients who discontinue NAT therapy.

DOI: 10.1007/s00415-011-5996-y

PMID: 21431380 [Indexed for MEDLINE]

Excluded b/c no oral DMD results

362. Can J Neurol Sci. 2011 May;38(3):429-33.

Adherence to multiple sclerosis disease-modifying therapies in Ontario is low.

Wong J(1), Gomes T, Mamdani M, Manno M, O'Connor PW.

Author information:

(1)Division of Neurology, The Leslie Dan Faculty of Pharmacy, University of

Toronto, Ontario,Canada.

BACKGROUND/OBJECTIVE: Differences in patient adherence to various

disease-modifying drugs (DMDs) in the treatment of multiple sclerosis (MS) are

not well understood. The goal of this study was to evaluate adherence of adult MS

patients in Ontario with public drug plan coverage to various DMDs: intramuscular

interferon beta-1a (i.m. IFNβ-1a, Avonex), subcutaneous interferon beta-1a (s.c.

IFNβ-1a, Rebif), subcutaneous interferon beta-1b (IFNβ-1b, Betaseron) or

glatiramer acetate (Copaxone).

METHODS: In this retrospective cohort study, Ontario Public Drug Plan

beneficiaries aged 15 or older who were newly treated with i.m. IFNβ-1a, s.c.

IFNβ-1a, IFNβ-1b or glatiramer acetate between April 2006 and March 2008 were

followed forward until treatment discontinuation, switch to another DMD or a

maximum two year follow-up period. Cumulative persistence rates were analyzed by

the Kaplan-Meier method. The proportion of patients reaching the study endpoints

after the two year follow-up period was also calculated.

RESULTS: Cumulative persistence rates for all four DMDs were similar over time

(p=0.80), ranging from 73.6-79.1% at six months, 59.1-63.1% at one year and

41.5-47.4% at two years. After two years, the proportion of patients who had

discontinued treatment, switched to another DMD or died was similar among DMDs

(p=0.79, Fisher's exact test). Switching between DMD types was low and occurred

in 3.4-6.5% of new DMD users.

CONCLUSIONS: Adherence to DMDs in adult MS patients in Ontario is poor, which is

consistent with previously reported adherence rates to MS DMDs in other regions.

No significant differences in adherence exist between the DMDs evaluated in this

study.

PMID: 21515501 [Indexed for MEDLINE]

363. Clin Ther. 2011 May;33(5):598-607. doi: 10.1016/j.clinthera.2011.04.021.

Excluded b/c no oral DMD results

Retrospective evaluation of the impact of copayment increases for specialty

medications on adherence and persistence in an integrated health maintenance

organization system.

Kim YA(1), Rascati KL, Prasla K, Godley P, Goel N, Dunlop D.

Author information:

(1)College of Pharmacy, The University of Texas at Austin, Austin, Texas

78712-0127, USA.

BACKGROUND: Specialty drugs are generally defined as high-cost injectable,

infused, oral, or inhaled drugs that require close monitoring. Specialty drugs

account for an increasing percentage of total drug expenditures, and management

of specialty drugs has become a priority. A Central Texas-based integrated health

maintenance organization system implemented a specialty drug benefit to manage

expensive specialty drug costs.

OBJECTIVES: Our objective was to measure and compare the change in adherence and

persistence after implementation of copayment increases for select specialty

medications used on a long-term basis (at least 2 years).

METHODS: Patients who were long-term users of anti-inflammatory,

immunosuppressant, cancer, and multiple sclerosis medications were selected. The

intervention group consisted of those whose out-of-pocket payment for specialty

medications increased, and the control group consisted of those whose

out-of-pocket costs did not change. Adherence, defined by proportion of days

covered, was measured every 3 months for 12 months before and after the change.

Individual growth model analysis evaluated the changes in adherence. Cox

regression analysis determined the difference in persistence between groups.

RESULTS: There were 178 and 202 patients in the intervention and control groups,

respectively. The growth model showed a small but statistically significant

decrease in proportion of days covered of 0.040 after copay changes in the

intervention versus control group (P < 0.001) for immunosuppressants. The Cox

regression analysis indicated a higher probability of intervention patients on

anti-inflammatory drugs (hazard ratio [HR] = 2.53; 95% CI, 1.38-4.62) and

immunosuppressants (HR = 3.01; 95% CI, 1.20-7.56) would be nonpersistent compared

with those in their control groups.

CONCLUSIONS: The move to the specialty formulary allows for closer scrutiny of

specialty utilization by pharmacists, who actively monitor utilization and

access. Despite the minimal adherence decrease and significant persistence

changes with certain drug types, the results indicated relatively more stability

with specialty drug use than reported with traditional pharmaceuticals.

Copyright © 2011 Elsevier HS Journals, Inc. All rights reserved.

DOI: 10.1016/j.clinthera.2011.04.021

PMID: 21665044 [Indexed for MEDLINE]

364. Int J Neurosci. 2011 Apr;121(4):171-5. doi: 10.3109/00207454.2010.541572.

Excluded b/c no primary data (narrative review)

Will new injection devices for interferon β-1a s.c. affect treatment adherence in

patients with multiple sclerosis? An expert opinion in the Middle East.

Deleu D(1), Alsharoqi I, Al Jumah MA, Al Tahan AR, Bohlega S, Dahdaleh M, Inshasi

J, Khalifa A, Szólics M, Yamout BI.

Author information:

(1)Hamad Medical Corporation, Department of Neurology Medicine, Hamad Medical

Corporation, Doha, Qatar. doc_deleu@hotmail.com

Treatment adherence to disease modifying drugs (DMDs) in multiple sclerosis is

sub-optimal. This, in turn, can affect patients' long-term responses to therapy.

A key factor that influences treatment adherence is the need for self-injection

of DMDs, which can be demanding and disruptive for patients, and difficult for

those with cognitive difficulties or reduced manual dexterity. In addition, pain

resulting from poor injection technique, and needle anxiety, may both compromise

adherence. Changes to the formulation of interferon (IFN) beta-1a for

subcutaneous injection that were designed to improve injection local

tolerability, and changes in drug-delivery technology, designed to make

injections simpler and more convenient for patients, were reviewed by a group of

experts on MS in the Middle East. The group also considered the possible effects

of these changes in drug delivery technology on patient adherence to IFN beta-1a

s.c.

DOI: 10.3109/00207454.2010.541572

PMID: 21329472 [Indexed for MEDLINE]

365. BMC Neurol. 2011 Mar 30;11:40. doi: 10.1186/1471-2377-11-40.

Excluded b/c no primary data (study design)

Drug adherence and multidisciplinary care in patients with multiple sclerosis:

protocol of a prospective, web-based, patient-centred, nation-wide, Dutch cohort

study in glatiramer acetate treated patients (CAIR study).

Jongen PJ(1), Hengstman G, Hupperts R, Schrijver H, Gilhuis J, Vliegen JH,

Hoogervorst E, van Huizen M, van Munster E, Samijn J, de Schryver E, Siepman T,

Tonk M, Zandbergen E, ten Holter J, van der Kruijk R, Borm G.

Author information:

(1)MS4 Research Institute, Nijmegen, the Netherlands. ms4ri@kpnmail.nl

BACKGROUND: Multiple sclerosis (MS) is a chronic inflammatory demyelinating

disease of the central nervous system, for which no definitive treatment is

available. Most patients start with a relapsing-remitting course (RRMS).

Disease-modifying drugs (DMDs) reduce relapses and disability progression. First

line DMDs include glatiramer acetate (GA), interferon-beta (INFb)-1a and INFb-1b,

which are all administered via injections. Effectiveness of DMD treatment depends

on adequate adherence, meaning year-long continuation of injections with a

minimum of missed doses. In real-life practice DMD-treated patients miss 30% of

doses. The 6-month discontinuation rate is up to 27% and most patients who

discontinue do so in the first 12 months.Treatment adherence is influenced by the

socio-economic situation, health care and caregivers, disease, treatment and

patient characteristics. Only a few studies have dealt with adherence-related

factors in DMD-treated patients. Self-efficacy expectations were found to be

related to GA adherence. Patient education and optimal support improve adherence

in general. Knowledge of the aspects of care that significantly relate to

adherence could lead to adherence-improving measures. Moreover, identification of

patients at risk of inadequate adherence could lead to more efficient care.In the

near future new drugs will become available for RRMS. Detailed knowledge on

factors prognostic of adherence and on care aspects that are associated with

adequate adherence will improve the chances of these drugs becoming effective

treatments. We investigate in RRMS patients the relationship between drug

adherence and multidisciplinary care, as well as factors associated with

adherence. Given the differences in the frequency of administration and in the

side effects between the DMDs we decided to study patients treated with the same

DMD, GA.

METHODS/DESIGN: The Correlative analyses of Adherence In Relapsing remitting MS

(CAIR) study is an investigator-initiated, prospective, web-based,

patient-centred, nation-wide cohort study in the Netherlands.The primary

objective is to investigate whether GA adherence is associated with specific

disciplines of care or quantities of specific care. The secondary objective is to

investigate whether GA adherence is associated with specific aspects of the

socio-economic situation, health care and caregivers, disease, treatment or

patient characteristics.All data are acquired on-line via a study website. All

RRMS patients in the Netherlands starting GA treatment are eligible. Patients are

informed by neurologists, nurses, and websites from national MS patient

organisations. All data, except on disability, are obtained by patient

self-reports on pre-defined and random time points. The number of missed doses

and the number of patients having discontinued GA treatment at 6 and 12 months

are measures of adherence. Per care discipline the number of sessions and the

total duration of care are measures of received care. The full spectrum of

non-experimental care that is available in the Netherlands is assessed. Care

includes 'physical' contacts, contacts by telephone or internet, health-promoting

activities and community care activities. Care received over the preceding 14

days is assessed by patients at baseline and every other week thereafter up to

month 12. Every 3 months neurologists and nurses record care disciplines to which

patients have been referred.The Dutch Adherence Questionnaire-90 (DAQ-90) is a

90-item questionnaire based on the World Health Organisation (WHO) 2003 report on

adherence and comprehensively assesses five domains of evidence-based

determinants of adherence: socio-economic, health care and caregivers, disease,

treatment, and patient-related factors. In addition, self-efficacy is assessed by

the MS Self-Efficacy Scale (MSSES), and mood and health-related quality of life

(HRQoL) by the Multiple Sclerosis Quality of Life-54 questionnaire (MSQoL-54).

Relapses and adverse events probably or definitively related to GA are also

reported.

DISCUSSION: In this study data is mainly acquired by patients' self-reporting via

the internet. On-line data acquisition by patients does not require study visits

to the hospital and can easily be integrated into daily life. The web-based

nature of the study is believed to prevent missing data and study drop-outs.

Moreover, the automated process of filling in questionnaires ensures completeness

and consistency, thus improving data quality. The combination of patient-reported

outcomes, fully web-based data capture and nation-wide information to all

eligible patients are distinguishing features of the study and contribute to its

scientific potential.

TRIAL REGISTRATION: Netherlands Trial Register (NTR): NTR2432.

DOI: 10.1186/1471-2377-11-40

PMCID: PMC3080802

PMID: 21450086 [Indexed for MEDLINE]

366. J Neurol. 2011 Mar;258(3):402-11. doi: 10.1007/s00415-010-5766-2. Epub 2010 Oct

Excluded b/c no oral DMD results

6.

A multicenter study of the predictors of adherence to self-injected glatiramer

acetate for treatment of relapsing-remitting multiple sclerosis.

Zwibel H(1), Pardo G, Smith S, Denney D, Oleen-Burkey M.

Author information:

(1)Neuroscience Consultants, Comprehensive Multiple Sclerosis Center, Coral

Gables, FL, USA.

Treatment with disease-modifying immunomodulators is recommended for patients

with relapsing-remitting MS (RRMS). However, continuous adherence to treatment

with these injected therapies can be challenging. The main objective was to

examine the predictors of adherence to glatiramer acetate using a study model

derived from Prochaska's transtheoretical model of change. We conducted a

12-week, prospective, observational study. Potential predictors included

readiness stage, MS self-efficacy, decisional balance (pros and cons of

self-injection), and injection competence. Adults with RRMS, either

treatment-naïve (TN) or treatment-experienced (TE), taking glatiramer acetate for

the first time were studied. Interventions (including injection training) were

implemented to promote adherence. The evaluable population included 146 TN

patients and 88 TE patients who had previously discontinued beta-interferons.

Adherence rates did not differ between TN and TE groups (86% for both at week

12); however, predictors of adherence did. For TN patients, greater functional

self-efficacy, higher self-injection competence at baseline, and improvement in

self-injection competence over the first month of therapy predicted adherence.

For TE patients, lower body mass index and longer duration of MS predicted

adherence. Interventions to improve self-efficacy and self-injection competence

should be a priority when treating TN patients. Behavioral predictors of

adherence in TE patients warrant further study.

DOI: 10.1007/s00415-010-5766-2

PMID: 20922405 [Indexed for MEDLINE]

367. Patient Prefer Adherence. 2011 Feb 27;5:101-8. doi: 10.2147/PPA.S10506.

Excluded b/c no primary data (narrative review)

Patient considerations in the management of multiple sclerosis: development and

clinical utility of oral agents.

Girouard N(1), Soucy N.

Author information:

(1)MS Clinic, Ottawa Hospital, Ottawa, Ontario, Canada.

Multiple sclerosis (MS) is one of the most frequently occurring disabling

neurological disorders among young adults in Canada. It is a chronic inflammatory

disorder of the central nervous system (CNS) that is thought to be immune

mediated in nature. An estimated 55,000-75,000 Canadians suffer from this

debilitating disease. Starting in the mid-1990s, we witnessed the beginning of a

new era in the treatment of MS. Treatments finally became available to help

modify the course of the disease. Early initiation of treatment soon after

diagnosis has become the expectation in many MS clinics, warranting many

decisions to be made by the patient with the assistance of their health care

team. Currently, there are two categories of disease-modifying therapies (DMTs)

available: immunomodulatory and immunosuppressant agents. Although

disease-modifying therapies are not a cure and are only moderately effective,

they offer a possible slowing of any progression that may occur over time, a

decrease in relapse activity, and a decrease in the amount of new lesions

developing in the CNS found on magnetic resonance imaging. Not only have these

agents been partly effective but up to now they have only been available

parenterally, which has many limitations, including a major factor in determining

the best outcome for the treatment: adherence. Four new DMTs will likely become

available to Canadians over the next five years. Fingolimod, cladribine,

teriflunomide, and laquinimod are likely to be marketed as the first oral DMTs in

Canada. The US Food and Drug Administration approved fingolimod in September 2010

as a first-line therapy for relapsing forms of MS. Dalfampridine is also

available in the US as an agent able to improve walking. Even if these agents

present with higher efficacy and a promising safety and tolerability profile,

thus possibly demonstrating better adherence, it will be imperative for the

health care professionals to focus on monitoring and supporting the patient to

ensure reliable reporting of side effects and to improve overall adherence. In

the near future, more treatments will become available to the MS population, and

choices will become even more complex so that ongoing support, open

communication, and education are required to tame any uncertainties about

decisions made regarding treatments.

DOI: 10.2147/PPA.S10506

PMCID: PMC3063656

PMID: 21448467

368. Neurology. 2011 Feb 22;76(8 Suppl 3):S28-37. doi: 10.1212/WNL.0b013e31820db40f.

Excluded b/c no primary data (narrative review)

Future clinical challenges in multiple sclerosis: Relevance to sphingosine

1-phosphate receptor modulator therapy.

Hohlfeld R(1), Barkhof F, Polman C.

Author information:

(1)Institute for Clinical Neuroimmunology, Munich, Germany.

reinhard.hohlfeld@med.uni-muenchen.de

The limitations of established therapies for multiple sclerosis (MS) are

well-known and include the need for injections, treatment adherence and

convenience issues, partial efficacy, and, in some cases, a risk of potentially

life-threatening adverse events, such as progressive multifocal

leukoencephalopathy. Recently, attention has focused on developing more effective

therapies that are administered orally and target neurodegeneration as well as

inflammation. In this review, we provide an outlook on the future clinical

challenges for MS treatment and management, and focus specifically on the

emerging sphingosine 1-phosphate receptor (S1PR) modulators. We highlight the

importance of improving our understanding of the neurobiological basis of MS to

develop well-tolerated targeted therapies and the need to include advanced MRI

assessments that quantify neurodegeneration in interventional studies in MS. As

more treatments become available, often with complex pharmacodynamic actions,

objective assessment of benefit-to-risk profiles becomes increasingly important

to ensure that patients receive appropriate care. Pharmacovigilance and immune

monitoring will become important aspects of patient treatment and management in

the future. With respect to S1PR modulation, we review the experimental agents

that are in clinical development for MS and summarize the steps taken in

postmarketing surveillance to ensure that fingolimod (FTY720) has a

well-characterized safety profile.

DOI: 10.1212/WNL.0b013e31820db40f

PMID: 21339488 [Indexed for MEDLINE]

369. J Neuroimmunol. 2011 Feb;231(1-2):15-22. doi: 10.1016/j.jneuroim.2010.09.016.

Excluded b/c no primary data (narrative review)

Epub 2010 Oct 13.

Emerging disease-modifying oral therapies for multiple sclerosis.

Losy J(1), Kalinowska-Łyszczarz A.

Author information:

(1)Department of Clinical Neuroimmunology, Poznan University of Medical Sciences,

ul. Przybyszewskiego 49, 60-355 Poznań, Poland. jlosy@ump.edu.pl

Although therapy for multiple sclerosis (MS) has changed substantially over the

past few decades, introducing immunomodulatory drugs into everyday clinical

practice, it is still not satisfactory enough in halting the disease progression

and increasing disability. Moreover, its injection-based administration leads to

suboptimal adherence, even further reducing the potential treatment benefits.

Emerging disease-modifying oral agents for MS are therefore warranted. In this

paper advances in the novel oral therapeutic approaches to MS treatment are

reviewed.

Copyright © 2010 Elsevier B.V. All rights reserved.

DOI: 10.1016/j.jneuroim.2010.09.016

PMID: 20947176 [Indexed for MEDLINE]

370. J Neurosci Nurs. 2011 Feb;43(1):3-14; quiz 15-6.

Excluded b/c no primary data (narrative review)

Emerging oral therapies for multiple sclerosis.

Miller CE(1), Umhauer MA.

Author information:

(1)William C. Baird Multiple Sclerosis Research Center, Jacobs Neurological

Institute, University at Buffalo, The State University of New York, Buffalo, NY,

USA. cmiller@TheJNI.org

Despite notable advances in the understanding of multiple sclerosis (MS) and the

availability of several treatment options, there is a need for therapies that are

more effective, safe, convenient, and well tolerated. Several new oral MS

therapies are being investigated. Because data on these new therapies continue to

emerge, nurses will play a pivotal role in educating patients regarding the

benefits and risks of potential treatments and in monitoring patients for

response, safety, tolerability, and adherence. This article reviews the oral MS

therapies recently approved as well as those currently in development or

submitted for Food and Drug Administration approval.

PMID: 21338040 [Indexed for MEDLINE]

Excluded b/c no oral DMD results

371. J Med Internet Res. 2011 Jan 24;13(1):e12. doi: 10.2196/jmir.1687.

Use of an online community to develop patient-reported outcome instruments: the

Multiple Sclerosis Treatment Adherence Questionnaire (MS-TAQ).

Wicks P(1), Massagli M, Kulkarni A, Dastani H.

Author information:

(1)PatientsLikeMe, Research & Development, Cambridge, MA 02141, USA.

pwicks@patientslikeme.com

BACKGROUND: Patients with multiple sclerosis (MS) may face barriers, such as

treatment fatigue, memory problems, or side effects, that may influence their

adherence to medication.

OBJECTIVE: The objective of our study was to use an online community to develop a

self-report questionnaire to quantify adherence and barriers to achieving

adherence, that is specific to MS disease-modifying treatments (DMTs) and

predictive of missed doses.

METHODS: A review of the scientific literature and analysis of discussions

between MS patients on PatientsLikeMe.com were used to generate survey items

salient to patients. Cognitive debriefing was used to refine the items. The

Multiple Sclerosis Treatment Adherence Questionnaire (MS-TAQ) contains 30

questions in three subscales: Barriers, Side Effects, and Coping Strategies.

RESULTS: MS patients completed an online survey (response rate: 431 of 1209

invited, 35.7%). Between 16% (14/86) and 51% (51/100) of MS patients missed at

least 1 dose of their DMT in the previous 28 days, with significant

between-treatment differences. The MS-TAQ Barriers scale was positively

correlated with the proportion of doses missed (r = .5), demonstrating a stronger

relationship between adherence and perceived barriers than was found with

clinical or demographic variables (r ≈ .3). The Coping Strategies subscale was

negatively correlated with missed doses (r = -.3), suggesting that use of more

coping strategies is associated with higher adherence.

CONCLUSIONS: Online communities can provide domains of interest and psychometric

data to more rapidly develop and prototype patient-reported outcome instruments.

The MS-TAQ offers patients and clinicians a simple method for identifying

barriers to adherence, which may then be targeted through interventions.

DOI: 10.2196/jmir.1687

PMCID: PMC3221333

PMID: 21266318 [Indexed for MEDLINE]

372. Patient Prefer Adherence. 2011 Jan 20;5:73-84. doi: 10.2147/PPA.S15702.

Excluded b/c no oral DMD results

Comparison of adherence and persistence among multiple sclerosis patients treated

with disease-modifying therapies: a retrospective administrative claims analysis.

Halpern R(1), Agarwal S, Dembek C, Borton L, Lopez-Bresnahan M.

Author information:

(1)Health Economics and Outcomes Research, i3 Innovus, Eden Prairie, MN, USA;

PURPOSE: To compare adherence and persistence among patients with multiple

sclerosis (MS) initiated on disease-modifying therapy (DMTs), including

intramuscular (IM) interferon beta-1a (IFNβ-1a), subcutaneous (SC) IFNβ-1a,

IFNβ-1b, or glatiramer acetate (GA).

METHODS: MS patients initiated on IM-IFNβ-1a, SC-IFNβ-1a, IFNβ-1b, or GA between

January 1, 2000 and January 2, 2008 were identified from a retrospective claims

database study associated with a large US health plan. The date of DMT initiation

was the index date; patients were observed for 6 months before and 12-36 months

after the index date. Adherence to the index DMT was measured with a medication

possession ratio (MPR), the proportion of days patients possessed their index

DMTs; MPR ≥ 0.80 was considered adherent. Persistence was time in days from index

date until the earlier of a minimum 60-day gap in DMT therapy or the last DMT

claim during follow-up. Adherence and persistence were modeled with logistic and

Cox proportional hazard regressions, respectively.

RESULTS: The study population comprised 6,680 patients in the DMT cohorts:

IM-IFNβ-1a (N = 2,305, 34.5%); IFNβ-1b (N = 894, 13.4%); GA (N = 2,270, 34.0%);

and SC-IFNβ-1a (N = 1,211, 18.1%). The IM-IFNβ-1a cohort had significantly higher

regression-adjusted odds of adherence relative to the other cohorts: 52.4% higher

odds versus the IFNβ-1b cohort (OR = 0.656, CI = 0.561-0.768); 33.5% higher odds

versus the GA cohort (OR = 0.749, CI = 0.665-0.844); and 20.6% higher odds versus

the SC-IFNβ-1a cohort (OR = 0.829, CI = 0.719-0.957). There were no consistent

differences in persistence between the cohorts.

CONCLUSION: IM-IFNβ-1a patients had significantly higher odds of adherence

compared with other DMT cohorts, possibly attributable to IM-IFNβ-1a's less

frequent dosing schedule. The benefits of adherence may include better quality of

life, lower risk of relapse, and fewer hospitalizations and emergency visits,

making adherence a critical component of MS management.

DOI: 10.2147/PPA.S15702

PMCID: PMC3058604

PMID: 21423591

373. Adv Ther. 2011 Jan;28(1):51-61. doi: 10.1007/s12325-010-0093-7. Epub 2010 Dec 6.

Excluded b/c no oral DMD results

Impact of adherence to disease-modifying therapies on clinical and economic

outcomes among patients with multiple sclerosis.

Tan H(1), Cai Q, Agarwal S, Stephenson JJ, Kamat S.

Author information:

(1)HealthCore, Inc., 800 Delaware Ave., Wilmington, DE 19801, USA.

jtan@healthcore.com

BACKGROUND: Adherence to disease-modifying therapies (DMTs) is essential for the

reduction of multiple sclerosis (MS) progression and relapse. However, only

limited data currently exist on the impact of treatment adherence on MS-related

clinical and economic outcomes in the real world setting.

OBJECTIVE: To assess the impact of treatment adherence on MS-related

hospitalizations (inpatient [INP]), ER visits, MS relapses, and medical costs.

DESIGN/METHODS: Patients with ≥ 1 ICD-9-CM code for MS who received ≥ 1 DMT

between July 1, 2004 and June 30, 2008 were identified using the administrative

claims database. The first DMT received during the study period was defined as

the index treatment and ≥ 6-month preindex and ≥ 12-month postindex continuous

health-plan enrollment were required for inclusion. Adherence was assessed using

the medication possession ratio (MPR); patients with MPR ≥ 80% were regarded as

adherent. Multivariate analyses were used to evaluate the impact of adherence on

MS-related outcomes after controlling for baseline demographic and clinical

characteristics.

RESULTS: In this cohort (n=2446), 59.6% of the patients were adherent to their

DMT. Compared with the nonadherent group, adherent patients were significantly

less likely to have MS-related INP (odds ratio [OR]: 0.63, 95% confidence

interval [CI], 0.47-0.83) and MS relapses (OR: 0.71, 95% CI, 0.59-0.85). No

significant difference was found in ER risk between adherent and nonadherent

groups (8.4% vs. 10.5%, P=0.068, OR: 0.80, 95% CI: 0.60-1.07). On average, the

adherent group incurred lower medical costs than the nonadherent group ($3380,

95% CI, $3046-$3750 vs. $4348, 95% CI, $3828-$4940, P=0.003).

CONCLUSION: Treatment adherence is associated with better clinical and economic

outcomes including lower risks for MS-related hospitalization, MS relapse, and

less MS-related medical costs. Treatments that require infrequent administrations

and have favorable adherence profiles may benefit patients who are unable to

adhere to DMT therapies. Such treatments may be important in improving disease

outcomes and may be suitable therapeutic candidates for the management of MS.

DOI: 10.1007/s12325-010-0093-7

PMID: 21153000 [Indexed for MEDLINE]

374. Biol Pharm Bull. 2011;34(6):933-6.

Relapse of experimental autoimmune encephalomyelitis after discontinuation of

Excluded b/c no real-world adherence/persistence

FTY720 (Fingolimod) treatment, but not after combination of FTY720 and pathogenic

autoantigen.

Yoshida Y(1), Tsuji T, Fujita T, Kohno T.

Author information:

(1)Department of Pathological Biochemistry, Faculty of Pharmaceutical Sciences,

Setsunan University, Hirakata, Osaka 573-0101, Japan.

FTY720 (Fingolimod) is known to have a significant therapeutic effect on

experimental autoimmune encephalomyelitis (EAE). Here, we used an EAE mouse

model, which had been established by immunizing C57BL/6J mice with a partial

peptide of myelin oligodendrocyte glycoprotein (MOG₃₅₋₅₅), to examine the relapse

of EAE upon discontinuation of treatment with FTY720 alone or in combination with

MOG₃₅₋₅₅. Relapse was confirmed to occur in all animals (n=6) within one week

after discontinuation of FTY720, with increase in the number of lymphocytes

infiltrating the spinal cord and demyelination. However, in the case of

combination therapy with FTY720 and MOG₃₅₋₅₅, relapse following discontinuation

of treatment was completely suppressed. The autoantigenic peptide might serve to

suppress the clonal selection of relapse-associated autoantigen-specific T cells.

PMID: 21628899 [Indexed for MEDLINE]

375. CNS Drugs. 2011 Jan;25(1):37-52. doi: 10.2165/11539820-000000000-00000.

Excluded b/c no primary data (narrative review)

Oral therapies for multiple sclerosis: a review of agents in phase III

development or recently approved.

Gold R(1).

Author information:

(1)Department of Neurology, St Josef-Hospital, Ruhr University, Bochum, Germany.

ralf.gold@ruhr-uni-bochum.de

Several disease-modifying therapies are approved for the management of multiple

sclerosis (MS). While reasonably effective, these therapies require long-term

parenteral self-injection, which is inconvenient for some patients and can be

associated with injection-related adverse effects. Consequently, there is a need

in MS for an oral therapy option. Currently, five oral therapies are in phase III

development or have recently been approved for the treatment of

relapsing-remitting MS: cladribine (approved in Russia and Australia), fingolimod

(approved in the US and Russia), BG-12 (phase III), laquinimod (phase III) and

teriflunomide (phase III). While the availability of oral therapies has been much

anticipated by physicians and patients, neurologists will need to be cautious in

selecting such therapy, which may appear to have efficacy and convenience

advantages versus current therapies, but may also carry novel safety and

tolerability concerns. The decision to use these new therapies will most likely

be based on an overall assessment of efficacy, safety, tolerability and

adherence, the potential need for monitoring and cost effectiveness. The

objective of this article is to review the currently available data for each of

these new oral therapies, which addresses the mechanism of action, efficacy and

safety, and to provide a perspective on the potential future role of these

therapies within clinical practice. Although better patient compliance is

expected with the oral agents compared with the injectables, the safety profiles

of these new oral drugs will have to be watched carefully.

DOI: 10.2165/11539820-000000000-00000

PMID: 21128693 [Indexed for MEDLINE]

376. Core Evid. 2011;6:13-21. doi: 10.2147/CE.S10101. Epub 2011 Jan 6.

Evidence-based assessment of potential use of fingolimod in treatment of

Excluded b/c no primary data (narrative review)

relapsing multiple sclerosis.

Portaccio E(1).

Author information:

(1)Department of Neurology, University, of Florence, Florence, Italy.

Multiple sclerosis is an autoimmune inflammatory demyelinating disease of the

central nervous system and represents one of the most common causes of chronic

neurologic disability in young adults. All the current disease-modifying drugs

are administered parenterally, and can be associated with varying degrees of

injection site or infusion-related reactions. Together with other side effects,

the parenteral route of administration is one of the key factors affecting

adherence to therapy in multiple sclerosis. Fingolimod (FTY720) is an

immunomodulator that acts on sphingosine 1-phosphate (S1P) receptors and is the

first oral drug approved by the US Food and Drug Administration for the treatment

of relapsing-remitting multiple sclerosis. Downmodulation of S1P receptor type 1

(S1P(1)) slows the egress of lymphocytes from lymph nodes and recirculation to

the central nervous system, reduces astrogliosis, and inhibits angiogenesis

during chronic neuroinflammation. Fingolimod also regulates the migration of B

cells and dendritic cells, and enhances endothelial barrier function. Results

from Phase II and III clinical trials provide robust evidence of the efficacy of

fingolimod in relapsing-remitting multiple sclerosis. While some caution should

be exercised in terms of safety issues, the introduction of fingolimod represents

a great advance in the treatment of relapsing-remitting multiple sclerosis. The

pharmacologic data on fingolimod and its efficacy and safety in multiple

sclerosis are reviewed in this paper.

DOI: 10.2147/CE.S10101

PMCID: PMC3065557

PMID: 21468239

377. Eur J Neurol. 2011 Jan;18(1):69-77. doi: 10.1111/j.1468-1331.2010.03110.x.

Excluded b/c no oral DMD results

The Global Adherence Project (GAP): a multicenter observational study on

adherence to disease-modifying therapies in patients with relapsing-remitting

multiple sclerosis.

Devonshire V(1), Lapierre Y, Macdonell R, Ramo-Tello C, Patti F, Fontoura P,

Suchet L, Hyde R, Balla I, Frohman EM, Kieseier BC; GAP Study Group.

Collaborators: Ballario C, Bestoso S, Caceres F, Carra A, Fernandez J, Fiol M,

Garcia O, Piedrabuena R, Sinay V, Barnett M, Beran R, Butler E, Butzkueven H,

Cameron J, Crimmins D, Macdonell R, Sandstrom P, Schwartz R, Sedal L, Serisier D,

Spira P, Sturm J, Waddy H, Yiannikas C, Baumhackl U, Höger F, Strasser-Fuchs S,

Vass K, Wörle C, Bartholome E, Decoo D, Dubois B, Mendes M, Arts R, Attig E, Bhan

V, Bouchard J, Brunet D, Charest S, Christie S, Cote J, Devonshire V, Esmail S,

Lapierre Y, Maclean G, Melanson M, Paulseth J, Veloso F, Witt N, Fiedler J, Hönig

I, Mares J, Pitha J, Vachova M, Frederiksen J, Sperling B, Stenager E, Clavelou

P, Lauxerois L, Maillet-Vioud M, Pelletier P, Pittion-Vouyovitch P, Suchet L,

Blodau A, Bode L, Boldt H, Bollensen E, Buchner H, Elias W, Emskötter T, Faiss J,

Freidel M, Gottwald K, Haupts M, Heidenreich F, Hofman W, Kaeder M, Käfferlein W,

Kausch U, Kieseier B, Köhler J, Kotterba S, Lang M, Lensch E, Meier U, Nadjafi U,

Oelmann H, Otto V, Putzki N, Reifschneider G, Reske D, Theis O, Wildemann B,

Windhagen S, Ziebold U, Zotter J, Abedini M, Abolfazli R, Ebrahimi Meimandi H,

Etemadifar M, Ghaffarpour M, Hatamian H, Khandaghi R, Lotfi J, Mazaher S,

Moghaddasi M, Motamed M, Nabavi S, Najlorahim A, Nikkhah K, Nikseresht A,

Shaghaghi H, Sharafoddinzadeh N, Tabassi A, Tabatabaee S, Ziaie J, Murphy R,

Karni A, Karussis D, Mannu L, Millefiorini E, Patti F, Quattrone A, Sosso L,

Llamas L, Núnez L, Anten B, Jongen S, Moll J, Basílio C, Carvalho M, Figueiredo

J, Fontoura P, Ginestal R, Martins A, Monteiro E, Morganho A, Pato A, Pedrosa R,

Rente S, Silva A, Agüera E, Andrés de Frutos C, Ángel Hernandez Fdez M, García

CA, Benavides AB, Bujanda B, Boakin WD, Ruiz LB, Gutierrez JC, Lorenzo PC,

González M, Grau L, Fernández MG, Hernández MA, Ayuso GI, Lacruz-Bescós F,

Martínez E, Martínez M, Sola EM, Merchán M, Munteis E, Sempere AP, Ramió i

Torrento L, Ramo-Tello C, Renedo F, González JR, Sánchez V, López FS, Izquierdo

AY, Andersson M, Borre B, Dahle C, Kaugessar T, Landtblom A, Martin C, Jensen SM,

Nilsson P, Radberg J, Svenningsson A, Träff J, Müller M, Roth S, Hobart J, Soto

A.

Author information:

(1)University of British Columbia Hospital MS Clinic, Canada.

Comment in

Eur J Neurol. 2011 Jan;18(1):1-2.

BACKGROUND: most disease-modifying therapies (DMTs) for multiple sclerosis (MS)

are self-injectable medications that must be taken on an ongoing basis to reduce

disease activity. Thus, adherence to therapy becomes an important challenge that

must be addressed to maximize benefits of therapy. This study evaluated rates of

adherence to prescribed treatment and explored factors affecting adherence

amongst patients with relapsing-remitting MS.

METHODS: this was an observational, multicenter, multinational, phase 4 study.

Patients and physicians received paper questionnaires regarding adherence to DMTs

approved at the time of the study, including intramuscular interferon beta-1a

(IFNβ-1a), subcutaneous IFNβ-1a, IFNβ-1b, and glatiramer acetate. Quality of life

and cognition data also were collected. Multivariate analysis was conducted to

identify factors associated with adherence to long-term DMTs.

RESULTS: two thousand six hundred and forty-eight patients were studied,

revealing an average treatment duration of 31 months. Seventy-five percent of

patients (n = 1923) were adherent to therapy. The most common reasons for

non-adherence were forgetting to administer the injection (50.2%) and other

injection-related reasons (32.0%). Adherent patients reported better quality of

life (P < 0.05) and fewer neuropsychological issues (P < 0.001) than non-adherent

patients. Adherent patients had significantly shorter duration of disease (P <

0.001) and shorter duration of therapy (P = 0.005) than non-adherent patients.

Women were more likely than men to adhere to treatment.

CONCLUSION: identifying factors that affect adherence to prescribed treatments is

the first step in improving adherence of patients with MS to therapy, thereby

helping maximize the benefits of long-term DMTs.

DOI: 10.1111/j.1468-1331.2010.03110.x

PMID: 20561039 [Indexed for MEDLINE]

Excluded b/c no primary data (expert opinion)

378. Eur J Neurol. 2011 Jan;18(1):1-2. doi: 10.1111/j.1468-1331.2010.03111.x.

'Let's stick together' Adherence to disease-modifying therapies in multiple

sclerosis.

Berger T.

Comment on

Eur J Neurol. 2011 Jan;18(1):69-77.

DOI: 10.1111/j.1468-1331.2010.03111.x

PMID: 20550560 [Indexed for MEDLINE]

379. Eur Neurol. 2011;65(2):59-67. doi: 10.1159/000323216. Epub 2011 Jan 4.

Excluded b/c no oral DMD results

Adherence to disease-modifying therapies in spanish patients with relapsing

multiple sclerosis: two-year interim results of the global adherence project.

Arroyo E(1), Grau C, Ramo-Tello C, Parra J, Sánchez-Soliño O; GAP Study Group.

Collaborators: Ramo C, Grau L, Roquer J, Munteis E, Martínez E, Ramió i Torrento

LL, Merchán M, Brieva L, Lacruz F, Bujanda M, de Castro P, Marzo E, Hernández MA,

González M, de Andrés C, Martínez M, Izquierdo G, Sánchez F, Agüera E, Sánchez V,

Guerrero M, Arnal C, Bowakin D, Renedo F, Cacho J, Yusta A, Pérez A, Belenguer A.

Author information:

(1)Biogen Idec Iberia SL, Madrid, Spain. estefania.arroyo@biogenidec.com

The post-marketing international Global Adherence Project investigated adherence

to disease-modifying therapy for relapsing-remitting multiple sclerosis. We

report adherence data from the first 2 years in the Spanish subset of patients (n

= 254 at baseline). The overall adherence rate was 85.4%. Patients taking

intramuscular (IM) interferon-β (IFNβ)-1a were significantly more adherent

(96.4%) compared with patients taking subcutaneous (SC) IFNβ-1a 22 μg (79.1%; p =

0.0064), SC IFNβ-1a 44 μg (79.6%; p = 0.0064) and glatiramer acetate (82.7%; p =

0.0184). At year 1 (n = 142), the overall adherence rate was 86.6%. Patients on

IM IFNβ-1a were significantly more adherent than patients on SC IFNβ-1a 22 μg

(93.9 vs. 66.7%; p = 0.0251). At year 2 (n = 131), the overall adherence rate was

82% (87.5% for IM IFNβ-1a, 80.0% for SC IFNβ-1a 22 μg, 77.8% for SC IFNβ-1a 44

μg, 85.2% for IFNβ-1b, and 80.0% for glatiramer acetate). In conclusion,

adherence remained high among all disease-modifying therapies over the first 2

years of the study and was significantly higher for IM IFNβ-1a, at visit 1,

compared with SC IFNβ-1a.

Copyright © 2011 S. Karger AG, Basel.

DOI: 10.1159/000323216

PMID: 21212677 [Indexed for MEDLINE]

380. J Med Econ. 2011;14(5):617-27. doi: 10.3111/13696998.2011.602444. Epub 2011 Jul

Excluded b/c no real-world adherence/persistence

22.

Cost-effectiveness of natalizumab versus fingolimod for the treatment of

relapsing multiple sclerosis.

O'Day K(1), Meyer K, Miller RM, Agarwal S, Franklin M.

Author information:

(1)Xcenda, Palm Harbor, FL, USA.

BACKGROUND: With the addition of new agents for the treatment of multiple

sclerosis (MS) (e.g., fingolimod), there is a need to evaluate the relative value

of newer therapies in terms of cost and effectiveness, given healthcare resource

constraints in the United States.

OBJECTIVE: To assess the cost-effectiveness of natalizumab vs fingolimod in

patients with relapsing MS.

METHODS: A decision analytic model was developed to estimate the incremental cost

per relapse avoided of natalizumab and fingolimod from a US managed care payer

perspective. Two-year costs of treating patients with MS included drug

acquisition costs, administration and monitoring costs, and costs of treating MS

relapses. Effectiveness was measured in terms of MS relapses avoided (data from

AFFIRM and FREEDOMS trials). One-way and probabilistic sensitivity analyses were

conducted to assess uncertainty.

RESULTS: Mean 2-year estimated treatment costs were $86,461 (natalizumab) and

$98,748 (fingolimod). Patients receiving natalizumab had a mean of 0.74 relapses

avoided per 2 years vs 0.59 for fingolimod. Natalizumab dominated fingolimod in

the incremental cost-effectiveness analysis, as it was less costly and more

effective in reducing relapses. One-way sensitivity analysis showed the results

of the model were robust to changes in drug acquisition costs, administration

costs, and costs of treating MS relapses. Probabilistic sensitivity analysis

showed natalizumab was cost-effective 95.1% of the time, at a willingness-to-pay

(WTP) threshold of $0 per relapse avoided, increasing to 96.3% of the time at a

WTP threshold of $50,000 per relapse avoided.

LIMITATIONS: Absence of data from direct head-to-head studies comparing

natalizumab and fingolimod, use of relapse rate reduction rather than sustained

disability progression as primary model outcome, assumption of 100% adherence to

MS treatment, and not capturing adverse event costs in the model.

CONCLUSIONS: Natalizumab dominates fingolimod in terms of incremental cost per

relapse avoided, as it is less costly and more effective.

DOI: 10.3111/13696998.2011.602444

PMID: 21777161 [Indexed for MEDLINE]

381. Mult Scler Int. 2011;2011:271321. doi: 10.1155/2011/271321. Epub 2011 Aug 2.

Excluded b/c no oral DMD results

The impact of comorbid depression on adherence to therapy for multiple sclerosis.

Tarrants M(1), Oleen-Burkey M, Castelli-Haley J, Lage MJ.

Author information:

(1)Medical Affairs, Teva Pharmaceuticals, 901 East 104th Street, Suite 900,

Kansas City, MO 64131, USA.

Objective. Examine the impact of comorbid depression on adherence to

disease-modifying therapy (DMT) for multiple sclerosis (MS). Methods. A

retrospective database was used to identify patients with MS treated with a DMT.

Patients with MS and comorbid depression were matched to patients with MS only.

Adherence to DMT was proxied by the medication possession ratio (MPR) and

multivariate regressions were used to examine the association between comorbid

depression and adherence to DMT. Results. Patients with comorbid depression had a

10 point lower MPR (P < 0.01) and were less likely to achieve a MPR of at least

80% (odds ratio (OR) = 0.55; 95% confidence interval (CI) 0.42-0.74) than those

without depression. While treatment with an antidepressant generally had no

significant impact on the likelihood of achieving an MPR threshold of 80% (OR =

1.32; 95% CI 0.50-3.48), adherence to antidepressant therapy guidelines were

associated with improved adherence to DMT therapy. Conclusions. MS patients with

comorbid depression were approximately half as likely to be adherent to their DMT

relative to patients with MS without depression. Although treatment with

antidepressant therapy generally did not improve the likelihood of adherence,

treatment with antidepressants for at least 6 months was associated with better

adherence to DMT.

DOI: 10.1155/2011/271321

PMCID: PMC3196992

PMID: 22096632

382. Patient Prefer Adherence. 2011;5:629-43. doi: 10.2147/PPA.S26479. Epub 2011 Dec

Excluded b/c no oral DMD results

28.

The MS Choices Survey: findings of a study assessing physician and patient

perspectives on living with and managing multiple sclerosis.

Riñon A(1), Buch M, Holley D, Verdun E.

Author information:

(1)Merck Serono S.A. - Geneva, Switzerland, a branch of Merck Serono S.A.,

Coinsins, Switzerland, an affiliate of Merck KGaA, Darmstadt, Germany.

BACKGROUND: Treatment of multiple sclerosis (MS) with disease-modifying drugs

(DMDs) can reduce relapse frequency and delay disability progression. Although

adherence to DMDs is difficult to measure accurately, evidence suggests that poor

adherence is common and can compromise treatment success. There are likely to be

multiple factors underlying poor adherence. To better understand these factors,

the global MS Choices Survey investigated patient and physician perspectives

regarding key aspects of MS diagnosis, treatment adherence and persistence, and

disease management.

METHODS: The survey was conducted in seven countries and involved patients with

MS (age 18-60 years; MS diagnosis for ≥1 year; current treatment with a DMD) and

physicians (neurologist for 3-30 years; treating ≥15 patients with MS per average

month; >60% of time spent in clinical practice). Separate questionnaires were

used for physicians and patients, each containing approximately 30 questions.

RESULTS: Questionnaires were completed by 331 patients and 280 physicians.

Several differences were observed between the responses of patients and

physicians, particularly for questions relating to treatment adherence. Overall,

the proportion of patients reporting taking a treatment break (31%) was almost

twice that estimated by physicians (on average 17%). The reasons cited for poor

adherence also differed between patients and physicians. For example, more

physicians cited side effects as the main reason for poor patient adherence

(82%), than responding patients (42%).

CONCLUSIONS: Physicians may underestimate the scale of poor adherence to DMDs,

which could impact on their assessment of treatment efficacy and result in

inappropriate treatment escalation. In addition, disparities were identified

between patient and physician responses regarding the underlying reasons for poor

adherence. Improvements in the dialog between patients and neurologists may

increase adherence to DMDs.

DOI: 10.2147/PPA.S26479

PMCID: PMC3259078

PMID: 22259240

Excluded b/c no real-world adherence/persistence

383. Clin Immunol. 2010 Oct;137(1):15-20. doi: 10.1016/j.clim.2010.06.005.

Reconstitution of circulating lymphocyte counts in FTY720-treated MS patients.

Johnson TA(1), Shames I, Keezer M, Lapierre Y, Haegert DG, Bar-Or A, Antel J.

Author information:

(1)Neuroimmunology Unit, Montreal Neurological Institute, McGill University,

Montreal, Quebec, Canada.

FTY720 (Fingolimod) reduces multiple sclerosis disease activity by inducing

lymphopenia and inhibiting lymphocyte re-entry from lymph nodes. Peripheral

lymphocyte reconstitution following drug discontinuation has been considered

relatively rapid (2-4 weeks), based on short-term studies. We investigated the

kinetics of lymphocyte reconstitution in MS patients in open label extension

phases of FTY720 clinical trials who discontinued therapy after prolonged use

(>1-5 years), and examined histological features of a mediastinal lymph node

obtained from a lymphopenic FTY720 patient. Although three patients showed

reconstitution of peripheral lymphocytes within the predicted timeline, two

patients continued to be lymphopenic 9 and 34 months after therapy cessation.

Lymph nodes from the latter patient showed preserved architecture.

Notwithstanding preserved lymph node integrity, time for lymphocyte

reconstitution after prolonged FTY720 therapy can be significantly greater than

predicted by shorter-term studies. This is relevant for clinical decisions

regarding management of patients using this therapy and for introducing alternate

therapies.

Copyright © 2010 Elsevier Inc. All rights reserved.

DOI: 10.1016/j.clim.2010.06.005

PMID: 20599429 [Indexed for MEDLINE]

Excluded b/c no primary data (narrative review)

384. J Neurosci Nurs. 2010 Oct;42(5 Suppl):S19-29.

Enhancing adherence through education.

Smrtka J(1), Caon C, Saunders C, Becker BL, Baxter N.

Author information:

(1)Ft. Lauderdale MS Center, Pompano Beach, FL, USA.

jsmrtka.fusionhealth@gmail.com

The treatment of multiple sclerosis (MS) has advanced greatly since the

introduction of disease-modifying therapies (DMTs) in the early 1990s. Although

the DMTs have exhibited significant efficacy in relapsing-remitting MS and other

forms of the disease, the degree of benefit depends heavily on patient adherence

to recommended regimens. This article addresses some of the most pressing areas

of unmet need in educating advanced-practice nurses, neurologists, patients, and

support care partners regarding strategies that can overcome obstacles to

adherence. The observations presented here are based on clinical experience with

real-life cognitive, psychosocial, and cultural impediments to adherence. The

article also explores the ways in which adherence may be affected by emerging

therapies for MS (such as oral agents) as well as the educational needs that will

arise with the further evolution of MS care.

PMID: 21049830 [Indexed for MEDLINE]

385. J Neurosci Nurs. 2010 Oct;42(5 Suppl):S10-8.

Excluded b/c no primary data (narrative review)

Factors that influence adherence and strategies to maintain adherence to injected

therapies for patients with multiple sclerosis.

Saunders C(1), Caon C, Smrtka J, Shoemaker J.

Author information:

(1)carol@saunders.net

A key aspect of the management of care for patients with multiple sclerosis (MS)

is identifying which patients are struggling with adherence. Disease-modifying

therapies for the treatment of MS are primarily preventative, with modest

efficacy, and they can possibly cause significant side effects. These factors can

lead patients to stop taking their medication because they continue to experience

symptoms or relapses of the disease, and/or they have painful injection-site

reactions on most parts of their bodies. However, striving for 100% adherence to

the prescribed treatment regimen remains the goal and is the surest way to reap

all the benefits associated with immunomodulatory therapy. Building trust,

promoting injection self-efficacy, and educating patients about proper injection

techniques to prevent injection-site reactions have the greatest impact on

patient adherence. Factors including family involvement, financial stability and

support, hope, and faith can also have a positive impact on adherence.

Identifying patients who may be struggling with depression, which is easily

treatable but often not well addressed, and providing access to mental health

services are important. Although many strides have been made with regard to MS

patient care, outlining strategies that can maintain or encourage adherence can

provide practitioners with tools to facilitate their patients' health and

well-being.

PMID: 21049829 [Indexed for MEDLINE]

386. J Neurosci Nurs. 2010 Oct;42(5 Suppl):S5-9.

Excluded b/c no primary data (narrative review)

Injectable disease-modifying therapy for relapsing-remitting multiple sclerosis:

a review of adherence data.

Caon C(1), Saunders C, Smrtka J, Baxter N, Shoemaker J.

Author information:

(1)Multiple Sclerosis Center at Wayne State University School of Medicine,

Detroit, MI, USA. crcaon@med.wayne.edu

Long-term adherence to disease-modifying therapy in relapsing-remitting multiple

sclerosis (RRMS) is associated with improved patient outcomes, including a

reduced risk of relapse and a better preserved quality of life. However, the

unpredictable nature of the disease--even when it is being treated--may make it

difficult to convince patients of the importance of treatment adherence. A number

of studies have attempted to pinpoint factors that affect adherence. Nursing

interventions that address some of these factors may improve adherence and, thus,

the disease course for a variety of RRMS patients. This article summarizes

literature that approximates the prevalence and impact of nonadherence and

reviews factors identified in clinical trials that affect adherence. Nursing

interventions that can improve adherence, including telephone counseling and

motivational interview techniques, are also addressed.

PMID: 21049828 [Indexed for MEDLINE]

387. J Neurosci Nurs. 2010 Oct;42(5 Suppl):S1-4.

Excluded b/c no primary data (narrative review)

Introduction: adherence to disease-modifying therapies--key to optimizing

outcomes in relapsing multiple sclerosis.

Caon C(1), Saunders C, Smrtka J, Davis K, Davis A, Kay JM, Baxter N.

Author information:

(1)Multiple Sclerosis Center at Wayne State University School of Medicine,

Detroit, MI, USA. crcaon@med.wayne.edu

PMID: 21049827 [Indexed for MEDLINE]

388. Mult Scler. 2010 Oct;16(10):1258-67. doi: 10.1177/1352458510375568. Epub 2010 Aug

Excluded b/c no primary data (narrative review)

4.

The management of multiple sclerosis in children: a European view.

Ghezzi A(1), Banwell B, Boyko A, Amato MP, Anlar B, Blinkenberg M, Boon M,

Filippi M, Jozwiak S, Ketelslegers I, Kornek B, Lim M, Lindstrom E, Nadj C,

Neuteboom R, Rocca MA, Rostasy K, Tardieu M, Wassmer E, Catsman-Berrevoets C,

Hintzen R.

Author information:

(1)Centro Studi SM, H S. Antonio Abate, Gallarate, VA, Italy.

About 3-5% of all patients with multiple sclerosis experience the onset of their

disease under the age of 16. A significant proportion of paediatric multiple

sclerosis patients develop significant cognitive disturbances and persistent

physical disability. The high relapse rate and the morbidity in the paediatric

multiple sclerosis population has triggered the use of disease-modifying

therapies that have been shown to reduce relapse rate, disease progression and

cognitive decline in adult patients with multiple sclerosis. Hard evidence for

the right treatment and its appropriate timing is scarce in paediatric multiple

sclerosis. Nevertheless, expertise in this field has grown thanks to recent

open-label trials and experience generated in specialized centres. In spring

2009, a first meeting was held in Rotterdam with clinicians from 11 European

countries (one from Canada) that are all active in the management of paediatric

multiple sclerosis. One of the aims was to generate a common view on the

management of paediatric multiple sclerosis patients. The result of this meeting

is presented here to help standardize treatment and to support clinicians with

less experience in this field.

DOI: 10.1177/1352458510375568

PMID: 20685764 [Indexed for MEDLINE]

Excluded b/c no primary data (narrative review)

389. Neuropsychiatr Dis Treat. 2010 Sep 7;6:365-73.

Disease-modifying therapies in relapsing-remitting multiple sclerosis.

González-Andrade F(1), Alcaraz-Alvarez JL.

Author information:

(1)Department of Medicine, Metropolitan Hospital, Av. Mariana de Jesús Oe8 y

Occidental, 170125, Quito, Ecuador. fabriciogonzaleza@yahoo.es

CLINICAL QUESTION: What is the best current disease-modifying therapy for

relapsing-remitting multiple sclerosis?

RESULTS: The evidence shows that the most effective disease-modifying therapy for

delaying short- to medium-term disability progression, prevention of relapses,

reducing the area and activity of lesions on magnetic resonance imaging, with the

least side effects, is high-dose, high-frequency subcutaneous interferon-β1a 44

μg three times per week.

IMPLEMENTATION: The pitfalls in treatment of MS can be avoided by remembering the

following points: The most effective therapy to prevent or delay the appearance

of permanent neurological disability with the fewest side effects should be

chosen, and treatment should not be delayed.Adherence to treatment should be

monitored closely, and needs comprehensive patient information and education to

establish long-term adherence, which is a critical determinant of long-term

outcome.The correct approach to the disease includes disease management, symptom

management, and patient management. A combination of tools is necessary to ease

the various symptoms, which fall into three broad categories, i.e.

rehabilitation, pharmacological, and procedural.It is important to understand

that no treatment modality should be used alone, unless it is in itself

sufficient to remedy the particular symptom/problem.

PMCID: PMC2938285

PMID: 20856600

390. Ther Clin Risk Manag. 2010 Sep 7;6:391-9.

Excluded b/c no primary data (narrative review)

Emerging oral treatments in multiple sclerosis - clinical utility of cladribine

tablets.

Gasperini C(1), Ruggieri S, Pozzilli C.

Author information:

(1)Department of Neurosciences, S Camillo Forlanini Hospital, Rome, Italy;

Multiple sclerosis (MS) is a chronic inflammatory disorder of the central nervous

system (CNS) that represents one of the first causes of neurological disability

in young adults. Although the pathogenesis of MS is still unclear, an autoimmune

mechanism has been demonstrated. According to this evidence in the last 15 years

different treatments acting on the immune system have been developed. Current

disease-modifying drugs (DMDs) for MS require regular and frequent parenteral

administration and are associated with limited long-term treatment adherence.

Moreover the clinical efficacy of these disease-modifying drugs is suboptimal.

Thus, there is an important need for the development of new therapeutic

strategies. Several oral therapies (fingolimod, fumaric acid, teriflunomide,

laquinimod) are in development; Among these cladribine is the only therapy with

the potential for short-course dosing. Cladribine is an immunosuppressant that

offers sustained regulation of the immune system through a preferential

lymphocyte depleting action. Cladribine has a well-characterized and well-known

safety profile, derived from more than 15 years of use of the parenteral

formulation both in the oncology field and in MS. This paper reviews the new oral

emerging treatments and presents the available data about the use of cladribine

in MS and the future perspective of its clinical use.

PMCID: PMC2940747

PMID: 20856685

391. Am J Manag Care. 2010 Sep;16(8 Suppl):S227-33.

Excluded b/c no primary data (narrative review)

Will the newer oral MS agents be welcomed by managed care organizations?

Lipsy RJ(1).

Author information:

(1)University of Arizona, College of Pharmacy, 8530 E Green Acres Dr, Tucson, AZ

85715, USA. lipsy@pharmacy.arizona.edu

Of the new generation of multiple sclerosis (MS) drugs, 4 oral

agents--dalfampridine, laquinimod, cladribine, and fingolimod--could produce

significant changes in the treatment landscape for MS. Current first-line

treatments, which are administered via injection, are associated with poor

treatment adherence, often due to lack of efficacy (perceived and real), adverse

drug reactions, cost, and injection anxiety. Although concerns about safety and

cost remain, preliminary results indicate that these oral agents are as effective

as, or even more effective than, current injectable treatments. Oral MS agents

are expected to cost patients less in out-of-pocket expenses, which will likely

increase treatment adherence and lead to an overall reduction in medical costs.

While many patients may prefer an oral MS drug, the ultimate choice of therapy

will be a shared patient-physician decision based on a multitude of factors,

including the efficacy of the current treatment regimen, patient compliance

history, and the difference in out-of-pocket expenses.

PMID: 20873947 [Indexed for MEDLINE]

392. Am J Manag Care. 2010 Sep;16(8 Suppl):S219-26.

Excluded b/c no primary data (narrative review)

Emerging oral agents for multiple sclerosis.

Fox EJ(1).

Author information:

(1)Central Texas Neurology Consultants, 16040 Park Valley Dr, Bldg B, Ste 100,

Round Rock, TX 78681, USA. foxtexms@gmail.com

A variety of emerging therapies for the treatment of multiple sclerosis (MS) are

currently in development or have recently been approved by the US Food and Drug

Administration (FDA). These new agents offer novel mechanisms of action and

potentially improved efficacy over existing first-line MS therapies. Much

attention has been given to emerging therapies delivered orally which, at

minimum, will likely improve long-term adherence over existing agents delivered

via injection. This article reviews the mechanisms of action, efficacy, and

safety and tolerability of 4 emerging oral therapies for MS: cladribine,

laquinimod, fingolimod, and dalfampridine. The first 3 of these agents are in

late development and may enter the market within the next year and a half.

Cladribine, laquinimod, and fingolimod have demonstrated impressive efficacy in

terms of clinical outcomes, such as annualized relapse rate and change in

disability scores, as well as magnetic resonance imaging variables.

Dalfampridine, which has already been approved by the FDA, is indicated as a

symptomatic therapy to improve walking in MS patients. Based on existing data,

these agents appear to have tolerable side-effect profiles, although the

long-term safety profiles of these drugs have yet to be elucidated. It remains to

be seen whether the safety profiles of these disease-modifying drugs will allow

them to displace existing first-line therapies or if agents such as dalfampridine

will become additional options alongside current dominant therapies.

PMID: 20873946 [Indexed for MEDLINE]

393. Am J Manag Care. 2010 Sep;16(8 Suppl):S211-8.

Excluded b/c no primary data (narrative review)

The current landscape and unmet needs in multiple sclerosis.

Markowitz CE(1).

Author information:

(1)Multiple Sclerosis Center, University of Pennsylvania, 3400 Spruce St, 3 W

Gates Bldg, Philadelphia, PA 19104, USA. cmarkowi@mail.med.upenn.edu

When introduced in the early and middle 1990s, current first-line pharmacologic

therapies for multiple sclerosis (MS)--interferon beta-1a, interferon beta-1b,

and glatiramer acetate--constituted a major advancement in MS treatment.

Nevertheless, disease progression, although typically delayed with these agents,

remains inevitable in most patients and constitutes a significant limitation of

the currently available treatments. Moreover, the first-line therapies all

require frequent subcutaneous or intramuscular injections, delivery modalities

that are associated with subpar treatment adherence. The demand for more

effective agents has produced a new generation of MS therapies with impressive

efficacy profiles--although their long-term safety and tolerability remain

largely unknown. Some of the new agents have been formulated for oral

administration, which will likely have a positive impact on treatment adherence.

These new agents are appearing during a time of major change in MS research. As

the old expectation of inevitable disease progression is being reconsidered, the

notion of sustained disease inactivity has become a credible, still somewhat

elusive, goal. Neuroprotection may also be possible with new and existing

treatments. At the same time, new imaging techniques, such as measuring disease

progression via T1-hypointense lesions ("black holes"), and a better

understanding of pathophysiologic factors in MS--such as the role of neurotrophic

growth factors and oxidative stress--are changing the ways that efficacy is

measured and how new agents are developed.

PMID: 20873945 [Indexed for MEDLINE]

Excluded b/c no oral DMD results

394. Ann Neurol. 2010 Sep;68(3):392-5. doi: 10.1002/ana.22074.

Natalizumab drug holiday in multiple sclerosis: poorly tolerated.

Killestein J(1), Vennegoor A, Strijbis EM, Seewann A, van Oosten BW, Uitdehaag

BM, Polman CH.

Author information:

(1)Department of Neurology, MS Center Amsterdam, VU University Medical Center,

Amsterdam, the Netherlands. j.killestein@vumc.nl

It has been suggested that natalizumab-associated progressive multifocal

leukoencephalopathy may be prevented by structured interruptions of treatment.

Evidence supporting such a drug holiday is not yet available. Here we present

initial observations in 10 multiple sclerosis patients who were stringently

monitored up to 6 months after discontinuation of the infusions. Cumulatively, a

combination of clinical relapse and new and/or enhanced lesions on magnetic

resonance imaging had occurred in 7 of 10 patients. Although numbers are small,

our data suggest that in patients who were switched to natalizumab because of

disease activity despite first-line treatment, a natalizumab drug holiday without

reinstatement of alternate disease-modifying therapy is poorly tolerated.

DOI: 10.1002/ana.22074

PMID: 20661928 [Indexed for MEDLINE]

395. Pharmacotherapy. 2010 Sep;30(9):916-27. doi: 10.1592/phco.30.9.916.

Excluded b/c no primary data (narrative review)

Treatment options for multiple sclerosis: current and emerging therapies.

Gawronski KM(1), Rainka MM, Patel MJ, Gengo FM.

Author information:

(1)Department of Pharmacy Practice, University at Buffalo, Buffalo, New York,

USA.

Multiple sclerosis is a chronic autoimmune disease affecting the central nervous

system, more specifically, the myelin sheath covering of nerve fibers in the

brain and spinal cord. This disease requires lifelong disease-modifying therapy,

and all of the currently available first-line disease-modifying agents are

parenteral formulations only. To date, eight drugs have entered or completed

phases II and III clinical trials, four of which are oral drugs. These include

five immunomodulators--cladribine, fingolimod, laquinimod, teriflunomide, and

dimethyl fumarate--and three monoclonal antibodies--alemtuzumab, daclizumab, and

rituximab. Although comparing these new drugs with available therapies is

difficult, they do show promise as potential first-line agents for the treatment

of multiple sclerosis. This marks a new frontier in the treatment of this

disease, as the advent of new oral drugs will lead to increased patient

compliance and contribute to longer sustained symptom-free periods and less

marked disability.

DOI: 10.1592/phco.30.9.916

PMID: 20795847 [Indexed for MEDLINE]

396. Mol Nutr Food Res. 2010 Aug;54(8):1164-71. doi: 10.1002/mnfr.200900494.

The estimated benefits of vitamin D for Germany.

Excluded b/c no primary data (narrative review)

Zittermann A(1).

Author information:

(1)Clinic for Thoracic and Cardiovascular Surgery, Heart Center North

Rhine-Westphalia, Ruhr University Bochum, Bad Oeynhausen, Germany.

azittermann@hdz-nrw.de

This article gives an overview of the vitamin D status in Germany, provides

evidence for an independent association of vitamin D deficiency with various

chronic diseases, and discusses preventive measures for improving vitamin D

status in Germany. The prevalence of vitamin D insufficiency is 40-45% in the

general German population. An additional 15-30% are vitamin D deficient. Vitamin

D can prevent falls and osteoporotic fractures in older people. There is also

accumulating evidence that vitamin D may prevent excess mortality and may

probably prevent some chronic diseases that occur in early life such as type 1

diabetes and multiple sclerosis. Adherence to present sun safety policy

(avoidance of the sun between 11 am and 3 pm) and dietary recommendations (5-10

microg daily for adults) would, however, definitively lead to vitamin D

deficiency. The estimated cost saving effect of improving vitamin D status in

Germany might be up to 37.5 billion euro annually. It should be the goal of

nutrition and medical societies to erase vitamin D deficiency in Germany within

the next 5-10 years. To achieve this goal, the daily production of at least 25

microg of vitamin D in the skin or an equivalent oral intake should be

guaranteed.

DOI: 10.1002/mnfr.200900494

PMID: 20373291 [Indexed for MEDLINE]

397. Nat Rev Neurol. 2010 Aug;6(8):421-2. doi: 10.1038/nrneurol.2010.88.

Excluded b/c no primary data (narrative review)

Multiple sclerosis: monitoring long-term treatments in multiple sclerosis.

Filippi M, Rocca MA.

Patients with relapsing-remitting multiple sclerosis have been shown to benefit

from disease-modifying treatments over prolonged periods. More focus should now

be placed on monitoring the long-term evolution of this disease (making use of

tools such as MRI), alongside the patients' response and adherence to such

treatments.

DOI: 10.1038/nrneurol.2010.88

PMID: 20689563 [Indexed for MEDLINE]

398. J Behav Med. 2010 Jun;33(3):219-27. doi: 10.1007/s10865-010-9247-y. Epub 2010 Feb

Excluded b/c no oral DMD results

3.

Treatment adherence in multiple sclerosis: association with emotional status,

personality, and cognition.

Bruce JM(1), Hancock LM, Arnett P, Lynch S.

Author information:

(1)Department of Psychology, University of Missouri-Kansas City, 4825 Troost

Building, Suite 111G, Kansas City, MO 64110, USA. brucejm@umkc.edu

The purpose of the present study was to prospectively examine the association

between treatment adherence and common neuropsychiatric symptoms in multiple

sclerosis (MS). Patients underwent a thorough psychiatric and neuropsychological

evaluation at the outset of the study. Patient adherence to disease modifying

therapies was then tracked for 8 weeks using self-report, a medication diary, and

an electronic monitoring device that recorded needle disposals. Results indicated

that MS patients with current mood or anxiety disorders were almost five times as

likely as MS patients with no psychiatric diagnosis to exhibit problems adhering

to their disease modifying therapies. Poor adherence was also associated with

memory difficulties, anxiety, depression, neuroticism, and low conscientiousness.

Findings highlight the importance of conducting a thorough psychiatric and

neuropsychological evaluation when clinicians suspect poor adherence to disease

modifying therapies. Pharmacological or psychotherapeutic treatment of

mood/anxiety disorders, use of scheduled reminders, and/or increased organization

and structure may lead to improved treatment adherence in MS.

DOI: 10.1007/s10865-010-9247-y

PMID: 20127401 [Indexed for MEDLINE]

399. BMC Neurol. 2010 Apr 30;10:28. doi: 10.1186/1471-2377-10-28.

Excluded b/c no real-world adherence/persistence

Patient-rated suitability of a novel electronic device for self-injection of

subcutaneous interferon beta-1a in relapsing multiple sclerosis: an

international, single-arm, multicentre, Phase IIIb study.

Devonshire V(1), Arbizu T, Borre B, Lang M, Lugaresi A, Singer B, Verdun di

Cantogno E, Cornelisse P.

Author information:

(1)Department of Neurology, University of British Columbia, Vancouver, BC,

Canada. vdev@shaw.ca

BACKGROUND: Multiple sclerosis (MS) currently requires long-term treatment with

disease-modifying drugs, administered parenterally up to once daily. The need for

regular self-injection can be a barrier to treatment for many patients.

Autoinjectors can help patients overcome problems or concerns with self-injection

and could, therefore, improve treatment adherence. This study was performed to

assess the suitability of a new electronic device for the subcutaneous (sc)

administration of interferon (IFN) beta-1a, 44 mcg three times weekly, for

relapsing MS.

METHODS: In this Phase IIIb, multicentre, single-arm study, patients with

relapsing MS who had been consistently self-injecting sc IFN beta-1a using an

autoinjector for at least 6 weeks were taught to use the new device and

self-administered treatment for 12 weeks thereafter. Patient-rated suitability of

the device was assessed at the end of Week 12 using the Patient User Trial

Questionnaire. Patient satisfaction with, and evaluation of, the injection

process was assessed using the MS Treatment Concern Questionnaire. Trainers

evaluated the device using the Trainer User Trial Questionnaire.

RESULTS: At Week 12, 71.6% (73/102) of patients considered the device 'very

suitable' or 'suitable' for self-injection; 92.2% (94/102) reported some degree

of suitability and only 7.8% (8/102) found the device 'not at all suitable'. At

Weeks 4, 8 and 12, most patients reported that injection preparation and

clean-up, performing injections and ease of device use in the previous 4 weeks

compared favourably with, or was equivalent to, their previous experience of

self-injection. Injection-related pain, injection reactions and 'flu-like'

symptoms remained stable over the 12 weeks. Each device feature was rated 'very

useful' or 'useful' by at least 80% of patients. All trainers and 95.2% (99/104)

of patients found device functions 'very easy' or 'easy' to use. Overall

convenience was considered the most important benefit of the device.

CONCLUSIONS: Most patients considered the new electronic injection device

suitable for the sc injection of IFN beta-1a. They found the device easy to use

with useful features, and reported benefits such as overall convenience. The

device may, therefore, increase treatment adherence in patients with MS,

particularly those with injection-related issues.

TRIAL REGISTRATION: NCT00735007.

DOI: 10.1186/1471-2377-10-28

PMCID: PMC2877661

PMID: 20433746 [Indexed for MEDLINE]

400. Clin Ther. 2010 Apr;32(4):717-28. doi: 10.1016/j.clinthera.2010.03.019.

Excluded b/c no real-world adherence/persistence

A health-economic evaluation of disease-modifying drugs for the treatment of

relapsing-remitting multiple sclerosis from the German societal perspective.

Nuijten M(1), Mittendorf T.

Author information:

(1)Ars Accessus Medica, Amsterdam, the Netherlands. nuijten@bmg.eur.nl

OBJECTIVE: This analysis compared the cost-effectiveness of interferon beta-1a

(IFNbeta-1a) 44 microg SC with that of other available first-line treatments for

relapsing-remitting multiple sclerosis (RRMS) from the German societal

perspective in 2008.

METHODS: A decision-analytic model was used to estimate the cost-effectiveness of

IFNbeta-1a 44 microg SC given 3 times weekly compared with that of IFNbeta-la 30

microg IM given once weekly, IFNbeta-1b 8 mIU given every other day, and

glatiramer acetate 20 mg SC given once daily. Data sources included the published

literature, clinical trials, German price/tariff lists, and national population

statistics. The time horizon of the model was 4 years, which was the maximum

duration of follow-up in published clinical trials.

RESULTS: The cost-effectiveness (cost per relapse avoided) of IFNbeta-la 44

microg SC compared with no active treatment was euro51,250, which compared

favorably with that of IFNbeta-la 30 microg IM (euro133,770), glatiramer acetate

(euro71,416), and IFNbeta-1b (euro54,475). When the cost of disease progression

was excluded, the cost per relapse avoided remained favorable for IFNbeta-1a 44

microg SC (euro54,292) compared with the other options (euro143,186, euro72,809,

and euro56,816, respectively). Indirect comparison of each available treatment

option with the next best alternative indicated that the incremental

cost-effectiveness of IFNbeta-la 44 microg SC (euro23,449) was consistent with

accepted thresholds. Sensitivity analyses in which the discount rate, frequency

of relapse and disease progression, costs of relapse and disease progression, and

adherence were varied did not affect the relative outcomes.

CONCLUSION: In this analysis from the German societal perspective, IFNbeta-la 44

microg SC had favorable overall cost-effectiveness versus no active treatment

compared with other available disease-modifying drugs for the treatment of RRMS.

DOI: 10.1016/j.clinthera.2010.03.019

PMID: 20435242 [Indexed for MEDLINE]

401. Int J Clin Pract. 2010 Apr;64(5):637-50. doi: 10.1111/j.1742-1241.2009.02261.x.

Excluded b/c no primary data (narrative review)

Current and future disease-modifying therapies in multiple sclerosis.

Lim SY(1), Constantinescu CS.

Author information:

(1)University of Nottingham, UK.

Comment in

Int J Clin Pract. 2010 Apr;64(5):532-4.

The development of disease-modifying therapies (DMT) in multiple sclerosis (MS)

has rapidly evolved over the last few years and continues to do so. Prior to the

United States Food and Drug Administration approval of the immunomodulatory

agent, interferon-beta1b in 1993, no other drug had been shown to alter the

course of the disease in a controlled study of MS. At present, there are five

licenced disease-modifying agents in MS - interferon-beta1b, interferon-beta1a,

glatiramer acetate, natalizumab and mitoxantrone. All have shown significant

therapeutic efficacy in large controlled trials. However, current therapies are

only partially effective and are not free from adverse effects. Moreover,

available DMTs are overwhelmingly biased in favour of those with

relapsing-remitting disease. Effective treatment for progressive MS is severely

limited, with only interferon-beta1b and mitoxantrone having licenced use in

secondary progressive, but not primary progressive disease. Monoclonal

antibodies, such as natalizumab selectively target immune pathways involved in

the pathogenic process of MS. Alemtuzumab, daclizumab and rituximab are other

notable monoclonal antibodies currently undergoing phase II and III trials in MS.

Alemtuzumab has so far shown promising therapeutic benefit in relapsing disease,

although immunological adverse effects have been a problem. Oral therapies have

the benefit of improved tolerability and patient compliance compared with current

parenteral treatments. Cladribine and fingolimod (FTY720) have shown encouraging

results in their phase III clinical trials. It is also worth noting the evidence

for starting DMT in patients with clinically isolated syndrome, whereby early

treatment has shown to delay the onset of clinically definite MS in separate

phase III studies.

DOI: 10.1111/j.1742-1241.2009.02261.x

PMID: 20456216 [Indexed for MEDLINE]

Excluded b/c no primary data (narrative review)

402. Int J Neurosci. 2010 Apr;120(4):273-9. doi: 10.3109/00207450903541087.

Adherence to disease-modifying drugs in patients with multiple sclerosis: a

consensus statement from the Middle East MS Advisory Group.

Yamout BI(1), Dahdaleh M, Al Jumah MA, Al-Shammri S, Al Sharoqi I, Al-Tahan AR,

Bohlega S, Deleu D, Inshasi J, Khalifa A, Szólics M.

Author information:

(1)Internal Medicine, American University of Beirut, Beirut, Lebanon.

yamoutba@idm.net.lb

Adherence to therapy is a key issue in chronic illnesses. In addition, several

features of multiple sclerosis (MS) and its treatment may increase the likelihood

of patient nonadherence and discontinuation of treatment. Nonadherence will

obviously compromise the efficacy of disease-modifying drugs in patients with MS.

This subject was discussed by a group of local MS specialists from the Middle

East. The group debated several key questions about the features and causes of

patient nonadherence and its management. Further, they made recommendations for

optimizing treatment adherence in this area.

DOI: 10.3109/00207450903541087

PMID: 20374075 [Indexed for MEDLINE]

403. J Manag Care Pharm. 2010 Apr;16(3):206-16.

Excluded b/c no real-world adherence/persistence

Gender differences in self-reported symptom awareness and perceived ability to

manage therapy with disease-modifying medication among commercially insured

multiple sclerosis patients.

Vlahiotis A(1), Sedjo R, Cox ER, Burroughs TE, Rauchway A, Lich R.

Author information:

(1)Express Scripts, Inc., 1 Express Way, St. Louis, MO 63121, USA.

avlahiotis@express-scripts.com

BACKGROUND: Multiple sclerosis (MS) is a chronic, neurodegenerative inflammatory

disease that affects approximately 400,000 Americans, the majority of whom are

female. Although MS prevalence is higher among females, males are more likely to

have a more progressive clinical course. For both genders, use of

disease-modifying medications (DMMs) in the clinical management of MS is pivotal

in altering the natural course and diminishing progressive disability over time.

OBJECTIVES: To evaluate gender differences in self-reported symptom awareness and

perceived ability to manage therapy among MS patients taking a DMM.

METHODS: During February 2008, a self-administered, 42-item survey was mailed to

4,700 commercially insured patients taking a DMM to treat MS. Survey items

measured self-reported clinical characteristics, symptom awareness, and perceived

ability to manage therapy. Bivariate analyses assessed associations of gender

with other predictor and outcome variables, including demographic

characteristics, clinical disease characteristics, specific DMM used at the time

of the survey, self-reported symptom awareness, and perceived ability to manage

therapy. Logistic regression analyses further assessed the associations of gender

with symptom awareness and perceived ability to manage MS after adjustment for

relevant covariates (age at diagnosis, educational level, income, current DMM,

type of pharmacy where drug was dispensed, frequency of flare-ups, and clinical

course of disease).

RESULTS: The response rate was 44.1% (n = 2,074). Of the 2,022 respondents with

useable surveys, 80.6% were female; 82.3% had relapsing remitting MS; and 83.1%

were taking one of the most commonly used DMMs (intramuscular interferon beta-1a

33.4%, subcutaneous interferon beta-1a 15.9%, and glatiramer acetate 33.8%).

Compared with female patients, males were older and a greater proportion had a

more progressive clinical course of disease. In multivariate models, female

patients were more likely than males to report recognition of a

relapse/exacerbation (odds ratio [OR] = 1.37, 95% CI = 1.03-1.82) and to report

knowing what to do when experiencing a relapse/exacerbation (OR = 1.34, 95% CI =

1.01- 1.77) or if they missed a dose of medication (OR = 1.78, 95% CI =

1.08-2.43). Females were also more likely to report awareness of treatment

options (OR = 1.48, 95% CI = 1.07-2.07) and to think that DMMs were helping their

MS (OR = 1.32, 95% CI = 1.02-1.77).

CONCLUSIONS: Female MS patients report better awareness of disease symptoms and

have more positive perceptions of their ability to manage therapy with DMMs than

male MS patients. These findings suggest that male MS patients may require

additional education and support to manage their disease and therapy needs.

Knowledge of these gender differences potentially could help managed care

organizations to improve therapy adherence by guiding gender-specific patient

support programs.

DOI: 10.18553/jmcp.2010.16.3.206

PMID: 20331325 [Indexed for MEDLINE]

404. BMC Ophthalmol. 2010 Mar 21;10:7. doi: 10.1186/1471-2415-10-7.

Excluded b/c no real-world adherence/persistence

Patients' knowledge and perception on optic neuritis management before and after

an information session.

Matti AI(1), Keane MC, McCarl H, Klaer P, Chen CS.

Author information:

(1)Department of Ophthalmology, Flinders Medical Centre, Flinders Drive, Bedford

Park SA 5042, Australia.

BACKGROUND: Patients' understanding of their condition affect the choice of

treatment. The aim of this study is to evaluate patients' understanding and

treatment preferences before and after an information session on the treatment of

acute optic neuritis.

METHODS: Participants were asked to complete a questionnaire consisting of 14

questions before and after an information session presented by a

neuro-ophthalmologist. The information session highlighted the treatment options

and the treatment effects based on the Optic Neuritis Treatment Trial in plain

patient language. The information session stressed the finding that high dose

intravenous steroid therapy accelerated visual recovery but does not change final

vision and that treatment with oral prednisone alone resulted in a higher

incidence of recurrent optic neuritis.

RESULTS: Before the information session, 23 (85%) participants knew that there

was treatment available for ON and this increased to 27 (100%) after the

information session. There were no significantly change in patients knowledge of

symptoms of ON and purpose of treatment before and after the information session.

Before the information session, 4 (14%) respondents reported they would like to

be treated by oral steroid alone in the event of an optic neuritis and 5 (19%)

did not respond. After the education session, only 1 patient (4%) indicated they

would undergo treatment with oral steroid alone but 25 (92%) indicated they would

undergo treatment with intravenous steroid treatment, alone or in combination

with oral treatment. Results indicated that there were significant differences in

the numbers of participants selecting that they would undergo treatment with a

steroid injection (n = 22, p = 0.016).

CONCLUSIONS: In this study, patients have shown good understanding of the

symptoms and signs of optic neuritis. The finding that significant increases in

the likelihood of patients engaging in best practice can be achieved with an

information session is very important. This suggests that patient knowledge of

available treatments and outcomes can play an important role in implementing and

adopting guideline recommendations.

DOI: 10.1186/1471-2415-10-7

PMCID: PMC2854102

PMID: 20302669 [Indexed for MEDLINE]

405. Curr Med Res Opin. 2010 Mar;26(3):663-74. doi: 10.1185/03007990903554257.

Excluded b/c no oral DMD results

Persistence and adherence to disease modifying drugs among patients with multiple

sclerosis.

Reynolds MW(1), Stephen R, Seaman C, Rajagopalan K.

Author information:

(1)United BioSource Corporation, Lexington, MA, USA.

matthew.reynolds@unitedbiosource.com

OBJECTIVE: This retrospective database study aimed to evaluate the adherence of

multiple sclerosis (MS) patients on immunomodulatory treatments using claims

data, and to identify differences between compliance and persistency measurements

in the context of this disease.

METHODS: Continuously enrolled MS patients treated with subcutaneous IFNbeta-1b

(Betaseron * ), subcutaneous IFNbeta-1a (Rebif dagger ), intramuscular IFNbeta-1a

(Avonex double dagger ), and subcutaneous glatiramer acetate (Copaxone section

sign ).) were identified from the PharMetrics patient-centric database, and all

information related to patient demographics and pharmacy claims for the drugs of

interest were extracted.

OUTCOME MEASURES: The main outcomes were treatment switches and discontinuations

for patients initiated on the drugs of interest. Various compliance and

persistency metrics including the proportion of days covered, treatment

prevalence at 6-monthly time points after initiation, and the continuous time on

drug were also examined.

RESULTS: A total of 6134 MS patients were started on one of the four drugs of

interest. The number of patients switching or discontinuing therapy rose over the

study period. The proportion of patients switching was similar between study

drugs, by the different metrics, with the highest switch rates for subcutaneous

IFNbeta-1b and the lowest for subcutaneous glatiramer acetate. Discontinuation

rates were highest for subcutaneous IFNbeta-1b and lowest for intramuscular

IFNbeta-1a. Regression models showed that intramuscular IFNbeta-1a and

subcutaneous IFNbeta-1a had similar and higher persistency compared to

subcutaneous IFNbeta-1b and subcutaneous glatiramer acetate.

CONCLUSIONS: Although treatment switching and discontinuation is common in MS

patients, there is some noticeable variability between drugs and across measures

of persistency and adherence. Also, claims data do not allow distinguishing

between clinical patterns of MS, direct estimation of disease severity and

observation of care that occurs outside of insurance coverage, and results need

to be cautiously interpreted. The compliance to the various MS drugs was 80% or

higher at all times for all four drugs. The highest rate of treatment persistency

existed in the intramuscular IFNbeta-1a initiator group, while subcutaneous

IFNbeta-1b was associated with a significantly lower persistence (p < 0.0001).

DOI: 10.1185/03007990903554257

PMID: 20070144 [Indexed for MEDLINE]

406. J Med Econ. 2010 Mar;13(1):90-8. doi: 10.3111/13696990903579501.

Excluded b/c no oral DMD results

Healthcare resource utilization following switch or discontinuation in multiple

sclerosis patients on disease modifying drugs.

Reynolds MW(1), Stephen R, Seaman C, Rajagopalan K.

Author information:

(1)Center for Epidemiology and Database Analytics, United BioSource Corporation,

430 Bedford Street, Lexington, MA 02420, USA.

matthew.reynolds@unitedbiosource.com

OBJECTIVE: The objective of this study was to explore the cost and utilization in

the period following discontinuations or switches of disease modifying drugs

(DMDs) for patients with multiple sclerosis (MS). Secondary objectives included

an assessment of the time to switch or discontinuation from index DMD treatment.

METHODS: Cases were defined as a billed MS diagnosis in continuously enrolled

patients initiated with interferon-beta1a IM, interferon-beta1b SC, glatiramer

acetate, and interferon-beta1a SC found in the PharMetrics Patient-Centric

Database. Information on patient demographics, diagnoses, procedures,

pharmacy-dispensed drugs, and costs was extracted; reasons for discontinuation

and expenses outside of the healthcare system were not available. Treatment

discontinuations and switches between study drugs were defined using pharmacy

prescription patterns and analyzed by descriptive and regression methods. The

non-pharmacy medical costs in the 18 months following switching or

discontinuation were compared to the costs in a randomly selected similar period

for those patients who did not switch or discontinue these agents.

RESULTS: A total of 5,772 MS patients were continuously enrolled and were treated

with one or more of the four drugs of interest, and about half of these patients

switched drugs or discontinued treatment for at least 90 days. Patients initiated

with interferon-beta1b SC were more likely to discontinue treatment compared to

interferon-beta1a IM users. Non-pharmaceutical medical costs were highest for

those switching treatments followed by those discontinuing DMDs in the 18 months

following a switch or discontinuation, compared to persistent users of these

drugs. Interferon beta1b SC initiators had higher costs following changes or

discontinuations, while glatiramer acetate and interferon-beta1a SC users had

lower subsequent costs compared to interferon-beta1a IM users.

LIMITATIONS: Unfortunately, the reasons for stopping the initial treatment cannot

be determined from analysis of an administrative claims database. Also, the MS

cases followed in this analysis are billing diagnostic events unconfirmed through

a review of medical records or other data sources. The results are unstratified

in terms of severity and thus while treatment patterns may vary for patients with

different types of MS (e.g., progressive vs. relapsing-remitting), this cannot be

examined in this analysis.

CONCLUSION: Changing or discontinuing DMDs is common among MS patients and is

associated with higher non-pharmaceutical medical costs that vary based on the

initiating drug and other demographics characteristics.

DOI: 10.3111/13696990903579501

PMID: 20078189 [Indexed for MEDLINE]

Excluded b/c no real-world adherence/persistence

407. N Engl J Med. 2010 Feb 4;362(5):387-401. doi: 10.1056/NEJMoa0909494. Epub 2010

Jan 20.

A placebo-controlled trial of oral fingolimod in relapsing multiple sclerosis.

Kappos L(1), Radue EW, O'Connor P, Polman C, Hohlfeld R, Calabresi P, Selmaj K,

Agoropoulou C, Leyk M, Zhang-Auberson L, Burtin P; FREEDOMS Study Group.

Collaborators: Calandra T, DiMarco J, Easton JD, Hudson LD, Kesselring J,

Laupacis A, Temkin N, Weinshenker BG, Zarbin M, Calabresi P, Hohlfeld R, Kappos

L, O'Connor P, Polman C, Beran R, Paine M, MacDonell R, Heard R, Boundy K, Van

Opdenbosch L, Bartholomé E, Pandololfo M, Seeldrayers P, Dubois B, Vande Gaer L,

Decoo D, Mulleners E, Bhan V, Brunet D, Kremenchutzky M, Selchen DH, Lachapelle

J, Christie S, Veloso F, O'Connor P, Devonshire V, Rektor I, Ondrich V, Kanovsky

P, Zapletalová O, Ambler Z, Ehler E, Meluzinova E, Havrdová E, Pazdera L, Vysata

O, Vachova M, Gross-Paju K, Kallela M, Kinnunen E, Sumelahti ML, Eralinna JP,

Airas L, Clavelou P, Moreau T, Vermersch P, Pelletier J, Camu W, Damier P,

Wiertlewski S, Labauge P, Gout O, Lubetzki C, Edan G, De Seze J, Menck S, Haas J,

Einhäupl M, Harms L, Wandinger KP, Zipp F, Kieseier B, Anders D, Berghoff M,

Oschmann P, Rosenkranz T, Heesen C, Bergh FT, Sailer M, Hohlfeld R, Bischoff F,

Marziniak M, Muller M, Kleiter I, Steinbrecher A, Kowalik A, Melms A, Weissert R,

Wiendl H, Karageorgiou C, Fakas N, Maltezou M, Mitsikostas D, Káposzta Z, Rozsa

C, Valikovics A, Kerényi L, Hutchinson M, Milo R, Miller A, Shahien R, Achiron A,

Polman C, Frequin S, Jongen P, Zwanikken C, Hintzen R, Anten B, Hupperts R,

Visser L, Kochanowicz J, Drozdowski W, Fryze W, Wajgt A, Selmaj K, Kozubski W,

Dorobek M, Pniewski J, Czlonkowska A, Kwiecinski H, Stepien A, Panea C, Boeru G,

Perju-Dumbrava L, Zaharia C, Popescu CD, Balasa R, Bejenaru O, Yakupov EZ,

Yakhhno N, Kotov S, Shmyrev V, Zavalishin I, Elchaninov AP, Stolyarov I, Odinak

M, Turcani P, Kurca E, Vyletĕlka J, Heckman J, Isaacs M, Coetzee C, Lycke J,

Hillert J, Olsson T, Kappos L, Gugleta K, Sturzenegger R, Schluep M, Goebels N,

Linnebank M, Irkec C, Karabudak R, Turan OF, Neyal M, Işik N, Sutlas PN,

Akman-Demir G, Siva A, Idiman E, Kocaman A, Zorlu Y, Sevim S, Cottrell D, Silber

E, Barnes D, Bates D, Constantinescu C, Sharrack B, Bendfeldt K, Radue EW.

Author information:

(1)Department of Neurology, University Hospital, University of Basel,

Switzerland. lkappos@uhbs.ch

Comment in

Ann Intern Med. 2010 May 18;152(10):JC5-6, JC5-7, JC5-8.

Expert Opin Pharmacother. 2010 Jul;11(10):1777-81.

Curr Neurol Neurosci Rep. 2010 Sep;10(5):333-5.

N Engl J Med. 2010 May 6;362(18):1738; author reply 1739-40.

N Engl J Med. 2010 Feb 4;362(5):456-8.

BACKGROUND: Oral fingolimod, a sphingosine-1-phosphate-receptor modulator that

prevents the egress of lymphocytes from lymph nodes, significantly improved

relapse rates and end points measured on magnetic resonance imaging (MRI), as

compared with either placebo or intramuscular interferon beta-1a, in phase 2 and

3 studies of multiple sclerosis.

METHODS: In our 24-month, double-blind, randomized study, we enrolled patients

who had relapsing-remitting multiple sclerosis, were 18 to 55 years of age, had a

score of 0 to 5.5 on the Expanded Disability Status Scale (which ranges from 0 to

10, with higher scores indicating greater disability), and had had one or more

relapses in the previous year or two or more in the previous 2 years. Patients

received oral fingolimod at a dose of 0.5 mg or 1.25 mg daily or placebo. End

points included the annualized relapse rate (the primary end point) and the time

to disability progression (a secondary end point).

RESULTS: A total of 1033 of the 1272 patients (81.2%) completed the study. The

annualized relapse rate was 0.18 with 0.5 mg of fingolimod, 0.16 with 1.25 mg of

fingolimod, and 0.40 with placebo (P<0.001 for either dose vs. placebo).

Fingolimod at doses of 0.5 mg and 1.25 mg significantly reduced the risk of

disability progression over the 24-month period (hazard ratio, 0.70 and 0.68,

respectively; P=0.02 vs. placebo, for both comparisons). The cumulative

probability of disability progression (confirmed after 3 months) was 17.7% with

0.5 mg of fingolimod, 16.6% with 1.25 mg of fingolimod, and 24.1% with placebo.

Both fingolimod doses were superior to placebo with regard to MRI-related

measures (number of new or enlarged lesions on T(2)-weighted images,

gadolinium-enhancing lesions, and brain-volume loss; P<0.001 for all comparisons

at 24 months). Causes of study discontinuation and adverse events related to

fingolimod included bradycardia and atrioventricular conduction block at the time

of fingolimod initiation, macular edema, elevated liver-enzyme levels, and mild

hypertension.

CONCLUSIONS: As compared with placebo, both doses of oral fingolimod improved the

relapse rate, the risk of disability progression, and end points on MRI. These

benefits will need to be weighed against possible long-term risks.

(ClinicalTrials.gov number, NCT00289978.)

Copyright 2010 Massachusetts Medical Society

DOI: 10.1056/NEJMoa0909494

PMID: 20089952 [Indexed for MEDLINE]

408. J Neurol. 2010 Feb;257(2):163-70. doi: 10.1007/s00415-009-5359-0.

Excluded b/c no primary data (narrative review)

Development of oral cladribine for the treatment of multiple sclerosis.

Hartung HP(1), Aktas O, Kieseier B, Giancarlo Comi GC.

Author information:

(1)Department of Neurology, Heinrich-Heine-University, Moorenstr. 5, 40225

Dusseldorf, Germany. hans-peter.hartung@uni-duesseldorf.de

Multiple sclerosis (MS) is a chronic immune-mediated disorder of the CNS in which

autoreactive CD4+ and CD8+ T lymphocytes, B lymphocytes, antibodies, macrophages

and cytokines synergize to attack myelin sheaths and injure underlying axons.

Current disease-modifying drugs (DMDs) for MS require regular and frequent

parenteral administration and are associated with limited long-term treatment

adherence. Of all the potential new oral MS agents in development, cladribine is

the only therapy with the potential for short-course dosing. Cladribine is an

immunosuppressant that offers targeted, sustained regulation of the immune system

and that has a well-characterized safety profile, derived from more than 15 years

of use of the parenteral formulation in oncology indications and MS. This paper

discusses the need for new MS therapies to improve treatment adherence, and

reviews the mechanism of action, existing efficacy and safety data, and the

clinical development of oral cladribine. The need for continuous risk monitoring

for all new potent immunoactive drugs under development is emphasized.

Preliminary results of the 96-week, double-blind, randomized, placebo-controlled,

multicenter CLARITY (CLAdRIbine Tablets Treating MS OrallY) study are encouraging

and provide the first complete phase III data on an oral DMD for MS.

DOI: 10.1007/s00415-009-5359-0

PMID: 19921304 [Indexed for MEDLINE]

409. J Neurosci Nurs. 2010 Feb;42(1):40-6.

Excluded b/c no real-world adherence/persistence

Effect of oral antihistamine on local injection site reactions with

self-administered glatiramer acetate.

Pardo G(1), Boutwell C, Conner J, Denney D, Oleen-Burkey M.

Author information:

(1)MS Center of Oklahoma, Mercy's NeuroScience Institute, Oklahoma City, OK USA.

Patients with multiple sclerosis often use injectable medication such as

glatiramer acetate or interferons to treat their disease. Subcutaneous injections

may be associated with local injection site reactions (LISRs), which can include

itching, pain, swelling, or redness. Although not serious, these side effects are

bothersome and can have a negative impact on adherence to the therapeutic

regimen, particularly in early phases of treatment. This randomized parallel

group study of 83 patients with multiple sclerosis who had recently begun

glatiramer acetate therapy investigated whether administration of an oral

antihistamine (cetirizine hydrochloride; Zyrtec, 10 mg) prior to each daily

subcutaneous injection of glatiramer acetate would lower the incidence of LISRs

compared with an oral placebo. Data for the outcome measures were derived from

patient diaries and from the clinic during the baseline and the treatment

periods. The primary outcome measure comparing the number of LISRs at 5 minutes

after injection over 2 weeks was slightly but not significantly lower in patients

who received cetirizine compared with patients who received placebo. Within-group

comparisons showed that there was a significant reduction in mean LISR score from

the 2-week baseline period to the 2-week cetirizine treatment period (0, 2, and 5

minutes after treatment). Both groups showed decreases in the average bothersome

ratings from the baseline to treatment periods. Use of cetirizine did not affect

the type of LISRs that was reported at any time point. There were no safety

concerns with the concurrent administration of cetirizine with glatiramer

acetate. Because there were no statistically significant differences on the

primary end point between patient groups taking cetirizine and those taking

placebo prior to glatiramer acetate injections, cetirizine use as a strategy to

reduce LISRs in patients on glatiramer acetate therapy cannot be recommended at

this time.

PMID: 20187348 [Indexed for MEDLINE]

410. Neurology. 2010 Jan 5;74 Suppl 1:S47-53. doi: 10.1212/WNL.0b013e3181c97f89.

Excluded b/c no primary data (narrative review)

Emerging multiple sclerosis oral therapies.

Rammohan KW(1), Shoemaker J.

Author information:

(1)The MS Center, Columbus, OH 43221, USA. Kottil.Rammohan@osumc.edu

Currently, patients suffering from multiple sclerosis (MS), a chronic

demyelinating disorder of the CNS, must be injected with medication to provide

modest relief for their symptoms. Five orally available therapies are being

evaluated in phase II/III clinical trials. If these therapies prove safe and

tolerable, oral compounds may improve patient endorsement and compliance.

Fingolimod, a novel immunosuppressant, significantly lowered annual relapse rates

in phase II/III trials. Laquinimod, an immunomodulator, reduced the cumulative

number of active lesions at the highest dose tested (0.6 mg/d) in a phase II

trial. Cladribine, another immunomodulator, reduced annual relapse rates by >50%

and gadolinium-positive lesions by >70% at both doses tested in a phase III

trial. Oral fumarate, with immunomodulatory and antioxidant properties, also

lowered the number of lesions in a phase II trial. Finally, teriflunomide, an

immunomodulator, significantly reduced MRI lesion activity and reduced annual

relapse rates in a phase II trial. In this report, we weigh the beneficial

outcomes of these compounds against their risks of adverse effects.

DOI: 10.1212/WNL.0b013e3181c97f89

PMID: 20038763 [Indexed for MEDLINE]

Excluded b/c no oral DMD results

411. Adv Health Econ Health Serv Res. 2010;22:175-93.

Cost sharing, benefit design, and adherence: the case of multiple sclerosis.

Dor A(1), Lage MJ, Tarrants ML, Castelli-Haley J.

Author information:

(1)George Washington University, Washington, DC, USA.

PURPOSE: The authors focus on understanding the relationship between costs and

cost sharing on medication adherence for individuals who initiated a

disease-modifying therapy (DMT) for the treatment of multiple sclerosis (MS).

DMTs reduce the risk of relapse and are an essential component of MS treatment.

Furthermore, the authors compare monthly payment levels for copayments versus

coinsurance and estimate the effects on adherence.

METHODS: Using the MarketScan Commercial Claims and Encounters database evidence

from July 1 2005 to March 31 2008, the authors employ a multivariate two-stage

least-squares model (2SLS) to examine the impact of copayments or coinsurance on

the medication possession ratio (MPR).

FINDINGS: Descriptive results show that the mean out-of-pocket (OOP) costs of DMT

per month were higher for patients with coinsurance than for patients with

copayments. For the cohort of patients with copayment there was little difference

in monthly copayments across adherence thresholds. Regression analysis shows that

an increase in cost sharing reduces adherence overall, but this effect was small

and insignificant in the copayment cohort. In contrast, in the coinsurance cohort

increased cost sharing was significantly associated with decreased adherence to

DMT medication; with a 10% increase in cost sharing leading to an 8.6% decline in

adherence.

IMPLICATIONS: Employers increasingly rely on coinsurance, despite evidence that

reliance on coinsurance results in lower adherence. Our research findings suggest

that coinsurance appears to be a greater obstacle to compliance, confirming

predictions found in the theoretical literature.

ORIGINALITY: This research converted counts of injectable treatments into a

continuous adherence measure. Previous literature on cost sharing did not examine

MS.

PMID: 20575233 [Indexed for MEDLINE]

412. Eur J Neurol. 2010 Jan;17(1):31-7. doi: 10.1111/j.1468-1331.2009.02728.x. Epub

Excluded b/c no real-world adherence/persistence

2009 Jul 9.

Efficacy of natalizumab in second line therapy of relapsing-remitting multiple

sclerosis: results from a multi-center study in German speaking countries.

Putzki N(1), Yaldizli O, Mäurer M, Cursiefen S, Kuckert S, Klawe C, Maschke M,

Tettenborn B, Limmroth V.

Author information:

(1)Department of Neurology, University Clinic Essen, University of

Duisburg-Essen, Germany. norman.putzki@uni-due.de

BACKGROUND: Natalizumab has been recommended for the treatment of

relapsing-remitting multiple sclerosis (RRMS) in patients with insufficient

response to interferon-beta/glatiramer acetate (DMT) or aggressive MS. The

pivotal trials were not conducted to investigate natalizumab monotherapy in this

patient population.

METHOD: Retrospective, multicenter study in Germany and Switzerland. Five major

MS centers reported all RRMS patients who initiated natalizumab >or=12 months

prior to study conduction.

RESULTS: Ninety-seven RRMS patients were included [69% female, mean age 36.5

years, mean Expanded Disability Status Scale (EDSS) 3.4; 93.8% were pre-treated

with DMT], mean treatment duration with natalizumab was 19.3 +/- 6.1 months. We

found a reduction of the annualized relapse rate from 2.3 to 0.2, 80.4% were

relapse free with natalizumab. EDSS improved in 12.4% and 89.7% were progression

free (change of >or= 1 EDSS point). Eighty-six per cent of patients with highly

active disease (>or= 2 relapses in the year and >or= 1 Gadolinium (Gd)+ lesion at

study entry, n = 20) remained relapse free. The mean number of Gd enhancing

lesions was reduced to 0.1 (0.8 at baseline). Discontinuation rate was 8.2% (4.1%

for antibody-positivity).

CONCLUSION: Natalizumab is effective after insufficient response to other DMT and

also in patients with high disease activity.

DOI: 10.1111/j.1468-1331.2009.02728.x

PMID: 19614963 [Indexed for MEDLINE]

413. Eur Neurol. 2010;63(2):101-6. doi: 10.1159/000276400. Epub 2010 Jan 16.

Excluded b/c no real-world adherence/persistence

Natalizumab reduces clinical and MRI activity in multiple sclerosis patients with

high disease activity: results from a multicenter study in Switzerland.

Putzki N(1), Yaldizli O, Bühler R, Schwegler G, Curtius D, Tettenborn B.

Author information:

(1)Department of Neurology at Cantonal Hospital St. Gallen, St. Gallen,

Switzerland. Norman.Putzki@kssg.ch

BACKGROUND: Natalizumab has been recommended for the treatment of

relapsing-remitting multiple sclerosis (RRMS) in patients with insufficient

response to interferon-beta or glatiramer acetate (disease-modifying

treatments-DMT) or in aggressive MS. The pivotal trials were not designed to

investigate natalizumab monotherapy in these patient populations.

AIM: To investigate the efficacy of natalizumab after treatment failure of

previous DMT and in highly active MS.

METHODS: A retrospective, multicenter study in Switzerland. Three major MS

centers reported all RRMS patients who initiated natalizumab>or=12 months prior

to study conduction.

RESULTS: 85 RRMS patients were included (72% female, mean age 37.3 years, mean

Expanded Disability Status Scale 3.1; 88.2% were pretreated with DMT), and mean

treatment duration with natalizumab was 18.4+/-2.6 months. 79% of the patients

were relapse-free during the observational period. The annualized relapse rate

decreased from 2.0+/-0.6 to 0.27+/-0.2, and 92.9% were progression-free after 12

months (p<0.001). The mean number of gadolinium-enhancing lesions decreased from

1.2+/-1.2 to 0.1+/-0.1 at 12 months' follow-up (91.7% reduction).Discontinuation

rate was 11.8% (7.1% for antibody positivity).

CONCLUSIONS: Patients who initiate natalizumab after previous high disease

activity experience a marked reduction of clinical and MRI disease activity.

Copyright (c) 2010 S. Karger AG, Basel.

DOI: 10.1159/000276400

PMID: 20090344 [Indexed for MEDLINE]

414. J Med Econ. 2010;13(4):633-40. doi: 10.3111/13696998.2010.527588. Epub 2010 Oct

Excluded b/c no oral DMD results

19.

Medication adherence with disease modifying treatments for multiple sclerosis

among US employees.

Kleinman NL(1), Beren IA, Rajagopalan K, Brook RA.

Author information:

(1)HCMS Group, Cheyenne, WY, USA.

OBJECTIVE: Medication adherence in chronic diseases like multiple sclerosis (MS)

plays an important role in predicting long-term outcomes, yet existing data on

adherence in employee populations are not found. The objective of this study is

to compare adherence among employees treated with disease modifying treatments

(DMTs) for MS in the year following treatment initiation.

METHODS: A healthcare claims database of US employees from 2001 to 2008 was used

to identify patients with MS based on two or more DMT prescriptions or one DMT

prescription with an MS diagnosis (ICD-9 340.xx). Employees continuously employed

and with health plan coverage for 1 year following DMT initiation were eligible.

Two measures were used in estimating adherence after DMT initiation: (1)

persistence (the number of days from DMT initiation to the first 30-day gap in

supply) and, (2) annual compliance, assessed by the medication possession ratio

(MPR = number of days with a medication supply in the year divided by 365 days).

Wilcoxon tests on time-to-event data and t-tests were used to compare persistence

and MPR, respectively, between DMT groups. Other measures of resource utilization

were also compared.

RESULTS: Overall, 358 employees [179 interferon [IFN]-β1a-IM (Avonex* = 'A'); 63

IFN-β1b (Betaseron†  = 'B'); 20 IFN-β1a-SC (Rebif‡  = 'R'); 96 glatiramer acetate

(Copaxone§  = 'C')] were eligible for analysis. No significant differences in

age, gender, and certain job-related variables existed between cohorts.

Persistence was better for 'A' than 'B' (p = 0.039), 'C' (p = 0.0007), and 'R' (p

= 0.130). At 1 year, a greater proportion of 'A' employees were persistent

(60.34%) than 'B' (42.86%, p = 0.016), 'C' (42.71%, p = 0.0052), and 'R' (45.00%,

p = 0.190). 'A' also had the highest MPR (0.782) which was significantly higher

than 'C' (MPR = 0.698, p = 0.0160) and statistically equivalent to 'B' (MPR =

0.705, p = 0.0576) and 'R' (MPR = 0.761, p = 0.7347).

LIMITATIONS: The study has limitations characteristic of administrative claims

database studies and small sample sizes. The population may not be representative

of undiagnosed/untreated MS patients, those not able to maintain employment, and

those not using the initial therapy.

CONCLUSIONS/RELEVANCE: Among employees treated with 'A', 'B', 'C', and 'R' for

MS, 'A' patients had significantly greater medication adherence.

DOI: 10.3111/13696998.2010.527588

PMID: 20958113 [Indexed for MEDLINE]

415. Mult Scler. 2010 Jan;16(1):112-20. doi: 10.1177/1352458509351897. Epub 2009 Dec

Excluded b/c no oral DMD results

7.

Objective adherence monitoring in multiple sclerosis: initial validation and

association with self-report.

Bruce JM(1), Hancock LM, Lynch SG.

Author information:

(1)Department of Psychology, University of Missouri-Kansas City, Kansas City, KS

64110, USA. brucejm@umkc.edu

Poor adherence to medication is commonplace and contributes to poor health

outcomes among numerous patient populations. Studies that have examined treatment

adherence in multiple sclerosis focus exclusively on retrospective self-reports

and/or imprecise measures of treatment discontinuation. To help address these

methodological limitations, the present longitudinal study compared adherence

outcomes for patients with multiple sclerosis using retrospective self-reports,

adherence diaries, and a novel electronic monitoring device. Sixty-seven patients

with relapsing-remitting multiple sclerosis were followed for a period of eight

weeks during which they used a medication diary and a sharps container that

captured electronically the time and date of each needle disposal. The patients

also reported at the outset and conclusion of the study how frequently they

missed doses. All measures of adherence were highly correlated. Patients reported

better adherence than was indicated by medication diaries and electronic

monitoring of needle disposals. Nearly one-fifth of the sample exhibited poor

adherence, missing more than 20% of their prescribed medication. The results

support the validity of electronic monitoring of needle disposal as an effective

means of measuring adherence to disease modifying therapies in multiple

sclerosis. In contrast, studies employing only self-report may underestimate poor

adherence. Larger scale studies that employ prospective objective methods are

necessary to gain a better understanding of adherence patterns in multiple

sclerosis.

DOI: 10.1177/1352458509351897

PMID: 19995835 [Indexed for MEDLINE]
