# Supplementary figures and images for: Real-world adherence to, and persistence with, once- and twice-daily oral disease-modifying drugs in patients with multiple sclerosis: a systematic review and meta-analysis
Source: BMC Neurol. 2020 Jul 16;20:281. doi: 10.1186/s12883-020-01830-0 (PMC7371467; doi:10.1186/s12883-020-01830-0)

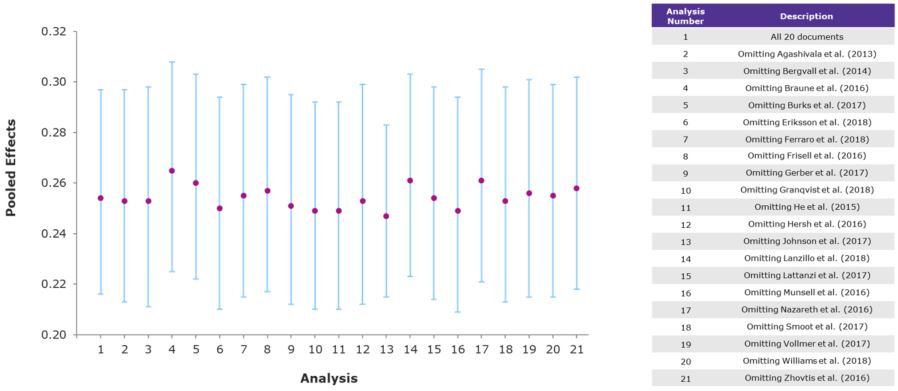

Supplement: Supplementary file 2 — Additional file 2: Supplementary Figure 1. Leave-one-out sensitivity analysis for the proportion of patients discontinuing a DMD. For studies for which results for treatment naive and treatment-experienced patients were reported separately (combined data were not available), data were combined; for studies reporting data for more than 1 oral DMD (combined data were not reported), data were combined; for studies reporting data for subgroups (combined data were not reported), data were combined. Abbreviations: DMD, disease-modifying drug. [file 12883_2020_1830_MOESM2_ESM.jpg]

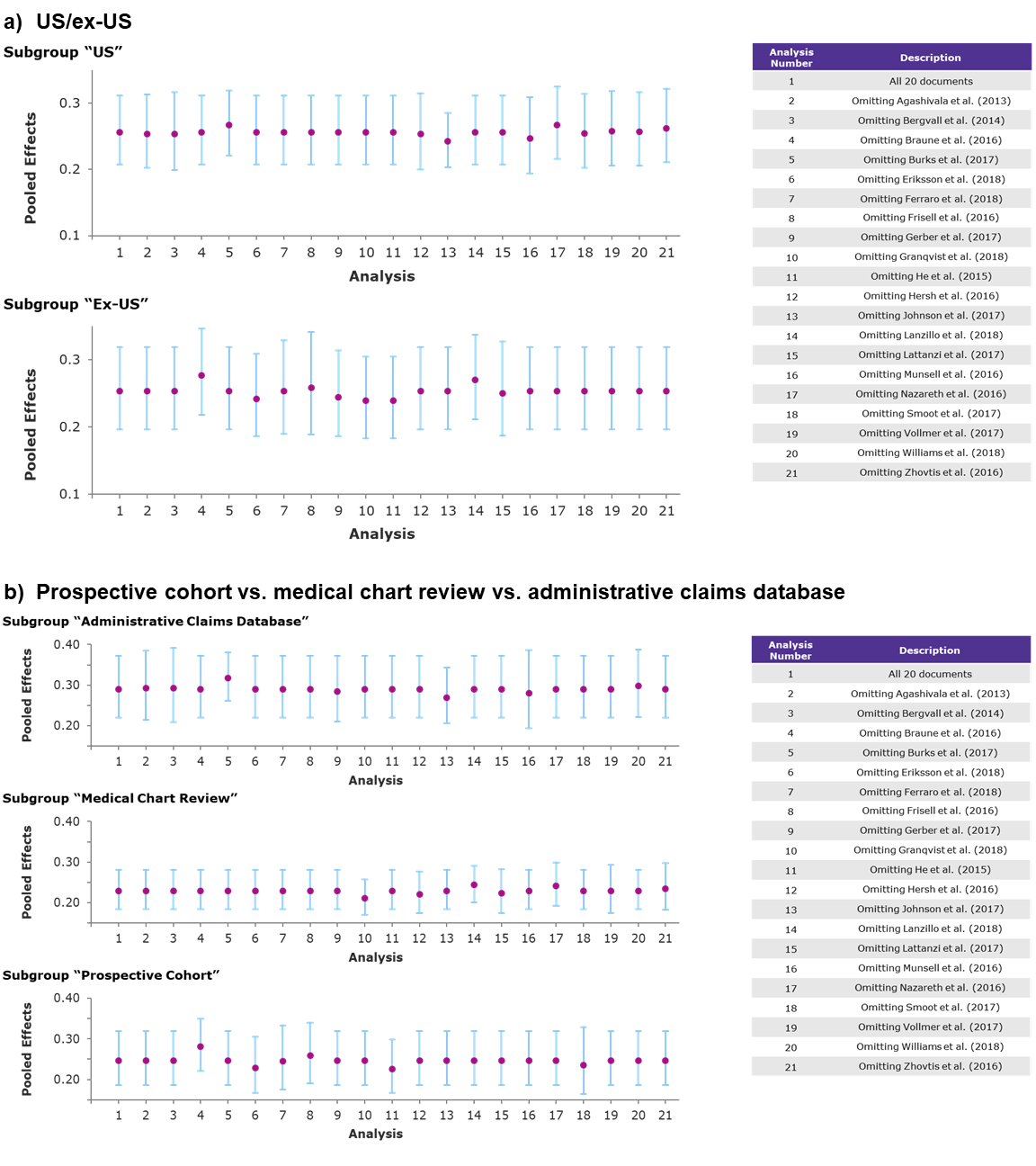

Supplement: Supplementary file 3 — Additional file 3: Supplementary Figure 2. Leave-one-out sensitivity analysis for subgroup analyses for the proportion of patients discontinuing a DMD. For studies for which results for treatment naive and treatment-experienced patients were reported separately (combined data were not available), data were combined; for studies reporting data for more than 1 oral DMD (combined data were not reported), data were combined; for studies reporting data for subgroups (combined data were not reported), data were combined. Abbreviations: DMD: disease-modifying drug. [file 12883_2020_1830_MOESM3_ESM.png]
